# Organophotocatalytic dearomatization of indoles, pyrroles and benzo(thio)furans via a Giese-type transformation

**DOI:** 10.1038/s42004-021-00460-y

**Published:** 2021-02-19

**Authors:** Yueteng Zhang, Peng Ji, Feng Gao, Yue Dong, He Huang, Changqing Wang, Ziyuan Zhou, Wei Wang

**Affiliations:** 1 Departments of Pharmacology and Toxicology and Chemistry and Biochemistry, and BIO5 Institute, University of Arizona, Tucson, AZ USA; 2 Department of Chemistry and Chemical Biology, Cornell University, Ithaca, NY USA; 3 National Clinical Research Centre for Infectious Diseases, Shenzhen Third People’s Hospital, The Second Hospital Affiliated to Southern University of Science and Technology, Shenzhen, China

**Keywords:** Synthetic chemistry methodology, Catalyst synthesis

## Abstract

Accessing fascinating organic and biological significant indolines via dearomatization of indoles represents one of the most efficient approaches. However, it has been difficult for the dearomatization of the electron deficient indoles. Here we report the studies leading to developing a photoredox mediated Giese-type transformation strategy for the dearomatization of the indoles. The reaction has been implemented for chemoselectively breaking indolyl C=C bonds embedded in the aromatic system. The synthetic power of this strategy has been demonstrated by using structurally diverse indoles bearing common electron-withdrawing groups including (thio)ester, amide, ketone, nitrile and even aromatics at either C_2_ or C_3_ positions and ubiquitous carboxylic acids as radical coupling partner with high *trans*-stereoselectivity (>20:1 dr). This manifold can also be applied to other aromatic heterocycles including pyrroles, benzofurans and benzothiophenes. Furthermore, enantioselective dearomatization of indoles has been achieved by a chiral camphorsultam auxiliary with high diastereoselectivity.

## Abstract (web summary)

Dearomatisation of indoles allows efficient access to indolines, but often is incompatible with electron-withdrawing substituents. Here a photoredox Giese-type dearomatisation of indoles yields 2,3-disubstituted indolines bearing electron-withdrawing groups.

## Introduction

The indolines have fascinated organic and medicinal chemists for decades^1–8^. The molecular architecture is a common core featured in numerous natural products, biologically active compounds particularly pharmaceutics and agrochemicals. This biogenically produced privileged structure^9^ provides highly biologically relevant three-dimensional chemical space for effective interaction with biological targets. Therefore, quickly accessing the framework with the capacity of engineering functional and stereochemical diversity can streamline the target- and diversity-oriented synthesis for biological studies and drug discovery.

**Fig. 1:**
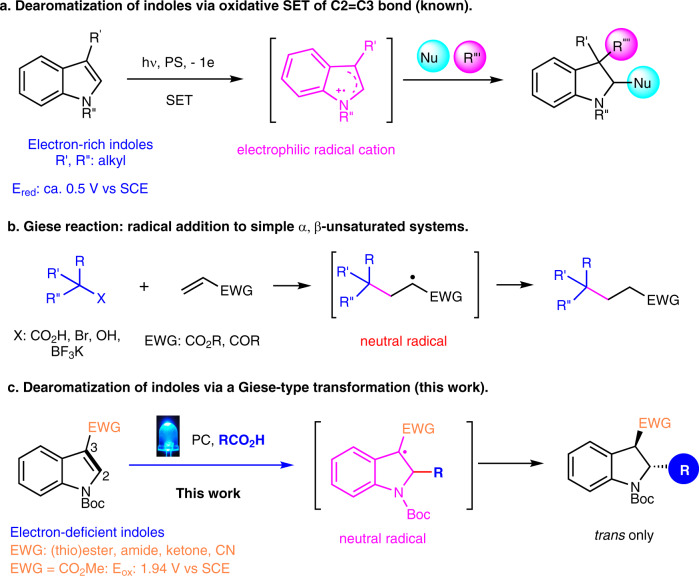
The synthesis of indolines by radical engaged dearomatization of indoles. **a** Dearomatization of indoles via oxidative SET of C2=C3 bond (known). **b** Giese reaction: radical addition to simple α, β-unsaturated systems. **c** Dearomatization of indoles via a Giese-type transformation (this work).

The dearomatization of arenes has become a powerful platform for the facile construction of highly valued molecular architectures^10–15^. The dearomatization of indoles constitutes the most efficient strategy for accessing indolines^1–8^. Indole is an electron-rich aromatic system containing enamine embedded C_2_–C_3_ π bond and strong nucleophilic C_3_ carbon. The reactivity has dictated indole dearomatization methodology development^1–8^ since Woodward’s pioneering study using a Pictet-Spengler type reaction to break the aromatic tryptamine in total synthesis of strychnine in 1954^16^. Impressively, this important array of reactivity from the intrinsically nucleophilic indoles upon activation by various tailored electrophiles has become a powerful manifold for the synthesis of structurally diverse indolines as it enables regioselective reactivity, facile ring formation, and efficient skeleton rearrangement^1–8^. Moreover, this reactivity has been leveraged beyond the 2e transfer pathway. Single-electron transfer (SET) involved oxidation-induced C–H functionalization of the nucleophilic indoles has been elegantly realized as powerful alternatives for indole dearomatization^17^, particularly mild, green visible light photocatalytic and electrochemical methods (Fig. 1a)^3,15,18–29^.

Despite the great success, it has been challenging for the dearomatization of electron-poor indoles, as evidenced by only a handful of examples^30–33^, which rely on ionic activation mode. Indoles bearing electron-withdrawing groups (EWGs) at N, C_2_ or C_3_ positions tend to make the C_2_=C_3_ π bond more difficult to react with electrophilic partners or in an oxidative SET process. The reduced C_2_=C_3_ π bond electron density can be reflected by the significant difference of their redox potentials. For example, E_redox_ of *N*,3-dimethyl indole is ca. +0.4 V vs SCE^34^, while *N*-methyl 3-acetyl indole is ca. +1.0 V vs SCE^34^. Therefore, an unique activation paradigm is needed to address this unmet synthetic challenge.

Giese reaction involving the reductive conjugation addition of radicals to electron-deficient C=C double bonds servers as a powerful tool for new C–C bond formation^35^. The original conditions using stoichiometric amounts of trialkyl tin reagents have motivated organic chemists to develop more practical protocols. Recent efforts on the study of photoredox catalysis under mild reaction conditions have made the process greener and more atom economical (Fig. 1b)^36–47^. In this process, the unsaturated C=C bond is transformed into a saturated C–C bond in a conjugate addition manner. We questioned whether the reaction could be applied for breaking unsaturated C=C bonds embedded in the indole aromatic structure. Specifically, we envisioned the incorporation of an EWG into C_2_ or C_3_ position of indoles, which could be viewed as the Michael acceptors for the Giese type transformation. The successful realization of this process could offer a distinct approach for the dearomatization of less developed electron-deficient indoles and would also expand the scope of the Giese reaction.

However, implementing the strategy faces significant roadblocks. Unlike an isolated C=C bond in a typical Giese reaction (Fig. 1b), breaking the unconventional C=C bond in stable indole aromatic systems overcomes a higher energy barrier. The precedent studies of direct addition of an electrophilic radical to the electron-rich C_2_=C_3_ bond of indoles in electrophilic aromatic substitution processes provide encouraging possibility^48,49^. Nonetheless, the reversed reactivity of the addition of a nucleophilic radical to an electron-poor C_2_=C_3_ bond of indoles is unknown. Moreover, even though incorporation of EWGs into the C_2_ or C_3_ positions of indoles could reverse the polarity from the innate nucleophilic to electrophilic system and serve as a potential radical acceptor, the weakly electron-deficient indoles render the Giese reaction more difficult because more electron deficient, less hindered α, β-unsaturated systems are generally used for effective nucleophilic radical addition^36–47^. Furthermore, in the photoredox process, possible oxidation of the weakly electron-deficient indole systems could complicate the process.

Herein we wish to disclose the results of the investigation, which leads to a photoorganocatalytic strategy for the dearomatization of electron-deficient indoles. An unconventional Giese-type transformation is successfully implemented for the first time (Fig. 1c). Notably, the protocol uses naturally abundant carboxylic acids as radical precursors^50–55^ for reacting with various functionalized indoles bearing common EWGs at either C_2_ or C_3_ positions including (thio)ester, amide, ketone, nitrile, and even neural H and phenyl moieties. Furthermore, this mild dearomatization method displays a broad substrate scope and a wide array of functional group tolerance and thus enables to deliver a wide array of 2,3-disubstituted indolines with high *trans*-stereoselectivity (>20:1 dr). This approach can also be applied to other aromatic heterocycles such as pyrroles, benzofurans, and benzothiophenes for the dearomatization. Finally, enantioselective dearomatization of indoles has been achieved by a chiral camphorsultam auxiliary with high ee (up to 98%).

## Results

### **E**xploration and optimization of the Giese-type reaction

In the initial exploratory studies, we chose *N*-Boc indole methyl ester **1a** as a radical acceptor and Boc-alanine **2a** as a radical precursor for the proposed Giese-type reaction. It is believed that the carboxylate **2a** can be selectively oxidized by the photocatalyst (PC, Ir[dF(CF_3_)ppy]_2_(dtbpy))PF_6_) to give the corresponding radical while the oxidation of the C_2_=C_3_ π bond is difficult because the E_1/2_* is +1.21 V (vs SCE)^50^ of the PC and **2a** salt E_ox_ is around +1.00 V (vs SCE)^56^ while the E_ox_ of the indole methyl ester **1a** is +1.94 V (vs SCE, see Supplementary Methods Section 1.8). Irradiation of a mixture of **1a** (0.2 mmol) with *N*-Boc-Alaine **2a** (0.26 mmol) in the presence of (Ir[dF(CF_3_)ppy]_2_(dtbpy))PF_6_ (5 mol%) and Cs_2_CO_3_ (0.2 mmol) in DMF (0.1 M) under N_2_ atmosphere with a 5 W blue LED strip was performed accordingly (Table 1, entry 1). Indeed, the desired indoline product **3a** was obtained with yield of 56% after 36 h irradiation without the observed oxidation of **1a** (entry 1). It is also noted that the reaction proceeded highly *trans* selectively. Encouraged by the results, we devoted efforts to optimize reaction conditions. When 4CzIPN was used as a PC^57^, a nearly quantitative yield was obtained (entry 2). Probing other parameters such as switching solvent to MeCN (entry 3), shortening time (entry 4) and lowering the amount of base (entry 5) revealed that the reaction performed in DMF for 36 h with 1 equiv. of Cs_2_CO_3_ and 5 mol% catalyst (entry 2) could give the best reaction yield. As expected, PC (entry 7) and visible light (entry 8) were indispensable for this process. These findings led to establishing the optimal protocol used for probing the scope of an organophotocatalytic dearomatization of indoles.

**Table 1:** Optimization of reaction conditions.


Entry	PC (5 mol%)	Solvent (0.1 M)	Time (h)	Yield (%)^a^
1	(Ir[dF(CF_3_)ppy]_2_(dtbpy))PF_6_	DMF	36	56
2	4CzIPN	DMF	36	99^b^
3	4CzIPN	MeCN	36	81
4	4CzIPN	DMF	24	75
5	4CzIPN	DMF	24	83^c^
6^d^	4CzIPN	DMF	36	91^d^
7	None	DMF	36	NR^e^
8	4CzIPN	DMF	36	NR^f^

Unless otherwise specified, to an oven-dried 10 mL-Schlenk tube equipped with a stir bar, was added 1a (55.0 mg, 0.2 mmol), PC (8.0 mg, 5 mol%), 1b (45.5 mg, 0.26 mmol), Cs_2_CO_3_ (65.0 mg, 0.2 mmol) and solvent (2.0 mL). The mixture was degassed by freeze-pump-thaw method, then sealed with parafilm. The solution was then stirred at rt under the irradiation of a 5 W blue LED strip for the indicated time.

*PC* photoredox catalyst, *DMF* dimethylformamide, *NMP* N-Methyl-2-pyrrolidone.

^a^1H NMR yield.

^b^Isolated yield.

^c^0.1 mmol Cs_2_CO_3_ was used.

^d^3 mol% PC was used.

^e^No reaction.

^f^No light.

### Scope of indoles and other heteroaromatics

**Fig. 2:**
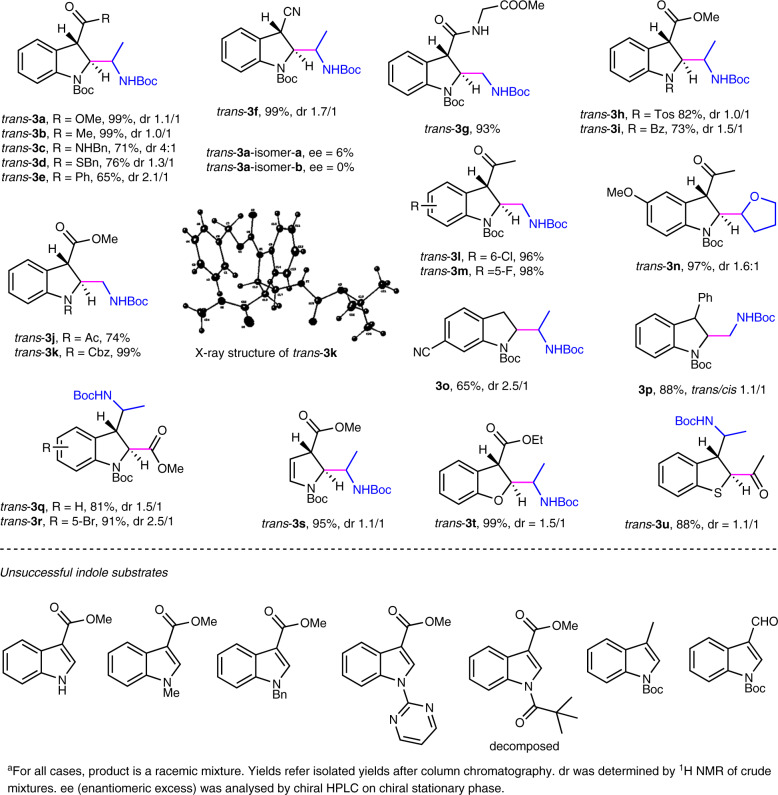
Scope of indoles and other hetereoaromatics. Unless specified, see the general procedure in Supplementary Methods Section 1.5 for the experimental protocol.

With optimized reaction conditions in hand, we first evaluated the radical-engaged dearomatization reactions utilizing various electron-deficient indoles as substrates. As shown in Fig. 2, this methodology serves as a mild and efficient approach for the synthesis of a wide range of 2,3-disubstituted indoline derivatives in high yields (up to 99%) (Fig. 2) (Supplementary Methods Section 1.5 and Supplementary Data 1). Notably, the protocol works for indoles bearing various EWGs beyond ester. Ketone (**3b** and **3c**), amide (**3c** and **3g**), thioester (**3d**) and cyanide (**3f**) can be served to afford broadly functionalized indolines in high yields. Furthermore, commonly used nitrogen protecting groups such as Tos (**3** **h**), Bz (**3i**), Ac (**3j**) and Cbz (**3k**) are tolerated very well. Incorporation of various substituents (e.g., MeO, F, Cl, Br) into the benzene ring in the indole skeleton does not affect dearomatization efficiency (**3l**, **3m**, **3n, 3o, 3q** and **3r**). Unexpectedly, in addition to electron-deficient indoles, the protocol works smoothly for indoles containing electron neutral H (**3o**) and phenyl (**3p**) moieties. Moreover, instead of EWG at C_3_, indole possessing C_2_ ester group also works well (**3q** and **3r**). Remarkably, other aromatic structures such as benzofuran, benzothiophene and pyrrole can attend the process and produce 2,3-dihydro-1*H*-pyrrole (**3s**), 2,3-dihydrobenzofuran (**3t**), and 2,3-dihydrobenzo[*b*]thiophene (**3u**), respectively in high yields. The obtained *trans* products were confirmed by the single X-ray analysis of *trans*-**3k** (CCDC-1994584, Supplementary Methods Section 1.10: Supplementary Tables 1–3 and Supplementary Data 3: CIF file). The unsuccessful indole substrates listed in Fig. 2 suggest that two EWGs are necessary for this process.

### Scope of carboxylic acids

**Fig. 3:**
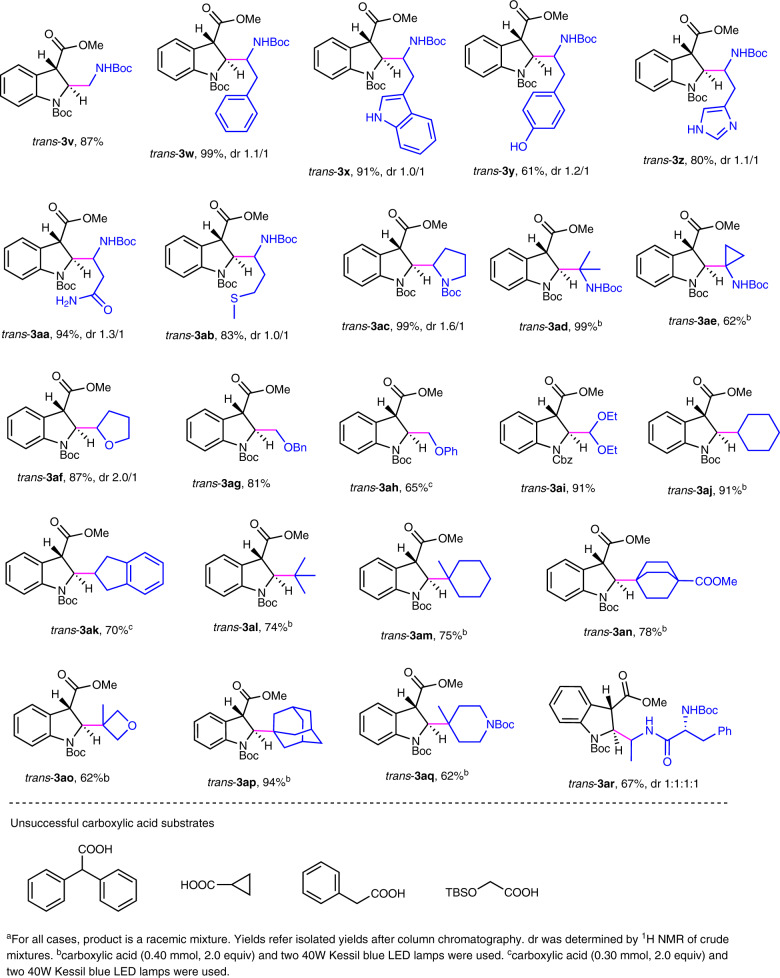
Scope of carboxylic acids. Unless specified, see the general procedure in Supplementary Methods Section 1.5 for the experimental protocol.

Next, we probed the structural alternation of carboxylic acids under the optimal reaction conditions (Fig. 3) (Supplementary Methods Section 1.6 and Supplementary Data 1). Again, this strategy provides a preparative power for the synthesis of various *trans*-selective 2,3-disubstituted indolines on account of their easy availability. Naturally abundant amino acids without requiring protection of side-chain functionalities serve as a good source to install α-amino alkyl groups at C_2_ position of indoline (**3v**-**3ae**). The mild reaction enables incorporation of highly strained structures (**3ae** and **3ao**). In addition, radicals generated from α-oxygen carboxylic acids efficiently engage in the dearomative process of electron-deficient indoles as well (**3af**-**3ai**). Next, non-α-heteroatom alkyl radicals bearing four-, five- and six- rings were probed. The corresponding products **3aj**, **3ak**, **3am**, **3ao** and **3aq** were delivered in high yields. This protocol was also successfully expanded to bridged carboxylic acids (**3an** and **3ap**) as alkyl radical precursors. As for hindered structures and less reactive radicals, stronger light power and a more amount of acid are needed to achieve good yields (**3ad**, **3ae**, **3aj**–**3aq**). It should also be pointed out that under the mild reaction conditions, this radical-based method exhibits broad functional group tolerance, as demonstrated for free hydroxyl (**3y**), acetal (**3ai**), thioether (**3ab**), amide (**3aa**), and heteroaromatic groups (**3x** and **3z**). Especially, electron-deficient indole was chemoselectively reacted in the presence of electron-rich indole, demonstrated by the case of **3x**. Furthermore, a dipeptide can also effectively participate in this process. In the scope study, we found that four unsuccessful carboxylic acids could not partipatite in the process (Fig. 3).

### Late stage dearomatization of indoles and gram-scale synthesis of indolines

**Fig. 4:**
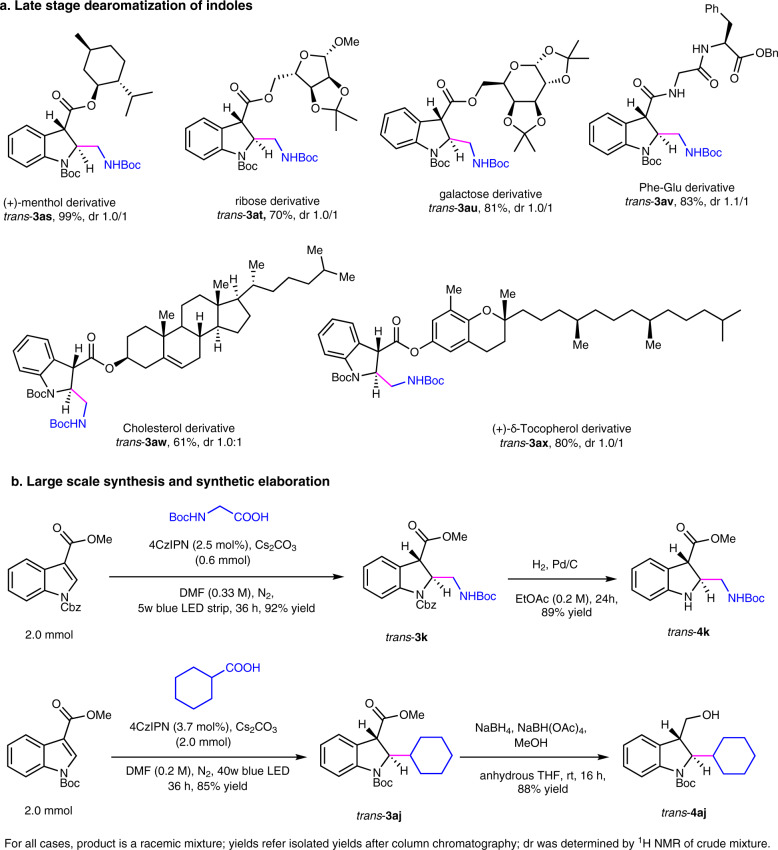
Late-stage dearomatization of indoles and gram-scale synthesis of indolines. **a** Late-stage dearomatization of indoles. **b** Large scale synthesis and synthetic elaboration.

To further demonstrate the utility of this mild dearomatization strategy, we performed a series of late-stage modifications on natural products. As shown in Fig. 4a (Supplementary Methods Section 1.5), the standard protocol was successfully applied to natively and selectively modify natural products (+)-menthol, cholesterol and (+)-δ-tocopherol to give *trans*-indoline-based analogues **3as**, **3aw** and **3ax** in 99, 61 and 80% yield, respectively. Moreover, pentose and hexose derived indoles gave the desired products **3at** − **3au**. Finally, *trans*-indoline containing dipeptide **3av** was prepared in high yield (83%). This approach can be applied in a gram scale (2 mmol) synthesis of indolines without loss of yields (Figs. 4b, 3k and **3aj**) (Supplementary Methods Section 1.5). Moreover, the obtained indolines can go further transformation such as deprotection of *N*-Boc indoline and reduction of methyl ester to alcohol (**4k** and **4aj**) (Supplementary Methods Section 1.5 and Supplementary Data 1).

### **A**symmetric dearomatization of indoles

With the success of efficient radical engaged dearomatization of indoles and other heteroaromatics, we seek to realize the asymmetric version of this synthetically useful approach. The development of radical engaged reactions including asymmetric dearomatization of indoles has been a formidably challenging task in photoredox catalysis and is much less developed, but highly sought area^58,59^. It is observed that so far, all reported but limited asymmetric examples employ the electron-rich indoles^18,22,60–62^. In this study, we proposed to realize the asymmetric manner using a chiral auxiliary induced chirality strategy because the carboxylate can be served as a handle for the incorporation of a chiral auxiliary^63^. However, they are rarely employed in radical processes^64,65^ probably because of the high reactivity of radial species.

Commonly used Evans chiral oxazolidinone auxiliary^66^ was explored in our initial attempt. Under the above established optimal reaction conditions, the reaction of chiral oxazolidinone (**5a**) with *N*-Boc alanine (**2b**) gave the product in quantitative yield but only poor distereoselectivity (1.6:1 dr, Table 2, entry 1). It appears that solvent has little effect on the distereoselectivity (entries 1–3), but noticed impact on reaction yield (entries 1, 2 and 5). As Lewis acid (LA) is often used to improve dr by reducing the rotation of the auxiliary through chelating two C = O bonds, we screened several LAs. However, no enhancement of dr value was observed, which probably attributes to the disruption of the chelation interaction by highly polar solvent DMF (entries 6–9). Switching to *L*-proline derived indole **5b** did not produce an encouraging result (entry 10). Gladly, when chiral camphorsultam^67^ was employed as a chiral auxiliary, excellent dr value (>20:1) was obtained in high yield (99%, entry 11). Shortening reaction time decreased the yield slightly (entry 12). These studies led to a protocol for the asymmetric dearomatization of electron-deficient indoles for the first time and provide an efficient approach to the synthesis of medicinally valued chiral indoline derived amino acids (β- and/or γ-amino acids).

**Table 2:** Optimization of asymmetric dearomatization reaction conditions.


Entry	Substrate	Additive	Solvent (0.1 M)	Yield(%)^a^	Dr^b^
1	**5a**	None	DMF	99^c^	1.6:1
2	**5a**	None	MeCN	78	1.3:1
3	**5a**	None	NMP	99	1.6:1
4^d^	**5a**	None	DMF	99	1.8:1
5^d^	**5a**	None	EtOAc	<5	Not determined
6	**5a**	BF_3_ (10 mol%)	DMF	61	1.4:1
7^e^	**5a**	Zn(OAc)_2_ (1.0 equiv.)	DMF	<5	Not determined
8^e^	**5a**	Mg(OAc)_2_ (1.0 equiv.)	DMF	52	1.5:1
9^e^	**5a**	LiOAc (1.0 equiv.)	DMF	65	1.4:1
10	**5b**	None	DMF	77	1.1:1
11	**5c**	None	DMF	99^c^	>20:1
12	**5c**	None	DMF	91%^f^	>20:1

Unless otherwise specified, to an oven-dried 10 mL-Schlenk tube equipped with a stir bar, was added **5** (0.1 mmol), photoredox catalyst (4CzIPN, 4.0 mg, 5 mol%), **2b** (23.0 mg, 0.13 mmol), Cs_2_CO_3_ (32.0 mg, 0.1 mmol) and solvent (1.0 mL). The mixture was degassed by freeze-pump-thaw method, then sealed with parafilm. The solution was then stirred at rt under the irradiation of a 5 W blue LED strip for 36 h.

*DMF* dimethylformamide, *NMP* N-Methyl-2-pyrrolidone.

^a1^H NMR yield.

^b^Determined by ^1^H NMR.

^c^Isolated yield.

^d^CsOAc (1.0 equiv.) used as base.

^e^No base used.

^f^Reaction time: 24 h.

**Fig. 5:**
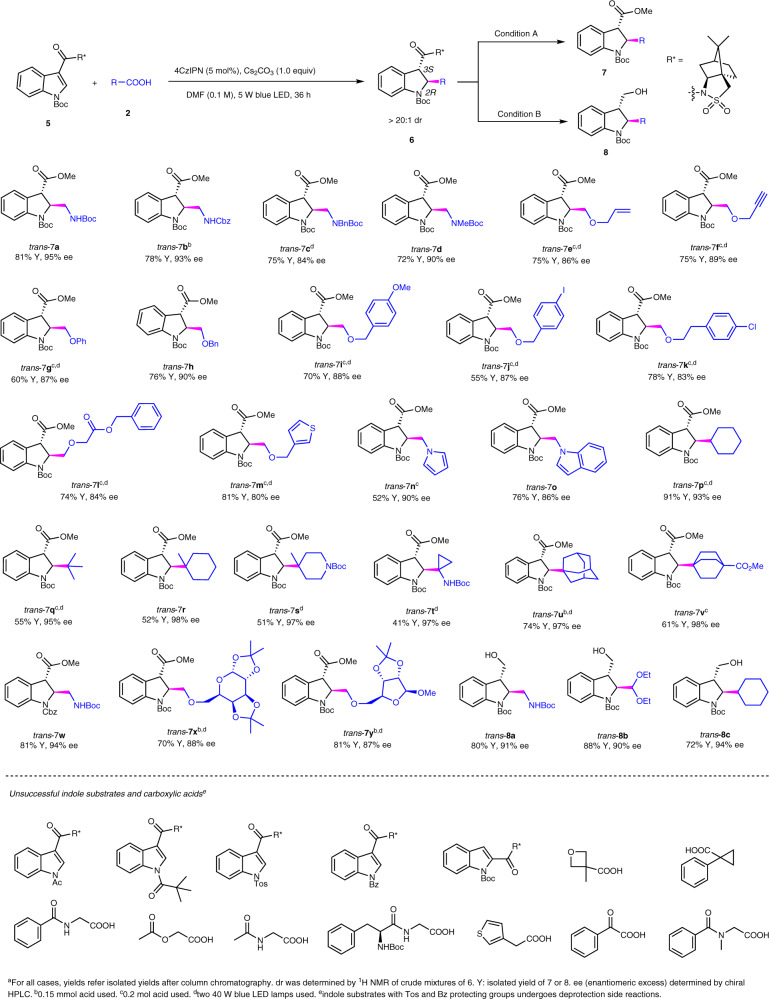
Scope of asymmetric dearomatization of indoles. Unless specified, see the general procedure in Supplementary Methods Section 1.6 for the experimental protocol.

Next, we evaluated the scope of the asymmetric process under the optimized reaction conditions (Fig. 5) (Supplementary Methods Section 1.6 and Supplementary Data 1 and 2). To get more accurate dr value of dearomatization indoline products, the camphorsultam auxiliary was removed by either hydrolysis (Condition A) or reduction (Condition B) to analyze the ee value of derived compounds such as ester or alcohol using chiral HPLC. The results reveal that this radical engaged asymmetric strategy serves as a general approach to enantioenriched *trans* 2,3-disubstituted indolines **7** with high enantioselectivity (up to 98% ee). α-Amino alkyl groups were successfully introduced to deliver pharmaceutically valued chiral β,γ-diamino acids with both high ee values and yields by using natural amino acids (**7a**–**d**). Besides, radicals produced from α-oxygen carboxylic acids reacted with indole substrates smoothly (**7e**–**m**). Notable, various functional groups were tolerated well under the reaction conditions such as allylic (**7e**), propargyl (**7f**), benzyl (**7h**) and halogens (**7j** and **7k**). Moreover, indolines bearing heteroaromatics were successfully obtained (**7m**–**o**). Again, the radical approach enables the incorporation of highly sterically demanding structures (**7p**–**v**), which are particularly challenging using catalytic ionic methods with high level of enantioselectivity. We also probed indoles with different protecting groups (PGs) on nitrogen. It was found that Boc and Cbz (**7w**) were untouched while other PGs such as Ac, Bz, Tos, and pivaloyl were sensitive to the reaction conditions. It seems that chiral moieties do not affect the newly formed stereogentic centers, as seen in the synthesis of chiral indoline saccharide derivatives with both good ee value and yields (**7x** and **7y**). The gram scale (1 mmol) synthesis of **8c** was also realized in 72% yield and 94% ee and the camphorsultam auxiliary was recovered at the same time (81% yield) (Supplementary Methods Section 1.7). The absolute conformation of the products **7** and **8** were determined to 2*R* and 3*S* by converting a known chiral compound (Supplementary Methods Section 1.7)^68^. In all cases except products **8a–c** as alcohols, chiral methyl esters **7** were used for chiral HPLC analysis. Finally, unsuccessful substrates provided in Fig. 5 reveal the limitations of this methodology.

## Discussion

A Giese-type process has been successfully implemented for the dearomatization of electron-poor indole systems in this study. The method can also be viewed as a decarboxylative Michael addition process, which has been intensively studied in recent years^35,50–55^. However, indoles are not the same as simple alkenes and to the best of our knowledge, breaking the stable C_2_=C_3_ bond embedded in the aromatic indoles with this strategy has not been reported. Furthermore, in a typical decarboxylative Michael addition process^35,50–55^, less hindered electron-deficient vinyls are generally used for effective transformation, whereas this process accomplishes with more complicated α, β-unsaturated systems. Therefore, the process significantly expands the scope of the synthetic strategy. Moreover, the process offers a distinct approach to highly vauled indolines. The dearomative structures are complementary to those of well studied electron-rich systems. The tethered EWGs such as ester, ketone, amide, nitrile, etc are versatile handles for further synthetic elaboration.

**Fig. 6:**
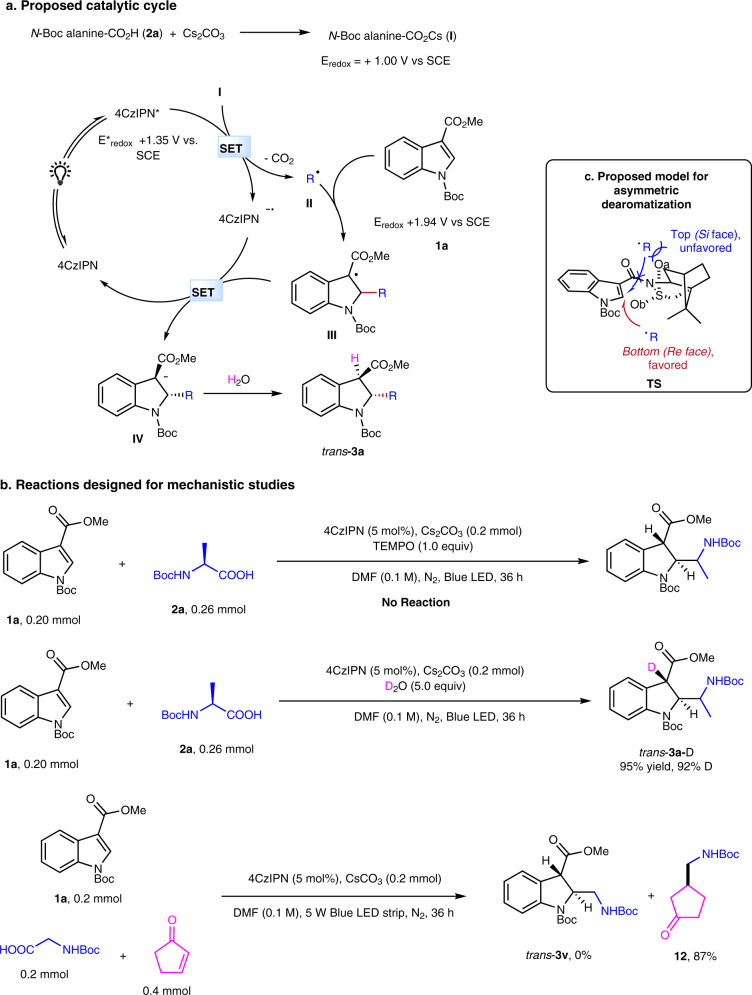
Proposed reaction mechanism and mechanistic studies. See Supplementary Methods Section 1.9 for the experimental protocol.

It is noted that although intermolecular radical addition to the indole C_2_=C_3_ double bond has been documented^48,49^, these processes also use electron-rich structures. Mechanistically, our dearomatization activation strategy is completely different from that of these radical engaged Friedel-Crafts type methods^48,49^. In our approach, an oxidative process is implemented to generate a nucleophilic radical for an addition to an electron-deficient C=C bond (Fig. 6a). The process delivers a dearomative product. In contrast, an opposite photoredoxcatalytic reductive activation produces an electrophilic radical for reacting with the electron-rich indole C_2_=C_3_ double bond. Therefore, an aromatic product is obtained instead via subsequent oxidation to accomplish the catalytic cycle^48,49^.

What we learned from this study is that the successful realization of the distinct indole dearomatization method lies in the rationalization of the reactivities of the radical species in organophotoredox catalytic cycle and enables achieving the chemoselectivity. In the intensively studied photoredox mediated indole dearomatization processes, direct oxidation of electron-rich indoles can be achieved because they have relatively low redox potentials. For example, E_redox_ of *N*,3-dimethyl indole is ca. +0.4 V vs SCE^34^. However, indoles bearing EWGs at N, C_2_ or C_3_ positions have much higher E_redox_. For instance, *N*-methyl 3-acetyl indole is ca. +1.0 V vs SCE^34^ while methyl *N*-Boc-3-indole carboxylate (**1a**, E_redox_ + 1.94 vs SCE, see Supplementary Methods Section 1.8) is even bigger. The significant difference suggests oxidative dearomatization of electrophilic indoles is difficult. The overlooked, reversed reactivity offers a unique opportunity for developing distinct dearomative methods, which have been successfully carried out in this study. Critically, the selective controlling reactivity makes the process possible (Fig. 6a). The excited 4CzIPN* (E*_redox_ + 1.35 vs SCE)^57^ can selectively oxidize *N*-Boc-alanine Cs salt **I** (as a representative example, E_redox_ + 1.00 vs SCE)^56^ without crossover oxidation of methyl *N*-Boc-3-indole carboxylate (**1a**, E_redox_ + 1.94 vs SCE). The resulting nucleophilic radical **II** then undergoes a Giese-type addition process with **1a** to produce radical **III**. The radical **III** is facial selectively reduced by 4CzIPN^•−^ to give the *trans*- anion **IV** and 4CzIPN to complete the redox cycle. Finally, protonation of anion **IV** delivers observed *trans*-dearomatization product **3a**. The pathway is consistent with the photoredox Giese reaction. Furthermore, our control experiments support the proposed pathway (Supplementary Methods Section 1.9). The radical engaged process is verified by a radical scavenger TEMPO suppressed reaction (Fig. 6b). The anion intermediate **IV** is validated by a D_2_O quenching experiment. To get more insights of the reactivity of **1a** compared with the classic Michael acceptor in Giese reaction, a competition experiment using **1a** and cyclopent-2-en-1-one reacting with *N*-Boc-glycine was performed. Interestingly, we didn’t observe *trans*-**3v**, but only product **12** coming from cyclopent-2-en-1-one with a yield of 87%. This result shows the much less reactivity of **1a** than commonly used electron-deficient olefins.

Besides, we were interested in how the chiral auxilary induces diasteroselectivity. Based on Curran’s work^69,70^, a model for the asymmetric dearomatization was proposed (Fig. 6c). The sulfonyl group, which is spatially located close to C2, is believed to play a major role in the inducement of asymmetric selectivity. Radical attacking C2 from the *Re* face(bottom) is favored due to less steric interaction between radical and equatorial β oxygen of the sulfonyl group. While axial α oxygen blocks the radical addition from the *Si* (top) face because of their strong steric interaction. The resulting configuration of the product from this model is conistent with our experimental results.

## Conclusion

In summary, we have developed an unprecedented versatile organophotoredox process for the dearomatization of electron-poor indoles. A distinct strategy that a photoredox mediated Giese-type transformation is introduced to break the electron-deficient aromatic C=C bonds for the first time. The preparative power of the dearomatization strategy has been demonstrated by the use of naturally abundant carboxylic acids and readily available structurally diverse indoles bearing common EWGs including (thio)ester, amide, ketone, nitrile, and even aromatics at either C_2_ or C_3_ positions. A wide array of 2,3-disubstituted indolines with high anti-stereoselectivity (>20:1 dr) are prepared. This powerful manifold can also be applied to other aromatic heterocycles such as pyrroles, benzofurans, and benzothiophenes for dearomatization. Furthermore, enantioselective dearomatization of indoles has been achieved by a chiral camphorsultam auxiliary with high enantioselectivity (up to 98% ee). The simplicity, efficiency, and board scope of this distinct synthetic strategy will be appreciated by organic and medicinal chemists to rapid access a library of synthetically and biologically important indolines.

## Methods

### Typical procedure for the synthesis of racemic products 3 (Figs. 2–4)

To an oven-dried 10 mL-Schlenk tube equipped with a stir bar, was added indole derivatives (0.2 mmol, 1.0 equiv.), 4CzIPN (8.0 mg, 5 mol%), acid (0.26 mmol, 1.3 equiv.), Cs_2_CO_3_ (65.0 mg, 0.2 mmol, 1.0 equiv.) and DMF (2.0 mL). The mixture was degassed by freeze-pump-thaw method, then sealed with parafilm. The solution was then stirred at room temperature under the irradiation of a 5w blue LED strip or two 40 w blue LED lamps for 36 h. After completion of the reaction, the mixture was diluted with 20 mL of water and extracted by EtOAc (3 × 10 mL). The organic layer was collected, dried by Na_2_SO_4_ and concentrated under vacuum. The residue was purified by flash column chromatography to afford the product.

### Typical procedure for the synthesis of products 7 and 8 (Fig. 5)

To an oven-dried 10 mL-Schlenk tube equipped with a stir bar, was added chiral auxiliary attached indole derivatives 5 (0.1 mmol, 1.0 equiv.), photocatalyst 4CzIPN (4.0 mg, 5 mol%), acid (0.13–0.2 mmol, 1.3–2.0 equiv.), Cs_2_CO_3_ (32.0 mg, 0.1 mmol, 1.0 equiv.) and DMF (1.0 mL). The mixture was degassed by freeze-pump-thaw method, then sealed with parafilm. The solution was then stirred at room temperature under the irradiation of a 5w blue LED strip or two 40 w blue LED lamps for the indicated time. After completion of the reaction, the mixture was diluted with 10 mL of water and extracted by EtOAc (3 × 5 mL). The combined organic layers were washed by 10 mL of brine, dried by Na_2_SO_4,_ and concentrated under vacuum. The residue was used in the next step without purification.

#### Condition A

the residue was dissolved in 0.8 mL of THF and 0.2 mL of water. To the solution was added LiOH (12 mg, 0.5 mmol) and H_2_O_2_ (113 μL, 30% (w/w) in water) at rt. The reaction was stirred at rt for 10 h, after which 10 mL of EtOAc was added. The mixture was washed by 0.5 M NaOH (3 × 5 mL). The combined aqueous layers were collected, washed once with 5 mL of Et_2_O and acidified with 2 M HCl to pH = 2–3. The aqueous solution was extracted by EtOAc (3 × 5 mL). The combined organic layers were dried by Na_2_SO_4_ and concentrated under a vacuum. The residue was used in next step without purification. The residue was dissolved in 0.75 mL of Et_2_O and 0.25 mL of MeOH. To the solution was added (trimethylsilyl)diazomethane solution (2 M, 110 μL) at 0 °C under nitrogen atmosphere, the mixture was stirred at room temperature for 20 min. The reaction mixture was concentrated in vacuo and purified by flash column chromatography or preparative TLC plate to afford the product.

#### Condition B

The residue was dissolved in 0.75 mL of EtOH and 0.25 mL of Et_2_O. To the solution was added LiCl (21 mg, 0.5 mmol) and NaBH_4_ (31.5 mg, 0.5 mmol) at rt. The reaction was stirred at rt for 3 h, after which the solution was concentrated and purified by column chromatography or preparative TLC plate to afford the product.

## Supplementary information

Peer Review File

Supplementary Information

Description of Additional Supplementary Files

Supplementary Data 1

Supplementary Data 2

Supplementary Data 3

## Data Availability

^1^H and ^13^C NMR spectra for products **3**, **7**, and **8**: see Supplementary Figs. 3–192 in Suppmentary Data 1. Chiral HPLC analysis for products **7**, **8**, and **11**: see Supplementary Figs. 193–254 in Suppmentary Data 2. The authors declare that all the other data including Supplementary Methods and compound structural characterization data, supporting the findings of this study are available within this paper, its Supplementary Information file. The X-ray crystallographic coordinates (Supplementary Methods: Supplementary Tables 1–3 and Supplementary Data 3: CIF file) for structures *trans*-3k reported in this study have been deposited at the Cambridge Crystallographic Data Centre (CCDC), under deposition numbers CCDC-1994584. These data can be obtained free of charge from The Cambridge Crystallographic Data Centre.

## References

[1] Roche SP, Tendoung J-JY, Tréguier B (2015). Advances in dearomatization strategies of indoles. Tetrahedron.

[2] Zheng C, You SL (2019). Catalytic asymmetric dearomatization (CADA) reaction-enabled total synthesis of indole-based natural products. Nat. Prod. Rep..

[3] Chen J-B, Jia Y-X (2017). Recent progress in transition-metal-catalyzed enantioselective indole functionalizations. Org. Biomol. Chem..

[4] Steven A, Overman LE (2007). Total synthesis of complex cyclotryptamine alkaloids: Stereocontrolled construction of quaternary carbon stereocenters. Angew. Chem. Int. Ed..

[5] Festa AA, Voskressensky LG, Van der Eycken EV (2109). Visible light-mediated chemistry of indoles and related heterocycles. Chem. Soc. Rev..

[6] Bandini M, Eichholzer A (2009). Catalytic functionalization of indoles in a new dimension. Angew. Chem. Int. Ed..

[7] Zhang D, Song H, Qin Y (2011). Total synthesis of indoline alkaloids: a cyclopropanation strategy. Acc. Chem. Res..

[8] Hua T-B, Xiao C, Yang Q-Q, Chen J-R (2019). Recent advances in asymmetric synthesis of 2-substituted indoline derivatives. Chin. Chem. Lett..

[9] Xu W, Gaviab DJ, Tang Y (2014). Biosynthesis of fungal indole alkaloids. Nat. Prod. Rep..

[10] Roche SP, Porco JA (2011). Dearomatization strategies in the synthesis of complex natural products. Angew. Chem. Int. Ed..

[11] Zheng C, You S-L (2016). Catalytic asymmetric dearomatization by transition-metal catalysis: a method for transformations of aromatic compounds. Chem.

[12] Wertjes WC, Southgate EH, Sarlah D (2018). Recent advances in chemical dearomatization of nonactivated arenes. Chem. Soc. Rev..

[13] Fan L, Liu J, Bai L, Wang Y, Luan X (2017). Rapid assembly of diversely functionalized spiroindenes by a three-component palladium-catalyzed C−H amination/phenol dearomatization domino reaction. Angew. Chem. Int. Ed..

[14] Bartoli G, Bencivenni G, Dalpozzo R (2010). Organocatalytic strategies for the asymmetric functionalization of indoles. Chem. Soc. Rev..

[15] Okumura M, Sarlah D (2020). Visible-light-induced dearomatizations. Eur. J. Org. Chem..

[16] Cannon JS, Overman LE (2012). Is there no end to the total syntheses of strychnine? Lessons learned in strategy and tactics in total synthesis. Angew. Chem. Int. Ed..

[17] Nagaraju K, Ma D (2018). Oxidative coupling strategies for the synthesis of indole alkaloids. Chem. Soc. Rev..

[18] Gentry EC, Rono LJ, Hale ME, Matsuura R, Knowles RR (2018). Enantioselective synthesis of pyrroloindolines via noncovalent stabilization of indole radical cations and applications to the synthesis of alkaloid natural products. J. Am. Chem. Soc..

[19] An J, Zou Y-Q, Yang Q-Q, Wang Q, Xiao W-J (2013). Visible light-induced aerobic oxyamidation of indoles: a photocatalytic strategy for the preparation of tetrahydro-5h-indolo[2,3-b]quinolinols. Adv. Synth. Catal..

[20] Wu K, Du Y, Wang T (2017). Visible-light-mediated construction of pyrroloindolines via an amidyl radical cyclization/carbon radical addition cascade: rapid synthesis of (+/−)-flustramide B. Org. Lett..

[21] Muhmel S, Alpers D, Hoffmann F, Brasholz M (2015). Iridium(III) photocatalysis: a visible-light-induced dearomatizative tandem [4+2] cyclization to furnish benzindolizidines. Chem. –Eur. J..

[22] Liang K (2018). Enantioselective radical cyclization of tryptamines by visible light-excited nitroxides. J. Org. Chem..

[23] Zhu M, Zheng C, Zhang X, You S-L (2019). Synthesis of cyclobutane-fused angular tetracyclic spiroindolines via visible-light-promoted intramolecular dearomatization of indole derivatives. J. Am. Chem. Soc..

[24] Zhang M, Duan Y, Li W, Cheng Y, Zhu C (2016). Visible-light-induced aerobic dearomative reaction of indole derivatives: access to heterocycle fused or spirocyclo indolones. Chem. Commun..

[25] Wu K, Du Y, Wei Z, Wang T (2018). Synthesis of functionalized pyrroloindolines via a visible-light-induced radical Cascade reaction: rapid synthesis of (+/−)-flustraminol B. Chem. Commun..

[26] Alpers D, Gallhof M, Witt J, Hoffmann F, Brasholz M (2017). A photoredox-induced stereoselective dearomative radical (4+2)-cyclization/1,4-addition cascade for the synthesis of highly functionalized hexahydro-1H-carbazoles. Angew. Chem., Int. Ed..

[27] Ma J (2020). Gadolinium photocatalysis: dearomative [2+2] cycloaddition/ring-expansion sequence with indoles. Angew. Chem. Int. Ed..

[28] Liu K (2020). Electrooxidation enables highly regioselectivedearomative annulation of indole and benzofuran derivatives. Nat. Commun..

[29] Wu J, Dou Y, Guillot R, Cyrille Kouklovsky C, Vincent G (2019). Electrochemical dearomative 2,3-difunctionalization of indoles. J. Am. Chem. Soc..

[30] Morimoto N, Morioku K, Suzuki H, Takeuchi Y, Nishina Y (2016). Lewis acid and fluoroalcohol mediated nucleophilic addition to the C2 position of indoles. Org. Lett..

[31] Wang L, Shao Y, Liu Y (2012). Nucleophilic addition of Grignard reagents to 3-acylindoles: Stereoselective synthesis of highly substituted indoline scaffolds. Org. Lett..

[32] Nandi RK (2018). Triflic acid as an efficient Brønsted acid promoter for the umpolung of N-Ac indoles in hydroarylation reactions. Adv. Synth. Catal..

[33] Hill JE, Lefebvre Q, Fraser LA, Clayden J (2018). Polycyclic indoline derivatives by dearomatizing anionic cyclization of indole and tryptamine-derived ureas. Org. Lett..

[34] Colonna, M. et al. A correlation between half-wave and ionization potentials for indoles and indolizines. *J. Chem. Soc. Perkin Trans*. II 1229-1231 (1986).

[35] Giese B (1983). Formation of C-C bonds by addition of free radicals to alkenes. Angew. Chem. Int. Ed. Engl..

[36] Jasperse CP, Curran DP, Fevig TL (1991). Radical reactions in natural product synthesis. Chem. Rev..

[37] Protti S, Dondi D, Fagnoni M, Albini A (2009). Assessing photochemistry as a green synthetic method. Carbon–carbon bond forming reactions. Green. Chem..

[38] Ryu I, Uehara S, Hirao H, Fukuyama T (2008). Tin-free Giese reaction and the related radical arbonylation using alkyl iodides and cyanoborohydrides. Org. Lett..

[39] Chu L, Ohta C, Zuo Z, MacMillan DWC (2014). Carboxylic acids as a traceless activation group for conjugate additions: a three-step synthesis of (±)-pregabalin. J. Am. Chem. Soc..

[40] Qin T (2017). Nickel-catalyzed Barton decarboxylation and Giese reactions: a practical take on classic transforms. Angew. Chem. Int. Ed..

[41] Millet A, Lefebvre Q, Rueping M (2016). Visible-light photoredox-catalyzed Giese reaction: decarboxylative addition of amino acid derived α-amino radicals to electron-deficient olefins. Chem. Eur. J..

[42] Ravelli D, Montanaro S, Zema M, Fagnoni M, Angelo Albini AA (2011). Tin-free, radical photocatalyzed addition to vinyl sulfones. Adv. Synth. Catal..

[43] Liu H (2018). One-pot photomediated Giese reaction/Friedel–Crafts hydroxyalkylation/oxidative aromatization to access naphthalene derivatives from toluenes and enones. ACS Catal..

[44] ElMarrouni A, Ritts CB, Balsells J (2018). Silyl-mediated photoredox-catalyzed Giese reaction: Addition of non-activated alkyl bromides. Chem. Sci..

[45] Kanegusuku ALG, Castanheiro T, Ayer SK, Roizen JL (2019). Sulfamyl radicals direct photoredox-mediated Giese reactions at unactivated C(3)-H bonds. Org. Lett..

[46] Lee GS, Hong SH (2018). Formal Giese addition of C(sp3)–H nucleophiles enabled by visible light mediated Ni catalysis of triplet enone diradicals. Chem. Sci..

[47] Ramirez NP, Jose C, Gonzalez-Gomez JC (2017). Decarboxylative Giese-type reaction of carboxylic acids promoted by visible light: a sustainable and photoredox-neutral protocol. Eur. J. Org. Chem..

[48] Furst L, Matsuura BS, Narayanam JMR, Tucker JW, Stephenson CRJ (2010). Visible light-mediated intermolecular C-H functionalization of electron-rich heterocycles with malonates. Org. Lett..

[49] O’Brien CJ (2018). Photoredox cyanomethylation of indoles: catalyst modification and mechanism. J. Org. Chem..

[50] Prier CK, Rankic DA, MacMillan DWC (2013). Visible light photoredox catalysis with transition metal complexes: applications in organic synthesis. Chem. Rev..

[51] Tellis JC (2016). Single-electron transmetalation via photoredox/nickel dual catalysis: unlocking a new paradigm for sp3-sp2 cross-coupling. Acc. Chem. Res..

[52] Romero NA, Nicewicz DA (2016). Organic photoredox catalysis. Chem. Rev..

[53] Xuan J, Zhang Z-G, Xiao W-J (2015). Visible-light-induced decarboxylative functionalization of carboxylic acids and their derivatives. Angew. Chem. Int. Ed..

[54] Huang H, Jia K, Chen Y (2016). Radical decarboxylative functionalizations enabled by dual photoredox catalysis. ACS Catal..

[55] Jin Y, Fu H (2017). Visible-light photoredox decarboxylative couplings. Asian J. Org. Chem..

[56] Zuo Z, MacMillan DWC (2014). Decarboxylative arylation of α-amino acids via photoredox catalysis: a one-step conversion of biomass to drug pharmacophore. J. Am. Chem. Soc..

[57] Huang H (2017). Visible-light-promoted nickel- and organic-dye-cocatalyzed formylation reaction of aryl halides and triflates and vinyl bromides with diethoxyacetic acid as a formyl equivalent. Angew. Chem. Int. Ed..

[58] Meggers E (2015). Asymmetric catalysis activated by visible light. Chem. Commun..

[59] Brimioulle R, Lenhart D, Maturi MM, Bach T (2015). Enantioselective catalysis of photochemical reactions. Angew. Chem. Int. Ed..

[60] Ding W (2014). Photocatalytic aerobic oxidation/semipinacol rearrangement sequence: a concise route to the core of pseudoindoxyl alkaloids. Tetrahedron Lett..

[61] Bu L (2018). Organocatalytic asymmetric cascade aerobic oxidation and semipinacol rearrangement reaction: a visible light-induced approach to access chiral 2,2-disubstituted indolin-3-ones. Chem. Asian J..

[62] Cheng YZ, Zhao QR, Zhang X, You SL (2019). Asymmetric dearomatization of indole derivatives with N-hydroxycarbamates enabled by photoredox catalysis. Angew. Chem. Int. Ed..

[63] Diaz-Munoz, G., Miranda, I. L., Sartori, S. K., de Rezende, D. C. & Alves Nogueira Diaz, M. Use of chiral auxiliaries in the asymmetric synthesis of biologically active compounds: a review. *Chirality* **31**, 776–812 (2019). 10.1002/chir.23103 31418934

[64] Garner P (2002). Development of an effective chiral auxiliary for hydroxyalkyl radicals. J. Org. Chem..

[65] Sibi MP, Shankar Manyem S, Zimmerman J (2003). Enantioselective radical processes. Chem. Rev..

[66] Evans DA, Bartroli J, Shih TL (1981). Enantioselective aldol condensations. 2. Erythro-selective chiral aldol condensations via boron enolates. J. Am. Chem. Soc..

[67] Heravi MM, Vahideh Zadsirjan V (2014). Recent advances in the application of the Oppolzer camphorsultam as a chiral auxiliary. Tetrahedron.: Asymmetry.

[68] Guo T, Yuan BH, Liu WJ (2017). Highly efficient asymmetric construction of novel indolines and tetrahydroquinoline derivatives via aza-Barbier/C-N coupling reaction. Org. Biomol. Chem..

[69] Curran DP, Kim BH, Daugherty J, Heffner TA (1988). The preparation of optically active Δ2-isoxazolines. A model for asymmetric induction in the non lewis acid catalyzed reactions of Oppolzer’s Chiral Sultam. Tetrahedron Lett..

[70] Curran DP, Shen W, Zhang J, Heffner TA (1990). Asymmetric radical addition, cyclization, and annulation reactions with Oppolzer’s Camphor Sultam. J. Am. Chem. Soc..

