# Supplementary material for: Organophotocatalytic dearomatization of indoles, pyrroles and benzo(thio)furans via a Giese-type transformation
Source: Commun Chem. 2021 Feb 19;4:20. doi: 10.1038/s42004-021-00460-y (PMC9814947; doi:10.1038/s42004-021-00460-y)
Supplement: Supplementary file 2 — Supplementary Information [file 42004_2021_460_MOESM2_ESM.pdf]

Supplementary Materials

Organophotocatalytic dearomatization of indoles, pyrroles and benzo(thio)furans via a Giese-type transformation

Yueteng Zhang[1], Peng Ji[1], Feng Gao[1], Yue Dong[1], He Huang[2], Changqing Wang[1], Ziyuan Zhou[3] and Wei Wang[1]*

[1]*Departments of Pharmacology and Toxicology and Chemistry and Biochemistry, and BIO5 Institute, University of Arizona, Tucson, Arizona 85721, USA*

[2]*Department of Chemistry and Chemical Biology, Cornell University, Ithaca, New York, 14852, USA.*

[3]*National Clinical Research Centre for Infectious Diseases, Shenzhen Third People's Hospital, The Second Hospital Affiliated to Southern University of Science and Technology, Shenzhen, 518112, China.*

Table of Contents

Supplementary Methods 2

1.1 General information 2

1.2 General procedure for the preparation of photocatalyst and substrate **1** 2

1.3 Preparative scale synthesis 12

1.4 Modification of indoline products 13

1.5 Synthesis of racemic product (±) **3** 13

1.6 Asymmetric synthesis of product **7** - **8** 36

1.7 Preparative scale synthesis, recycle of camphorsultam and confirmation of the absolute configuration of products **7** and **8**. 47

1.8 Cyclic voltammetry of **1a** 49

1.9 Mechanism study 49

1.10 Crystal data for *trans*-**3k**. 51

1.11 References 56

Supplementary Methods

1.1 General information

Commercially available reagents were purchased from Sigma Aldrich, Matrix Chemical, AKSci, Alfa Aesar, Acros, AmBeed or TCI, and used as received unless otherwise noted. Merck 60 silica gel was used for chromatography, and Whatman silica gel plates with a fluorescence F254 indicator were used for thin-layer chromatography (TLC) analysis. ^{1}H and ^{13}C NMR spectra were recorded on Bruker Avance 500 MHz or Varian 400 MHz. Chemical shifts in ^{1}H NMR spectra are reported in parts permillion (ppm) relative to residual chloroform (7.26 ppm) or dimethyl sulfoxide (2.50 ppm) as internal standards. 1H NMR data are reported as follows: chemical shift, multiplicity (s = singlet, d = doublet, quint = quintet, sext = sextet, m = multiplet, br = broad), coupling constant in Hertz (Hz) and hydrogen numbers based on integration intensities. ^{13}C NMR chemical shifts are reported in ppm relative to the central peak of $CDCl_3$ (77.16 ppm) or $(CD_3)_2SO$ (39.52 ppm) as internal standards. Both high-resolution and low-resolution mass spectrometry was performed in Analytical and Biological Mass Spectrometry Center. Both blue LED strip and 40 W Kessil Blue LEDs were purchased from Amazon. Cyclic voltammetry was performed at 25 °C on a CH Instrument CHI604xD electrochemical analyzer using a glassy carbon working electrode, a platinum wire counter electrode, and the Ag/AgCl reference electrode calibrated using ferrocene redox couple (4.8 V below vacuum). HPLC analyses with chiral stationary phase were carried out using a SHIMADZU HPLC system with a SPD-20A detector.

1.2 General procedure for the preparation of photocatalyst and substrate 1

Photocatalyst 4CzIPN were synthesized according to reported procedure.[1]

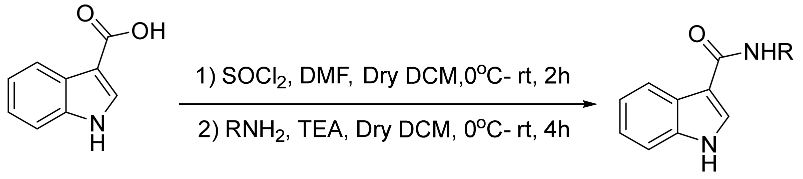

General procedure A

In ice bath, to a solution of 1*H*-indole-3-carboxylic acid (1.0 equiv) in dry DCM (0.2 M) was added thionyl chloride (1.2 equiv) and DMF (0.2 equiv), the resulting mixture was stirred at room temperature for 2h to get acyl chloride solution, which was used in next step. In ice bath, to a solution of amine (1.0 equiv) and TEA (3.0 equiv) in dry DCM (0.2 M) was added newly prepared acyl chloride DCM solution dropwise. The reaction mixture was stirred at room temperature for 4h and was diluted with DCM. The mixture was washed with saturated $NaHCO_3$, followed by 2N HCl and brine. Organic layer was dried by Na_2SO_4, concentrated under vacuum, and purified by flash column chromatography to afford product.

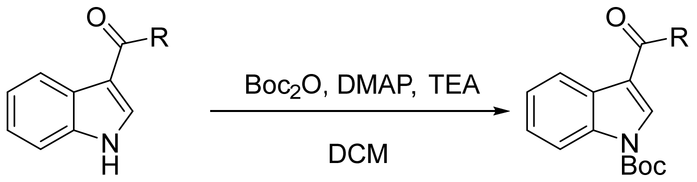

R = Me, OMe, OBn. NHBn, CN, etc

General procedure B to synthesize *N*-Boc indole derivatives 1c, 1g, 1l, 1m.

To a solution of indole derivatives in DCM (0.2 M) was added Boc_2O (1.2 equiv), DMAP (10 mol%) and TEA (0.5 equiv), the reaction mixture was stirred at room temperature for 2h. After the full conversion of substrates (monitored by TLC), the clear solution was concentrated under vacuum and purified by flash column chromatography to afford product (90-99% yield).

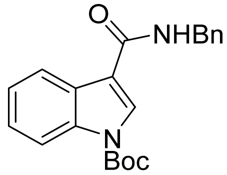

1c was prepared according to general procedure **B**: 91% yield, white solid. ^{1}H NMR (400 MHz, $CDCl_3$) δ 8.13 (d, J = 7.3 Hz, 1H), 8.09–7.99 (m, 2H), 6.31–7.26 (s, s, 6H), 6.56 (s, br, 1H), 4.61 (s, 2H), 1.62 (s, 9H). ^{13}C NMR (100 MHz, $CDCl_3$) δ 164.1, 149.2, 138.5, 135.6, 128.8, 127.9, 127.8, 127.5, 125.2, 123.8, 121.1, 116.0, 115.4, 84.9, 43.6, 28.2.

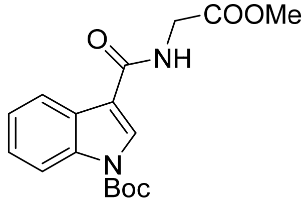

1g was prepared according to general procedure **B**: 82% yield, white solid. ^{1}H NMR (400 MHz, $CDCl_3$) δ 8.13–8.08 (m, 2H), 8.05–8.03 (m, 1H), 7.34–7.26 (m, 2H), 6.83 (t, J = 4.9 Hz, 1H), 4.25 (d, J = 5.3 Hz, 2H), 3.78 (s, 3H), 1.65 (s, 9H). ^{13}C NMR (100 MHz, $CDCl_3$) δ 171.0, 164.2, 149.1, 135.5, 128.3, 127.3, 125.1, 123.8, 120.9, 115.3, 115.3, 84.9, 77.5, 77.2, 76.8, 52.5, 52.5, 41.3, 28.1.

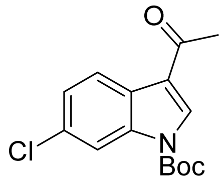

1l

1l was prepared according to general procedure **B**: 93% yield, white solid. ^{1}H NMR (400 MHz, $CDCl_3$) δ 8.27 (d, J = 8.5 Hz, 1H), 8.18–8.13 (m, 2H), 7.31 (dd, J = 8.5, 1.9 Hz, 1H), 2.55 (s, 3H), 1.71 (s, 9H). ^{13}C NMR (100 MHz, $CDCl_3$) δ 193.7, 148.8, 136.0, 132.6, 131.7, 126.0, 125.1, 123.7, 120.5, 115.4, 86.1, 28.2, 27.7.

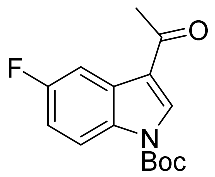

1m was prepared according to general procedure **B**: 99% yield. White solid. ^{1}H NMR (400 MHz, $CDCl_3$) δ 8.23 (s, 1H), 8.05 (dd, *J* = 9.2 Hz, 2.8 Hz, 2H), 7.10 (td, *J* = 8.8 Hz, 2.4 Hz, 1H), 2.55 (s, 3H), 1.71 (s, 9H). ^{13}C NMR (125 MHz, $CDCl_3$) δ 193.9, 160.5 (d, *J* = 240 Hz), 149.1, 133.6, 132.13, 128.63 (d, *J* = 10 Hz), 120.5 (d, *J* = 3.7 Hz), 116.2 (d, *J* = 6.2 Hz), 113.7 (d, *J* = 25 Hz), 108.7 (d, *J* = 25 Hz), 85.9, 28.2, 27.6.

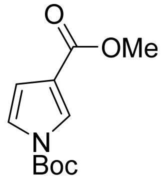

1s

1s was prepared according to general procedure **B**: 98% yield. White solid. ^{1}H NMR (400 MHz, $CDCl_3$) δ 7.76 (dd, *J* = 2.1, 1.7 Hz, 1H), 7.14 (dd, *J* = 3.3, 2.2 Hz, 1H), 6.53 (dd, *J* = 3.3, 1.6 Hz, 1H), 3.76 (s, 3H), 1.55 (s, 9H). ^{13}C NMR (100 MHz, $CDCl_3$) δ 164.6, 148.2, 124.9, 120.8 119.3, 112.0, 85.1, 51.5, 28.0.

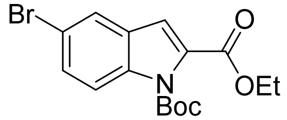

1s was prepared according to general procedure **B**: 99% yield. White solid. ^{1}H NMR (500 MHz, $CDCl_3$) δ 7.9 (d, *J* = 9.0 Hz, 1H), 7.14 (d, *J* = 2.0 Hz, 1H), 7.48 (dd, *J* = 9.0 Hz, 2.0 Hz, 1H), 7.00 (d, *J* = 0.5 Hz, 1H), 4.38 (q, *J* = 7.0 Hz, 2H), 1.62 (s, 9H), 1.39 (t, *J* = 7.0 Hz, 3H). ^{13}C NMR (126 MHz, $CDCl_3$) δ 161.8, 149.1, 136.5, 132.0, 129.7, 129.4, 124.7, 116.6, 116.6, 113.4, 85.2, 61.7, 27.8, 14.2.

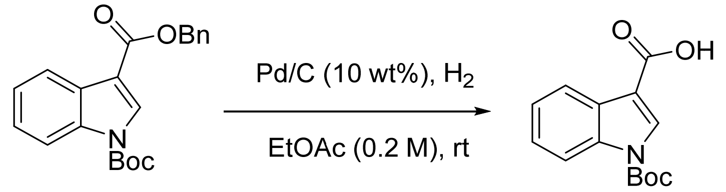

1-(*tert*-Butoxycarbonyl)-1*H*-indole-3-carboxylic acid was prepared by the following procedure: to a solution of 3-benzyl 1-(*tert*-butyl) 1*H*-indole-1,3-dicarboxylate (prepared according to general procedure **B**) in EtOAc (0.2M) was added 10 wt% Pd/C (10% wet). The reaction mixture was stirred under H_2 (in balloon) overnight. After completion of reaction, the mixture was filtrated through celite and concentrate under vacuum to obtain product as white solid, 99% yield.

General procedure C for the synthesis of 1i and 1j:

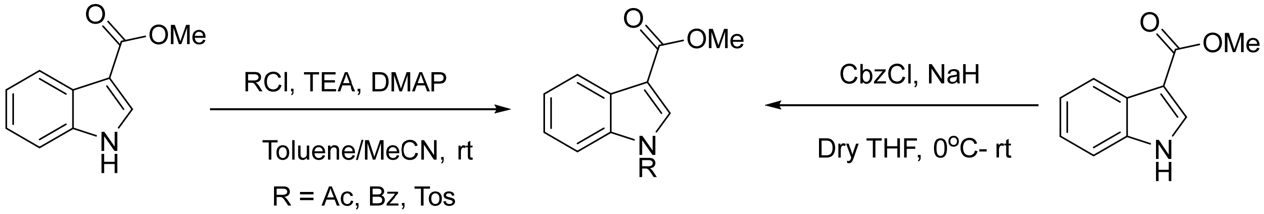

To a solution of indole derivatives in the solvent (toluene/MeCN = 1/1, 0.2 M) was added DMAP (20 mol%), TEA (1.2 equiv) and acyl chloride (1.2 equiv) The reaction mixture was stirred at room temperature till the full conversion of substrates (monitored by TLC). The reaction solution was concentrated under vacuum and purified by flash column chromatography to afford product.

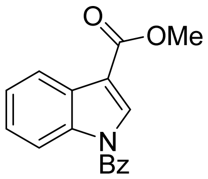

1i was synthesized according to general procedure **C**: 94% yield, white solid. ^{1}H NMR (400 MHz, $CDCl_3$) δ 8.38 (dd, J = 6.1, 3.1 Hz, 1H), 8.20 (dd, J = 5.1, 2.3 Hz, 1H), 7.99 (d, J = 3.4 Hz, 1H), 7.76 (dd, J = 7.1, 1.1 Hz, 2H), 7.69–7.62 (m, 1H), 7.61–7.53 (m, 2H), 7.48–7.39 (m, 2H), 3.91 (d, J = 3.2 Hz, 3H). ^{13}C NMR (100 MHz, $CDCl_3$) δ 168.7, 164.5, 136.5, 133.5, 133.5, 132.8, 129.6, 129.0, 127.8, 125.8, 125.1, 121.8, 116.3, 113.4, 51.7.

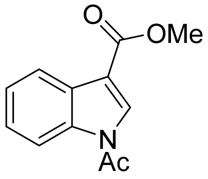

1j was synthesized according to general procedure **C**: toluene was used as solvent. 87% yield, white solid. ^{1}H NMR (400 MHz, $CDCl_3$) δ 8.30 (d, J = 7.3 Hz, 1H), 8.06–7.99 (m, 1H), 7.91 (s, 1H), 7.34–7.26 (m, 2H), 3.87 (d, J = 0.9 Hz, 3H), 2.55 (d, J = 1.1 Hz, 3H). ^{13}C NMR (100 MHz, $CDCl_3$) δ 168.6, 164.1, 135.7, 131.1, 131.1, 127.1, 125.7, 124.6, 121.3, 116.3, 113.4, 51.5, 23.7.

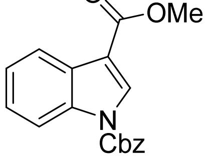

1k

1k was synthesized according to the following procedure: In ice bath, to a solution of indole derivatives in freshly distilled anhydrous THF (0.2 M) was added NaH (1.2 equiv, 60% in mineral oil). After stirring of mixture in ice bath for 30 min, CbzCl (1.2 equiv) was added and the reaction mixture was stirred at room temperature for 3h. After the full conversion of substrates, the reaction solution quenched by 0.5 ml MeOH, concentrated under vacuum and purified by flash column chromatography to afford product. 91% yield, white solid. ^{1}H NMR (400 MHz, $CDCl_3$) δ 8.30 (d, J = 4.3 Hz, 1H), 8.24–8.14 (m, 2H), 7.53–7.47 (m, 2H), 7.46–7.41 (m, 3H), 7.39–7.34 (m, 2H),

5.48 (s, 2H), 3.93 (s, 3H). ^{13}C NMR (100 MHz, $CDCl_3$) δ 164.5, 150.4, 135.7, 134.6, 131.7, 129.16, 129.00, 128.85, 127.6, 125.6, 124.4, 121.9, 115.3, 113.3, 69.6, 51.7.

General procedure D for the synthesis of 1as, 1at, 1au, 1av, 1aw, lax, 5a, 5b, 5c:

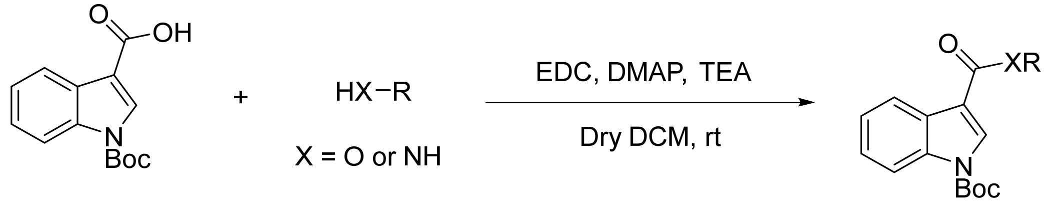

To a solution of 1-(*tert*-butoxycarbonyl)-1*H*-indole-3-carboxylic acid (1.0 equiv) in freshly distilled anhydrous DCM (0.2 M) was added TEA (2.0 equiv), DMAP (20 mol%), EDC (1.2 equiv) and alcohol or amine (1.0 equiv). The reaction mixture was stirred at room temperature for 16h. The reaction was diluted with DCM and washed by 1N HCl, saturated $NaHCO_3$ and brine. The organic layer was concentrated under vacuum and purified by flash column chromatography to afford product.

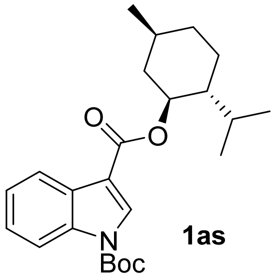

1as

1as was prepared according to procedure **D**: 78% yield, colorless oil. ^{1}H NMR (400 MHz, $CDCl_3$) δ 8.29 (s, 1H), 8.23–8.15 (m, 2H), 7.40–7.32 (m, 2H), 5.01 (td, *J* = 10.8, 4.3 Hz, 1H), 2.18 (d, *J* = 11.9 Hz, 1H), 2.08–1.97 (m, 1H), 1.75–1.69 (m, 11H), 1.62–1.54 (m, 2H), 1.22–1.13 (m, 2H), 0.95 (dd, *J* = 6.6, 4.3 Hz, 7H), 0.84 (d, *J* = 7.0 Hz, 3H). ^{13}C NMR (101 MHz, $CDCl_3$) δ 163.9, 149.1, 135.6, 131.9, 127.7, 125.0, 123.9, 121.8, 115.2, 113.0, 85.0, 77.5, 77.2, 76.8, 74.1, 47.4, 41.3, 34.4, 31.5, 28.1, 26.6, 23.7, 22.1, 20.9, 16.6.

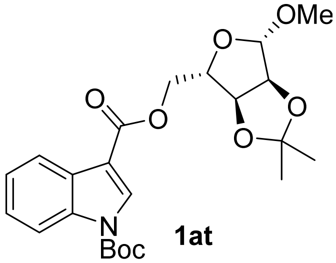

1at

1at was prepared according to procedure **D**: 88% yield, white solid. ^{1}H NMR (400 MHz, $CDCl_3$) δ 8.29 (s, 1H), 8.15 (t, *J* = 6.2 Hz, 2H), 7.39–7.29 (m, 2H), 5.01 (s, 1H), 4.76 (d, *J* = 5.8 Hz, 1H), 4.64 (d, *J* = 5.9 Hz, 1H), 4.55 (t, *J* = 6.7 Hz, 1H), 4.36 (dd, *J* = 6.5, 3.4 Hz, 2H), 3.33 (s, 3H), 1.67 (s, 9H), 1.49 (s, 3H), 1.31 (s, 3H).

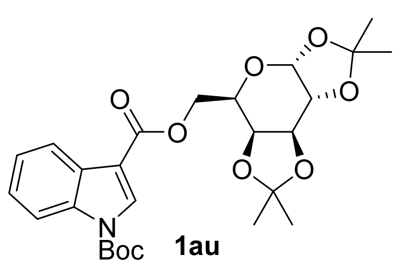

1au

1au was prepared according to procedure **D**: 76% yield, white solid. ^{1}H NMR (400 MHz, $CDCl_3$) δ 8.29 (s, 1H), 8.18 (t, *J* = 9.0 Hz, 2H), 7.39–7.28 (m, 2H), 5.58 (d, *J* = 4.9 Hz, 1H), 4.65 (dd, *J* = 7.8, 2.1 Hz, 1H), 4.54 (dd, *J* = 11.6, 4.3 Hz, 1H), 4.46 (dd, *J* = 11.5, 7.8 Hz, 1H), 4.34 (dd, *J* = 10.1, 2.9 Hz, 2H), 4.24 (dd, *J* = 6.3, 3.9 Hz, 1H), 1.67 (s, 9H), 1.53 (s, 3H), 1.48 (s, 3H), 1.34 (d, *J* = 9.7 Hz, 6H). ^{13}C NMR (100 MHz, $CDCl_3$) δ 164.1, 149.0, 135.6, 132.4, 127.6, 125.1, 124.0, 122.0, 115.2, 112.3, 109.8, 108.9, 96.4, 85.1, 71.3, 70.8, 70.6, 66.4, 63.6, 28.1, 26.1, 26.1, 25.1, 24.5.

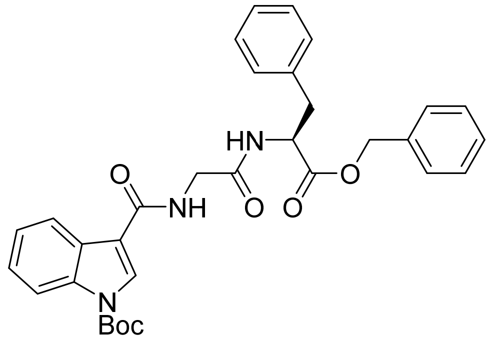

1av was prepared according to procedure **D**: 83% yield, white solid. ^{1}H NMR (400 MHz, $CDCl_3$) δ 8.15 (d, *J* = 6.6 Hz, 2H), 8.08 (d, *J* = 7.7 Hz, 1H), 7.37–7.18 (m, 8H), 7.12 (d, *J* = 2.7 Hz, 4H), 7.04 (d, *J* = 2.7 Hz, 2H), 5.10 (dd, *J* = 27.2, 12.2 Hz, 2H), 4.93 (d, *J* = 7.3 Hz, 1H), 4.19–4.04 (m, 2H), 3.11 (qd, *J* = 13.9, 6.2 Hz, 2H), 1.65 (s, 9H). ^{13}C NMR (100 MHz, $CDCl_3$) δ 171.21, 169.3, 164.5, 149.1, 135.7, 135.6, 135.12, 129.35, 128.6, 128.5, 128.5, 127.4, 127.1, 125.2, 123.9, 121.2, 115.3, 115.2, 85.0, 67.4, 53.6, 43.3, 37.9, 28.2.

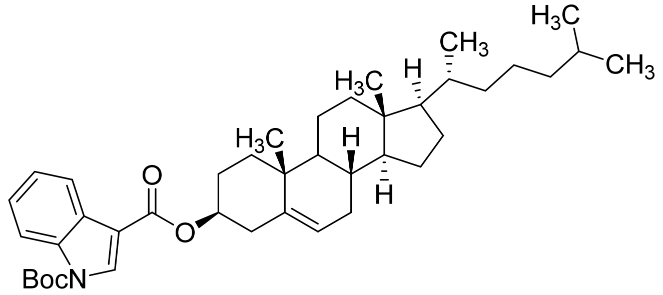

1aw was prepared according to procedure **D**: 83% yield, white solid. ^{1}H NMR (400 MHz, $CDCl_3$) δ 8.27 (s, 1H), 8.21–8.14 (m, 2H), 7.38–7.32 (m, 2H), 5.44 (d, *J* = 3.2 Hz, 1H), 4.96–4.86 (m, 1H), 2.52 (d, *J* = 7.3 Hz, 2H), 2.10–1.75 (m, 7H), 1.69 (s, 9H), 1.63–1.43 (m, 7H), 1.40–1.26 (m, 5H), 1.20–1.06 (m, 9H), 1.04–0.97 (m, 3H), 0.93 (d, *J* = 6.4 Hz, 3H), 0.88 (dd, *J* = 6.6, 1.3 Hz, 6H), 0.69 (s, 3H). ^{13}C NMR (100 MHz, $CDCl_3$) δ 163.8, 149.2, 139.8, 135.7, 132.1, 127.7, 125.1, 124.0, 122.9, 121.9, 115.3, 113.0, 85.1, 77.5, 77.2, 76.8, 74.2, 56.8, 56.3, 50.2, 42.4, 39.9, 39.6, 38.5,

37.2, 36.8, 36.3, 35.9, 35.0, 32.1, 32.0, 28.4, 28.2, 28.2, 28.1, 25.6, 24.8, 24.4, 24.0, 23.0, 22.7, 21.2, 19.5, 18.9, 12.0.

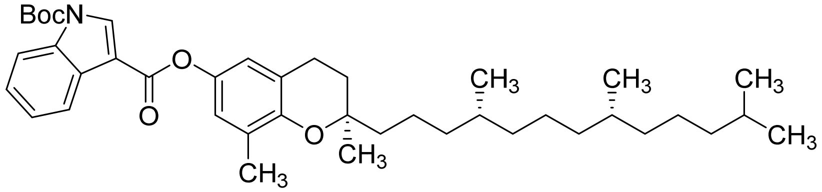

1ax was prepared according to procedure **D**: 95% yield, colorless oil. ^{1}H NMR (400 MHz, $CDCl_3$) δ 8.44 (s, 1H), 8.28–8.20 (m, 2H), 7.45–7.35 (m, 2H), 6.87 (s, 1H), 6.82 (s, 1H), 2.78 (d, *J* = 5.9 Hz, 2H), 2.21 (s, 3H), 1.89–1.72 (m, 12H), 1.62–1.55 (m, 4H), 1.48–1.36 (m, 5H), 1.36–1.31 (m, 13H), 1.19–1.11 (m, 7H), 0.89 (d, *J* = 6.0 Hz, 6H). ^{13}C NMR (100 MHz, $CDCl_3$) δ 163.3, 150.0, 149.0, 142.4, 135.7, 132.8, 127.8, 127.5, 125.4, 124.2, 121.9, 121.5, 121.2, 119.5, 115.3, 112.0, 85.3, 77.5, 77.2, 76.8, 76.3, 40.2, 39.5, 37.6, 37.6, 32.9. 32.8, 31.2, 28.2, 28.1, 24.9, 24.6, 24.4, 22.9, 22.8, 22.6, 21.1, 19.9, 19.8, 16.3.

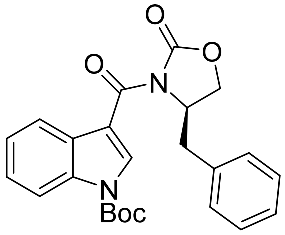

5a was prepared according to procedure **D**: 78% yield. ^{1}H NMR (400 MHz, $CDCl_3$) δ 8.35 (s, 1H), 8.19 (d, *J* = 7.9 Hz, 1H), 8.07 (d, *J* = 7.1 Hz, 1H), 7.42–7.20 (m, 6H), 5.00–4.87 (m, 1H), 4.29 (dd, *J* = 14.4, 8.3 Hz, 1H), 4.23–4.17 (m, 1H), 3.41 (d, *J* = 13.4 Hz, 1H), 2.92 (dd, *J* = 12.4, 10.0 Hz, 1H), 1.68 (s, 9H).

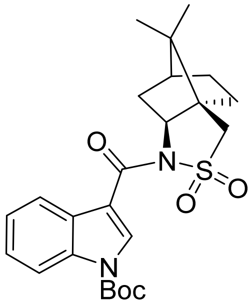

5b was prepared according to procedure **D**: White foam solid, 83% yield. ^{1}H NMR (400 MHz, $CDCl_3$) δ 8.56 (s, 1H), 8.12 (d, *J* = 7.9 Hz, 1H), 8.08 (d, *J* = 7.8 Hz, 1H), 7.40 – 7.28 (m, 2H), 4.28 (dd, *J* = 7.1, 4.8 Hz, 1H), 3.56 (d, *J* = 13.7 Hz, 1H), 3.46 (d, *J* = 13.7 Hz, 1H), 2.16 – 2.01 (m, 2H), 2.01 – 1.83 (m, 3H), 1.68 (s, 9H), 1.49 (dd, *J* = 19.3, 9.8 Hz, 1H), 1.38 (dd, *J* = 14.3, 6.9 Hz, 1H), 1.33 (s, 3H), 1.01 (s, 3H). ^{13}C NMR (101 MHz, $CDCl_3$) δ 163.7, 149.1, 135.2, 133.5, 128.3, 125.5, 124.1, 121.6, 115.2, 114.1, 85.2, 66.0, 53.7, 48.1, 47.9, 45.2, 38.5, 33.2, 28.1, 26.6, 21.3, 20.0. HRMS (ESI) calcd for $[C_{24}H_{30}N_2O_5S + Na]^+$: 481.1768, found: 481.1748.

Synthesis of *N*-Cbz-indole-3-carboxylic acid

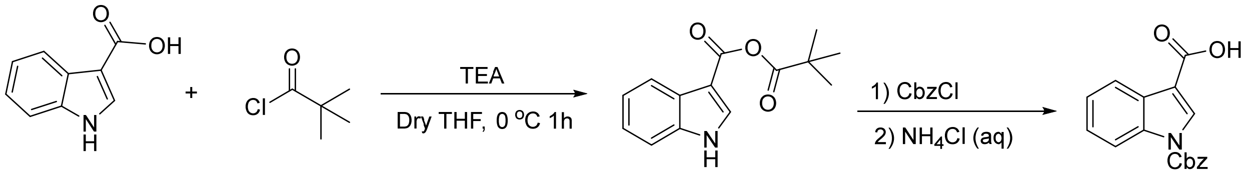

At 0 °C, to a solution of 1*H*-indole-3-carboxylic acid (1.0 equiv) in anhydrous THF was added TEA (2.0 equiv) and pivaloyl chloride (1.1 equiv). The reaction mixture was stirred for 30 min. After the filtration through a short celite, the filtrate was concentrated under vacuum and dissolved in anhydrous DCM (0.2 M). At 0 °C, to the solution of DCM was added ground NaOH (5.0 equiv), tetrabutylammonium hydrogensulfate (0.5 equiv) and CbzCl (1.5 equiv) in sequence. The mixture was stirred for 5h, after which saturated NH_4Cl (10 ml/ 1.0 mol subsatrate) was added to mixture followed by stirring for anther 1h. The DCM layer was separated, and aqueous layer was extracted by DCM. The combined organic layer was dried by Na_2SO_4, concentrated under vacuum and purified by flash column chromatography to afford product as white solid.

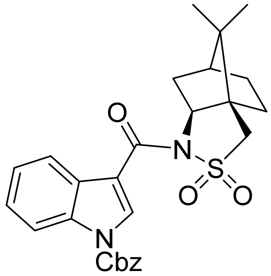

5c was prepared according to procedure **D**: White foam solid, 77% yield. ^{1}H NMR (400 MHz, $CDCl_3$) δ 8.58 (s, 1H), 8.17 (d, *J* = 7.8 Hz, 1H), 8.07 (d, *J* = 7.0 Hz, 1H), 7.48 (d, *J* = 6.8 Hz, 1H), 7.42 – 7.32 (m, 5H), 5.54 – 5.44 (m, 2H), 4.27 (dd, *J* = 7.1, 4.8 Hz, 1H), 3.56 (d, *J* = 13.7 Hz, 1H), 3.46 (d, *J* = 13.7 Hz, 1H), 2.16 – 2.01 (m, 2H), 2.01 – 1.83 (m, 3H), 1.49 (dd, *J* = 19.3, 9.8 Hz, 1H), 1.38 (dd, *J* = 14.3, 6.9 Hz, 1H), 1.31 (s, 3H), 0.99 (s, 3H). ^{13}C NMR (101 MHz, $CDCl_3$) δ 163.5, 150.3, 135.2, 134.7, 132.7, 128.8, 128.2, 128.1, 125.7, 124.3, 121.6, 115.1, 114.9, 69.3, 65.8, 53.6, 48.0, 47.8, 45.1, 38.4, 33.1, 26.6, 21.3, 19.9.

General procedure E for the synthesis of carboxylic acid 2e, 2f, 2k, 2m, 2n

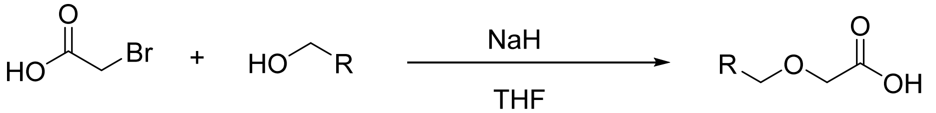

At 0 °C, alcohol (2.0 mmol, 2.0 equiv) in 2.0 mL dry THF was added slowly into the mixture of NaH (100mg, 60% in mineral oil, 2.5 mmol, 2.5 equiv) in 2.0 mL THF under N_2. After 10 min, a solution of bromoacetic acid in 2.0 mL dry THF was added slowly to reaction mixture by syringe. The reaction mixture was warmed to rt, stirred for 16h and finally quenched by 5.0 ml water. The mixture was diluted with 10 ml EtOAc and the solution was portioned in separatory funnel. Aqueous solution was collected, and organic layer was washed by 5.0 mL 1M NaOH. The water layers were combined and acidified with 2M HCl to pH = 1. The acidified aqueous phase extracted by EtOAc (3 x 10 ml), the combined layers were dried over anhydrous Na_2SO_4 and concentrated under vacuum to obtain product.

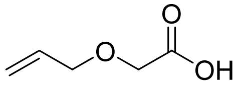

2-(Allyloxy)acetic acid **2e** was synthesized according to general procedure **E**. 65% yield, colorless oil. ^{1}H NMR (400 MHz, $CDCl_3$) δ 943 (s, br, 1H), 5.90 (dq, *J* = 11.0, 5.8 Hz, 1H), 5.29 (dd, *J* = 22.1, 13.8 Hz, 2H), 4.14 (s, 2H), 4.11 (d, *J* = 5.7 Hz, 2H). ^{13}C NMR (101 MHz, $CDCl_3$) δ 175.4, 133.1, 118.8, 72.5, 66.4.

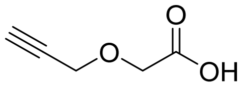

2-(Prop-2-yn-1-yloxy)acetic acid **2f** was synthesized according to general procedure **E**. 77% yield, colorless oil. ^{1}H NMR (400 MHz, $CDCl_3$) δ 9.22 (s, br, 1H), 4.33 – 4.28 (m, 2H), 4.24 (s, 2H), 2.50 (t, *J* = 2.1 Hz, 1H).

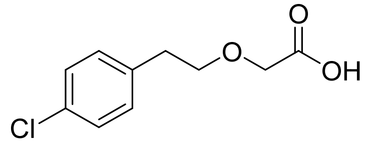

2-(4-Chlorophenethoxy)acetic acid **2k** was synthesized according to general procedure **E**: 71% yield, white solid. ^{1}H NMR (400 MHz, $CDCl_3$) δ 9.52 (s, br, 1H), 7.24 (d, *J* = 8.3 Hz, 2H), 7.14 (d, *J* = 8.2 Hz, 2H), 4.10 (s, 2H), 3.74 (t, *J* = 6.9 Hz, 2H), 2.89 (t, *J* = 6.9 Hz, 2H). ^{13}C NMR (101 MHz, $CDCl_3$) δ 175.5, 136.8, 132.3, 130.3, 128.6, 72.4, 67.9, 35.4.

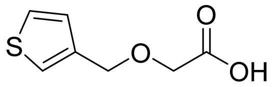

2-(Thiophen-3-ylmethoxy)acetic acid **2m** was synthesized according to general procedure **E**: 81% colorless oil. ^{1}H NMR (400 MHz, $CDCl_3$) δ 10.62 (s, br, 1H), 7.31 (d, *J* = 3.5 Hz, 1H), 7.26 (s, 1H), 7.10 (d, *J* = 4.7 Hz, 1H), 4.65 (s, 2H), 4.13 (s, 2H). ^{13}C NMR (101 MHz, $CDCl_3$) δ 175.8, 137.8, 127.5, 126.6, 124.1 68.5, 66.4.

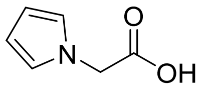

2-(1*H*-pyrrol-1-yl)acetic acid **2n** was synthesized according to general procedure **E**: 76%, colorless oil. ^{1}H NMR (400 MHz, $CDCl_3$) δ 9.31 (s, 1H), 6.67 (d, *J* = 1.9 Hz, 2H), 6.23 (d, *J* = 1.9 Hz, 2H), 4.70 (s, 2H).

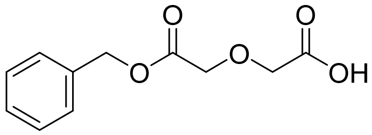

2-(2-(Benzyloxy)-2-oxoethoxy)acetic acid **2l**. At 0 ºC, to a solution of diglycolic acid (670 mg, 5.0 mmol) in acetone (0.2 M) was added TEA (905 μl, 6.5 mmol) and BnBr (415 μl, 3.5 mmol). The reaction mixture was warmed to rt and stirred for 16h. The mixture was filtered to remove salt. The filtrate was concentrated under vacuum and residue was dissolved into 20 ml saturated $NaHCO_3$. After washing EtOAc (3 x 10 ml), the aqueous solution was acidified to pH = 2. The acidified aqueous phase extracted by EtOAc (3 x 10 ml), the combined layers were dried over

anhydrous Na_2SO_4 and concentrated under vacuum to obtain product **2l** (556 mg, 71%) as light yellow oil. ^{1}H NMR (400 MHz, $CDCl_3$) δ 9.75 (s, br, 1H), 7.36 (s, 5H), 5.21 (s, 2H), 4.28 (s, 4H). ^{13}C NMR (101 MHz, $CDCl_3$) δ 174.1, 170.1, 135.0, 128.8, 128.8, 128.6, 68.6, 68.4, 67.2.

General procedure F for the synthesis of carboxylic acid 2j, 2x, 2y.

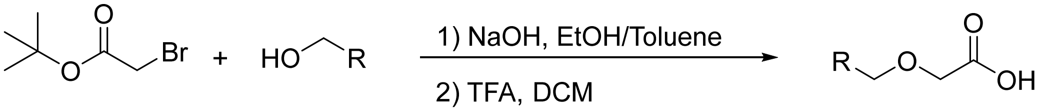

To a 5 mL solution of 5M NaOH was added tetrabutylammonium hydrogensulfate (101 mg, 30 mol%), 3.0 ml toluene, alcohol (1.0 mmol, 1.0 equiv) and *tert*-butyl bromoacetate (293 mg, 1.5 mmol, 1.5 equiv). The reaction mixture was stirred at rt form 16 hours, after which the mixture was extracted with EtOAc (3 x 10 ml). The combined organic layers were combined, concentrated under vacuum and purified by flash column chromatography to afford product. The *tert*-butyl esters dissolved in DCM (0.2 M) was treated with TFA (10 equiv). After stirring of 2 hours at rt, the solvent was removed under vacuum to get final product.

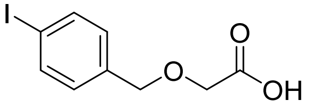

2-((4-Iodobenzyl)oxy)acetic acid **2j** was synthesized according to general procedure **F**: 64% yield for two steps, white solid. tert-Butyl 2-((4-iodobenzyl)oxy)acetate: ^{1}H NMR (400 MHz, $CDCl_3$) δ 7.67 (d, J = 8.2 Hz, 2H), 7.12 (d, J = 8.0 Hz, 2H), 4.55 (s, 2H), 3.97 (s, 2H), 1.47 (s, 9H). ^{13}C NMR (101 MHz, $CDCl_3$) δ 169.4, 137.6, 137.2, 129.9, 93.5, 81.8, 77.5, 77.2, 76.8, 72.6, 67.9, 28.2. **2k**: ^{1}H NMR (400 MHz, $CDCl_3$) δ 10.0 (s, br, 1H), 7.67 (d, J = 8.1 Hz, 2H), 7.09 (d, J = 8.0 Hz, 2H), 4.56 (s, 2H), 4.13 (s, 2H). ^{13}C NMR (101 MHz, $CDCl_3$) δ 175.4, 137.7, 136.3, 129.9, 93.9, 72.7, 66.7.

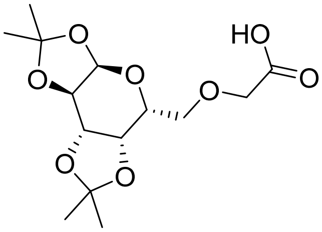

2-(((3a*R*,5*R*,5a*S*,8a*S*,8b*R*)-2,2,7,7-tetramethyltetrahydro-5*H*-bis([1,3]dioxolo)[4,5-*b*:4',5'-*d*]pyran-5-yl)methoxy)acetic acid **2x** was synthesized according to general procedure **F**: 69% yield for two steps, white solid. ^{1}H NMR (400 MHz, $CDCl_3$) δ 5.92 (s, br, 1H), 5.55 (d, J = 5.0 Hz, 1H), 4.66 (dd, J = 7.8, 2.3 Hz, 1H), 4.36 (dd, J = 4.9, 2.5 Hz, 1H), 4.30 (dd, J = 7.9, 1.8 Hz, 1H), 4.21 (d, J = 17.1 Hz, 1H), 4.12 (d, J = 4.7 Hz, 1H), 4.06 (dd, J = 12.0, 6.5 Hz, 1H), 3.79 – 3.67 (m, 2H), 1.54 (s, 3H), 1.47 (s, 3H), 1.36 (s, 3H), 1.34 (s, 3H). ^{13}C NMR (101 MHz, $CDCl_3$) δ 172.2, 110.0, 109.1, 96.3, 71.2, 71.0, 70.7, 70.5, 68.9, 66.4, 26.1, 26.0, 25.0, 24.6.

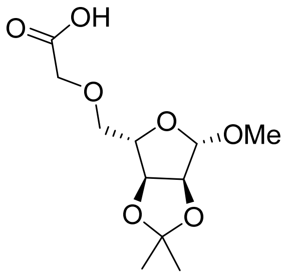

2-(((3a*S*,4*S*,6*S*,6a*S*)-6-methoxy-2,2-dimethyltetrahydrofuro[3,4-*d*][1,3]dioxol-4-yl)methoxy) acetic acid **2y** was synthesized according to general procedure **F**: 72% yield, white solid. ^{1}H NMR (400 MHz, $CDCl_3$) δ 10.07 (s, br, 1H), 4.99 (s, 1H), 4.74 (d, J = 5.9 Hz, 1H), 4.59 (d, J = 5.9 Hz, 1H), 4.40 (t, J = 4.8 Hz, 1H), 4.21 (d, J = 16.5 Hz, 1H), 4.04 (d, J = 16.5 Hz, 1H), 3.64 (ddd, J = 37.6, 9.7, 5.0 Hz, 2H), 3.37 (s, 3H), 1.48 (s, 3H), 1.31 (s, 3H). ^{13}C NMR (101 MHz, cdcl$_3$) δ 172.8, 112.7, 110.8, 85.6, 85.2, 82.0, 72.9, 68.4, 55.7, 26.6, 25.0.

1.3 Preparative scale synthesis

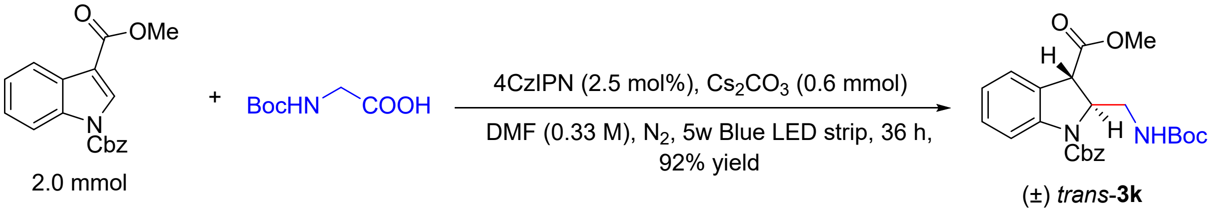

To an oven-dried 10 mL-Schlenk tube equipped with a stir bar, was added 1-benzyl 3-methyl 1*H*-indole-1,3-dicarboxylate **1k** (2.0 mmol, 1.0 equiv), 4CzIPN (40.0 mg, 2.5 mol%), Boc-glycine (2.6 mmol, 1.3 equiv), Cs_2CO_3 (195 mg, 0.6 mmol, 30 mol%) and DMF (6.0 mL). The reaction mixture was degassed by freeze-pump-thaw method, then sealed with parafilm. The solution was then stirred at room temperature under the irradiation of a 5w blue LED strip for 36h. After completion of the reaction, the mixture was diluted with water and extracted by EtOAc. The organic layer was collected, dried by Na_2SO_4 and concentrated under vacuum. The residue was purified by flash column chromatography (25% acetone in hexane) to afford product (±) *trans*-**3k** (810 mg, 92% yield) as white solid.

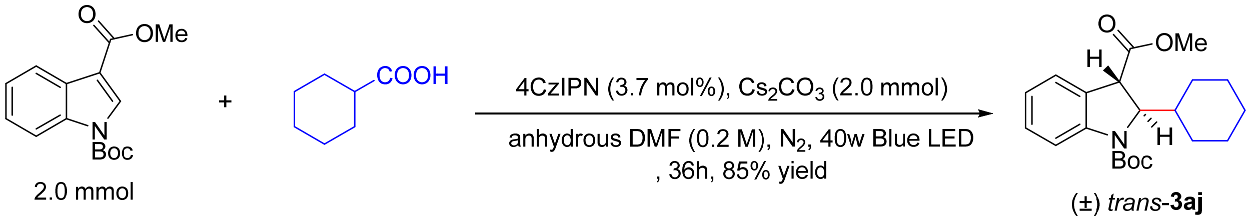

To an oven-dried 10 mL-Schlenk tube equipped with a stir bar, was added 1-(*tert*-Butyl) 3-methyl 1*H*-indole-1,3-dicarboxylate **1a** (550 mg, 2.0 mmol, 1.0 equiv), 4CzIPN (60 mg, 0.075 mmol, 3.7 mol%), cyclohexanecarboxylic acid (512 mg, 4.0 mmol, 2.0 equiv), Cs_2CO_3 (650 mg, 2.0 mmol, 1.0 equiv) and DMF (10 mL). The reaction mixture was degassed by freeze-pump-thaw method, then sealed with parafilm. The reaction was stirred at room temperature under the irradiation of two 40w Blue LED lamps for 36 h. After completion of the reaction, the mixture was diluted with water and extracted by EtOAc. The organic layer was collected, dried by Na_2SO_4

and concentrated under vacuum. The residue was purified by flash column chromatography (0-10% EtOAc in hexane) to afford product (±) *trans*-**3aj** (610 mg, 85% yield) as colorless oil.

1.4 Modification of indoline products

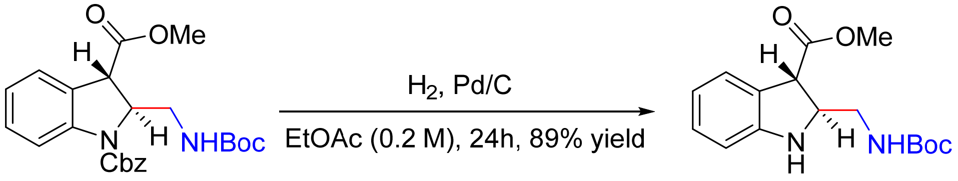

To 10 mL-Schlenk tube equipped with a stir bar was added (±) *trans*-**3k** (88mg, 0.2 mmol), EtOAc (2.0 ml) and 10% wet Pd/C (10 mg). The reaction mixture was filled with nitrogen and then exchanged with H_2 in balloon. After stirring of 24h under the H_2 at room temperature, the reaction mixture was filtrated through a short celite. The filtrate was concentrated under vacuum and was purified by flash column chromatography (30% EtOAc in hexane) to afford product (±) *trans*-**4k** (54.5 mg, 89% yield) as colorless oil. ^1H NMR (400 MHz, $CDCl_3$) δ 7.26 (d, 6.4 Hz, 1H), 7.08 (t, J = 7.6 Hz, 1H), 6.74 (t, J = 7.4 Hz, 1H), 6.63 (d, J = 7.8 Hz, 1H), 5.02 (s, 1H), 4.37 (d, J = 4.2 Hz, 2H), 3.93 (d, J = 7.3 Hz, 1H), 3.78 (s, 3H), 3.35 (s, 2H), 1.43 (s, 9H). ^{13}C NMR (100 MHz, $CDCl_3$) δ 172.2, 156.3, 150.1, 128.9, 125.3, 119.2, 110.2, 79.6, 77.5, 77.2, 76.8, 44.5, 28.4. HRMS (ESI) calcd for $[C_{16}H_{22}N_2O_4 + Na]^+$: 329.1472, found: 329.1470.

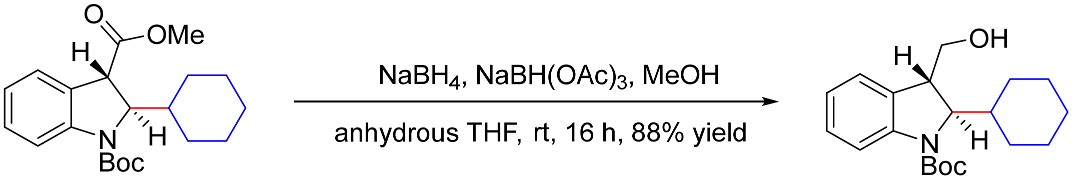

To 50 mL round bottom flask equipped with a stir bar was added (±) *trans*-**3aj** (168 mg, 0.47 mmol, 1.0 equiv), anhydrous THF (5.0 ml), $NaBH_4$ (71.0 mg, 1.87 mmol, 4.0 equiv), $NaBH(OAc)_3$ (21.0 mg, 0.1 mmol, 20 mol%) and MeOH (150 μl). The reaction mixture was stirred at room temperature for 16h. After the reaction was completed, the mixture was filtrated through a short celite. The filtrate was concentrated under vacuum and was purified by flash column chromatography (20% EtOAc in hexane) to afford product (±) *trans*-**4aj** (137 mg, 88% yield) as colorless oil. ^1H NMR (400 MHz, $CDCl_3$) δ 7.78 (s, br, 1H), 7.20–7.15 (m, 2H), 6.93 (t, J = 7.4 Hz, 1H), 4.11 (s, 1H), 3.59 (s, 2H), 3.13 (t, J = 6.3 Hz, 1H), 2.13 (s, br, 1H), 1.86 (s, 1H), 1.75 (d, J = 11.4 Hz, 1H), 1.59 (d, J = 25.9 Hz, 12H), 1.46 (d, J = 12.7 Hz, 1H), 1.27–1.05 (m, 4H), 0.85–0.76 (m, 1H). ^{13}C NMR (100 MHz, $CDCl_3$) δ 152.9, 143.0, 124.5, 122.5, 115.5, 80.8, 67.3, 66.2, 45.1, 41.8, 28.6, 28.5, 26.9, 26.6, 26.4, 26.2.

1.5 Synthesis of racemic product (±) 3

General procedure G:

To an oven-dried 10 mL-Schlenk tube equipped with a stir bar, was added indole derivatives (0.2 mmol, 1.0 equiv), photocatalyst 4CzIPN (8.0 mg, 5 mol%), acid (0.26 mmol, 1.3 equiv), Cs_2CO_3 (65.0 mg, 0.2 mmol, 1.0 equiv) and DMF (2.0 mL). The mixture was degassed by freeze-pump-thaw method, then sealed with parafilm. The solution was then stirred at room temperature

under the irradiation of a 5w blue LED strip or two 40 w blue LED lamps for 36h. After completion of the reaction, the mixture was diluted with 20 ml water and extracted by EtOAc (3 x 10 ml). The organic layer was collected, dried by Na_2SO_4 and concentrated under vacuum. The residue was purified by flash column chromatography to afford product.

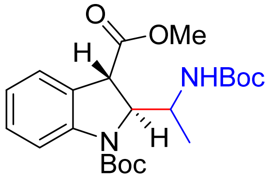

(±) *trans*-**3a**

(±) *trans*-**3a** (mixture of **isomer-a** and **isomer-b**) was synthesized according to the general procedure **G**. 1-(*tert*-Butyl) 3-methyl 1*H*-indole-1,3-dicarboxylate[2] (55.0 mg, 0.2 mmol, 1.0 equiv), 4CzIPN (8.0 mg, 0.01 mmol, 5 mol%), Boc-L-alanine (49.0 mg, 0.26 mmol, 1.3 equiv), Cs_2CO_3 (65.0 mg, 0.2 mmol, 1.0 equiv) and DMF (2.0 mL) were used. The reaction mixture was irritated by 5w Blue LED strip for 36 h. The residue was purified by flash column chromatography (10-20% acetone in hexane) to afford product (84.0 mg, 99% yield, dr = 1.2/1 by ^{1}H NMR) as white foam solid. ^{1}H NMR (500 MHz, $CDCl_3$) δ 7.58 (s, br, 1H), 7.29-7.25 (m, 1H), 7.22-7.18 (m, 1H), 6.97-6.92 (m, 1H), 4.86-4.85 (m, 0.6H), 4.80 (br, 0.7H), 4.09 (br, 0.5H), 3.94 (br, 0.5H), 3.85 (br, 0.5H), 3.80 (0.6H), 3.69-3.67 (m, 3H), 1.58-1.56 (m, 9H), 1.34-1.33 (m, 9H), 1.06 (d, *J* = 30 Hz, 3H). ^{13}C NMR (126 MHz, $CDCl_3$) δ 171.6, 171.5, 155.4, 155.3, 153.0, 152.8, 142.7, 142.5, 129.0, 127.8, 127.4, 125.6, 125.4, 123.0, 122.8, 116.4, 116.0, 81.9, 79.1, 65.9, 65.0, 52.7, 52.7, 49.6, 48.8, 48.4, 28.4, 28.4, 28.4, 17.3, 16.1. HRMS (ESI) calcd for $[C_{22}H_{32}N_2O_6 + H]^+$: 421.2333, found: 421.2343.

The racemic product was prepared from Boc-DL-alanine. Two diastereomers were separated by preparative TLC (Hexane/EtOAc/DCM = 6:1:1). We didn't observe any significant enantiomeric excess for each diastereomer.

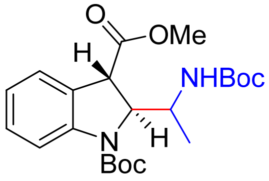

(±) *trans*-**3a**

(±) *trans*-**3a**-isomer-**a:** 6% ee. ^{1}H NMR (400 MHz, $CDCl_3$) ^{1}H NMR (400 MHz, $CDCl_3$) δ 7.60 (s, 2H), 7.31 (d, *J* = 7.4 Hz, 1H), 7.27 – 7.21 (m, 1H), 6.98 (t, *J* = 7.5 Hz, 1H), 4.87 (dd, *J* = 6.3, 2.2 Hz, 2H), 3.96 (s, 1H), 3.86 (s, 1H), 3.71 (s, 3H), 1.59 (s, 9H), 1.36 (s, 9H), 1.12 (d, *J* = 6.2 Hz, 3H). ^{13}C NMR (100 MHz, $CDCl_3$) δ 171.6, 155.4, 153.1, 142.6, 129.1, 127.8, 125.6, 123.2, 116.5, 82.1, 79.3, 65.2, 52.8, 49.8, 49.0, 28.5, 28.4, 17.5. HPLC: Daicel CHIRALPAK® IB; detected at 254 nm; *n*-hexane /*i*-propanol = 99/1; flow = 0.5 mL/min; Retention time: 17.262 min (minor), 19.575 min (major).

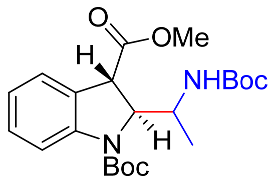

(±) *trans*-**3a**

(±) *trans*-**3a**-isomer-**b:** 0% ee. ^{1}H NMR (400 MHz, $CDCl_3$) δ 7.61 (s, 1H), 7.32 (d, *J* = 7.5 Hz, 1H), 7.22 (t, *J* = 7.6 Hz, 1H), 6.97 (t, *J* = 7.4 Hz, 1H), 4.82 (s, 2H), 4.09 (s, 1H), 3.80 (s, 1H), 3.72 (s, 3H), 1.60 (s, 10H), 1.36 (s, 9H), 1.05 (d, *J* = 6.7 Hz, 3H). ^{13}C NMR (101 MHz, $CDCl_3$) δ 171.5, 155.4, 152.8, 142.8, 129.1, 127.4, 125.5, 122.9, 116.1, 82.2, 79.4, 66.0, 52.8, 49.9, 48.7, 28.5, 28.5, 16.3. HPLC: Daicel CHIRALPAK® IB; detected at 254 nm; *n*-hexane /*i*-propanol = 97/3; flow = 0.5 mL/min; Retention time: 10.426 min, 12.140 min.

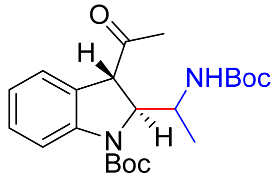

(±) *trans*-**3b**

(±) *trans*-**3b** was synthesized according to the general procedure **G**. *tert*-Butyl 3-acetyl-1*H*-indole-1-carboxylate[2] (52.0 mg, 0.2 mmol, 1.0 equiv), 4CzIPN (8.0 mg, 0.01 mmol, 5 mol%), Boc-L-alanine (49.0 mg, 0.26 mmol, 1.3 equiv), Cs_2CO_3 (65.0 mg, 0.2 mmol, 1.0 equiv) and DMF (2.0 mL) were used. The reaction mixture was irritated by 5w Blue LED strip for 36 h. The residue was purified by flash column chromatography (10-20% acetone in hexane) to afford product (88.8 mg, 93% yield, dr = 1.0/1 by ^{1}H NMR) as white foam solid. ^{1}H NMR (500 MHz, $CDCl_3$) δ 7.64 (s, br, 1H), 7.25-7.21 (m, 2H), 7.00-6.99 (m, 1H), 4.80 (d, *J* = 5.5 Hz, 0.6H), 4.73 (s, 0.4H), 4.05 (br, 0.4H), 3.95 (s, 0.5H), 3.82 (s, 0.5H), 3.78 (s, 0.5H), 2.18 (s, 1.4H), 2.16 (ds, 1.4H), 1.59 (s, 4.3H), 1.58 (s, 4.6H), 1.35 (s, 4.9H), 1.34 (s, 4.1H), 1.09 (d, *J* = 6.0 Hz, 1.4H), 1.03 (d, *J* = 7.0 Hz, 1.6H) ^{13}C NMR (126 MHz, $CDCl_3$) δ 204.5, 204.2, 155.4, 155.3, 152.8, 142.8, 129.1, 125.1, 124.9, 123.2, 123.0, 116.7, 116.4, 82.1, 79.3, 65.3, 64.4, 57.4, 57.2, 49.8, 49.4, 28.5, 28.5, 28.4, 28.4, 27.1, 27.0, 17.3, 16.3. HRMS (ESI) calcd for $[C_{22}H_{32}N_2O_5 + H]^+$: 405.2384, found: 405.2386.

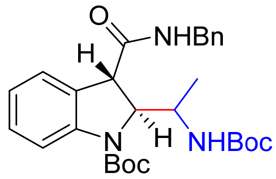

(±) *trans*-**3c**

(±) *trans*-**3c** was synthesized according to the general procedure **G**. *tert*-Butyl 3-(benzylcarbamoyl)-1*H*-indole-1-carboxylate (70.0 mg, 0.2 mmol, 1.0 equiv), 4CzIPN (8.0 mg, 0.01 mmol, 5 mol%), Boc-L-alanine (49.0 mg, 0.26 mmol, 1.3 equiv), Cs_2CO_3 (65.0 mg, 0.2 mmol, 1.0 equiv) and DMF (2.0 mL) were used. The reaction mixture was irritated by 5w Blue LED strip for 36 h. The residue was purified by flash column chromatography (Hexane/EtOAc/DCM = 3/1/1) to afford product (70.4 mg, 71% yield, dr = 4.0/1 by ^{1}H NMR) as light-yellow oil. **Major** isomer: ^{1}H NMR (400 MHz, $CDCl_3$) δ 7.68 (s, br, 1H), 7.31–7.10 (m, 7H), 6.97 (t, *J* = 7.4 Hz, 1H), 5.96 (s, 1H), 4.70 (s, br, 1H), 4.60 (s, 1H), 4.37 (ddd, *J* = 35.3, 15.0, 5.7 Hz, 2H), 4.14 (s, br, 1H), 3.73

(s, 1H), 1.56 (s, 9H), 1.29 (s, 9H), 1.13 (d, J = 6.7 Hz, 3H). ^{13}C NMR (100 MHz, $CDCl_3$) δ 171.3, 155.4, 138.0, 129.5, 128.8, 127.6, 127.4, 125.0, 123.2, 116.51, 82.12, 79.32, 68.22, 50.14, 49.64, 43.77, 28.5, 28.4, 16.62. **Minor** isomer: ^{1}H NMR (400 MHz, $CDCl_3$) δ 7.69 (s, br, 1H), 7.31–7.11 (m, 7H), 6.99 (t, J = 7.5 Hz, 1H), 5.87 (s, 1H), 5.09 (s, 1H), 4.63 (s, 1H), 4.38 (ddd, J = 44.6, 15.0, 5.7 Hz, 2H), 4.00 (s, 1H), 3.79 (s, 1H), 1.55 (s, 9H), 1.36 (s, 8H), 1.12 (d, J = 6.5 Hz, 3H). ^{13}C NMR (100 MHz, $CDCl_3$) δ 171.3, 155.4, 137.9, 129.7, 128.9, 127.7, 127.4, 125.2, 123.6, 116.9, 82.3, 79.3, 67.2, 50.7, 49.6, 43.7, 28.5, 16.8. HRMS (ESI) calcd for $[C_{28}H_{37}N_3O_5 + H]^+$: 496.2806, found: 496.2806.

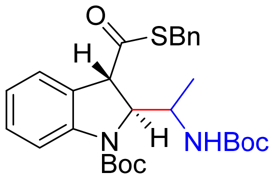

(±) *trans*-**3d**

(±) *trans*-**3d** was synthesized according to the general procedure **G**. *tert*-Butyl 3-((benzylthio)carbonyl)-1*H*-indole-1-carboxylate[3] (73.4 mg, 0.2 mmol, 1.0 equiv), 4CzIPN (8.0 mg, 0.01 mmol, 5 mol%), Boc-L-alanine (49.0 mg, 0.26 mmol, 1.3 equiv), Cs_2CO_3 (65.0 mg, 0.2 mmol, 1.0 equiv) and DMF (2.0 mL) were used. The reaction mixture was irritated by 5w Blue LED strip for 36 h. The residue was purified by flash column chromatography (10-20% acetone in hexane) to afford product (77.9 mg, 76% yield, dr = 1.3/1 by ^{1}H NMR) white solid. ^{1}H NMR (500 MHz, $CDCl_3$) δ 7.63 (br, 1H), 7.35-7.25 (m, 7H), 7.00 (t, J = 7.5 Hz, 1H), 4.80 (d, J = 6.0 Hz, 0.5H), 4.73 (s, 0.6H), 4.12 (d, J = 4.0 Hz, 2H), 4.00 (br, 0.5H), 3.97 (s, 0.7H), 1.61 (s, 5.1H), 1.60 (s, 4.0H), 1.39 (s, 4.0H), 1.37 (s, 5.0H), 1.14 (d, J = 7.0 Hz, 1.1H), 1.11 (d, J = 6.5 Hz, 2.0H) ^{13}C NMR (126 MHz, $CDCl_3$) δ 197.9, 197.8, 155.4, 152.6, 143.3, 137.0, 136.9, 129.6, 129.6, 129.0, 128.9, 128.8, 127.6, 127.6, 125.9, 125.8, 123.1, 122.9, 116.7, 116.4, 82.1, 79.4, 66.9, 66.0, 57.0, 56.6, 49.5, 33.7, 33.7, 28.4, 28.4, 28.3, 17.2, 16.4. HRMS (ESI) calcd for $[C_{28}H_{36}N_2O_5S + Na]^+$: 535.2243, found: 535.2237.

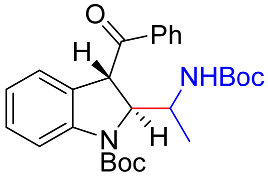

(±) *trans*-**3e**

(±) *trans*-**3e** was synthesized according to the general procedure **G**. *tert*-Butyl 3-benzoyl-1*H*-indole-1-carboxylate[4] (64.2 mg, 0.2 mmol, 1.0 equiv), 4CzIPN (8.0 mg, 0.01 mmol, 5 mol%), Boc-L-alanine (49.0 mg, 0.26 mmol, 1.3 equiv), Cs_2CO_3 (65.0 mg, 0.2 mmol, 1.0 equiv) and DMF (2.0 mL) were used. The reaction mixture was irritated by 5w Blue LED strip for 36 h. The residue was purified by flash column chromatography (10-20% EtOAc in hexane) to afford product (60.6 mg, 65% yield, dr = 2.1/1 by ^{1}H NMR) as light-yellow oil. ^{1}H NMR (500 MHz, $CDCl_3$) δ 8.03-8.01 (m, 2H), 7.80-7.55 (m, 2H), 7.53-7.49 (m, 2H), 7.18-7.15 (m, 1H), 6.94-6.92 (m, 1H), 6.83-6.79 (m, 1H), 4.98-4.87 (m, 2H), 4.10-4.03 (m, 1H), 1.62-1.60 (s, s, 9H), 1.39 (s, 6H), 1.33 (s, 3H), 1.20 (d, J = 6.0 Hz, 1H), 1.11 (d, J = 6.5 Hz, 2H). ^{13}C NMR (126 MHz, $CDCl_3$) δ 196.8, 196.6, 155.7, 153.2, 143.2, 136.0, 133.8, 133.7, 129.3, 129.3, 129.1, 128.9, 128.3, 125.1, 123.0, 122.8,

117.1, 116.7, 82.1, 81.9, 79.4, 79.2, 66.0, 65.4, 51.5, 51.4, 50.4, 50.3, 28.4, 28.4, 28.4, 28.3, 18.2, 16.7. HRMS (ESI) calcd for $[C_{27}H_{34}N_2O_5 + H]^+$: 467.2540, found: 467.2542.

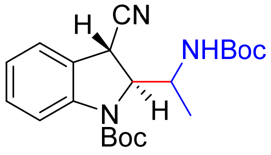

(±) *trans*-**3f**

(±) *trans*-**3f** was synthesized according to the general procedure **G**. *tert*-Butyl 3-cyano-1*H*-indole-1-carboxylate[2] (55.0 mg, 0.2 mmol, 1.0 equiv), 4CzIPN (8.0 mg, 0.01 mmol, 5 mol%), Boc-L-alanine (49.0 mg, 0.26 mmol, 1.3 equiv), Cs_2CO_3 (65.0 mg, 0.2 mmol, 1.0 equiv) and DMF (2.0 mL) were used. The reaction mixture was irritated by 5w Blue LED strip for 36 h. The residue was purified by flash column chromatography (10-20% acetone in hexane) to afford product (77.4 mg, 99% yield, dr = 1.7/1 by ^{1}H NMR) as white foam solid. ^{1}H NMR (400 MHz, Acetone-d6) δ 7.75 (s, br, 1H), 7.42-7.27 (m, 2H), 7.09-7.01 (m, 1H), 6.11 (br, 0.3H), 5.96 (d, J = 8.0 Hz, 0.6H), 4.91 (dd, J = 4.8 Hz, 2.8 Hz, 0.4H), 4.67 (dd, J = 3.6 Hz, 2.8 Hz, 0.6H), 4.53 (d, J = 2.4 Hz, 0.4H), 4.48 (d, J = 2.4 Hz, 0.6H), 1.61 (s, 9H), 1.38 (s, 9H), 1.26 (d, J = 7.2 Hz, 2H), 1.26 (d, J = 7.2 Hz, 2H), 1.01 (d, J = 7.2 Hz, 1H). ^{13}C NMR (126 MHz, $CDCl_3$) δ 155.4, 155.2, 152.3, 142.3, 125.1, 125.0, 123.8, 123.7, 119.1, 119.0, 116.7, 116.5. 82.8, 82.7, 79.9, 79.8, 67.8, 66.4, 49.7, 49.0, 33.4, 32.7, 17.2, 16.9. HRMS (ESI) calcd for $[C_{21}H_{29}N_3O_4 + H]^+$: 388.2231, found: 388.2243.

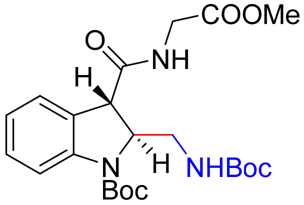

(±) *trans*-**3g**

(±) *trans*-**3g** was synthesized according to the general procedure **G**. *tert*-Butyl 3-((2-methoxy-2-oxoethyl)carbamoyl)-1*H*-indole-1-carboxylate (66.4 mg, 0.2 mmol, 1.0 equiv), 4CzIPN (8.0 mg, 0.01 mmol, 5 mol%), Boc-glycine (45.5 mg, 0.26 mmol, 1.3 equiv), Cs_2CO_3 (65.0 mg, 0.2 mmol, 1.0 equiv) and DMF (2.0 mL) were used. The reaction mixture was irritated by two 40w Blue LED lamps for 36 h. The residue was purified by flash column chromatography (20-50% EtOAc in hexane) to afford product (87.1 mg, 94% yield) as white foam solid. ^{1}H NMR (400 MHz, $CDCl_3$) δ 7.70 (s, br, 1H), 7.25 (d, J = 6.7 Hz, 2H), 7.00 (t, J = 7.4 Hz, 1H), 6.48 (s, 1H), 4.98 (s, 1H), 4.66 (s, 1H), 4.09–4.00 (m, 1H), 3.94–3.88 (m, 2H), 3.69 (s, 3H), 3.50–3.45 (m, 2H), 1.54 (s, 9H), 1.36 (s, 9H). ^{13}C NMR (100 MHz, $CDCl_3$) δ 171.5, 170.0, 156.5, 152.1, 142.8, 129.3, 127.4, 125.3, 123.2, 116.0, 82.0, 79.6, 63.6, 52.4, 50.8, 43.8, 41.5, 28.4, 28.4. HRMS (ESI) calcd for $[C_{23}H_{33}N_3O_7 + H]^+$: 464.2391, found: 464.2394.

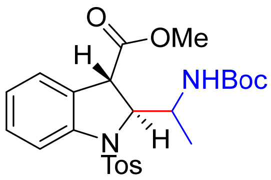

(±) *trans*-**3h**

(±) *trans*-**3h** was synthesized according to the general procedure **G**. Methyl 1-tosyl-1*H*-indole-3-carboxylate[2] (66.0 mg, 0.2 mmol, 1.0 equiv), 4CzIPN (8.0 mg, 0.01 mmol, 5 mol%), Boc-L-alanine (49.0 mg, 0.26 mmol, 1.3 equiv), Cs2CO3 (65.0 mg, 0.2 mmol, 1.0 equiv) and DMF (2.0 mL) were used. The reaction mixture was irritated by 5w Blue LED strip for 36 h. The residue was purified by flash column chromatography (25% EtOAc in hexane) to afford product (77.8 mg, 82% yield, dr = 1.0/1 by 1H NMR) as white foam solid. 1H NMR (400 MHz, CDCl3) δ 7.67 (t, J = 9.2 Hz, 1H), 7.45-7.44 (m, 2H), 7.27-7.20 (m, 2H), 7.10-7.02 (m, 3H), 5.40 (br, 0.4H), 4.67-4.65 (s, s, 1.6H), 4.00 (s, 0.5H), 3.90 (s, 0.5H), 3.74 (s, 0.5H), 3.64 (d, *J* = 1.6 Hz, 0.5H), 3.35-3.34 (s, s, 3H), 2.29-2.28 (s, s, 3H), 1.43 (s, 4.5H), 1.28 (br, 6.5H), 1.05 (d, *J* = 6.4 Hz). ^{13}C NMR (100 MHz, $CDCl_3$) δ 170.4, 170.2, 155.5, 144.1, 144.0, 142.1, 142.1, 133. 9, 129.9, 129.4, 129.3, 129.3, 129.0, 127.8, 127.8, 126.6, 125.3, 125.1, 117.6, 79.4, 79.3, 67.8, 67.6, 52.4, 52.3, 51.2, 50.9, 50.1, 49.5, 28.4, 28.3, 21.5, 17.7, 15.4. HRMS (ESI) calcd for $[C_{24}H_{30}N_2O_6S + H]^+$: 475.1897, found: 475.1897.

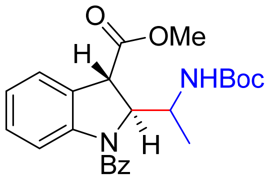

(±) *trans*-**3i**

(±) *trans*-**3i** was synthesized according to the general procedure **G**. 1-Benzyl 3-methyl 1*H*-indole-1,3-dicarboxylate[2] (55.8 mg, 0.2 mmol, 1.0 equiv), 4CzIPN (8.0 mg, 0.01 mmol, 5 mol%), Boc-L-alanine (49.0 mg, 0.26 mmol, 1.3 equiv), Cs_2CO_3 (65.0 mg, 0.2 mmol, 1.0 equiv) and DMF (2.0 mL) were used. The reaction mixture was irritated by 5w Blue LED strip for 36 h. The residue was purified by flash column chromatography (Hexane/EtOAc/DCM = 3/1/1) to afford product (60.0 mg, 73% yield, dr = 1.5/1) as white solid. ^{1}H NMR (400 MHz, $CDCl_3$) δ 7.45–7.29 (m, 6H), 7.27–7.24 (m, 1H), 6.91–6.88(m, 2H), 4.99 (s, 1H), 3.96 (s, br, 1H), 3.77 (s, 1H), 3.70-3.66 (m, 4H), 1.27 (s, 3.8H), 1.25 (s, 5.5H), 1.04 (s, 1.46H), 0.88 (d, *J* = 6.2 Hz, 2H). ^{13}C NMR (100 MHz, $CDCl_3$) δ 171.5, 171.5, 169.9, 169.7, 155.4, 142.8, 142.3, 136.1, 135.9, 130.7, 130.7, 128.7, 128.7, 127.8, 127.8, 125.9, 125.6, 124.3, 124.1, 117.3, 116.7, 79.3, 68.0, 67.6, 52.9, 52.9, 50.1, 49.5, 48.9, 29.8, 28.5, 18.3, 16.4. HRMS (ESI) calcd for $C_{24}H_{28}N_2NaO_5$ $[M + Na]^+$ 447.1896, found 447.1890.

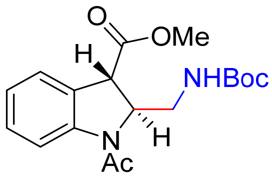

(±) *trans*-**3**j

(±) *trans*-**3j** was synthesized according to the general procedure **G**. Methyl 1-acetyl-1*H*-indole-3-carboxylate (43.4 mg, 0.2 mmol, 1.0 equiv), 4CzIPN (8.0 mg, 0.01 mmol, 5 mol%), Boc-glycine (45.5 mg, 0.26 mmol, 1.3 equiv), Cs_2CO_3 (65.0 mg, 0.2 mmol, 1.0 equiv) and DMF (2.0 mL) were used. The reaction mixture was irritated by 5w Blue LED strip for 36 h. The residue was purified by flash column chromatography (Hexane/EtOAc = 1/1) to afford product (52.0 mg, 74% yield) as light-yellow oil. ^{1}H NMR (400 MHz, $CDCl_3$) δ 8.07 (s, 1H), 7.38 (d, *J* = 5.4 Hz, 1H), 7.25 (t, *J* = 7.5 Hz, 1H), 7.05 (t, *J* = 7.4 Hz, 1H), 5.15 (s, 1H), 4.93 (s, 1H), 3.89 (s, 1H), 3.68 (s, 3H), 3.34 (t, *J* = 7.2 Hz, 1H), 3.15 (s, 1H), 2.37 (s, 3H), 1.38 (s, 9H). ^{13}C NMR (100 MHz, $CDCl_3$) δ 171.1,

169.2, 156.2, 141.9, 129.2, 125.9, 124.3, 118.3, 115.3, 80.0, 62.2, 52.8, 49.6, 43.6, 28.4, 23.4. HRMS (ESI) calcd for $[C_{18}H_{24}N_2O_5 + H]^+$: 349.1758, found: 375.1757.

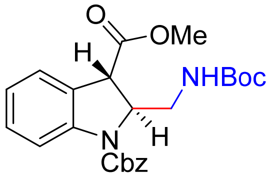

(±) *trans*-**3k**

(±) *trans*-**3k** was synthesized according to the general procedure **G**. 1-Benzyl 3-methyl 1*H*-indole-1,3-dicarboxylate[2] **1k** (61.8 mg, 0.2 mmol, 1.0 equiv), 4CzIPN (8.0 mg, 0.01 mmol, 5 mol%), Boc-glycine (45.5 mg, 0.26 mmol, 1.3 equiv), Cs_2CO_3 (65.0 mg, 0.2 mmol, 1.0 equiv) and DMF (2.0 mL) were used. The reaction mixture was irritated by 5w Blue LED strip for 36 h. The residue was purified by flash column chromatography (10-25% acetone in hexane) to afford product (88.0 mg, 99% yield) as white solid. ^{1}H NMR (400 MHz, $CDCl_3$) δ 7.82 (s, br, 1H), 7.44–7.43 (m, 2H), 7.40–7.34 (m, 4H), 7.23 (t, *J* = 6.2 Hz, 1H), 7.00 (t, *J* = 7.5 Hz, 1H), 5.29 (s, 2H), 4.98 (q, *J* = 2.4 Hz, 1H), 4.85 (s, br, 1H), 4.11 (s, 1H), 3.69 (s, s, 3H), 3.47 (s, 2H), 1.36 (s, 9H). ^{13}C NMR (100 MHz, $CDCl_3$) δ 171.4, 156.3, 152.8, 141.7, 135.9, 129.1, 128.7, 128.5, 128.4, 128.2, 125.9, 123.3, 115.7, 79.6, 67.8, 62.1, 52.7, 49.2, 43.4, 28.3. HRMS (ESI) calcd for $[C_{24}H_{28}N_2O_6 + H]^+$: 441.2020, found: 441.2022.

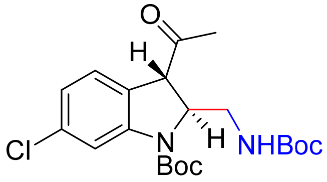

(±) *trans*-**3l**

(±) *trans*-**3l** was synthesized according to the general procedure **G**. *tert*-Butyl 3-acetyl-6-chloro-1*H*-indole-1-carboxylate (58.7 mg, 0.2 mmol, 1.0 equiv), 4CzIPN (8.0 mg, 0.01 mmol, 5 mol%), Boc-glycine (45.5 mg, 0.26 mmol, 1.3 equiv), Cs_2CO_3 (65.0 mg, 0.2 mmol, 1.0 equiv) and DMF (2.0 mL) were used. The reaction mixture was irritated by 5w Blue LED strip for 36 h. The residue was purified by flash column chromatography (25-40% EtOAc in hexane) to afford product (81.6 mg, 96% yield) as white solid. ^{1}H NMR (400 MHz, $CDCl_3$) δ 7.80 (s, 1H), 7.16 (d, *J* = 8.0 Hz, 1H), 6.95 (dd, *J* = 8.0, 1.7 Hz, 1H), 4.81 (s, 2H), 3.97 (s, 1H), 3.42-3.38 (m, 2H), 2.20 (s, 3H), 1.56 (s, 9H), 1.36 (s, 9H). ^{13}C NMR (101 MHz, $CDCl_3$) δ 203.7, 156.3, 151.7, 143.5, 134.8, 125.9, 122.9, 116.5, 82.6, 76.8, 57.0, 43.4, 28.4, 28.4, 27.5. HRMS (ESI) calcd for $[C_{21}H_{29}ClN_2O_5 + Na]^+$: 447.1657, found 447.1656.

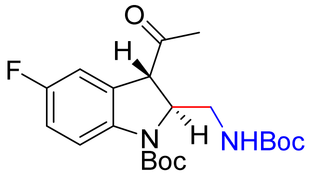

(±) *trans*-**3m**

(±) *trans*-**3m** was synthesized according to the general procedure **G**. *tert*-Butyl 3-acetyl-5-fluoro-1*H*-indole-1-carboxylate (55.4 mg, 0.2 mmol, 1.0 equiv), 4CzIPN (8.0 mg, 0.01 mmol, 5 mol%),

Boc-glycine (45.5 mg, 0.26 mmol, 1.3 equiv), Cs_2CO_3 (65.0 mg, 0.2 mmol, 1.0 equiv) and DMF (2.0 mL) were used. The reaction mixture was irritated by two 40w Blue lamps for 36 h. The residue was purified by flash column chromatography (hexane/DCM/EtOAc = 3/1/1) to afford product (83.0 mg, 98% yield) as light yellow oil. ^{1}H NMR (400 MHz, cdcl$_3$) δ 7.71 (s, br, 1H), 7.98–6.90 (m, 2H), 4.81 (s, 2H), 3.98 (s, 1H), 3.44–3.38 (m, 2H), 2.21 (s, 3H), 1.55 (s, 9H), 1.36 (s, 9H). ^{13}C NMR (100 MHz, cdcl$_3$) δ 203.5 (s), 158.8 (d, J = 240.4 Hz), 160.0, 157.6, 156.3, 152.0, 138.4, 129.0, 116.76 (d, J = 7.8 Hz), 115.54 (s), 115.4 (d, J = 22.7 Hz), 112.66 (d, J = 25.7 Hz), 82.1, 79.7, 61.7, 57.4, 43.5, 28.5, 28.4, 27.5. HRMS (ESI) calcd for $[C_{25}H_{32}N_2O_4 + Na]^+$: 447.2254, found 447.2252.

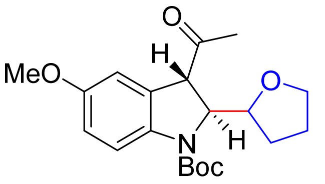

(±) *trans*-**3n**

(±) *trans*-**3n** was synthesized according to the general procedure **G**. *tert*-Butyl 3-acetyl-5-methoxy-1*H*-indole-1-carboxylate[5] (57.9 mg, 0.2 mmol, 1.0 equiv), 4CzIPN (8.0 mg, 0.01 mmol, 5 mol%), tetrahydrofuran-2-carboxylic acid (41.8 mg, 0.26 mmol, 1.3 equiv), Cs_2CO_3 (65.0 mg, 0.2 mmol, 1.0 equiv) and DMF (2.0 mL) were used. The reaction mixture was irritated by 5w Blue LED strip for 36 h. The residue was purified by flash column chromatography (25% acetone in hexane) to afford product (70.0 mg, 97% yield, dr = 1.6/1) as light yellow oil. ^{1}H NMR (500 MHz, $CDCl_3$) δ 7.67-7.35 (s, br, s, br, 1H), 6.81–6.74 (m, 2H), 4.87 (d, J = 2.9 Hz, 0.37H), 4.69 (d, J = 5.0 Hz, 0.60 H), 4.21-4.19 (m, 1H), 3.96 (s, 1H), 3.85–3.79 (m, 1H), 3.78–3.67 (m, 5H), 2.17-2.17 (s, s, 3H), 2.17 (s, 2H), 1.93-1.81 (m, 4H), 1.54-1.54 (s, s, 9H). ^{13}C NMR (125 MHz, $CDCl_3$) δ 205.2, 204.9, 156.02, 155.93, 152.7, 136.4, 129.8, 117.3, 116.9, 113.5, 111.6, 111.2, 81.3, 79.5, 78.9, 68.6, 68.6, 64.2, 63.2, 55.9, 55.7, 55.7, 28.4, 27.1, 26.4, 25.7, 25.5. HRMS (ESI) calcd for $[C_{20}H_{27}N_2O_5 + Na]^+$: 384.1781, found 384.1781.

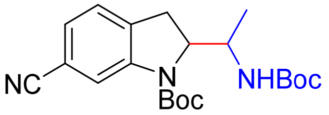

(±) **3o**

(±) **3o** was synthesized according to the general procedure **G**. *tert*-Butyl 6-cyano-1*H*-indole-1-carboxylate[6] (55.6 mg, 0.2 mmol, 1.0 equiv), 4CzIPN (8.0 mg, 0.01 mmol, 5 mol%), Boc-L-alanine (49.0 mg, 0.26 mmol, 1.3 equiv), Cs_2CO_3 (65.0 mg, 0.2 mmol, 1.0 equiv) and DMF (2.0 mL) were used. The reaction mixture was irritated by 5w Blue LED strip for 36 h. The residue was purified by flash column chromatography (25% acetone in hexane) to afford product (50.0 mg, 65% yield, dr = 2.5/1) as colorless oil. ^{1}H NMR (400 MHz, $CDCl_3$) δ 7.85 (br, 1H), 7.23-7.16 (m, 2H), 4.76 (br, 1H), 4.54-4.45 (m, 1H), 4.15 (s, 0.7H), 3.88 (br, 0.3H), 3.36-3.27 (m, 1H), 2.90-2.80 (m, 1H), 1.59-1.58 (s, s, 9H), 1.36 (s, 2.8H), 1.31 (s, 6.2H), 1.07 (d, J = 6.8 Hz, 0.85H), 1.02 (d, J = 6.8 Hz, 2.12H). ^{13}C NMR (100 MHz, $CDCl_3$) δ 155.3, 152.3, 143.7, 136.1, 127.2, 126.7, 125.2, 125.0, 119.3, 118.8, 118.2, 111.2, 111.1, 82.7, 79.3, 63.5, 62.5, 49.4, 31.6, 30.6, 28.4, 28.4, 28.4, 28.4, 16.2. HRMS (ESI) calcd for $[C_{21}H_{29}N_3O_4 + Na]^+$: 410.2050, found 410.2041.

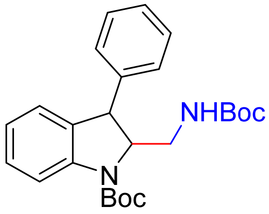

(±) **3p**

(±) **3p** was synthesized according to the general procedure **G**. *tert*-Butyl 3-phenyl-1H-indole-1-carboxylate[7] (48.6 mg, 0.2 mmol, 1.0 equiv), 4CzIPN (8.0 mg, 0.01 mmol, 5 mol%), Boc-glycine (45.5 mg, 0.26 mmol, 1.3 equiv), Cs_2CO_3 (65.0 mg, 0.2 mmol, 1.0 equiv) and DMF (2.0 mL) were used. The reaction mixture was irritated by 5w Blue LED strip for 36 h. The residue was purified by preparative TLC (10% acetone in hexane) to afford product as colorless oil (**isomer a** 29.3 mg, **isomer b** 26.7 mg, 88% yield in total, dr = 1.1/1). **Isomer a**: ^{1}H NMR (400 MHz, $CDCl_3$) δ 7.66 (s, 1H), 7.35-7.21 (m, 7H), 7.06–6.97 (m, 2H), 4.92 (d, *J* = 9.1 Hz, 1H), 4.76 (s, 1H), 3.12–3.05 (m, 1H), 2.79– 2.74 (m, 1H), 1.59 (s, 9H), 1.33 (s, 9H). ^{13}C NMR (101 MHz, $CDCl_3$) δ 155.8, 142.4, 136.5, 132.7, 129.7, 129.0, 128.2, 127.9, 125.3, 123.1, 116.6, 82.0, 79.0, 77.5, 77.2, 76.8, 63.2, 50.2, 42.2, 28.5. **Isomer b**: ^{1}H NMR (400 MHz, $CDCl_3$) δ 7.72 (s, 1H), 7.30–7.15 (m, 5H), 7.06–6.93 (m, 4H), 4.91 (s, br, 1H), 4.33 (s, 1H), 4.22 (s, 1H), 3.51 (t, *J* = 5.1 Hz, 2H), 1.56 (s, 9H), 1.41 (s, 9H). ^{13}C NMR (100 MHz, $CDCl_3$) δ 156.3, 152.7, 144.0, 142.1, 133.4, 128.9, 128.2, 127.4, 127.1, 125.8, 123.3, 115.7, 81.9, 68.2, 50.1, 44.1, 28.5, 28.5. HRMS (ESI) calcd for $[C_{25}H_{32}N_2O_4 + Na]^+$: 447.2254, found 447.2252.

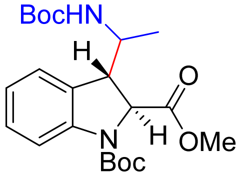

(±) *trans*-**3q**

(±) *trans*-**3q** was synthesized according to the general procedure **G**. 1-(*tert*-Butyl) 2-methyl 1*H*-indole-1,2-dicarboxylate[8] (55.6 mg, 0.2 mmol, 1.0 equiv), 4CzIPN (8.0 mg, 0.01 mmol, 5 mol%), Boc-L-alanine (49.0 mg, 0.26 mmol, 1.3 equiv), Cs_2CO_3 (65.0 mg, 0.2 mmol, 1.0 equiv) and DMF (2.0 mL) were used. The reaction mixture was irritated by 5w Blue LED strip for 36 h. The residue was purified by flash column chromatography (15% acetone in hexane) to afford product (68.0 mg, 81% yield, dr = 1.5/1) as white solid. ^{1}H NMR (400 MHz, $CDCl_3$) δ 7.90 (d, *J* = 6.8 Hz, 0.7H), 7.47 (d, *J* = 6.4 Hz, 0.3H), 7.23-7.20 (m, 1H), 7.12 (d, *J* = 6.4 Hz, 1H), 6.95-6.91 (m, 1H), 4.74-4.56 (m, 2H), 4.06 (br, 0.4H), 3.89 (br, 0.6H), 3.70-3.70 (s, s, 3H), 3.62 (br, 0.4H), 3.45-3.44 (m, 0.6H), 1.58 (br, 3.9H), 1.47 (s, 5.1H), 1.42 (s, 9H), 1.04-1.03 (m, 3H). ^{13}C NMR (100 MHz, $CDCl_3$) δ 172.0, 171.8, 155.2, 155.0, 151.4, 142.2, 143.1, 128.7, 128.6, 128.0, 127.9, 125.6, 125.1, 124.5, 122.5, 122.3, 114.7, 114.6, 81.4, 79.5, 63.8, 63.1, 62.8, 52.3, 49.9, 49.7, 49.4, 28.3, 28.2, 16.6, 16.2. HRMS (ESI) calcd for $[C_{22}H_{32}N_2O_6 + Na]^+$: 443.2153, found 443.2146.

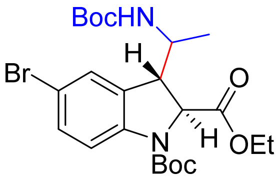

(±) *trans*-**3r**

(±) *trans*-**3r** was synthesized according to the general procedure **G**. 1-(*tert*-Butyl) 2-ethyl 5-bromo-1*H*-indole-1,2-dicarboxylate (73.6 mg, 0.2 mmol, 1.0 equiv), 4CzIPN (8.0 mg, 0.01 mmol, 5 mol%), Boc-L-alanine (49.0 mg, 0.26 mmol, 1.3 equiv), Cs_2CO_3 (65.0 mg, 0.2 mmol, 1.0 equiv) and DMF (2.0 mL) were used. The reaction mixture was irritated by 5w Blue LED strip for 36 h. The residue was purified by flash column chromatography (Hexane/Acetone = 5/1) to afford product (93.0 mg, 91% yield, dr = 2.5/1) as white solid. ^{1}H NMR (500 MHz, $CDCl_3$) δ 7.78 (d, *J* = 8.5 Hz, 0.6H), 7.30 (d, *J* = 7.5 Hz, 1.3H), 7.25-7.22 (s, s, 1H), 4.71-4.53 (m, 2H), 4.19-4.16 (m, 2H), 4.02-3.90 (s, br, s, br, 1H), 3.54 (s, 0.3H), 3.42 (s, 0.6H), 1.57 (s, 3H), 1.47 (s, 6H), 1.42-1.42 (s, s, 9H), 1.25 (t, *J* = 7.0 Hz, 3H), 1.04 (d, *J* = 6.5 Hz, 3H). ^{13}C NMR (100 MHz, $CDCl_3$) δ 171.4, 171.2, 155.2, 151.5, 142.8, 142.7, 131.8, 131.7, 130.7, 130.6, 128.3, 127.8, 116.4, 116.2, 115.0, 114.8.81.9, 79.8, 64.0, 61.6, 49.7, 49.5, 28.3, 28.2, 16.6, 16.3, 14.2, 14.1. HRMS (ESI) calcd for $[C_{23}H_{33}BrN_2O_6+ Na]^+$: 535.1414, found 535.1412.

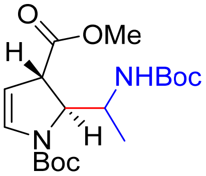

(±) *trans*-**3s**

(±) *trans*-**3s** was synthesized according to the general procedure **G**. 1-(*tert*-Butyl) 3-methyl 1*H*-pyrrole-1,3-dicarboxylate (45.0 mg, 0.2 mmol, 1.0 equiv), 4CzIPN (8.0 mg, 0.01 mmol, 5 mol%), Boc-L-alanine (49.0 mg, 0.26 mmol, 1.3 equiv), Cs_2CO_3 (65.0 mg, 0.2 mmol, 1.0 equiv) and DMF (2.0 mL) were used. The reaction mixture was irritated by 5w Blue LED strip for 36 h. The residue was purified by flash column chromatography (10% acetone in hexane) to afford product (70.3 mg, 95% yield, dr = 1.1/1) as light yellow oil. ^{1}H NMR (400 MHz, $CDCl_3$) δ 6.66-6.52 (s, br, s, br, 1H), 5.91 (s, br, 0.2H), 5.32 (s, br, 0.2H), 4.97-4.92 (s, s, 1H), 4.47 (s, 0.92), 3.92 (s, br, 1H), 3.67-3.67(m, 3H), 3.35 (s, br, 1H), 1.45-1.44 (s, s, br, 9H), 1.38-1.38 (s, s, 9H), 1.08 (s, br, 1.6H), 1.01(s, br, 1.4H). ^{13}C NMR (100 MHz, $CDCl_3$) δ 172.4, 172.2, 155.5, 132.6, 132.2, 104.7, 104.2, 81.4, 79.1, 63.8, 62.6, 52.6, 52.5, 52.5, 52.5, 51.9, 51.0, 50.5. 28.5, 28.5, 28.4, 28.4. 17.3, 16.0, 15.0. HRMS (ESI) calcd for $[C_{18}H_{30}N_2O_6+ Na]^+$: 393.1996, found 393.1994.

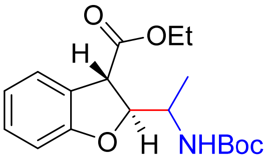

(±) *trans*-**3t**

(±) *trans*-**3t** was synthesized according to the general procedure **G**. Ethyl benzofuran-3-carboxylate[9] (38.0 mg, 0.2 mmol, 1.0 equiv), 4CzIPN (8.0 mg, 0.01 mmol, 5 mol%), Boc-L-alanine (49.0 mg, 0.26 mmol, 1.3 equiv), Cs_2CO_3 (65.0 mg, 0.2 mmol, 1.0 equiv) and DMF (2.0

mL) were used. The reaction mixture was irritated by 5w Blue LED strip for 36 h. The residue was purified by flash column chromatography (25% acetone in hexane) to afford product (70.0 mg, 99% yield, dr = 1.5/1) as colorless oil. ^{1}H NMR (400 MHz, $CDCl_3$) δ 7.33 (dd, J = 16 Hz, 7.6 Hz, 1H), 7.19-7.14 (m, 1H), 6.90-6.85 (m, 1H), 6.78 (dd, J = 8.0 Hz, 4.0 Hz, 1H), 5.05 (dd, J = 8.0 Hz, 1.6 Hz, 0.6H), 4.99 (dd, J = 6.8 Hz, 6.4 Hz, 0.5H), 4.76 (br, 0.4H), 4.59 (d, J = 8.4 Hz, 0.6H), 4.26-4.16 (m, 3H), 4.02 (br, 0.6H), 3.90 (br, 0.5H), 1.43 (s, 5H), 1.38 (s, 4H), 1.35 (d, J = 6.8 Hz, 1.6H), 1.31 (q, J = 6.4 Hz, 3H), 1.19 (d, J = 6.8Hz, 1.4H). ^{13}C NMR (100 MHz, $CDCl_3$) δ 171.1, 170.8, 159.4, 159.2, 155.8, 155.3, 129.5, 129.3, 125.4, 125.3, 124.6, 124.1, 120.9, 120.8, 109.7, 109.7, 87.2, 87.1, 79.7, 61.7, 50.2, 49.5, 48.9, 28.4, 28.4, 18.9, 16.4, 14.3. HRMS (ESI) calcd for $[C_{18}H_{25}NO_5 + Na]^+$: 358.1625, found 358.1618.

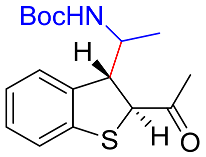

(±) *trans*-**3u**

(±) *trans*-**3u** was synthesized according to the general procedure **G**. Ethyl benzofuran-3-carboxylate (38.0 mg, 0.2 mmol, 1.0 equiv), 4CzIPN (8.0 mg, 0.01 mmol, 5 mol%), Boc-L-alanine (49.0 mg, 0.26 mmol, 1.3 equiv), Cs_2CO_3 (65.0 mg, 0.2 mmol, 1.0 equiv) and DMF (2.0 mL) were used. The reaction mixture was irritated by 5w Blue LED strip for 36 h. The residue was purified by flash column chromatography (10% acetone in hexane) to afford product (56.0 mg, 88% yield, dr = 1.1/1) as white solid. ^{1}H NMR (400 MHz, $CDCl_3$) δ 7.21–7.17 (m, 1H), 7.14-7.12 (m, 2H), 7.06-7.01 (m, 1H), 4.67-4.61 (m, 1H), 4.19–3.87 (m, 3H), 2.23 (d, J = 2.1 Hz, 3H), 1.41 (s, 9H), 1.10 (d, J = 6.7 Hz, 3H). ^{13}C NMR (101 MHz, $CDCl_3$) δ 203.3, 203.0, 155.4, 155.4, 139.4, 138.8, 138.4, 128.6, 128.6, 126.3, 126.0, 124.9, 122.2, 122.1, 79.8, 58.6, 58.1, 53.6, 53.1, 50.6, 49.1, 28.5, 28.5, 26.9, 26.5, 18.0, 17.2. HRMS (ESI) calcd for $[C_{17}H_{23}NO_3S + Na]^+$: 344.1291, found 344.1285.

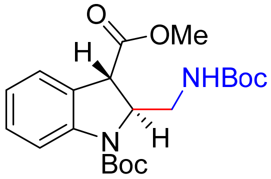

(±) *trans*-**3v**

(±) *trans*-**3v** was synthesized according to the general procedure **G**. 1-(*tert*-Butyl) 3-methyl 1*H*-indole-1,3-dicarboxylate (55.0 mg, 0.2 mmol, 1.0 equiv), 4CzIPN (8.0 mg, 0.01 mmol, 5 mol%), Boc-glycine (45.5 mg, 0.26 mmol, 1.3 equiv), Cs_2CO_3 (65.0 mg, 0.2 mmol, 1.0 equiv) and DMF (2.0 mL) were used. The reaction mixture was irritated by 5w Blue LED strip for 36 h. The residue was purified by flash column chromatography (10-20% EtOAc in hexane) to afford product (70.6 mg, 87% yield) as colorless oil. ^{1}H NMR (400 MHz, $CDCl_3$) δ 7.65 (br, 1H), 7.33 (d, J = 7.2 Hz, 1H), 7.22(t, J = 7.6 Hz, 1H), 6.97 (td, J = 7.2 Hz, 0.4 Hz, 1H), 4.87-4.85 (m, 1H), 4.01 (s, 1H), 3.71(s, 3H), 3.49-3.42 (m, 2H), 1.57 (s, 9H), 1.36 (s, 9H). ^{13}C NMR (100 MHz, $CDCl_3$) δ 171.6, 156.3, 152.2, 142.1, 129.0, 127.1, 125.9, 122.9, 115.7, 82.0, 79.6, 61.9, 52.7, 49.2, 43.7, 28.5, 28.4. HRMS (ESI) calcd for $[C_{21}H_{30}N_2O_6 + Na]^+$: 429.1996, found 429.1985.

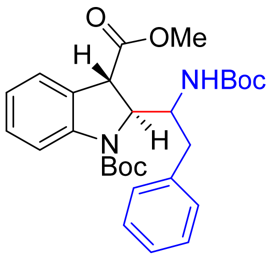

(±) *trans*-**3w**

(±) *trans*-**3w** was synthesized according to the general procedure **G**. 1-(*tert*-Butyl) 3-methyl 1*H*-indole-1,3-dicarboxylate (55.0 mg, 0.2 mmol, 1.0 equiv), 4CzIPN (8.0 mg, 0.01 mmol, 5 mol%), Boc-L-phenylalanine (25.5 mg, 0.26 mmol, 1.3 equiv), Cs_2CO_3 (65.0 mg, 0.2 mmol, 1.0 equiv) and DMF (2.0 mL) were used. The reaction mixture was irritated by 5w Blue LED strip for 36 h. The residue was purified by flash column chromatography (10% acetone in hexane) to afford product (99.2 mg, 99% yield, dr = 1.1/1 by ^{1}H NMR) as colorless oil.^{1}H NMR (400 MHz, $CDCl_3$) δ 7.62 (br, 1H), 7.33-7.15 (m, 7H), 7.01-6.93 (m, 1H), 5.00 (dd, *J* = 6.8 Hz, 2.4 Hz, 0.5H), 4.86 (br, 1H), 4.47 (br, 0.5H), 4.18 (br, 0.5H), 3.91(s, 1H), 3.69 (s, 3H), 2.96(d, *J* = 11.6Hz, 0.5H), 2.80 (d, *J* = 6.0 Hz), 2.64 (br, 0.5H), 1.56 (s, 4.2H), 1.54 (s, 4.8H), 1.29 (s, 4.5H), 1.23 (s, 5.1H). ^{13}C NMR (100 MHz, $CDCl_3$) δ 171.5, 171.4, 155.4, 152.8, 152.2, 142.5, 137.5, 137.5, 129.4, 129.2, 129.0, 128.5, 128.4, 126.5, 126.5, 125.5, 125.3, 123.1, 122.7, 116.4, 115.9, 81.9, 81.8, 79.2, 64.9, 64.1, 54.5, 54.2, 52.7, 52.7, 52.6, 49.4, 47.8, 37.5, 28.4, 28.4, 28.2. HRMS (ESI) calcd for $[C_{28}H_{36}N_2O_6+ Na]^+$: 519.2466, found: 519.2456.

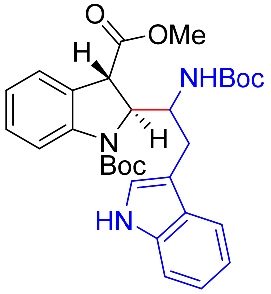

(±) *trans*-**3x**

(±) *trans*-**3x** was synthesized according to the general procedure **G**. 1-(*tert*-Butyl) 3-methyl 1*H*-indole-1,3-dicarboxylate (55.0 mg, 0.2 mmol, 1.0 equiv), 4CzIPN (8.0 mg, 0.01 mmol, 5 mol%), Boc-L-tryptophan (79.1 mg, 0.26 mmol, 1.3 equiv), Cs_2CO_3 (65.0 mg, 0.2 mmol, 1.0 equiv) and DMF (2.0 mL) were used. The reaction mixture was irritated by 5w Blue LED strip for 36 h. The residue was purified by flash column chromatography (25% EtOAc in hexane) to afford product (97.5 mg, 91% yield, dr = 1.0/1 by ^{1}H NMR) as light yellow oil. ^{1}H NMR (400 MHz, $CDCl_3$) δ 8.49 (s, 0.5H), 8.43 (s, 0.5H), 7.81-7.57 (m, 2H), 7.35-7.23 (m, 3H), 7.18-7.11 (m, 2H), 7.04-6.91 (m, 2H), 5.10 (dd, *J* = 7.2 Hz, 2.0 Hz, 0.5H), 5.00 (br, 1H), 4.64 (br, 0.5H), 4.30-4.23 (m, 0.5H), 3.96 (s, 1H), 3.67-3.65(s, s, 3H), 3.14-2.87(m, 2H), 1.53 (s, 9H), 1.35-1.26 (s, s, 9H) ^{13}C NMR (100 MHz, $CDCl_3$) δ 171.6, 171.6, 155.6, 152.9, 152.4, 142.5, 136.4, 136.3, 129.1, 129.0, 127.7, 127.6, 125.6, 125.2, 123.1, 122.9, 122.6, 121.9, 121.8, 119.3, 118.6, 116.4, 115.8, 111.3, 111.0, 110.7, 81.9, 79.2, 65.1, 64.0, 64.0, 53.8, 53.2, 52.7, 52.6, 52.6, 49.6, 47.6, 28.4, 28.3, 28.2, 27.0. HRMS (ESI) calcd for $[C_{30}H_{37}N_3O_6+ Na]^+$: 558.2575, found: 558.2565.

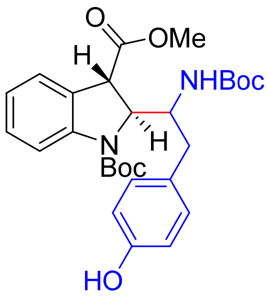

(±) *trans*-**3y**

(±) *trans*-**3y** was synthesized according to the general procedure **G**. 1-(*tert*-Butyl) 3-methyl 1*H*-indole-1,3-dicarboxylate (55.0 mg, 0.2 mmol, 1.0 equiv), 4CzIPN (8.0 mg, 0.01 mmol, 5 mol%), Boc-L-tyrosine (73.1 mg, 0.26 mmol, 1.3 equiv), Cs_2CO_3 (65.0 mg, 0.2 mmol, 1.0 equiv) and DMF (2.0 mL) were used. The reaction mixture was irritated by 5w Blue LED strip for 36 h. The residue was purified by flash column chromatography (25% EtOAc in hexane) to afford product (62.4 mg, 61% yield, dr = 1.2/1 by ^{1}H NMR) as light yellow oil. ^{1}H NMR (400 MHz, $CDCl_3$) δ 7.59 (br, 1H), 7.31-7.19 (m, 2H), 7.00-6.93 (m, 3H), 6.67-6.62 (m, 2H), 4.97 (dd, *J* = 6.8 Hz, 1.6 Hz, 0.5H), 4.87 (br, 0.6H), 4.45(br, 0.5H), 4.05 (br, 0.6H), 3.86 (s, 1H), 3.70 (s, 3H), 2.90-2.55 (m, 2H), 1.56-1.54 (s, s, 9H), 1.28-1.24 (s, s, 9H).^{13}C NMR (100 MHz, $CDCl_3$) δ 171.6, 171.6, 155.8, 155.2, 155.1, 142.4, 130.4, 130.1, 129.1, 128.6, 128.5, 127.7, 127.2, 125.7, 125.3, 123.3, 122.8, 116.5, 116.0, 115.5, 115.5, 82.2, 79.7, 65.1, 64.2, 55.3, 54.6, 52.9, 52.8, 49.7, 47.8, 37.1, 36.6, 28.5, 28.4, 28.3. HRMS (ESI) calcd for $[C_{28}H_{36}N_2O_7 + Na]^+$: 535.2415, found: 535.2419.

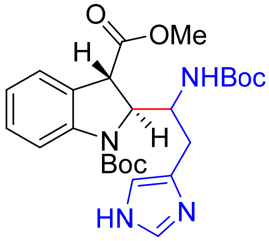

(±) *trans*-**3z**

(±) *trans*-**3z** was synthesized according to the general procedure **G**. 1-(*tert*-Butyl) 3-methyl 1*H*-indole-1,3-dicarboxylate (55.0 mg, 0.2 mmol, 1.0 equiv), 4CzIPN (8.0 mg, 0.01 mmol, 5 mol%), Boc-L-histidine (46.4 mg, 0.26 mmol, 1.3 equiv), Cs_2CO_3 (65.0 mg, 0.2 mmol, 1.0 equiv) and DMF (2.0 mL) were used. The reaction mixture was irritated by 5w Blue LED strip for 36 h. The residue was purified by flash column chromatography (EtOAc with 1% ammonia aqueous) to afford product (77.0 mg, 80% yield, dr = 1.1/1 by ^{1}H NMR) as light yellow foam solid. ^{1}H NMR (400 MHz, $CDCl_3$) δ 7.50 (d, *J* = 11.6 Hz, 2H), 7.28-7.17 (s, 2H), 6.95 (t, *J* = 11.6 Hz, 1H), 6.75 (s, 0.6H), 6.68 (s, br, 0.4H), 5.42 (s, br, 0.4H), 5.18 (s, br, 0.46), 4.99-4.98 (m, 0.5H), 4.81 (s, br, 0.5H), 4.06 (s, br, 1H), 3.85 (s, 1H), 3.66-3.64 (s, s, 3H), 2.89-2.71 (m, 2H), 1.56-1.54 (s, s, 9H), 1.31-1.28 (s, s, 9H). ^{13}C NMR (100 MHz, $CDCl_3$) δ 210.9, 171.6, 155.9, 155.7, 153.3, 142.3, 125.2, 135.0, 129.0, 128.9, 128.0, 125.7, 123.3, 116.5, 82.2, 79.6, 79.4, 64.9, 63.7, 54.0, 52.7, 52.7, 52.7, 49.2, 47.85, 28.4, 28.4, 28.3, 28.3. HRMS (ESI) calcd for $[C_{25}H_{34}N_4O_6 + Na]^+$: 487.2551, found: 487.2555.

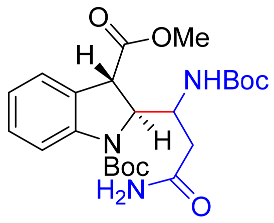

(±) *trans*-**3aa**

(±) *trans*-**3aa** was synthesized according to the general procedure **G**. 1-(*tert*-Butyl) 3-methyl 1*H*-indole-1,3-dicarboxylate (55.0 mg, 0.2 mmol, 1.0 equiv), 4CzIPN (8.0 mg, 0.01 mmol, 5 mol%), Boc-L- asparagine (51.0 mg, 0.26 mmol, 1.3 equiv), Cs_2CO_3 (65.0 mg, 0.2 mmol, 1.0 equiv) and DMF (2.0 mL) were used. The reaction mixture was irritated by 5w Blue LED strip for 36 h. The residue was purified by flash column chromatography (10% MeOH in EtOAc with 1% ammonia aqueous) to afford product (87.0 mg, 94% yield, dr = 1.3/1 by ^{1}H NMR) as light yellow foam solid. ^{1}H NMR (400 MHz, $CDCl_3$) δ 7.50 (br, 1H), 7.29 (t, *J* = 4.4 Hz, 1H), 7.19 (q, *J* = 4.8 Hz, 1H), 6.98-6.93 (m, 1H), 6.45 (br, 1H), 5.93-5.57 (m, 2H), 4.99 (d, *J* = 4.4 Hz, 0.4H), 4.90 (br, 0.6H), 4.27-4.20 (s, s, 1H), 4.01 (s, 1H), 3.66 (s, 3H), 2.50-2.32 (m, 2H), 1.55 (s, 9H), 1.31-1.30 (s, s, 9H). ^{13}C NMR (100 MHz, $CDCl_3$) δ 210.8, 173.2, 173.0, 171.6, 171.4, 156.0, 155.8, 152.8, 142.2, 129.0, 128.8, 127.7, 125.7, 123.3, 123.1, 116.4, 116.3, 82.2, 79.6, 65.2, 64.1, 54.0, 52.7, 52.7, 50.1, 48.9, 47.9, 36.9, 28.4, 28.4, 28.3. HRMS (ESI) calcd for $[C_{23}H_{33}N_3O_7 + H]^+$: 464.2391, found: 464.2396.

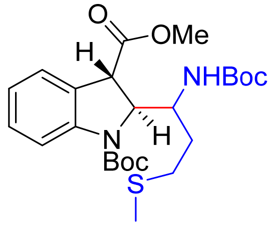

(±) *trans*-**3ab**

(±) *trans*-**3ab** was synthesized according to the general procedure **G**. 1-(*tert*-Butyl) 3-methyl 1*H*-indole-1,3-dicarboxylate (55.0 mg, 0.2 mmol, 1.0 equiv), 4CzIPN (8.0 mg, 0.01 mmol, 5 mol%), Boc-L-methionine (64.8 mg, 0.26 mmol, 1.3 equiv), Cs_2CO_3 (65.0 mg, 0.2 mmol, 1.0 equiv) and DMF (2.0 mL) were used. The reaction mixture was irritated by 5w Blue LED strip for 36 h. The residue was purified by flash column chromatography (25% Acetone in hexane) to afford product (80.0 mg, 83% yield, dr = 1.0/1 by ^{1}H NMR) as white foam solid. ^{1}H NMR (500 MHz, $CDCl_3$) δ 7.55 (s, br, 1H), 7.29 (d, *J* = 7.5 Hz, 1H), 7.20 (dd, *J* = 17.6, 7.7 Hz, 1H), 6.95 (dtd, *J* = 13.0, 7.5, 1.0 Hz, 1H), 4.88-4.68 (m, 2H), 4.20 (s, 0.39H), 3.94 (s, 0.44H), 3.89 (s, 0.42H), 3.85 (s, 0.5H), 3.69 (s, 1.50H), 3.68 (s, 1.49H), 2.59– 2.44 (m, 2H), 2.04-2.03 (s, s, 3H), 1.89-1.84 (m, 0.5H), 1.70-1.68 (m, 0.7H), 1.65–1.56 (m, 10H), 1.30 (s, s, 9H). ^{13}C NMR (125 MHz, $CDCl_3$) δ 171.6, 155.8, 142.6, 129.1, 127.9, 127.1, 125.7, 125.4, 123.2, 122.8, 116.4, 115.9, 82.1, 79.3, 65.5, 64.4, 53.7, 52.7, 52.6, 49.5, 48.0, 30.8, 30.7, 28.3, 28.2, 15.6, 15.3. HRMS (ESI) calcd for $[C_{24}H_{36}N_2O_6S + H]^+$: 481.2367, found: 481.2374.

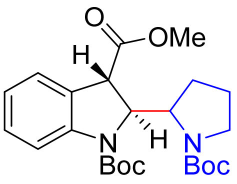

(±) *trans*-**3ac**

(±) *trans*-**3ac** was synthesized according to the general procedure **G**. 1-(*tert*-Butyl) 3-methyl 1*H*-indole-1,3-dicarboxylate (55.0 mg, 0.2 mmol, 1.0 equiv), 4CzIPN (8.0 mg, 0.01 mmol, 5 mol%), *N*-Boc proline (56.0 mg, 0.26 mmol, 1.3 equiv), Cs_2CO_3 (65.0 mg, 0.2 mmol, 1.0 equiv) and DMF (2.0 mL) were used. The reaction mixture was irritated by two 40w Blue LED lamps for 36 h. The residue was purified by flash column chromatography (10% Acetone in hexane) to afford product (89.0 mg, 99% yield, dr = 1.6/1) as colorless oil. ^{1}H NMR (400 MHz, $CDCl_3$) δ 7.70 (s, br, s, br, 1H), 7.34–7.28 (m, 1H), 7.22 (t, J = 7.6 Hz, 1H), 6.96 (dd, J = 17.2, 7.6 Hz, 1H), 5.26 (s, 0.58 H), 4.94 (s, 0.36 H), 4.29 (d, J = 4.9 Hz, 0.59H), 3.98 (s, 0.74 H), 3.76–3.67 (m, 4H), 3.49–3.28 (m, 2H), 1.93–1.71 (m, 4H), 1.56–1.55 (s, s, 9H), 1.40–1.37 (s ,s , 9H). ^{13}C NMR (100 MHz, $CDCl_3$) δ 171.8, 171.6, 154.7, 152.9, 152.3, 143.2, 142.7, 129.1, 128.8, 127.5, 125.8, 125.1, 123.1, 122.7, 116.7, 115.9, 81.5, 80.0, 64.4, 63.9, 63.1, 60.8, 58.6, 52.6, 48.7, 48.0, 47.7, 47.0, 28.5, 28.5, 28.4, 27.5, 23.2. HRMS (ESI) calcd for $[C_{24}H_{34}N_2O_6+ Na]^+$: 469.2309, found 469.2303.

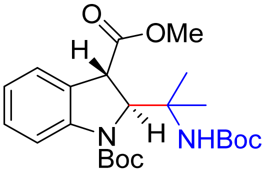

(±) *trans*-**3ad**

(±) *trans*-**3ad** was synthesized according to the general procedure **G**. 1-(*tert*-Butyl) 3-methyl 1*H*-indole-1,3-dicarboxylate (55.0 mg, 0.2 mmol, 1.0 equiv), 4CzIPN (8.0 mg, 0.01 mmol, 5 mol%), 2-((*tert*-butoxycarbonyl)amino)-2-methylpropanoic acid (81.3 mg, 0.4 mmol, 2.0 equiv), Cs_2CO_3 (65.0 mg, 0.2 mmol, 1.0 equiv) and DMF (2.0 mL) were used. The reaction mixture was irritated by two 40w Blue LED lamps for 36 h. The residue was purified by flash column chromatography (10-30% EtOAc in hexane) to afford product (96.0 mg, 99% yield) as white solid. ^{1}H NMR (400 MHz, $CDCl_3$) δ 7.56 (s, br, 1H), 7.31 (d, J = 7.4 Hz, 1H), 7.24 (t, J = 7.9 Hz, 1H), 7.00 (t, J = 7.5 Hz, 1H), 5.66 (s, 1H), 5.03 (s, br, 1H), 4.00 (s, 1H), 3.69 (s, 3H), 1.59 (s, 9H), 1.39 (s, 9H), 1.23 (s, 6H). ^{13}C NMR (100 MHz, $CDCl_3$) δ 171.6, 154.6, 153.9, 143.4, 129.3, 128.8, 125.1, 123.6, 117.5, 82.5, 78.8, 68.8, 57.2, 52.7, 47.9, 28.5, 28.3, 23.7, 21.3. HRMS (ESI) calcd for $[C_{23}H_{34}N_2O_6+ Na]^+$: 457.2309, found: 457.2314.

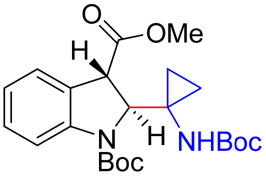

(±) *trans*-**3ae**

(±) *trans*-**3ae** was synthesized according to the general procedure **G**. 1-(*tert*-Butyl) 3-methyl 1*H*-indole-1,3-dicarboxylate (55.0 mg, 0.2 mmol, 1.0 equiv), 4CzIPN (10.0 mg, 0.015 mmol, 7.5 mol%), 1-((*tert*-butoxycarbonyl)amino)cyclopropane-1-carboxylic acid (80.4 mg, 0.4 mmol, 2.0

equiv), Cs_2CO_3 (65.0 mg, 0.2 mmol, 1.0 equiv) and DMF (2.0 mL) were used. The reaction mixture was irritated by two 40w Blue LED lamps for 36 h. The residue was purified by flash column chromatography (10-20% EtOAc in hexane) to afford product (53.0 mg, 62% yield) as colorless oil. ^{1}H NMR (400 MHz, $CDCl_3$) δ 7.64 (s, br 1H), 7.32 (d, *J* = 7.4 Hz, 1H), 7.19 (t, *J* = 7.7 Hz, 1H), 6.94 (d, *J* = 7.4 Hz, 1H), 4.76 (s, 2H), 4.37 (s, 1H), 3.67 (s, 3H), 1.57 (s, 9H), 1.15 (s, 10H), 1.04 (s, 1H), 0.96–0.79 (m, 2H). ^{13}C NMR (100 MHz, $CDCl_3$) δ 172.1, 155.5, 152.6, 142.7, 128.5, 128.1, 126.0, 122.7, 115.6, 81.6, 79.3, 68.2, 52.5, 50.4, 36.7, 28.6, 28.2, 16.7, 11.5. HRMS (ESI) calcd for $[C_{23}H_{32}N_2O_6+ Na]^+$: 455.2153, found: 455.2156.

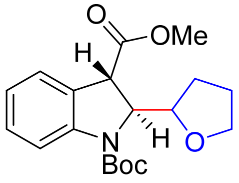

(±) *trans*-**3af**

(±) *trans*-**3af** was synthesized according to the general procedure **G**. 1-(*tert*-Butyl) 3-methyl 1*H*-indole-1,3-dicarboxylate (55.0 mg, 0.2 mmol, 1.0 equiv), 4CzIPN (8.0 mg, 0.01 mmol, 5 mol%), tetrahydrofuran-2-carboxylic acid (41.8 mg, 0.26 mmol, 1.3 equiv), Cs_2CO_3 (65.0 mg, 0.2 mmol, 1.0 equiv) and DMF (2.0 mL) were used. The reaction mixture was irritated by 5w Blue LED strip for 36 h. The residue was purified by flash column chromatography (20% Et_2O in hexane) to afford product (60.0 mg, 87% yield, dr = 2.0/1 by ^{1}H NMR) as colorless oil. ^{1}H NMR (400 MHz, $CDCl_3$) δ 7.62 (s, 1H), 7.34 (d, *J* = 7.4 Hz, 1H), 7.20 (d, *J* = 7.9 Hz, 1H), 6.97 (t, *J* = 7.4 Hz, 1H), 5.08 (s, 0.33 H), 4.86 (s, 0.66H), 4.28 (q, *J* = 6.0 Hz, 0.43H), 4.16 – 4.04 (m, 1.39H), 3.90– 3.70 (m, 6H), 2.02-1.75 (m, 4H), 1.56 (s, 9H). ^{13}C NMR (100 MHz, $CDCl_3$) δ 172.1, 171.7, 152.5, 142.4, 128.9, 128.7, 128.7, 128.0, 125.6, 125.6, 125.2, 125.1122.8, 116.2, 116.1, 116.0, 116.0, 81.5, 81.4, 79.4, 79.3, 78.8, 78.7, 68.8, 68.8, 68.7, 68.7, 68.6, 64.6, 64.5, 63.4, 63.2, 52.8, 52.8, 52.7, 52.7, 52.6, 52.5, 52.4, 52.4, 52.4, 47.2, 47.1, 46.9, 28.5, 28.5, 28.5, 28.4, 26.3, 25.7, 25.6. HRMS (ESI) calcd for $[C_{19}H_{25}NO_5+ H]^+$: 348.1805, found: 348.1810.

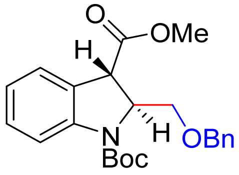

(±) *trans*-**3ag**

(±) *trans*-**3ag** was synthesized according to the general procedure **G**. 1-(*tert*-Butyl) 3-methyl 1*H*-indole-1,3-dicarboxylate (55.0 mg, 0.2 mmol, 1.0 equiv), 4CzIPN (8.0 mg, 0.01 mmol, 5 mol%), 2-(benzyloxy)acetic acid (43.6 mg, 0.26 mmol, 1.3 equiv), Cs_2CO_3 (65.0 mg, 0.2 mmol, 1.0 equiv) and DMF (2.0 mL) were used. The reaction mixture was irritated by 5w Blue LED strip for 36 h. The residue was purified by flash column chromatography (2% EtOAc in hexane) to afford product (55 mg, 81% yield) as light yellow oil. ^{1}H NMR (400 MHz, $CDCl_3$) δ 7.79 (s, br, 1H), 7.3 (d, *J* = 6.8 Hz, 1H), 7.31-7.22 (m, 6H), 7.00-6.97 (m, 1H), 4.99 (s, 1H), 4.55– 4.49 (m, 2H), 4.16 (s, 1H), 3.76-3.72 (m, 4H), 3.52 (s, 1H), 1.53 (s, 9H). ^{13}C NMR (100 MHz, $CDCl_3$) δ 171.9, 151.9, 142.4, 138.1, 128.9, 128.5, 127.8, 127.6, 125.8, 122.7, 115.54, 81.4, 77.5, 77.2, 76.8, 73.2, 69.8, 61.2, 52.6, 48.8, 28.5. HRMS (ESI) calcd for $[C_{23}H_{27}NO_5+ Na]^+$: 420.1781, found: 420.1781.

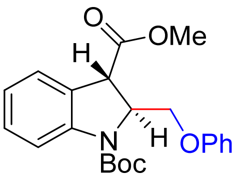

(±) *trans*-**3ah**

(±) *trans*-**3ah** was synthesized according to the general procedure **G**. 1-(*tert*-Butyl) 3-methyl 1*H*-indole-1,3-dicarboxylate (55.0 mg, 0.2 mmol, 1.0 equiv), 4CzIPN (10.0 mg, 0.015 mmol, 7.5 mol%), 2-phenoxyacetic acid (45.6 mg, 0.3 mmol, 1.5 equiv), Cs_2CO_3 (65.0 mg, 0.2 mmol, 1.0 equiv) and DMF (2.0 mL) were used. The reaction mixture was irritated by two 40w Blue LED lamps for 36 h. The residue was purified by flash column chromatography (0-20% EtOAc in hexane) to afford product (49.8 mg, 65% yield) as colorless oil. ^{1}H NMR (400 MHz, $CDCl_3$) δ 7.77 (s, br, 1H), 7.39 (d, J = 7.4 Hz, 1H), 7.26 (t, J = 7.3 Hz, 4H), 7.02 (t, J = 7.4 Hz, 1H), 6.95 (t, J = 7.1 Hz, 1H), 6.87 (d, J = 8.1 Hz, 2H), 5.19 (s, 1H), 4.30 (d, J = 7.0 Hz, 1H), 4.22 (s, 1H), 4.01 (s, br, 1H), 3.75 (s, 3H), 1.59 (s, 9H). ^{13}C NMR (100 MHz, $CDCl_3$) δ 171.7, 158.6, 152.0, 142.3, 129.6, 129.1, 125.9, 123.0, 121.3, 115., 114.8, 82.0, 67.5, 60.8, 52.8, 48.7, 28.57. HRMS (ESI) calcd for $[C_{22}H_{25}NO_5 + Na]^+$: 406.1625, found: 406.1629.

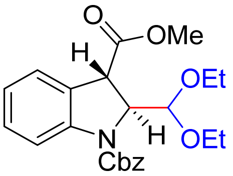

(±) *trans*-**3ai**

(±) *trans*-**3ai** was synthesized according to the general procedure **G**. 1-Benzyl 3-methyl 1*H*-indole-1,3-dicarboxylate (61.8 mg, 0.2 mmol, 1.0 equiv), 4CzIPN (8.0 mg, 0.01 mmol, 5 mol%), 2,2-diethoxyacetic acid (59.2 mg, 0.4 mmol, 2.0 equiv), Cs_2CO_3 (65.0 mg, 0.2 mmol, 1.0 equiv) and DMF (2.0 mL) were used. The reaction mixture was irritated by 5w Blue LED strip for 36 h. The residue was purified by flash column chromatography (25% Acetone in hexane) to afford product (75.1 mg, 91% yield) as colorless oil. ^{1}H NMR (400 MHz, $CDCl_3$) δ 7.88 (s, br, 1H), 7.4 (d, J = 7.3 Hz, 2H), 7.40–7.32 (m, 4H), 7.21 (s, br, 1H), 6.98 (t, J = 7.4 Hz, 1H), 5.40-5.21 (s, s, 2H), 5.03 (s, 1H), 4.74 (s, 1H), 4.39 (d, J = 1.7 Hz, 1H), 3.71 (d, J = 2.0 Hz, 3H), 3.56-3.27 (s, s, 4H), 1.16 (s, 3H), 0.86 (t, J = 6.6 Hz, 3H). ^{13}C NMR (100 MHz, $CDCl_3$) δ 171.8, 142.3, 136.0, 128.6, 128.5, 128.3, 128.3, 125.0, 122.9, 115.1, 101.6, 67.4, 65.4, 63.9, 63.7, 52.5, 52.4, 45.8, 15.2, 15.1. HRMS (ESI) calcd for $[C_{23}H_{27}NO_6 + Na]^+$: 436.1731, found: 436.1734.

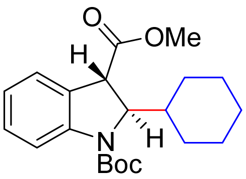

(±) *trans*-**3aj**

(±) *trans*-**3aj** was synthesized according to the general procedure **G**. 1-(*tert*-Butyl) 3-methyl 1*H*-indole-1,3-dicarboxylate (55.0 mg, 0.2 mmol, 1.0 equiv), 4CzIPN (8.0 mg, 0.01 mmol, 5 mol%), cyclohexanecarboxylic acid (51.2 mg, 4.0 mmol, 2.0 equiv), Cs_2CO_3 (65.0 mg, 0.2 mmol, 1.0

equiv) and DMF (2.0 mL) were used. The reaction mixture was irritated by two 40w Blue LED lamps for 36 h. The residue was purified by flash column chromatography (0-10% EtOAc in hexane) to afford product (64.0 mg, 89% yield) as colorless oil. ^{1}H NMR (400 MHz, $CDCl_3$) δ 7.68 (s, br, 1H), 7.29 (d, *J* = 7.4 Hz, 1H), 7.21 (t, *J* = 7.7 Hz, 1H), 6.95 (t, *J* = 7.5 Hz, 1H), 4.68 (s, 1H), 3.85 (s, 1H), 3.70 (s, 3H), 1.96 (s, 1H), 1.79–1.72 (m, 1H), 1.65 (t, *J* = 11.3 Hz, 3H), 1.57 (s, 9H), 1.48 (d, *J* = 12.5 Hz, 1H), 1.31–0.98 (m, 4H), 0.87–0.71 (m, 1H). ^{13}C NMR (100 MHz, $CDCl_3$) δ 172.3, 152.5, 143.1, 128.9, 128.2, 125.2, 122.6, 115.7, 81.2, 66.5, 52.6, 47.5, 41.5, 28.6, 28.5, 26.5, 26.5, 26.3, 26.1. HRMS (ESI) calcd for $[C_{21}H_{29}NO_4 + H]^+$: 360.2169, found: 360.2174.

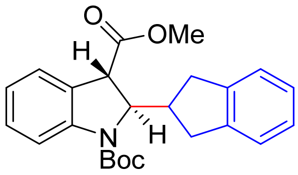

(±) *trans*-**3ak**

(±) *trans*-**3ak** was synthesized according to the general procedure **G**. 1-(*tert*-Butyl) 3-methyl 1*H*-indole-1,3-dicarboxylate (55.0 mg, 0.2 mmol, 1.0 equiv), 4CzIPN (8.0 mg, 0.01 mmol, 5 mol%), 2,3-dihydro-1*H*-indene-2-carboxylic acid (64.8 mg, 0.4 mmol, 2.0 equiv), Cs_2CO_3 (65.0 mg, 0.2 mmol, 1.0 equiv) and DMF (2.0 mL) were used. The reaction mixture was irritated by two 40w Blue LED lamps for 36 h. The residue was purified by preparative TLC (2% EtOAc in hexane) to afford product (55 mg, 70% yield) as light yellow oil. ^{1}H NMR (400 MHz, $CDCl_3$) δ 7.73 (s, 1H), 7.30 (t, *J* = 7.2 Hz, 2H), 7.19– 7.12 (m, 4H), 7.00 (t, *J* = 7.2 Hz, 1H), 5.05 (s, 1H), 3.85 (s, 1H), 3.73 (s, 3H), 3.10-3.16 (m, 1H), 3.03 (dd, *J* = 15.8, 8.4 Hz, 1H), 2.93–2.84 (m, 2H), 2.64 (dd, *J* = 15.6, 8.8 Hz, 1H), 1.59 (s, 9H). ^{13}C NMR (100 MHz, CDC_3) δ 172.0, 152.5, 142.9, 142.5, 129.1, 127.7, 126.5, 125.6, 124.6, 122.9, 116.2, 81.5, 77.5, 77.2, 76.8, 64.6, 52.7, 48.6, 42.7, 35.0, 33.9, 28.5. HRMS (ESI) calcd for $[C_{24}H_{27}NO_4 + Na]^+$: 416.1832, found: 416.1837.

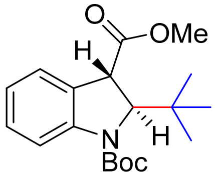

(±) *trans*-**3al**

(±) *trans*-**3al** was synthesized according to the general procedure **G**. 1-(*tert*-Butyl) 3-methyl 1*H*-indole-1,3-dicarboxylate (55.0 mg, 0.2 mmol, 1.0 equiv), 4CzIPN (8.0 mg, 0.01 mmol, 5 mol%), pivalic acid (26.5 mg, 0.26 mmol, 1.3 equiv), Cs_2CO_3 (65.0 mg, 0.2 mmol, 1.0 equiv) and DMF (2.0 mL) were used. The reaction mixture was irritated by two 40w Blue LED lamps for 36 h. The residue was purified by preparative TLC (3% EtOAc in hexane) to afford product (49.0 mg, 74% yield) as light yellow oil. ^{1}H NMR (400 MHz, $CDCl_3$) δ 7.57 (s, 1H), 7.29– 7.20 (m, 2H), 6.98 (t, *J* = 7.4 Hz, 1H), 4.65 (s, 1H), 3.80 (s, 1H), 3.67 (s, 3H), 1.55 (s, 9H), 0.85 (s, 9H). ^{13}C NMR (100 MHz, CDC_3) δ 172.2, 153.8, 143.9, 129.8, 128.7, 124.8, 123.1, 117.3, 81.2, 70.1, 52.6, 48.1, 36.4, 28.4, 26.12. HRMS (ESI) calcd for $[C_{19}H_{27}NO_4 + Na]^+$: 356.1832, found: 356.1825.

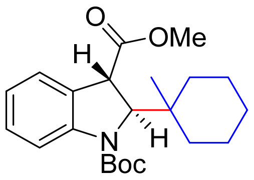

(±) *trans*-**3am**

(±) *trans*-**3am** was synthesized according to the general procedure **G**. 1-(*tert*-Butyl) 3-methyl 1*H*-indole-1,3-dicarboxylate (55.0 mg, 0.2 mmol, 1.0 equiv), 4CzIPN (8.0 mg, 0.01 mmol, 5 mol%), 1-methylcyclohexane-1-carboxylic acid (36.9 mg, 0.26 mmol, 1.3 equiv), Cs_2CO_3 (65.0 mg, 0.2 mmol, 1.0 equiv) and DMF (2.0 mL) were used. The reaction mixture was irritated by two 40w Blue LED lamps for 36 h. The residue was purified by flash column chromatography (10% Et_2O in hexane) to afford product (44.0 mg, 75% yield) as colorless oil. ^{1}H NMR (500 MHz, $CDCl_3$) δ 7.54 (s, 1H), 7.27 (dt, *J* = 7.5, 0.5 Hz, 1H), 7.24–7.20 (m, 1H), 6.98 (td, *J* = 7.5, 1.1 Hz, 1H), 4.65 (s, 1H), 3.86 (s, 1H), 3.67 (s, 3H), 1.55 (s, 9H), 1.54–1.05 (m, 10H), 0.76 (s, 3H). ^{13}C NMR (126 MHz, $CDCl_3$) δ 172.6, 154.2, 144.5, 130.3, 128.7, 124.7, 123.2, 117.6, 81.2, 71.3, 52.5, 47.2, 39.14, 33.73, 33.2, 28.4, 26.2, 21.6, 18.4. HRMS (ESI) calcd for $[C_{22}H_{31}NO_4+ Na]^+$: 396.2145, found 396.2136.

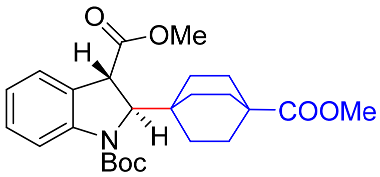

(±) *trans*-**3an**

(±) *trans*-**3an** was synthesized according to the general procedure **G**. 1-(*tert*-Butyl) 3-methyl 1*H*-indole-1,3-dicarboxylate (55.0 mg, 0.2 mmol, 1.0 equiv), 4CzIPN (10.0 mg, 0.01 mmol, 7.5 mol%), 4-(methoxycarbonyl)bicyclo[2.2.2]octane-1-carboxylic acid (84.8 mg, 0.4 mmol, 2.0 equiv), Cs_2CO_3 (65.0 mg, 0.2 mmol, 1.0 equiv) and DMF (2.0 mL) were used. The reaction mixture was irritated by two 40w Blue LED lamps for 36 h. The residue was purified by flash column chromatography (10-30% EtOAc in hexane) to afford product (55 mg, 78% yield) as white foam solid. ^{1}H NMR (400 MHz, $CDCl_3$) δ 7.53 (s, br, 1H), 7.29–7.20 (m, 2H), 6.99 (t, *J* = 7.4 Hz, 1H), 4.60 (s, 1H), 3.83 (s, 1H), 3.66 (s, 3H), 3.60 (s, 3H), 1.71 (t, *J* = 7.9 Hz, 6H), 1.54 (s, 9H), 1.490–1.42 (m, 3H), 1.39–1.33 (m, 3H). ^{13}C NMR (100 MHz, $CDCl_3$) δ 178.2, 172.0, 153.7, 143.7, 129.5, 128.7, 124.8, 123.3, 117.4, 81.4, 69.0, 52.6, 51.7, 47.4, 38.7, 36.9, 28.4, 28.1, 26.5. HRMS (ESI) calcd for $[C_{25}H_{33}NO_6+ Na]^+$: 466.2200, found: 466.2199.

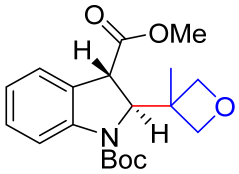

(±) *trans*-**3ao**

(±) *trans*-**3ao** was synthesized according to the general procedure **G**. 1-(*tert*-Butyl) 3-methyl 1*H*-indole-1,3-dicarboxylate (55.0 mg, 0.2 mmol, 1.0 equiv), 4CzIPN (10.0 mg, 0.015 mmol, 7.5 mol%), 3-methyloxetane-3-carboxylic acid (46.4 mg, 0.4 mmol, 2.0 equiv), Cs_2CO_3 (65.0 mg, 0.2

mmol, 1.0 equiv) and DMF (2.0 mL) were used. The reaction mixture was irritated by two 40w Blue LED lamps for 36 h. The residue was purified by flash column chromatography (10% acetone in hexane) to afford product (43.0 mg, 62% yield) as white foam solid. ^{1}H NMR (400 MHz, $CDCl_3$) δ 7.63 (s, br, 1H), 7.29– 7.23 (m, 2H), 6.98 (t, J = 7.5 Hz, 1H), 5.06 (d, J = 2.7 Hz, 1H), 4.82 (d, J = 6.0 Hz, 1H), 4.65 (d, J = 5.9 Hz, 1H), 4.19 (d, J = 5.9 Hz, 1H), 4.13 (d, J = 6.0 Hz, 1H), 3.74 (s, 3H), 3.44 (d, J = 2.4 Hz, 1H), 1.57 (s, 9H), 1.26 (s, 3H). ^{13}C NMR (100 MHz, $CDCl_3$) δ 171.6, 152.9, 143.1, 129.3, 127.4, 125.2, 123.1, 116.4, 82.2, 81.3, 78.78, 66.2, 53.0, 52.9, 48.5, 44.4, 28.5, 20.0. HRMS (ESI) calcd for $[C_{19}H_{25}NO_5 + Na]^+$: 370.1625, found: 370.1627.

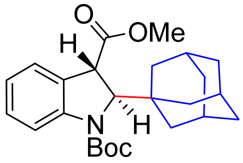

(±) *trans*-**3ap**

(±) *trans*-**3ap** was synthesized according to the general procedure **G**. 1-(*tert*-Butyl) 3-methyl 1*H*-indole-1,3-dicarboxylate (55.0 mg, 0.2 mmol, 1.0 equiv), 4CzIPN (10.0 mg, 0.015 mmol, 7.5 mol%), 1-adamantanecarboxylic acid (74.0 mg, 0.4 mmol, 2.0 equiv), Cs_2CO_3 (65.0 mg, 0.2 mmol, 1.0 equiv) and DMF (2.0 mL) were used. The reaction mixture was irritated by two 40w Blue LED lamps for 36 h. The residue was purified by flash column chromatography (10% acetone in hexane) to afford product (77.0 mg, 94% yield) as white foam solid. ^{1}H NMR (400 MHz, $CDCl_3$) δ 7.56 (s, br, 1H), 7.27 (d, J = 7.6 Hz, 1H), 7.22 (t, J = 7.7 Hz, 1H), 6.98 (t, J = 7.5 Hz, 1H), 4.48 (s, 1H), 3.89 (s, 1H), 3.66 (s, 3H), 1.92 (s, 3H), 1.65 (d, J = 12.0 Hz, 3H), 1.61–1.46 (m, 15H), 1.41 (s, s, 3H). ^{13}C NMR (100 MHz, $CDCl_3$) δ 172.4, 153.9, 144.1, 130.0, 128.6, 124.7, 123.1, 117.3, 81.1, 70.9, 52.5, 46.4, 38.2, 38.0, 37.0, 28.5, 28.1. HRMS (ESI) calcd for $[C_{25}H_{33}NO_4 + Na]^+$: 434.2302, found: 434.2307.

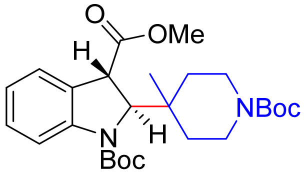

(±) *trans*-**3aq**

(±) *trans*-**3aq** was synthesized according to the general procedure **G**. 1-(*tert*-Butyl) 3-methyl 1*H*-indole-1,3-dicarboxylate (55.0 mg, 0.2 mmol, 1.0 equiv), 4CzIPN (8.0 mg, 0.01 mmol, 5 mol%), 1-(*tert*-butoxycarbonyl)-4-methylpiperidine-4-carboxylic acid (97.3 mg, 0.4 mmol, 2.0 equiv), Cs_2CO_3 (65.0 mg, 0.2 mmol, 1.0 equiv) and DMF (2.0 mL) were used. The reaction mixture was irritated by two 40w Blue LED lamps for 36 h. The residue was purified by flash column chromatography (25% acetone in hexane) to afford product (60.0 mg, 62% yield) as colorless oil. ^{1}H NMR (400 MHz, cdcl3) δ 7.50 (s, br, 1H), 7.30–7.19 (m, 2H), 6.98 (t, J = 7.4 Hz, 1H), 4.69 (s, 1H), 3.85–3.82 (s, s, 3H), 3.67 (s, 3H), 2.94–2.88 (m, 2H), 1.54 (s, 9H), 1.42 (s, 9H), 0.81 (s, 3H). ^{13}C NMR (100 MHz, $CDCl_3$) δ 171.9, 155.0, 153.9, 144.0, 129.6, 128.8, 124.8, 123.4, 117.6, 81.6, 79.4, 70.2, 52.7, 47.24, 39.7, 37.8, 33.1, 32.6, 28.5,28.4, 17.6. HRMS (ESI) calcd for $[C_{26}H_{38}N_2O_6 + Na]^+$: 497.2622, found: 497.2627.

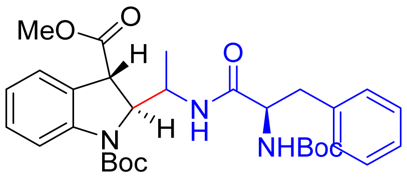

(±) *trans*-**2ar**

(±) *trans*-**2ar** was synthesized according to the general procedure **G**. 1-(*tert*-Butyl) 3-methyl 1*H*-indole-1,3-dicarboxylate (55.0 mg, 0.2 mmol, 1.0 equiv), 4CzIPN (8.0 mg, 0.01 mmol, 5 mol%), Boc-L-Phe-L-Ala-OH (67.2 mg, 0.26 mmol, 1.3 equiv), Cs_2CO_3 (65.0 mg, 0.2 mmol, 1.0 equiv) and DMF (2.0 mL) were used. The reaction mixture was irritated by 5w Blue LED strip for 36 h. The residue was purified by flash column chromatography (15% Acetone in hexane) to afford product (76.0 mg, 67% yield, dr = 1.6/1.0/1.0/1.0 by ^{1}H NMR) as light yellow oil. ^{1}H NMR (400 MHz, $CDCl_3$) δ 7.56 (br, 1H), 7.32-7.17 (m, 7H), 6.98-6.95 (m, 1H), 5.10 (d, 5.6 Hz, 0.5H), 4.92 (dd, 6.4 Hz, 0.3H), 4.83 (d, 4.8 Hz, 0.4H), 4.74 (br, 0.4H), 4.2 (br, 2H), 3.69 (m, 3H), 2.98-2.77 (m, 2H), 2.16-2.15 (m, 1H), 1.58-1.52 (m, 9H), 1.39-1.36 (m, 9H), 1.11-0.87 (m, 3H). ^{13}C NMR (100 MHz, $CDCl_3$) δ 210.8, 207.0, 171.3, 171.2, 171.1, 170.7, 155.4, 153.6, 142.3, 136.9, 136.9, 126.8, 136.7, 129.5, 129.4, 129.1, 129.1, 128.8, 128.6, 128.5, 126.9, 126.8, 126.7, 125.5, 123.3, 123.2, 122.9, 116.7, 116.4, 115.9, 82.4, 82.3, 79.9, 79.8, 65.4, 64.8, 56.1, 55.7, 55.5, 53.9, 52.8, 49.1, 48.8, 38.8, 38.4, 38.2, 28.4, 28.3, 17.1, 14.6. HRMS (ESI) calcd for $[C_{31}H_{41}N_3O_7+ Na]^+$: 590.2837, found: 590.2838.

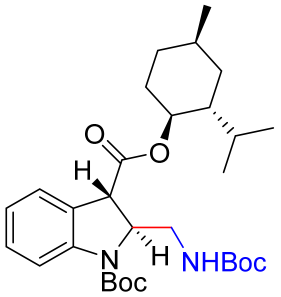

(±) *trans*-**3as**

(±) *trans*-**3as** was synthesized according to the general procedure **G**. 1-(*tert*-butyl) 3-((1*S*,2*R*,4*R*)-2-isopropyl-4-methylcyclohexyl) 1*H*-indole-1,3-dicarboxylate **1as** (79.9 mg, 0.2 mmol, 1.0 equiv), 4CzIPN (8.0 mg, 0.01 mmol, 5 mol%), Boc-glycine (45.5 mg, 0.26 mmol, 1.3 equiv), Cs_2CO_3 (65.0 mg, 0.2 mmol, 1.0 equiv) and DMF (2.0 mL) were used. The reaction mixture was irritated by 5w Blue LED strip for 36 h. The residue was purified by flash column chromatography (10% acetone in hexane) to afford product (117 mg, 99% yield, dr = 1.1/1) as white foam solid. ^{1}H NMR (400 MHz, $CDCl_3$) δ 7.67 (s, br, 1H), 7.31 (t, J = 6.6 Hz, 1H), 7.21 (t, J = 7.6 Hz, 1H), 6.96 (dd, J = 14.0, 7.2 Hz, 1H), 4.84 (d, J = 4.2 Hz, 2H), 4.67–4.61 (m, 1H), 3.93 (d, J = 9.4 Hz, 1H), 3.43 (d, J = 5.4 Hz, 2H), 1.97–1.63 (m, 4H), 1.57 (s, 9H), 1.37 (s, 11H), 1.03–0.93 (m, 2H), 0.89–0.82 (m, 7H), 0.67 (d, J = 6.9 Hz, 1.59H), 0.61 (d, J = 6.8 Hz, 1.44H). ^{13}C NMR (100 MHz, $CDCl_3$) δ 170.7, 170.6, 156.2, 142.0, 129.0, 127.5, 125.8, 125.6, 122.9, 122.7, 120.3, 119.3, 115.8, 115.7, 81.8, 79.5, 75.6, 75.5, 62.0, 61.8, 49.9, 49.7, 47.2, 47.1, 43.8, 43.8, 40.8, 40.7, 34.3, 34.2, 31.5, 31.4, 28.5, 28.4, 28.4, 26.3, 25.9, 23.5, 23.1, 22.1, 22.0, 21.0, 20.8, 16.4, 16.0. HRMS (ESI) calcd for $[C_{30}H_{46}N_2O_6+ Na]^+$: 553.3248, found: 553.3252.

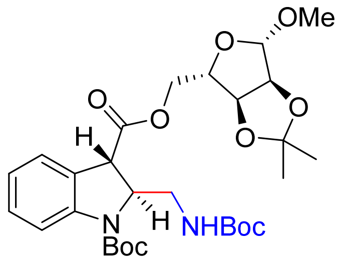

(±) *trans*-**3at**

(±) *trans*-**3at** was synthesized according to the general procedure **G**. 1-(*tert*-butyl) 3-(((3a*S*,4*S*,6*S*,6a*S*)-6-methoxy-2,2-dimethyltetrahydrofuro[3,4-*d*][1,3]dioxol-4-yl)methyl) 1*H*-indole-1,3-dicarboxylate **1at** (89.5 mg, 0.2 mmol, 1.0 equiv), 4CzIPN (8.0 mg, 0.01 mmol, 5 mol%), Boc-glycine (45.5 mg, 0.26 mmol, 1.3 equiv), Cs_2CO_3 (65.0 mg, 0.2 mmol, 1.0 equiv) and DMF (2.0 mL) were used. The reaction mixture was irritated by 5w Blue LED strip for 36 h. The residue was purified by flash column chromatography (hexane/DCM/EtOAc = 3/1/1) to afford product (81.0 mg, 70% yield, dr = 1.0/1) as white foam solid. ^{1}H NMR (400 MHz, cdcl$_3$) δ 7.66 (s, br, 1H), 7.38 (t, *J* = 6.6 Hz, 1H), 7.23 (t, *J* = 6.2 Hz, 1H), 6.98 (td, *J* = 7.4, 2.3 Hz, 1H), 4.95 (d, *J* = 6.8 Hz, 1H), 4.88 (s, 2H), 4.66 (d, *J* = 5.8 Hz, 0.52H), 4.60 (d, *J* = 5.9 Hz, 0.52H), 4.55 (d, *J* = 5.9 Hz, 0.53H), 4.48 (d, *J* = 5.9 Hz, 0.49H), 4.38 (dd, *J* = 11.7, 6.0 Hz, 1H), 4.21 (dd, *J* = 11.3, 6.4 Hz, 1H), 4.08 (ddd, *J* = 11.2, 6.5, 1.8 Hz, 1H), 4.03 (s, 1H), 3.45-3.40 (m, 2H), 3.27 (s, 1.47H), 3.20 (s, 1.45H), 2.62 (s, 0.52H), 2.17 (s, 0.75H), 1.57 (s, 9H), 1.47 (d, *J* = 4.3 Hz, 3H), 1.37 (s, 9H), 1.32 (s, 1.68H), 1.29 (s, 1.60H). ^{13}C NMR (100 MHz, $CDCl_3$) δ 170.7, 170.6, 156.3, 152.2, 142.1, 129.2, 127.0, 126.0, 123.0, 115.8, 115.8, 112.7, 112.6, 109.7, 85.3, 85.3, 84.3, 84.2, 82.0, 81.8, 79.7, 69.6, 65.9, 65.7, 61.8, 55.1, 55.1, 53.9, 49.3, 43.7, 31.9, 29.4, 28.5, 28.4, 26.6, 26.5, 25.1, 25.0. HRMS (ESI) calcd for $[C_{29}H_{42}N_2O_{10}+ Na]^+$: 601.2732, found: 601.2736.

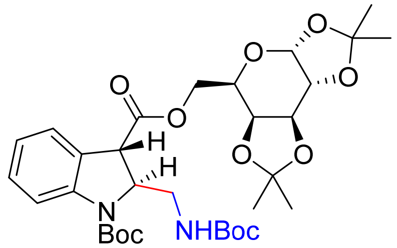

(±) *trans*-**3au**

(±) *trans*-**3au** was synthesized according to the general procedure **G**. 1-(*tert*-butyl) 3-(((3a*R*,5*R*,5a*S*,8a*S*,8b*R*)-2,2,7,7-tetramethyltetrahydro-5*H*-bis([1,3]dioxolo)[4,5-*b*:4',5'-d]pyran-5-yl)methyl) 1*H*-indole-1,3-dicarboxylate **1au** (100.6 mg, 0.2 mmol, 1.0 equiv), 4CzIPN (8.0 mg, 0.01 mmol, 5 mol%), Boc-glycine (45.5 mg, 0.26 mmol, 1.3 equiv), Cs_2CO_3 (65.0 mg, 0.2 mmol, 1.0 equiv) and DMF (2.0 mL) were used. The reaction mixture was irritated by 5w Blue LED strip for 36 h. The residue was purified by flash column chromatography (25% EtOAc in hexane) to afford product (103 mg, 81% yield, dr = 1.2/1) as white foam solid. ^{1}H NMR (400 MHz, $CDCl_3$) δ 7.65 (s, 1H), 7.37 (t, *J* = 6.4 Hz, 1H), 7.19 (t, *J* = 7.7 Hz, 1H), 6.94 (dd, *J* = 12.9, 7.0 Hz, 1H), 5.52 (d, *J* = 4.9 Hz, 0.51H), 5.48 (d, *J* = 4.9 Hz, 0.44H), 4.86–4.83 (m, 2H), 4.57 (dd, *J* = 7.8, 2.3 Hz, 1H), 4.33–4.14 (m, 4H), 4.01–3.93 (m, 2H), 3.44-3.43 (m, 2H), 1.55 (s, 9H), 1.46-1.35 (m, 15H), 1.30 (s, 6H). ^{13}C NMR (100 MHz, $CDCl_3$) δ 170.9, 156.3, 156.2, 152.2, 142.0, 128.9, 128.9, 126.9, 126.1, 126.1, 113.0, 122.9, 115.6, 109.7, 109.7, 108.8, 96.3, 96.3, 81.8, 79.5, 71.0, 70.9,

70.7, 70.7, 70.5, 70.4, 66.0, 65.7, 64.4, 64.2, 61.7, 61.6, 49.2, 43.6, 28.4, 28.4, 28.3, 26.1, 26.0, 26.0, 25.0, 25.0, 24.5. HRMS (ESI) calcd for [$C_{32}H_{46}N_2O_{11}$+ Na]$^+$: 657.2994, found: 657.3000.

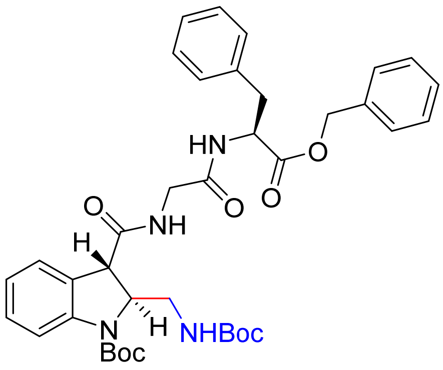

(±) *trans*-**3av**

(±) *trans*-**3av** was synthesized according to the general procedure **G**. *tert*-butyl (*S*)-3-((2-((1-(benzyloxy)-1-oxo-3-phenylpropan-2-yl)amino)-2-oxoethyl)carbamoyl)-1*H*-indole-1-carboxylate **1av** (111 mg, 0.2 mmol, 1.0 equiv), 4CzIPN (8.0 mg, 0.01 mmol, 5 mol%), Boc-glycine (45.5 mg, 0.26 mmol, 1.3 equiv), Cs_2CO_3 (65.0 mg, 0.2 mmol, 1.0 equiv) and DMF (2.0 mL) were used. The reaction mixture was irritated by two 40w Blue lamps for 36 h. The residue was purified by flash column chromatography (25% EtOAc in hexane) to afford product (114 mg, 83% yield, dr = 1.1/1) as white foam solid. ^{1}H NMR (400 MHz, $CDCl_3$) δ 7.71 (s, br, 1H), 7.34 (s, 3H), 7.27–7.14 (m, 7H), 6.99 (d, *J* = 12.7 Hz, 3H), 6.73 (s, br, 2H), 5.15–5.06 (m, 3H), 4.89-4.84 (m, 1H), 4.69 (s, 1H), 3.96 (dd, *J* = 16.3, 4.0 Hz, 1H), 3.89 (s, 1H), 3.79–3.71 (m, 1H), 3.53-3.44 (m, 2H), 3.12–2.99 (m, 2H), 1.56 (s, 9H), 1.38 (s, 9H). ^{13}C NMR (100 MHz, $CDCl_3$) δ 171.6, 171.6, 171.2, 168.2, 168.1, 156.5, 155.9, 142.6, 135.6, 135.5, 135.1, 129.3, 129.3, 128.7, 128.7, 128.6, 128.6, 127.2, 127.2, 125.3, 125.2, 123.3, 116.1, 82.0, 79.6, 67.5, 67.4, 63.4, 63.3, 53.5, 53.3, 50.7, 43.8, 43.2, 37.9, 37.8, 28.4, 28.4. HRMS (ESI) calcd for [$C_{38}H_{46}N_4O_8$+ Na]$^+$: 709.3208, found: 709.3220.

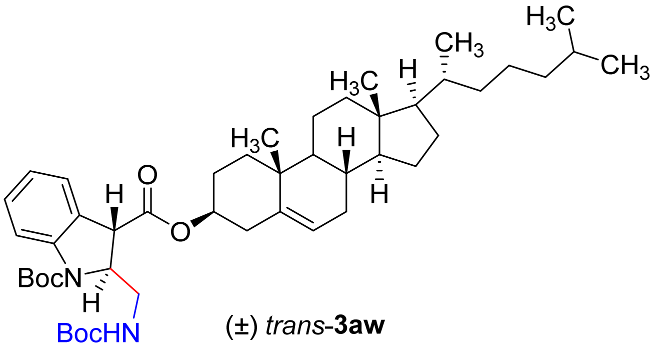

(±) *trans*-**3aw**

(±) *trans*-**3aw** was synthesized according to the general procedure **G**. *tert*-butyl (*S*)-3-((2-((1-(benzyloxy)-1-oxo-3-phenylpropan-2-yl)amino)-2-oxoethyl)carbamoyl)-1*H*-indole-1-carboxylate **1aw** (111 mg, 0.2 mmol, 1.0 equiv), 4CzIPN (8.0 mg, 0.01 mmol, 5 mol%), Boc-glycine (45.5 mg, 0.26 mmol, 1.3 equiv), Cs_2CO_3 (65.0 mg, 0.2 mmol, 1.0 equiv) and DMF (2.0 mL) were used. The reaction mixture was irritated by two 40w Blue lamps for 36 h. The residue was purified by flash column chromatography (25% EtOAc in hexane) to afford product (83.8 mg, 83% yield, dr = 1.1/1) as white foam solid. ^{1}H NMR (400 MHz, $CDCl_3$) δ 7.66 (s, br, 1H), 7.35 (d, *J* = 7.5 Hz, 1H), 7.23 (t, *J* = 7.7 Hz, 1H), 6.98 (t, *J* = 7.3 Hz, 1H), 5.38 (d, *J* = 3.7 Hz, 0.5H),

5.31 (d, J = 4.6 Hz, 0.5H), 4.88–4.82 (m, 2H), 4.66–4.56 (m, 1H), 3.95 (s, 1H), 3.50-3.45 (m, 2H), 2.36–3.21 (m, 2H), 2.02–1.74 (m, 6H), 1.58–1.32 (m, 28H), 1.15–0.97 (m, 12H), 0.94–0.85 (m, 10H), 0.67 (s, 3H). ^{13}C NMR (100 MHz, $CDCl_3$) δ 170.5, 156.3, 152.4, 142.1, 139.5, 129.0, 127.5, 125.7, 125.6, 123.0, 123.0, 123.0, 115.8, 82.0, 79.6, 75.4, 75.3, 61.8, 56.8, 56.3, 50.1, 49.6, 43.9, 42.4, 39.8, 39.6, 38.2, 38.0, 37.0, 37.0, 36.7, 36.3, 35.9, 32.0, 32.0, 32.0, 32.0, 30.5, 29.8, 28.5, 28.4, 28.1, 27.9, 27.8, 24.4, 24.0, 23.0, 22.7, 21.2, 19.5, 18.8, 15.4, 12.0. HRMS (ESI) calcd for $[C_{47}H_{72}N_2O_6 + Na]^+$: 761.5463, found: 761.5477.

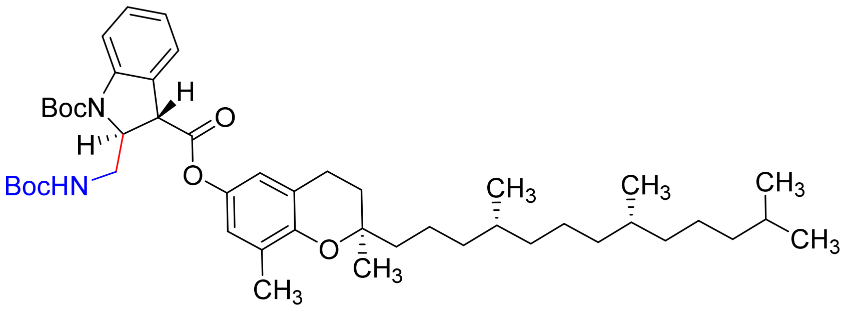

(±) *trans*-**3ax**

(±) *trans*-**3ax** was synthesized according to the general procedure **G**. 1-(*tert*-butyl) 3-((*R*)-2,8-dimethyl-2-((4*R*,8*R*)-4,8,12-trimethyltridecyl)chroman-6-yl) 1*H*-indole-1,3-dicarboxylate **1aw** (129 mg, 0.2 mmol, 1.0 equiv), 4CzIPN (8.0 mg, 0.01 mmol, 5 mol%), Boc-glycine (45.5 mg, 0.26 mmol, 1.3 equiv), Cs_2CO_3 (65.0 mg, 0.2 mmol, 1.0 equiv) and DMF (2.0 mL) were used. The reaction mixture was irritated by two 40w Blue lamps for 36 h. The residue was purified by flash column chromatography (25% EtOAc in hexane) to afford product (124 mg, 80% yield, dr = 1.1/1) as white foam solid. ^{1}H NMR (400 MHz, $CDCl_3$) δ 7.70 (s, br, 1H), 7.50 (d, J = 7.3 Hz, 1H), 7.30–7.23 (m, 1H), 7.03 (t, J = 7.3 Hz, 1H), 6.63 (s, 1H), 6.58 (m, 1H), 4.98 (s, 1H), 4.87 (s, 1H), 4.24 (s, 1H), 3.53 (s, 2H), 2.70 (s, 2H), 2.12 (s, 3H), 1.81–1.70 (m, 2H), 1.59–1.53 (m, 12H), 1.40 (s, 14H), 1.29-1.25 (m, 12H), 1.17–1.06 (m, 7H), 0.86 (t, J = 7.4 Hz, 9H). ^{13}C NMR (100 MHz, $CDCl_3$) δ 170.3, 156.4, 152.3, 150.0, 142.6, 142.2, 129.2, 127.5, 127.0, 125.9, 123.1, 121.1, 120.9, 118.9, 115.9, 115.8, 112.7, 82.0, 79.7, 76.3, 61.9, 49.5, 43.8, 40.2, 39.5, 37.5, 37.4, 32.9, 32.8, 31.1, 28.5, 28.4, 28.1, 24.9, 24.6, 24.3, 24.3, 22.8, 22.8, 22.5, 21.1, 19.9, 19.8, 16.20. HRMS (ESI) calcd for $[C_{47}H_{72}N_2O_7 + Na]^+$: 777.5412, found: 777.5425.

1.6 Asymmetric synthesis of product 7 - 8

The racemic products were synthesized by using carboxylic acid reacting with indoles according to the procedure **G**.

General procedure H for the asymmetric synthesis of 7:

Step 1: To an oven-dried 10 mL-Schlenk tube equipped with a stir bar, was added chiral auxiliary attached indole derivatives **4a** (0.1 mmol, 1.0 equiv), photocatalyst 4CzIPN (4.0 mg, 5 mol%), acid (0.13-0.2 mmol, 1.3-2.0 equiv), Cs_2CO_3 (32.0 mg, 0.1 mmol, 1.0 equiv) and DMF (1.0 mL). The mixture was degassed by freeze-pump-thaw method, then sealed with parafilm. The solution was then stirred at room temperature under the irradiation of a 5w blue LED strip or two 40 w blue LED lamps for indicated time. After completion of the reaction, the mixture was diluted with 10 ml water and extracted by EtOAc (3 x 5 ml). The combined organic layers were washed by 10 ml bine, dried by Na_2SO_4 and concentrated under vacuum. The residue was used in next step without purification.

Step 2. The residue was dissolved in 0.8 ml THF and 0.2 mL water. To the solution was added LiOH (12 mg, 0.5 mmol) and H_2O_2 (113 μl, 30% (w/w) in water) at rt. The reaction was stirred at rt for 10 h, after which 10 ml EtOAc was added. The mixture was washed by 0.5 M NaOH (3 x 5 ml). The combined aqueous layers were collected, washed once with 5 mL Et_2O and acidified with 2 M HCl to pH = 2-3. The aqueous solution was extracted by EtOAc (3 x 5 ml). The combined organic layers were dried by Na_2SO_4 and concentrated under vacuum. The residue was used in next step without purification.

Step 3. The residue was dissolved in 0.75 ml Et_2O and 0.25 mL MeOH. To the solution was added (trimethylsilyl)diazomethane solution (2M, 110 μl) at 0 °C under nitrogen atmosphere, the mixture was stirred at room temperature for 20 min. The reaction mixture was concentrated in vacuo and purified by flash column chromatography or preparative TLC plate to afford product.

General procedure I for the asymmetric synthesis of 8:

Step 1: To an oven-dried 10 mL-Schlenk tube equipped with a stir bar, was added chiral auxiliary attached indole derivatives **4a** (0.1 mmol, 1.0 equiv), photocatalyst 4CzIPN (4.0 mg, 5 mol%), acid (0.13-0.2 mmol, 1.3-2.0 equiv), Cs_2CO_3 (32.0 mg, 0.1 mmol, 1.0 equiv) and DMF (1.0 mL). The mixture was degassed by freeze-pump-thaw method, then sealed with parafilm. The solution was then stirred at room temperature under the irradiation of a 5w blue LED strip or two 40 w blue LED lamps for indicated time. After completion of the reaction, the mixture was diluted with 10 mL of water and extracted by EtOAc (3 × 5 mL). The combined organic layers were washed by 10 mL of bine, dried by Na_2SO_4 and concentrated under vacuum. The residue was used in next step without purification.

Step 2. The residue was dissolved in 0.75 mL of EtOH and 0.25 mL of Et_2O. To the solution was added LiCl (21 mg, 0.5 mmol) and $NaBH_4$ (31.5 mg, 0.5 mmol) at rt. The reaction was stirred at rt for 3 h, after which the solution was concentrated and purified by column chromatography or preparative TLC plate to afford product.

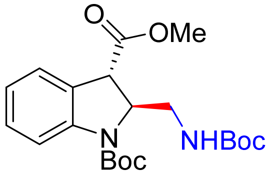

trans-**7a**

trans-**7a** was synthesized according to general procedure **H**. Acid (0.13 mmol, 1.3 equiv), Cs_2CO_3 (32.0 mg, 0.1 mmol, 1.0 equiv), 5 w blue LED strips for 36 h. Purified by flash chromatograph column (10-20 % EtOAc in hexane), 32.9 mg, colorless oil, 81 % yield, 95 % ee. Please refer *trans*-**3v** for ^{1}H and ^{13}C NMR data. $[\alpha]_D^{20}$ +57.5 (*c* 1.1, $CHCl_3$). HPLC: Daicel CHIRALPAK® IC; detected at 254 nm; *n*-hexane /*i*-propanol = 85/15; flow = 0.5 mL/min; Retention time: 14.673 min (2*R*,3*R*, minor), 19.588 min (2*S*,3*S*, major).

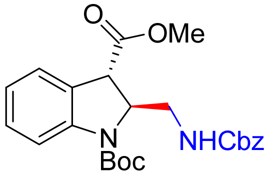

trans-**7b**

trans-**7b** was synthesized according to general procedure **H**. Acid (0.13 mmol, 1.3 equiv), Cs_2CO_3 (32.0 mg, 0.1 mmol, 1.0 equiv), 5 w blue LED strips for 36 h. Purified by flash chromatograph column (10-25 % EtOAc in hexane), 34.3 mg, 78 % yield, 93 % ee. $[\alpha]_D^{21}$ +32.0 (*c* 1.1, $CHCl_3$). ^{1}H NMR (400 MHz, $CDCl_3$) δ 7.65 (s, 1H), 7.40 – 7.22 (m, 7H), 6.99 (t, *J* = 7.3 Hz, 1H), 5.23 (s, 1H), 5.06 (s, 2H), 4.92 (d, *J* = 2.4 Hz, 1H), 3.97 (s, 1H), 3.71 (s, 3H), 3.75-3.53 (m, 2H), 1.57 (s, 9H). ^{13}C NMR (100 MHz, $CDCl_3$) δ 171.4, 156.9, 152.3, 141.9, 136.4, 129.1, 128.6, 128.2, 128.0, 127.0, 125.9, 123.0, 115.8, 82.1, 66.9, 61.6, 52.7, 49.1, 44.2, 28.4. HPLC: Daicel CHIRALPAK® IC; detected at 254 nm; *n*-hexane /*i*-propanol = 75/25; flow = 0.5 mL/min; Retention time: 15.961 min (2*R*,3*R*, minor), 17.982 min (2*S*,3*S*, major). HRMS (ESI) calcd for $[C_{24}H_{28}N_2O_6 + Na]^+$: 463.1840, found: 463.1824.

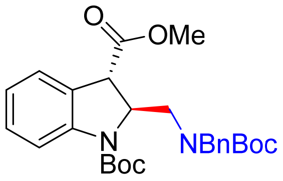

trans-**7c**

trans-**7c** was synthesized according to general procedure **H**. Acid (0.13 mmol, 1.3 equiv), Cs_2CO_3 (32.0 mg, 0.1 mmol, 1.0 equiv), two 40 w blue LED lamps for 9 h. Purified by flash chromatograph column (10 % acetone in hexane), 37.2 mg, 75% yield, 84% ee. $[\alpha]_D^{22}$ +50.5 (*c* 1.1, $CHCl_3$). ^{1}H NMR (400 MHz, $CDCl_3$) δ 7.74 (s, 1H), 7.41 – 7.23 (m, 7H), 6.99 (t, *J* = 7.4 Hz, 1H), 4.97 (s, 1H), 4.63 – 4.37 (m, 2H), 4.28 (s, rotamer, 0.5H), 3.96 (s, rotamer, 0.5H), 3.68 (s, rotamer, 3.5H), 3.49 – 3.39 (m, rotamer, 1.5H), 1.53 (s, 9H), 1.41 (s, 9H). ^{13}C NMR (100 MHz, $CDCl_3$) δ 171.8, 156.5, 155.9, 151.9, 141.8, 138.2, 138.1, 129.0, 128.9, 128.6, 127.3, 126.0, 123.0, 116.0, 115.6, 81.7, 80.5, 61.2, 60.2, 52.6, 51.2, 50.3, 48.8, 48.2, 47.9, 28.5, 28.4. HPLC: Daicel CHIRALPAK® IC; detected at 254 nm; *n*-hexane /*i*-propanol = 75/25; flow = 0.5 mL/min; Retention time: 9.766 min (2*R*,3*R*, minor), 11.869 min (2*S*,3*S*, major). HRMS (ESI) calcd for $[C_{28}H_{36}N_2O_6 + Na]^+$: 519.2466, found: 519.2449.

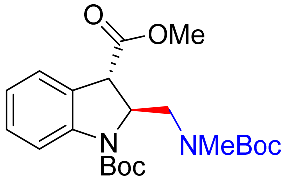

trans-**7d**

trans-**7d** was synthesized according to general procedure **H**. Acid (0.13 mmol, 1.3 equiv), Cs_2CO_3 (32.0 mg, 0.1 mmol, 1.0 equiv), 5 w blue LED strips for 36 h. Purified by flash chromatograph column (10-20 % EtOAc in hexane), 37.2 mg, 72% yield, 90% ee. $[\alpha]_D^{21}$ +96.6 (*c* 1.1, $CHCl_3$). ^{1}H NMR (400 MHz, $CDCl_3$) δ 7.78 (s, br, 1H), 7.34 (d, *J* = 7.4 Hz, 1H), 7.22 (s, 1H), 6.97 (s, 1H), 4.94 (s, 1H), 4.26 (s, rotamer, 0.5H), 3.99 (s, rotamer, 0.5H), 3.69 (s, rotamer, 3.5H), 3.51 - 3.39 (m, 1.5H), 2.91-2.85 (s, s, rotamer, 3H), 1.57 (s, 9H), 1.39 (s, 9H). ^{13}C NMR (100 MHz, $CDCl_3$) δ 171.9, 171.6, 156.7, 155.6, 151.9, 141.8, 129.1, 128.5, 127.0, 126.0, 123.0, 115.9, 115.5, 81.7, 80.2, 79.8, 61.0, 60.2, 52.6, 50.3, 48.7, 48.3, 35.3, 34.6, 28.5, 28.4. HPLC: Daicel CHIRALPAK® IB; detected at 254 nm; *n*-hexane /*i*-propanol = 98/2; flow = 0.5 mL/min; Retention time: 11.230 min (2*R*,3*R*, minor), 13.134 min (2*S*,3*S*, major). HRMS (ESI) calcd for $[C_{22}H_{32}N_2O_6 + Na]^+$: 443.2153, found: 443.2137.

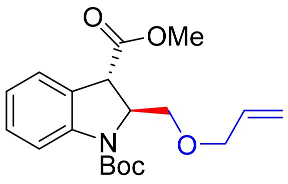

trans-**7e**

trans-**7e** was synthesized according to general procedure **H**. Acid (0.20 mmol, 2.0 equiv), Cs_2CO_3 (32.0 mg, 0.1 mmol, 1.0 equiv), two 40 W blue LED lamps for 36 h. Purified by flash chromatograph column (10-20 % EtOAc in hexane), 26.0 mg, 75% yield, 86% ee. $[\alpha]_D^{23}$ +91.7 (*c* 1.0, $CHCl_3$). ^1H NMR (400 MHz, $CDCl_3$) δ 7.75 (s, br, 1H), 7.36 (d, *J* = 7.5 Hz, 1H), 7.22 (t, *J* = 7.7 Hz, 1H), 6.98 (t, *J* = 7.5 Hz, 1H), 5.83 (ddd, J = 22.6, 10.7, 5.5 Hz, 1H), 5.17 (dd, *J* = 19.5, 13.9 Hz, 2H), 4.96 (s, 1H), 4.13 (d, *J* = 2.0 Hz, 1H), 4.03 – 3.94 (m, 2H), 3.76 – 3.73 (m, 4H), 3.48 (t, *J* = 7.3 Hz, 1H), 1.57 (s, 9H). ^{13}C NMR (100 MHz, $CDCl_3$) δ 171.9, 152.0, 142.4, 134.5, 128.9, 127.3, 125.9, 122.8, 117.2, 115.6, 81.6, 72.1, 69.7, 61.1, 52.7, 48.7, 28.5. HPLC: Daicel CHIRALPAK® IB; detected at 254 nm; *n*-hexane /*i*-propanol = 95/5; flow = 0.5 mL/min; Retention time: 8.560 min (2*R*,3*R*, minor), 9.991 min (2*S*,3*S*, major). HRMS (ESI) calcd for $[C_{19}H_{25}NO_5 + Na]^+$: 370.1625, found: 370.1611.

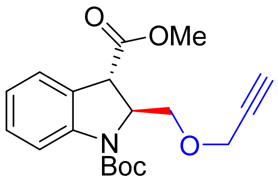

trans-**7f**

trans-**7f** was synthesized according to general procedure **H**. Acid (0.20 mmol, 2.0 equiv), Cs_2CO_3 (32.0 mg, 0.1 mmol, 1.0 equiv), two 40 W blue LED lamps for 36 h. Purified by flash chromatograph column (10-20 % EtOAc in hexane), 26.0 mg, 71% yield, 89% ee. $[\alpha]_D^{23}$ +112.4 (*c* 1.0, $CHCl_3$). ^1H NMR (400 MHz, $CDCl_3$) δ 7.75 (s, br, 1H), 7.36 (d, *J* = 7.4 Hz, 1H), 7.23 (t, *J* = 7.7 Hz, 1H), 6.98 (t, *J* = 7.4 Hz, 1H), 4.98 (s, 1H), 4.14 (s, 2H), 4.12 (s, 1H), 3.84 (dd, *J* = 8.9, 2.7 Hz, 1H), 3.73 (s, 3H), 3.59 (s, 1H), 2.42 (s, 1H), 1.58 (s, 9H). ^{13}C NMR (100 MHz, $CDCl_3$) δ 171.8, 151.9, 142.3, 129.0, 127.1, 125.8, 122.8, 115.6, 81.7, 79.5, 77.5, 77.2, 76.8, 74.9, 69.4, 60.9, 58.5, 52.7, 48.7, 28.5. HPLC: Daicel CHIRALPAK® IB; detected at 254 nm; *n*-hexane /*i*-propanol = 95/5; flow = 0.5 mL/min; Retention time: 10.365 min (2*R*,3*R*, minor), 12.437 min (2*S*,3*S*, major). HRMS (ESI) calcd for $[C_{19}H_{23}NO_5 + Na]^+$: 368.1468, found: 368.1454.

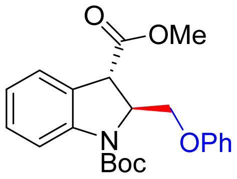

trans-**7g**

trans-**7g** was synthesized according to general procedure **H**. Acid (0.20 mmol, 2.0 equiv), Cs_2CO_3 (32.0 mg, 0.1 mmol, 1.0 equiv), two 40 W blue LED lamps for 36 h. Purified by flash chromatograph column (0-20 % EtOAc in hexane), 23.0 mg, 60% yield, 87% ee. $[\alpha]_D^{23}$ +66.2 (*c* 1.0, $CHCl_3$). Please refer *trans*-**3ah** for ^1H and ^{13}C NMR data. HPLC: Daicel CHIRALPAK® IB;

detected at 254 nm; *n*-hexane /*i*-propanol = 96/4; flow = 0.5 mL/min; Retention time: 10.406 min (2*R*,3*R*, minor), 12.148 min (2*S*,3*S*, major).

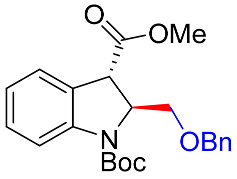

trans-**7h**

trans-**7h** was synthesized according to general procedure **H**. Acid (0.13 mmol, 1.3 equiv), Cs_2CO_3 (32.0 mg, 0.1 mmol, 1.0 equiv), 5 W blue LED strips for 36 h. Purified by flash chromatograph column (0-20 % EtOAc in hexane), 30.2 mg, 76% yield, 90% ee. $[\alpha]_D^{23}$ +102.7 (*c* 1.1, $CHCl_3$). Please refer *trans*-**3ag** for ^1H and ^{13}C NMR data. HPLC: Daicel CHIRALPAK® IC; detected at 254 nm; *n*-hexane /*i*-propanol = 75/25; flow = 0.5 mL/min; Retention time: 9.700 min (2*R*,3*R*, minor), 11.178 min (2*S*,3*S*, major).

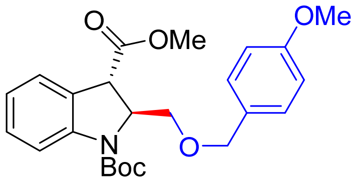

trans-**7i**

trans-**7i** was synthesized according to general procedure **H**. Acid (0.20 mmol, 2.0 equiv), Cs_2CO_3 (32.0 mg, 0.1 mmol, 1.0 equiv), two 40 W blue LED lamps for 36 h. Purified by flash chromatograph column (10-20 % EtOAc in hexane), 29.9 mg, 70% yield, 88% ee. $[\alpha]_D^{23}$ +83.0 (*c* 1.0, $CHCl_3$). ^1H NMR (400 MHz, $CDCl_3$) δ 7.80 (s, br, 1H), 7.37 (d, *J* = 7.4 Hz, 1H), 7.23 (d, *J* = 7.7 Hz, 1H), 7.16 (d, *J* = 8.4 Hz, 2H), 6.99 (t, *J* = 7.5 Hz, 1H), 6.85 (d, *J* = 8.5 Hz, 2H), 4.99 (s, 1H), 4.52 – 4.38 (m, 2H), 4.14 (s, 1H), 3.75 (d, J = 25.3 Hz, 7H), 3.49 (s, 1H), 1.54 (s, 9H). ^{13}C NMR (100 MHz, $CDCl_3$) δ 171.8, 159.3, 151.9, 142.4, 130.1, 129.2, 128.9, 127.3, 125.8, 122.7, 115.5, 113.8, 81.4, 72.8, 69.4, 61.1, 55.3, 52.6, 48.7, 28.4. HPLC: Daicel CHIRALPAK® IB; detected at 254 nm; *n*-hexane /*i*-propanol = 95/5; flow = 0.5 mL/min; Retention time: 12.069 min (2*R*,3*R*, minor), 15.908 min (2*S*,3*S*, major). HRMS (ESI) calcd for $[C_{24}H_{29}NO_6 + Na]^+$: 450.1887, found: 450.1872.

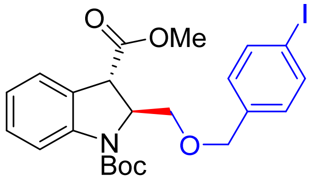

trans-**7j**

trans-**7j** was synthesized according to general procedure **H**. Acid (0.20 mmol, 2.0 equiv), Cs_2CO_3 (32.0 mg, 0.1 mmol, 1.0 equiv), two 40 W blue LED lamps for 36 h. Purified by flash chromatograph column (10-20 % EtOAc in hexane), 29.9 mg, 70% yield, 88% ee. $[\alpha]_D^{23}$ +49.5 (*c* 1.0, $CHCl_3$). ^1H NMR (400 MHz, $CDCl_3$) δ 7.79 (s, br, 1H), 7.63 (d, *J* = 8.1 Hz, 2H), 7.37 (d, *J* = 7.4 Hz, 1H), 7.23 (d, *J* = 7.7 Hz, 1H), 7.04 – 6.91 (m, 3H), 4.98 (s, 1H), 4.45 (q, *J* = 12.4 Hz, 2H), 4.13 (s, 1H), 3.73 (s, 4H), 3.54 (s, 1H), 1.53 (s, 9H). ^{13}C NMR (100 MHz, $CDCl_3$) δ 171.8, 151.9,

142.4, 137.8, 137.6, 129.4, 129.0, 127.2, 125.8, 122.8, 115.5, 93.2, 81.8, 72.5, 70.1, 61.2, 52.7, 48.7, 28.5. HPLC: Daicel CHIRALPAK® IB; detected at 254 nm; *n*-hexane /*i*-propanol = 95/5; flow = 0.5 mL/min; Retention time: 11.253 min (2*R*,3*R*, minor), 14.747 min (2*S*,3*S*, major). HRMS (ESI) calcd for $[C23H_{26}INO_5 + Na]^+$: 546.0748, found: 546.0729.

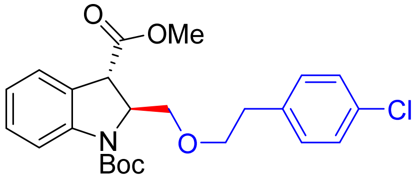

trans-**7k**

trans-**7k** was synthesized according to general procedure **H**. Acid (0.20 mmol, 2.0 equiv), Cs_2CO_3 (32.0 mg, 0.1 mmol, 1.0 equiv), two 40 W blue LED lamps for 36 h. Purified by flash chromatograph column (10-20 % EtOAc in hexane), 34.8 mg, 78% yield, 83% ee. $[\alpha]_D^{23}$ +12.0 (*c* 1.1, $CHCl_3$). ^1H NMR (400 MHz, $CDCl_3$) δ 7.80 (s, br, 1H), 7.34 (d, *J* = 7.4 Hz, 1H), 7.21 (dd, *J* = 15.2, 8.1 Hz, 3H), 7.00 (t, *J* = 8.5 Hz, 3H), 4.93 (s, 1H), 3.99 (s, 1H), 3.72 (s, 3H), 3.66 (dd, *J* = 9.2, 6.5 Hz, 2H), 3.61 – 3.55 (m, 1H), 3.51 (s, 1H), 2.74 (t, *J* = 6.4 Hz, 2H), 1.57 (s, 9H). ^{13}C NMR (100 MHz, $CDCl_3$) δ 171.8, 151.9, 137.6, 131.9, 130.4, 128.9, 128.4, 127.3, 125.8, 122.7, 115.5, 81.5, 71.8, 70.6, 61.0, 52.6, 48.5, 35.7, 28.5. HPLC: Daicel CHIRALPAK® IB; detected at 254 nm; *n*-hexane /*i*-propanol = 95/5; flow = 0.5 mL/min; Retention time: 10.018 min (2*R*,3*R*, minor), 11.967 min (2*S*,3*S*, major). HRMS (ESI) calcd for $[C_{24}H_{28}ClNO_5 + Na]^+$: 468.1548, found: 468.1532.

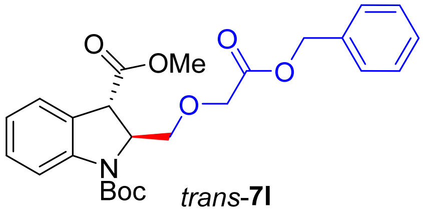

trans-**7l**

trans-**7l** was synthesized according to general procedure **H**. Acid (0.20 mmol, 2.0 equiv), Cs_2CO_3 (32.0 mg, 0.1 mmol, 1.0 equiv), two 40 W blue LED lamps for 36 h. Purified by flash chromatograph column (10-20 % EtOAc in hexane), 33.7 mg, 74% yield, 84% ee. $[\alpha]_D^{23}$ +74.7 (*c* 1.1, $CHCl_3$). ^1H NMR (400 MHz, $CDCl_3$) δ 7.77 (s, br, 1H), 7.41 – 7.32 (m, 6H), 7.23 (t, *J* = 7.8 Hz, 1H), 6.99 (t, *J* = 7.5 Hz, 1H), 5.16 (s, 2H), 5.00 (s, 1H), 4.32 (d, *J* = 2.2 Hz, 1H), 4.13 (s, 2H), 3.87 (dd, *J* = 9.2, 3.6 Hz, 1H), 3.73 (s, 3H), 3.65 (s, 1H), 1.57 (s, 9H). ^{13}C NMR (100 MHz, $CDCl_3$) δ 171.8, 170.1, 151.9, 142.2, 135.4, 128.9, 128.7, 128.5, 128.4, 125.9, 122.8, 115.5, 81.6, 71.2, 68.3, 66.6, 60.8, 52.6, 48.4, 28.5. HPLC: Daicel CHIRALPAK® IB; detected at 254 nm; *n*-hexane /*i*-propanol = 75/25; flow = 0.5 mL/min; Retention time: 9.250 min (2*R*,3*R*, minor), 9.945 min (2*S*,3*S*, major). HRMS (ESI) calcd for $[C_{25}H_{29}NO_7 + Na]^+$: 478.1836, found: 478.1819.

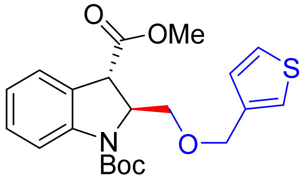

trans-**7m**

trans-**7m** was synthesized according to general procedure **H**. Acid (0.20 mmol, 2.0 equiv), Cs_2CO_3 (32.0 mg, 0.1 mmol, 1.0 equiv), two 40 W blue LED lamps for 36 h. Purified by flash chromatograph column (10-20 % EtOAc in hexane), 32.6 mg, 81% yield, 80% ee. $[\alpha]_D^{24}$ -135.8 (*c* 1.5, $CHCl_3$). ^{1}H NMR (400 MHz, $CDCl_3$) δ 7.79 (s, br, 1H), 7.37 (d, *J* = 7.5 Hz, 1H), 7.28 – 7.22 (m, 2H), 7.11 (s, 1H), 6.99 (dd, *J* = 13.0, 5.9 Hz, 2H), 4.99 (s, 1H), 4.60 – 4.47 (m, 2H), 4.14 (d, *J* = 1.8 Hz, 1H), 3.81 – 3.68 (m, 4H), 3.53 (d, *J* = 8.8 Hz, 1H), 1.55 (s, 9H). ^{13}C NMR (100 MHz, $CDCl_3$) 171.8, 151.9, 142.4, 139.2, 128.9, 127.1, 126.1, 125.8, 122.8, 122.7, 115.5, 81.5, 69.7, 68.5, 61.1, 52.6, 48.7, 28.4. HPLC: Daicel CHIRALPAK® IB; detected at 254 nm; *n*-hexane /*i*-propanol = 95/5; flow = 0.5 mL/min; Retention time: 11.217 min (2*R*,3*R*, minor), 13.812 min (2*S*,3*S*, major). HRMS (ESI) calcd for $[C_{21}H_{25}NO_5S + Na]^+$: 426.1346, found: 426.1331.

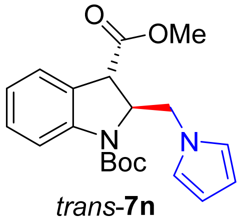

trans-**7n**

trans-**7n** was synthesized according to general procedure **H**. Acid (0.20 mmol, 2.0 equiv), Cs_2CO_3 (32.0 mg, 0.1 mmol, 1.0 equiv), 5 W blue LED strips for 36 h. Purified by flash chromatograph column (10-20 % EtOAc in hexane), 18.5 mg, 52% yield, 90% ee. $[\alpha]_D^{23}$ +57.3 (*c* 1.0, $CHCl_3$). ^{1}H NMR (400 MHz, $CDCl_3$) δ 7.77 (s, br, 1H), 7.34 (d, *J* = 7.4 Hz, 1H), 7.26 (t, *J* = 7.4 Hz, 1H), 7.00 (t, *J* = 7.3 Hz, 1H), 6.64 (s, 2H), 6.14 (s, 2H), 5.15 (s, 1H), 4.35 (d, *J* = 13.1 Hz, 1H), 3.97 – 3.82 (m, 1H), 3.78 (s, 1H), 3.66 (s, 3H), 1.62 (s, 9H). ^{13}C NMR (100 MHz, $CDCl_3$) 171.0, 151.8, 141.7, 129.3, 126.4, 126.0, 123.0, 121.2, 115.8, 109.0, 82.0, 62.2, 52.7, 51.0, 48.6, 28.5. HPLC: Daicel CHIRALPAK® IC; detected at 254 nm; *n*-hexane /*i*-propanol = 95/5; flow = 0.5 mL/min; Retention time: 13.118 min (2*R*,3*R*, minor), 14.227 min (2*S*,3*S*, major). HRMS (ESI) calcd for $[C_{20}H_{24}N_2O_4 + Na]^+$: 379.1628, found: 379.1614.

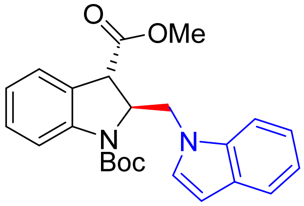

trans-**7o**

trans-**7o** was synthesized according to general procedure **H**. Acid (0.13 mmol, 1.3 equiv), Cs_2CO_3 (32.0 mg, 0.1 mmol, 1.0 equiv), 5 W blue LED strips for 36 h. Purified by flash chromatograph column (10-20 % EtOAc in hexane), 30.8 mg, 76% yield, 86% ee. $[\alpha]_D^{24}$ +90.8 (*c* 1.1, $CHCl_3$). ^{1}H NMR (400 MHz, $CDCl_3$) δ 7.92 (s, br, 1H), 7.65 (d, *J* = 7.6 Hz, 1H), 7.47 (s, 1H), 7.31 (dt, *J* = 15.2, 6.7 Hz, 3H), 7.15 (t, *J* = 7.2 Hz, 1H), 7.07 (d, *J* = 12.2 Hz, 2H), 6.54 (s, 1H), 5.37 (s, 1H), 4.52 (d, *J* = 12.3 Hz, 1H), 4.10 (dd, *J* = 14.0, 8.6 Hz, 1H), 3.79 (s, 1H), 3.58 (s, 3H), 1.54 (d, *J* = 22.6 Hz, 9H). ^{13}C NMR (100 MHz, $CDCl_3$) 170.7, 151.9, 141.6, 136.7, 129.4, 128.6, 128.0, 126.5, 126.2, 123.2, 122.0, 121.1, 119.8, 116.1, 109.3, 102.4, 82.0, 61.2, 52.6, 48.7, 48.9, 28.4. HPLC: Daicel CHIRALPAK® IB; detected at 254 nm; *n*-hexane /*i*-propanol = 90/10; flow = 0.5 mL/min; Retention time: 12.839 min (2*R*,3*R*, minor), 15.049 min (2*S*,3*S*, major). HRMS (ESI) calcd for $[C_{24}H_{26}N_2O_4 + Na]^+$: 429.1785, found: 429.1790.

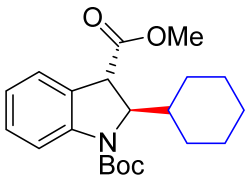

trans-**7p**

trans-**7p** was synthesized according to general procedure **H**. Acid (0.20 mmol, 2.0 equiv), Cs_2CO_3 (32.0 mg, 0.1 mmol, 1.0 equiv), two 40 W blue LED lamps for 36 h. Purified by flash chromatograph column (10% EtOAc in hexane), 32.7 mg, 91% yield, 93% ee. $[\alpha]_D^{24}$ +5.0 (*c* 1.1, $CHCl_3$). Please refer *trans*-**3aj** for ^1H and ^{13}C NMR data. HPLC: Daicel CHIRALPAK® IB; detected at 254 nm; *n*-hexane /*i*-propanol = 95/5; flow = 0.5 mL/min; Retention time: 7.878 min (2*S*,3*R*, minor), 9.032 min (2*R*,3*S*, major).

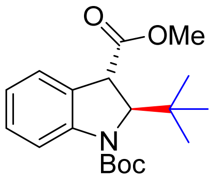

trans-**7q**

trans-**7q** was synthesized according to general procedure **H**. Acid (0.20 mmol, 2.0 equiv), Cs_2CO_3 (32.0 mg, 0.1 mmol, 1.0 equiv), two 40 W blue LED lamps for 36 h. Purified by flash chromatograph column (10 % EtOAc in hexane), 18.3 mg, 55% yield, 95% ee. $[\alpha]_D^{22}$ +60.9 (*c* 1.1, $CHCl_3$). Please refer *trans*-**3al** for ^1H and ^{13}C NMR data. HPLC: Daicel CHIRALPAK® IC; detected at 254 nm; *n*-hexane /*i*-propanol = 95/5; flow = 0.5 mL/min; Retention time: 9.377 min (2*R*,3*R* major), 10.470 min (2*S*,3*S*, minor).

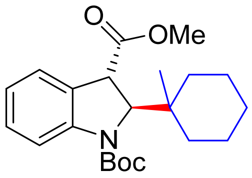

trans-**7r**

trans-**7r** was synthesized according to general procedure **H**. Acid (0.13 mmol, 1.3 equiv), Cs_2CO_3 (32.0 mg, 0.1 mmol, 1.0 equiv), 5 W blue LED strips for 36 h. Purified by flash chromatograph column (10-20% EtOAc in hexane), 19.4 mg, 52% yield, 98% ee. $[\alpha]_D^{23}$ -20.9 (*c* 1.1, $CHCl_3$). Please refer *trans*-**3am** for ^1H and ^{13}C NMR data. HPLC: Daicel CHIRALPAK® IC; detected at 254 nm; *n*-hexane /*i*-propanol = 95/5; flow = 0.5 mL/min; Retention time: 9.552 min (2*S*,3*S*, major), 10.700 min (2*R*,3*R*, minor).

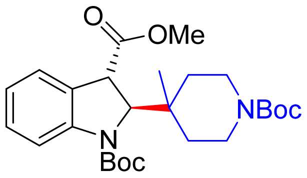

trans-**7s**

trans-**7s** was synthesized according to general procedure **H**. Acid (0.13 mmol, 1.3 equiv), Cs_2CO_3 (32.0 mg, 0.1 mmol, 1.0 equiv), two 40 W blue LED lamps for 36 h. Purified by flash chromatograph column (10-20% EtOAc in hexane), 24.2 mg, 51% yield, 97% ee. $[\alpha]_D^{23}$ -10.1 (*c* 1.1, $CHCl_3$). Please refer *trans*-**3aq** for ^1H and ^{13}C NMR data. HPLC: Daicel CHIRALPAK® IC; detected at 254 nm; *n*-hexane /*i*-propanol = 95/5; flow = 0.5 mL/min; Retention time: 32.740 min (2*S*,3*S*, major), 37.610 min (2*R*,3*R*, minor).

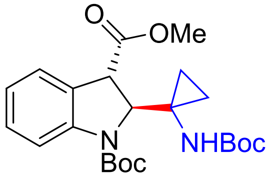

trans-**7t**

trans-**7t** was synthesized according to general procedure **H**. Acid (0.13 mmol, 1.3 equiv), Cs_2CO_3 (32.0 mg, 0.1 mmol, 1.0 equiv), two 40 W blue LED lamps for 36 h. Purified by flash chromatograph column (10-20% EtOAc in hexane), 17.8 mg, 41% yield, 97% ee. $[\alpha]_D^{23}$ -57.9 (*c* 1.0, $CHCl_3$). Please refer *trans*-**3ae** for ^1H and ^{13}C NMR data. HPLC: Daicel CHIRALPAK® IC; detected at 254 nm; *n*-hexane /*i*-propanol = 95/5; flow = 0.5 mL/min; Retention time: 14.160 min (2*R*,3*R*, minor), 17.392 min (2*S*,3*S*, major).

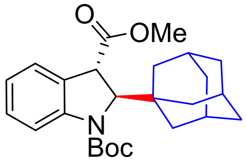

trans-**7u**

trans-**7u** was synthesized according to general procedure **H**. Acid (0.15 mmol, 1.5 equiv), Cs_2CO_3 (32.0 mg, 0.1 mmol, 1.0 equiv), two 40 W blue LED lamps for 36 h. Purified by flash chromatograph column (10-20% EtOAc in hexane), 30.4 mg, 74% yield, 97% ee. $[\alpha]_D^{23}$ -49.2 (*c* 1.0, $CHCl_3$). Please refer *trans*-**3ap** for ^1H and ^{13}C NMR data. HPLC: Daicel CHIRALPAK® IC; detected at 254 nm; *n*-hexane /*i*-propanol = 98/2; flow = 0.5 mL/min; Retention time: 12.866 min (2*S*,3*S*, major), 16.766 min (2*R*,3*R*, minor).

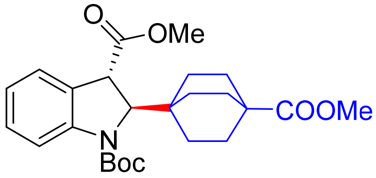

trans-**7v**

trans-**7v** was synthesized according to general procedure **H**. Acid (0.20 mmol, 2.0 equiv), Cs_2CO_3 (32.0 mg, 0.1 mmol, 1.0 equiv), 5 W blue LED strips for 36 h. Purified by flash chromatograph column (10-20% EtOAc in hexane), 27.0 mg, 61% yield, 98% ee. $[\alpha]_D^{23}$ -49.6 (*c* 1.1, $CHCl_3$). Please refer *trans*-**3an** for ^1H and ^{13}C NMR data. HPLC: Daicel CHIRALPAK® IB; detected at 254 nm; *n*-hexane /*i*-propanol = 95/5; flow = 0.5 mL/min; Retention time: 11.467 min (2*R*,3*R*, minor), 12.657 min (2*S*,3*S*, major).

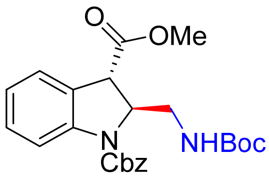

trans-**7w**

trans-**7w** was synthesized according to general procedure **H**. Acid (0.13 mmol, 1.3 equiv), Cs_2CO_3 (32.0 mg, 0.1 mmol, 1.0 equiv), 5 W blue LED strips for 36 h. Purified by flash chromatograph column (10-20 % EtOAc in hexane), 35.6 mg, 81% yield, 94% ee. $[\alpha]_D^{22}$ +16.2 (*c* 1.1, $CHCl_3$ Please refer *trans*-**3k** for ^{1}H and ^{13}C NMR data. HPLC: Daicel CHIRALPAK® IA; detected at 254 nm; *n*-hexane /*i*-propanol = 85/15; flow = 0.5 mL/min; Retention time: 18.594 min (2*S*,3*S*, major), 22.977 min (2*R*,3*R*, minor).

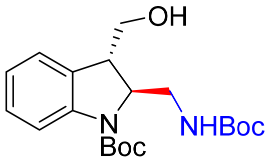

trans-**8a**

trans-**8a** was synthesized according to general procedure **I**. Acid (0.13 mmol, 1.3 equiv), Cs_2CO_3 (32.0 mg, 0.1 mmol, 1.0 equiv), 5 W blue LED strips for 36 h. Purified by flash chromatograph column (20 % EtOAc in hexane), 30.4 mg, 80% yield, 91% ee. $[\alpha]_D^{21}$ -168.2 (*c* 1.1, $CHCl_3$). ^{1}H NMR (400 MHz, $CDCl_3$) δ 7.64 (s, br, 1H), 7.24 – 7.12 (m, 2H), 6.95 (t, *J* = 7.4 Hz, 1H), 5.04 (s, 1H), 4.39 (s, 1H), 3.72 (dd, *J* = 10.7, 5.2 Hz, 1H), 3.58 (t, *J* = 9.2 Hz, 1H), 3.48 – 3.27 (m, 2H), 3.17 (s, 1H), 2.62 (s, 1H), 1.57 (s, 9H), 1.38 (s, 9H). ^{13}C NMR (100 MHz, $CDCl_3$) 156.4, 152.8, 142.4, 130.3, 128.4, 124.9, 122.8, 115.7, 81.8, 79.5, 65.4, 62.6, 47.3, 44.3, 28.5, 28.4. HPLC: Daicel CHIRALPAK® IA; detected at 254 nm; *n*-hexane /*i*-propanol = 90/10; flow = 0.5 mL/min; Retention time: 11.274 min (2*S*,3*S*, major), 16.523 min (2*R*,3*R*, minor). HRMS (ESI) calcd for $[C_{20}H_{30}NO_5 + Na]^+$: 401.2047, found: 401.2029.

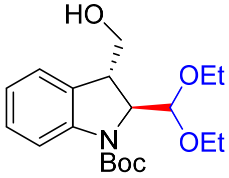

trans-**8b**

trans-**8b** was synthesized according to general procedure **I**. Acid (0.13 mmol, 1.3 equiv), Cs_2CO_3 (32.0 mg, 0.1 mmol, 1.0 equiv), 5 W blue LED strips for 36 h. Purified by flash chromatograph column (10-20 % EtOAc in hexane), 30.9 mg, 88% yield, 90% ee. $[\alpha]_D^{24}$ -358.3 (*c* 1.1, $CHCl_3$). ^{1}H NMR (400 MHz, $CDCl_3$) δ 7.74 (s, br, 1H), 7.14 (dd, *J* = 12.7, 7.4 Hz, 2H), 6.93 (t, *J* = 7.4 Hz, 1H), 4.94 (s, 1H), 4.31 (s, 1H), 3.85 – 3.72 (m, 2H), 3.73 – 3.53 (m, 4H), 3.50 – 3.37 (m, 1H), 2.30 (s, 1H), 1.57 (s, 9H), 1.24 (t, *J* = 7.0 Hz, 3H), 0.95 (t, *J* = 7.0 Hz, 3H). ^{13}C NMR (100 MHz, $CDCl_3$) 152.5, 143.1, 131.3, 127.9, 124.2, 122.5, 115.0, 101.5, 81.5, 65.9, 65.9, 65.7, 64.0, 43.1, 28.5, 15.4, 15.3. HPLC: Daicel CHIRALPAK® IC; detected at 254 nm; *n*-hexane /*i*-propanol = 75/25; flow = 0.5 mL/min; Retention time: 8.926 min (2*S*,3*S*, major), 11.823 min (2*R*,3*R*, minor). HRMS (ESI) calcd for $[C_{19}H_{29}NO_5 + Na]^+$: 374.1938, found: 374.1922.

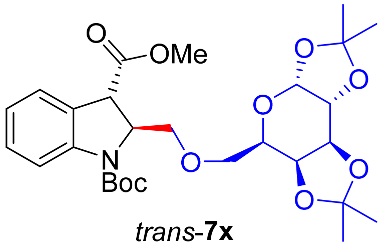

trans-**7x**

trans-**7x** was synthesized according to general procedure **H**. Acid (0.15 mmol, 1.5 equiv), Cs_2CO_3 (32.0 mg, 0.1 mmol, 1.0 equiv), 5 W blue LED strips for 36 h. Purified by flash chromatograph column (20 % EtOAc in hexane), 38.4 mg, 70% yield, 88% ee. $[\alpha]_D^{24}$ -156.1 (*c* 1.1, $CHCl_3$). ^1H NMR (400 MHz, $CDCl_3$) δ 7.78 (s, br, 1H), 7.35 (d, *J* = 7.4 Hz, 1H), 7.21 (t, *J* = 7.6 Hz, 1H), 6.96 (t, *J* = 7.5 Hz, 1H), 5.48 (d, *J* = 4.9 Hz, 1H), 4.95 (s, 1H), 4.58 – 4.51 (m, 1H), 4.27 (dd, *J* = 4.8, 2.2 Hz, 1H), 4.18 (s, 1H), 4.09 (d, *J* = 8.0 Hz, 1H), 3.87 (t, *J* = 5.8 Hz, 1H), 3.79 – 3.66 (m, 5H), 3.57 (dd, *J* = 10.0, 6.6 Hz, 2H), 1.57 (s, 8H), 1.50 (s, 3H), 1.41 (s, 3H), 1.31 (s, 6H). ^{13}C NMR (100 MHz, $CDCl_3$) 172.0, 152.0, 142.4, 128.9, 125.9, 122.7, 115.6, 109.3, 108.7, 96.4, 81.6, 71.1, 70.7, 69.9, 66.9, 61.0, 52.6, 48.5, 28.5, 26.2, 26.1, 25.1, 24.5. HPLC: Daicel CHIRALPAK® IB; detected at 254 nm; *n*-hexane /*i*-propanol = 95/5; flow = 0.5 mL/min; Retention time: 12.884 min (2*S*,3*S*, major), 15.000 min (2*R*,3*R*, minor). HRMS (ESI) calcd for $[C_{28}H_{39}N_2O_{10} + Na]^+$: 572.2466, found: 572.2443.

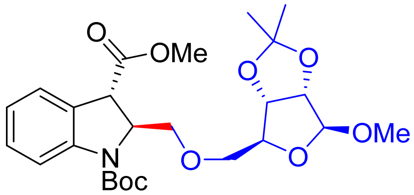

trans-**7y**

trans-**7y** was synthesized according to general procedure **H**. Acid (0.15 mmol, 1.5 equiv), Cs_2CO_3 (32.0 mg, 0.1 mmol, 1.0 equiv), 5 W blue LED strips for 36 h. Purified by flash chromatograph column (20 % EtOAc in hexane), 39.9 mg, 81% yield, 87% ee. $[\alpha]_D^{25}$ -291.4 (*c* 1.1, $CHCl_3$). ^1H NMR (400 MHz, $CDCl_3$) δ 7.78 (s, br, 1H), 7.35 (d, *J* = 7.4 Hz, 1H), 7.22 (t, *J* = 7.6 Hz, 1H), 6.98 (t, *J* = 7.4 Hz, 1H), 4.96 (s, 1H), 4.91 (s, 1H), 4.54 (d, *J* = 5.9 Hz, 1H), 4.45 (d, *J* = 6.0 Hz, 1H), 4.24 (t, *J* = 6.7 Hz, 1H), 4.14 (d, *J* = 2.5 Hz, 1H), 3.73 (s, 4H), 3.56 – 3.42 (m, 3H), 3.25 (s, 3H), 1.57 (s, 9H), 1.45 (s, 3H), 1.28 (s, 3H). ^{13}C NMR (100 MHz, $CDCl_3$) 171.9, 151.9, 142.3, 129.0, 126.0, 122.8, 112.4, 109.6, 85.3, 84.9, 82.0, 81.6, 72.2, 70.9, 61.0, 60.5, 54.9, 52.7, 48.6, 29.8, 28.6, 26.5, 25.0. HPLC: Daicel CHIRALPAK® IB; detected at 254 nm; *n*-hexane /*i*-propanol = 96/4; flow = 0.5 mL/min; Retention time: 11.574 min (2*R*,3*R*, minor), 13.228 min (2*S*,3*S*, major). HRMS (ESI) calcd for $[C_{25}H_{35}N_2O_9 + Na]^+$: 516.2204, found: 516.2201.

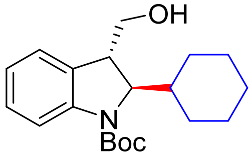

trans-**8c**

trans-**8c** was synthesized according to general procedure **I**. Acid (2.0 mmol, 2.0 equiv), Cs_2CO_3 (320 mg, 1.0 mmol, 1.0 equiv), two 40 W blue lamps for 36 h. Purified by flash chromatograph

column (10-20 % EtOAc in hexane), 238 mg, 72% yield, 94% ee. $[\alpha]_D^{24}$ -124.1 (*c* 1.1, $CHCl_3$). ^1H NMR (400 MHz, $CDCl_3$) δ 7.78 – 7.45 (s, s, 1H), 7.23 – 7.12 (m, 2H), 6.93 (t, *J* = 7.4 Hz, 1H), 4.11 (s, 1H), 3.59 (s, 2H), 3.13 (t, *J* = 6.3 Hz, 1H), 2.13 (s, 1H), 1.93 – 1.69 (m, 2H), 1.62 1.56 (d, *J* = 25.9 Hz, 12H), 1.46 (d, *J* = 12.7 Hz, 1H), 1.31 – 1.00 (m, 4H), 0.81 (dt, *J* = 12.1, 7.6 Hz, 1H). ^{13}C NMR (100 MHz, $CDCl_3$) 152.9, 143.2, 131.5, 128.2, 124.6, 122.5, 115.5, 80.8, 67.3, 66.2, 45.1, 41.8, 28.6, 28.5, 26.9, 26.6, 26.4, 26.2. HPLC: Daicel CHIRALPAK® IC; detected at 254 nm; *n*-hexane /*i*-propanol = 90/10; flow = 0.5 mL/min; Retention time: 9.762 min (2*R*,3*S*, major), 12.125 min (2*S*,3*R*, minor). HRMS (ESI) calcd for $[C_{20}H_{29}NO_3 + Na]^+$: 354.2040, found: 354.2025.

1.7 Preparative scale synthesis, recycle of camphorsultam and confirmation of the absolute configuration of products 7 and 8.

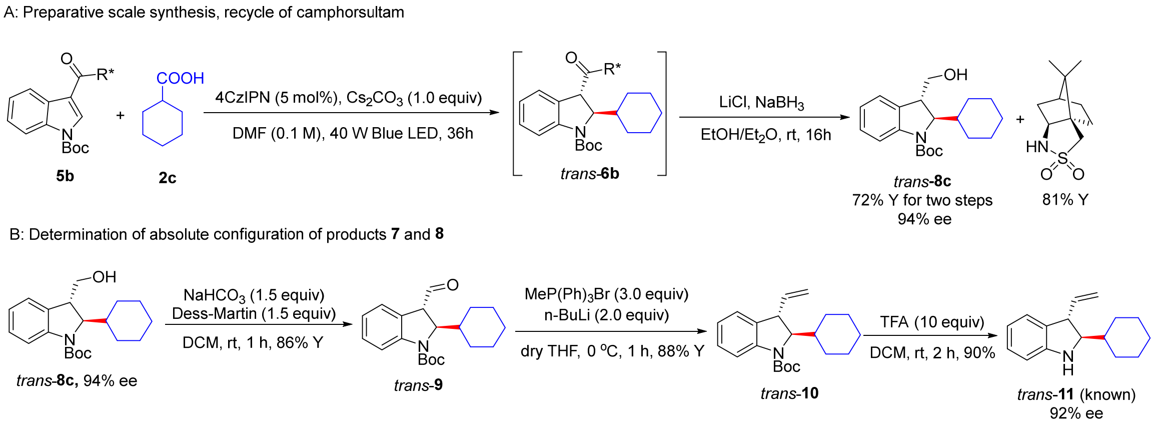

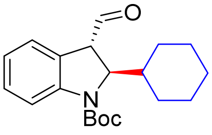

trans-**9**

To a solution of *trans*-**8c** (214 mg, 0.65 mmol, 1.0 equiv) in 10 ml DCM was added $NaHCO_3$ (82 mg, 0.98 mmol, 1.5 equiv) and Dess–Martin periodinane (415 mg, 0.98 mmol, 1.5 equiv). After 1 h of stirring, the solution was quenched by $NaHSO_3$ (saturated, 2 mL) and diluted with 10 ml DCM. The mixture was washed with 5 mL saturated $NaHCO_3$ and dried by Na_2SO_4. The organic solvent was removed under vacuum and purified by flash column chromatography (10% EtOAc in hexane) to get product *trans*-**9** as colorless oil (192 mg, 90% yield). ^1H NMR (400 MHz, $CDCl_3$) δ 9.61 (d, *J* = 1.6 Hz, 1H), 7.71 (s, br, 1H), 7.26 (dd, *J* = 6.0, 5.2 Hz, 2H), 7.00 (t, *J* = 7.4 Hz, 1H), 4.68 (s, 1H), 3.73 (s, 1H), 2.01 (s, 1H), 1.77 (d, *J* = 12.7 Hz, 1H), 1.65 – 1.47 (m, 13H), 1.26 – 1.04 (m, 4H), 0.85 – 0.73 (m, 1H). ^{13}C NMR (100 MHz, $CDCl_3$) 197.2, 152.3, 143.5, 124.9, 122.9, 115.9, 81.5, 63.7, 55.2, 41.2, 28.6, 28.5, 26.7, 26.5, 26.3, 26.1.

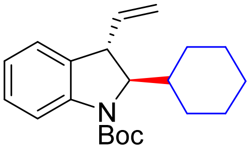

trans-**10**

Under N_2, at – 78 °C to a solution of methyltriphenylphosphonium bromide (535 mg, 1.5 mmol, 3.0 equiv) in 10 mL dry THF was added n-BuLi (400 ul, 2.5 M, 1.0 mmol, 2.0 equiv) dropwise. After 1 h of stirring at 0 °C, the solution of *trans*-**9** (164 mg, 0.5 mmol, 1.0 equiv) in 1 mL dry THF was added to above solution at – 78 °C. After stirring at 0 °C for 1 h, the solution was quenched by NH_4Cl (saturated, 2 mL) and diluted with 10 ml bine. The mixture was extracted by 3 x 10 mL EtOAc and the collected organic layers were dried by Na_2SO_4. The organic solvent was removed under vacuum and purified by flash column chromatography (10% EtOAc in hexane) to get product *trans*-**10** as colorless oil (137mg, 84% yield). ^{1}H NMR (400 MHz, $CDCl_3$) δ 7.65 (s, br, 1H), 7.17 (t, *J* = 7.5 Hz, 1H), 7.08 (d, *J* = 7.3 Hz, 1H), 6.95 (t, *J* = 7.4 Hz, 1H), 5.85 (ddd, *J* = 17.5, 9.9, 8.0 Hz, 1H), 5.00 (dd, *J* = 22.8, 13.5 Hz, 2H), 4.07 (s, 1H), 3.61 (d, *J* = 7.4 Hz, 1H), 1.91 (s, 1H), 1.76 – 1.52 (m, 15H), 1.15 (ddd, *J* = 27.4, 16.2, 7.5 Hz, 4H), 0.97 – 0.79 (m, 2H). ^{13}C NMR (100 MHz, $CDCl_3$) 152.9, 142.7, 140.5, 133.3, 127.9, 125.0, 122.6, 115.5, 114.0, 81.0, 70.1, 46.9, 41.9, 28.7, 28.6, 27.0, 26.9, 26.5, 26.2.

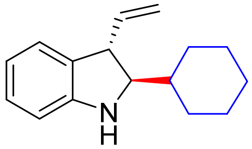

trans-**11**

To a solution of *trans*-**10** (131 mg, 0.4 mmol, 1.0 equiv) in 5 mL DCM was added TFA (300 ul, 4 mmol, 10.0 equiv) dropwise at 0 °C. After stirring at room temperature for 3h, 10 mL water was added to quench reaction. The mixture was diluted with 10 mL DCM and washed by 2 x 5 mL saturated $NaHCO_3$. The collected organic layers were dried by Na_2SO_4. The organic solvent was removed under vacuum and purified by flash column chromatography (20% EtOAc in hexane) to get product *trans*-**11**[10] (82mg, 91% yield, 92% ee). $[\alpha]_D^{24}$ +1322.9 (*c* 1.1, $CHCl_3$). ^{1}H NMR (400 MHz, $CDCl_3$) 7.06 – 6.94 (m, 2H), 6.69 (t, *J* = 7.4 Hz, 1H), 6.60 (d, *J* = 7.7 Hz, 1H), 5.86 (ddd, *J* = 17.2, 9.9, 8.7 Hz, 1H), 5.23 – 5.08 (m, 2H), 3.90 (s, 1H), 3.69 (t, *J* = 8.7 Hz, 1H), 3.42 (dd, *J* = 8.7, 6.4 Hz, 1H), 1.84 – 1.66 (m, 5H), 1.60 – 1.47 (m, 1H), 1.29 – 1.01 (m, 5H). ^{13}C NMR (100 MHz, $CDCl_3$) 150.5, 140.3, 131.3, 127.9, 124.7, 118.5, 115.8, 109.1, 71.2, 51.1, 42.9, 30.3, 29.5, 26.7, 26.4, 16.3. HPLC: Daicel CHIRALPAK® OD-H; detected at 254 nm; *n*-hexane /*i*-propanol = 98/2; flow = 1.0 mL/min; Retention time: 5.005 min (2*R*,3*S*, major), 6.445 min (2*S*,3*R*, minor). HRMS (ESI) calcd for $[C_{16}H_{21}N + H]^+$: 228.1747, found: 228.1736.

1.8 Cyclic voltammetry of 1a

Voltammetric measurements were recorded on a CH Instruments: Model 600E Series Electrochemical Analyzer using a standard three electrodes setup in dry and degassed MeCN (10 mL), with ferrocene as an internal reference ($E_{ox}^{1/2}$ = + 0.40 V vs SCE) and Bu_4NPF_6 as the electrolyte (0.10 mmol). Cyclic voltammograms were recorded at a scan rate of 0.1 V/s.

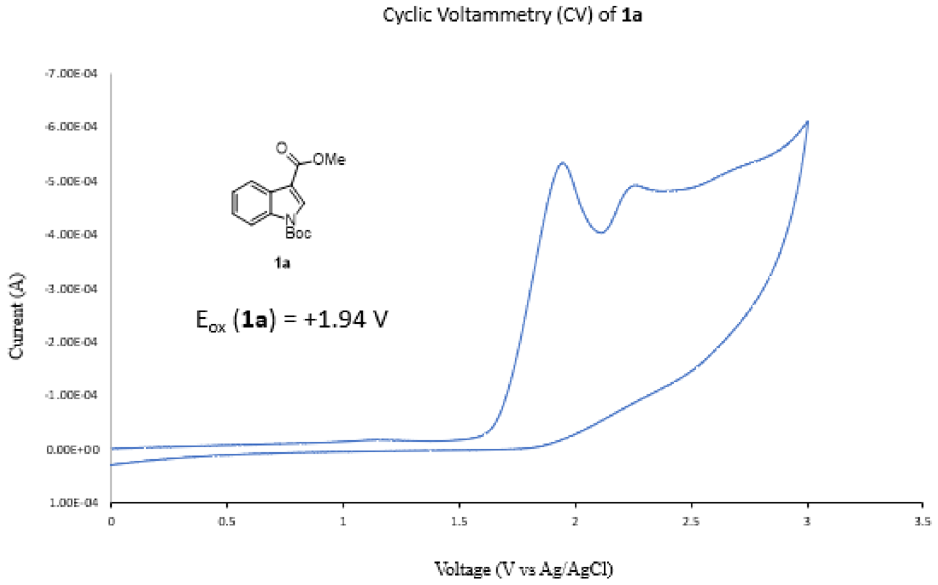

Supplementary Figure 1. Cyclic voltammetry of **1a**

1.9 Mechanism study

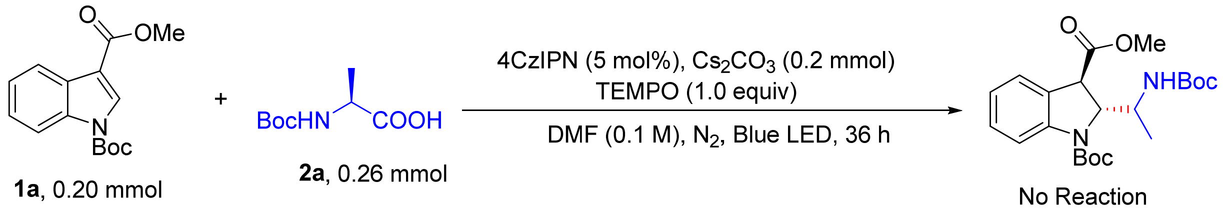

To an oven-dried 10 mL-Schlenk tube equipped with a stir bar, was added **1a** (0.2 mmol, 1.0 equiv), 4CzIPN (8.0 mg, 5 mol%), Boc-Alaine (0.26 mmol, 1.3 equiv), Cs_2CO_3 (65.0 mg, 0.2 mmol, 1.0 equiv), TEMPO (31.2 mg, 1.0 equiv) and DMF (2.0 ml). The reaction mixture was degassed by freeze-pump-thaw method, then sealed with parafilm. The solution was then stirred at room temperature under the irradiation of a 5w blue LED strip for 36h.

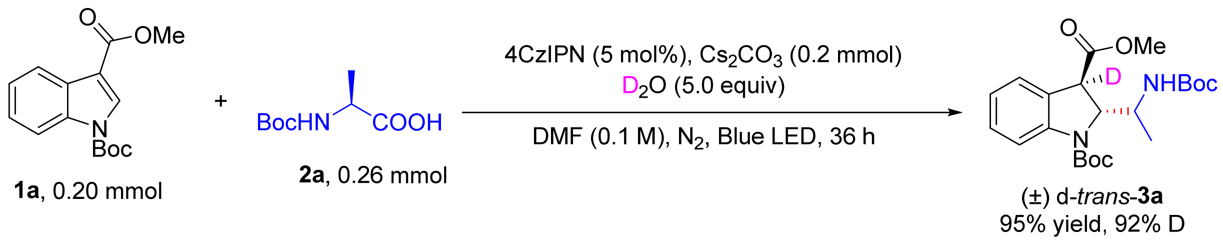

To an oven-dried 10 mL-Schlenk tube equipped with a stir bar, was added **1a** (0.2 mmol, 1.0 equiv), 4CzIPN (8.0 mg, 5 mol%), Boc-Alaine (0.26 mmol, 1.3 equiv), Cs_2CO_3 (65.0 mg, 0.2 mmol, 1.0 equiv), 20 µl D_2O and anhydrous MeCN (1.0 mL). The mixture was stirred at room temperature for 10 min. After MeCN was removed under vacuum, anhydrous DMF (2.0 ml) and 20 µl D_2O was added to tube. The reaction mixture was degassed by freeze-pump-thaw method, then sealed with parafilm. The solution was then stirred at room temperature under the irradiation of a 5w blue LED strip for 36h. After completion of the reaction, the mixture was diluted with water and extracted by EtOAc. The organic layer was collected, dried by Na_2SO_4 and concentrated under vacuum. The residue was purified by flash column chromatography to afford product (±) d-*trans*-**3a** (95% yield, 92% deuteration ratio, dr = 1.1/1). HRMS (ESI) calcd for $[C_{22}H_{31}DN_2O_6 + H]^+$: 444.2215, found: 444.2213.

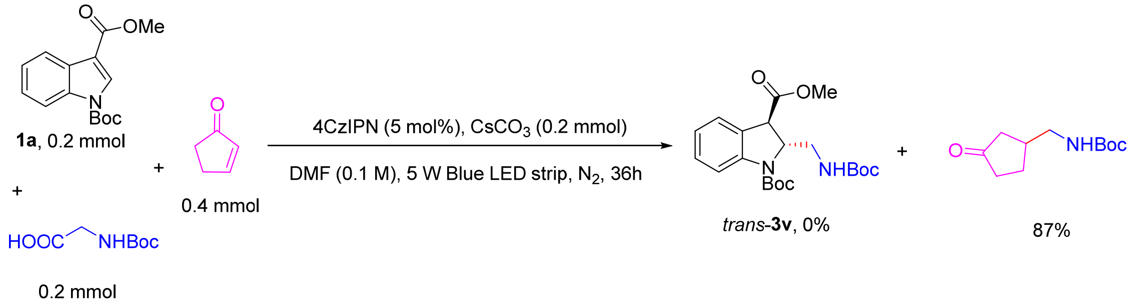

To an oven-dried 10 mL-Schlenk tube equipped with a stir bar, was added **1a** (0.2 mmol, 1.0 equiv), 4CzIPN (8.0 mg, 5 mol%), Boc-Glycine (0.20 mmol, 1.0 equiv), cyclopent-2-en-1-one (0.4 mmol, 33.5 µL, 2.0 equiv), Cs_2CO_3 (65.0 mg, 0.2 mmol, 1.0 equiv) and DMF (2.0 ml). The reaction mixture was degassed by freeze-pump-thaw method, then sealed with parafilm. The solution was then stirred at room temperature under the irradiation of a 5w blue LED strip for 36h. Only byproduct *tert*-butyl ((3-oxocyclopentyl)methyl)carbamate was formed with 37 mg (87% yield). ^1H NMR (400 MHz, $CDCl_3$) δ 4.68 (s, 1H), 3.19 (s, 2H), 2.42 – 2.26 (m, 3H), 2.22 – 2.11 (m, 2H), 1.90 (dd, J = 20.1, 11.5 Hz, 1H), 1.67 – 1.54 (m, 1H), 1.43 (s, 9H).

1.10 Crystal data for *trans*-3k.

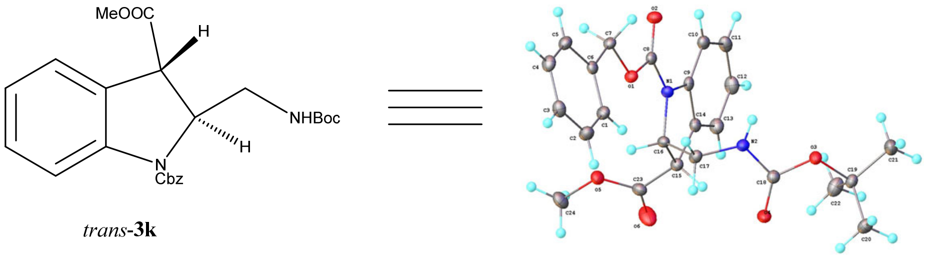

Supplementary Figure 2. Crystal structure of *trans*-**3k**

Supplementary Table 1. Crystal data and structure refinement for compound *trans*-**3k**.

Identification code	mo_YZ001_0m
Empirical formula	$C_{24}H_{28}N_2O_6$
Formula weight	440.48
Temperature/K	100
Crystal system	triclinic
Space group	P-1
a/Å	9.348(3)
b/Å	9.557(3)
c/Å	14.427(4)
α/°	72.333(9)
β/°	77.148(9)
γ/°	63.908(8)
Volume/Å^3	1097.0(5)
Z	2
ρ_{calc}g/cm^3	1.334
μ/mm^{-1}	0.096
F(000)	468.0
Crystal size/mm^3	0.31 × 0.15 × 0.08
Radiation	MoKα (λ = 0.71073)
2Θ range for data collection/°	4.878 to 53.718
Index ranges	$-11 \le h \le 11$, $-12 \le k \le 12$, $-18 \le l \le 18$
Reflections collected	47843
Independent reflections	4715 [R_{int} = 0.0238, R_{sigma} = 0.0117]
Data/restraints/parameters	4715/0/297
Goodness-of-fit on F^2	1.009
Final R indexes [I>=2σ (I)]	R_1 = 0.0318, wR_2 = 0.0812

Final R indexes [all data]	R_1 = 0.0353, wR_2 = 0.0844
Largest diff. peak/hole / e Å^{-3}	0.32/-0.20

Supplementary Table 2. Crystal data and structure refinement for compound *trans*-**3k**.

Identification code	mo_YZ001_0m
Empirical formula	$C_{24}H_{28}N_2O_6$
Formula weight	440.48
Temperature/K	100
Crystal system	triclinic
Space group	P-1
a/Å	9.348(3)
b/Å	9.557(3)
c/Å	14.427(4)
α/°	72.333(9)
β/°	77.148(9)
γ/°	63.908(8)
Volume/Å^3	1097.0(5)
Z	2
ρ_{calc}g/cm^3	1.334
μ/mm^{-1}	0.096
F(000)	468.0
Crystal size/mm^3	0.31 × 0.15 × 0.08
Radiation	MoKα (λ = 0.71073)
2Θ range for data collection/°	4.878 to 53.718
Index ranges	$-11 \leq h \leq 11$, $-12 \leq k \leq 12$, $-18 \leq l \leq 18$
Reflections collected	47843
Independent reflections	4715 [R_{int} = 0.0238, R_{sigma} = 0.0117]
Data/restraints/parameters	4715/0/297
Goodness-of-fit on F^2	1.009
Final R indexes [I>=2σ (I)]	R_1 = 0.0318, wR_2 = 0.0812

Final R indexes [all data]	R_1 = 0.0353, wR_2 = 0.0844
Largest diff. peak/hole / e Å^{-3}	0.32/-0.20

Molecular structure. The displacement ellipsoids are at 50% probability level; the hydrogen atoms are at predicted positions except the hydrogen at N2, which was refined explicitly.

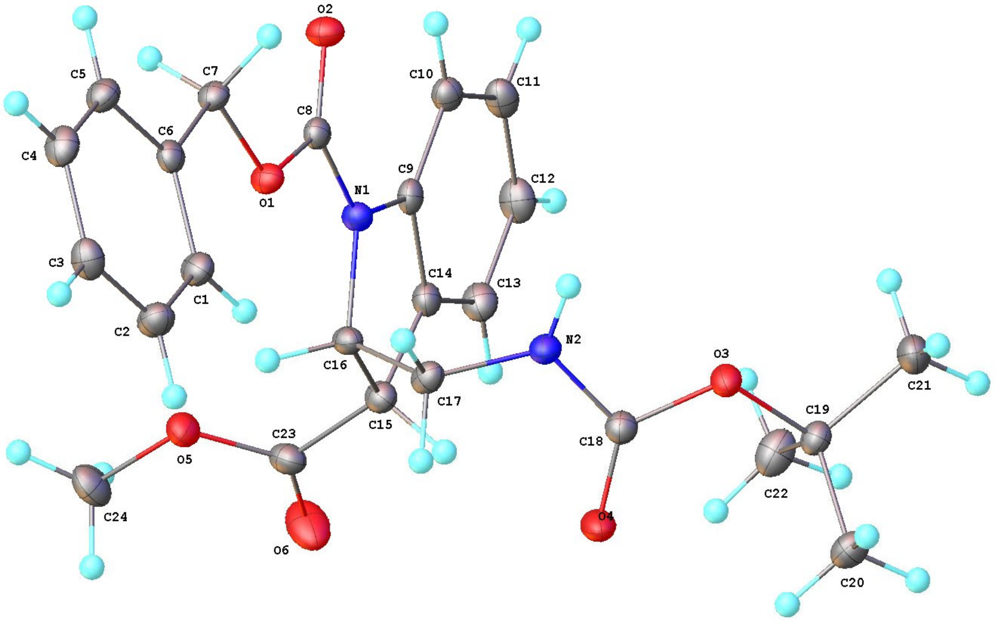

H-bonding. There are 2 molecules per unit cell, and they are connected by the H-bonds between N2 and O2.

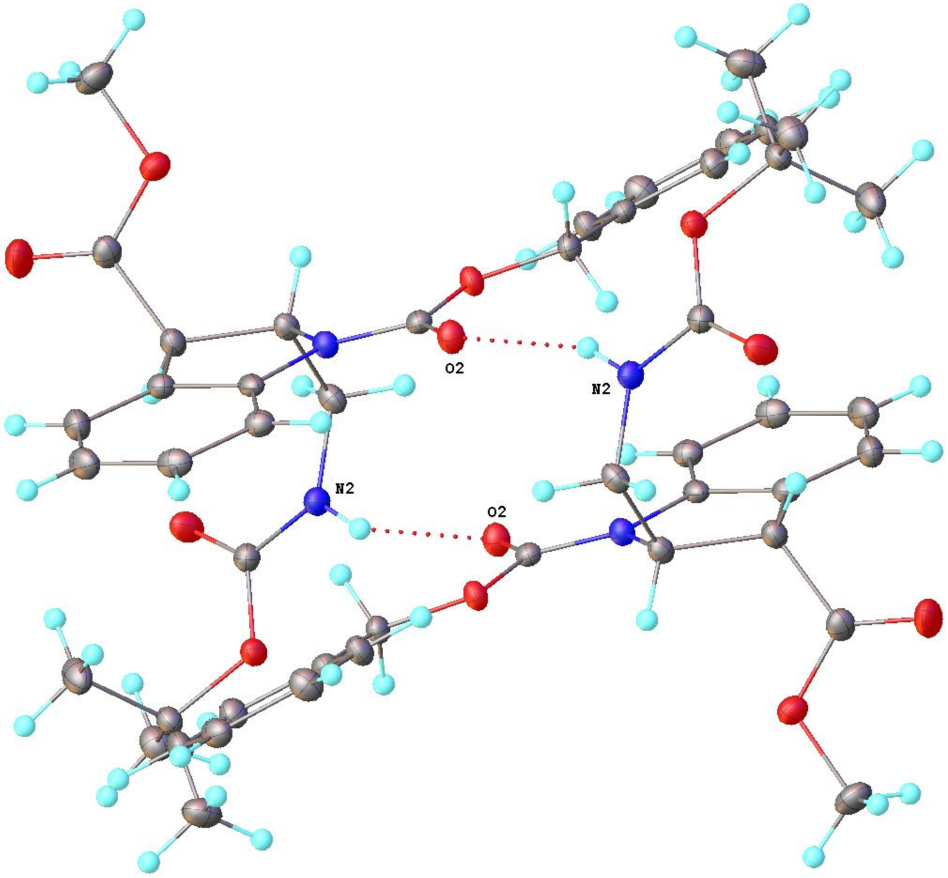

Unit cell and packing. View almost along axis b.

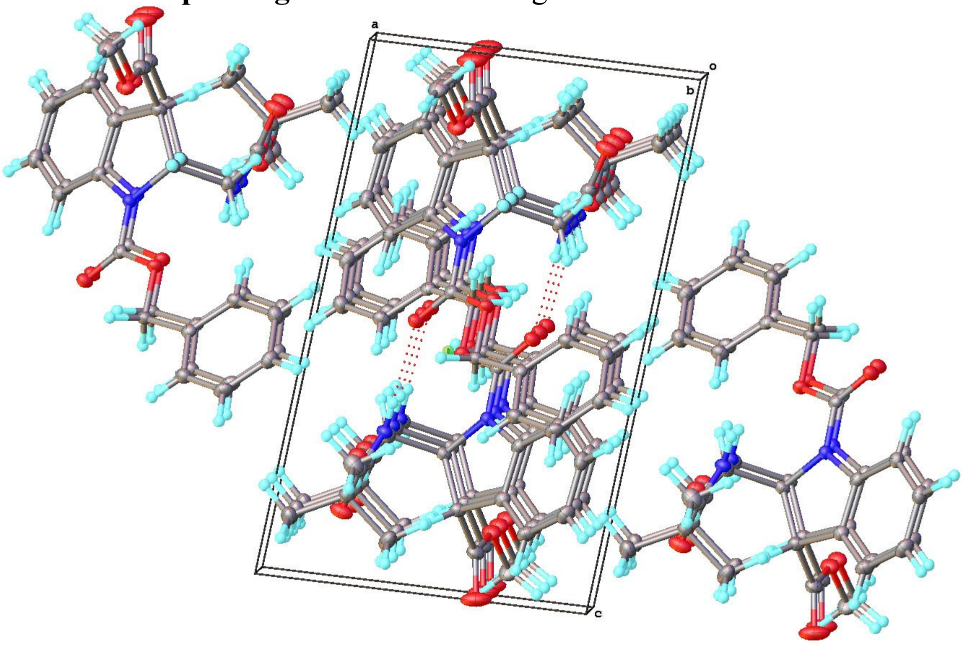

Supplementary Table 3. Fractional Atomic Coordinates (×10^4) and Equivalent Isotropic Displacement Parameters (Å^2×10^3) for compound *trans*-**3k**. U_{eq} is defined as 1/3 of of the trace of the orthogonalised U_{IJ} tensor.

**Atom**	***x***	***y***	***z***	**U(eq)**
O1	5240.2(8)	7062.2(8)	5485.1(5)	15.24(15)
O2	3258.9(9)	6697.6(9)	5061.3(5)	18.03(16)
O3	7545.8(9)	36.3(9)	7468.9(5)	17.48(16)
O5	3249.5(9)	7157.0(9)	8685.3(5)	20.07(17)
O4	7695.6(10)	1273.9(9)	8549.2(6)	24.15(18)
N2	6951.4(10)	2639.2(10)	7023.3(6)	15.98(18)
N1	4061.7(10)	5591.5(10)	6597.3(6)	13.85(17)
O6	3246.0(13)	5053.8(11)	9910.9(6)	35.9(2)
C9	2962.5(12)	4862.5(11)	7014.2(7)	14.3(2)
C8	4112.3(11)	6471.1(11)	5661.7(7)	13.87(19)
C6	6679.7(12)	8578.9(12)	4399.6(7)	14.6(2)
C16	5111.0(12)	5313.7(12)	7330.6(7)	14.5(2)
C18	7425.9(12)	1309.0(12)	7754.4(7)	16.2(2)
C14	3083.8(12)	4251.4(12)	8013.4(7)	15.9(2)
C17	6835.9(12)	4164.5(12)	7094.4(7)	16.4(2)
C23	3564.5(13)	5607.8(13)	9065.0(8)	19.1(2)
C7	5281.5(12)	8127.3(12)	4531.0(7)	14.9(2)
C1	7849.4(13)	7847.2(12)	5041.8(8)	18.1(2)
C10	1916.5(12)	4675.9(12)	6559.9(8)	16.7(2)
C5	6818.6(13)	9762.6(12)	3581.4(8)	18.5(2)
C15	4332.4(12)	4591.0(12)	8314.7(7)	16.2(2)
C11	995.5(12)	3845.9(13)	7138.5(8)	19.7(2)
C3	9244.7(13)	9495.5(13)	4066.7(8)	19.8(2)
C19	7600.5(12)	-1433.2(12)	8216.1(7)	16.2(2)
C2	9124.3(13)	8307.5(13)	4877.6(8)	21.2(2)
C20	9148.4(13)	-2241.3(13)	8695.7(8)	21.9(2)
C13	2150.2(13)	3444.7(13)	8581.0(8)	20.4(2)
C4	8091.5(13)	10216.9(13)	3415.6(8)	20.5(2)
C12	1096.8(13)	3244.4(13)	8136.7(8)	22.2(2)
C21	7570.7(15)	-2479.7(13)	7621.6(9)	25.3(2)
C22	6112.0(14)	-1018.0(14)	8944.8(9)	25.2(2)
C24	2493.6(15)	8175.4(15)	9374.5(9)	25.8(2)

1.11 References

(1) Huang, H.; Yu, C.; Zhang, Y.; Zhang, Y.; Mariano, P. S.; Wang, W. Chemo- and Regioselective Organo-Photoredox Catalyzed Hydroformylation of Styrenes via a Radical Pathway. *J. Am. Chem. Soc.*, **139**, 9799-9802 (2017**)**.

(2) Chen, L.; Shen, J. J.; Gao, Q.; Xu, S. Synthesis of Cyclic Chiral Alpha-amino Boronates by Copper-catalyzed Asymmetric Dearomative Borylation of Indoles. *Chem. Sci.*, **9**, 5855-5859 (**2018)**.

(3) Zhang, Y.; Ji, P.; Hu, W.; Wei, Y.; Huang, H.; Wang, W. Organocatalytic Transformation of Aldehydes to Thioesters with Visible Light. *Chem.–Eur. J.*, **25**, 8225-8228 (2019).

(4) Wang, L.; Shao, Y.; Liu, Y. Nucleophilic Addition of Grignard Reagents to 3-Acylindoles: Stereoselective Synthesis of Highly Substituted Indoline Scaffolds. *Org. Lett.*, **14**, 3978-3981 (2012**)**.

(5) Mambrini, A.; Gori, D.; Guillot, R.; Kouklovsky, C.; Alezra, V. Oxidative Coupling of Enolates Using Memory of Chirality: An Original Enantioselective Synthesis of Quaternary Alpha-amino Acid Derivatives. *Chem. Commun.*, **54**, 12742-12745 (2018**)**.

(6) Liu, Y.; Hu, X.; Wu, Y.; Zhang, W.; Chen, X.; You, X.; Hu, L. Synthesis and Structure-activity Relationship of Novel Bisindole Amidines Active Against MDR Gram-positive and Gram-negative Bacteria. *Eur. J. Med. Chem.*, **150**, 771-782 (2018**)**.

(7) Kuwano, R.; Kashiwabara, M. Ruthenium-catalyzed Asymmetric Hydrogenation of *N*-boc-indoles. *Org. Lett.*, **8**, 2653-2655 (2006**)**.

(8) Kubota, K.; Hayama, K.; Iwamoto, H.; Ito, H. Enantioselective Borylative Dearomatization of Indoles through Copper(I) Catalysis. *Angew. Chem., Int. Ed.*, **54**, 8809-8813 (2015**)**.

(9) Xu, L.; Liu, F.; Xu, L. W.; Gao, Z.; Zhao, Y. M. A Total Synthesis of Paeoveitol. *Org. Lett.*, **18**, 3698-3701 (2016**)**.
