# Supplementary material for: Organophotocatalytic dearomatization of indoles, pyrroles and benzo(thio)furans via a Giese-type transformation
Source: Commun Chem. 2021 Feb 19;4:20. doi: 10.1038/s42004-021-00460-y (PMC9814947; doi:10.1038/s42004-021-00460-y)
Supplement: Supplementary file 3 — Description of Additional Supplementary Files [file 42004_2021_460_MOESM3_ESM.pdf]

Description of Additional Supplementary Files

File Name: Supplementary Data 1
Description: Original ^{1}H and ^{13}C NMR Spectra.

File Name: Supplementary Data 2
Description: Chiral HPLC analysis trace.
