# Supplementary material for: Organophotocatalytic dearomatization of indoles, pyrroles and benzo(thio)furans via a Giese-type transformation
Source: Commun Chem. 2021 Feb 19;4:20. doi: 10.1038/s42004-021-00460-y (PMC9814947; doi:10.1038/s42004-021-00460-y)
Supplement: Supplementary file 4 — Supplementary Data 1 [file 42004_2021_460_MOESM4_ESM.pdf]

Supplementary Data 1: ^{1}H and ^{13}C NMR Spectra

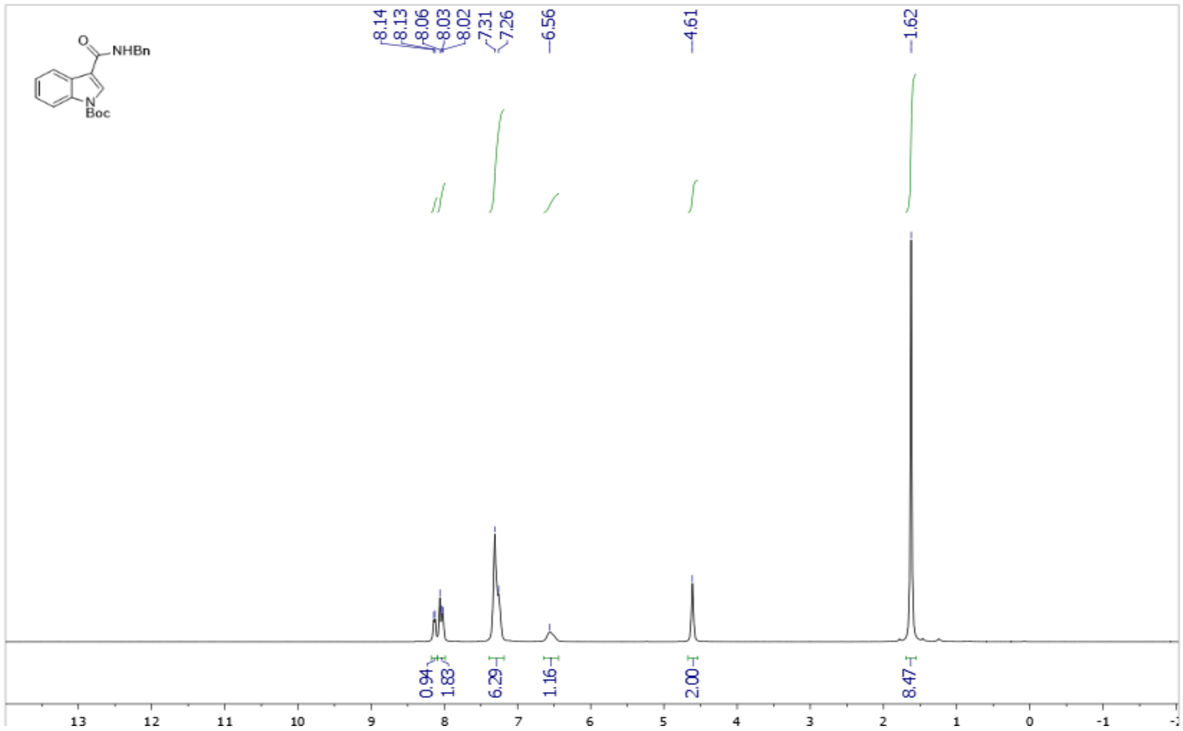

Supplementary Figure 3. ^{1}H NMR spectra of **1c**

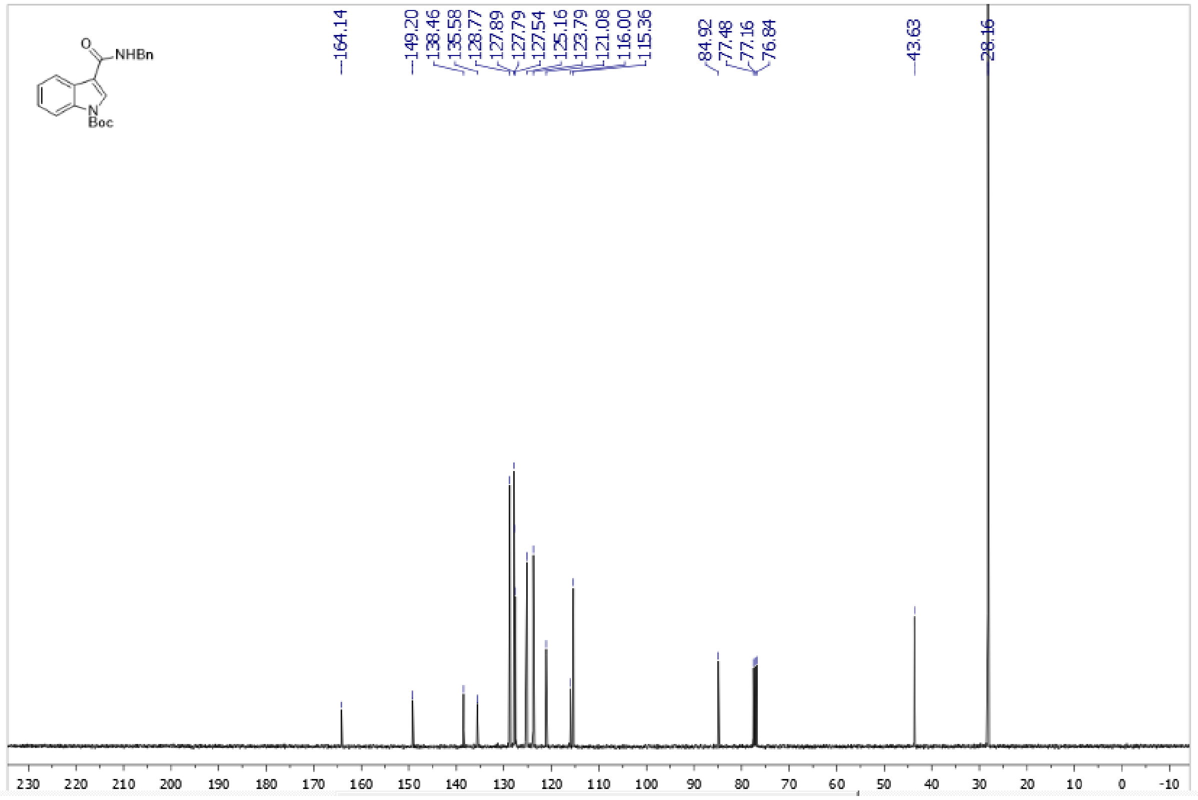

Supplementary Figure 4. ^{13}C NMR spectra of **1c**

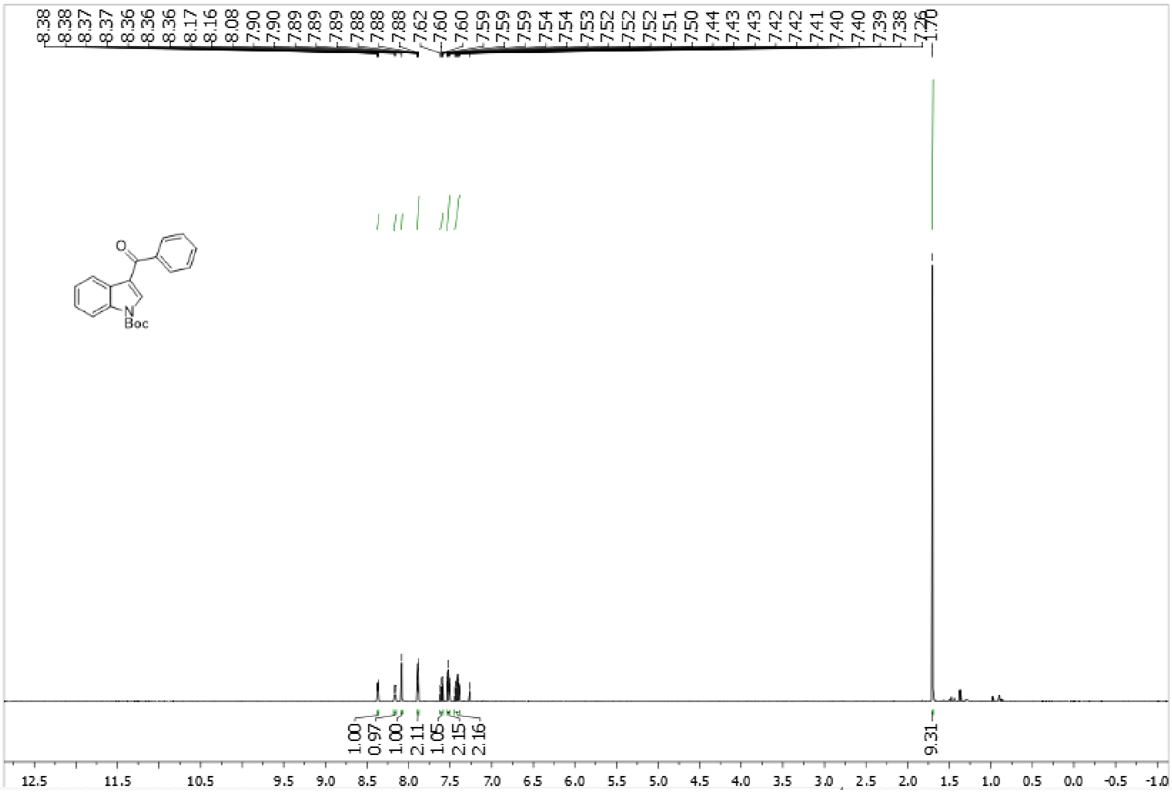

Supplementary Figure 5. ^{1}H NMR spectra of **1e**

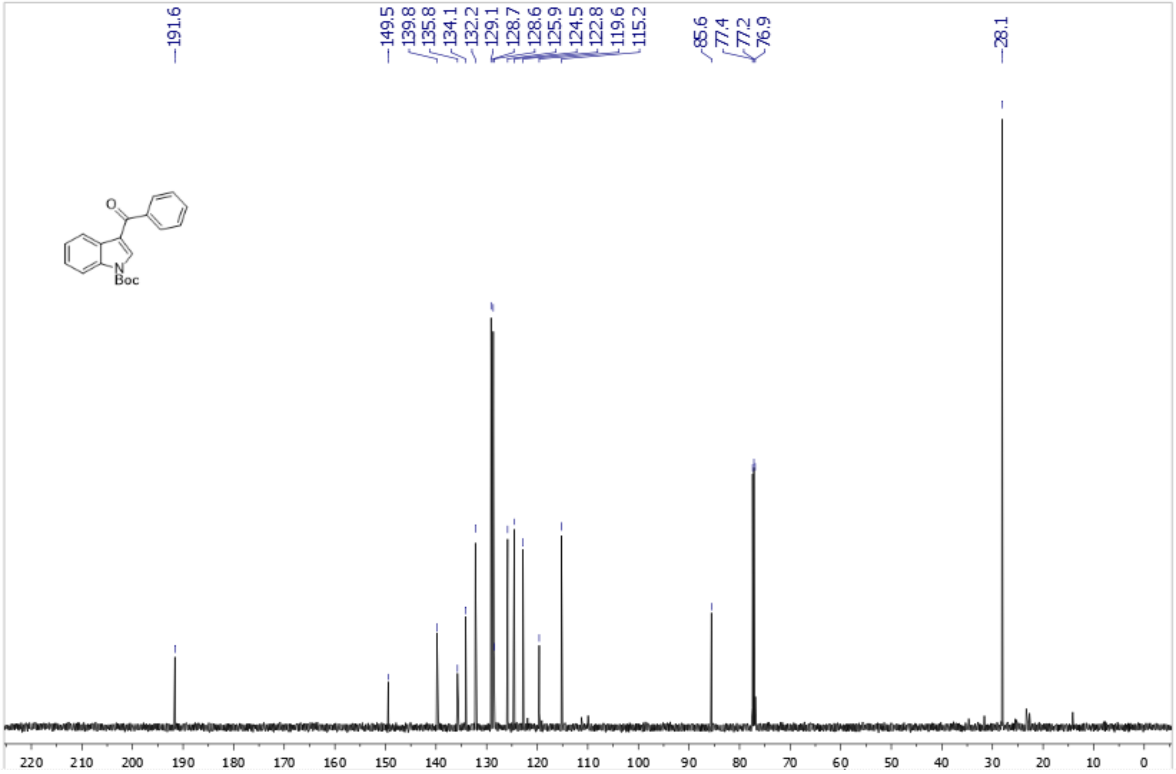

Supplementary Figure 6. ^{13}C NMR spectra of **1e**

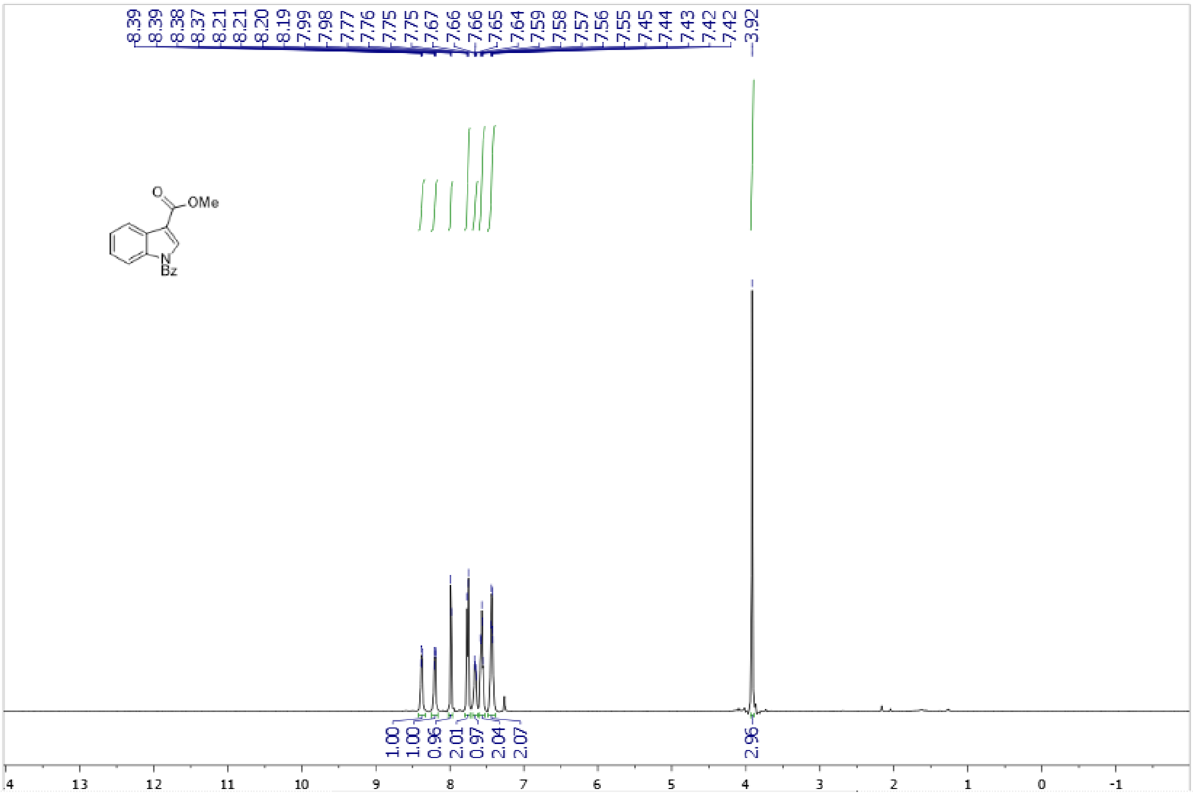

Supplementary Figure 7. ^{1}H NMR spectra of **1i**

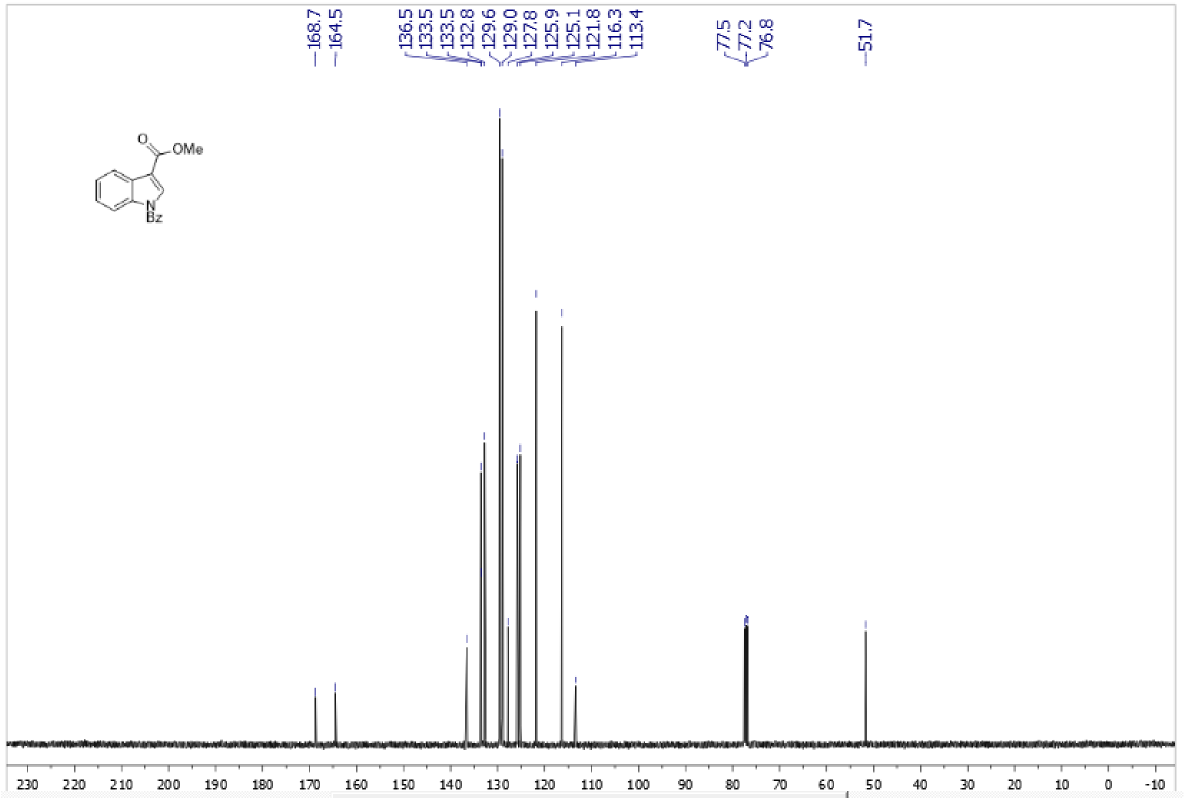

Supplementary Figure 8. ^{13}C NMR spectra of **1i**

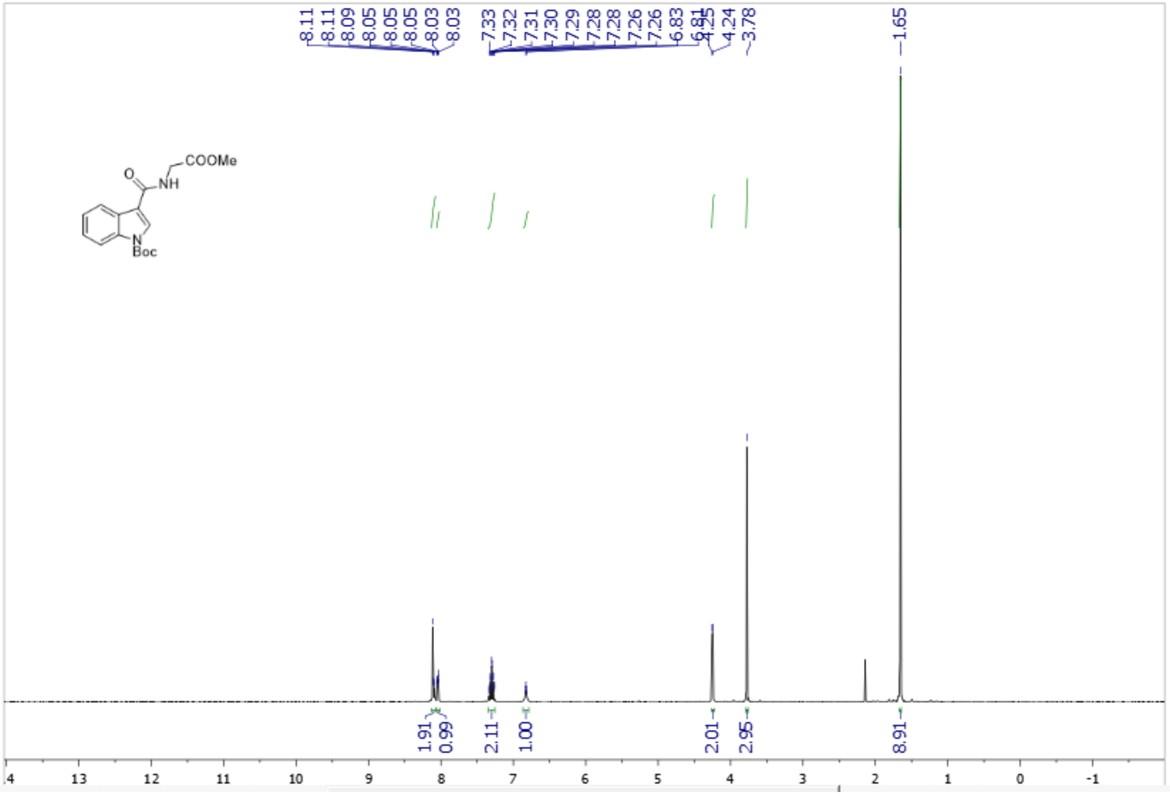

Supplementary Figure 9. ^{1}H NMR spectra of **1g**

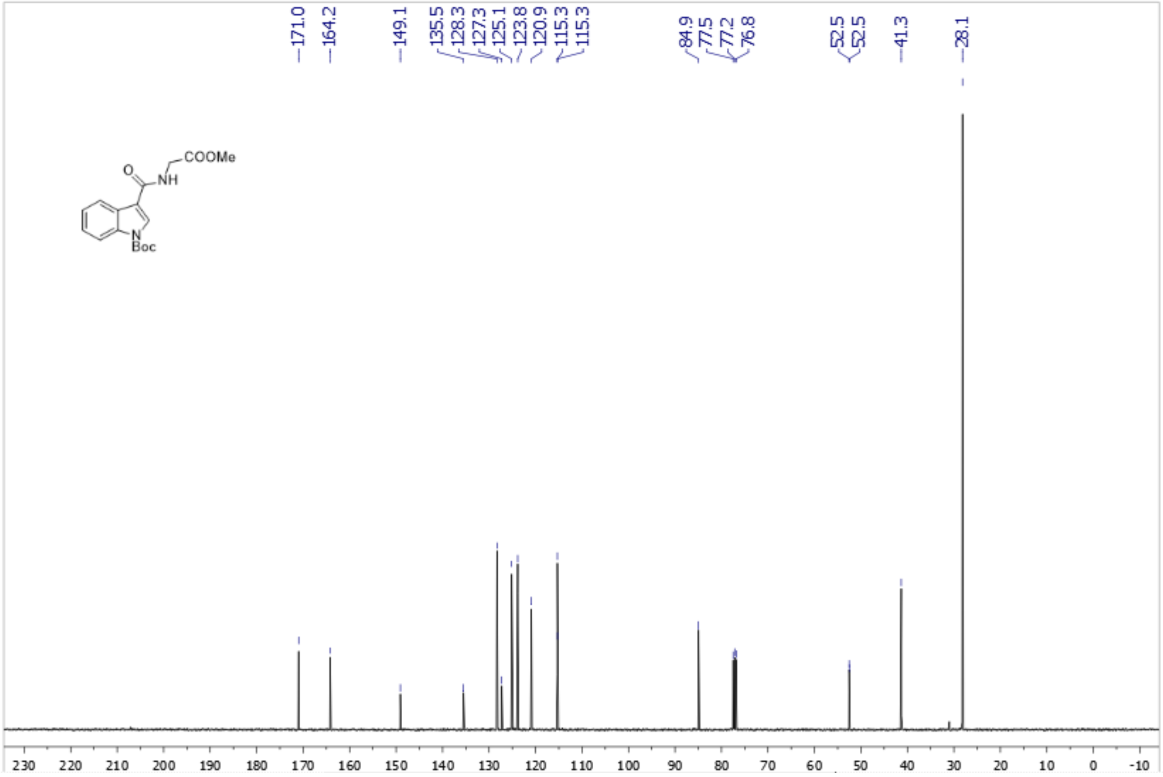

Supplementary Figure 10. ^{13}C NMR spectra of **1g**

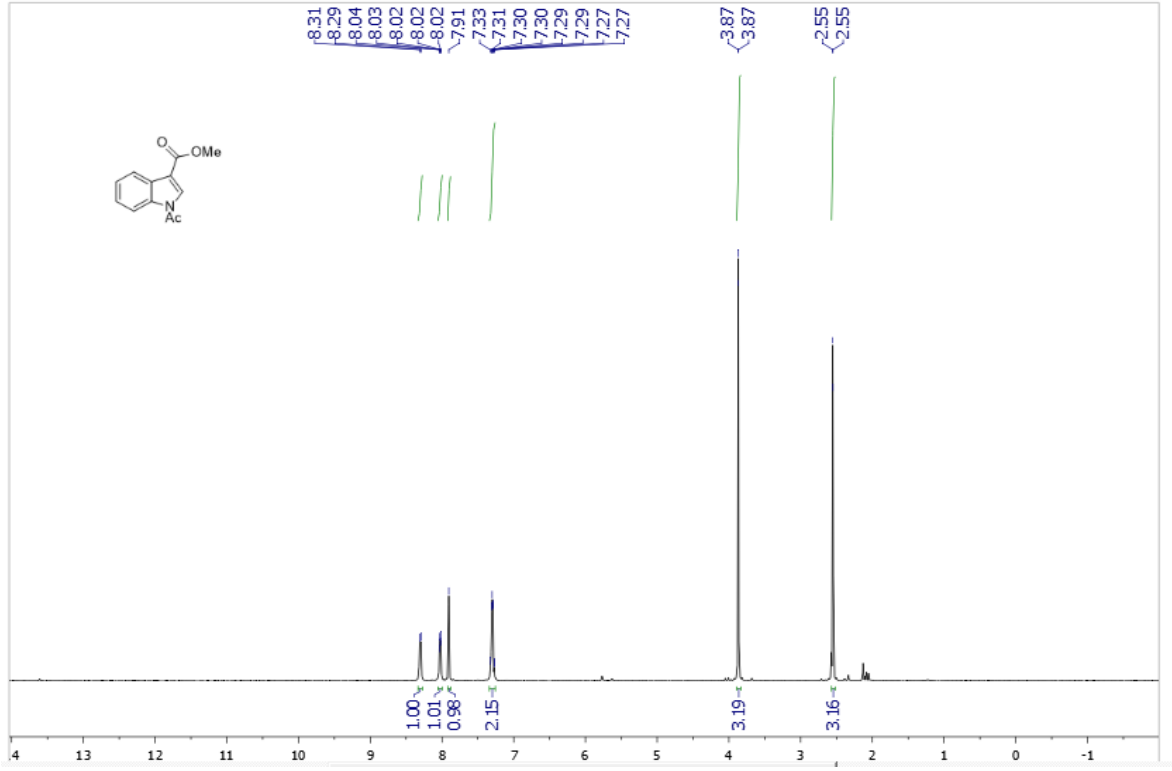

Supplementary Figure 11. ^{1}H NMR spectra of **1j**

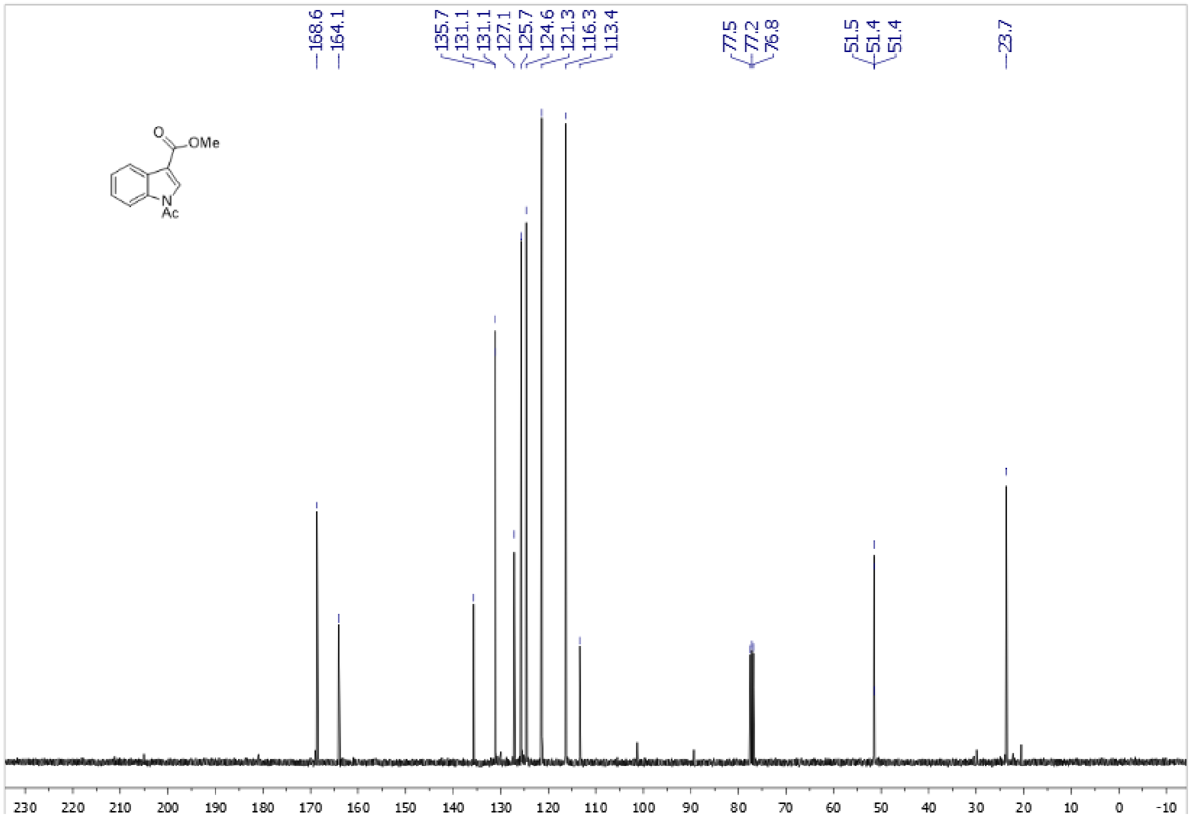

Supplementary Figure 12. ^{13}C NMR spectra of **1j**

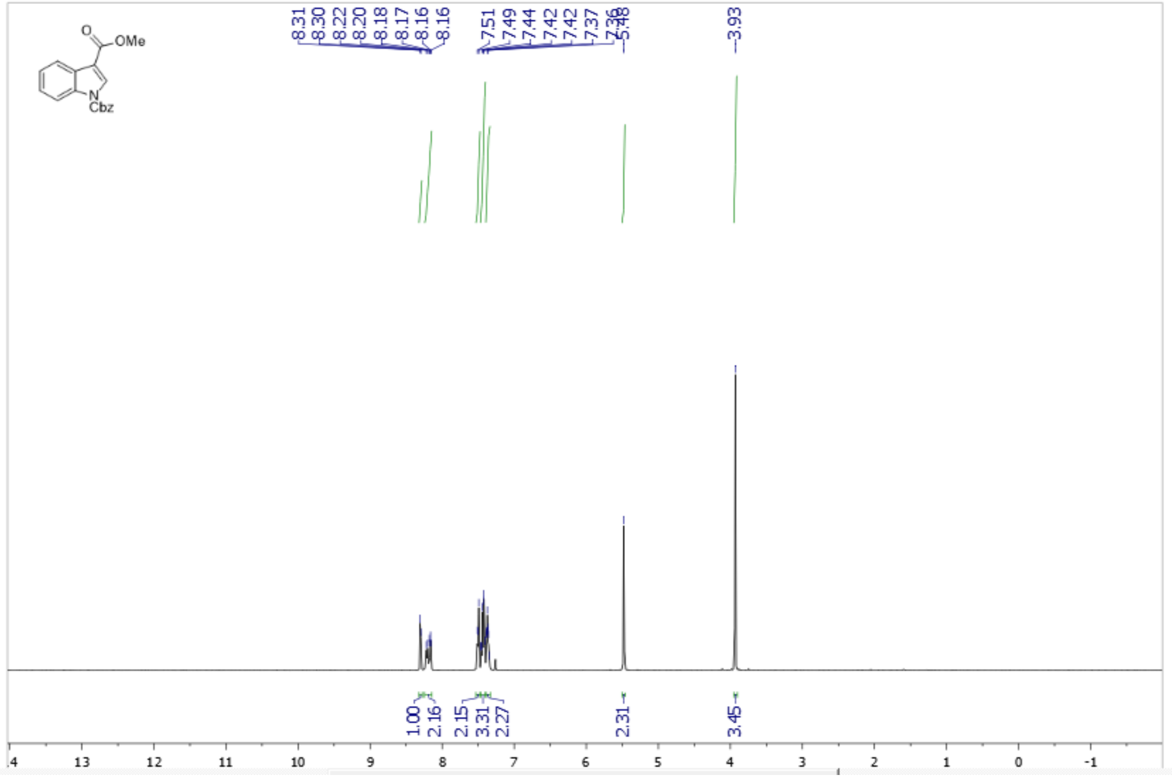

Supplementary Figure 13. ^{1}H NMR spectra of **1k**

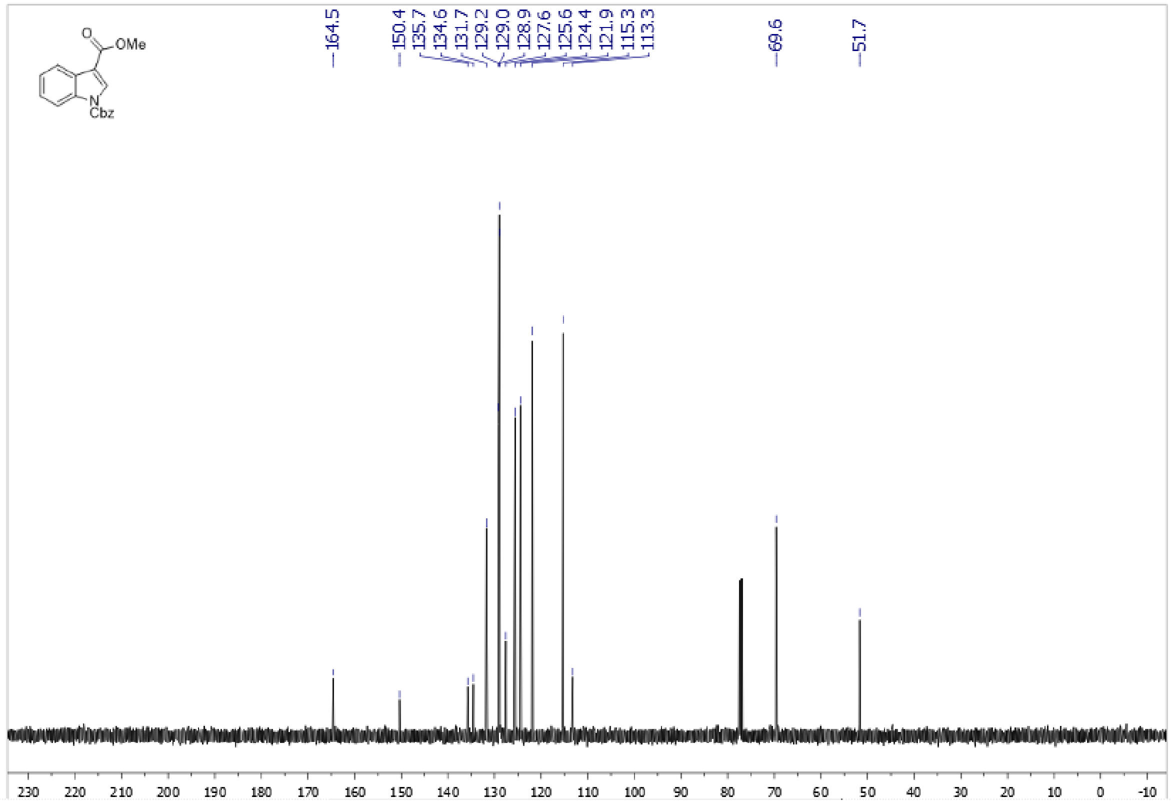

Supplementary Figure 14. ^{13}C NMR spectra of **1k**

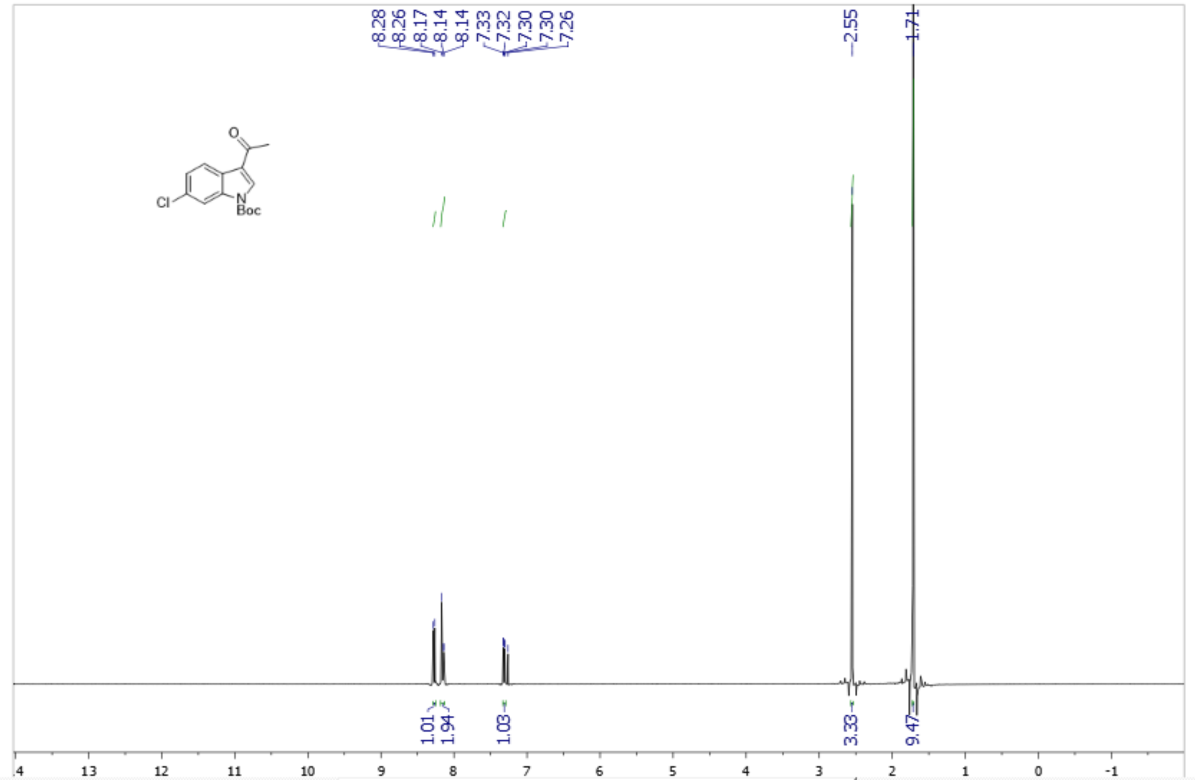

Supplementary Figure 15. ^{1}H NMR spectra of **1l**

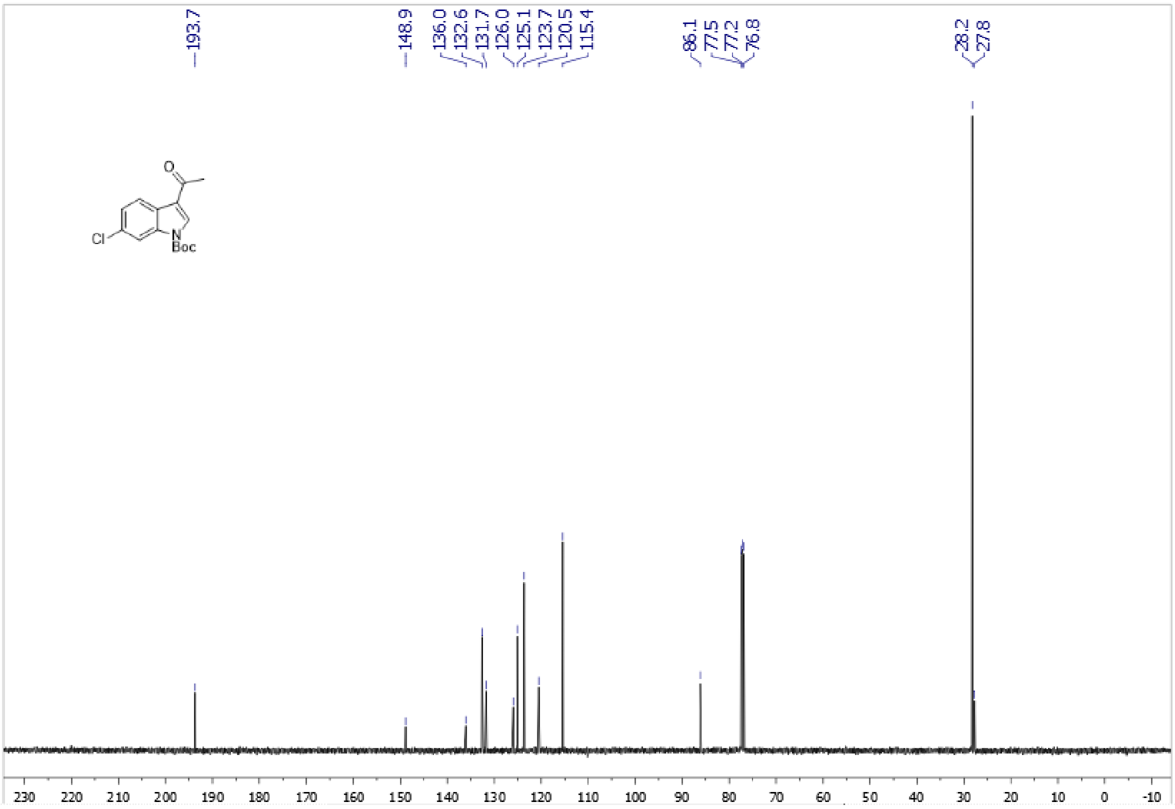

Supplementary Figure 16. ^{13}C NMR spectra of **1l**

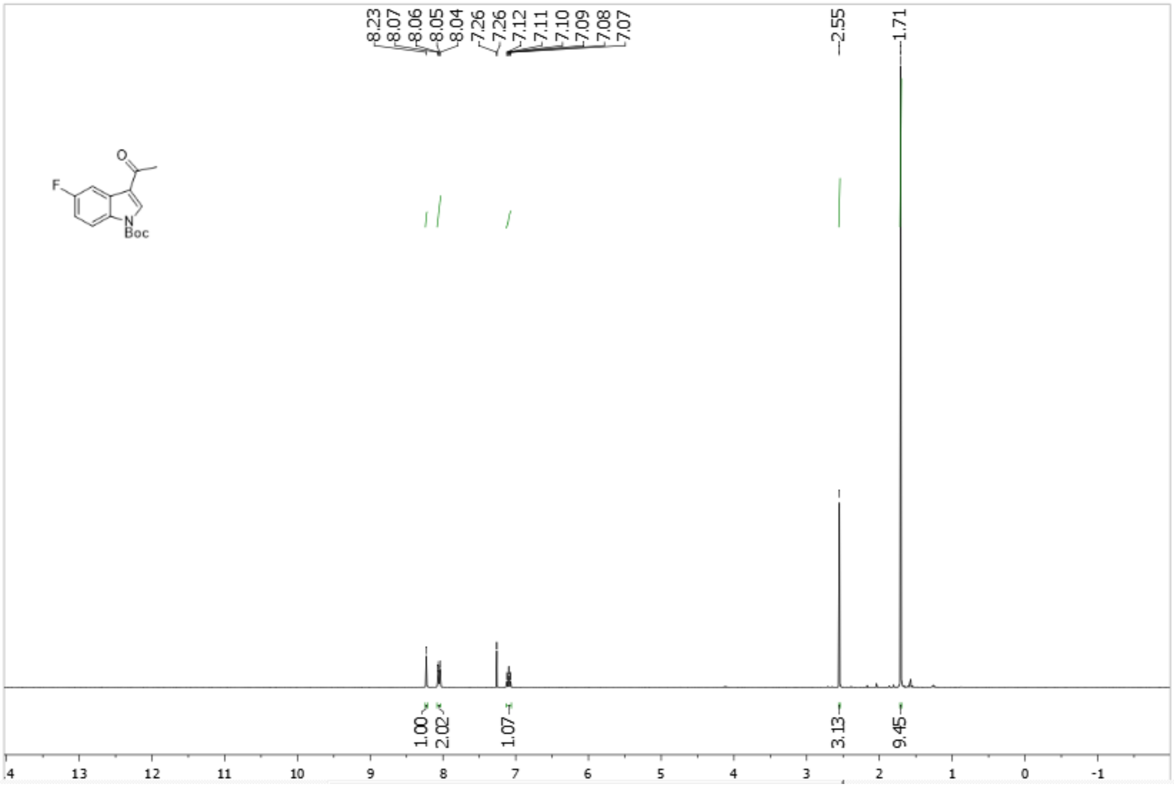

Supplementary Figure 17. ^{1}H NMR spectra of **1m**

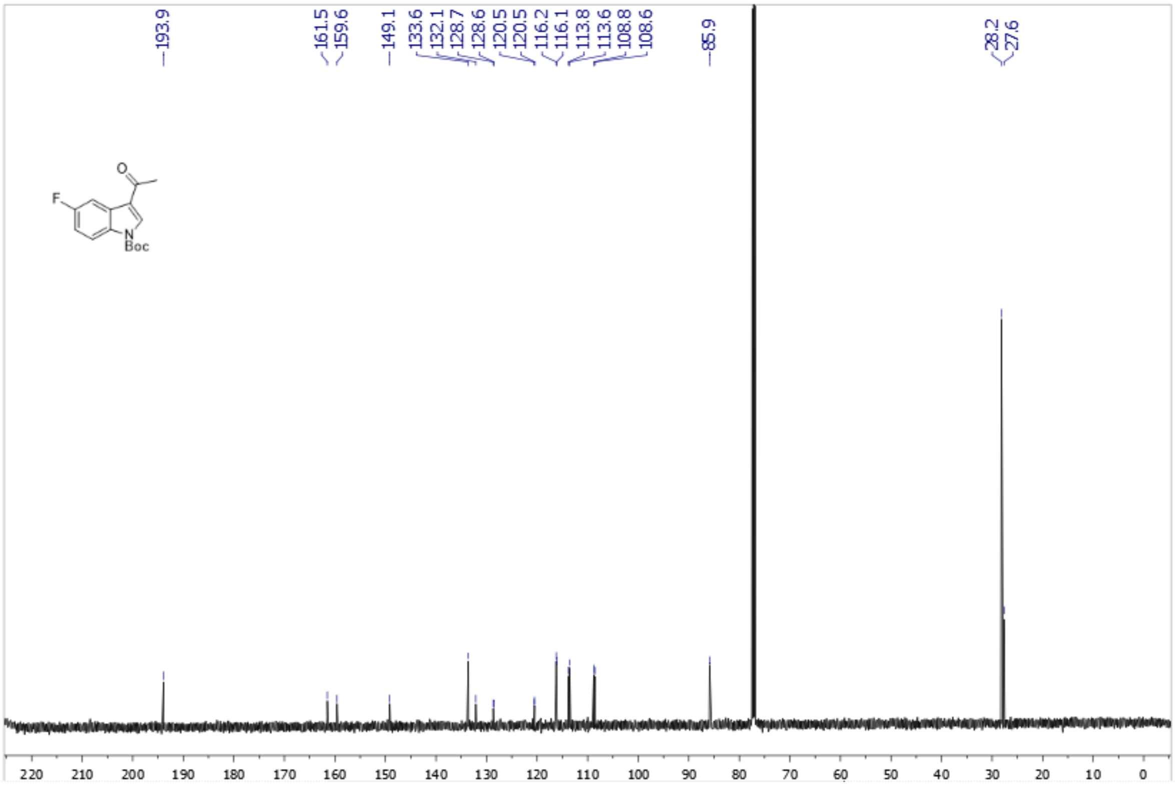

Supplementary Figure 18. ^{13}C NMR spectra of **1m**

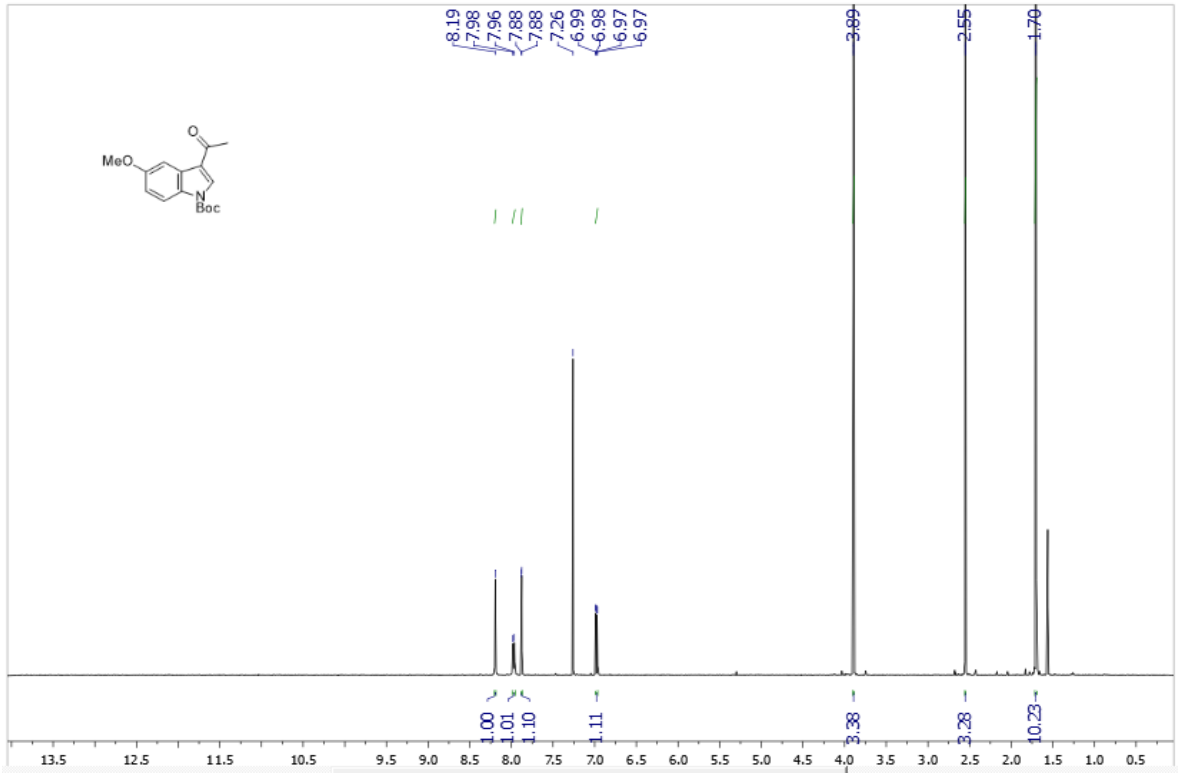

Supplementary Figure 19. ^{1}H NMR spectra of **1n**

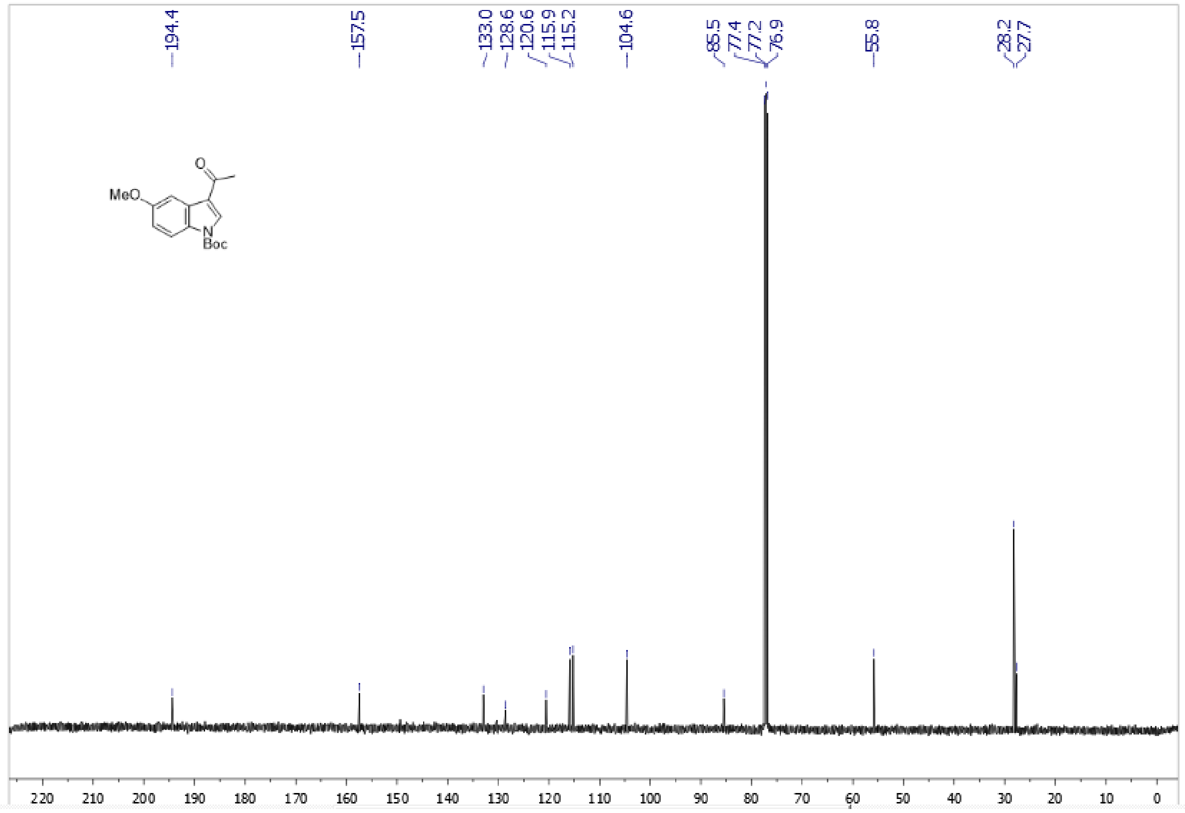

Supplementary Figure 20. ^{13}C NMR spectra of **1n**

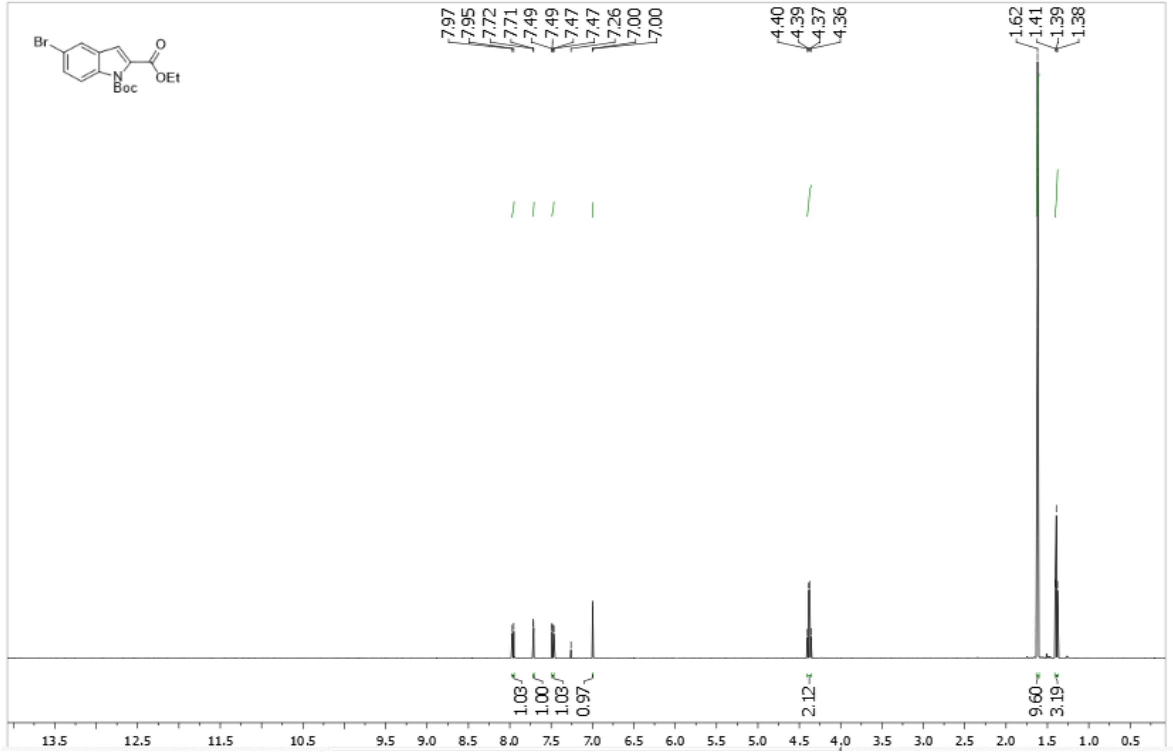

Supplementary Figure 21. ^{1}H NMR spectra of **1r**

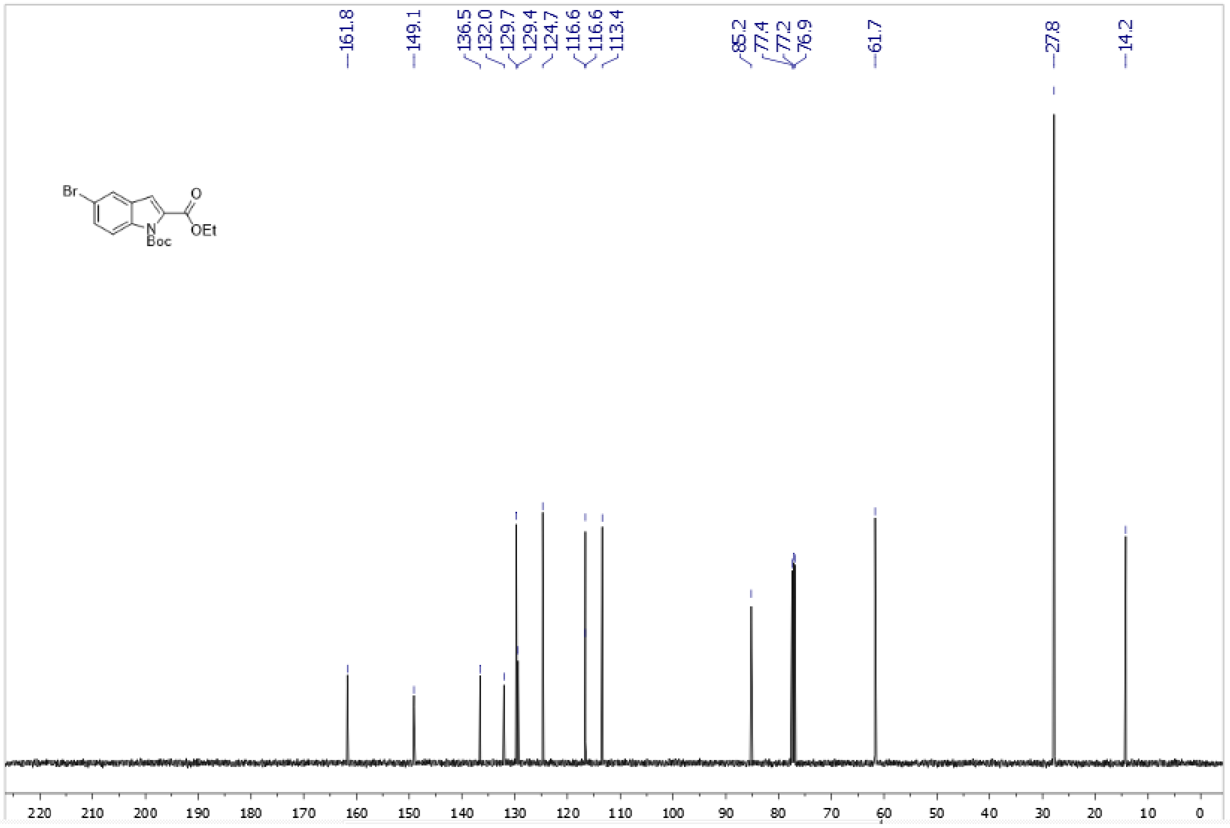

Supplementary Figure 22. ^{13}C NMR spectra of **1r**

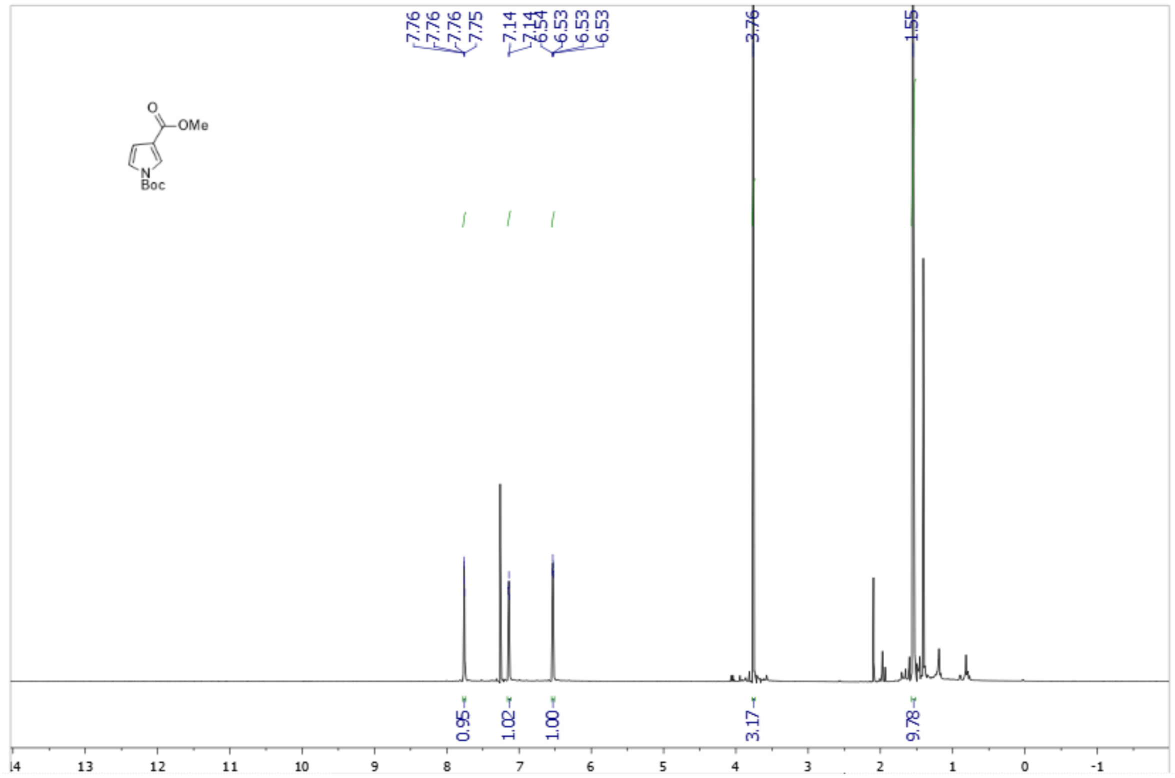

Supplementary Figure 23. ^{1}H NMR spectra of **1s**

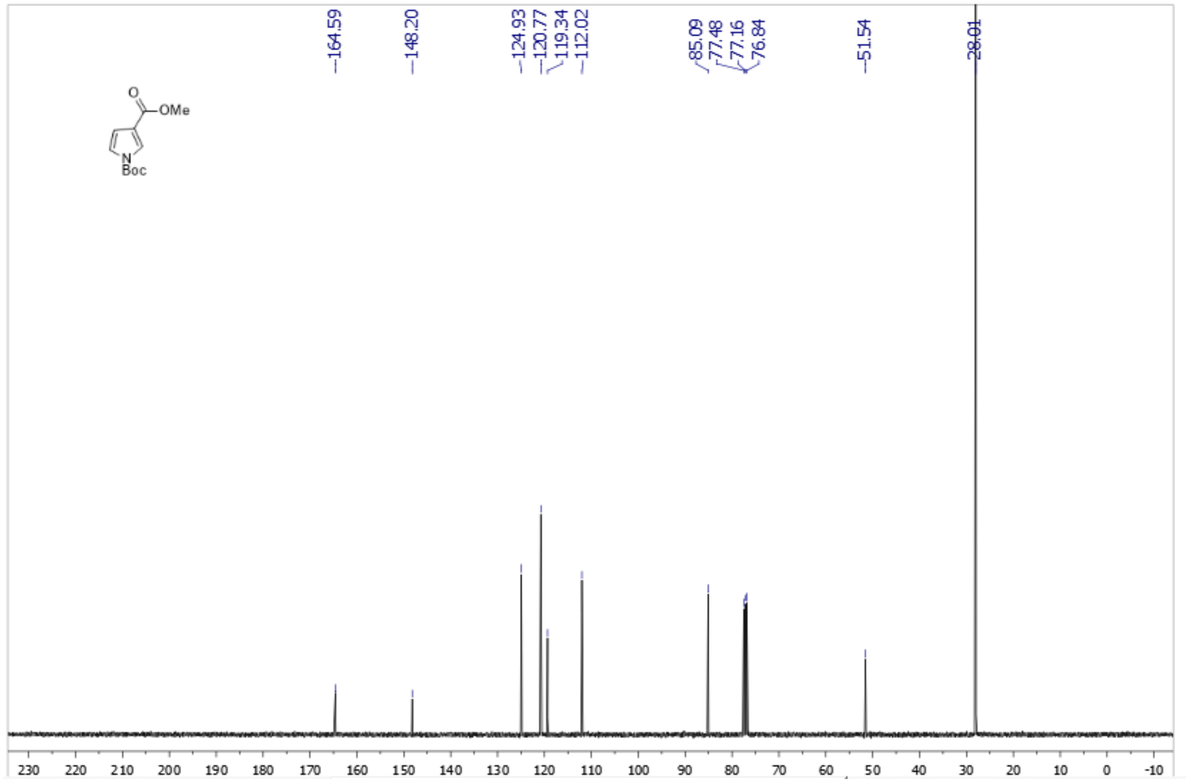

Supplementary Figure 24. ^{13}C NMR spectra of **1s**

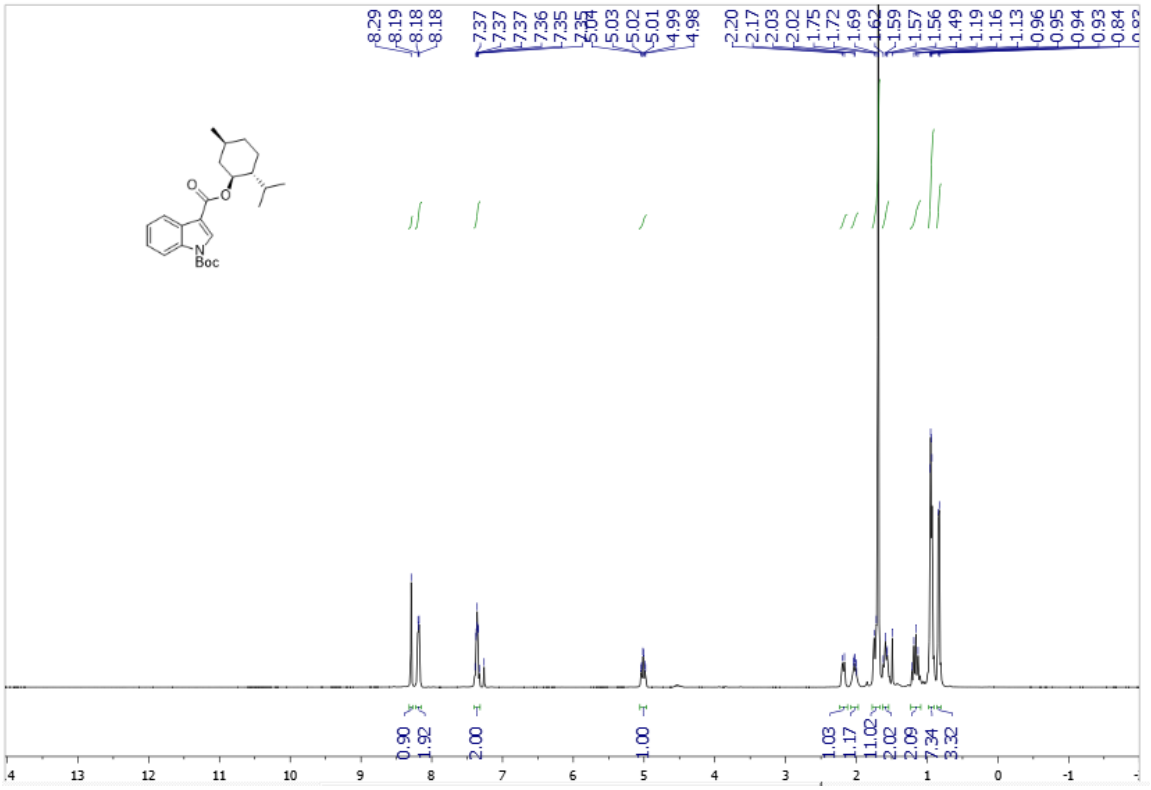

Supplementary Figure 25. ^{1}H NMR spectra of **1as**

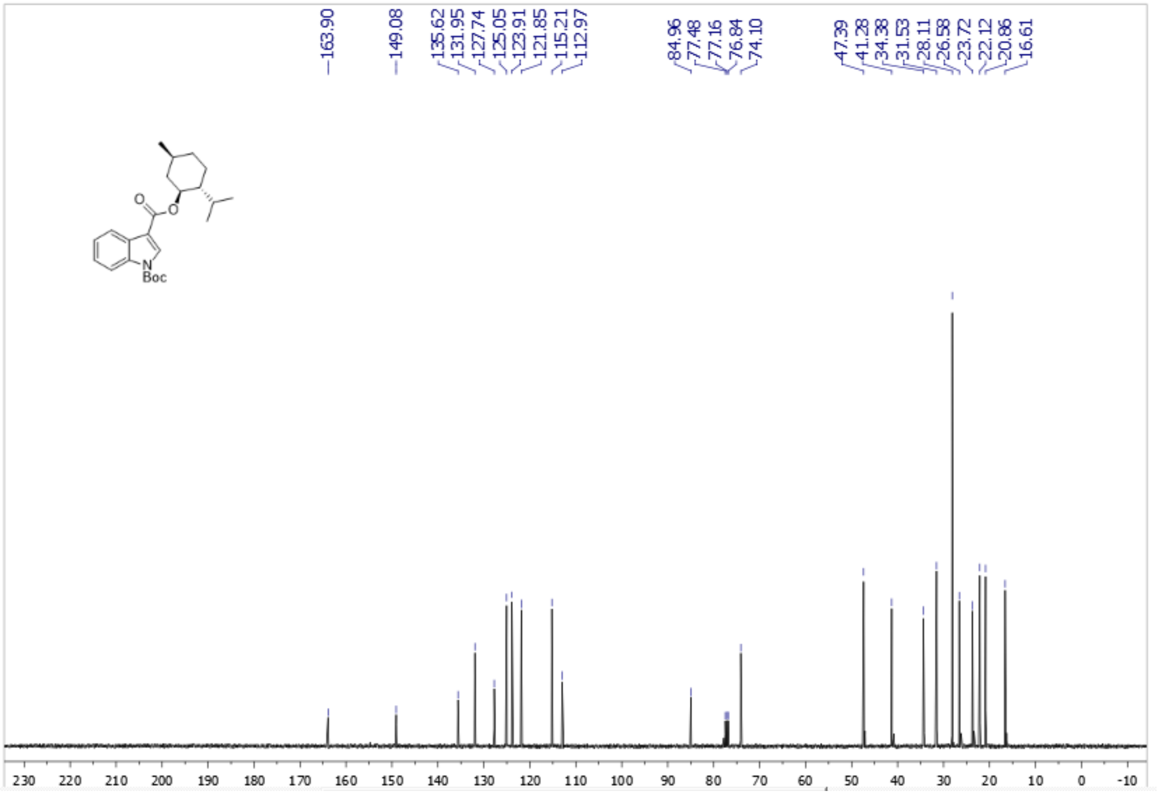

Supplementary Figure 26. ^{13}C NMR spectra of **1as**

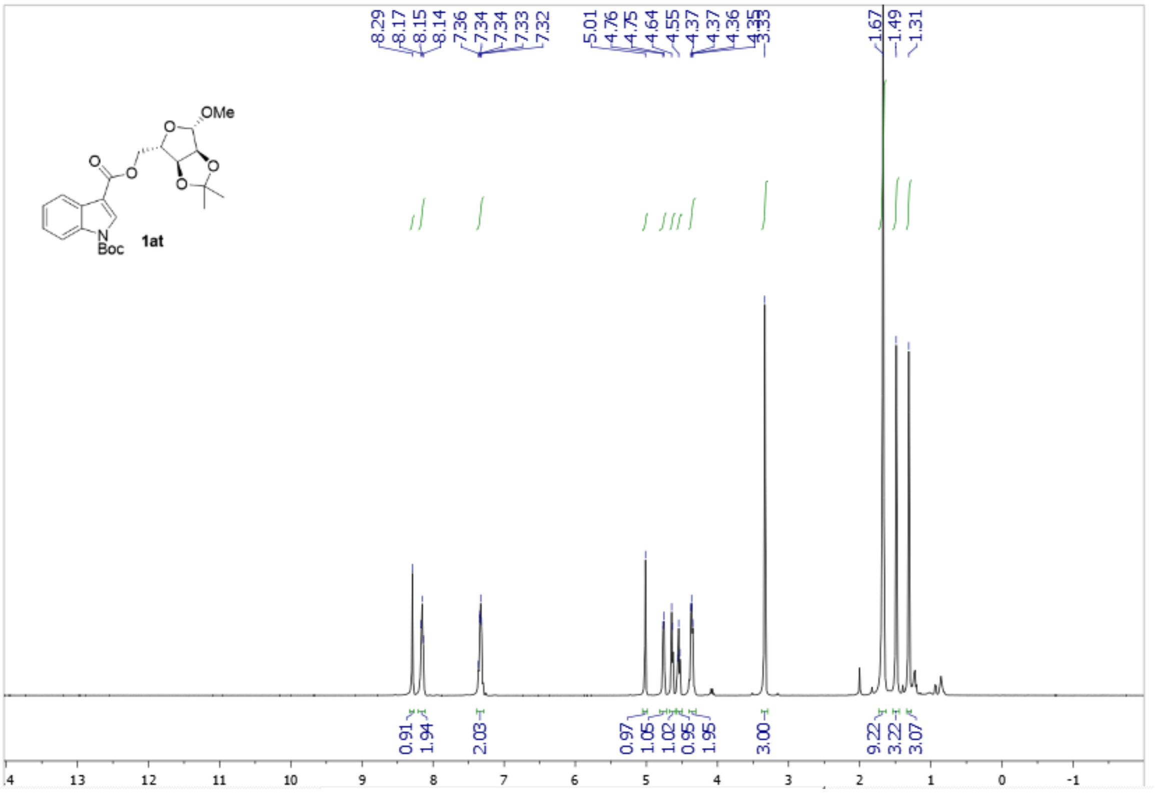

Supplementary Figure 27. ^{1}H NMR spectra of **1at**

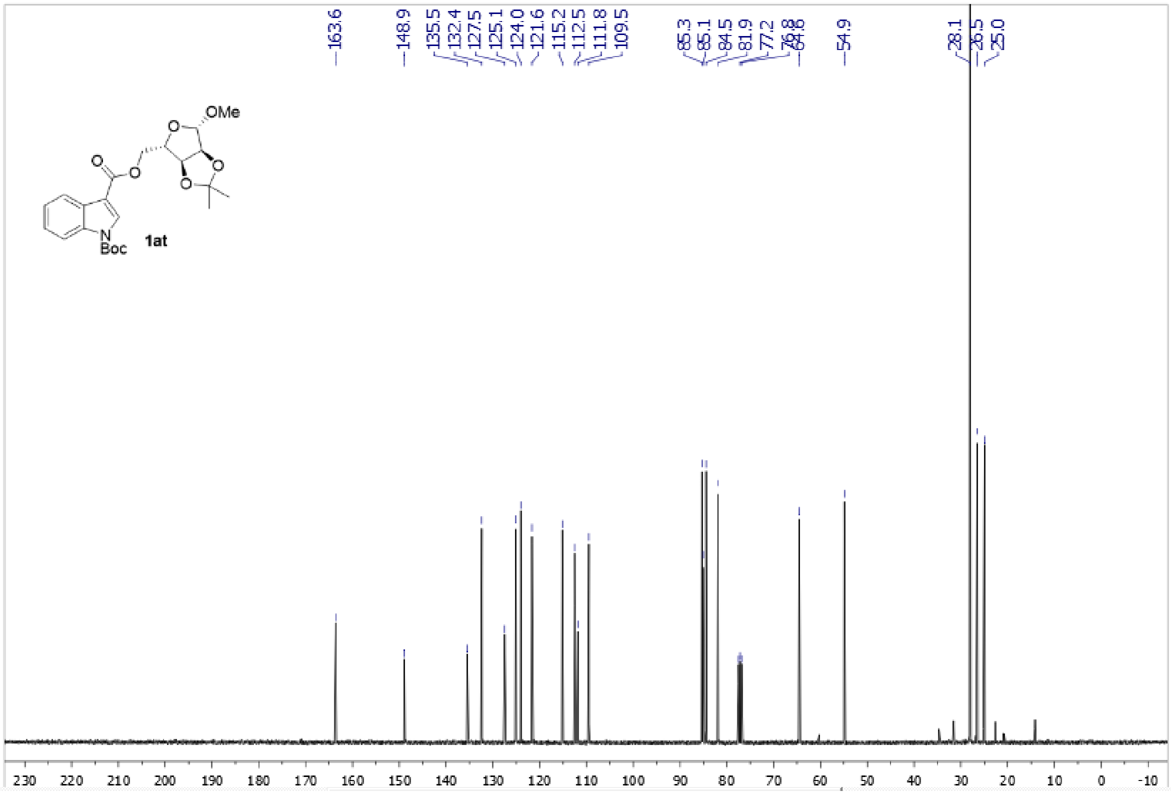

Supplementary Figure 28. ^{13}C NMR spectra of **1at**

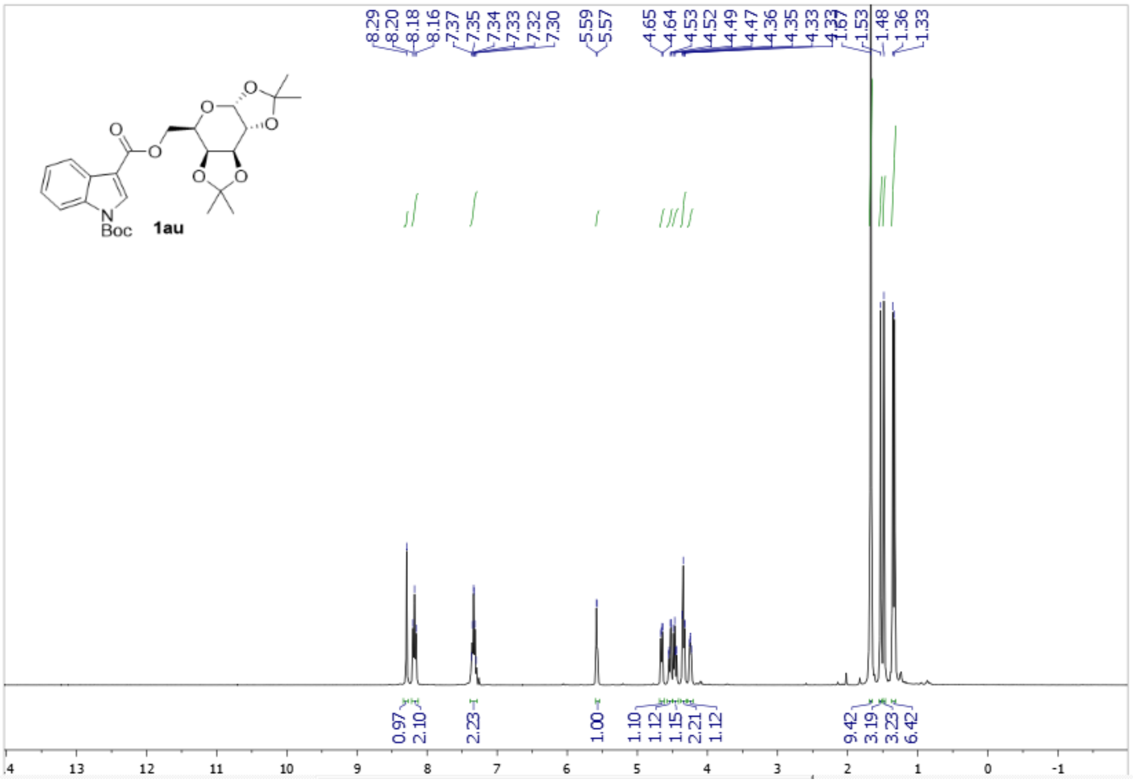

Supplementary Figure 29. ^{1}H NMR spectra of **1au**

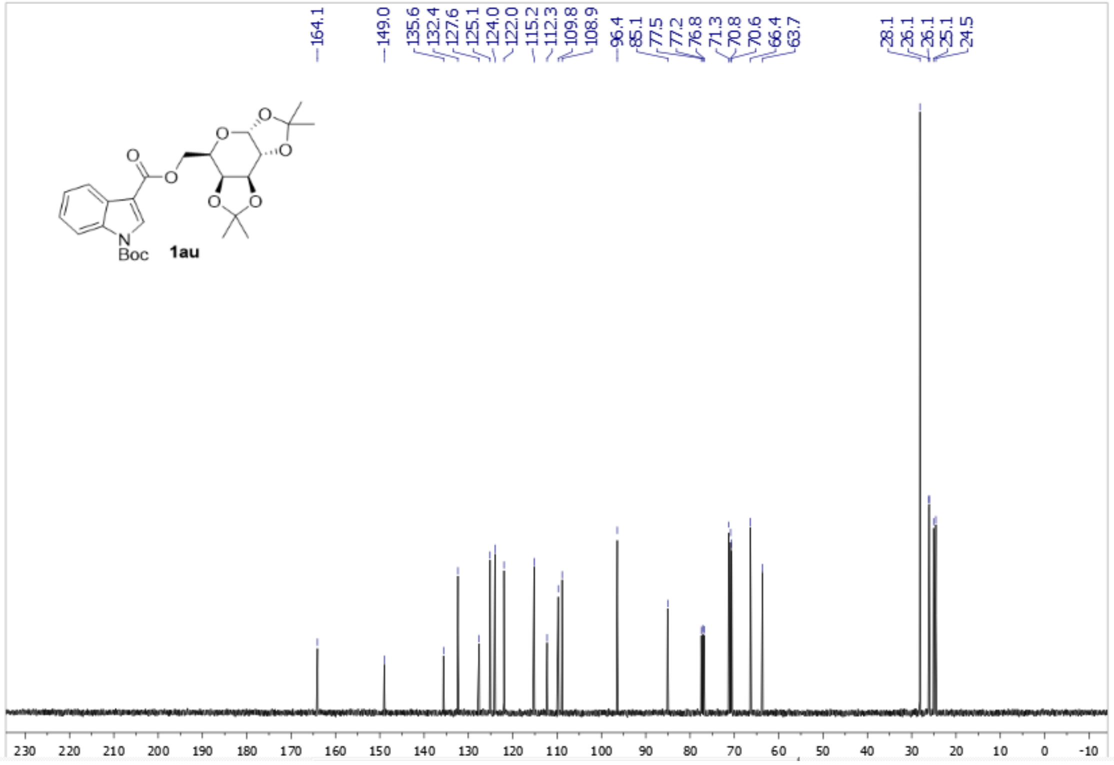

Supplementary Figure 30. ^{13}C NMR spectra of **1au**

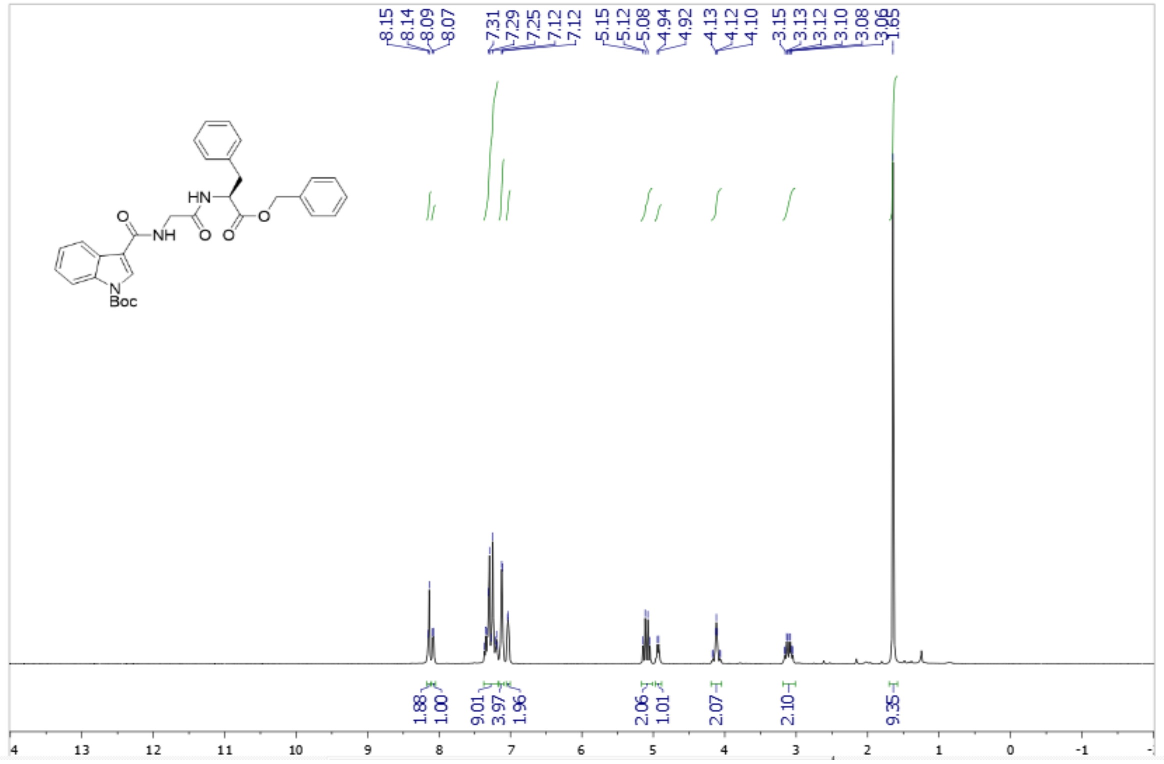

Supplementary Figure 31. ^{1}H NMR spectra of **1av**

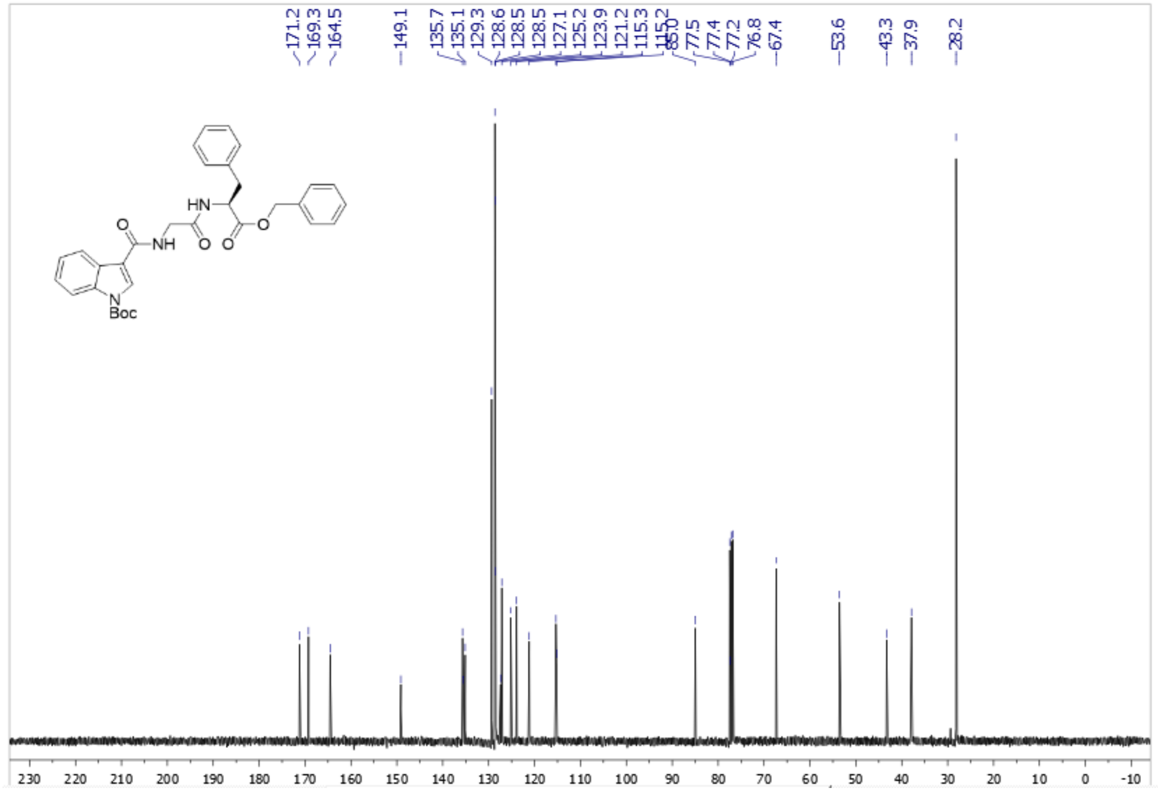

Supplementary Figure 32. ^{13}C NMR spectra of **1av**

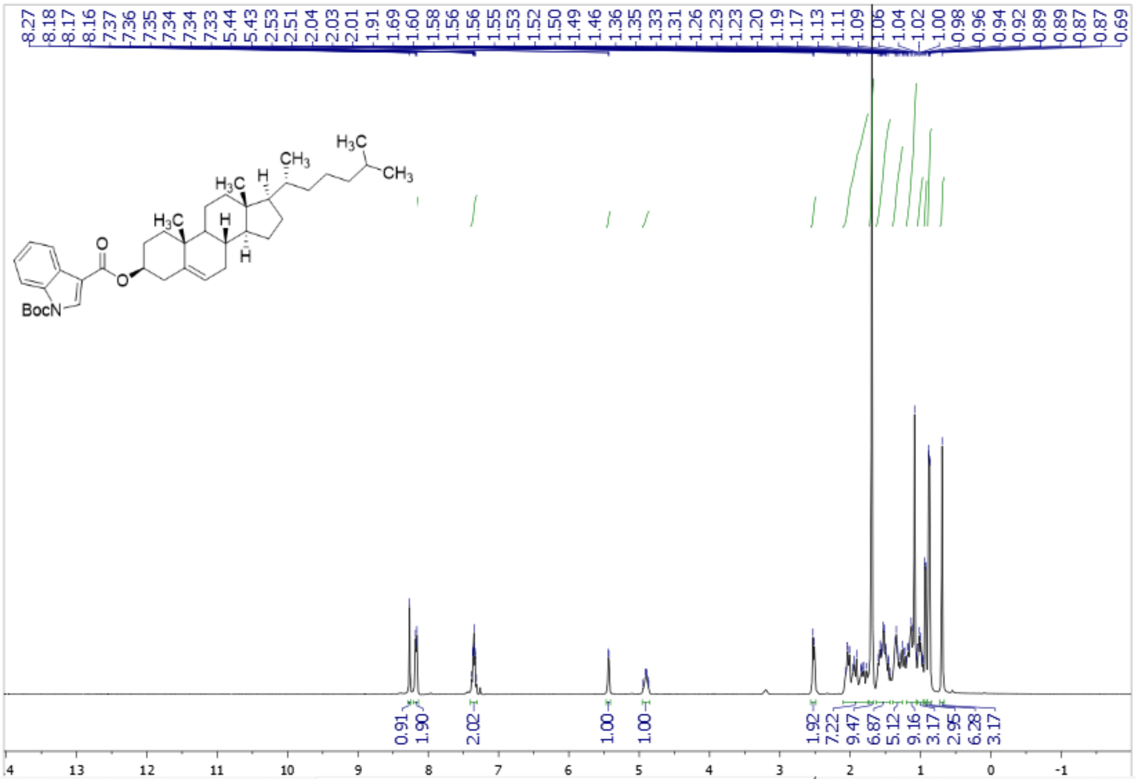

Supplementary Figure 33. ^{1}H NMR spectra of **1aw**

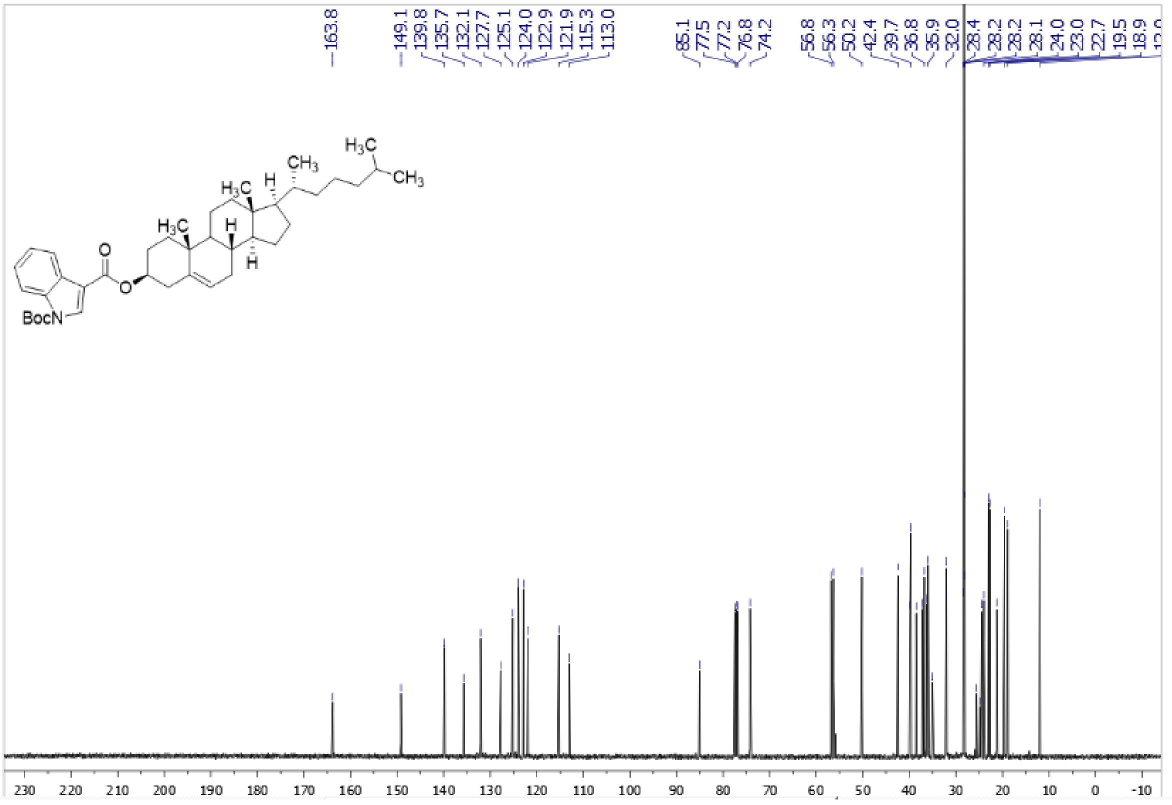

Supplementary Figure 34. ^{13}C NMR spectra of **1aw**

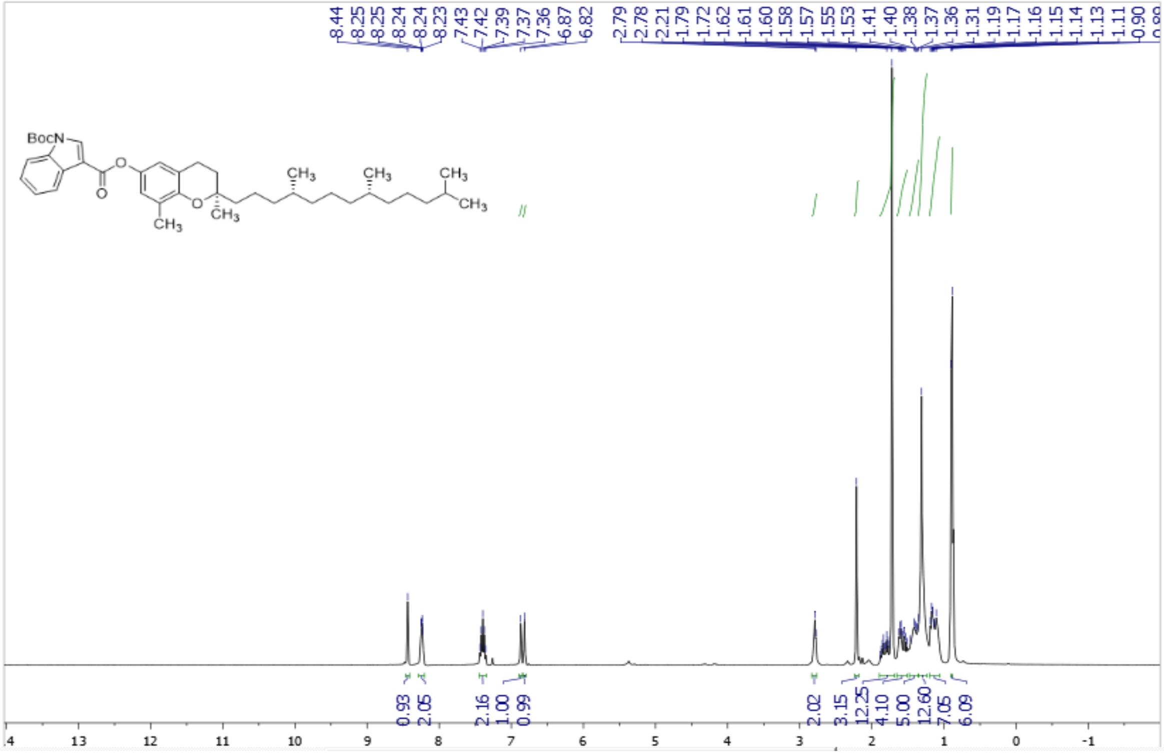

Supplementary Figure 35. ^{1}H NMR spectra of **1ax**

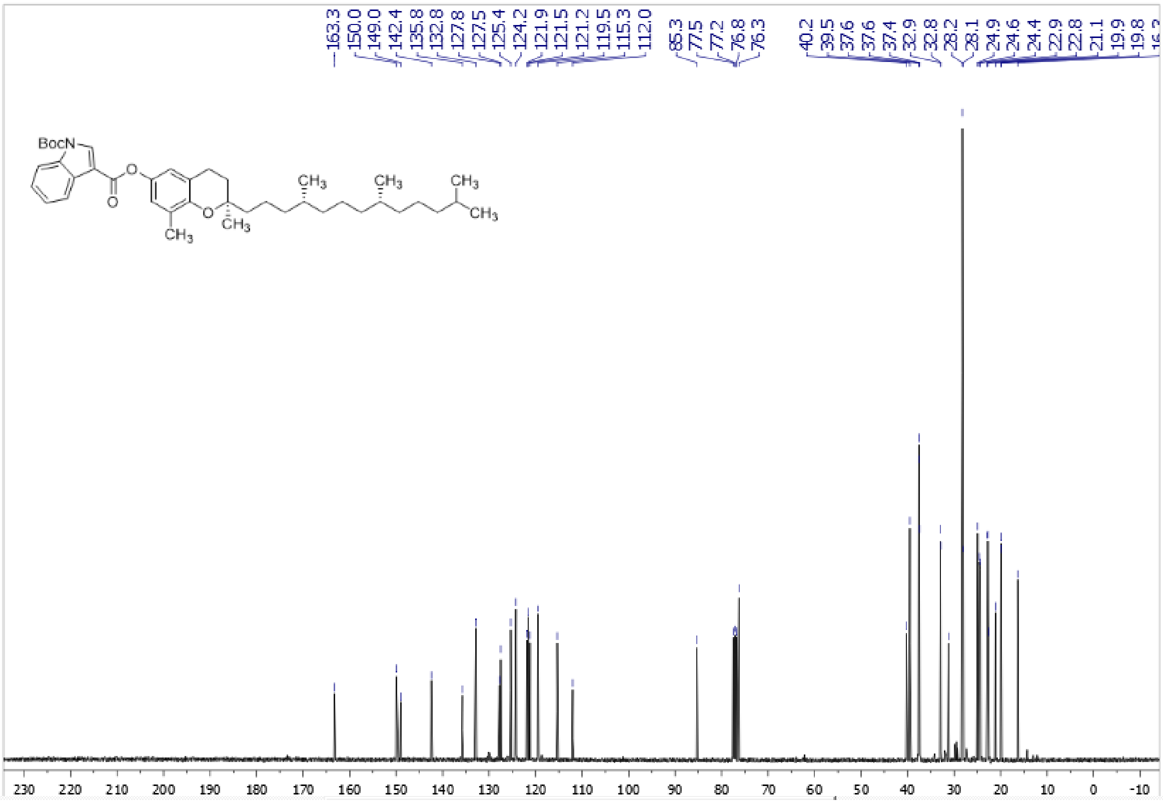

Supplementary Figure 36. ^{13}C NMR spectra of **1ax**

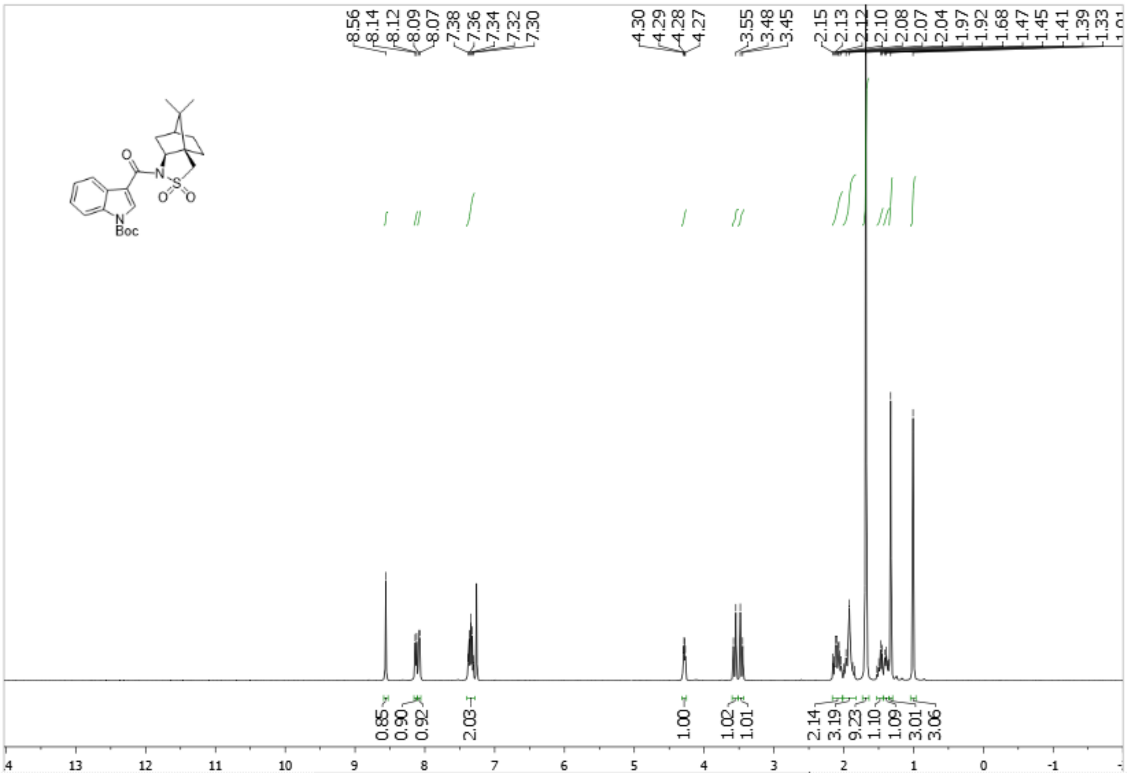

Supplementary Figure 37. ^{1}H NMR spectra of **5b**

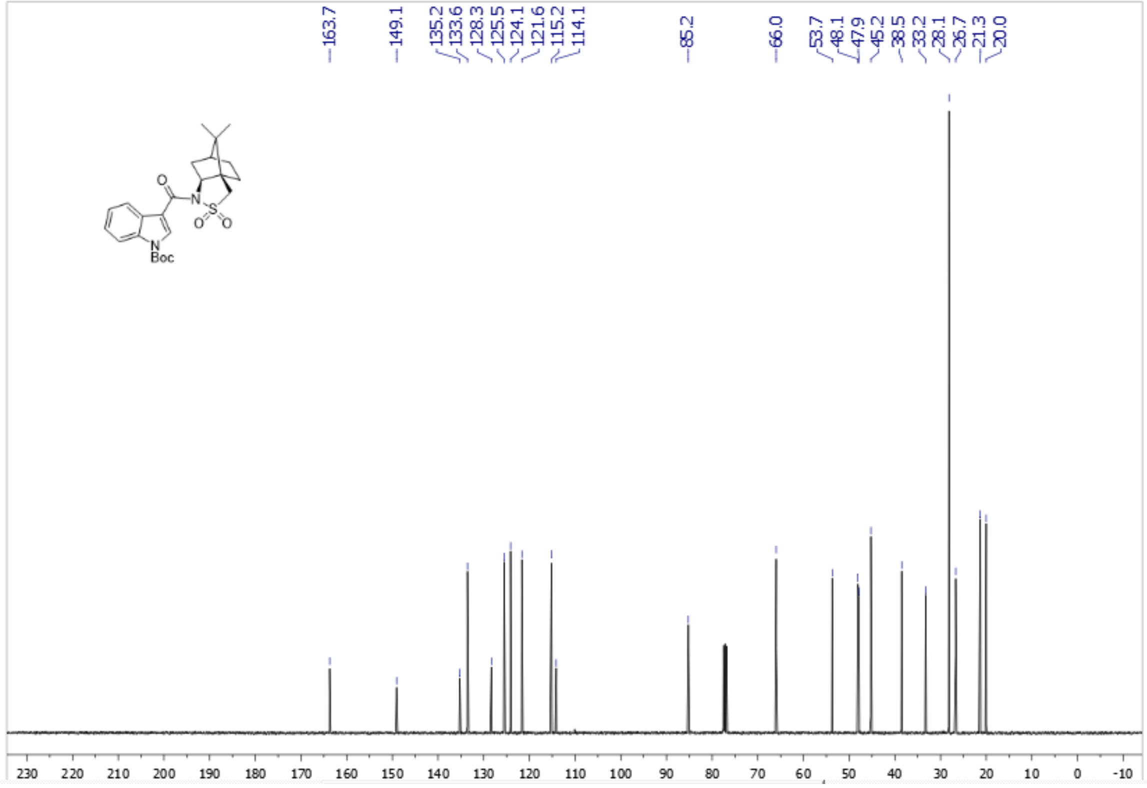

Supplementary Figure 38. ^{13}C NMR spectra of **5b**

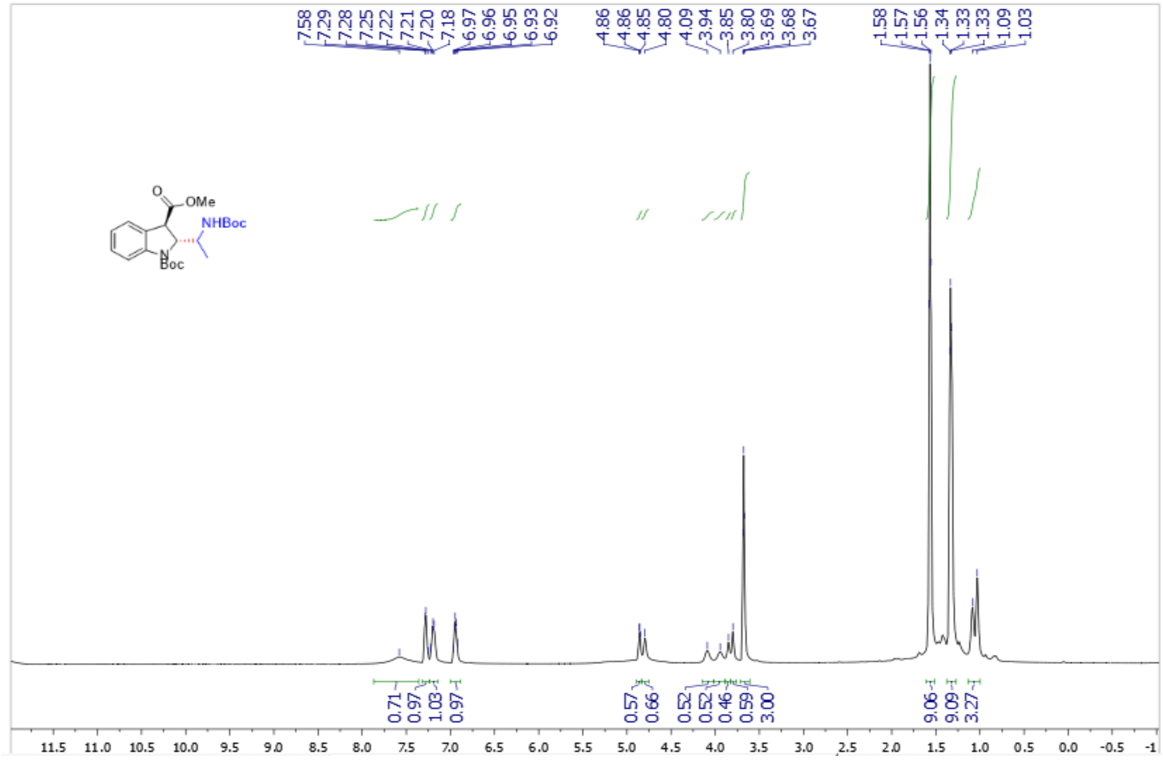

Supplementary Figure 39. ^{1}H NMR spectra of *trans*-**3a**

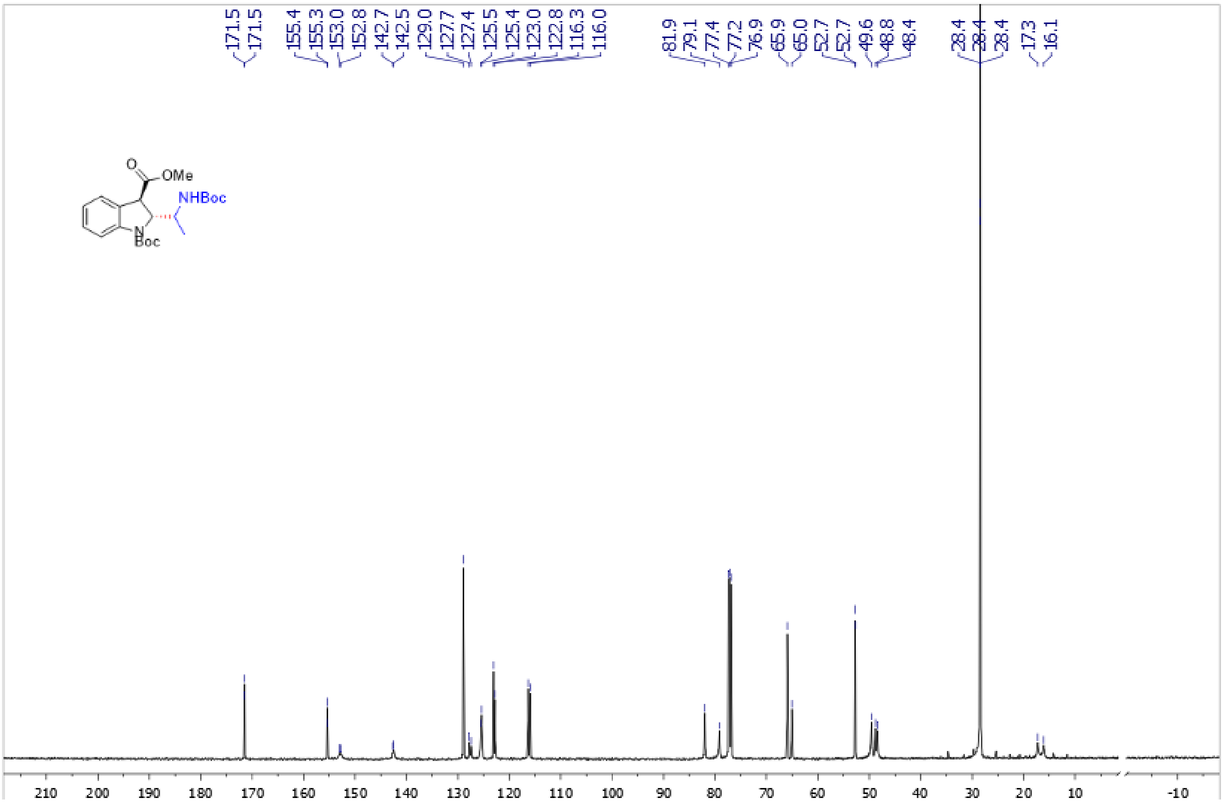

Supplementary Figure 40. ^{13}C NMR spectra of *trans*-**3a**

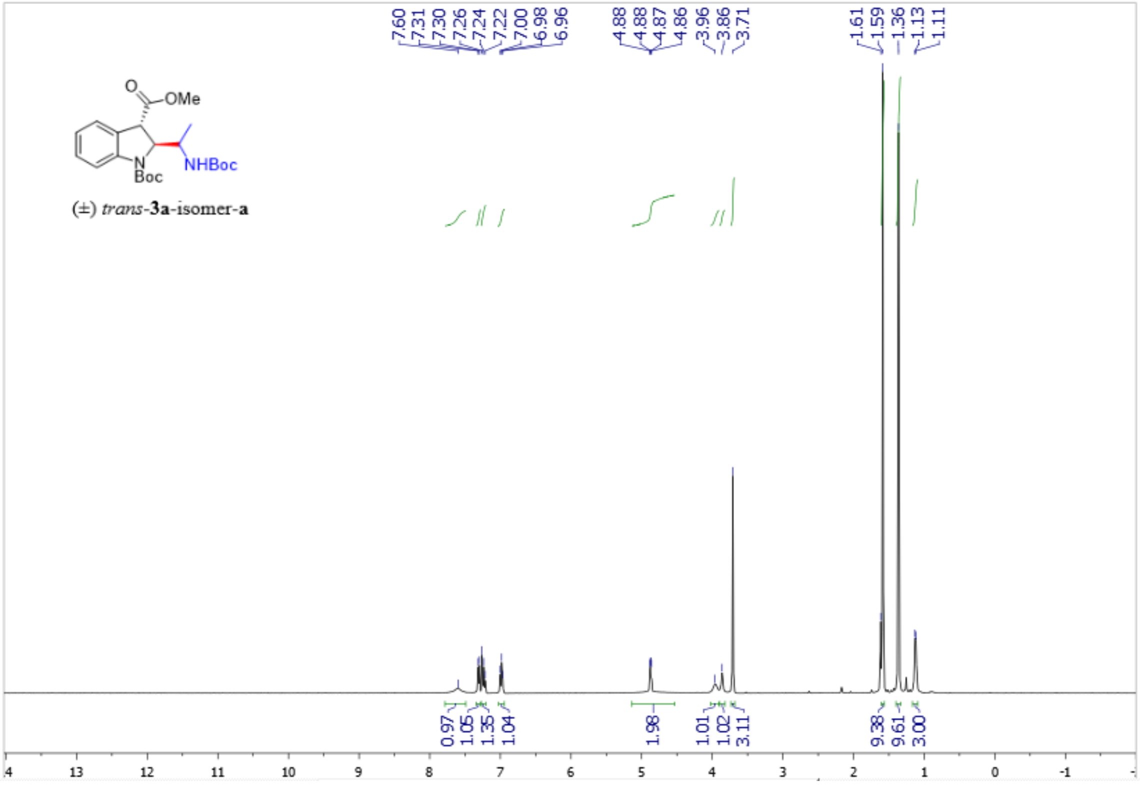

Supplementary Figure 41. ^{1}H NMR spectra of *trans*-**3a**-isomer-**a**

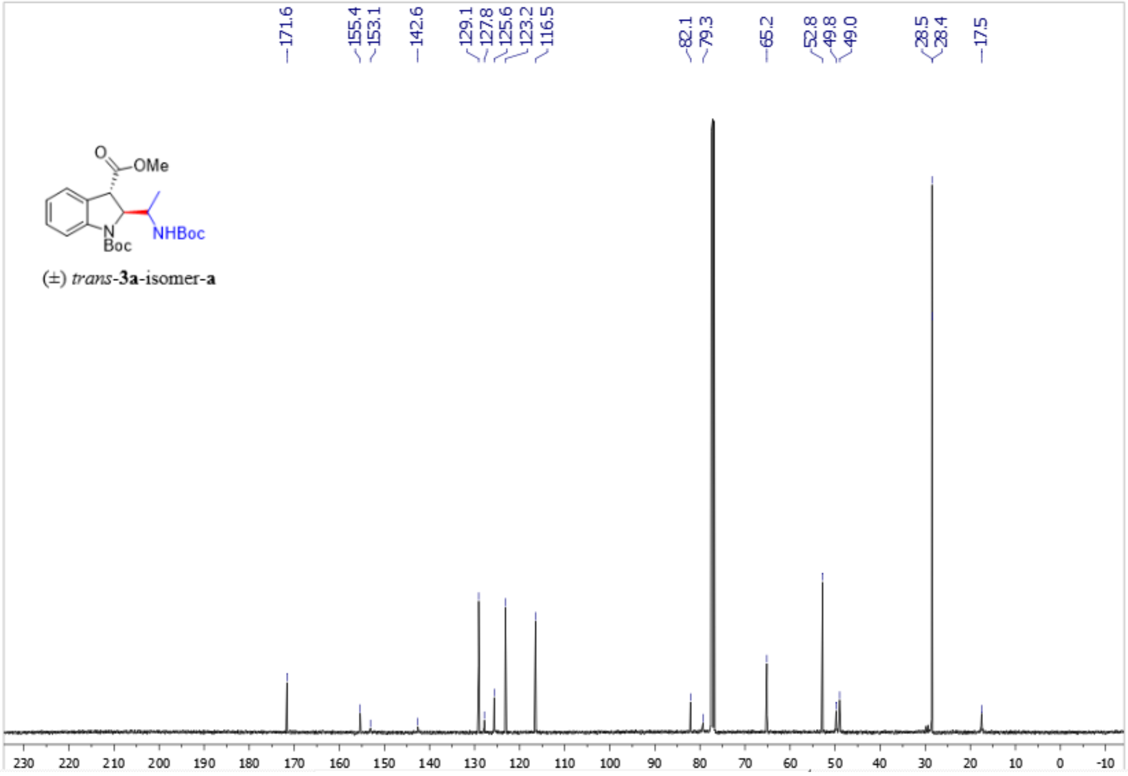

Supplementary Figure 42. ^{13}C NMR spectra of *trans*-**3a**-isomer-**a**

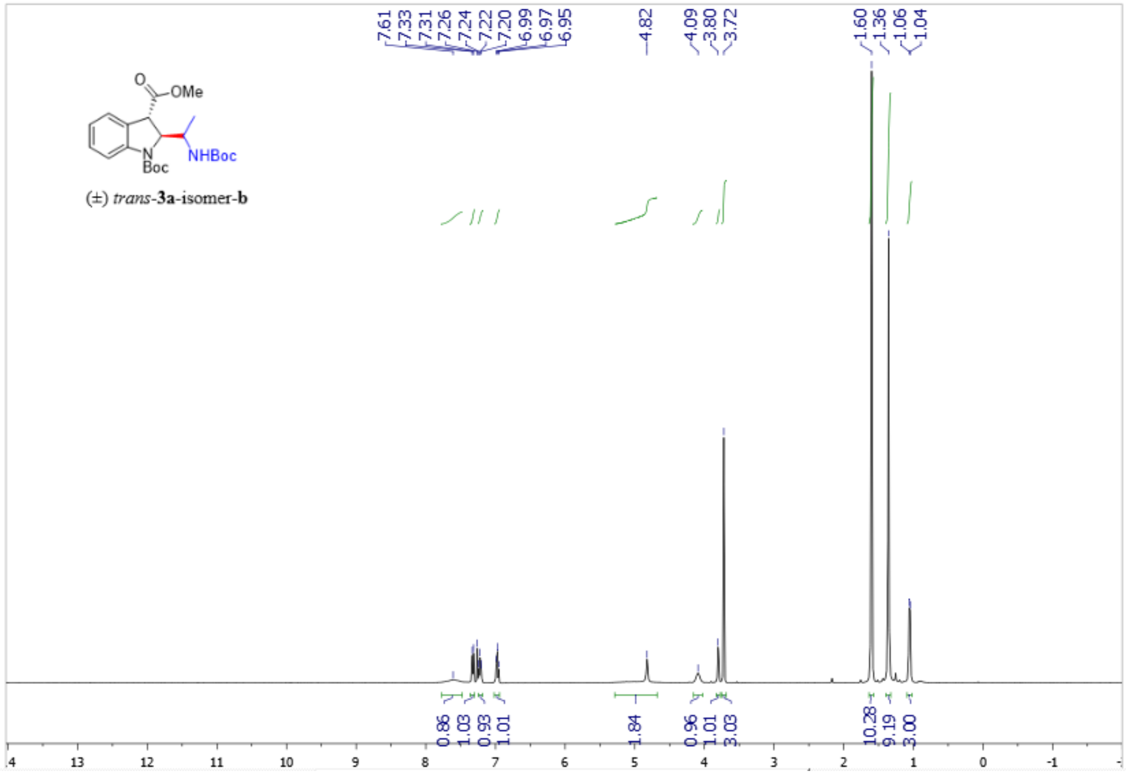

Supplementary Figure 43. ^{1}H NMR spectra of *trans*-**3a**-isomer-**b**

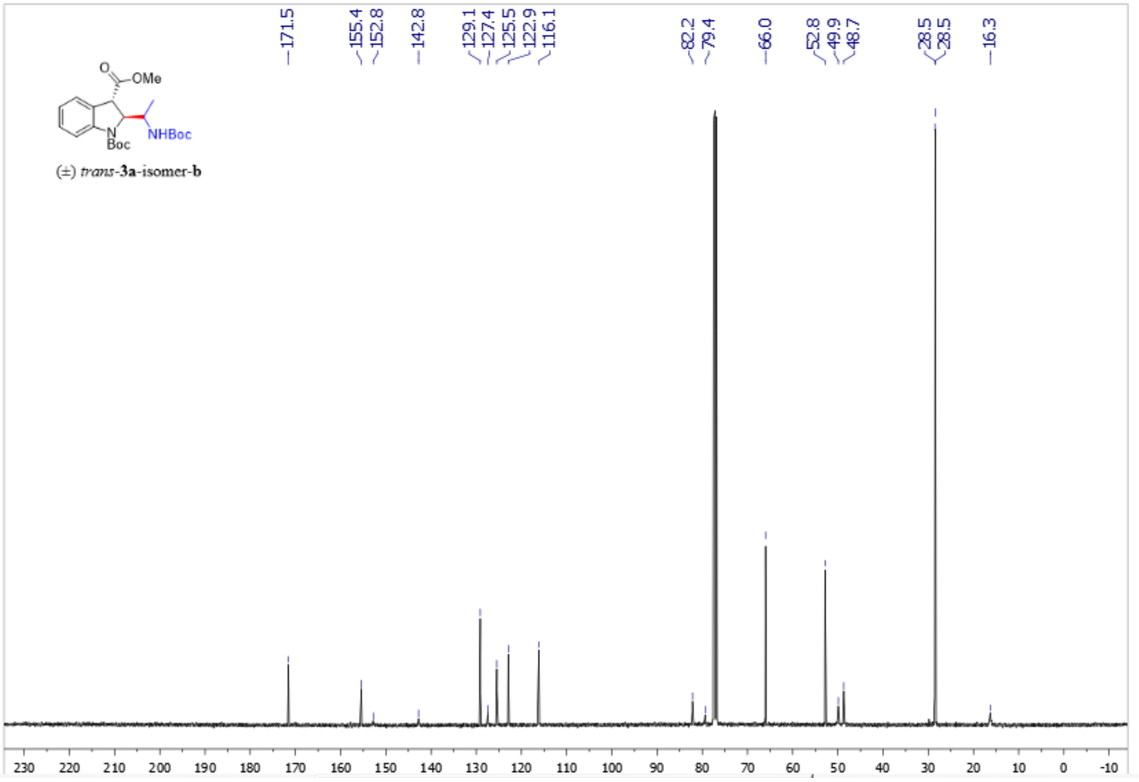

Supplementary Figure 44. ^{13}C NMR spectra of *trans*-**3a**-isomer-**b**

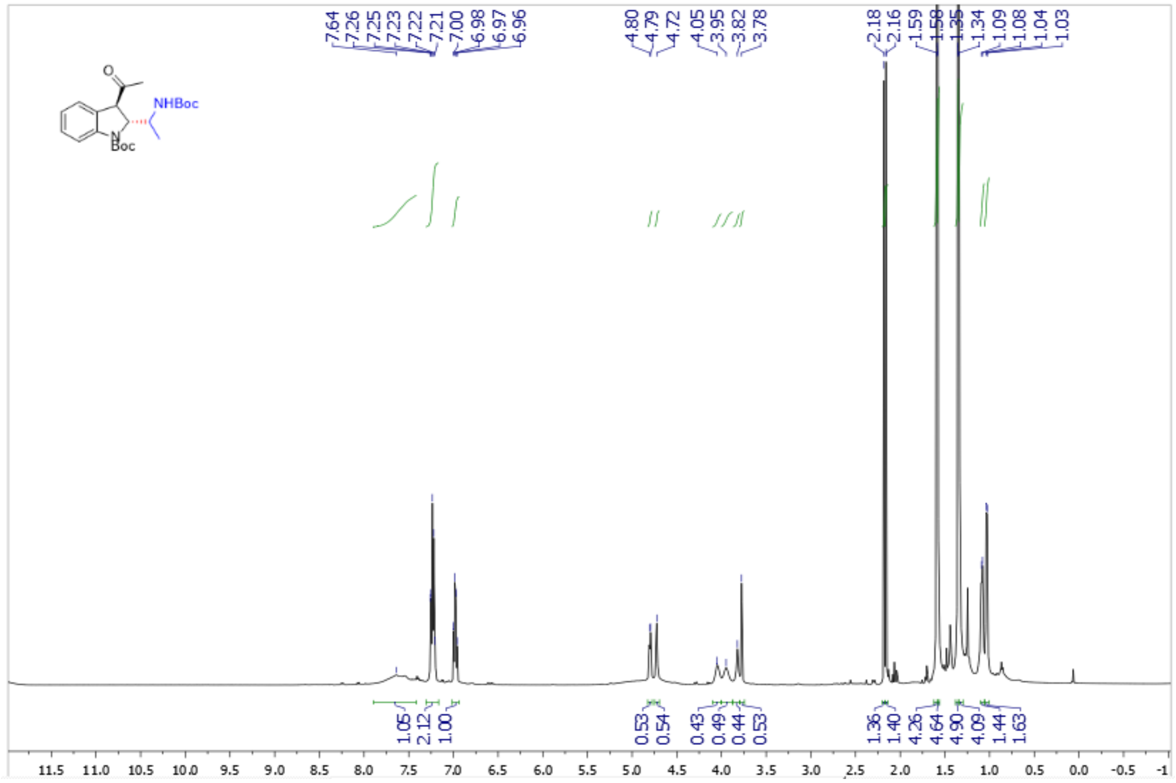

Supplementary Figure 45. ^{1}H NMR spectra of *trans*-**3b**

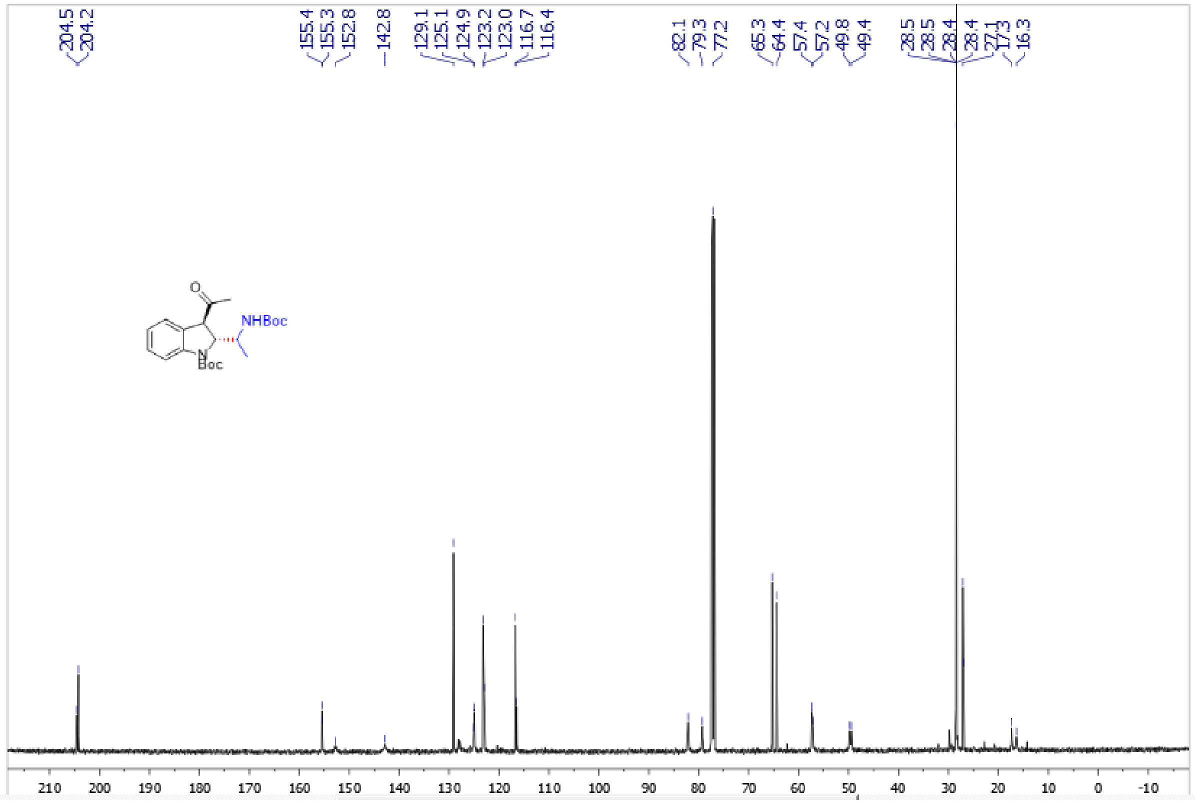

Supplementary Figure 46. ^{13}C NMR spectra of *trans*-**3b**

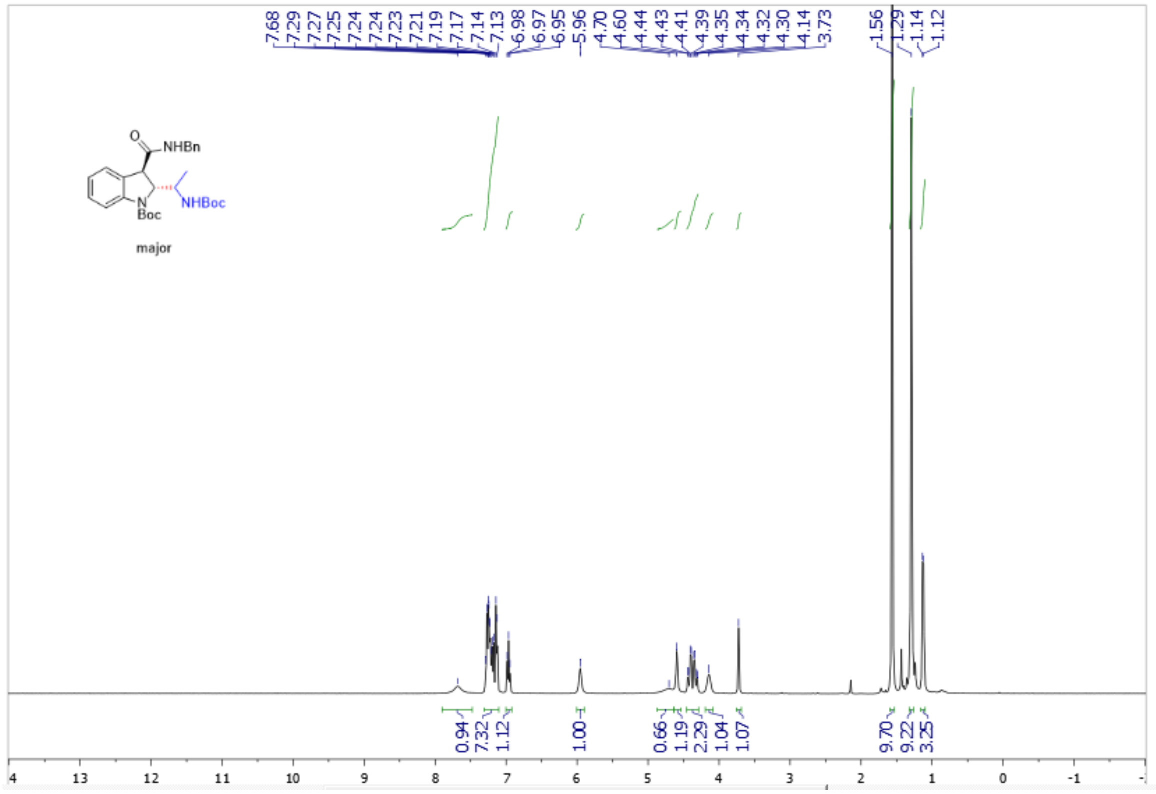

Supplementary Figure 47. ^{1}H NMR spectra of *trans*-**3c-major**

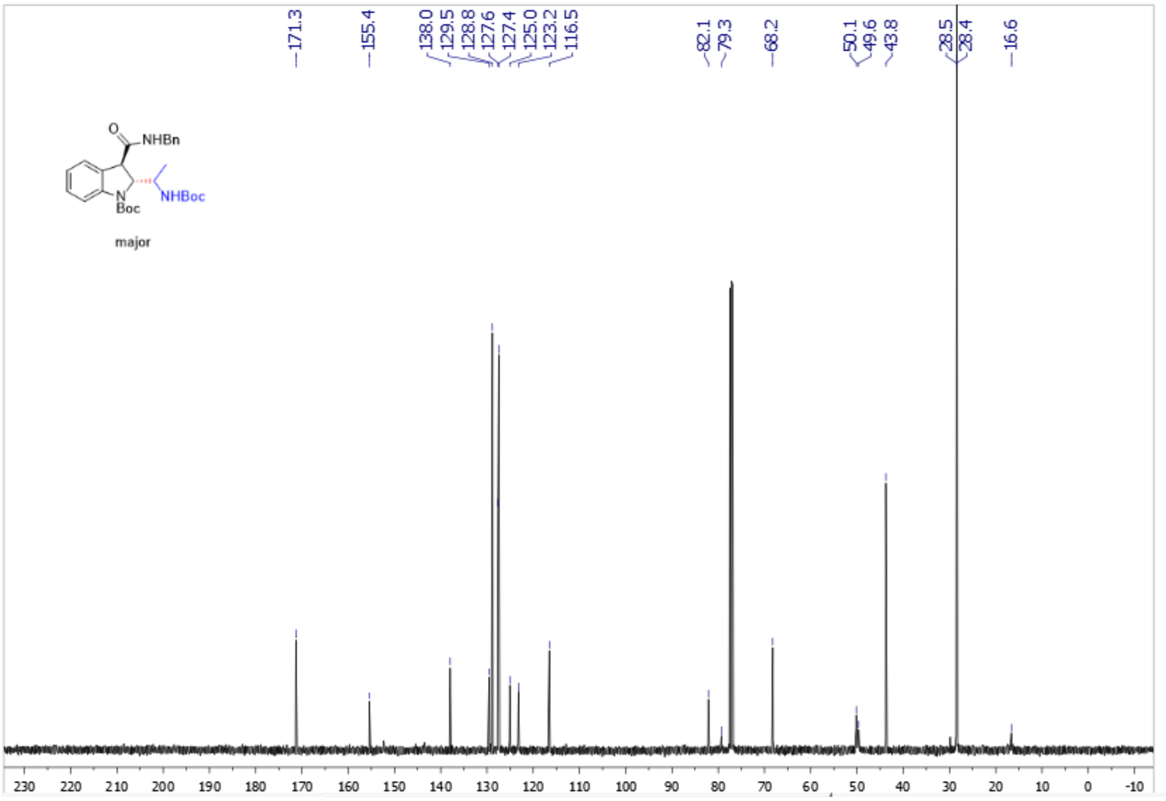

Supplementary Figure 48. ^{13}C NMR spectra of *trans*-**3c-major**

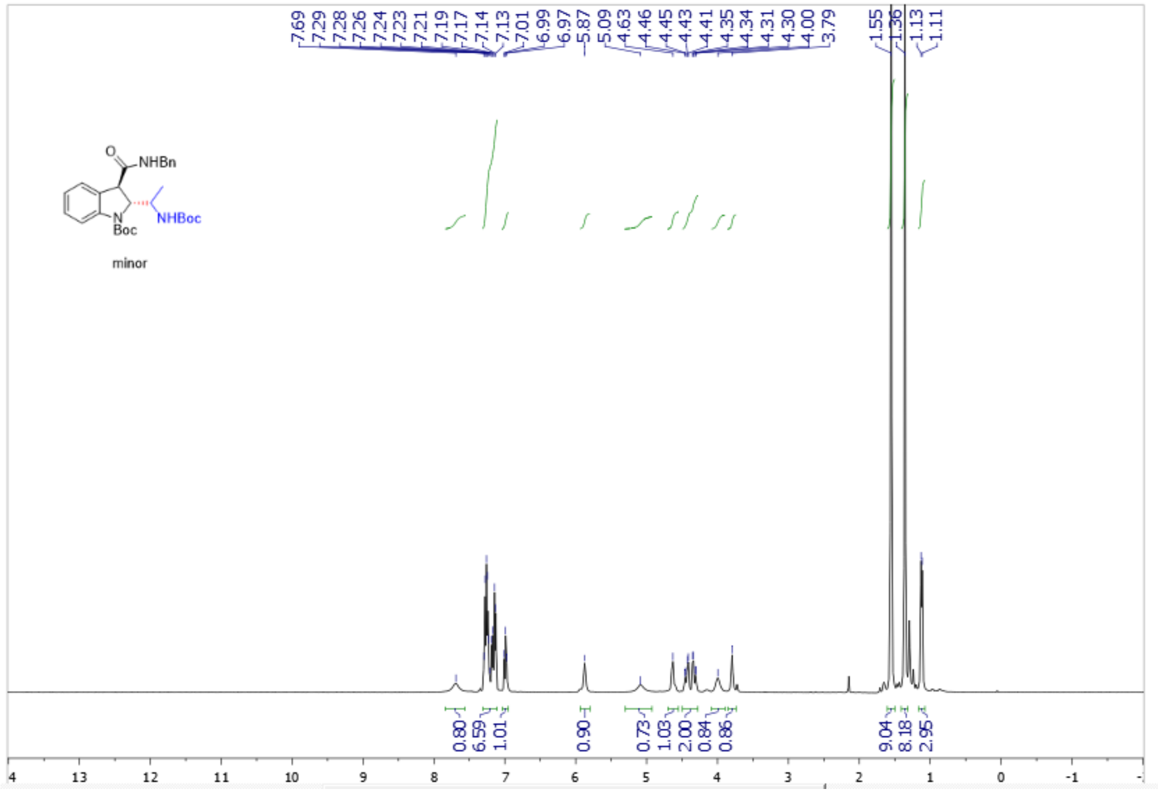

Supplementary Figure 49. ^{1}H NMR spectra of *trans*-**3c-minor**

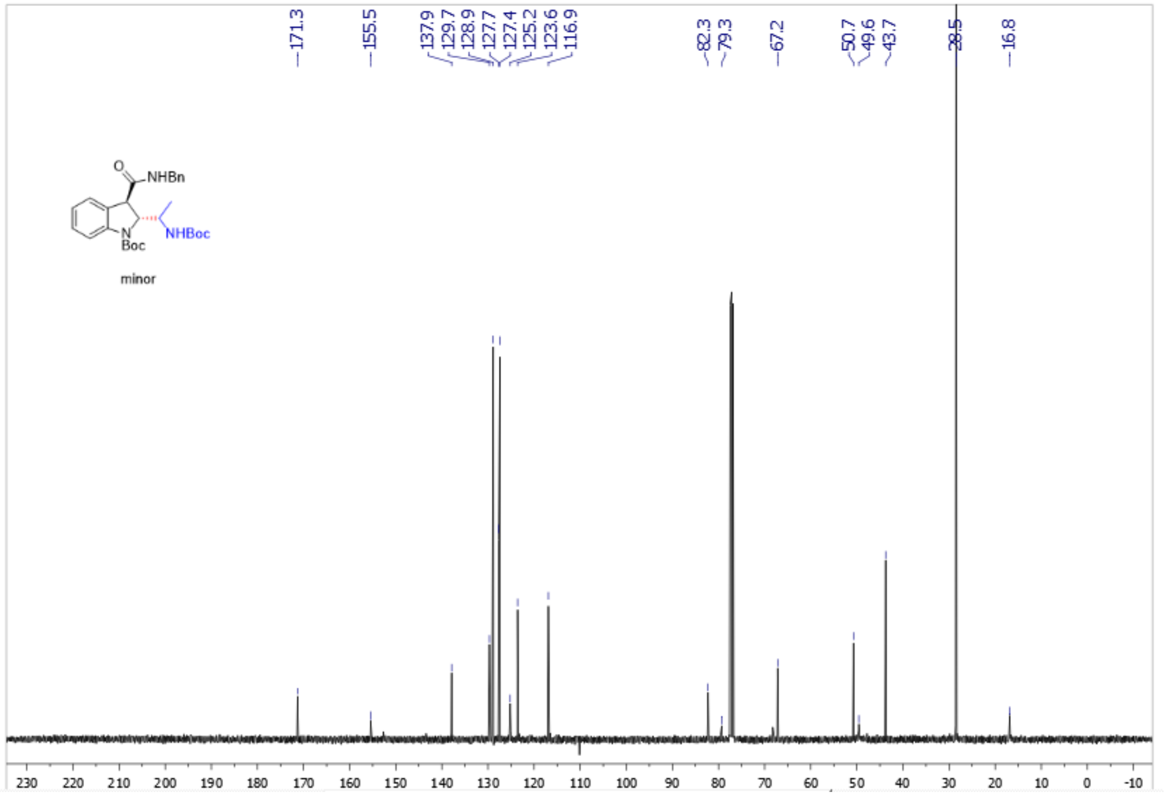

Supplementary Figure 50. ^{13}C NMR spectra of *trans*-**3c-minor**

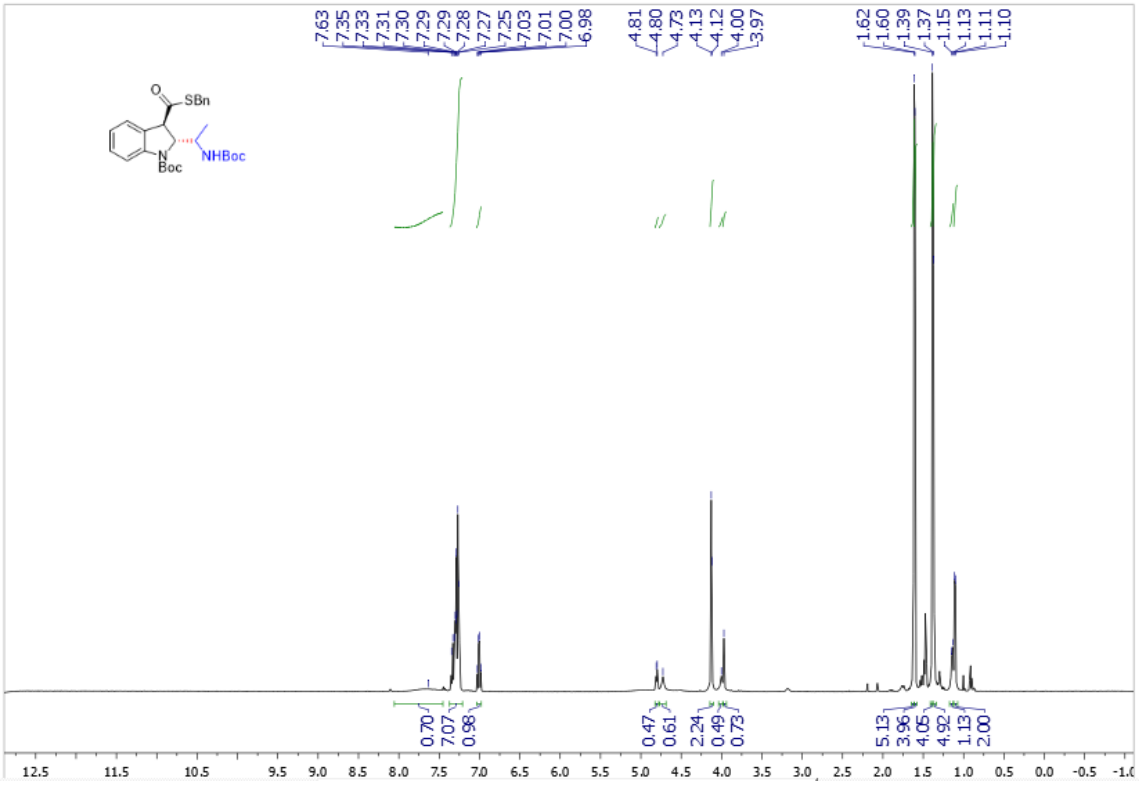

Supplementary Figure 51. ^{1}H NMR spectra of *trans*-**3d**

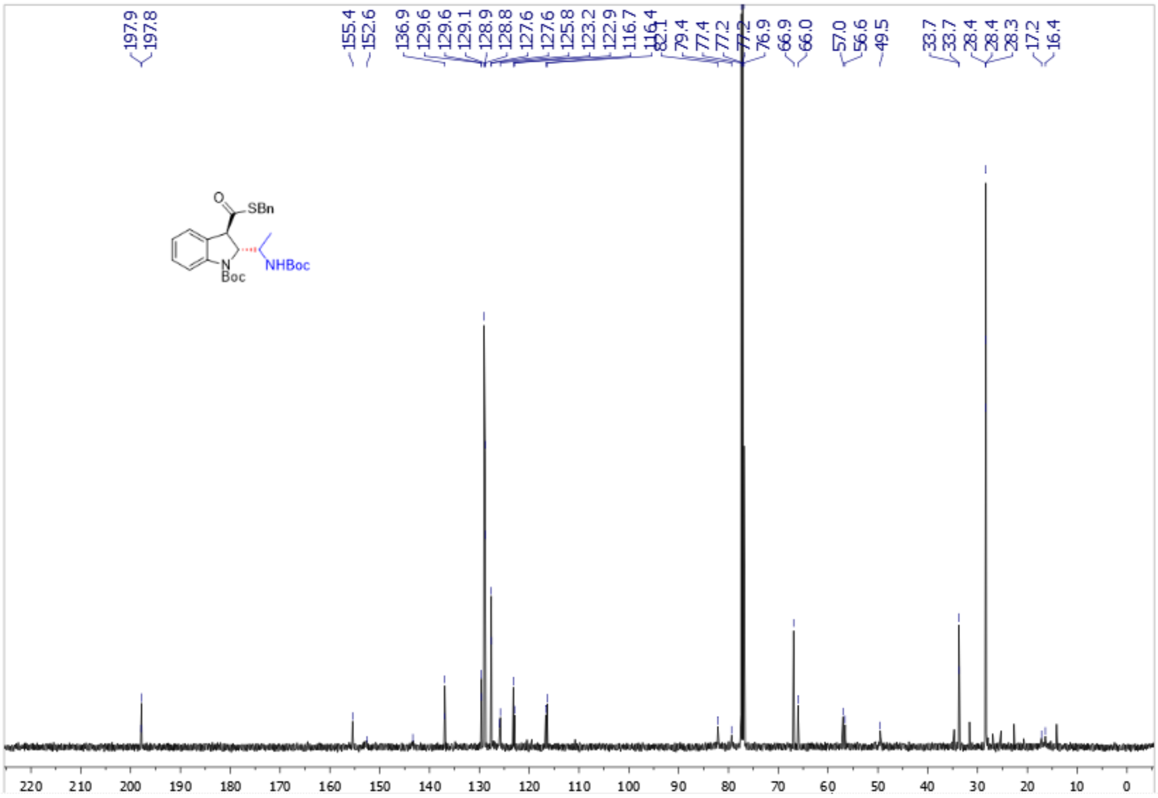

Supplementary Figure 52. ^{13}C NMR spectra of *trans*-**3d**

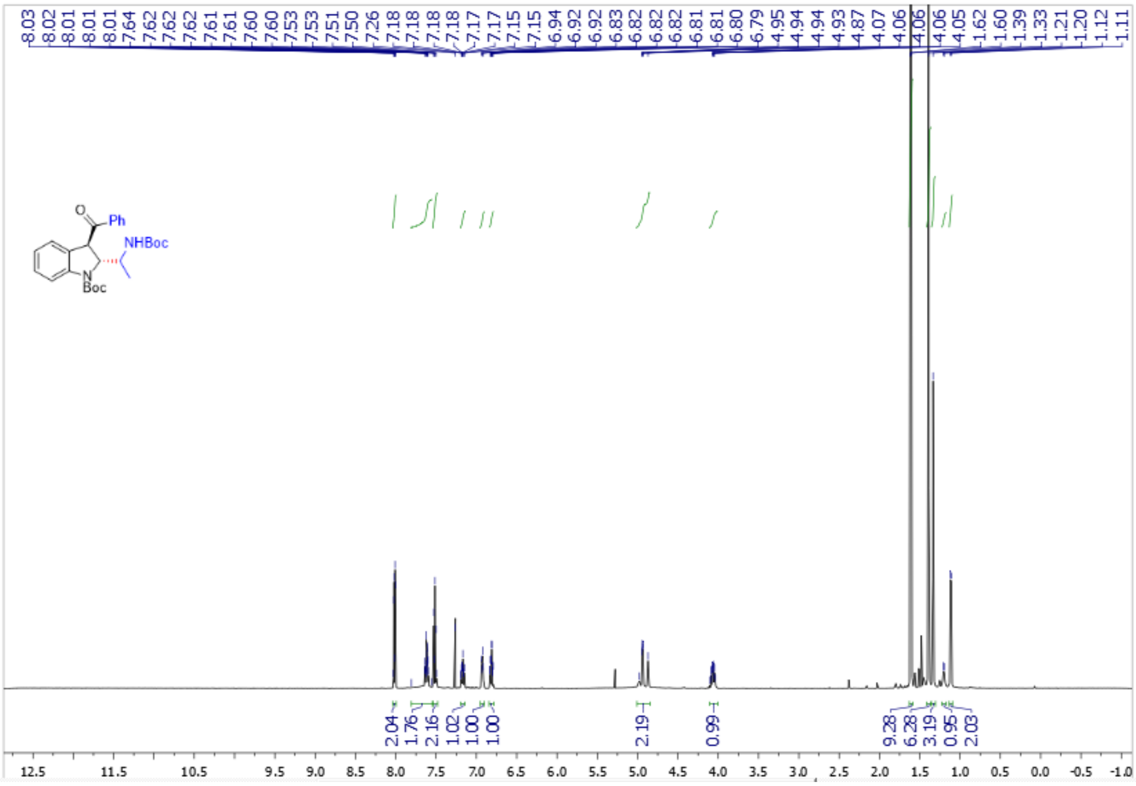

Supplementary Figure 53. ^{1}H NMR spectra of *trans*-**3e**

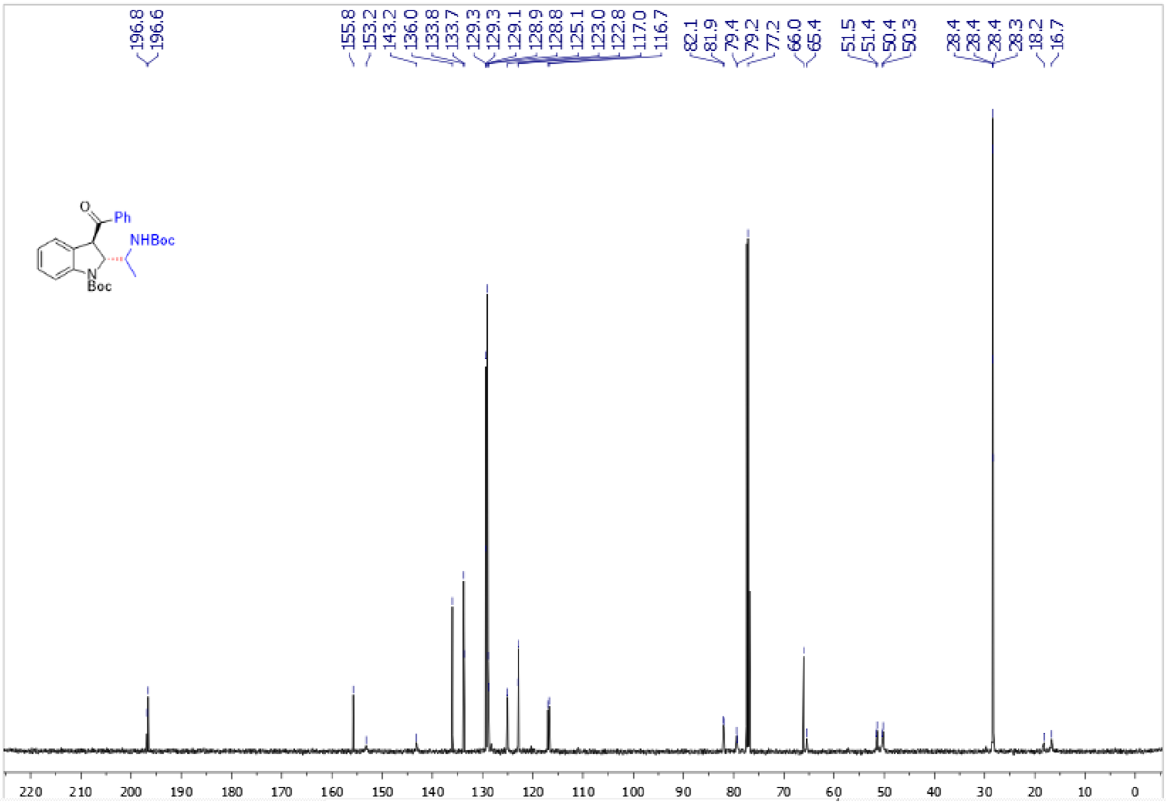

Supplementary Figure 54. ^{13}C NMR spectra of *trans*-**3e**

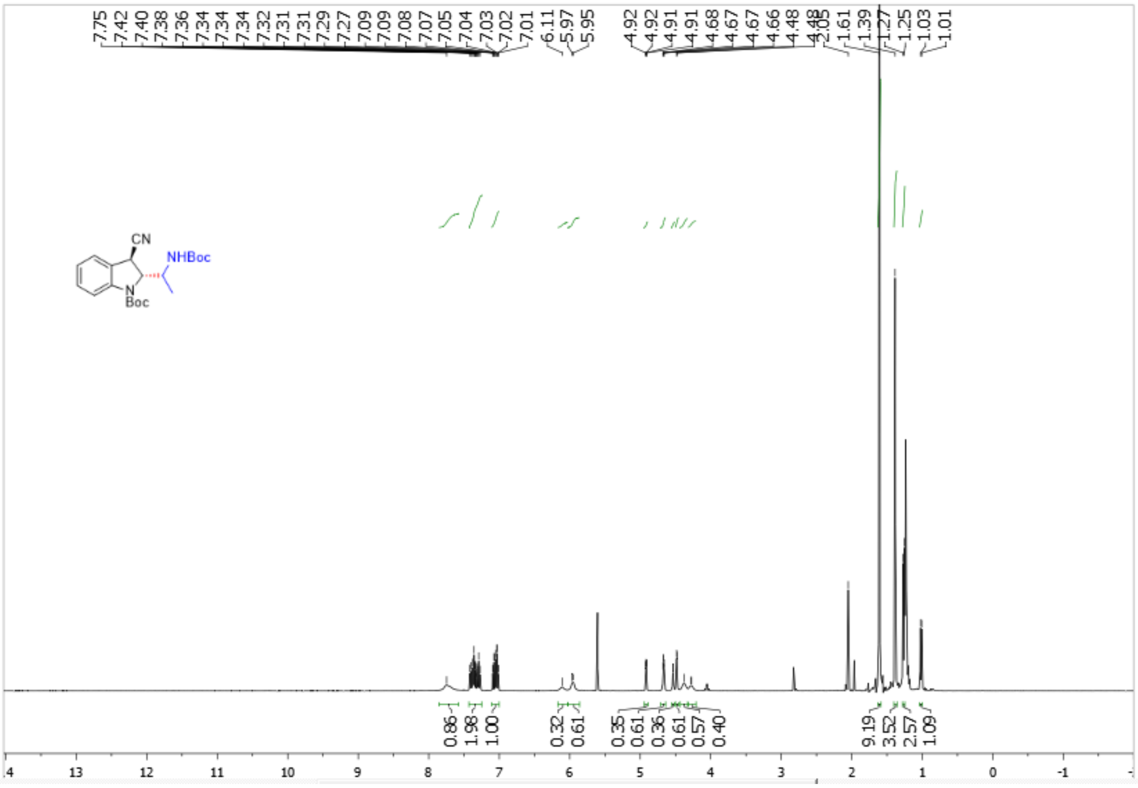

Supplementary Figure 55. ^{1}H NMR spectra of *trans*-**3f**

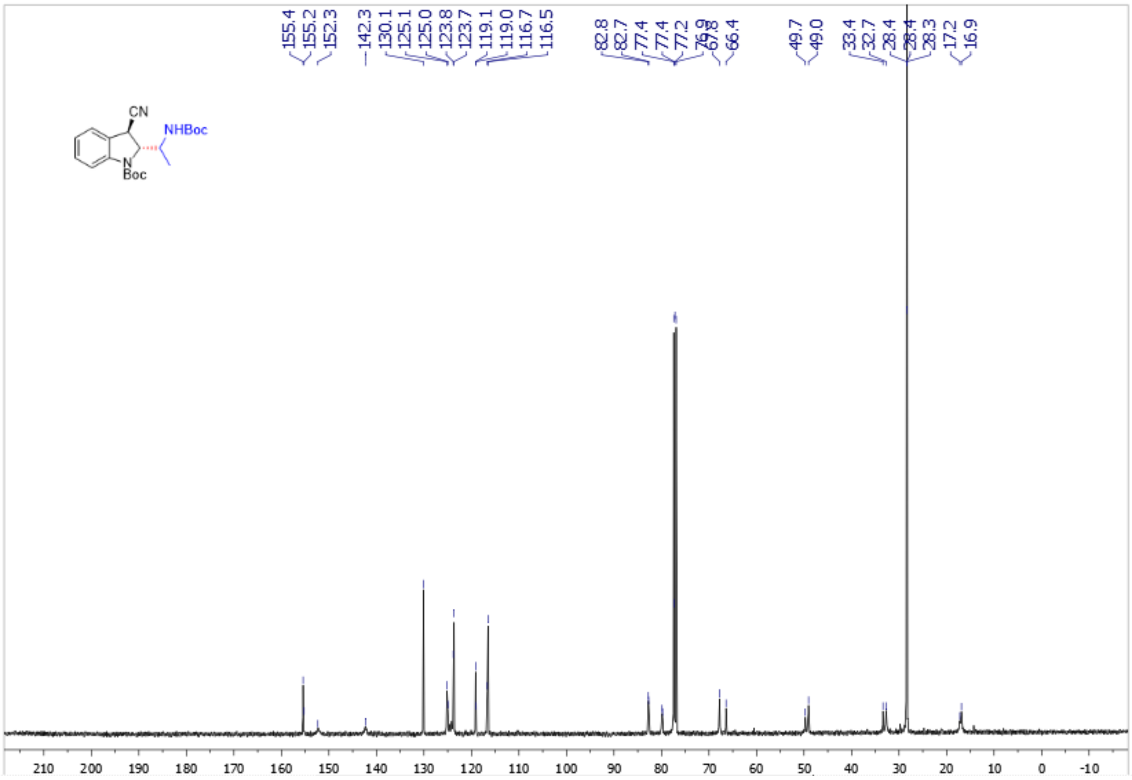

Supplementary Figure 56. ^{13}C NMR spectra of *trans*-**3f**

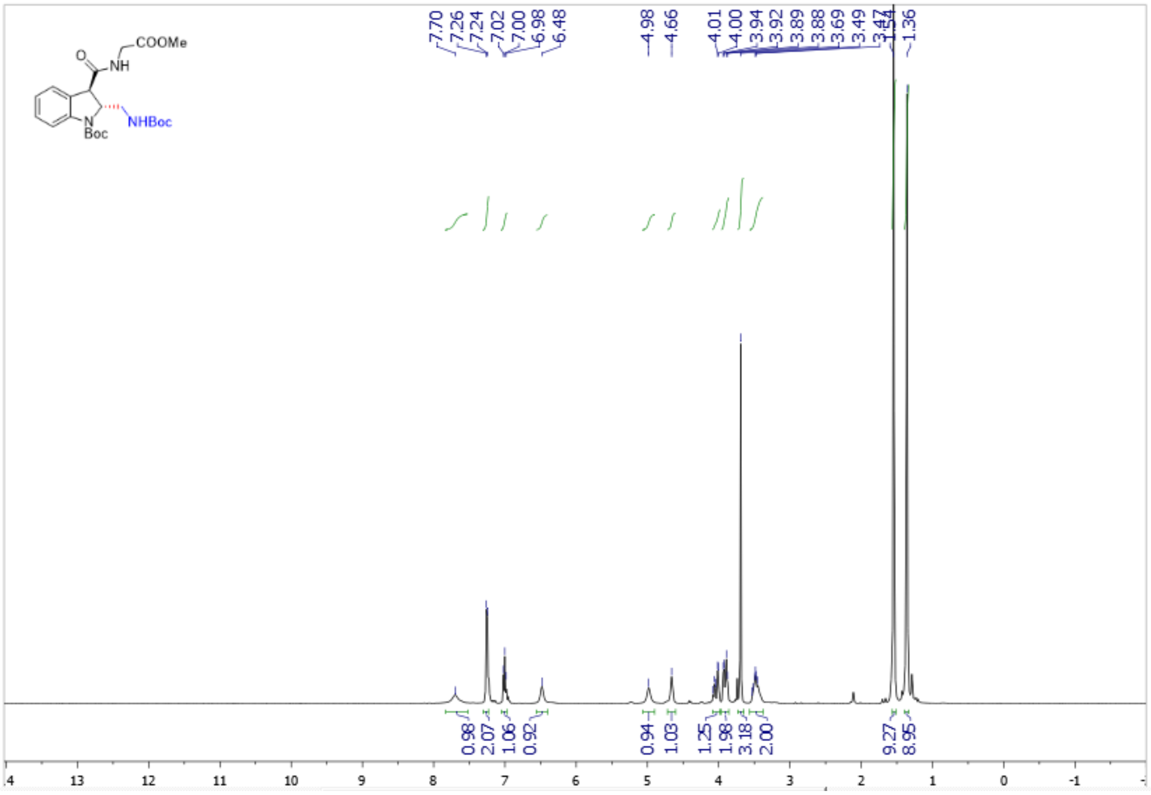

Supplementary Figure 57. ^{1}H NMR spectra of *trans*-**3g**

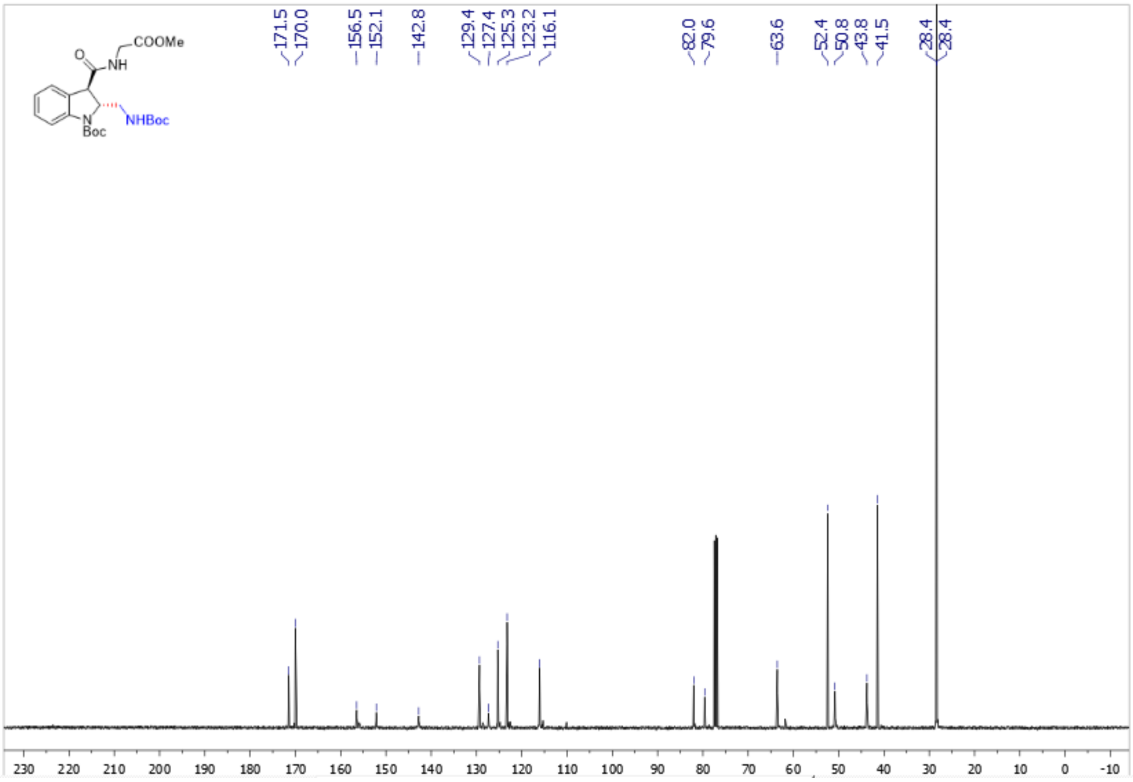

Supplementary Figure 58. ^{13}C NMR spectra of *trans*-**3g**

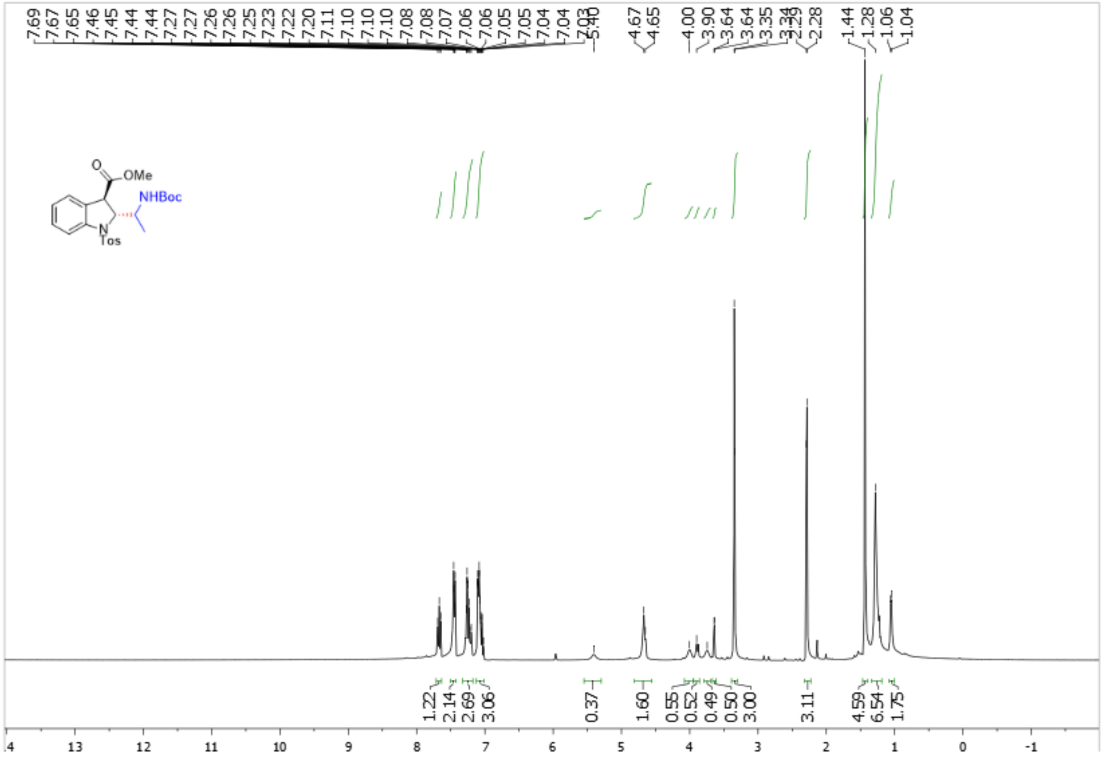

Supplementary Figure 59. ^{1}H NMR spectra of *trans*-**3h**

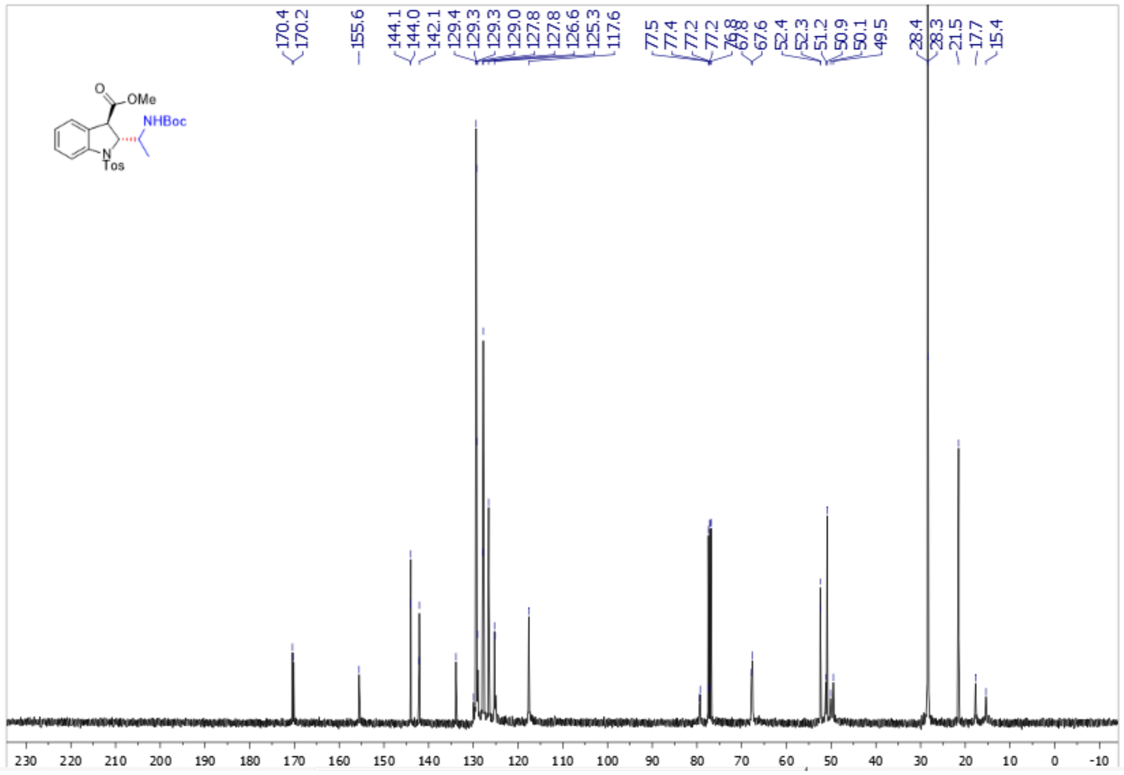

Supplementary Figure 60. ^{13}C NMR spectra of *trans*-**3h**

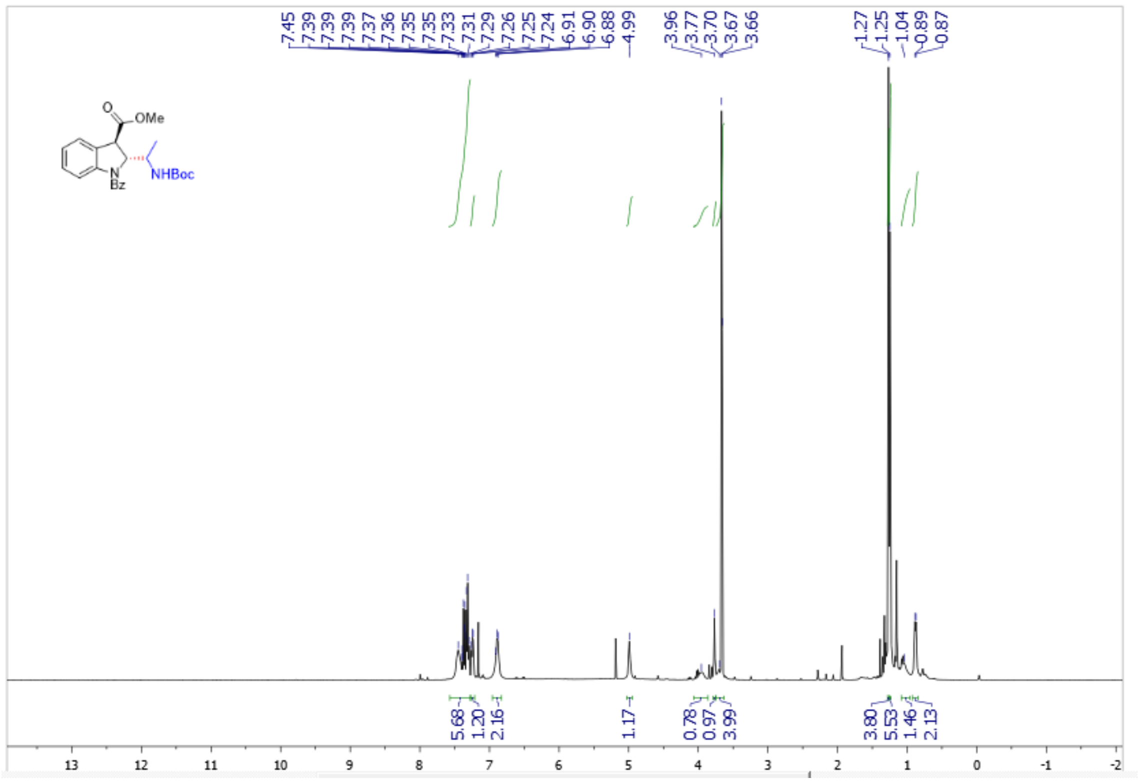

Supplementary Figure 61. ^{1}H NMR spectra of *trans*-**3i**

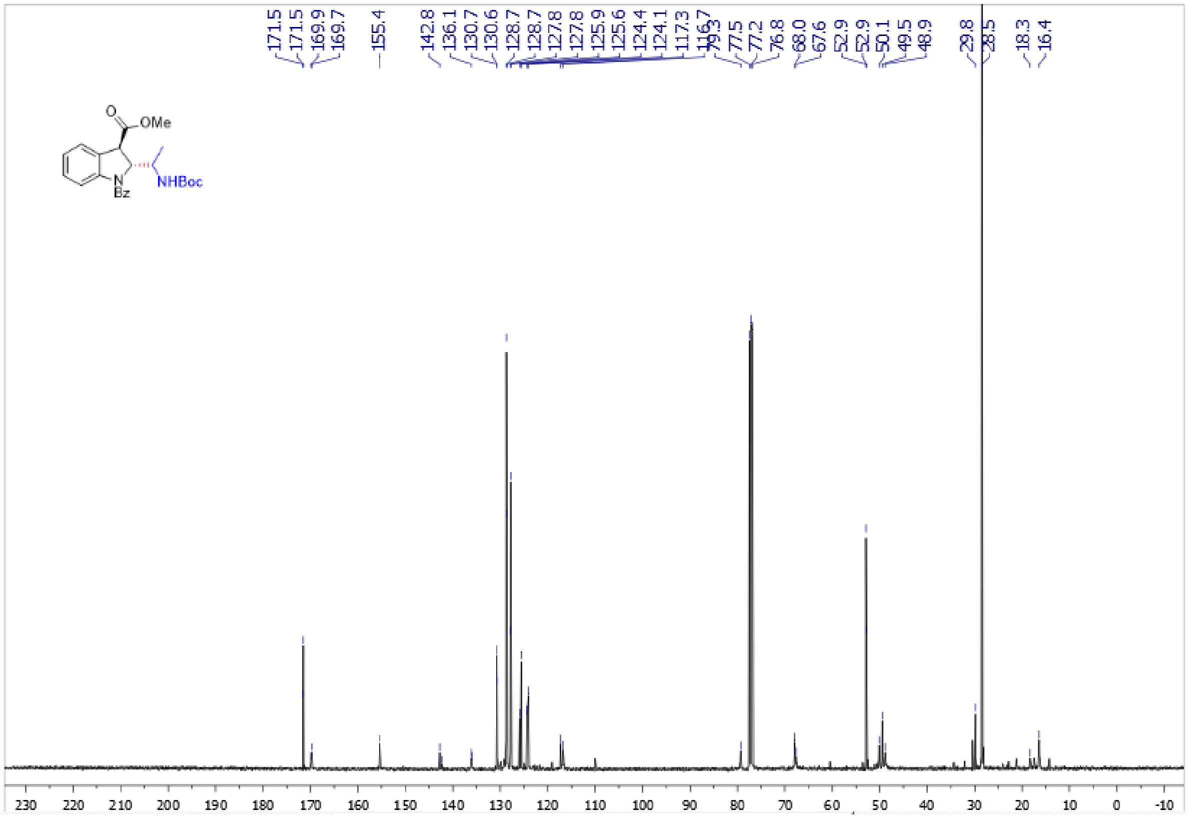

Supplementary Figure 62. ^{13}C NMR spectra of *trans*-**3i**

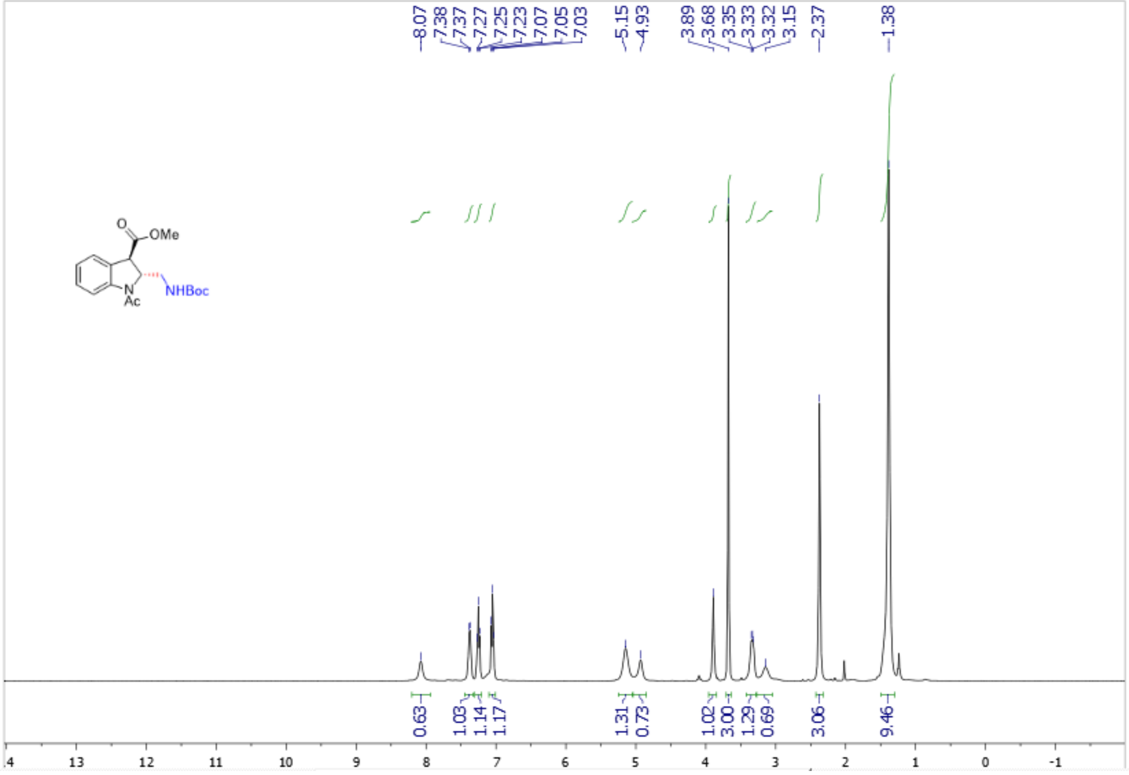

Supplementary Figure 63. ^{1}H NMR spectra of *trans*-**3j**

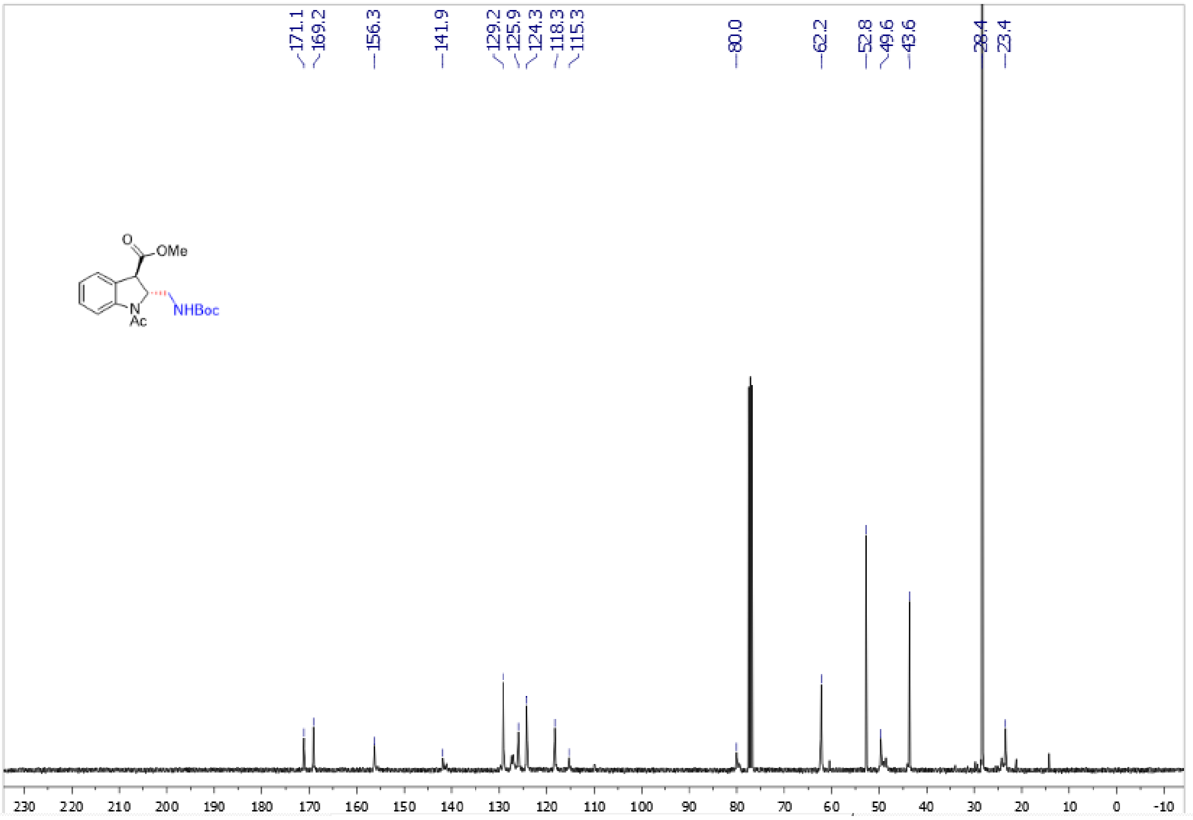

Supplementary Figure 64. ^{13}C NMR spectra of *trans*-**3j**

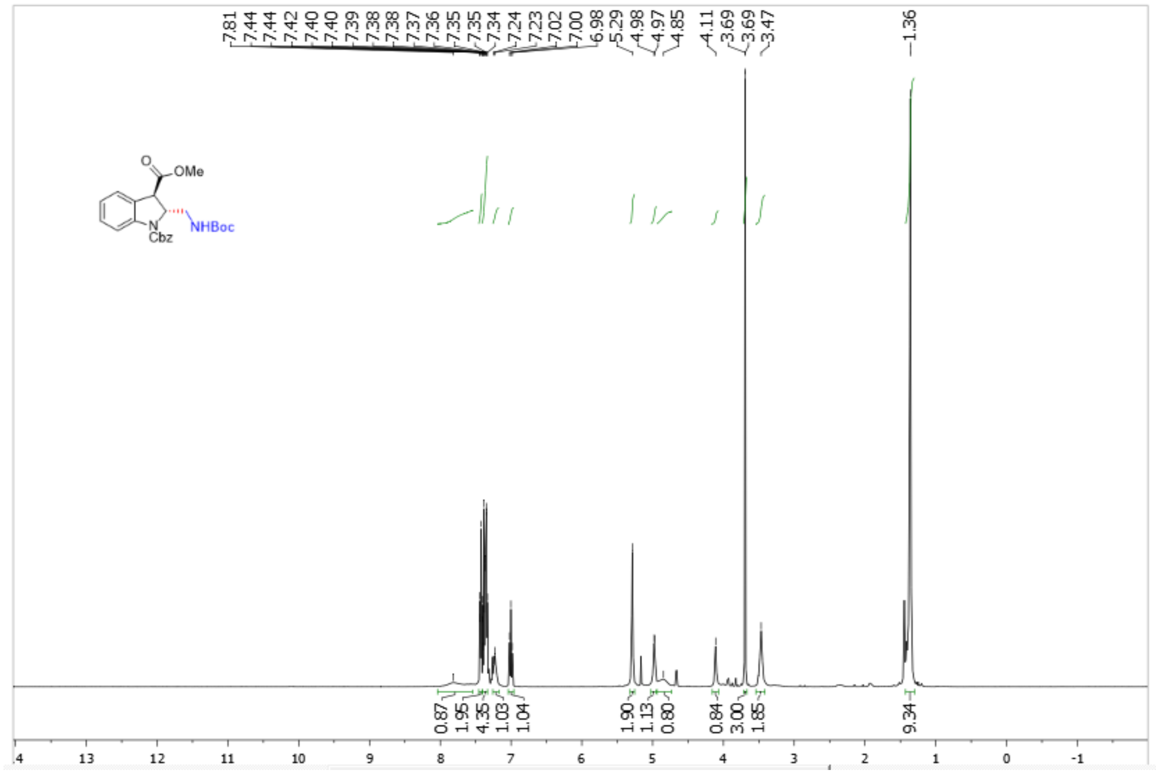

Supplementary Figure 65. ^{1}H NMR spectra of *trans*-**3k**

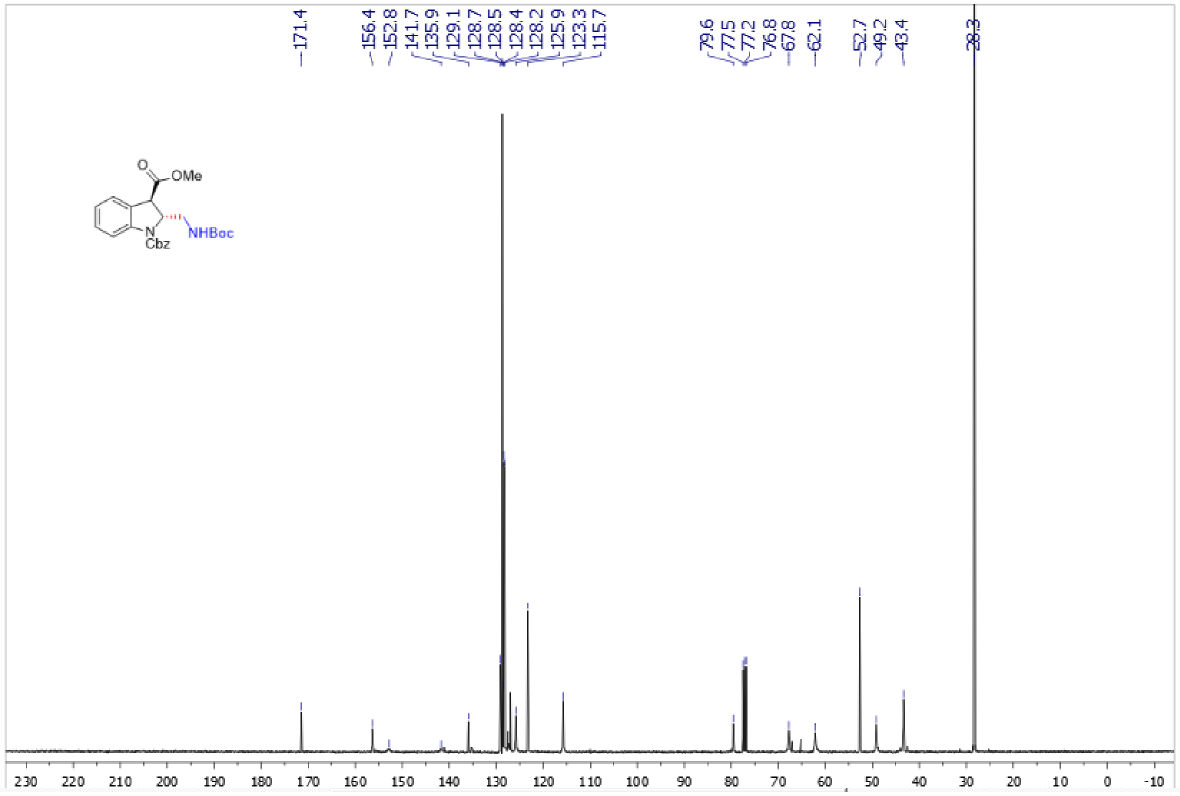

Supplementary Figure 66. ^{13}C NMR spectra of *trans*-**3k**

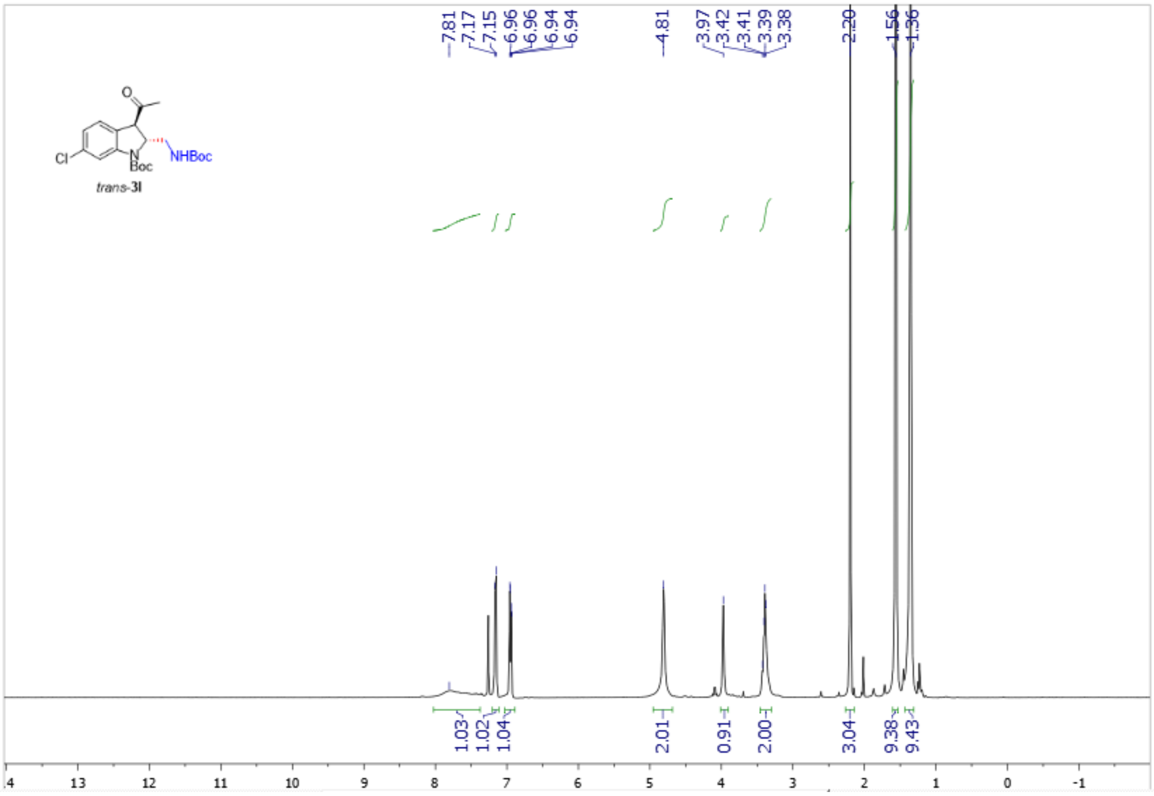

Supplementary Figure 67. ^{1}H NMR spectra of *trans*-**3l**

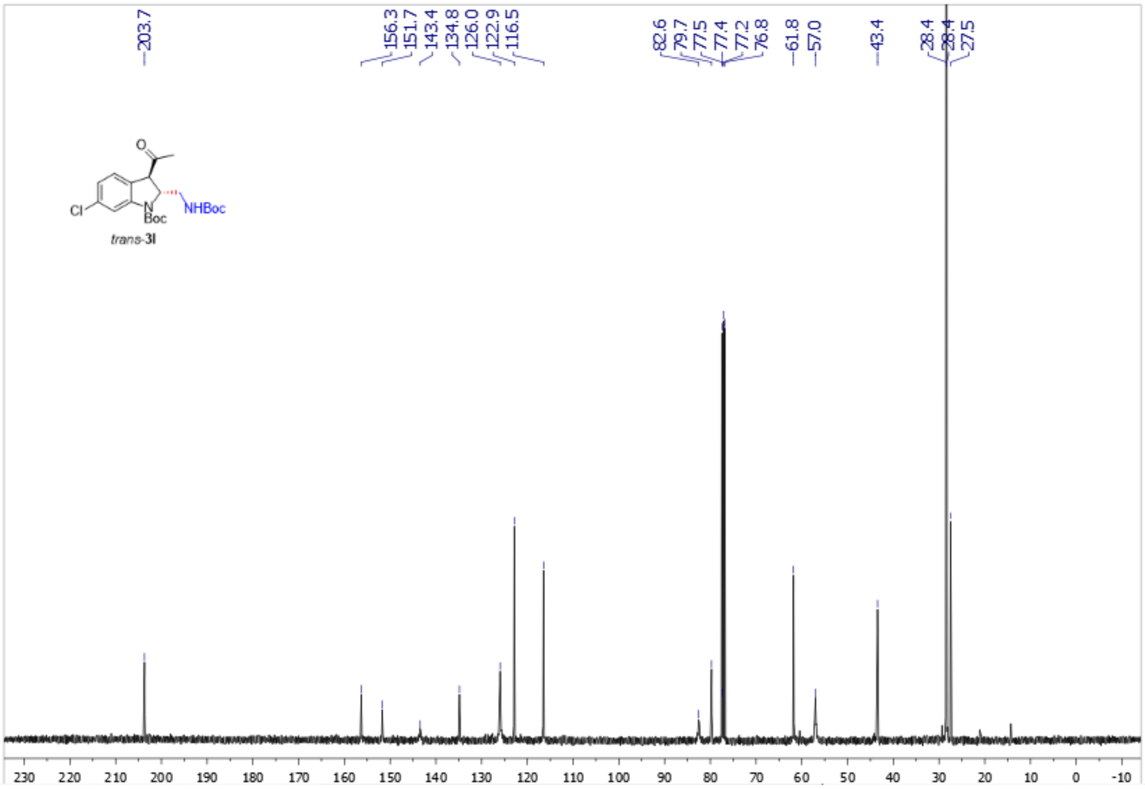

Supplementary Figure 68. ^{13}C NMR spectra of *trans*-**3l**

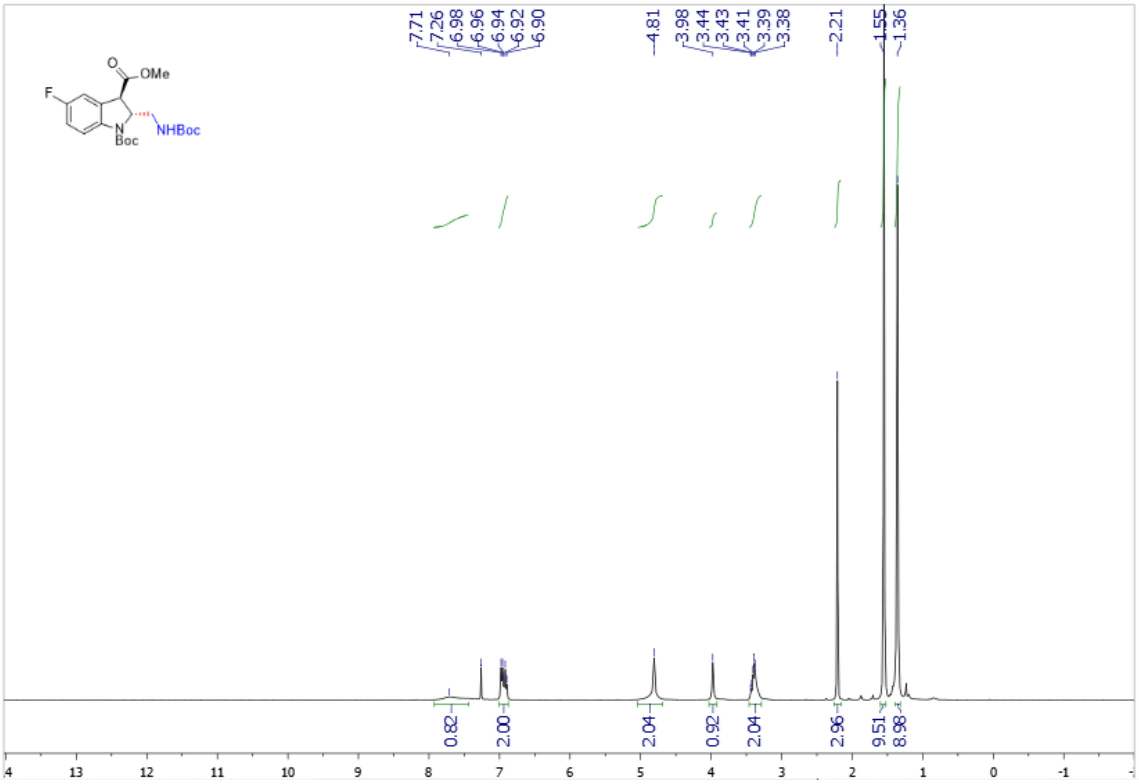

Supplementary Figure 69. ^{1}H NMR spectra of *trans*-**3m**

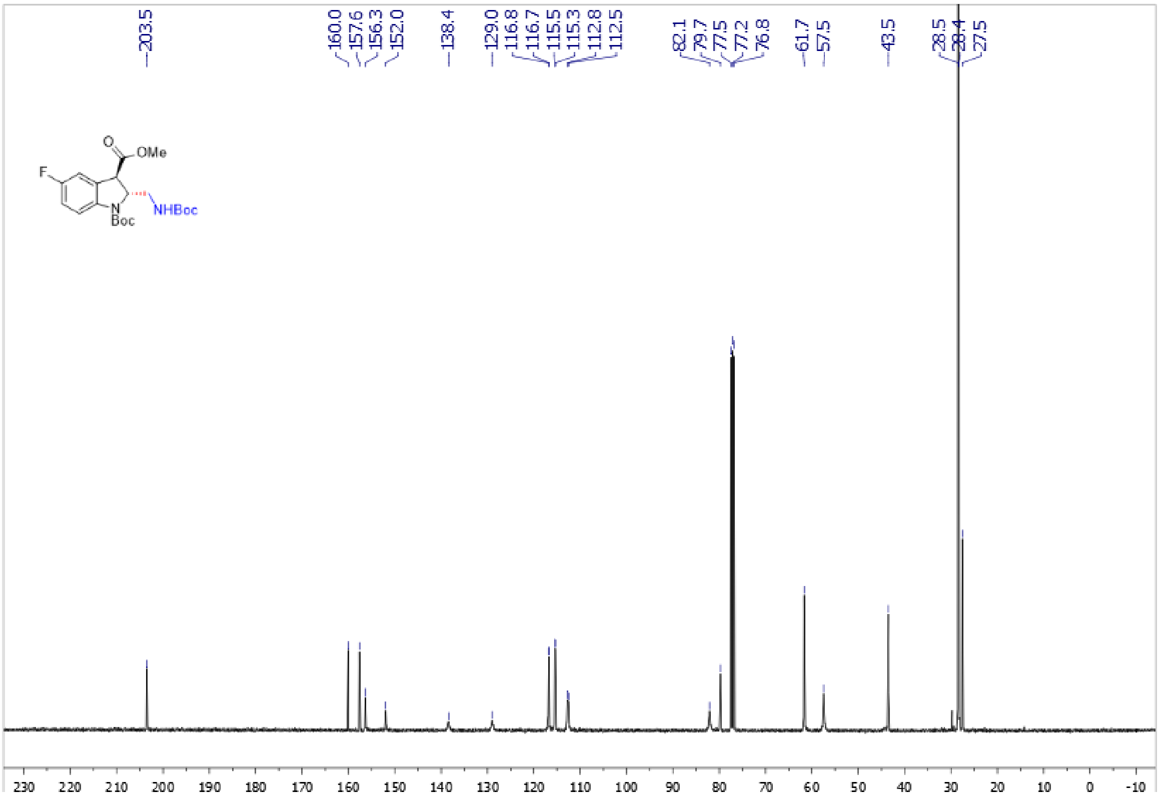

Supplementary Figure 70. ^{13}C NMR spectra of *trans*-**3m**

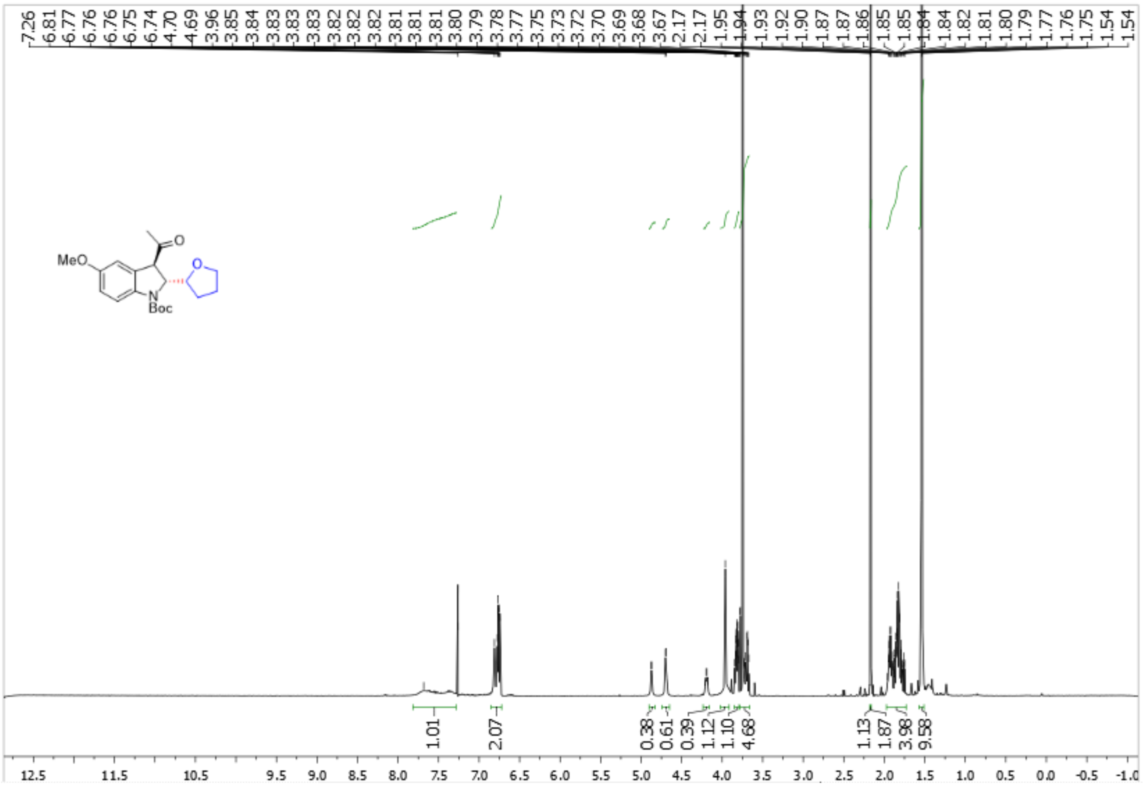

Supplementary Figure 71. ^{1}H NMR spectra of *trans*-**3n**

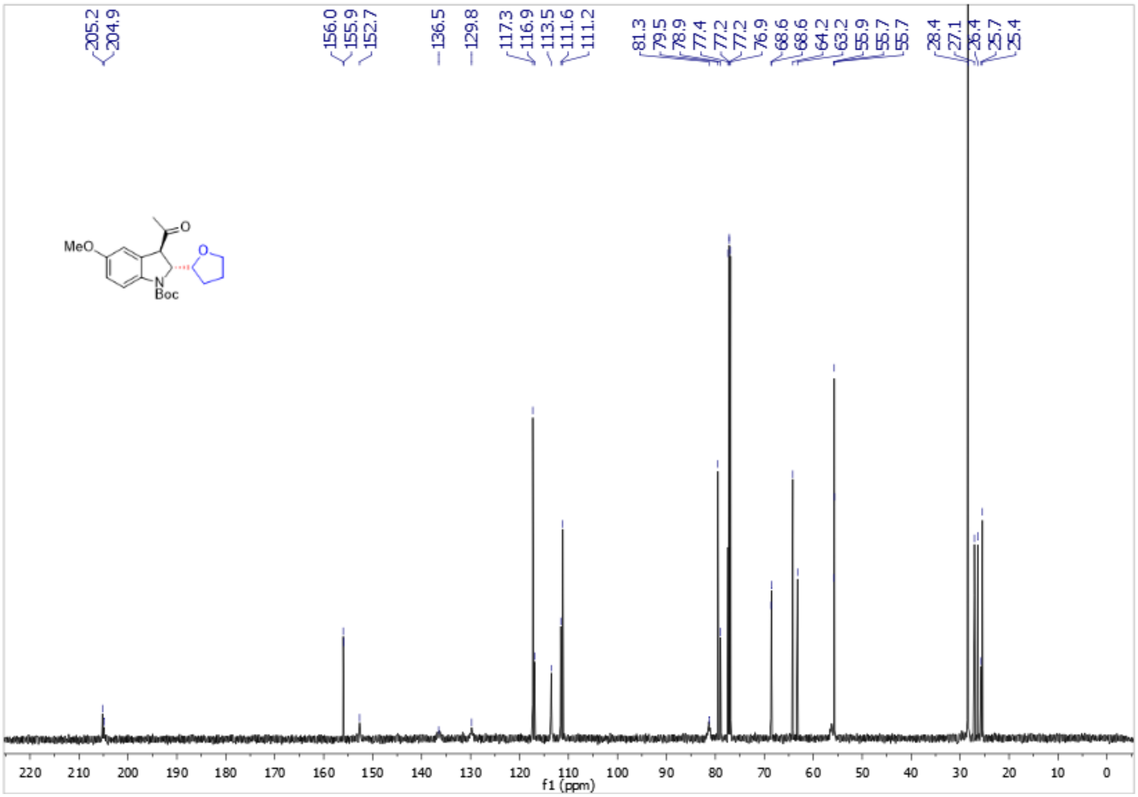

Supplementary Figure 72. ^{13}C NMR spectra of *trans*-**3n**

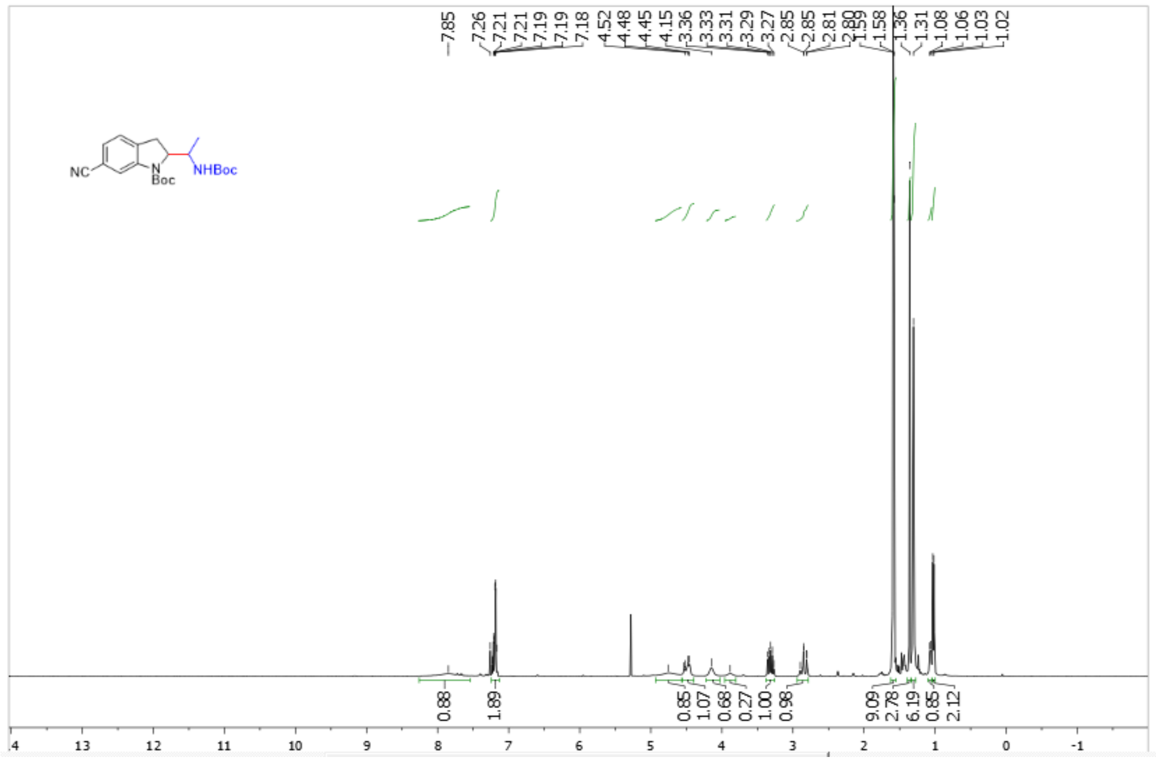

Supplementary Figure 73. ^{1}H NMR spectra of **3o**

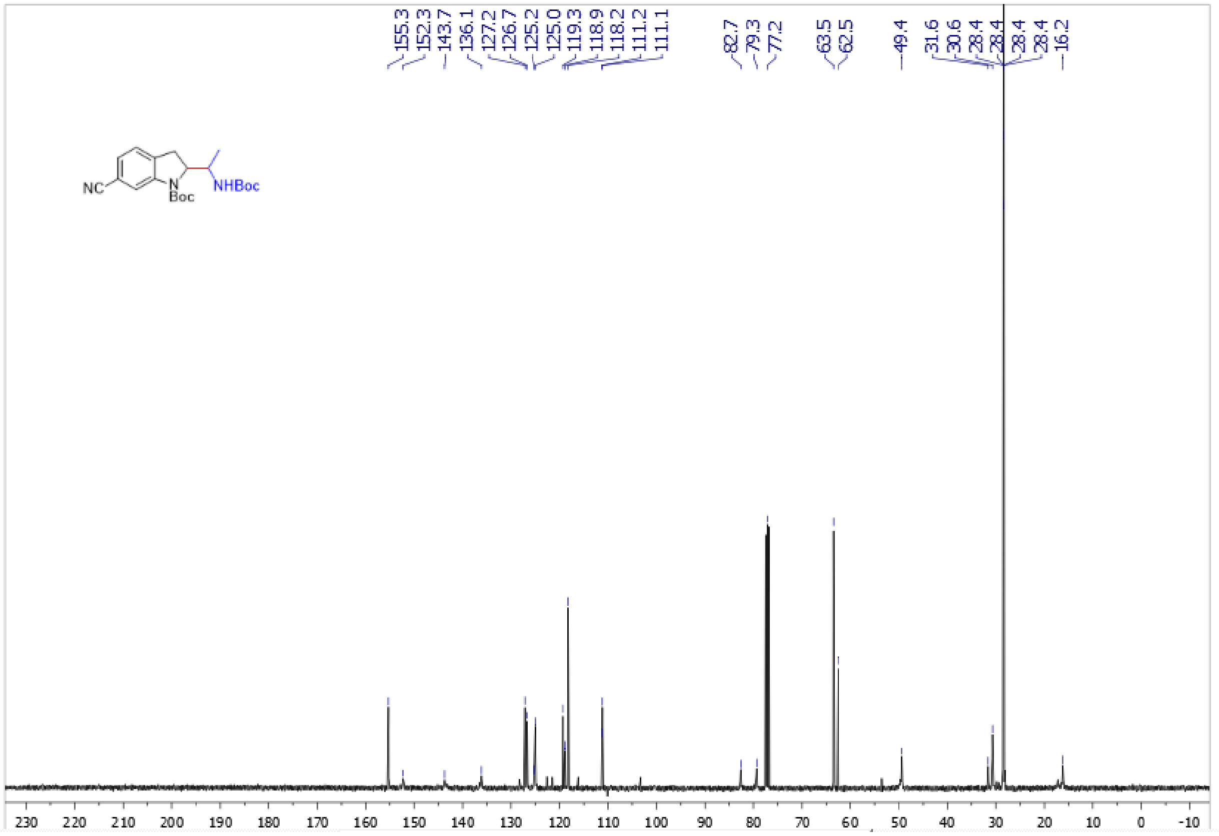

Supplementary Figure 74. ^{13}C NMR spectra of **3o**

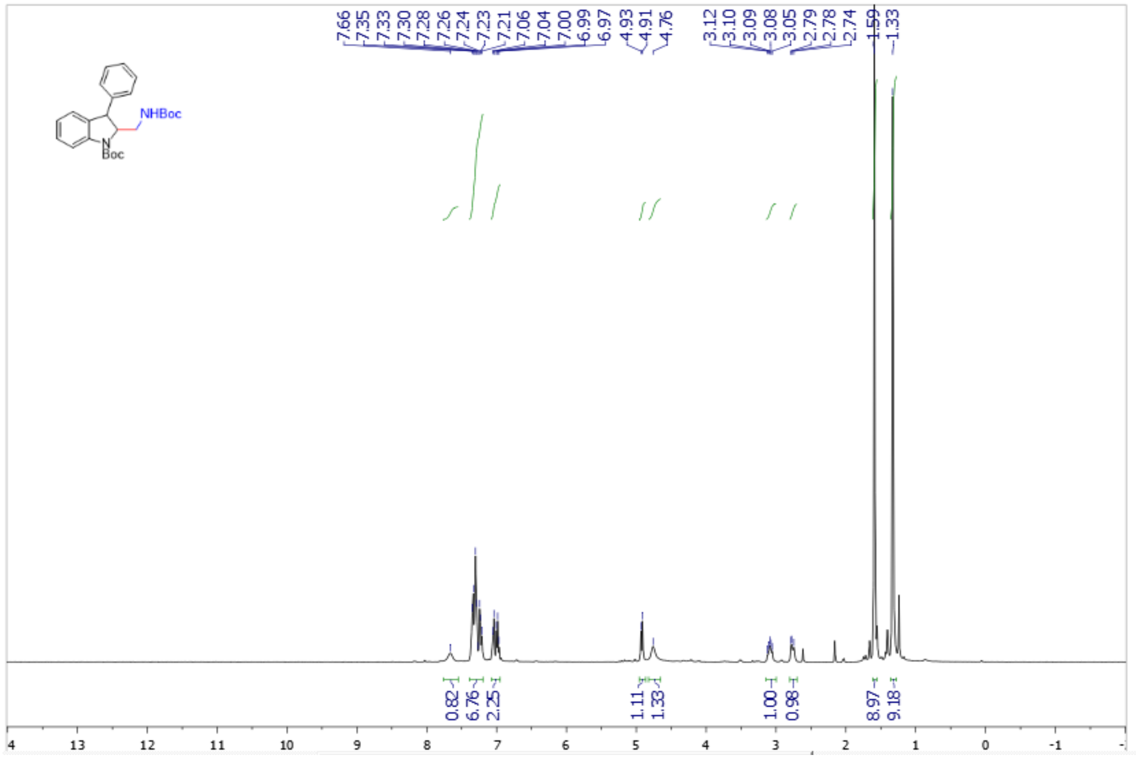

Supplementary Figure 75. ^{1}H NMR spectra of **3p–isomer-a**

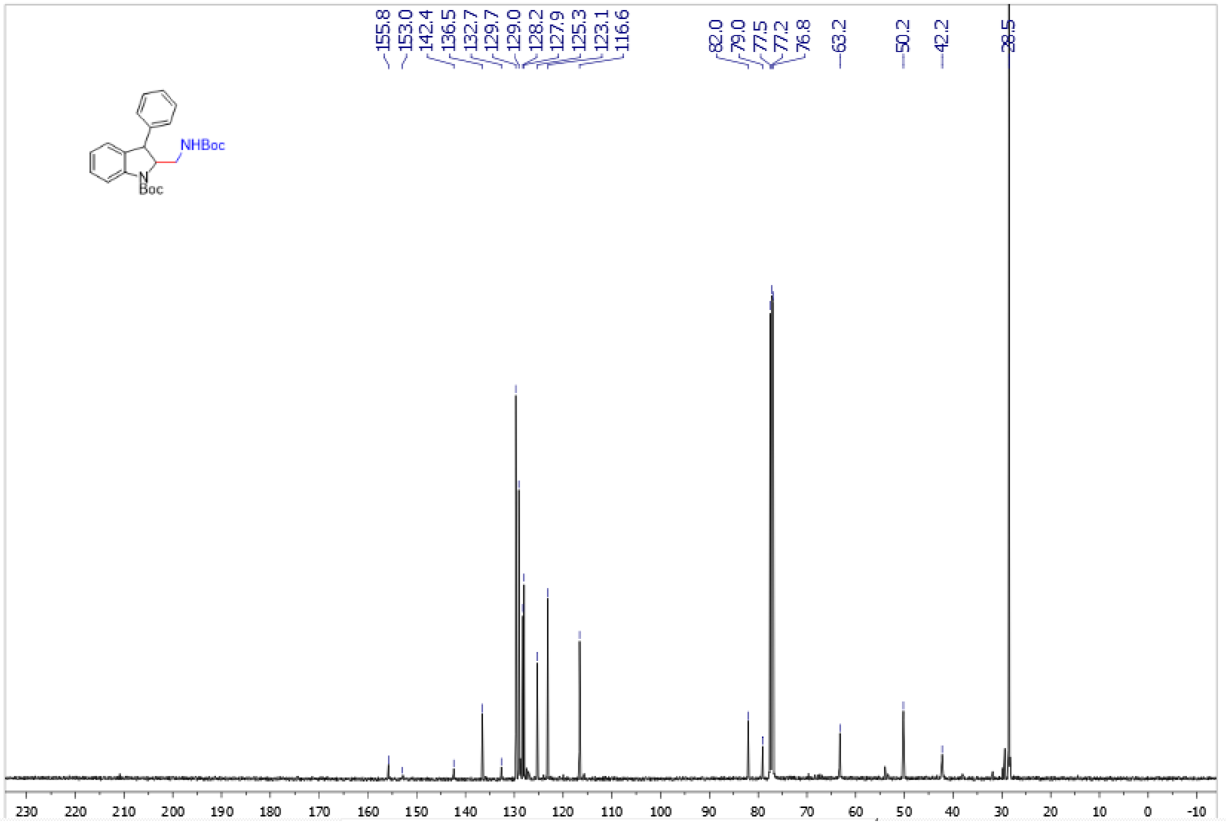

Supplementary Figure 76. ^{13}C NMR spectra of **3p–isomer-a**

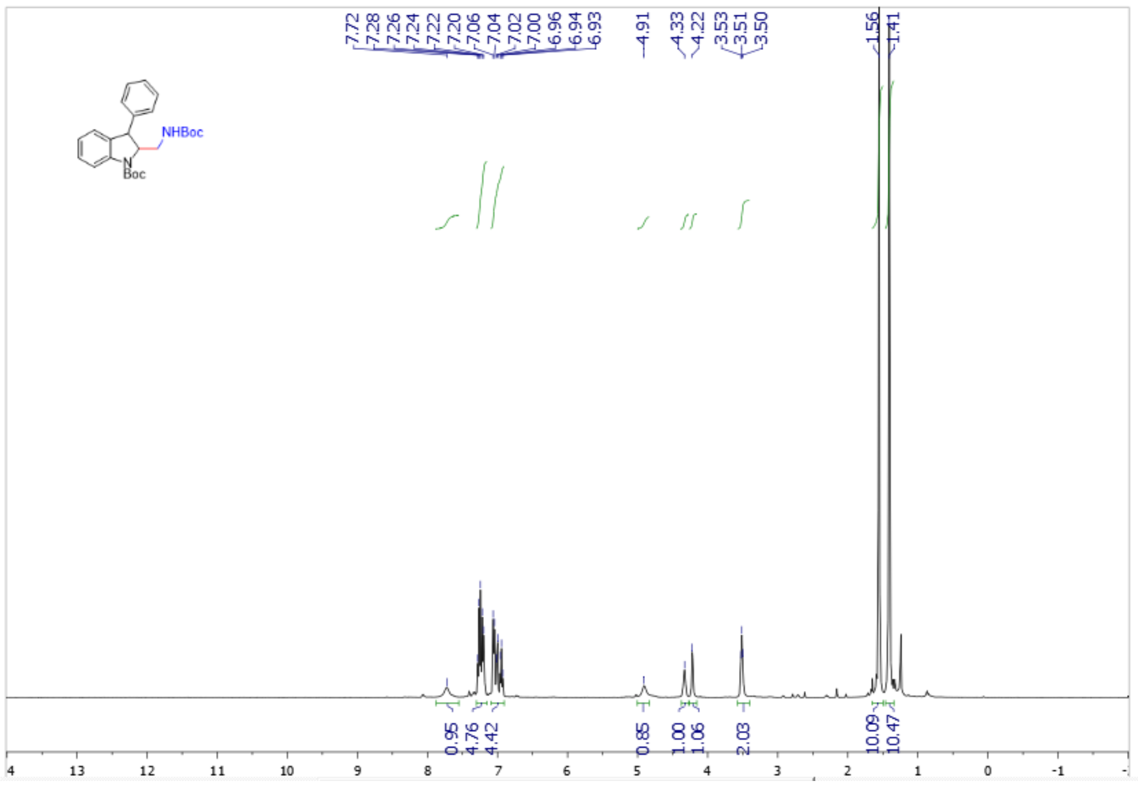

Supplementary Figure 77. ^{1}H NMR spectra of **3p–isomer-b**

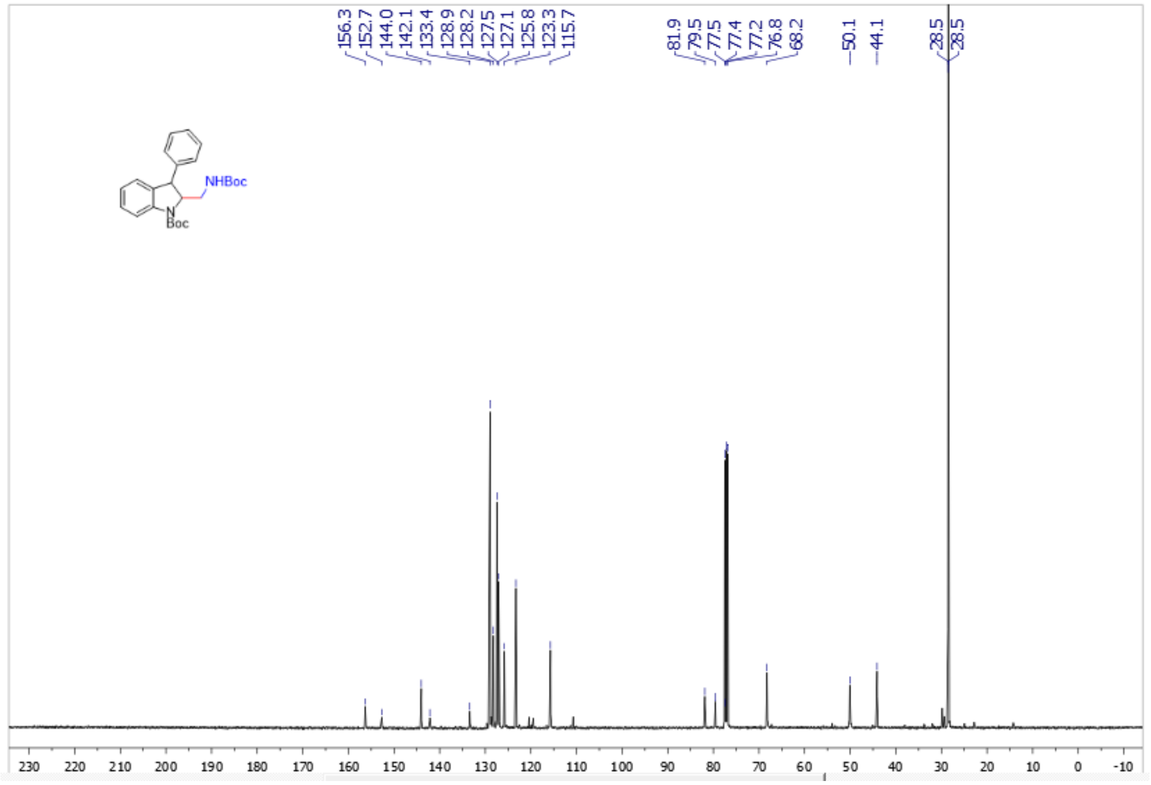

Supplementary Figure 78. ^{13}C NMR spectra of **3p–isomer-b**

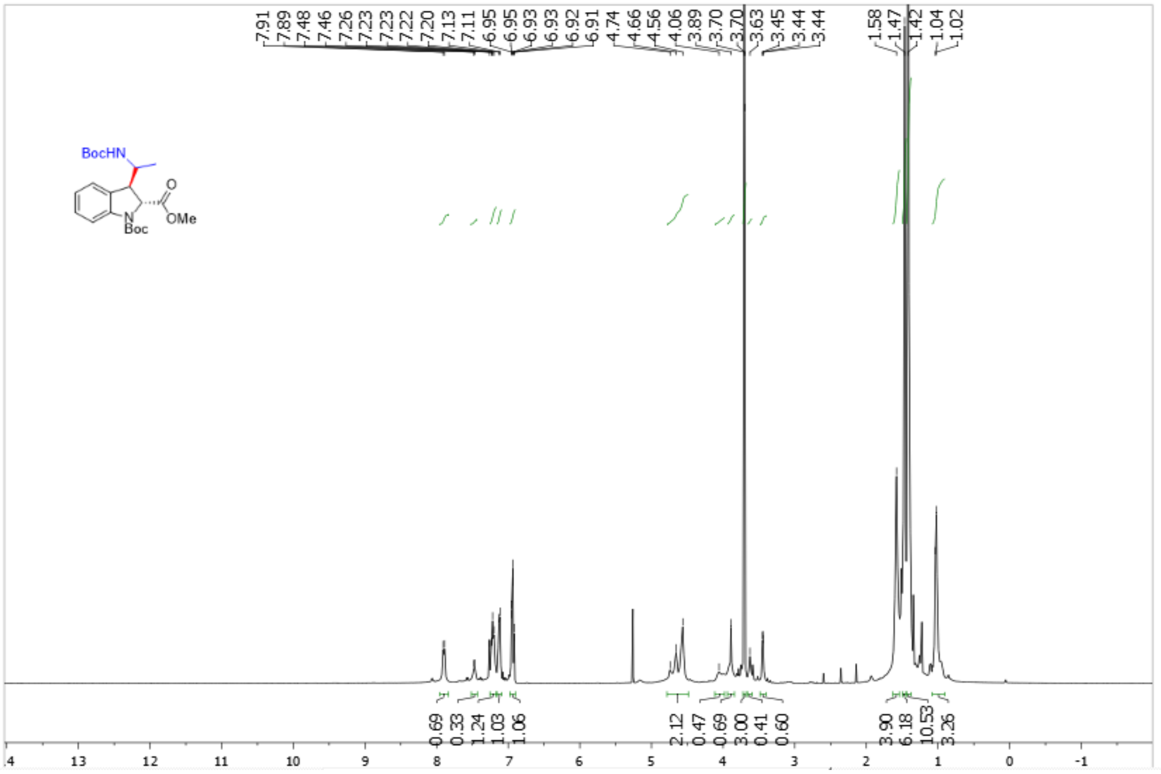

Supplementary Figure 79. ^{1}H NMR spectra of *trans*-**3q**

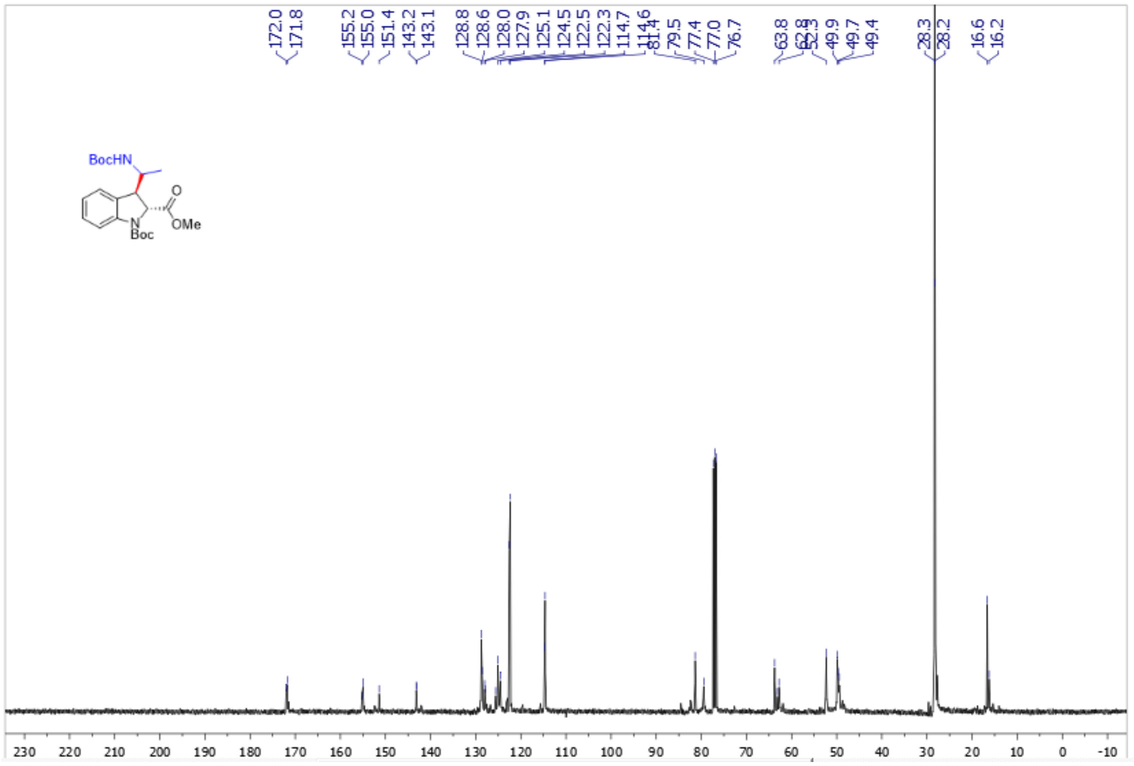

Supplementary Figure 80. ^{13}C NMR spectra of *trans*-**3q**

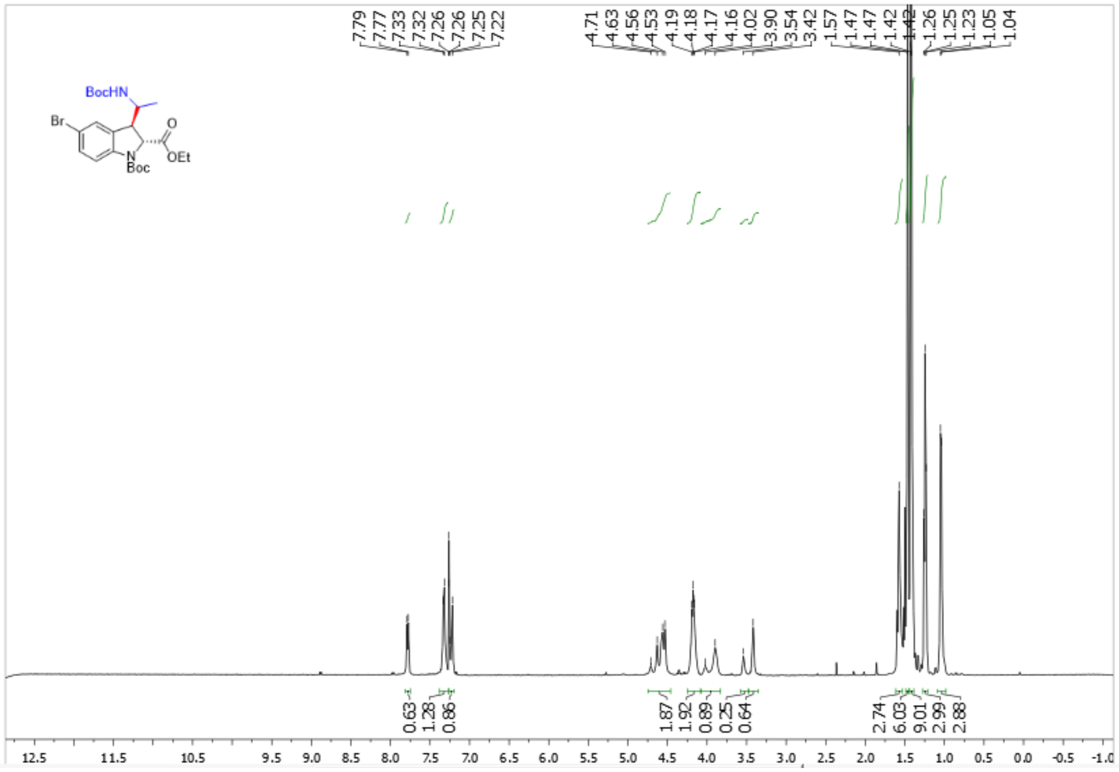

Supplementary Figure 81. ^{1}H NMR spectra of *trans*-**3r**

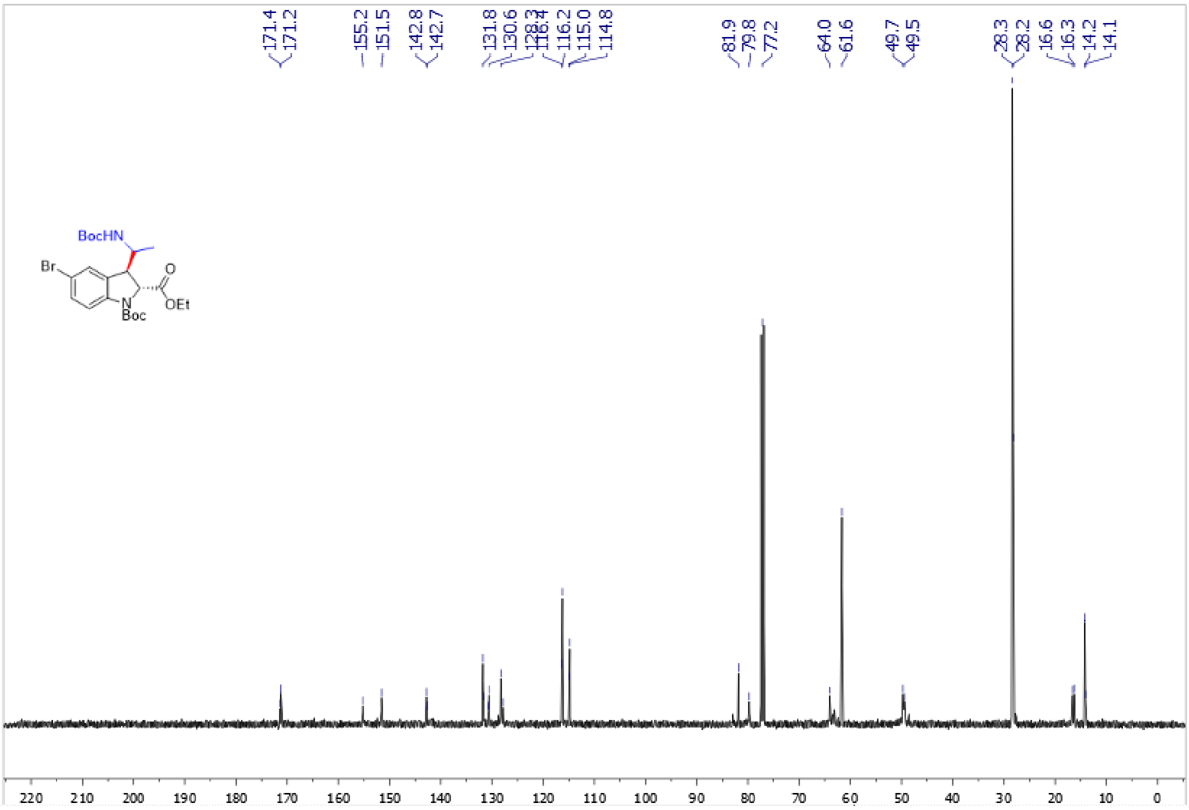

Supplementary Figure 82. ^{13}C NMR spectra of *trans*-**3r**

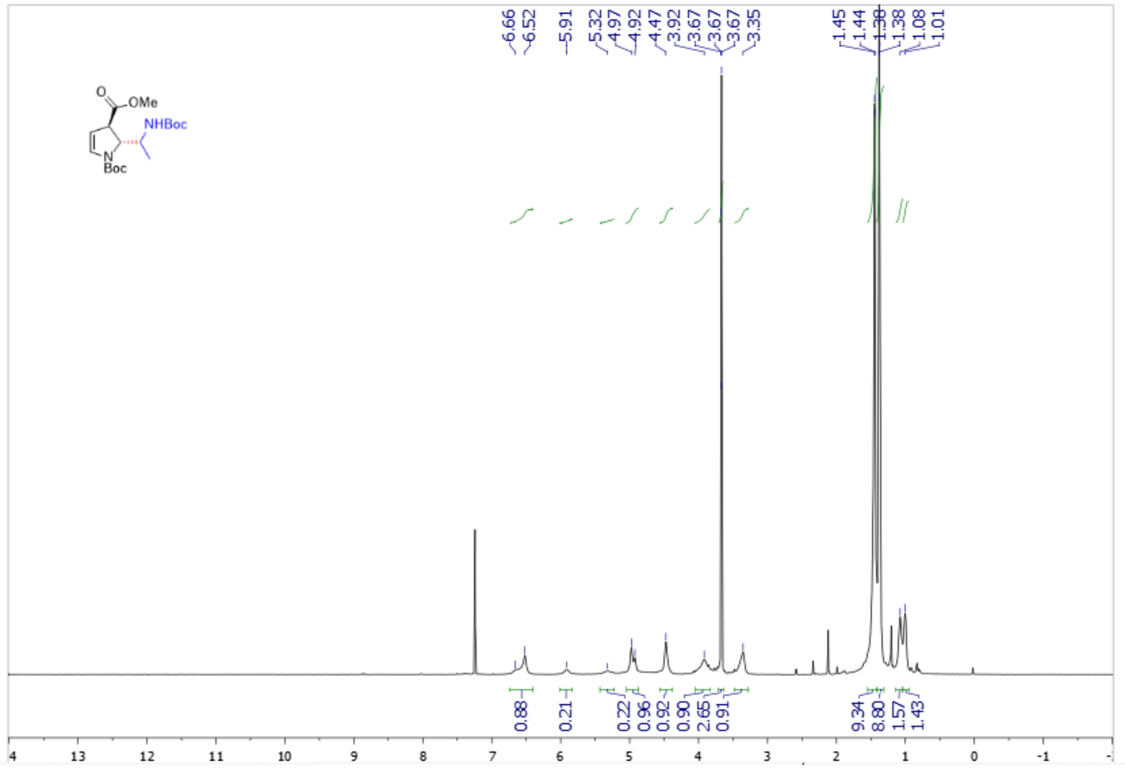

Supplementary Figure 83. ^{1}H NMR spectra of *trans*-**3s**

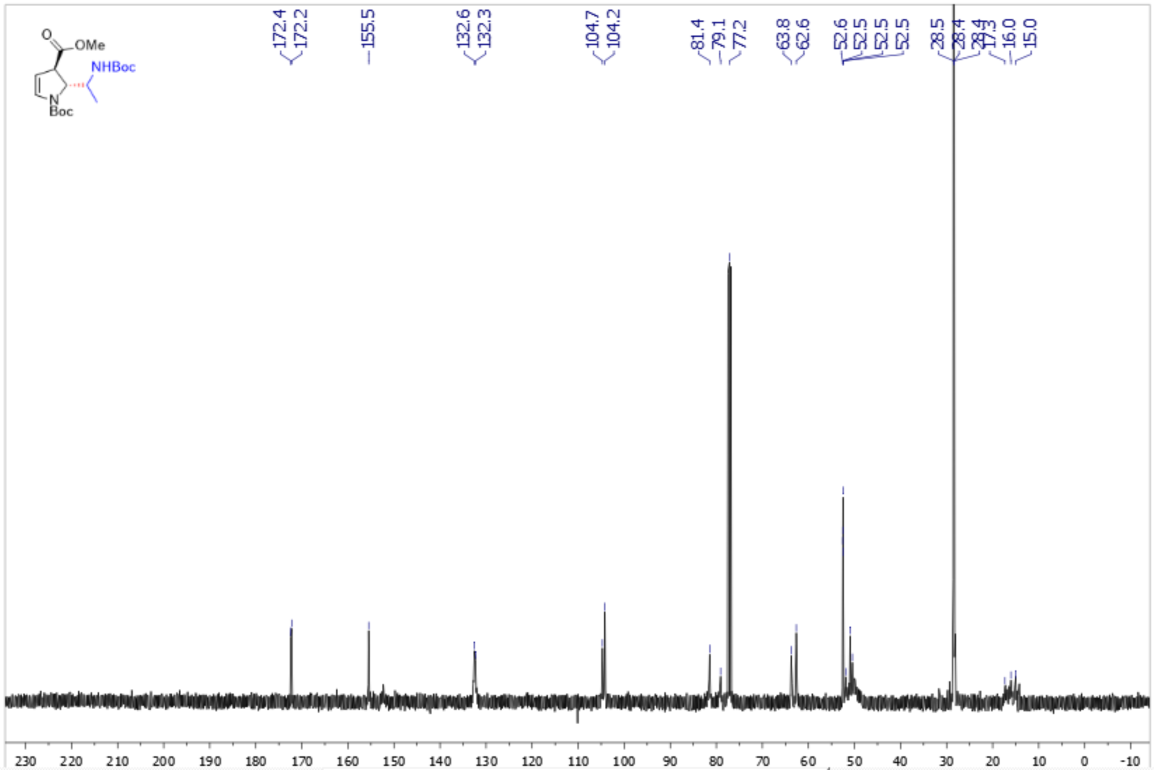

Supplementary Figure 84. ^{13}C NMR spectra of *trans*-**3s**

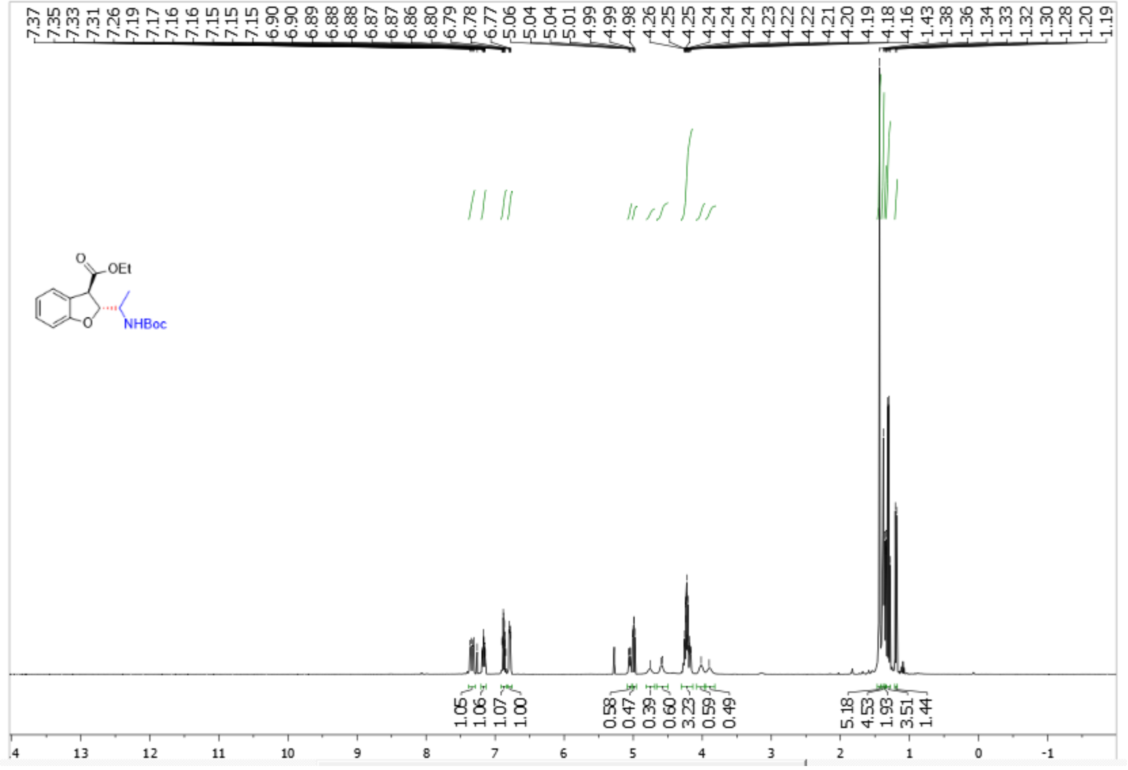

Supplementary Figure 85. ^{1}H NMR spectra of *trans*-**3t**

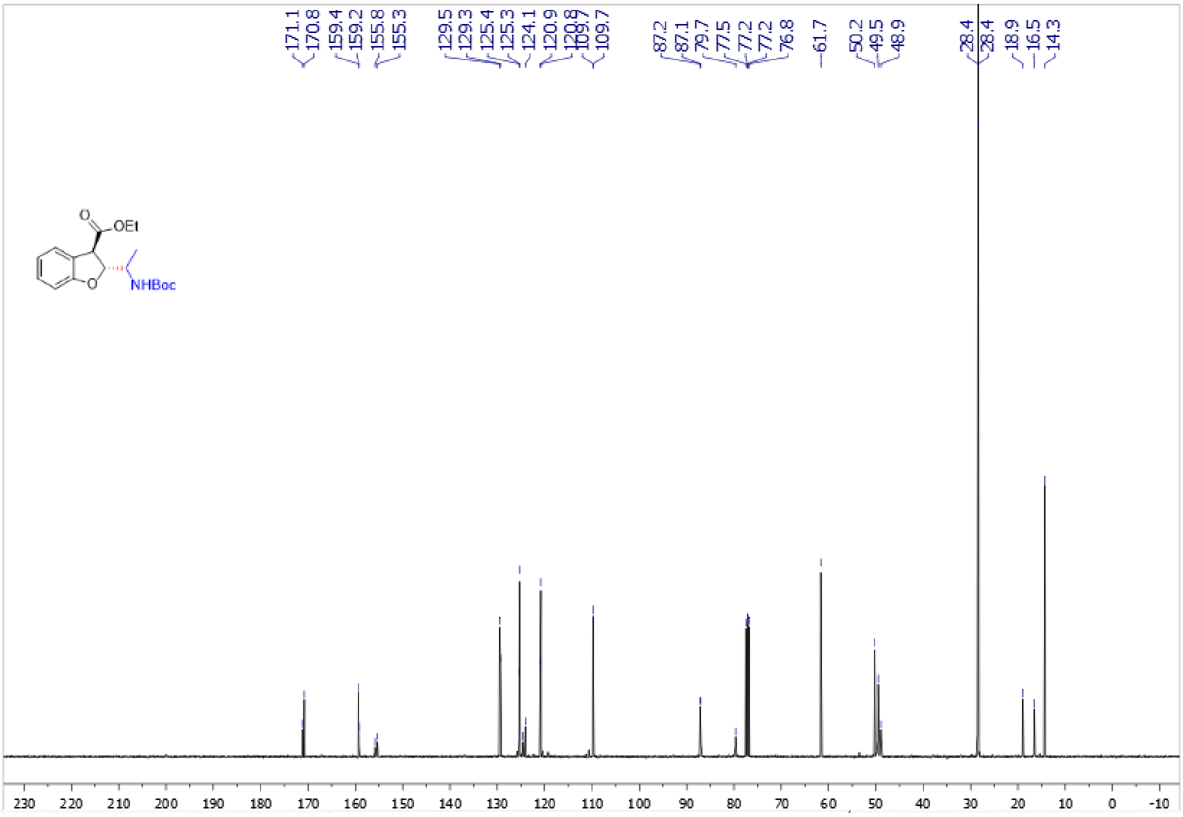

Supplementary Figure 86. ^{13}C NMR spectra of *trans*-**3t**

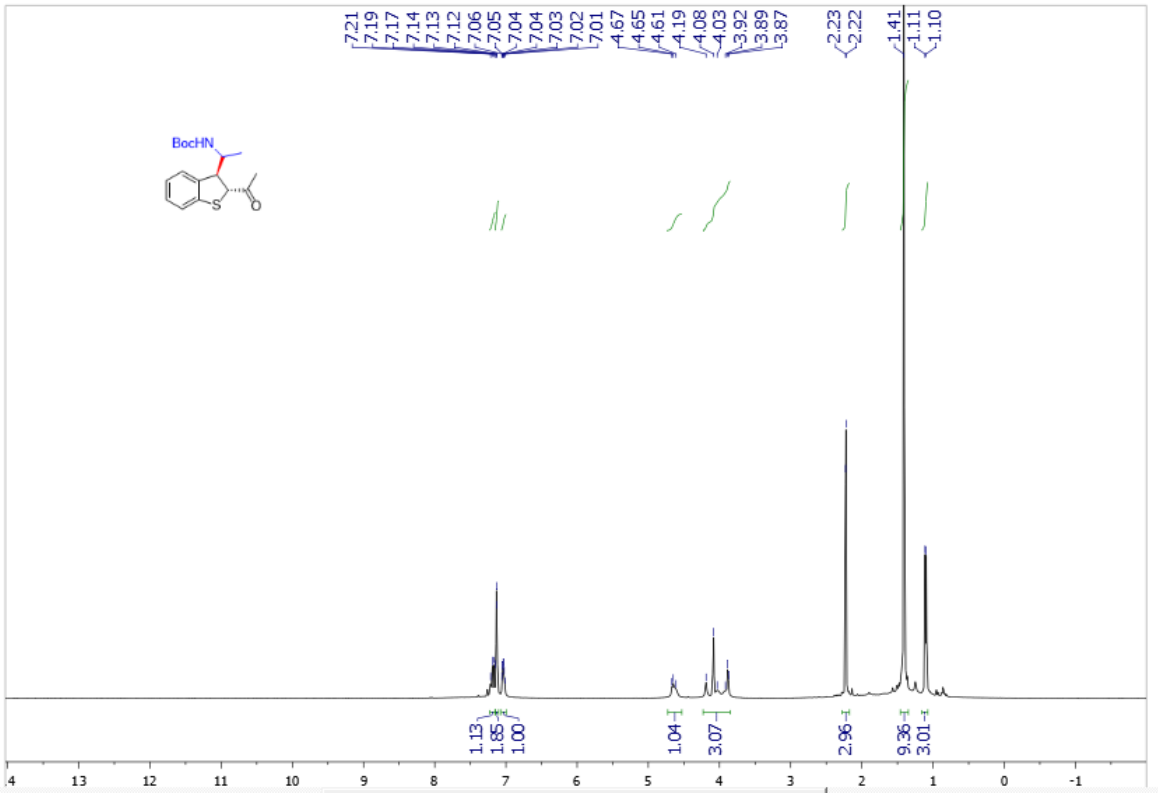

Supplementary Figure 87. ^{1}H NMR spectra of *trans*-**3u**

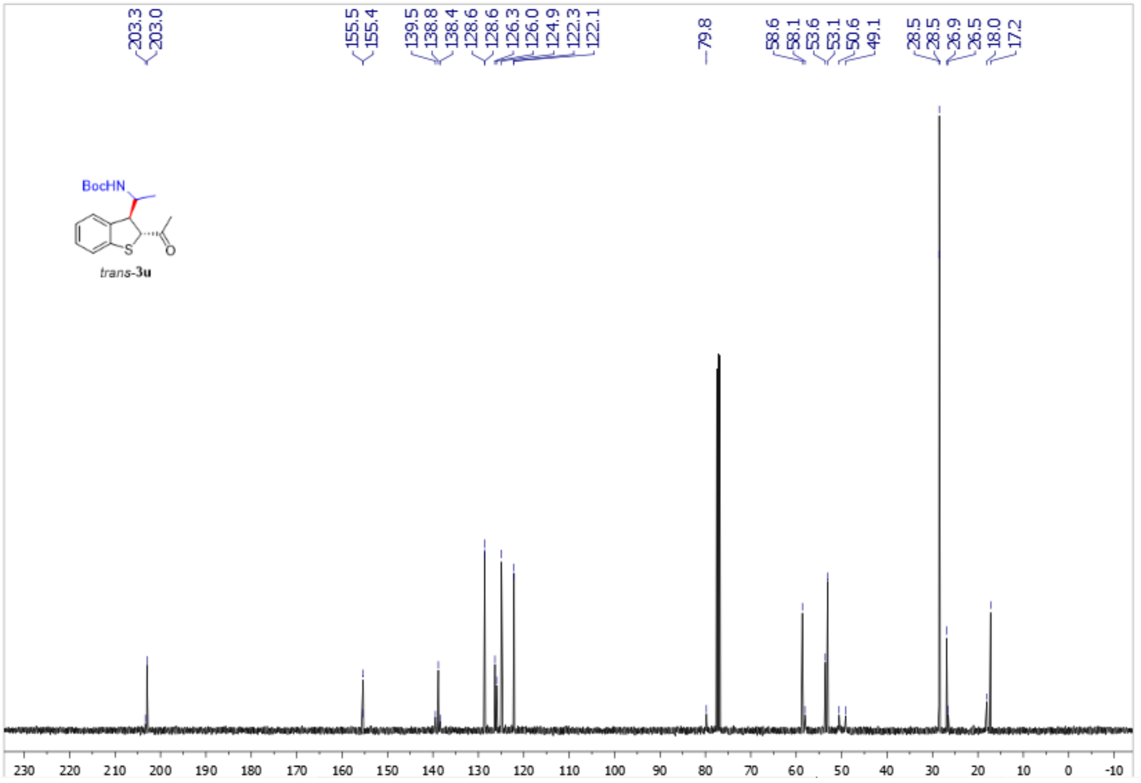

Supplementary Figure 88. ^{13}C NMR spectra of *trans*-**3u**

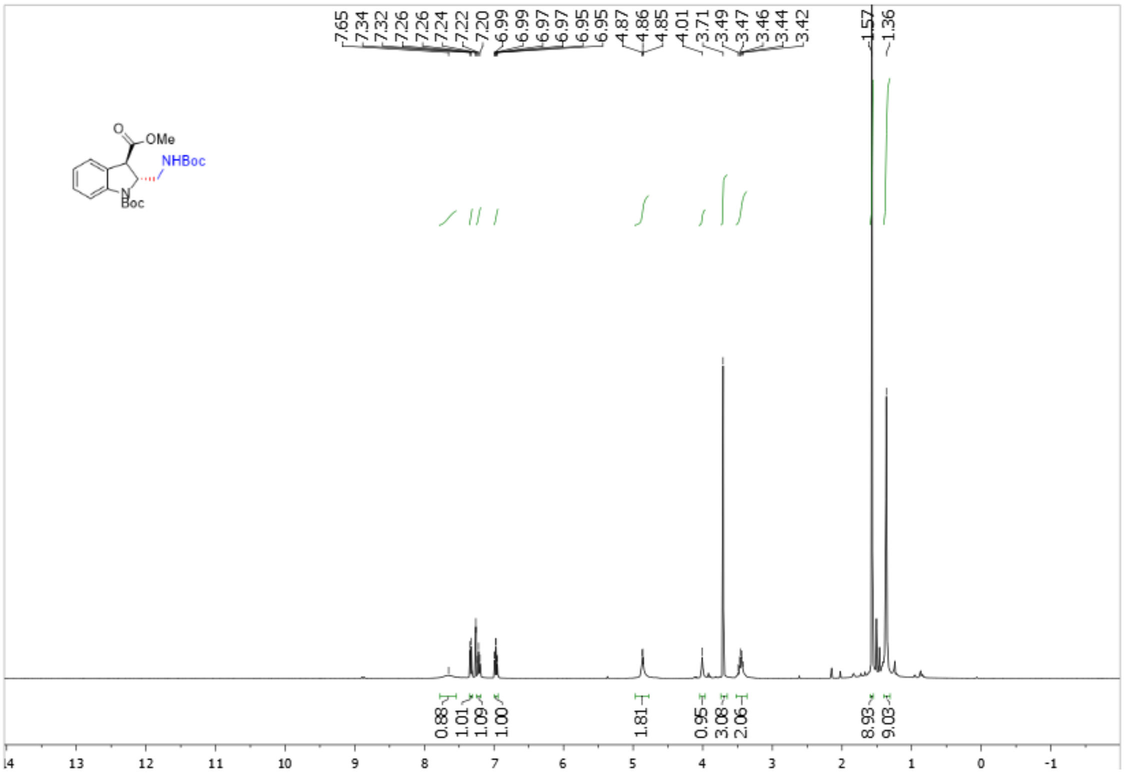

Supplementary Figure 89. ^{1}H NMR spectra of *trans*-**3v**

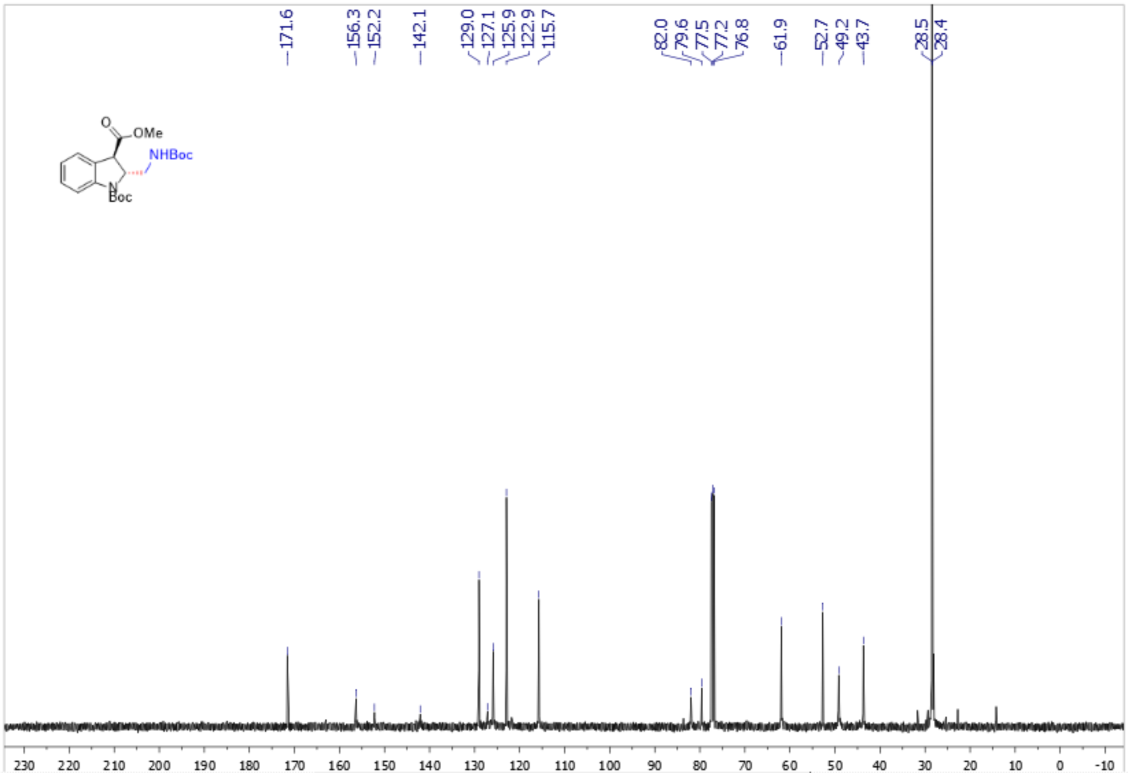

Supplementary Figure 90. ^{13}C NMR spectra of *trans*-**3v**

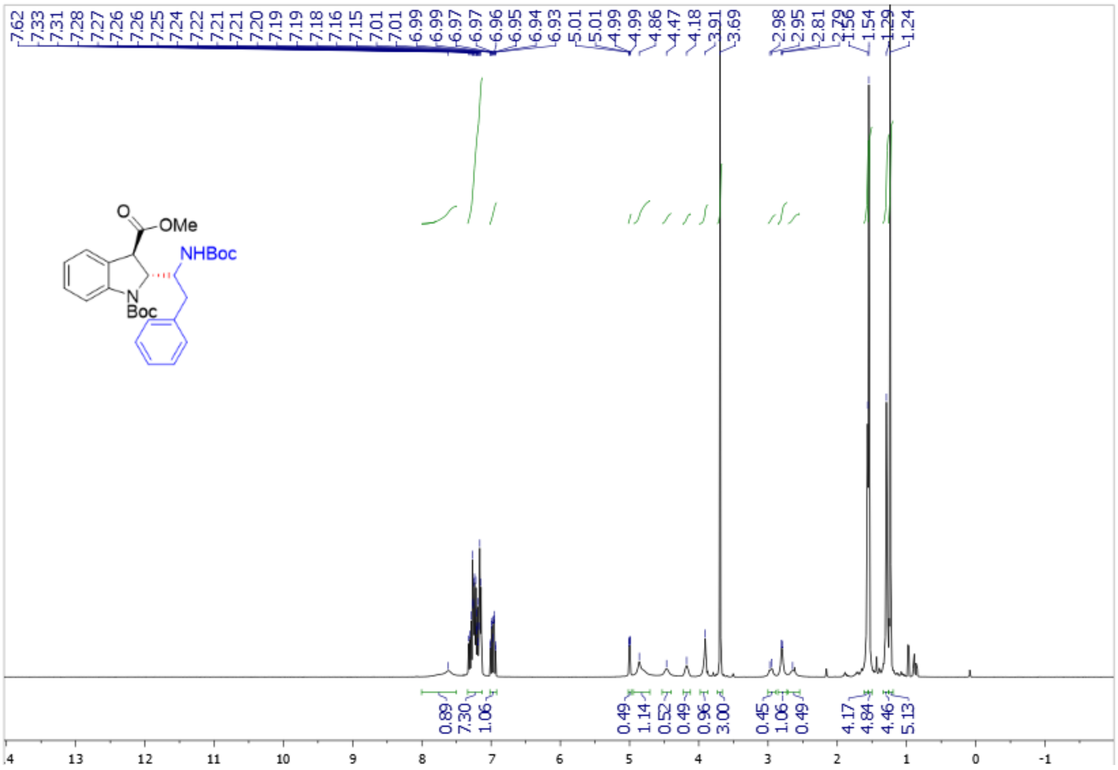

Supplementary Figure 91. ^{1}H NMR spectra of *trans*-**3w**

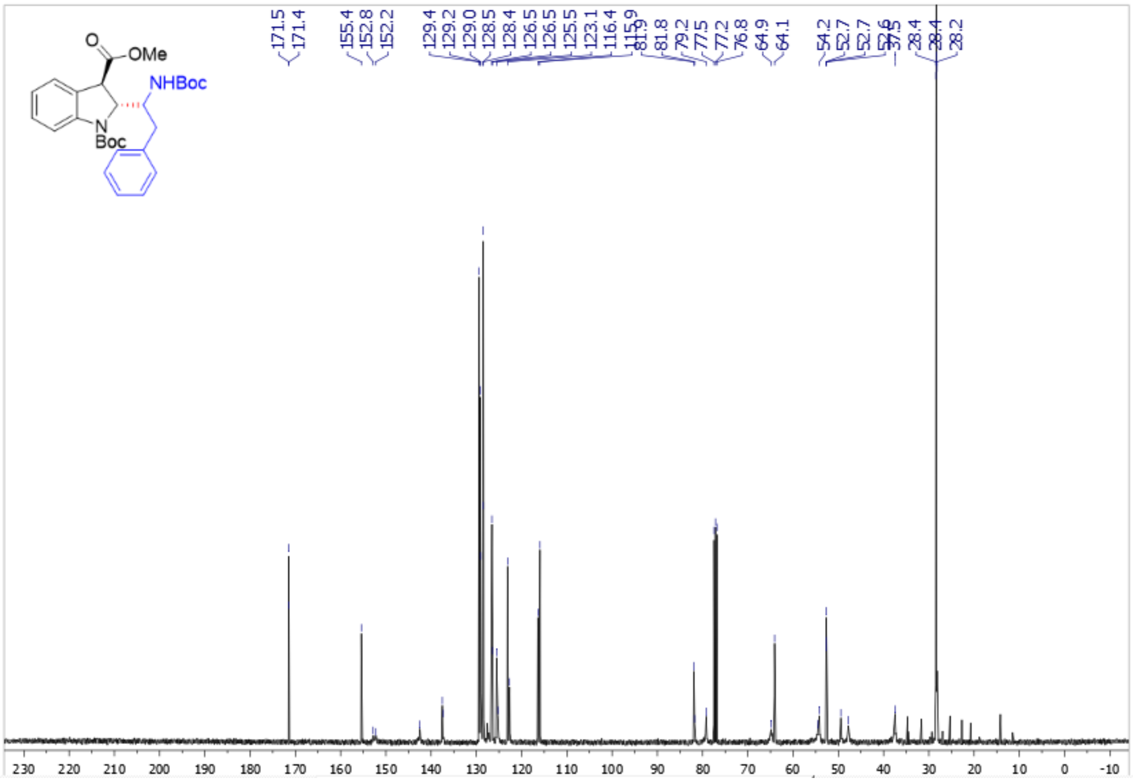

Supplementary Figure 92. ^{13}C NMR spectra of *trans*-**3w**

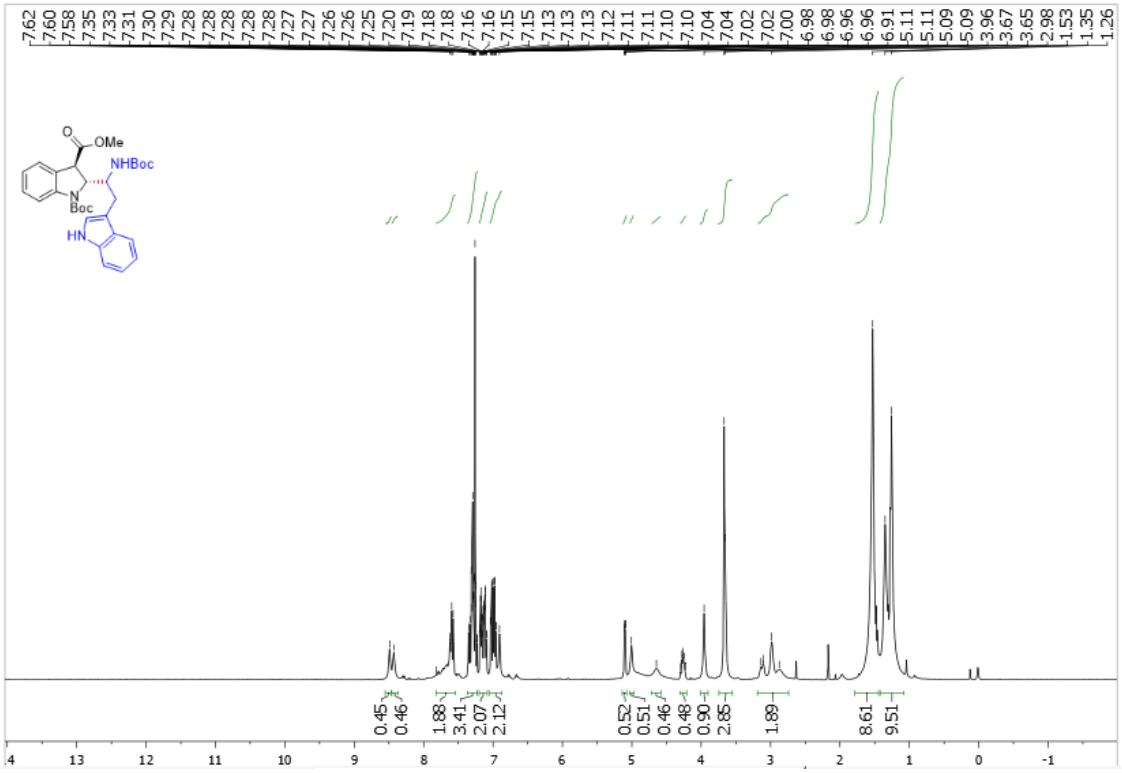

Supplementary Figure 93. ^{1}H NMR spectra of *trans*-**3x**

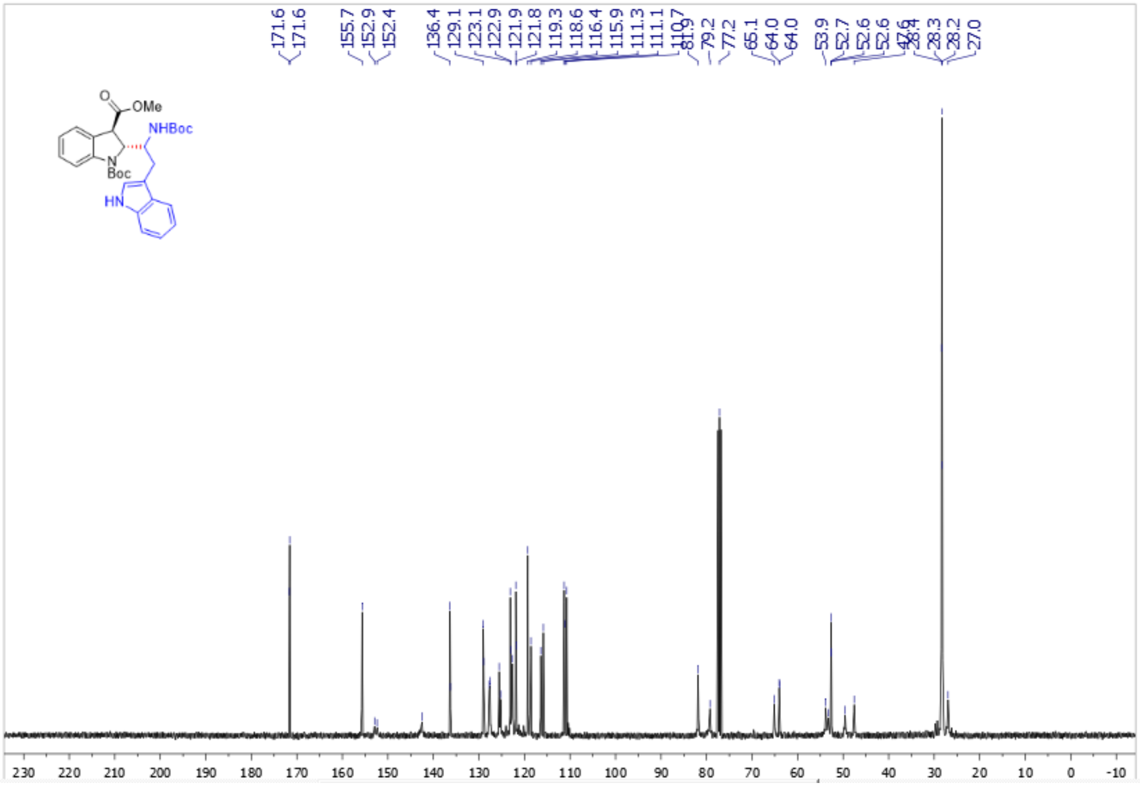

Supplementary Figure 94. ^{13}C NMR spectra of *trans*-**3x**

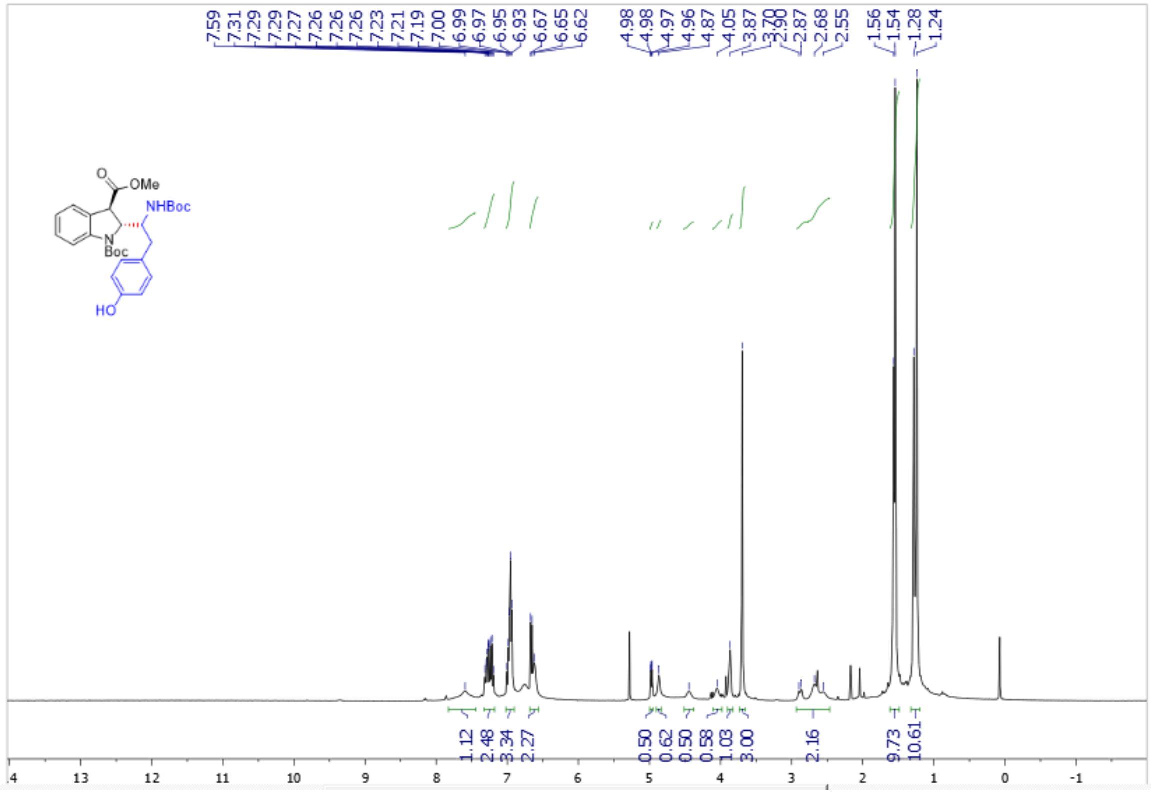

Supplementary Figure 95. ^{1}H NMR spectra of *trans*-**3y**

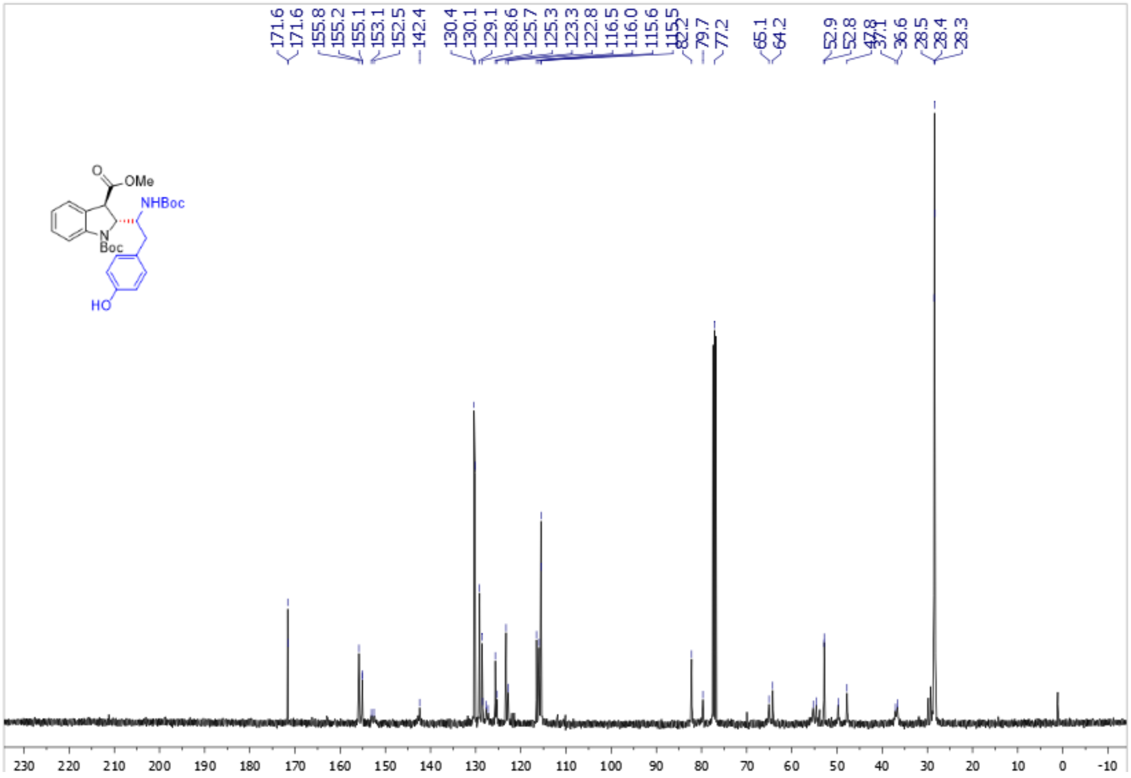

Supplementary Figure 96. ^{13}C NMR spectra of *trans*-**3y**

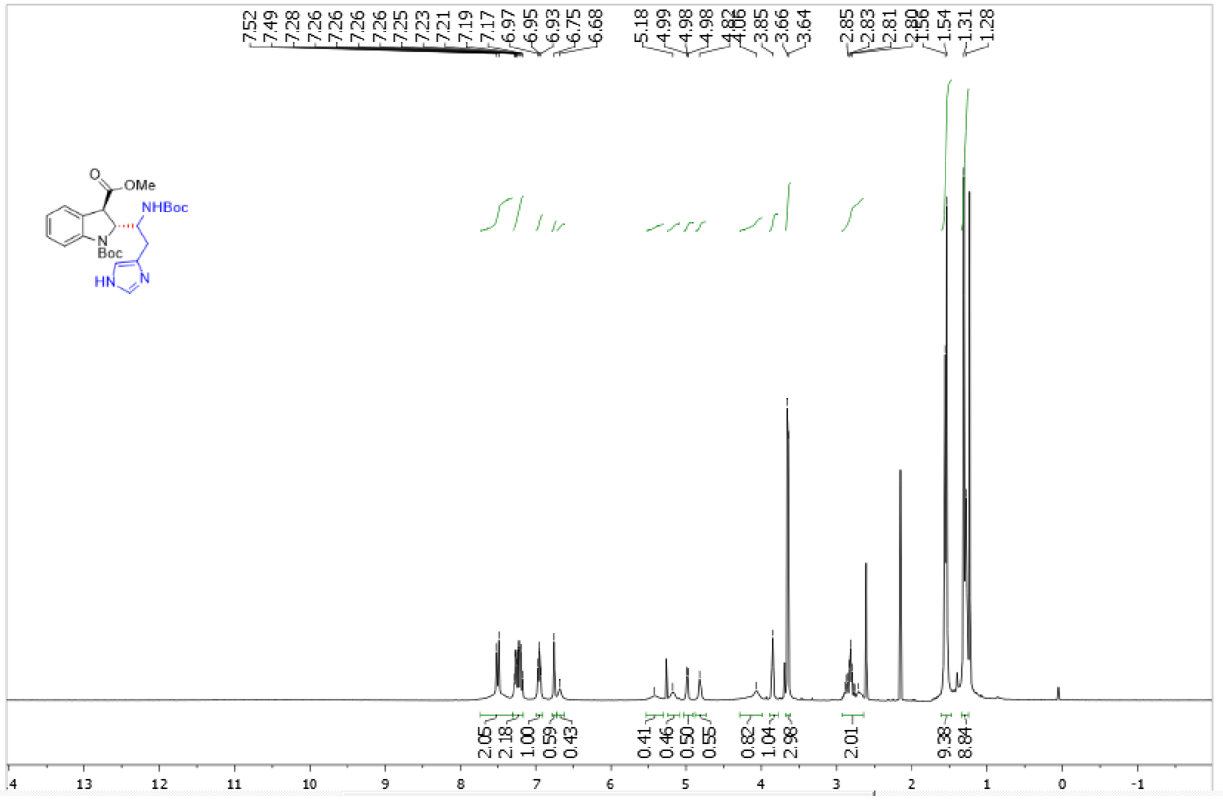

Supplementary Figure 97. ^{1}H NMR spectra of *trans*-**3z**

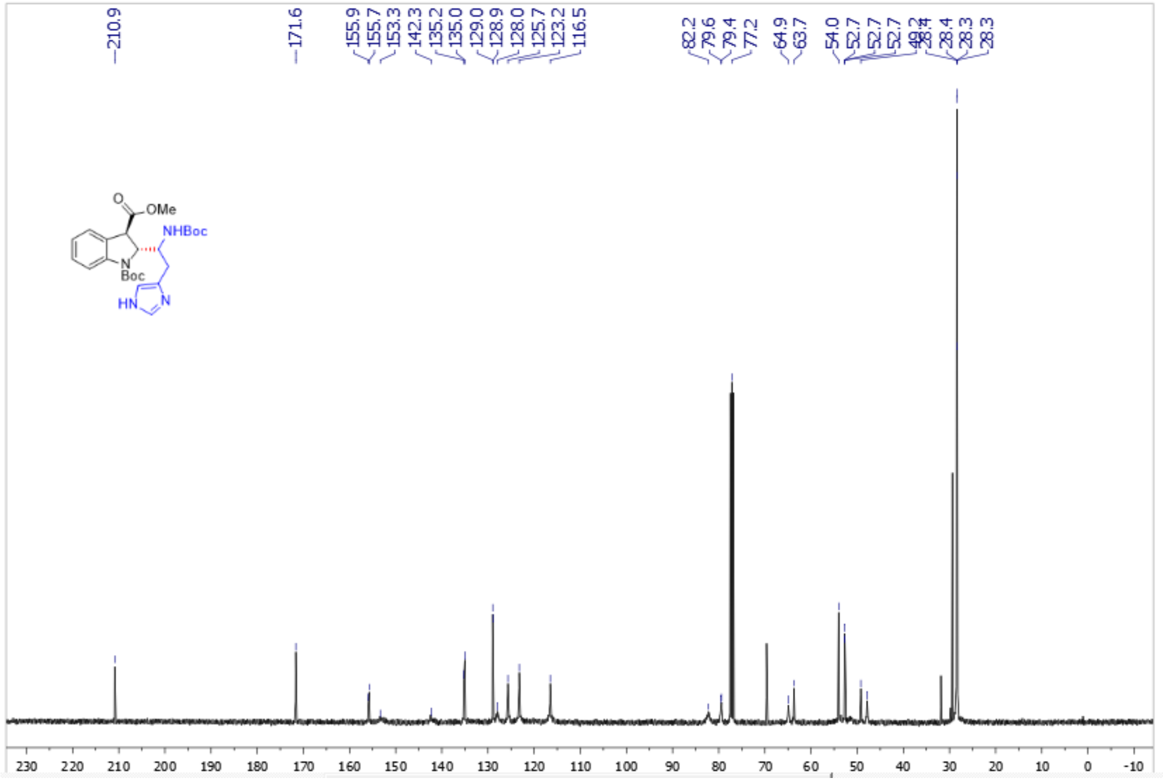

Supplementary Figure 98. ^{13}C NMR spectra of *trans*-**3z**

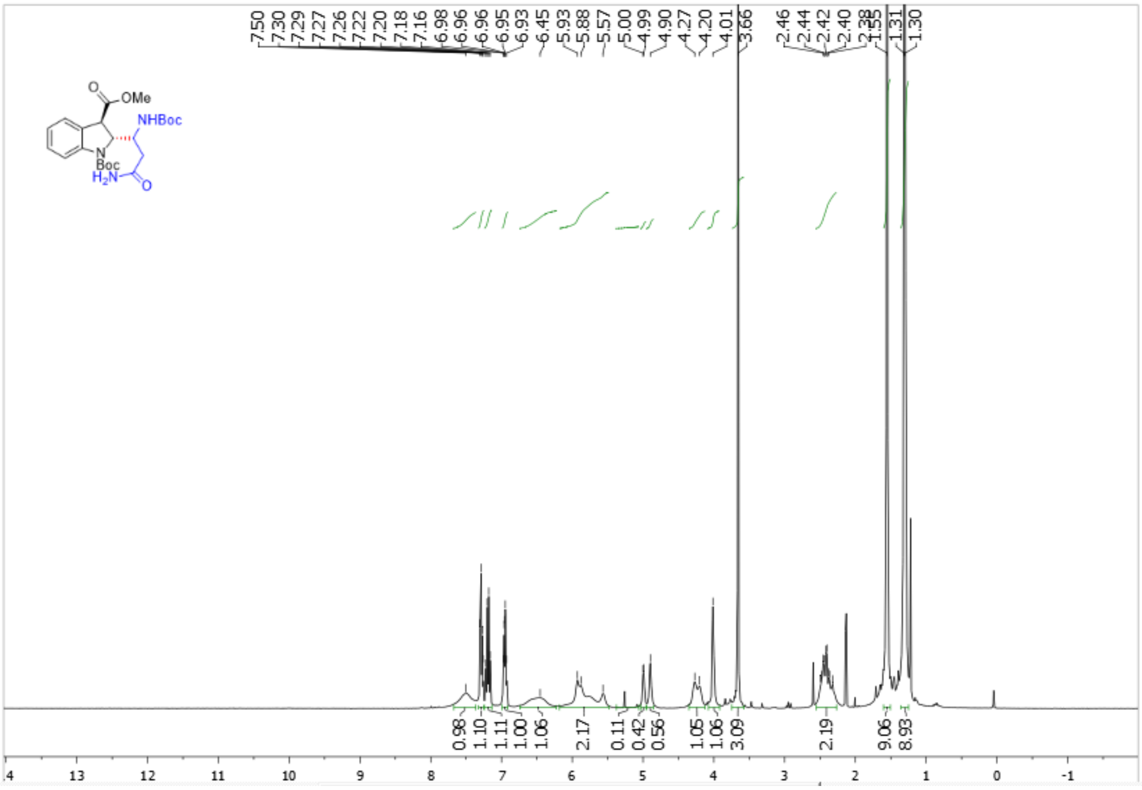

Supplementary Figure 99. ^{1}H NMR spectra of *trans*-**3aa**

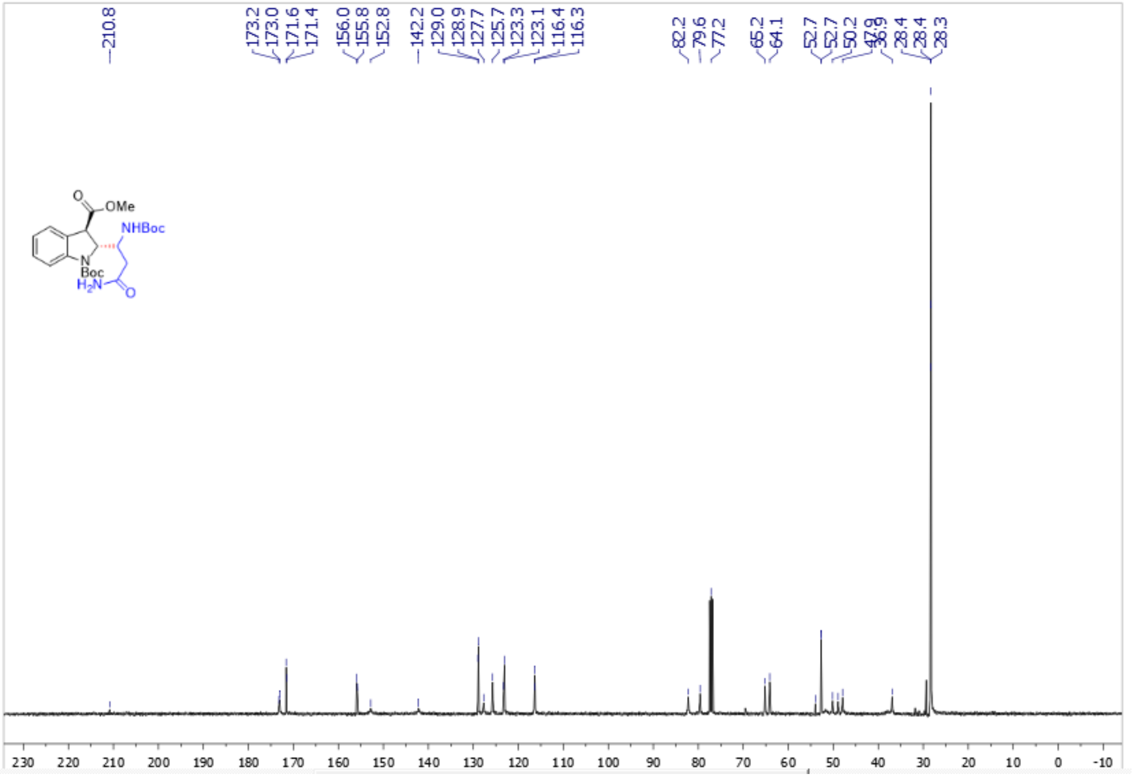

Supplementary Figure 100. ^{13}C NMR spectra of *trans*-**3aa**

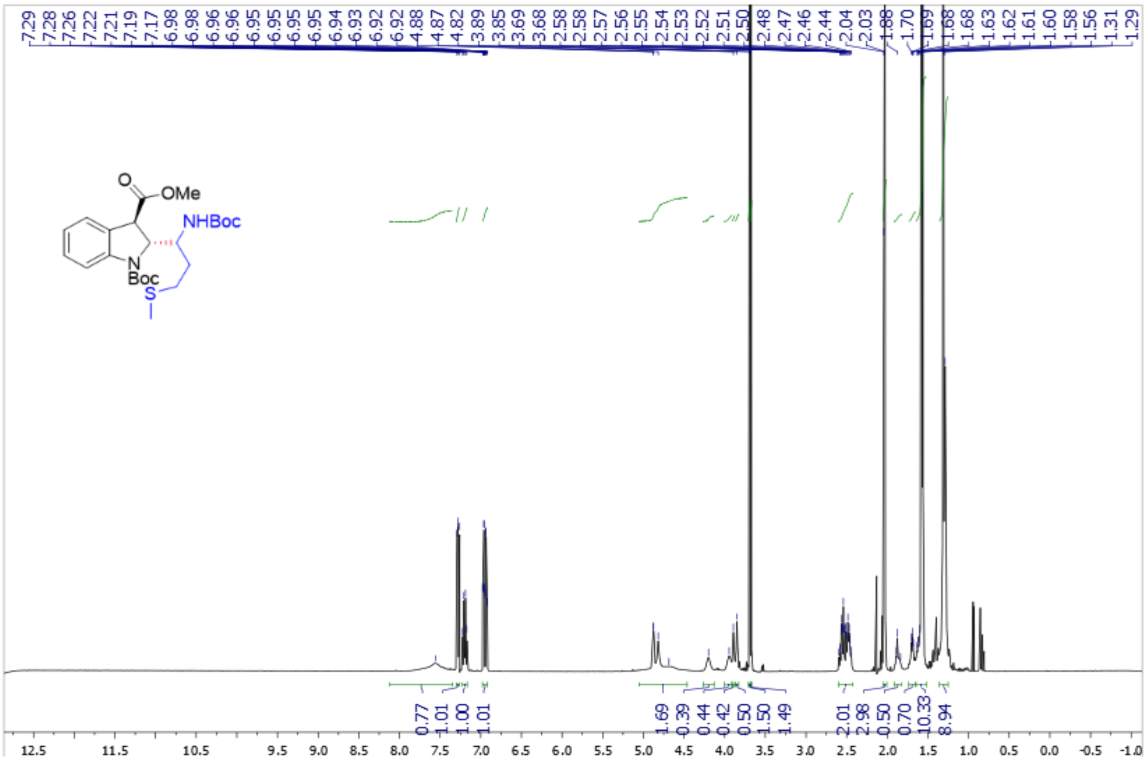

Supplementary Figure 101. ^{1}H NMR spectra of *trans*-**3ab**

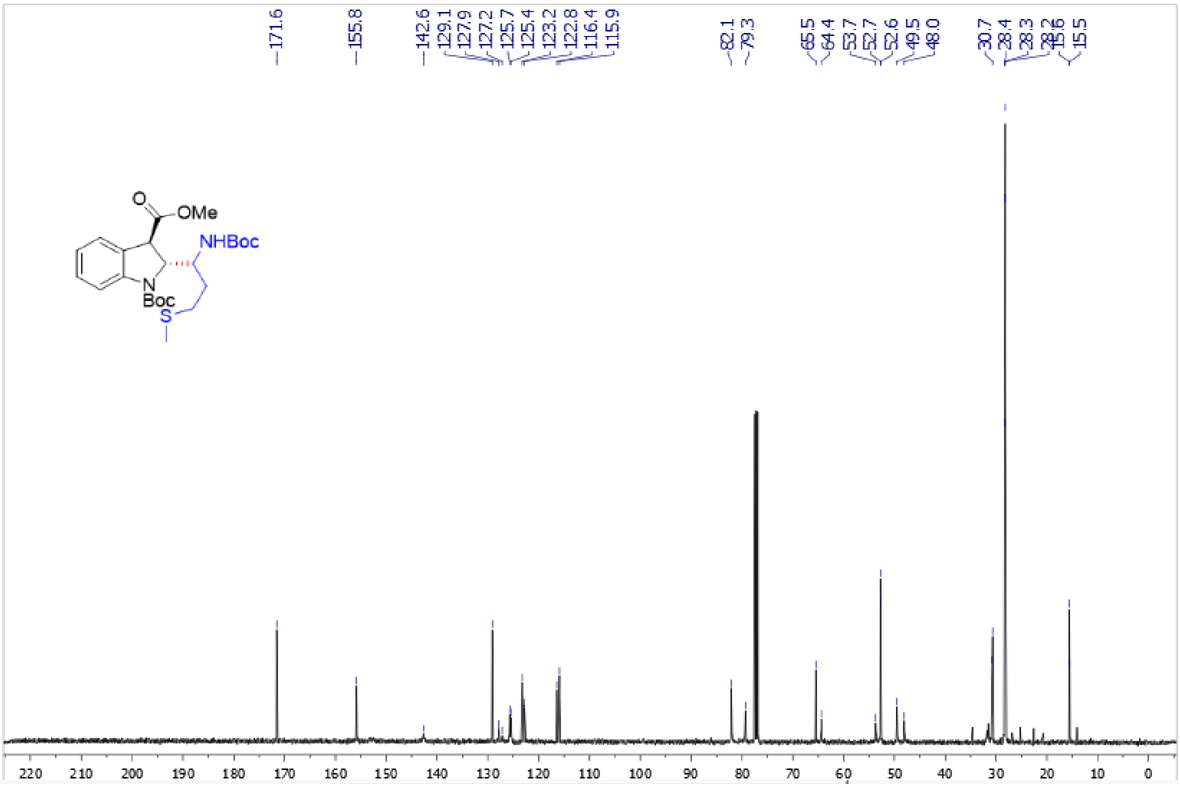

Supplementary Figure 102. ^{13}C NMR spectra of *trans*-**3ab**

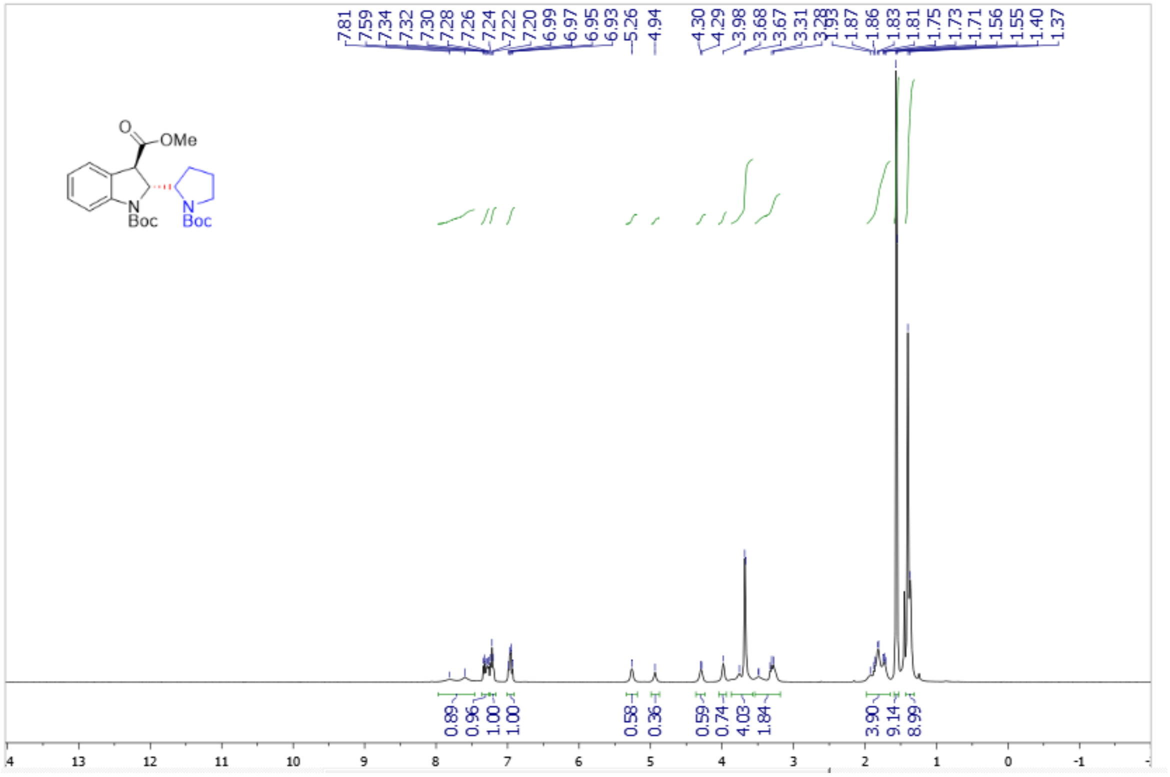

Supplementary Figure 103. ^{1}H NMR spectra of *trans*-**3ac**

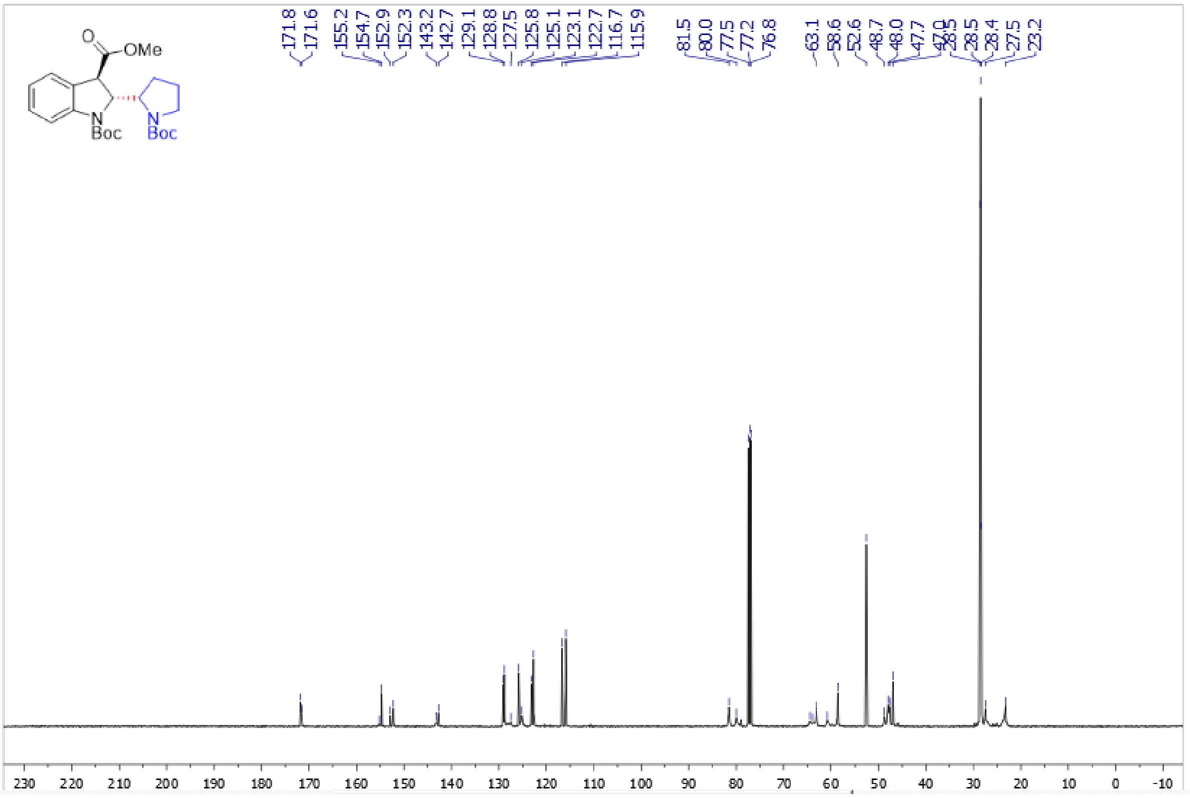

Supplementary Figure 104. ^{13}C NMR spectra of *trans*-**3ac**

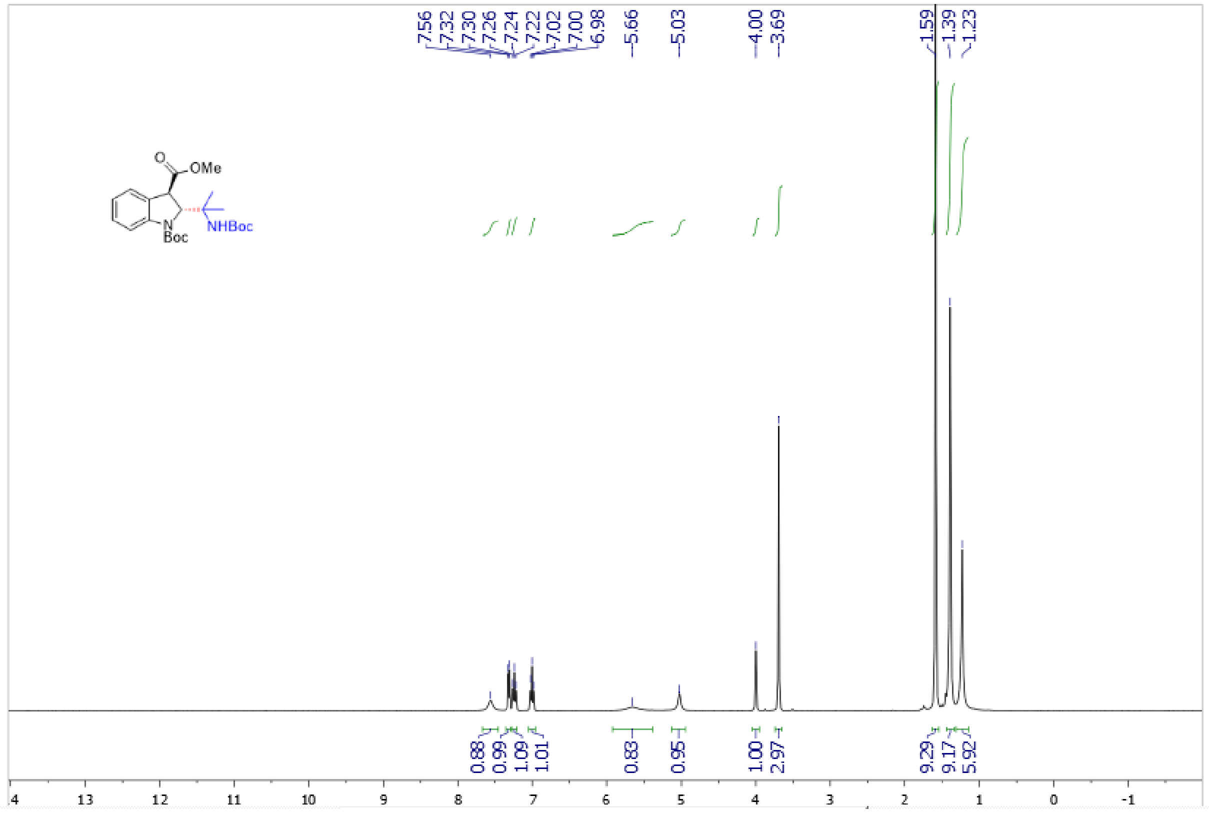

Supplementary Figure 105. ^{1}H NMR spectra of *trans*-**3ad**

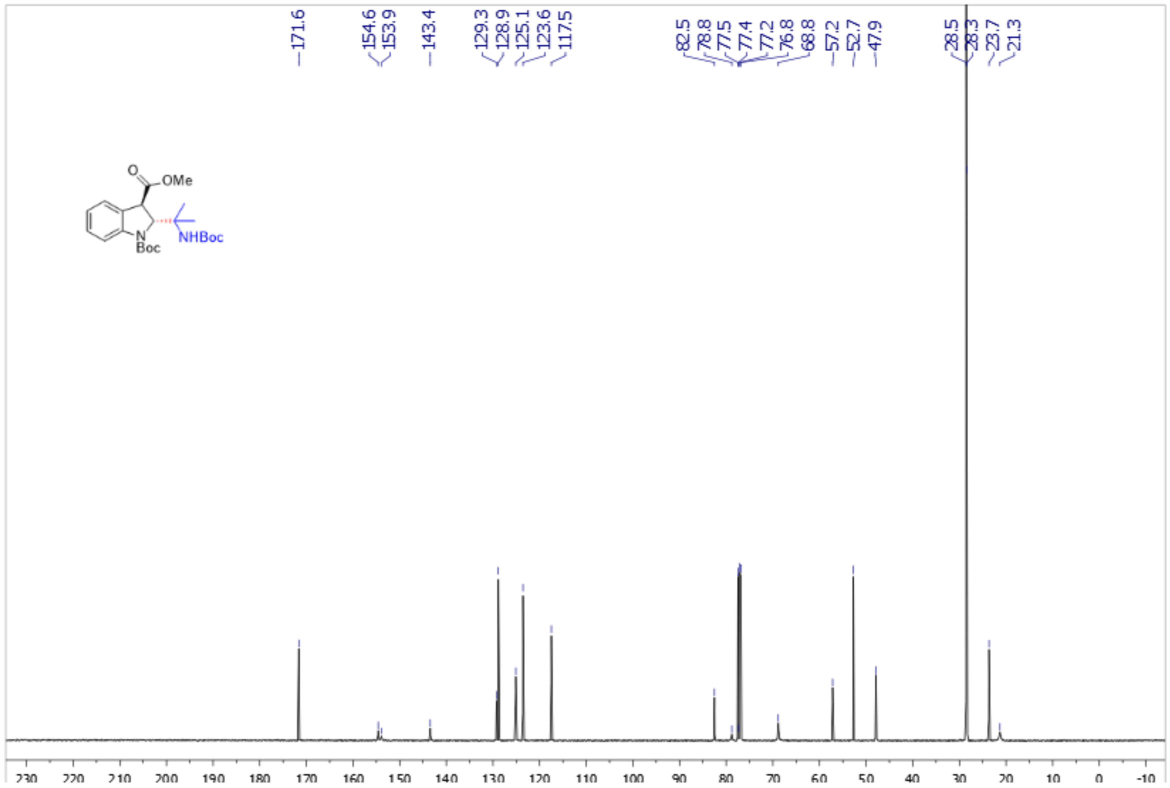

Supplementary Figure 106. ^{13}C NMR spectra of *trans*-**3ad**

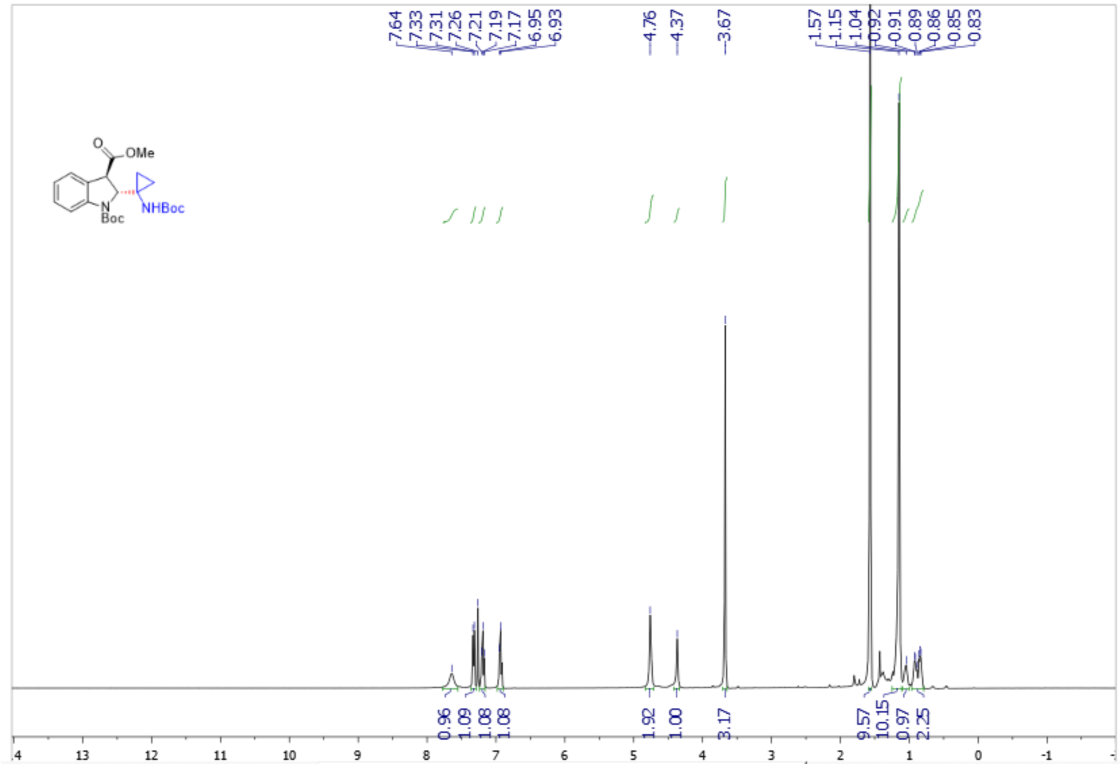

Supplementary Figure 107. ^{1}H NMR spectra of *trans*-**3ae**

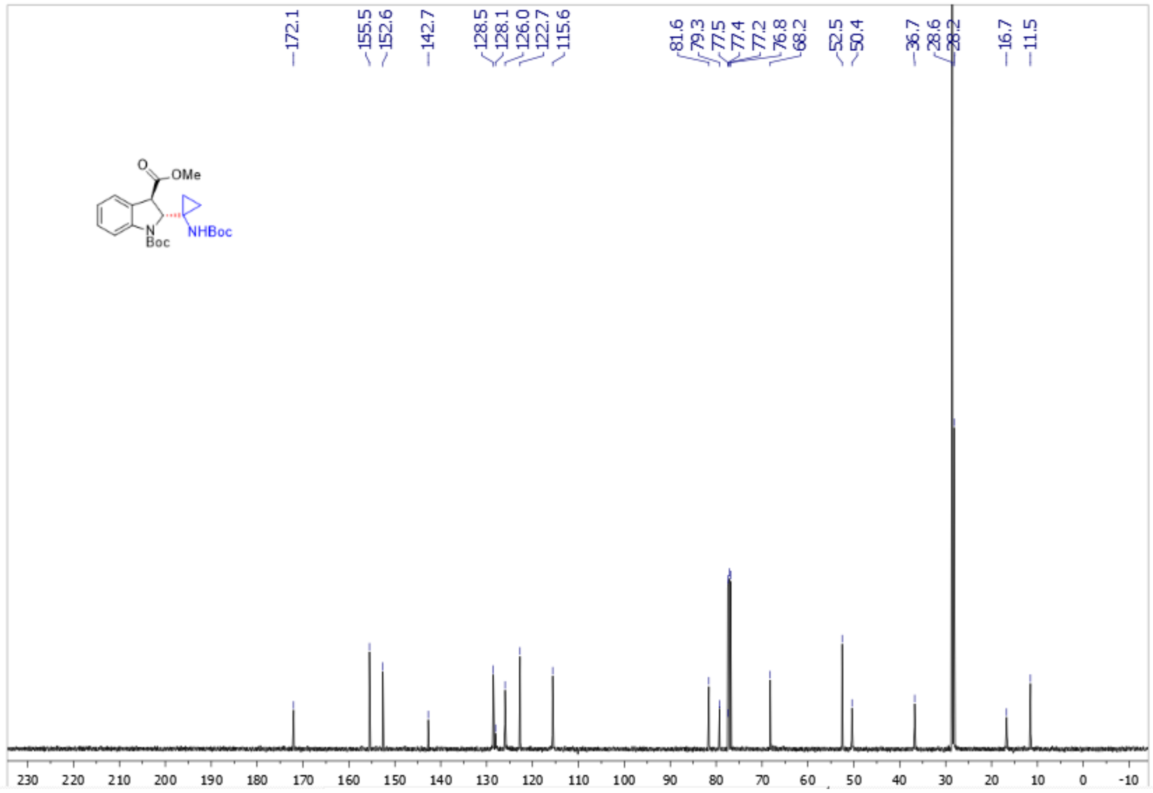

Supplementary Figure 108. ^{13}C NMR spectra of *trans*-**3ae**

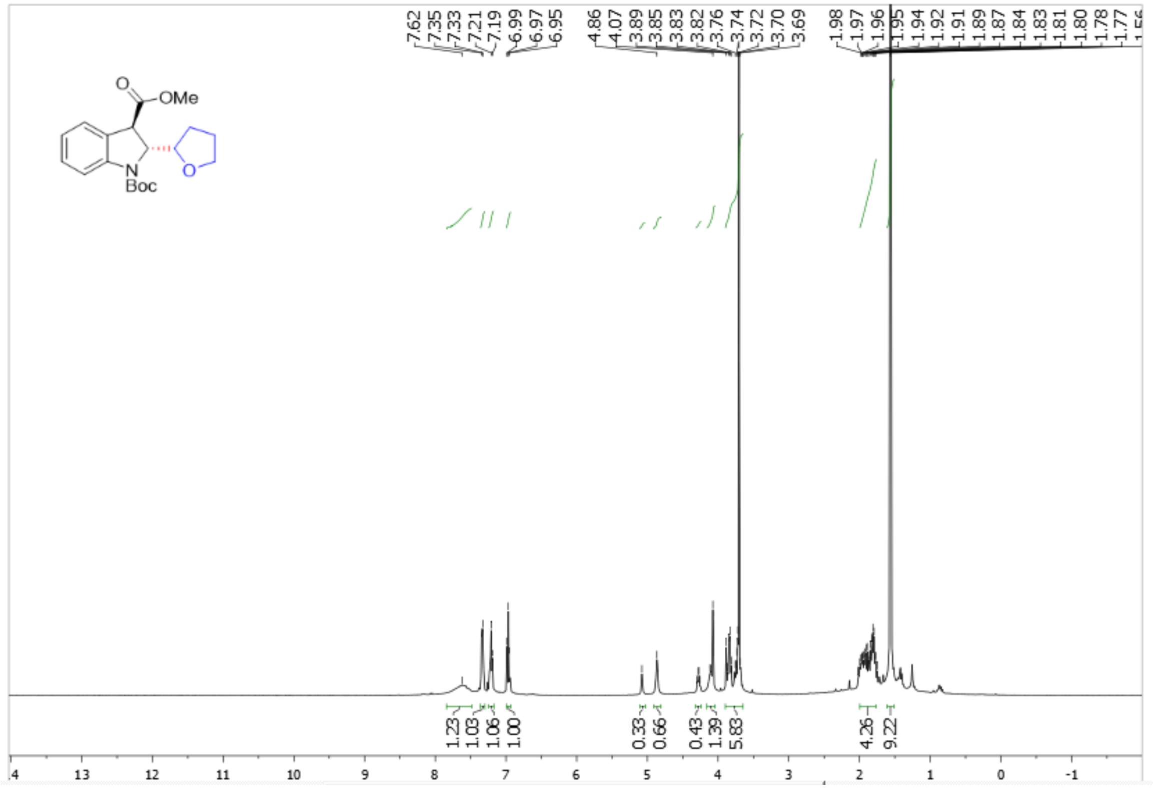

Supplementary Figure 109. ^{1}H NMR spectra of *trans*-**3af**

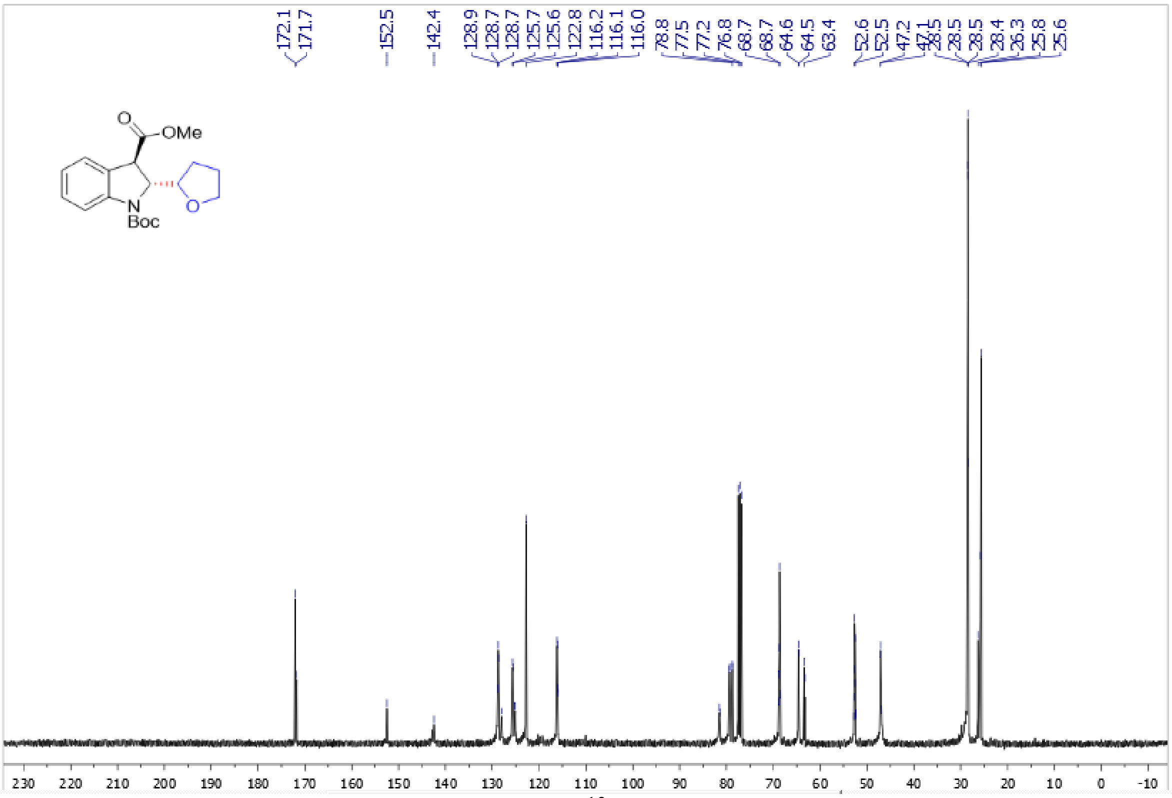

Supplementary Figure 110. ^{13}C NMR spectra of *trans*-**3af**

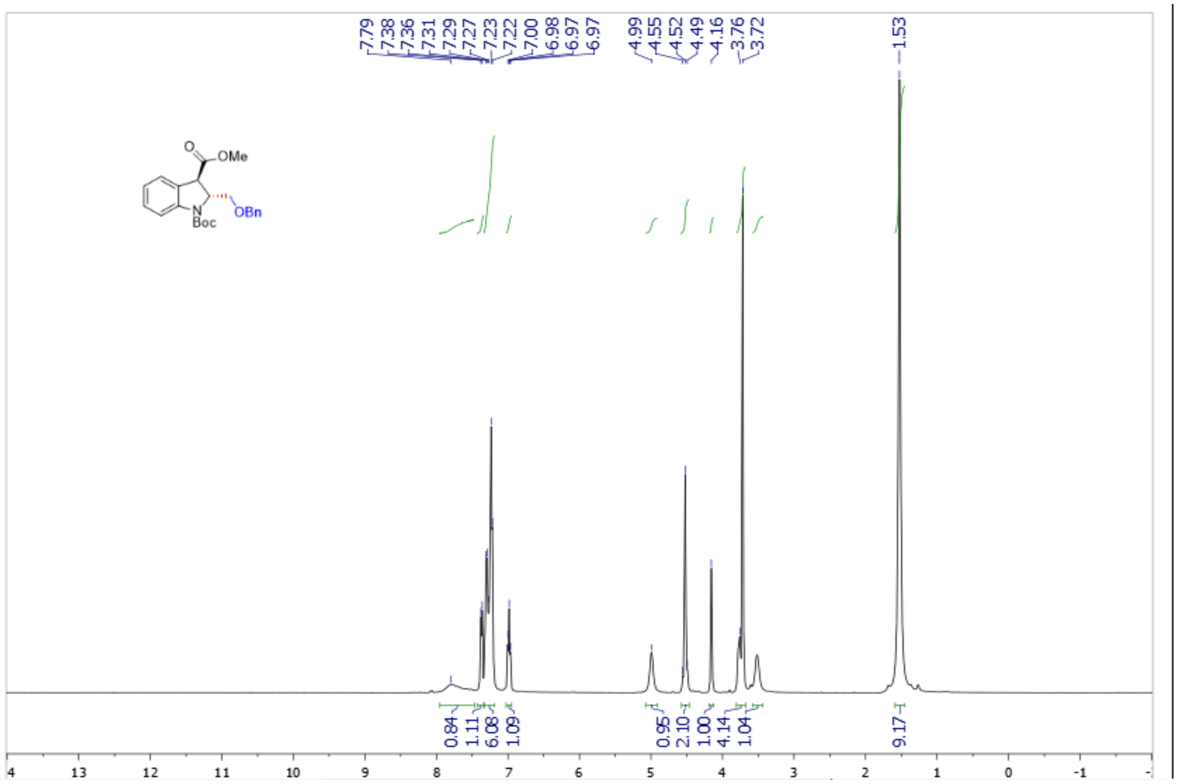

Supplementary Figure 111. ^{1}H NMR spectra of *trans*-**3ag**

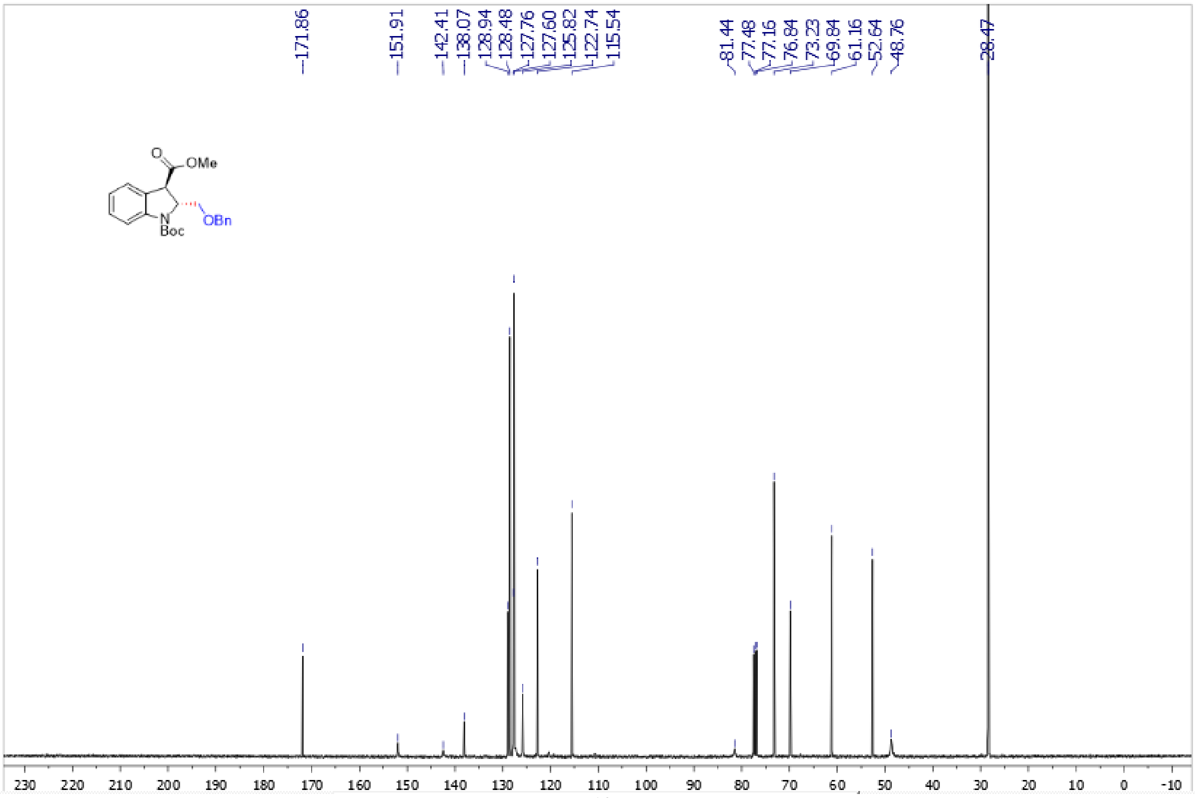

Supplementary Figure 112. ^{13}C NMR spectra of *trans*-**3ag**

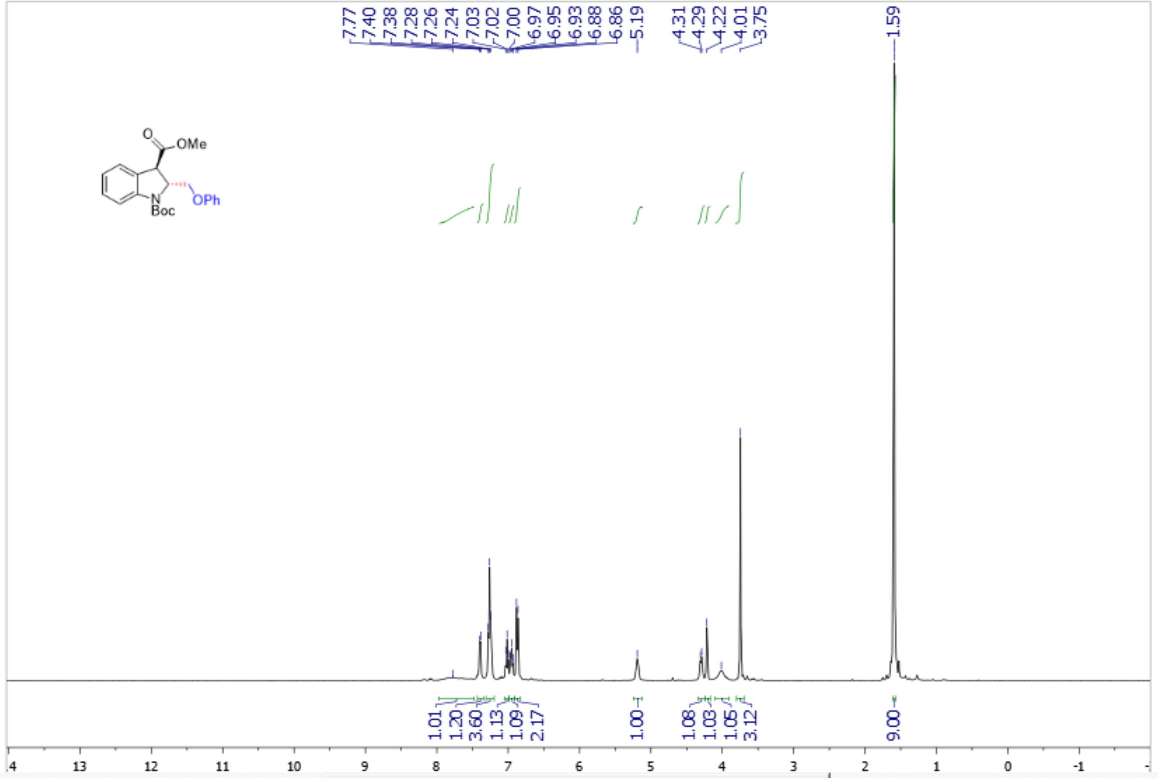

Supplementary Figure 113. ^{1}H NMR spectra of *trans*-**3ah**

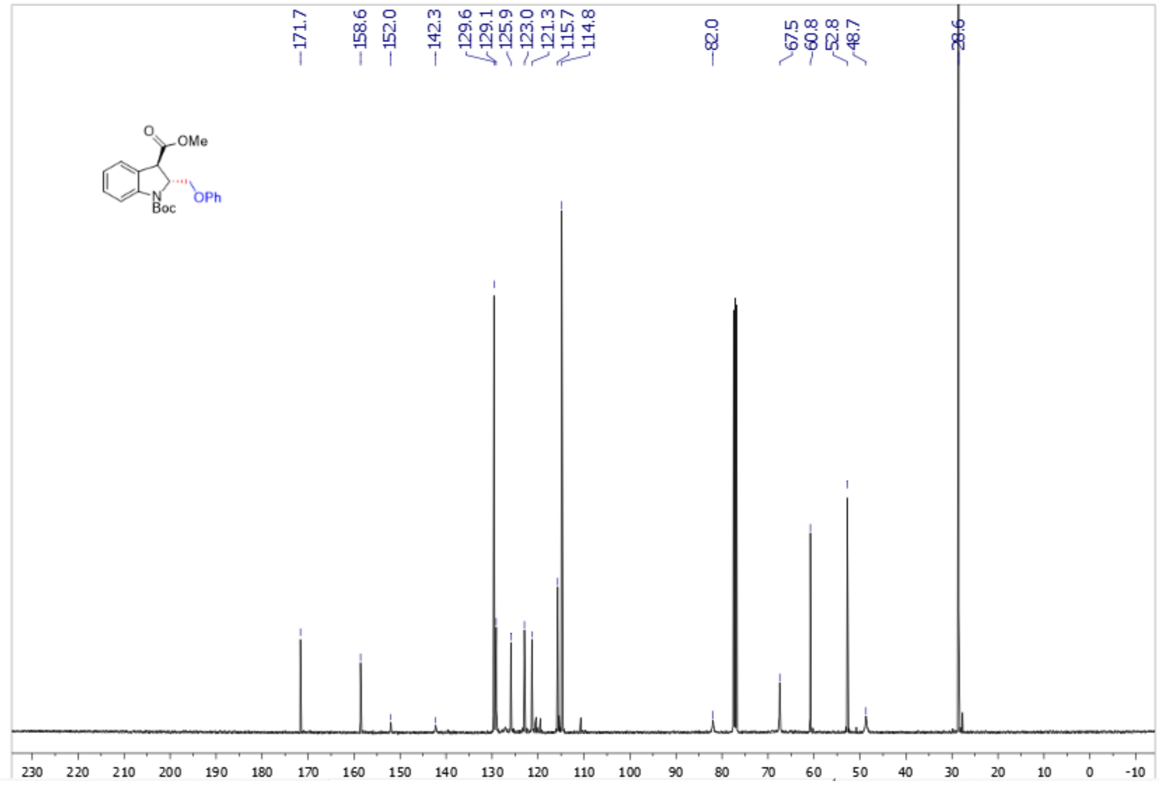

Supplementary Figure 114. ^{13}C NMR spectra of *trans*-**3ah**

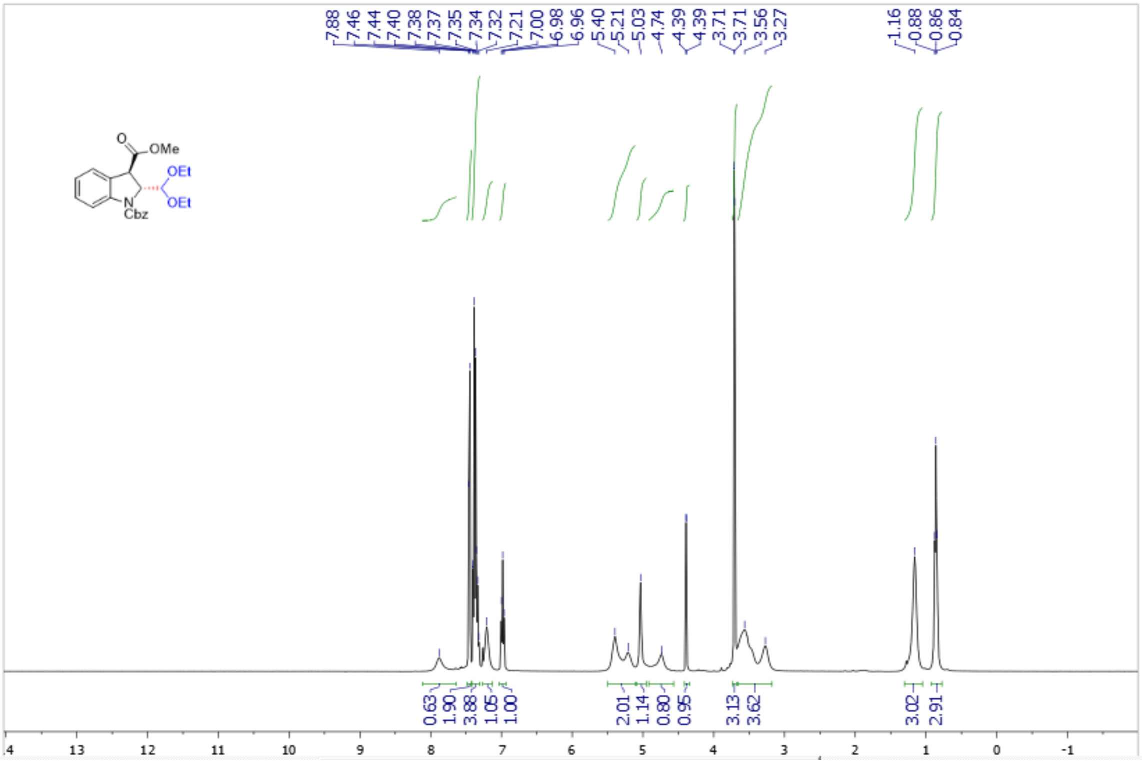

Supplementary Figure 115. ^{1}H NMR spectra of *trans*-**3ai**

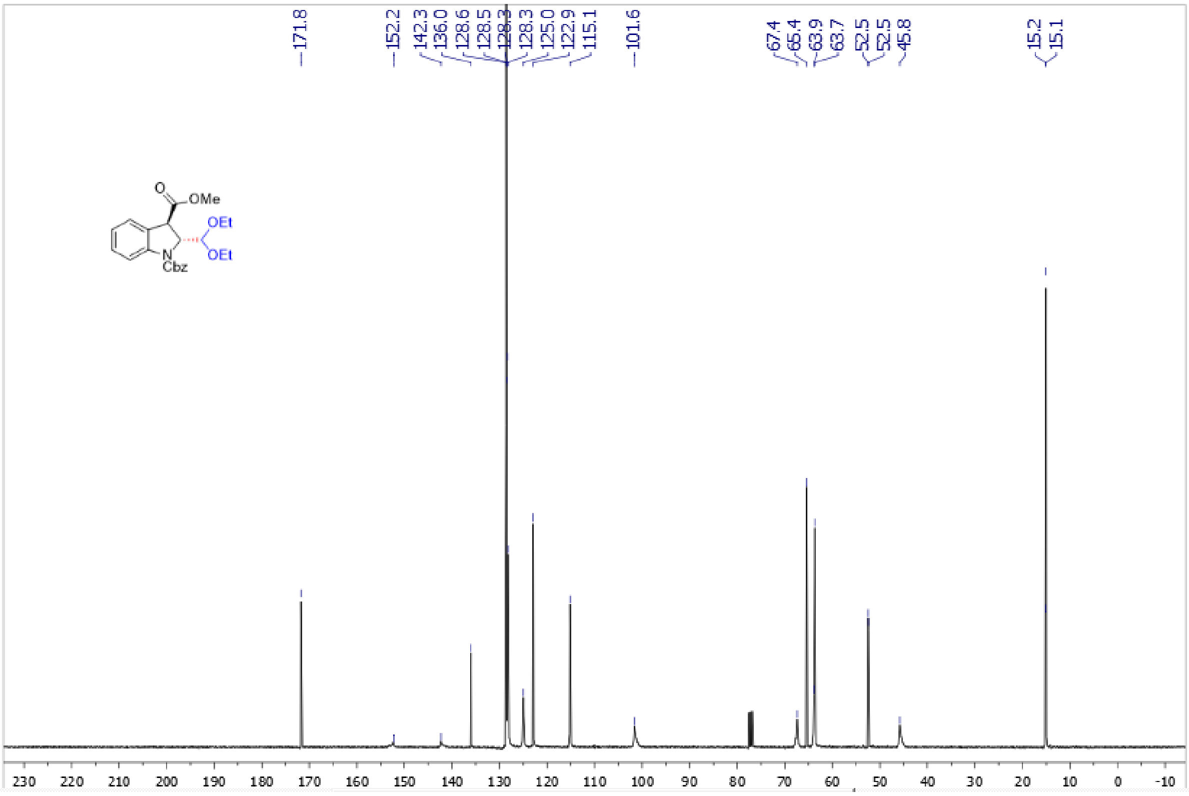

Supplementary Figure 116. ^{13}C NMR spectra of *trans*-**3ai**

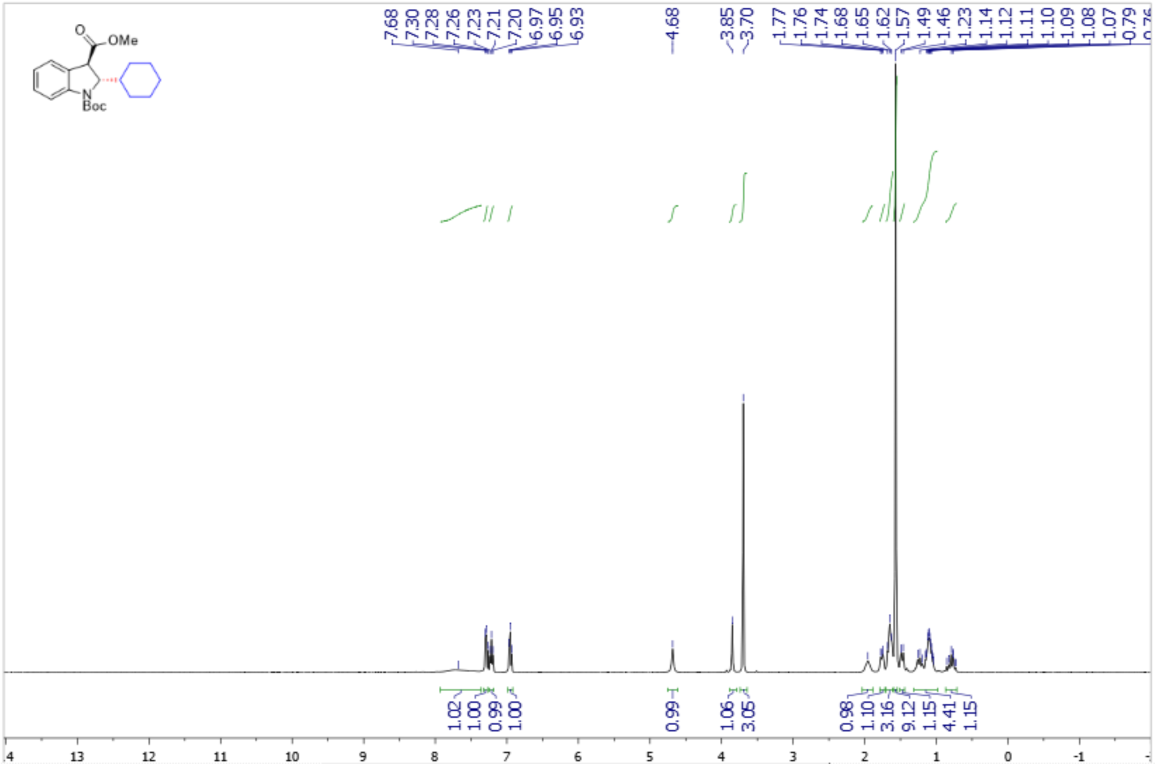

Supplementary Figure 117. ^{1}H NMR spectra of *trans*-**3aj**

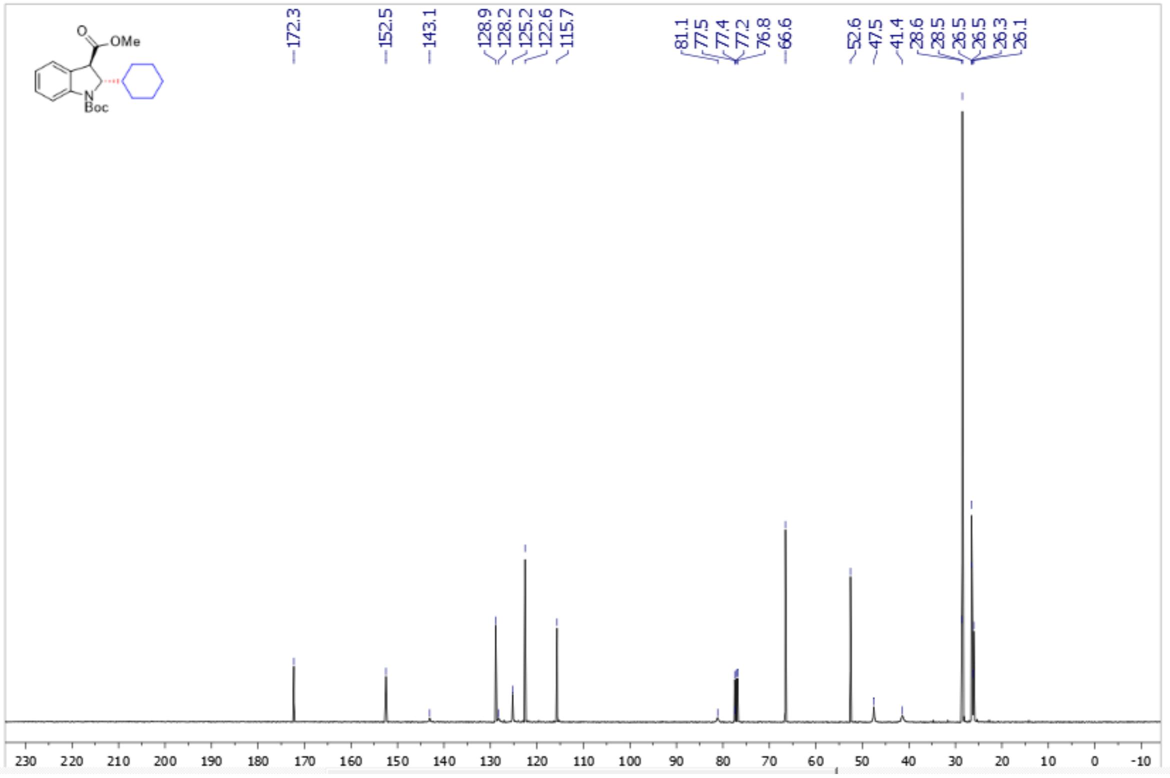

Supplementary Figure 118. ^{13}C NMR spectra of *trans*-**3aj**

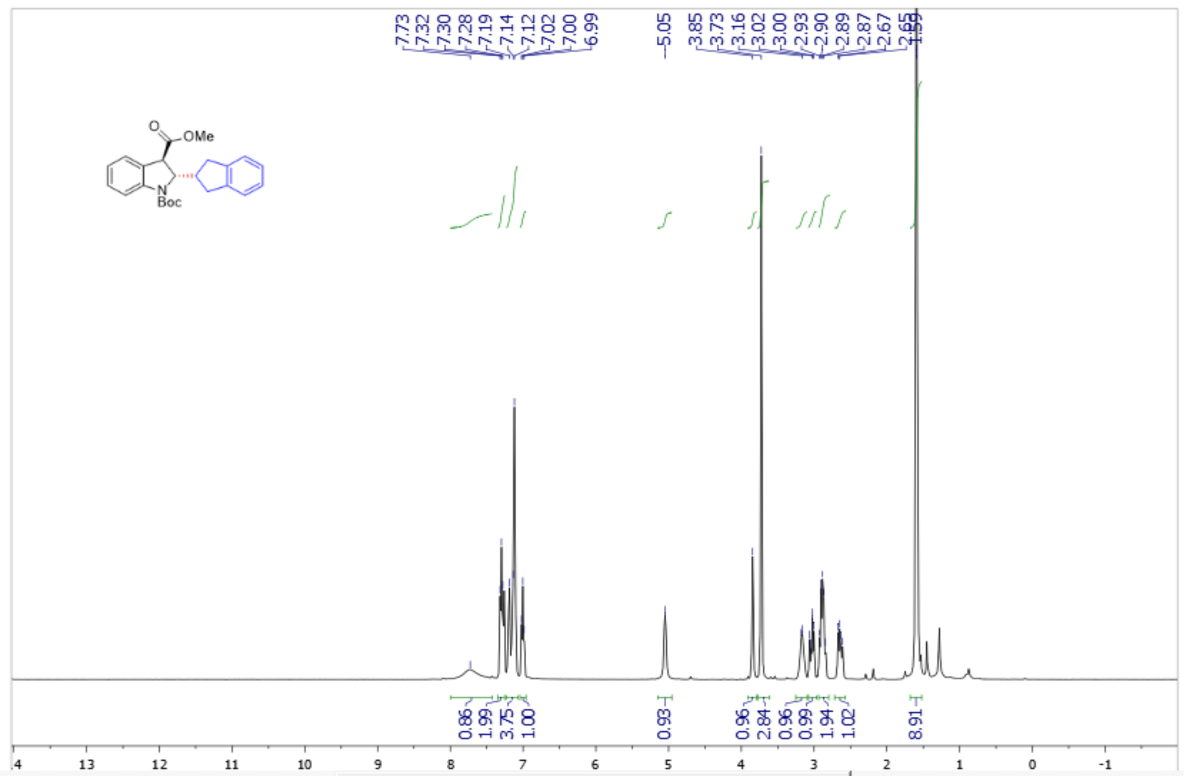

Supplementary Figure 119. ^{1}H NMR spectra of *trans*-**3ak**

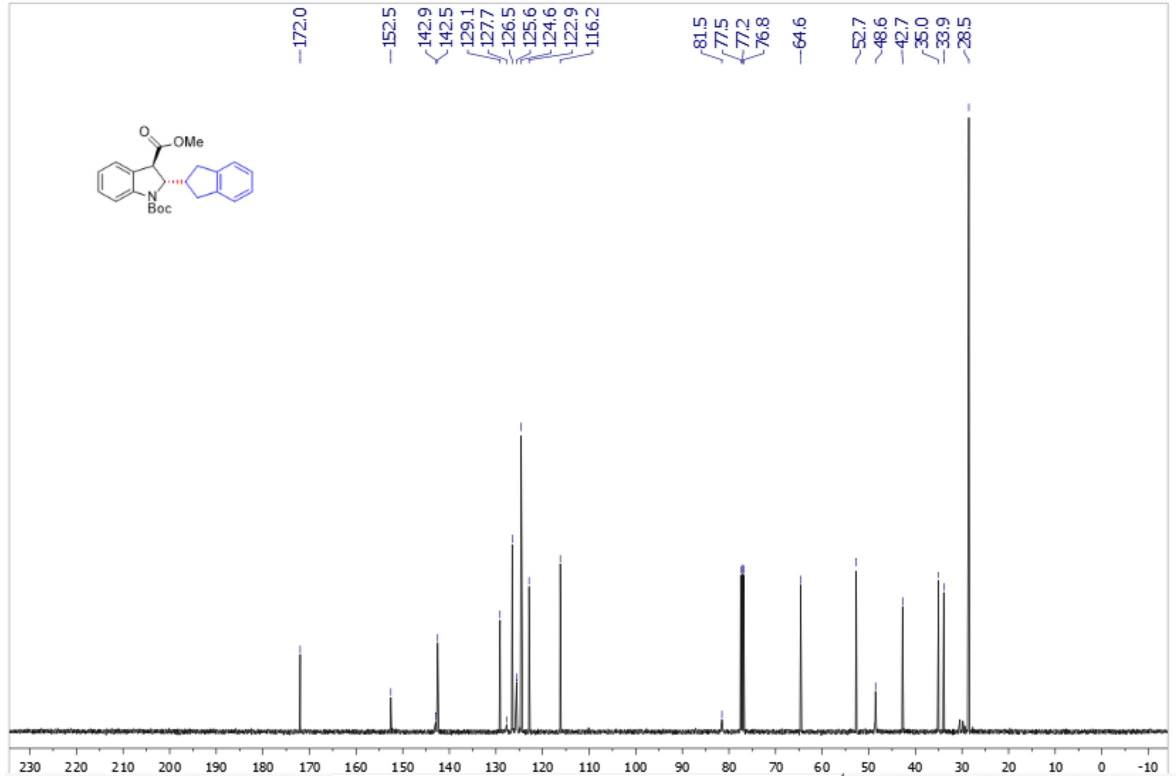

Supplementary Figure 120. ^{13}C NMR spectra of *trans*-**3ak**

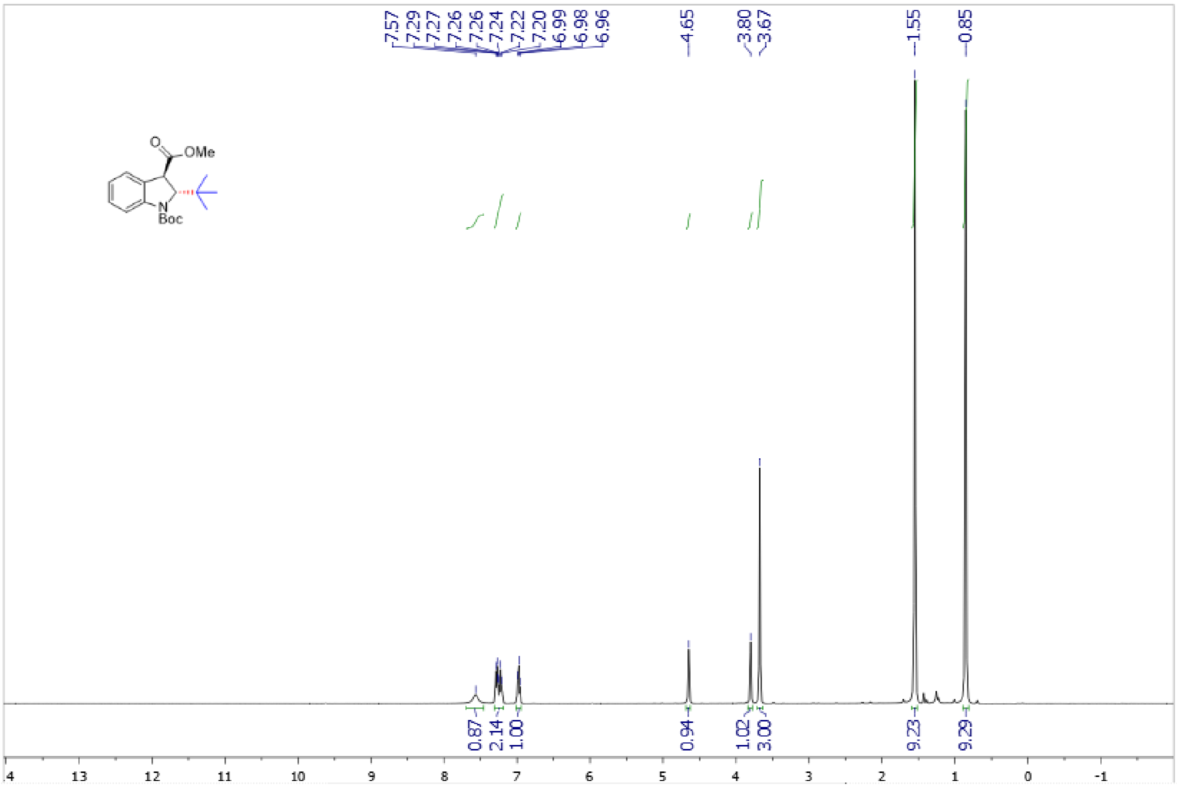

Supplementary Figure 121. ^{1}H NMR spectra of *trans*-**3al**

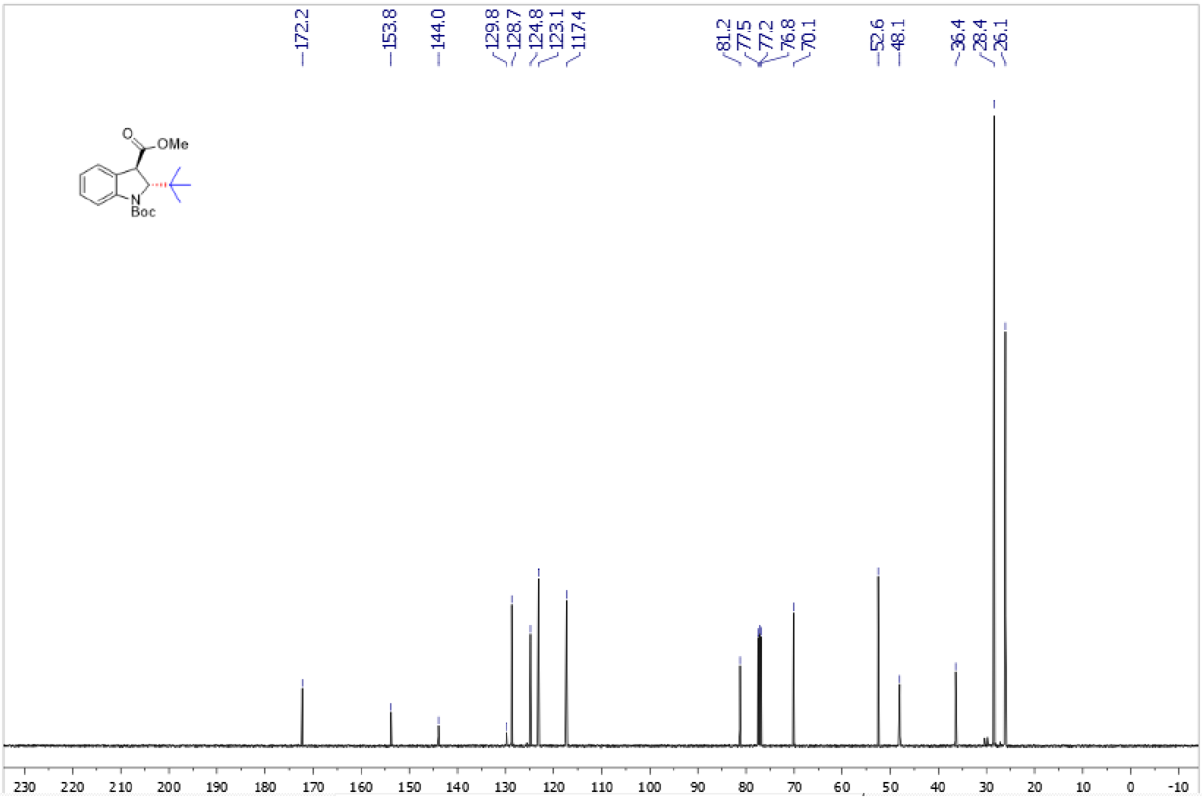

Supplementary Figure 122. ^{13}C NMR spectra of *trans*-**3al**

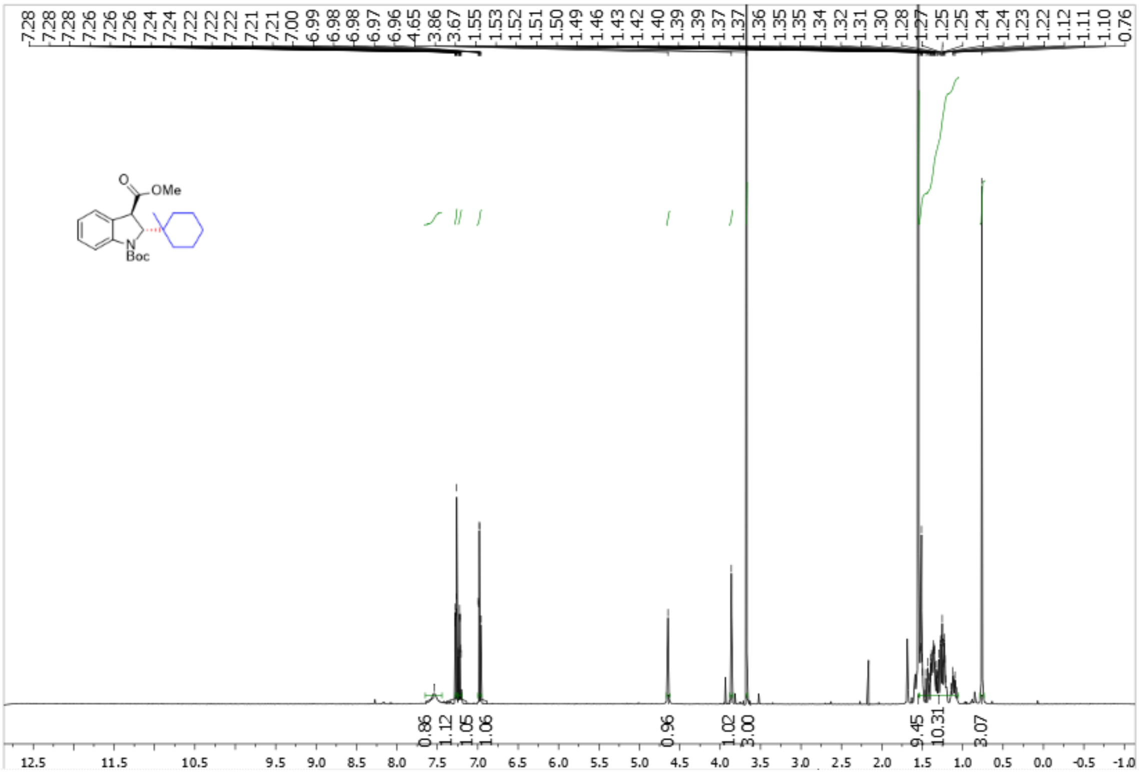

Supplementary Figure 123. ^{1}H NMR spectra of *trans*-**3am**

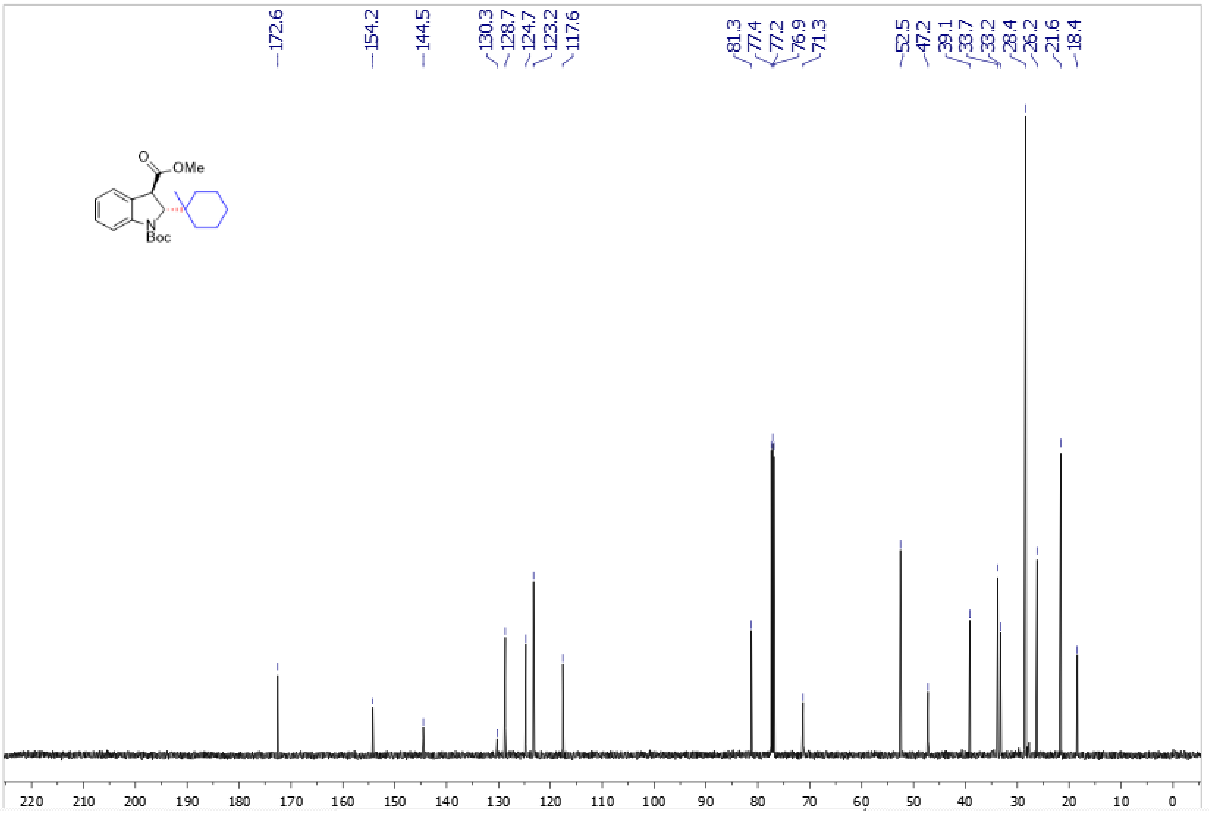

Supplementary Figure 124. ^{13}C NMR spectra of *trans*-**3am**

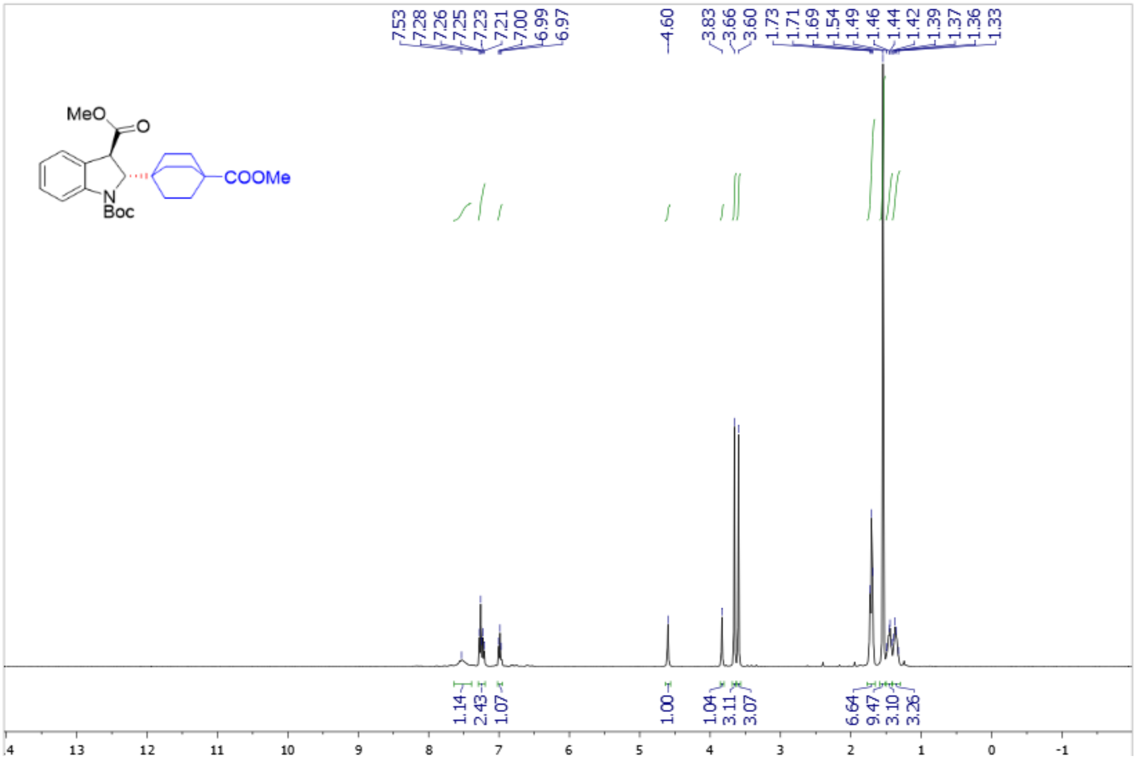

Supplementary Figure 125. ^{1}H NMR spectra of *trans*-**3an**

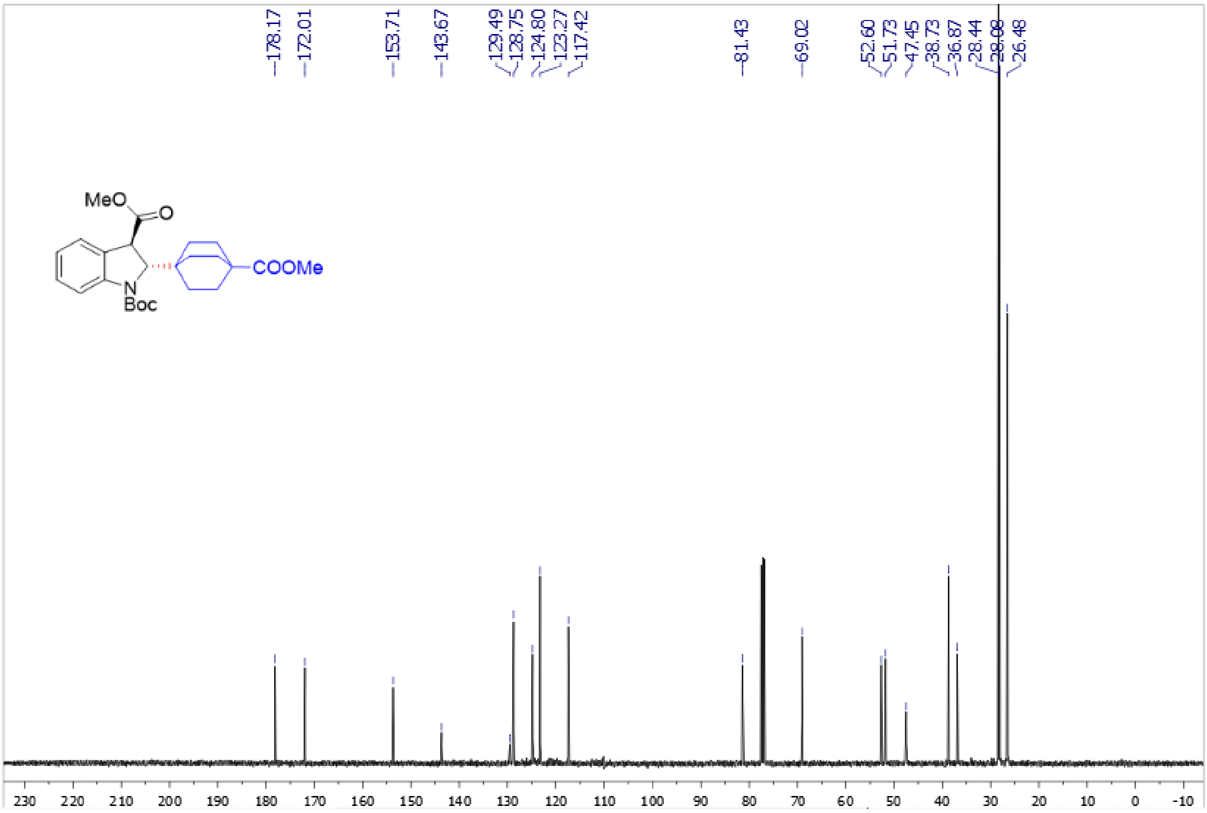

Supplementary Figure 126. ^{13}C NMR spectra of *trans*-**3an**

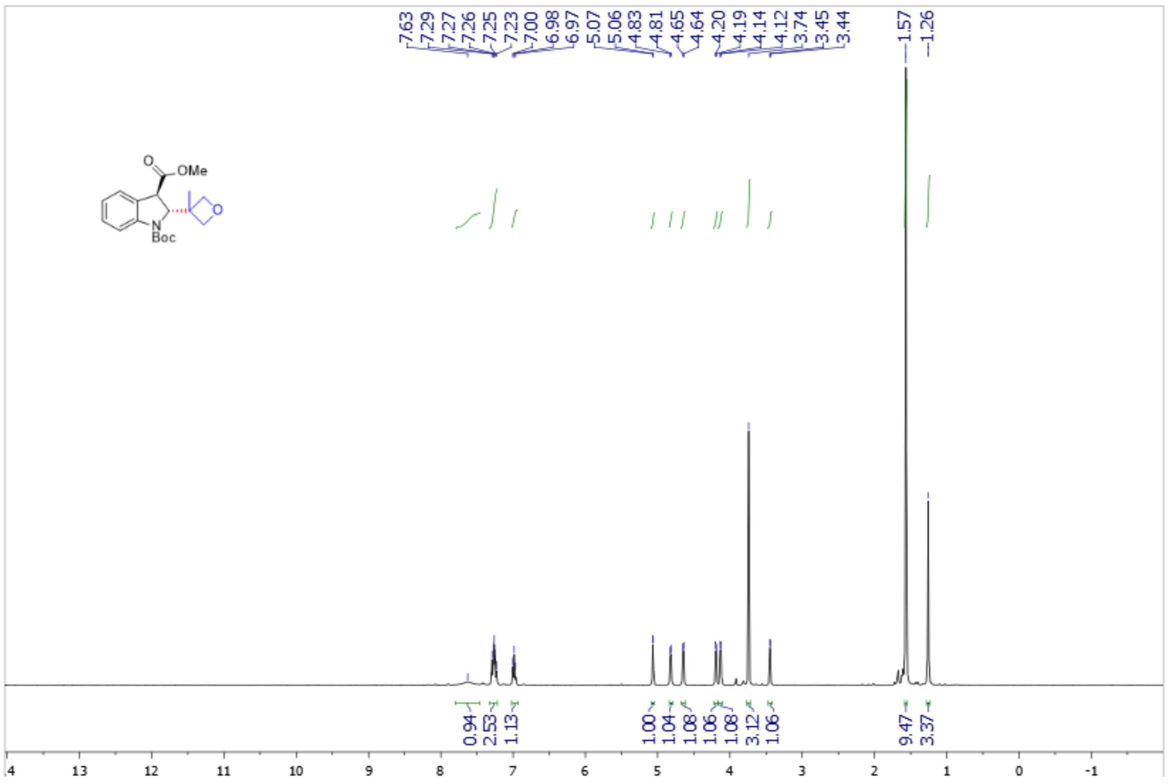

Supplementary Figure 127. ^{1}H NMR spectra of *trans*-**3ao**

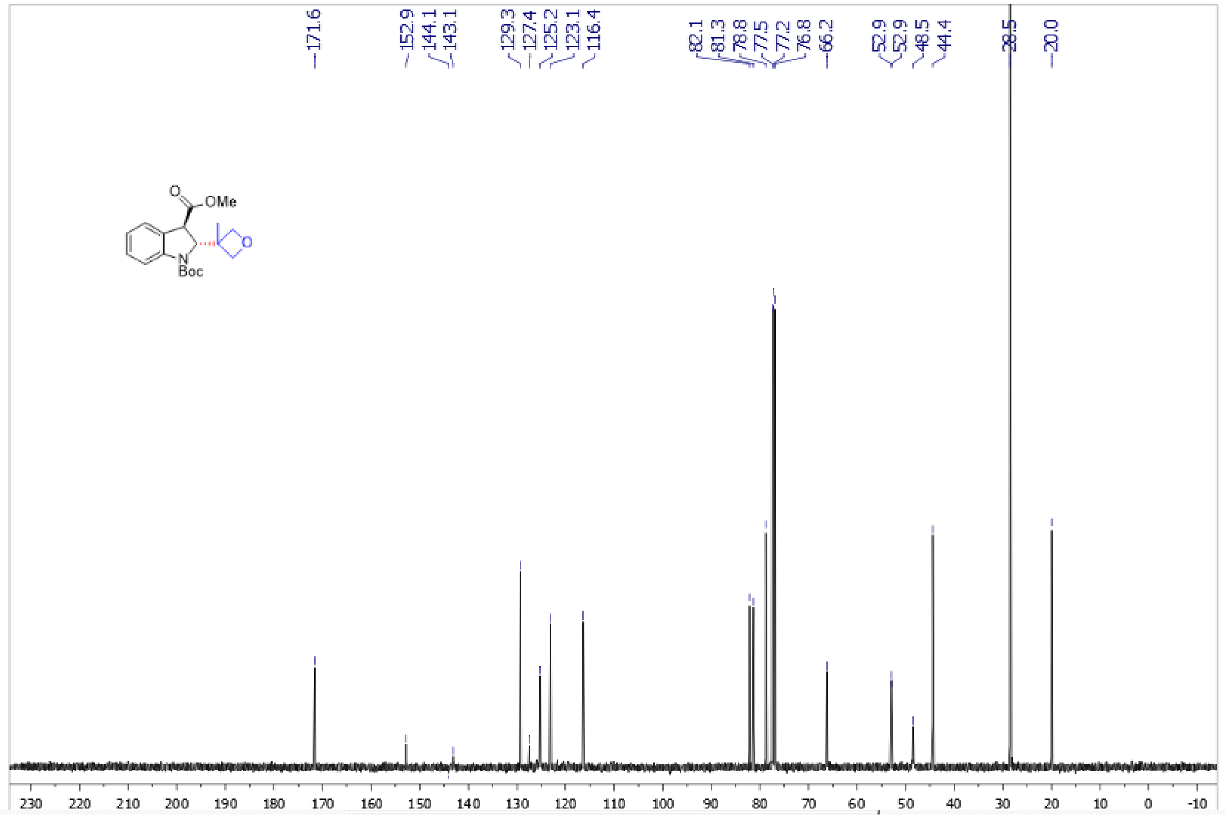

Supplementary Figure 128. ^{13}C NMR spectra of *trans*-**3ao**

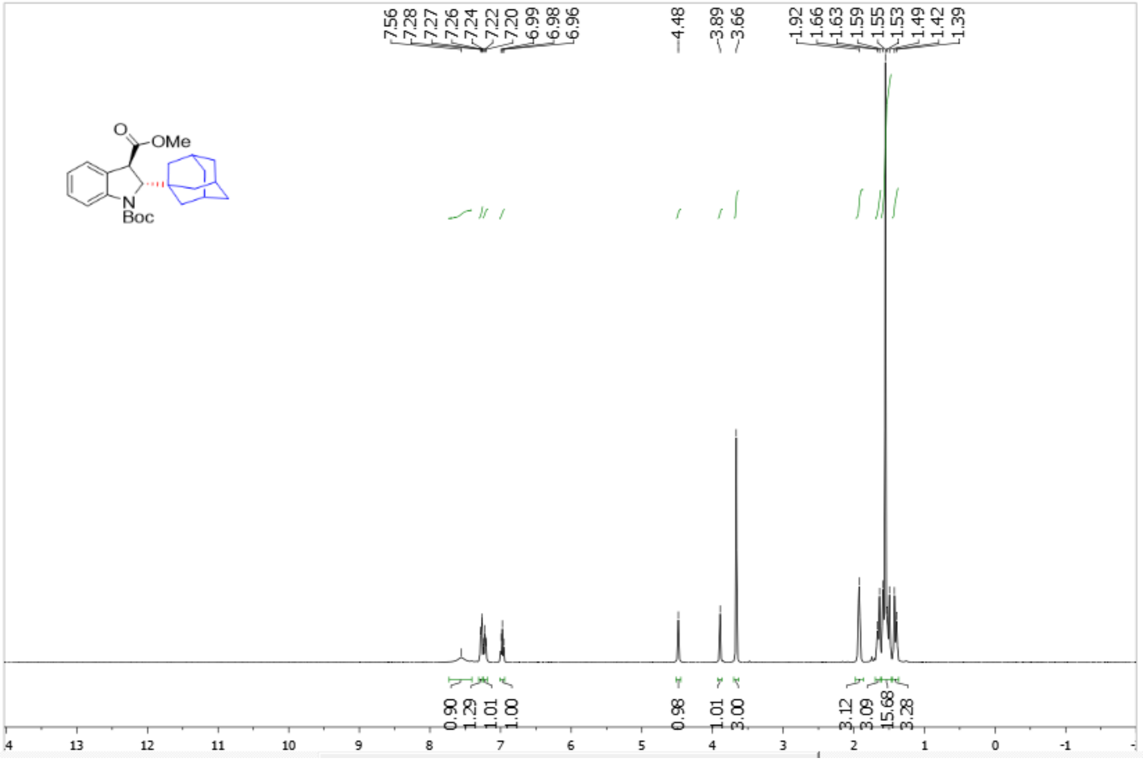

Supplementary Figure 129. ^{1}H NMR spectra of *trans*-**3ap**

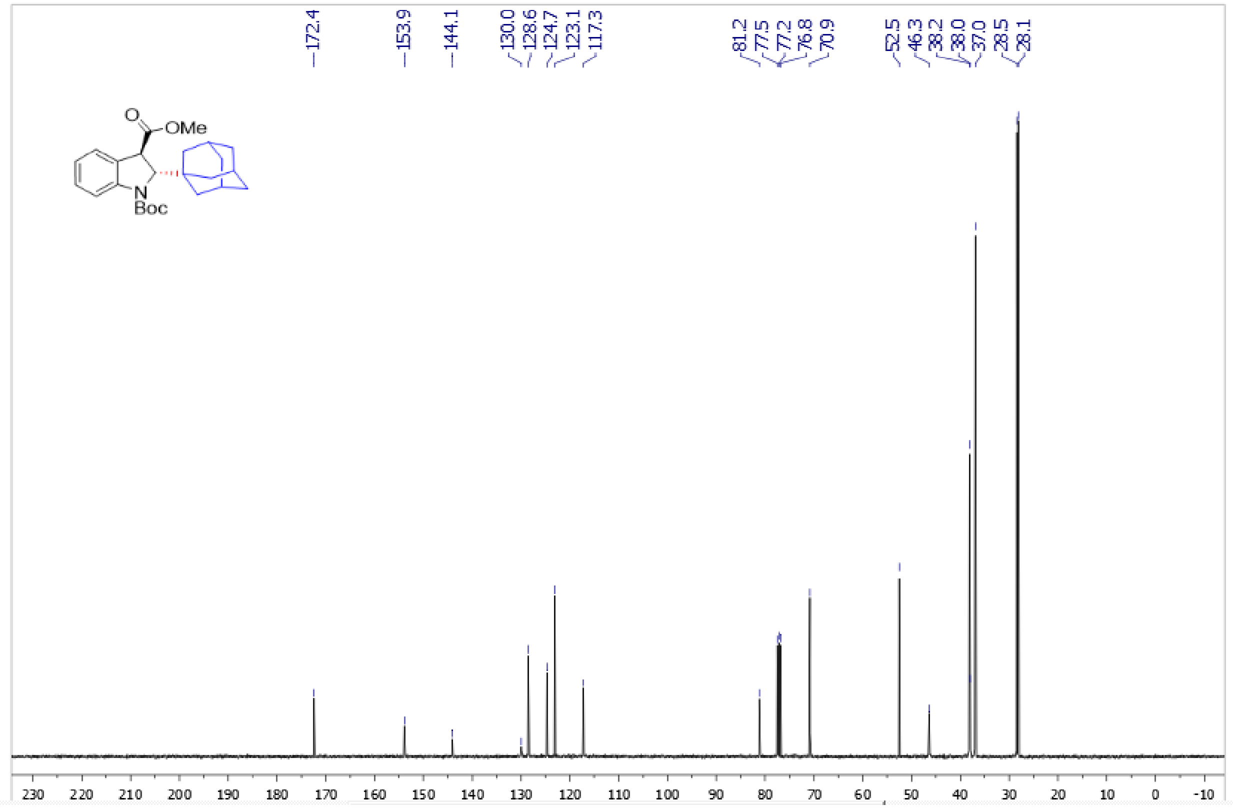

Supplementary Figure 130. ^{13}C NMR spectra of *trans*-**3ap**

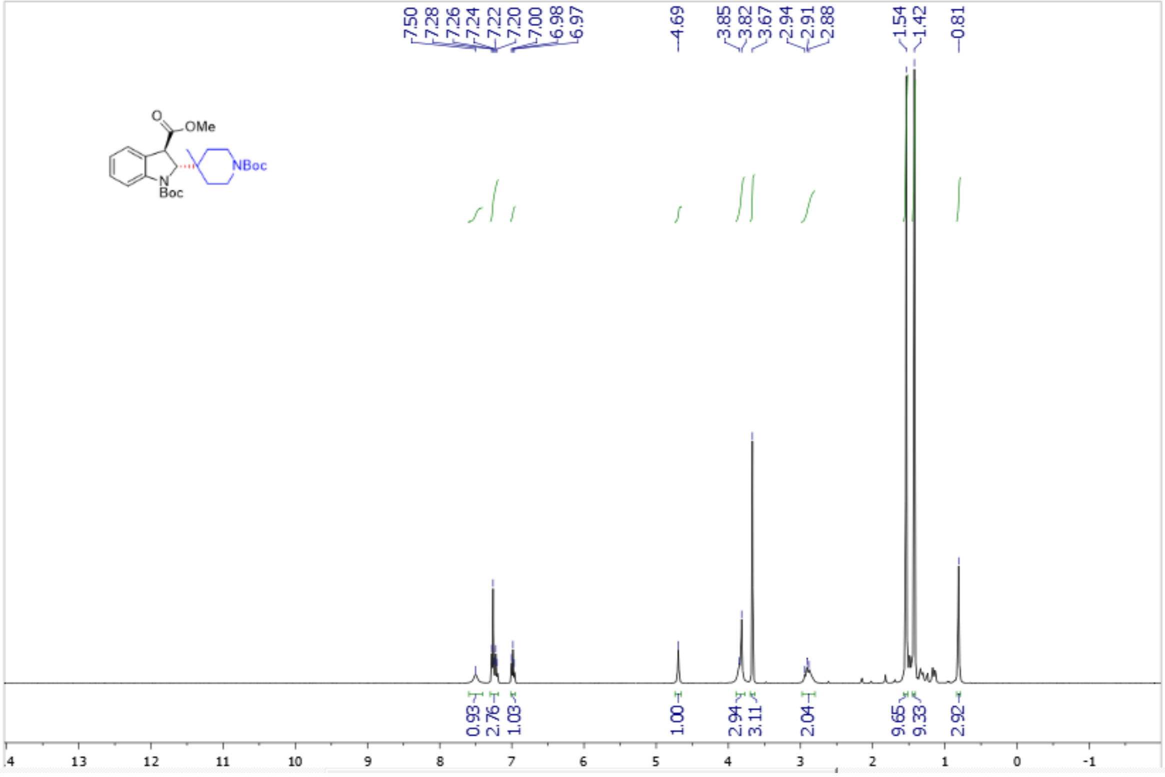

Supplementary Figure 131. ^{1}H NMR spectra of *trans*-**3aq**

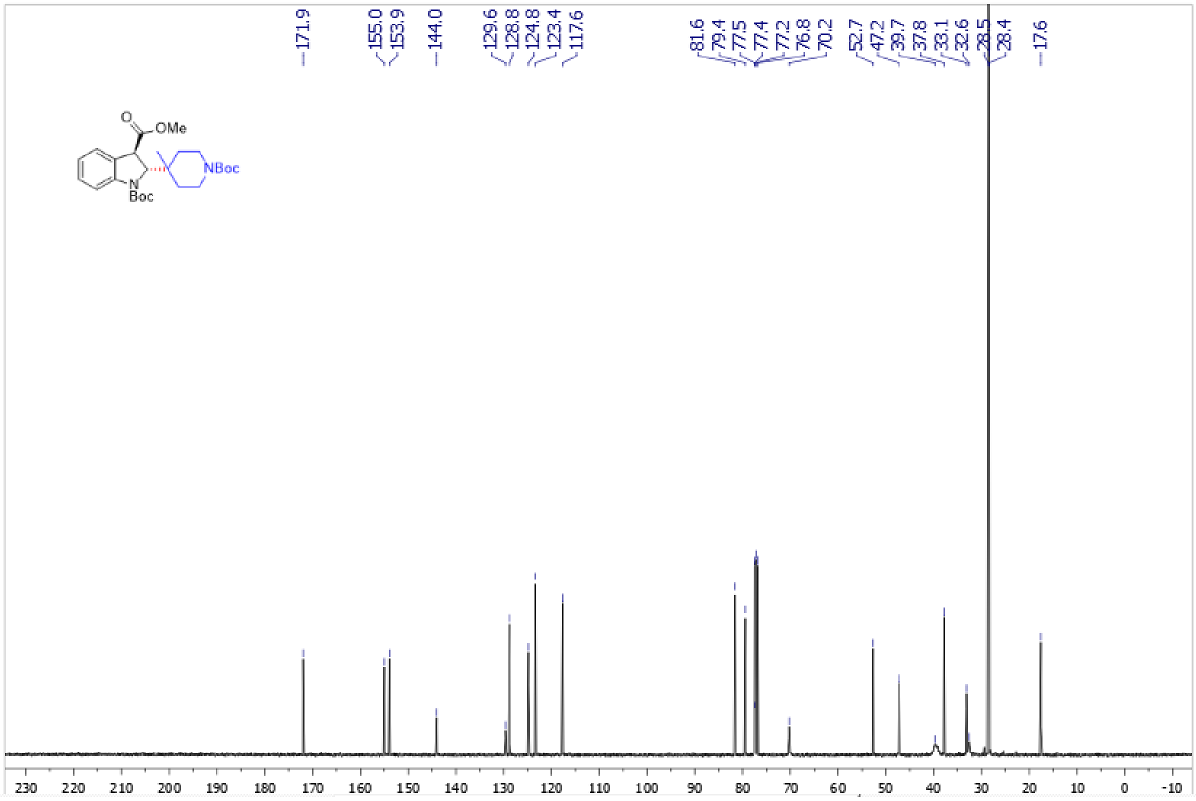

Supplementary Figure 132. ^{13}C NMR spectra of *trans*-**3aq**

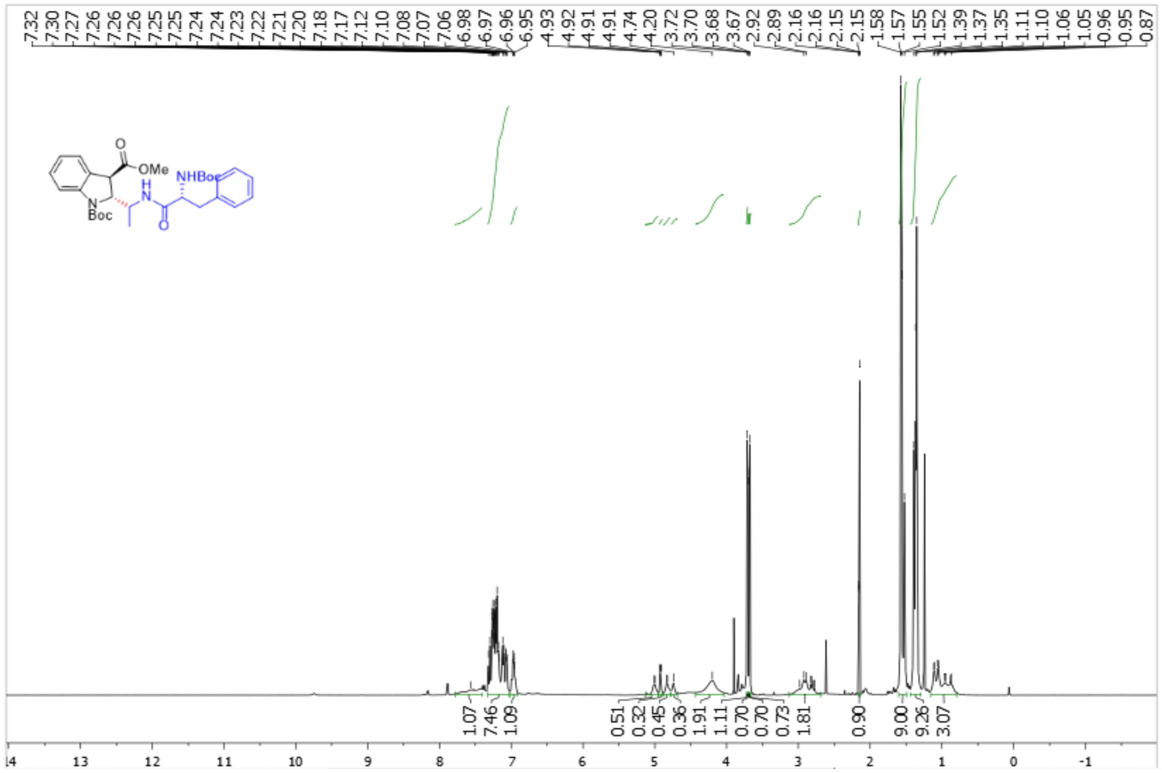

Supplementary Figure 133. ^{1}H NMR spectra of *trans*-**3ar**

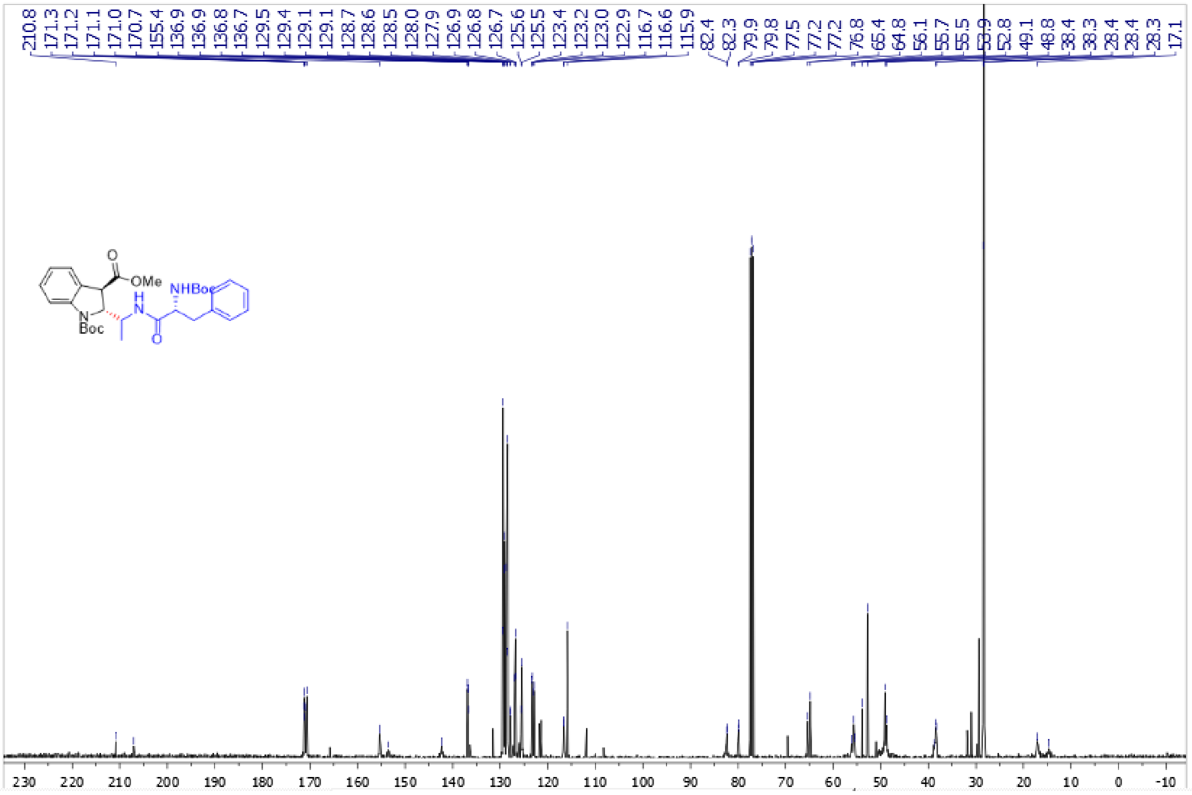

Supplementary Figure 134. ^{13}C NMR spectra of *trans*-**3ar**

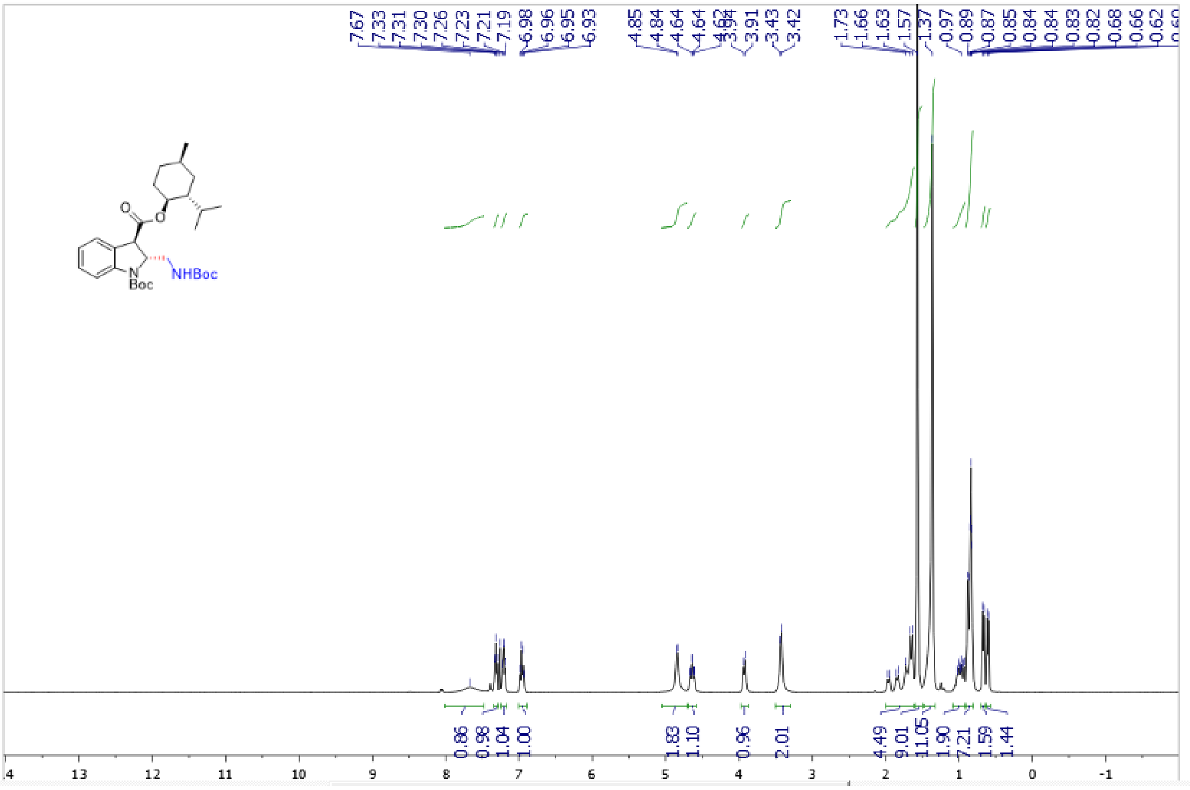

Supplementary Figure 135. ^{1}H NMR spectra of *trans*-**3as**

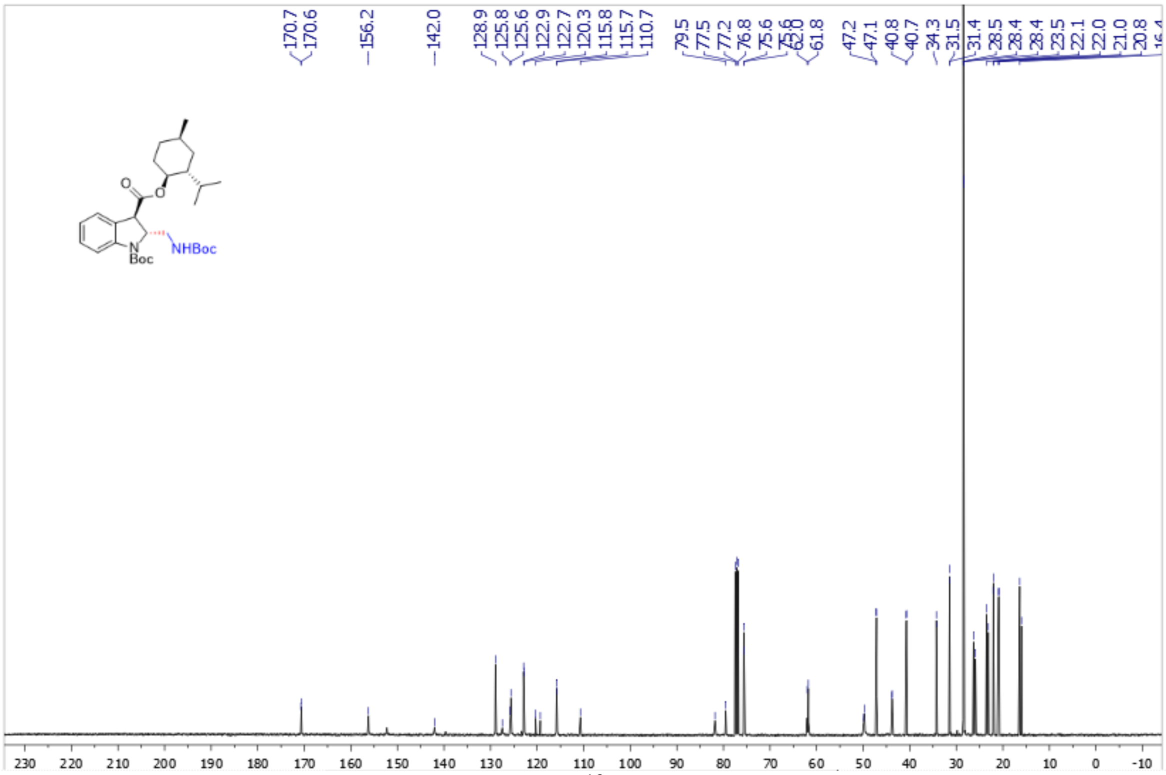

Supplementary Figure 136. ^{13}C NMR spectra of *trans*-**3as**

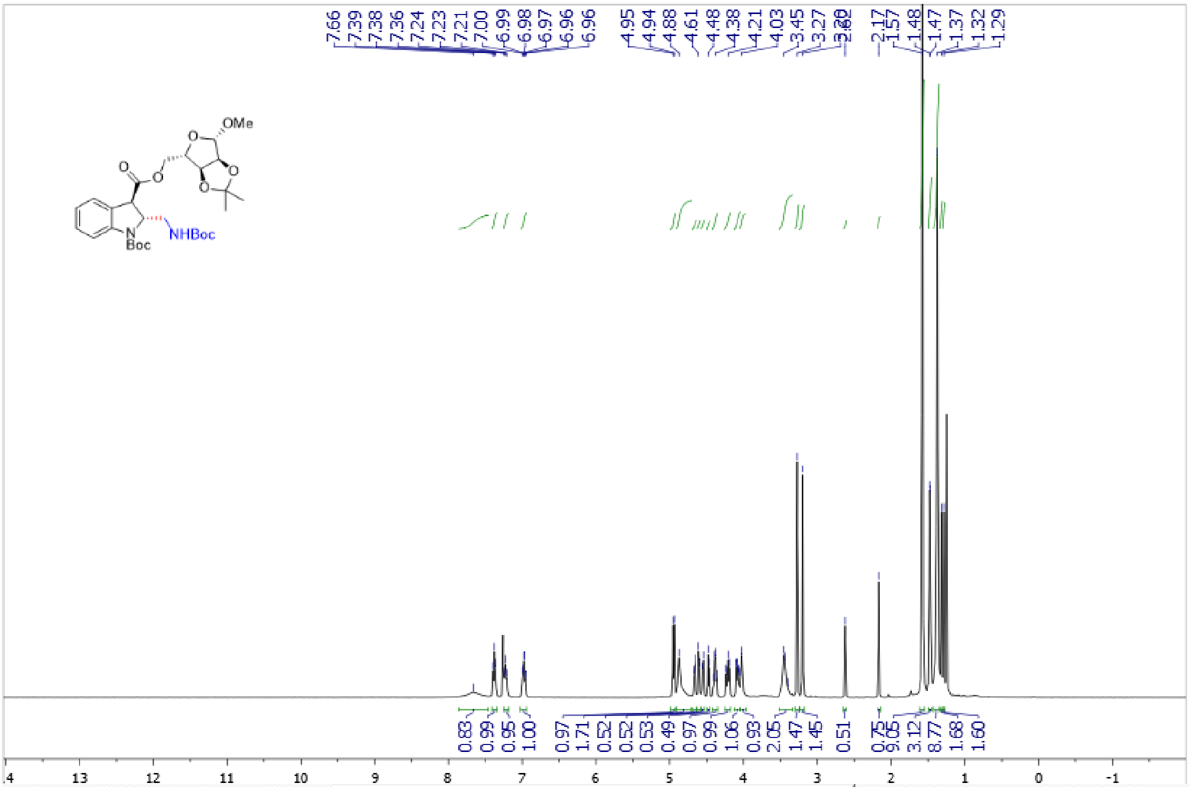

Supplementary Figure 137. ^{1}H NMR spectra of *trans*-**3at**

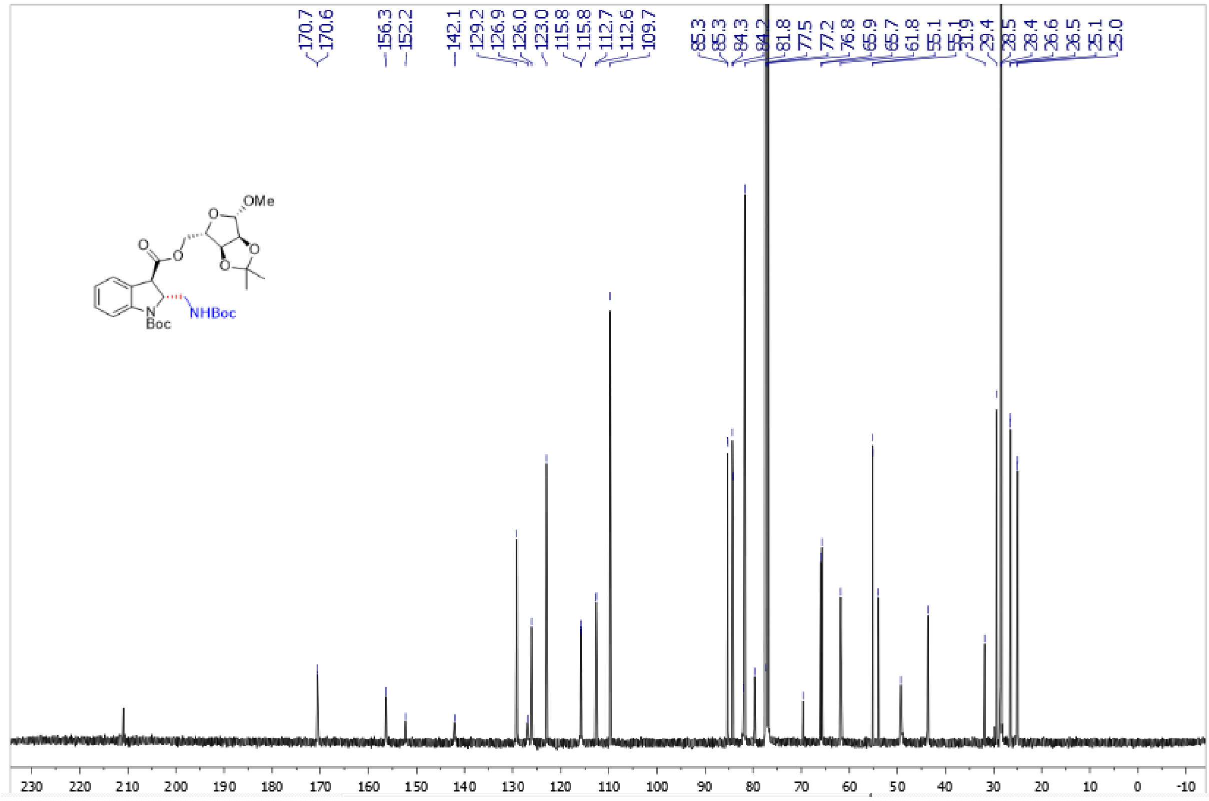

Supplementary Figure 138. ^{13}C NMR spectra of *trans*-**3at**

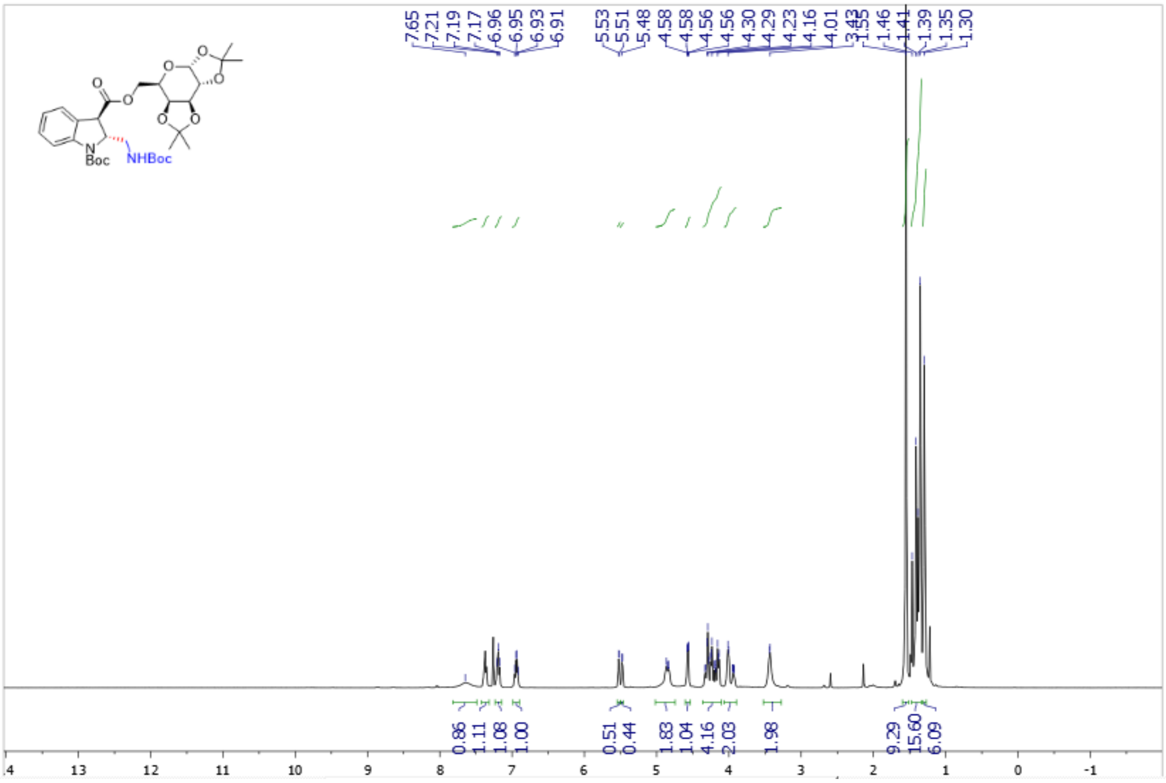

Supplementary Figure 139. ^{1}H NMR spectra of *trans*-**3au**

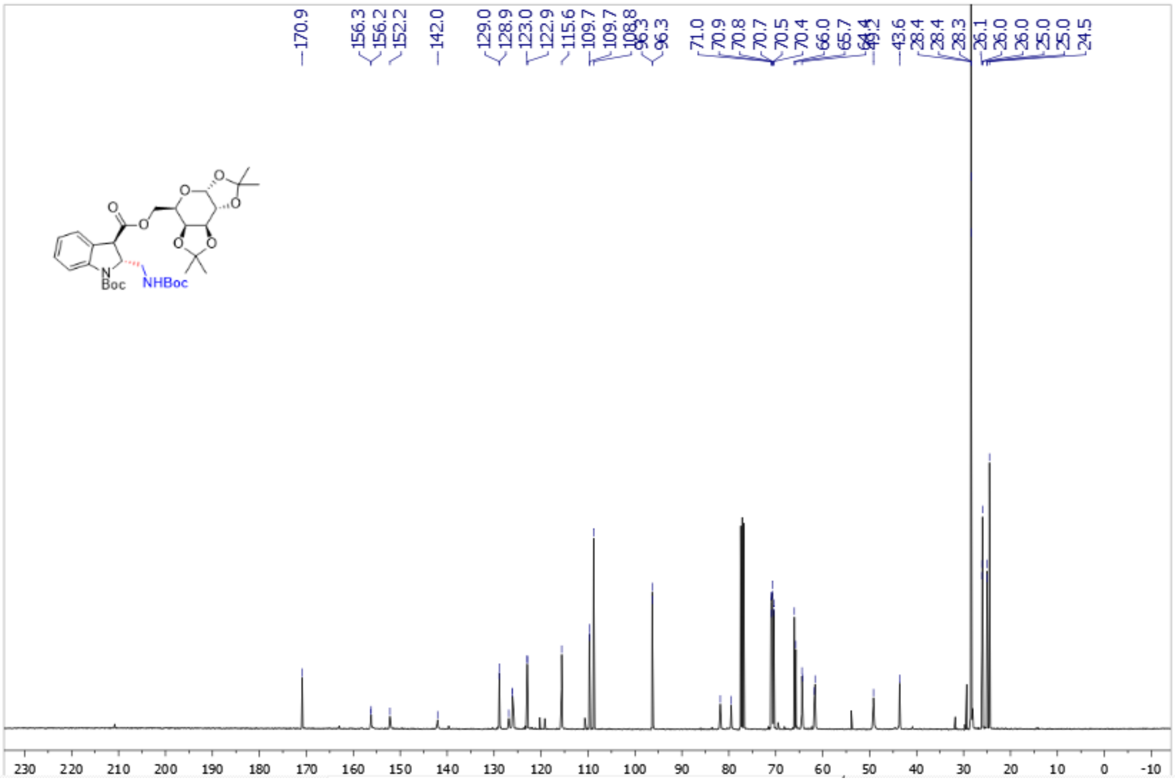

Supplementary Figure 140. ^{13}C NMR spectra of *trans*-**3au**

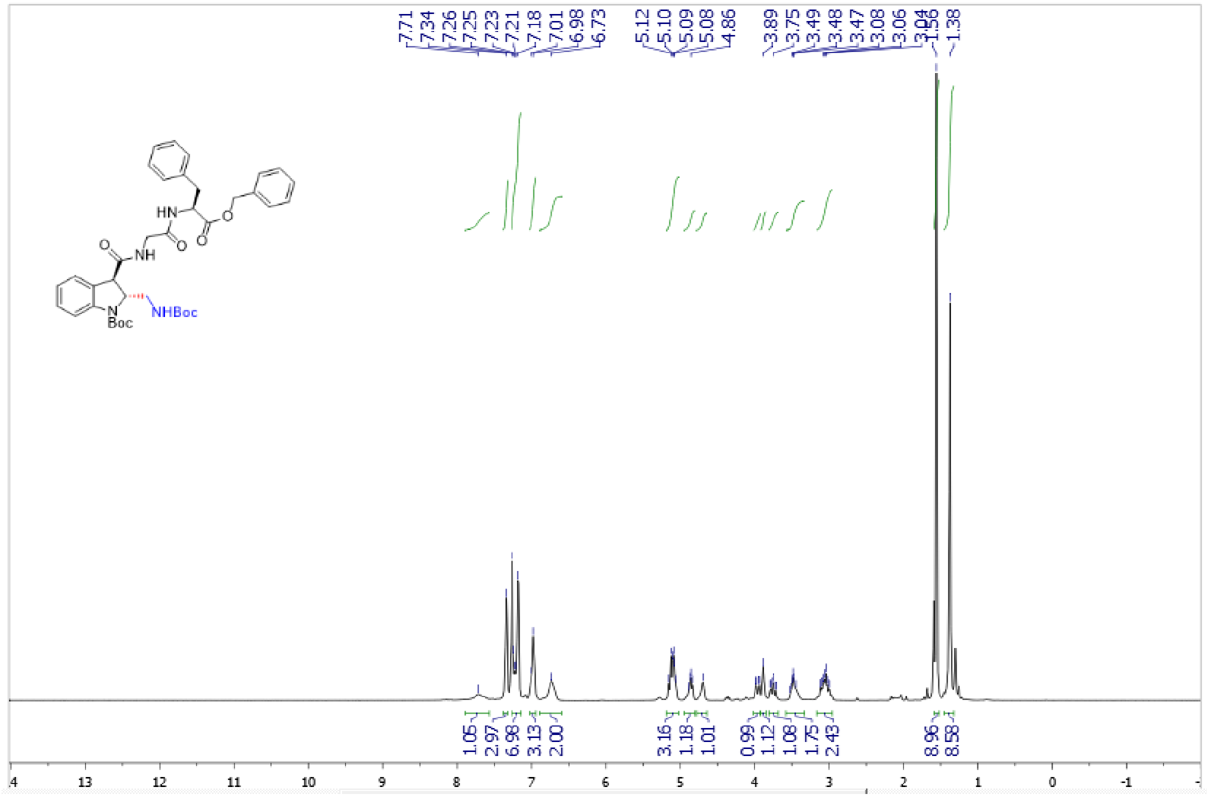

Supplementary Figure 141. ^{1}H NMR spectra of *trans*-**3av**

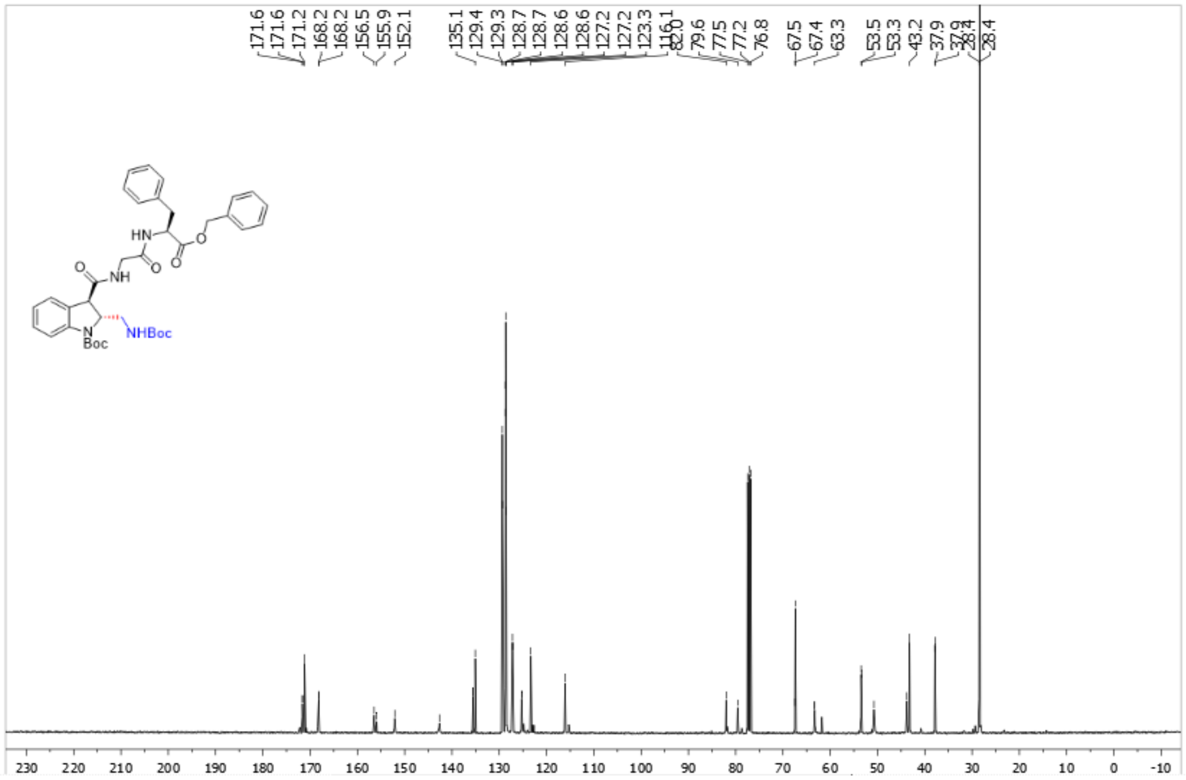

Supplementary Figure 142. ^{13}C NMR spectra of *trans*-**3av**

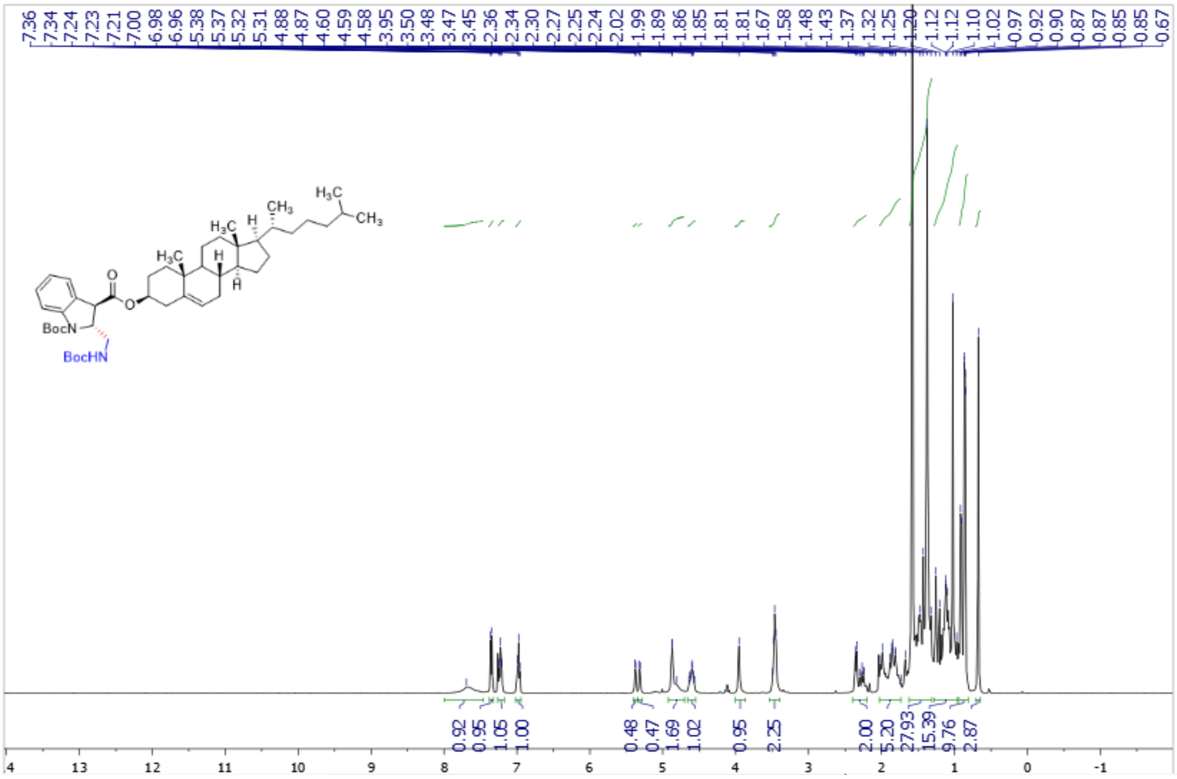

Supplementary Figure 143. ^{1}H NMR spectra of *trans*-**3aw**

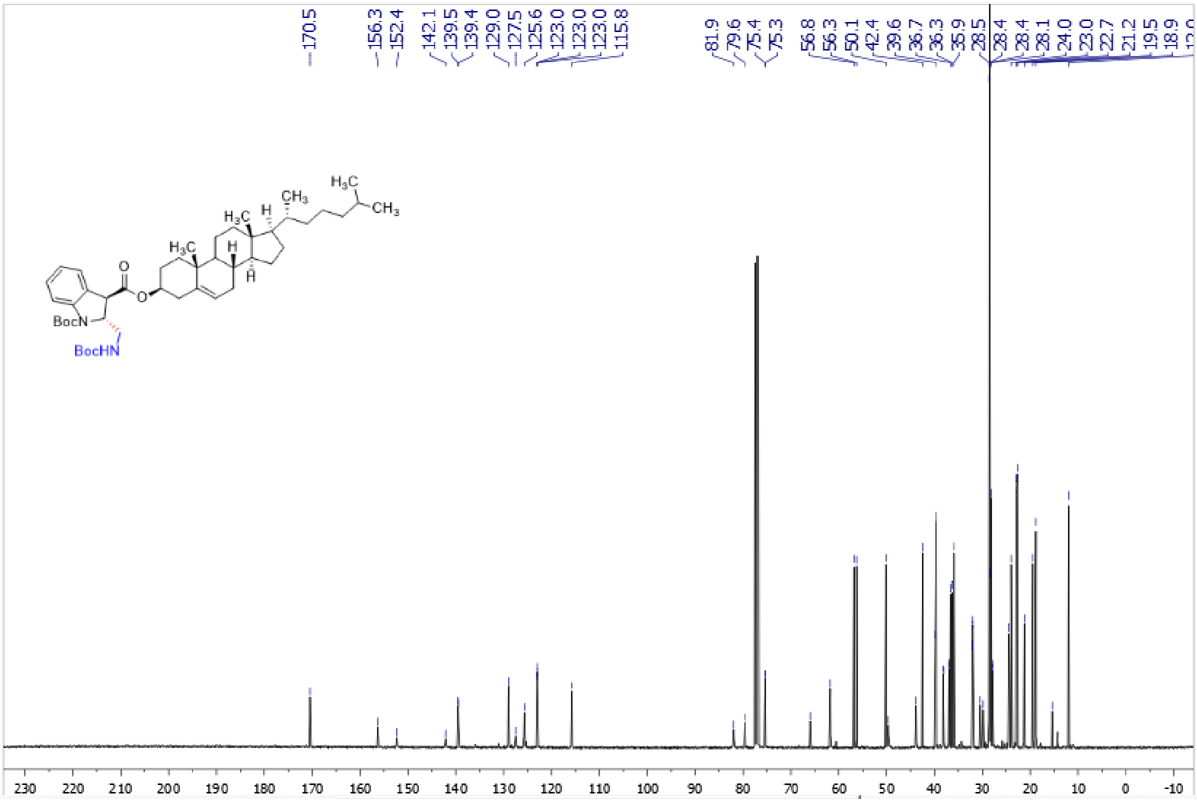

Supplementary Figure 144. ^{13}C NMR spectra of *trans*-**3aw**

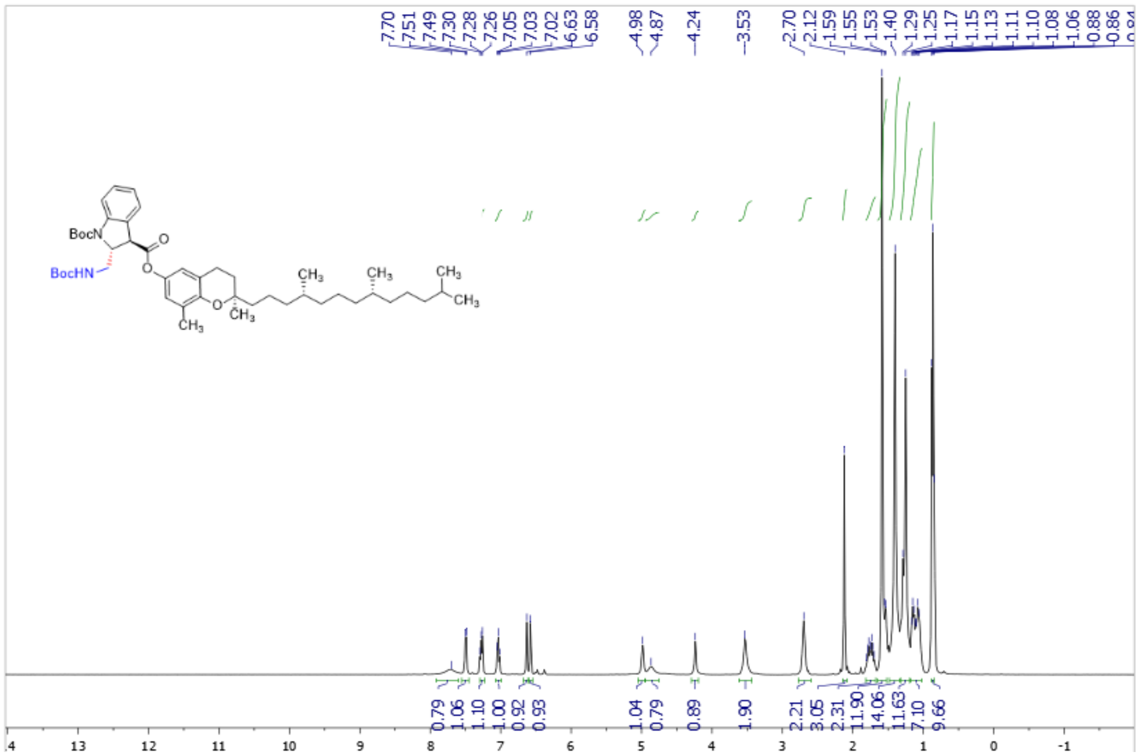

Supplementary Figure 145. ^{1}H NMR spectra of *trans*-**3ax**

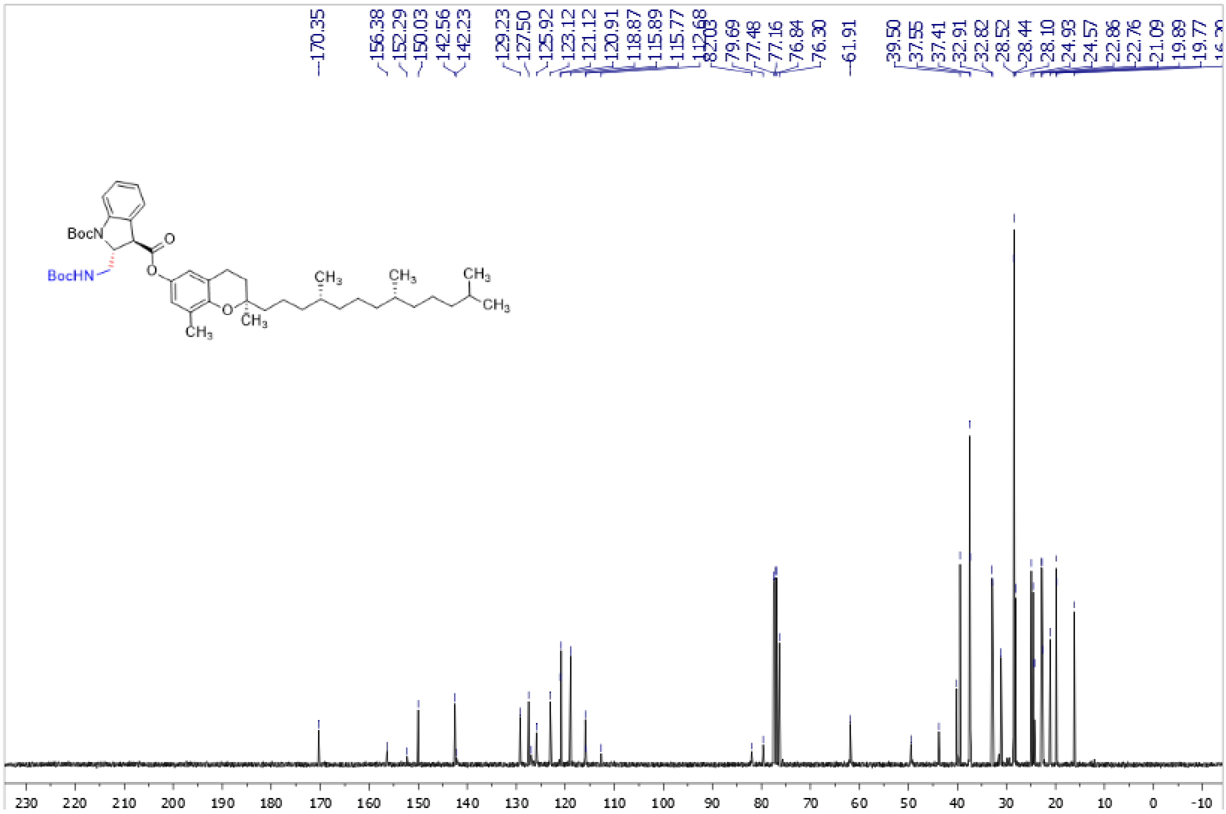

Supplementary Figure 146. ^{13}C NMR spectra of *trans*-**3ax**

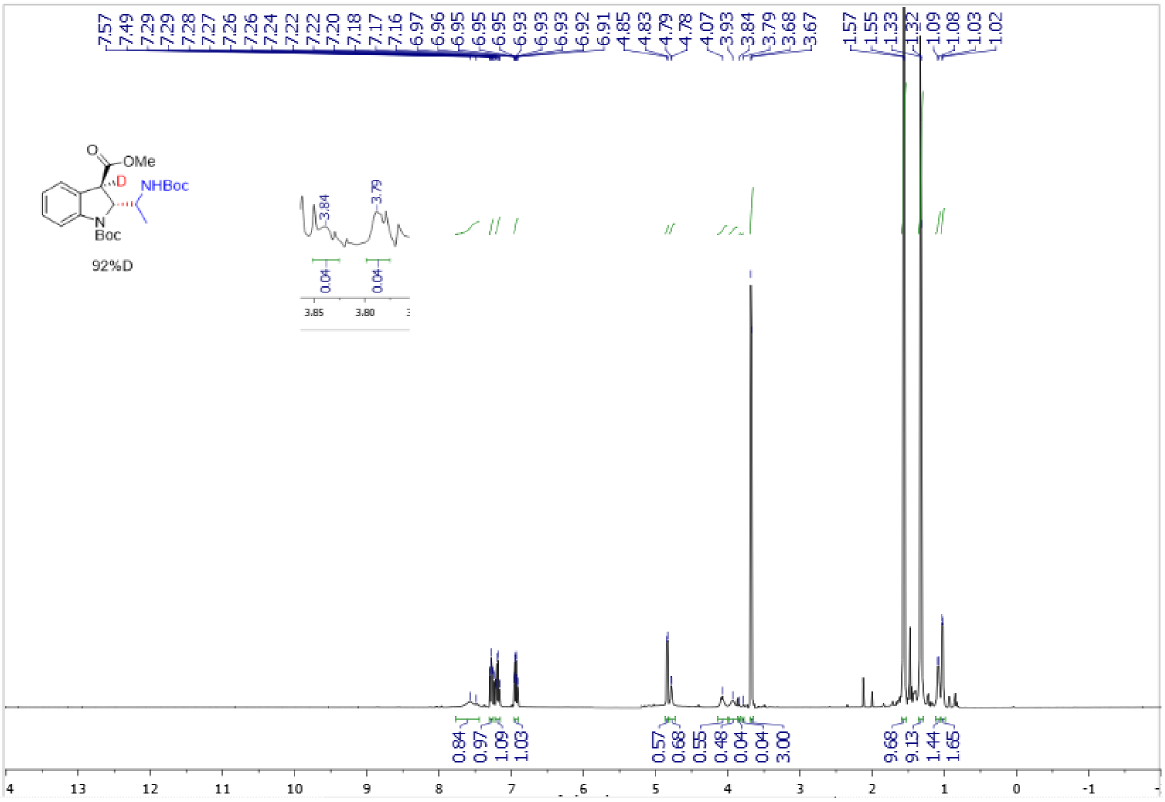

Supplementary Figure 147. ^{1}H NMR spectra of *trans*-**3a-*d***

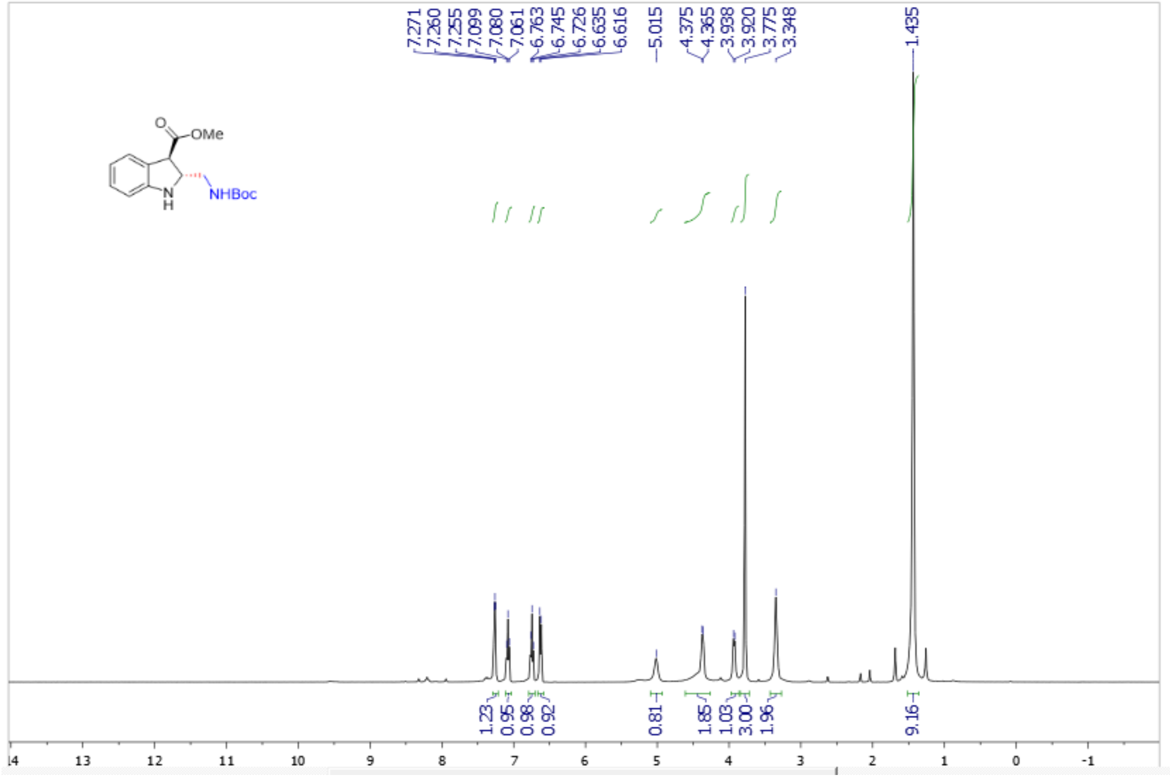

Supplementary Figure 148. ^{1}H NMR spectra of *trans*-**4k**

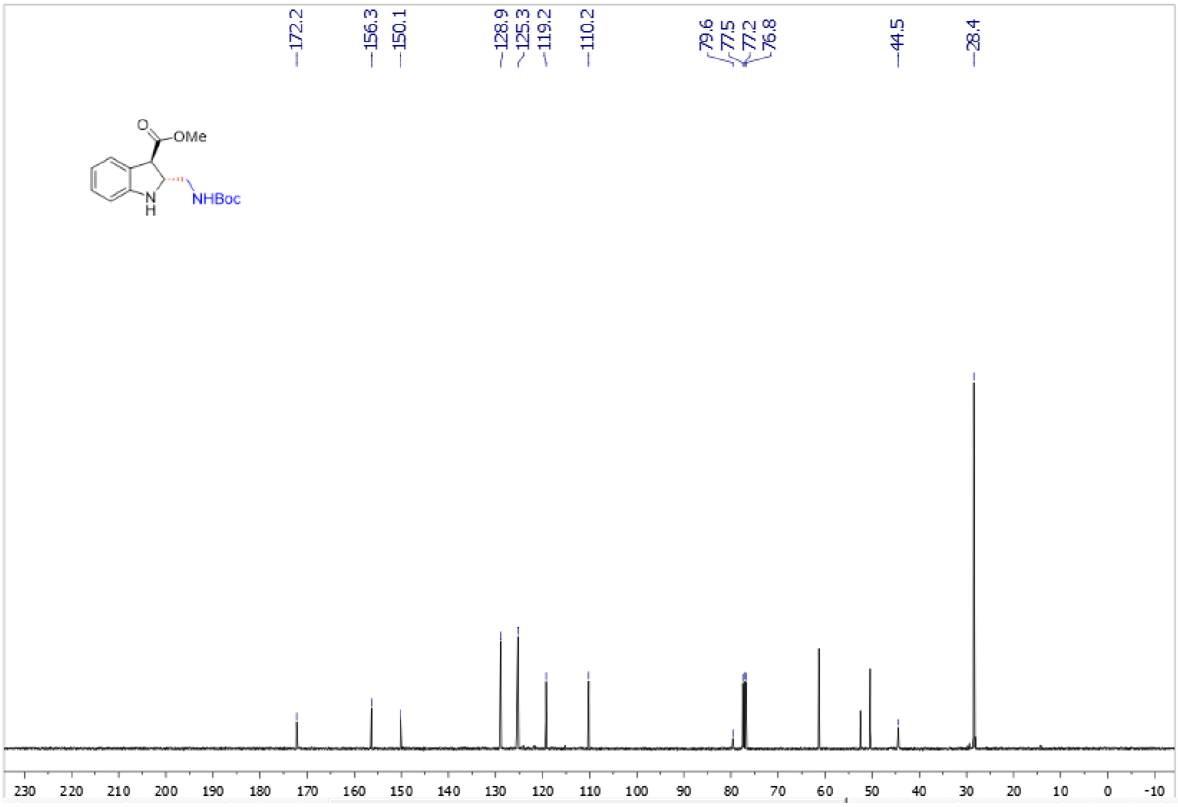

Supplementary Figure 149. ^{13}C NMR spectra of *trans*-**4k**

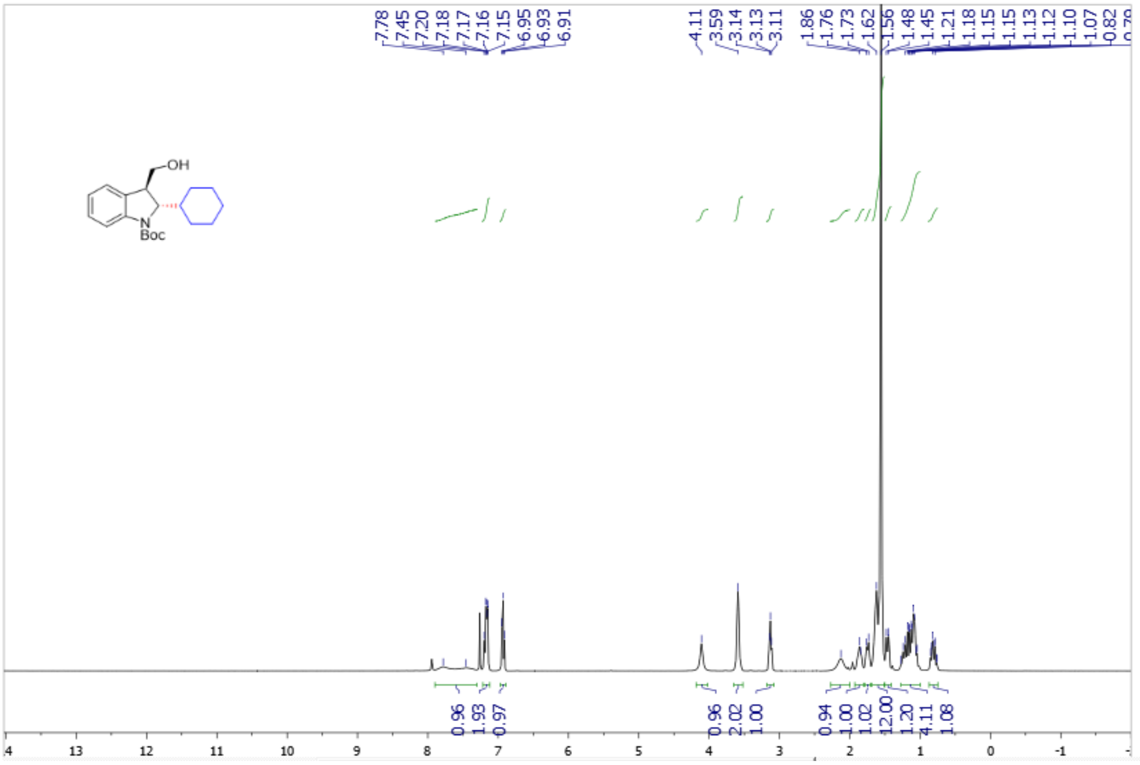

Supplementary Figure 150. ^{1}H NMR spectra of *trans*-**4aj**

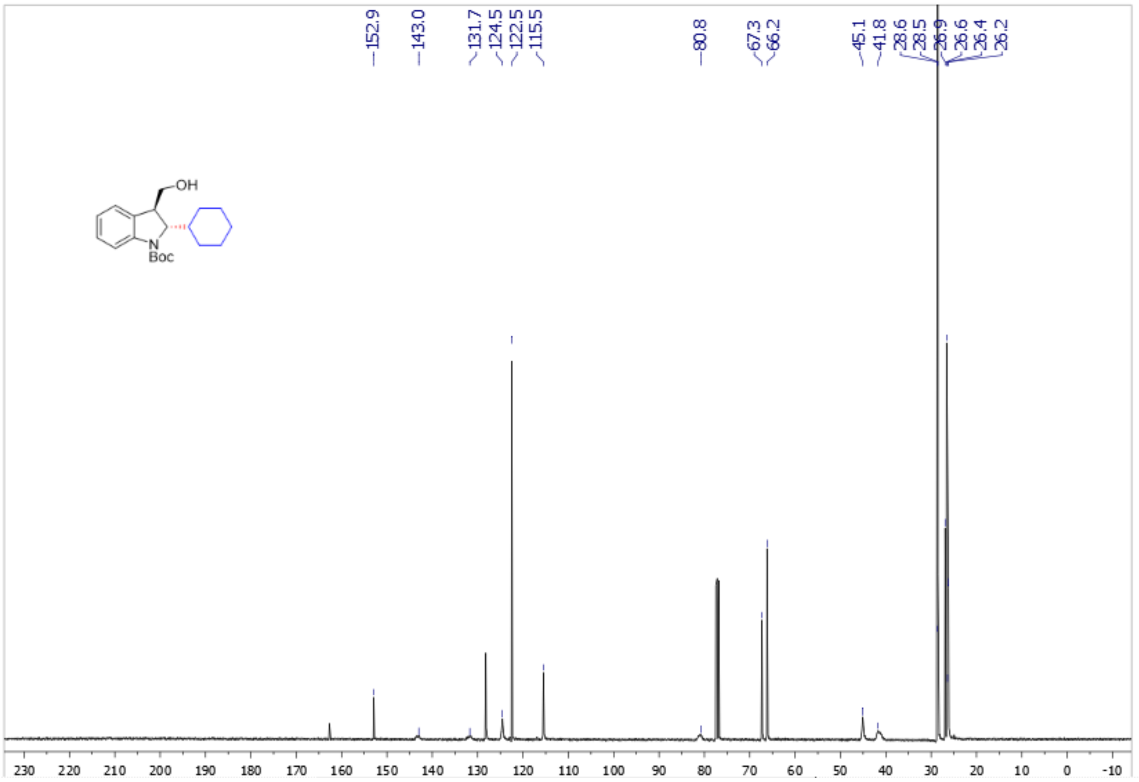

Supplementary Figure 151. ^{13}C NMR spectra of *trans*-**4aj**

For original ^{1}H and ^{13}C NMR spetra of **7a, 7g, 7h**, **7p, 7q, 7r, 7s, 7t, 7u, 7v,** and **7w**, please see corresponding **3v, 3ah, 3ag**, **3aj, 3al, 3am, 3aq, 3ae, 3ap, 3an** and **3k**.

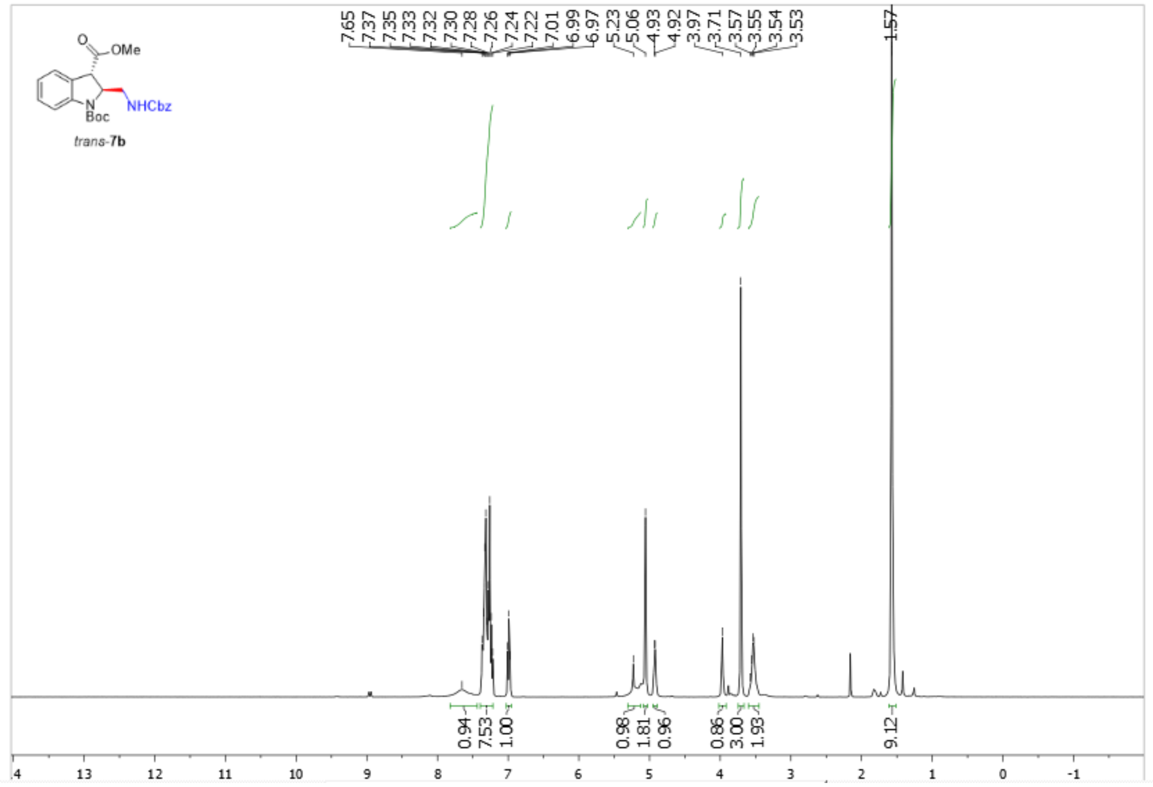

Supplementary Figure 152. ^{1}H NMR spectra of *trans*-**7b**

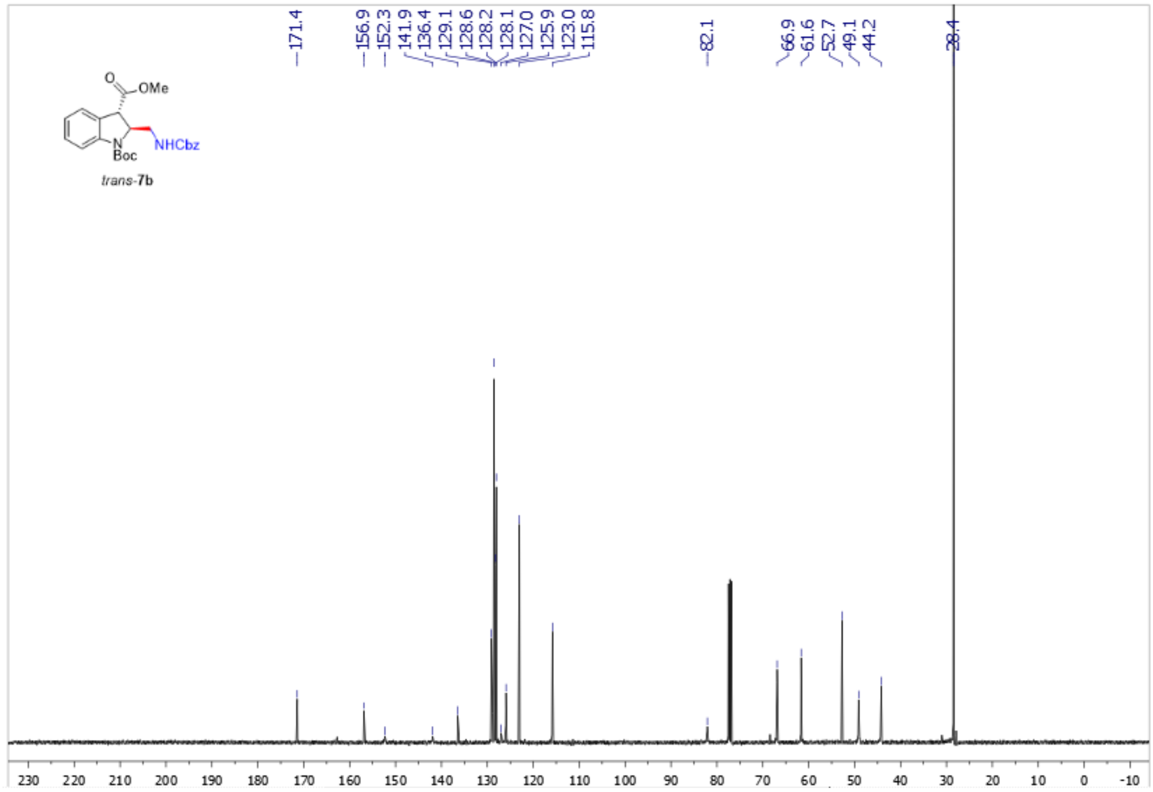

Supplementary Figure 153. ^{13}C NMR spectra of *trans*-**7b**

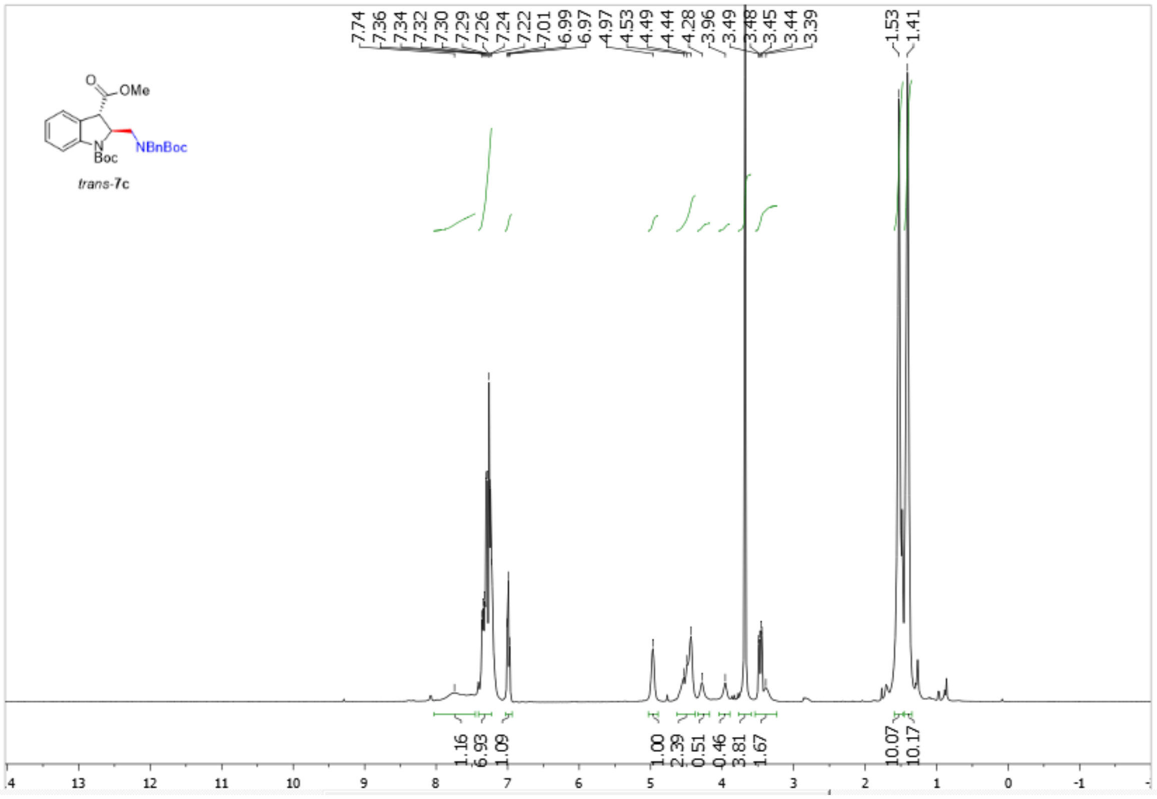

Supplementary Figure 154. ^{1}H NMR spectra of *trans*-**7c**

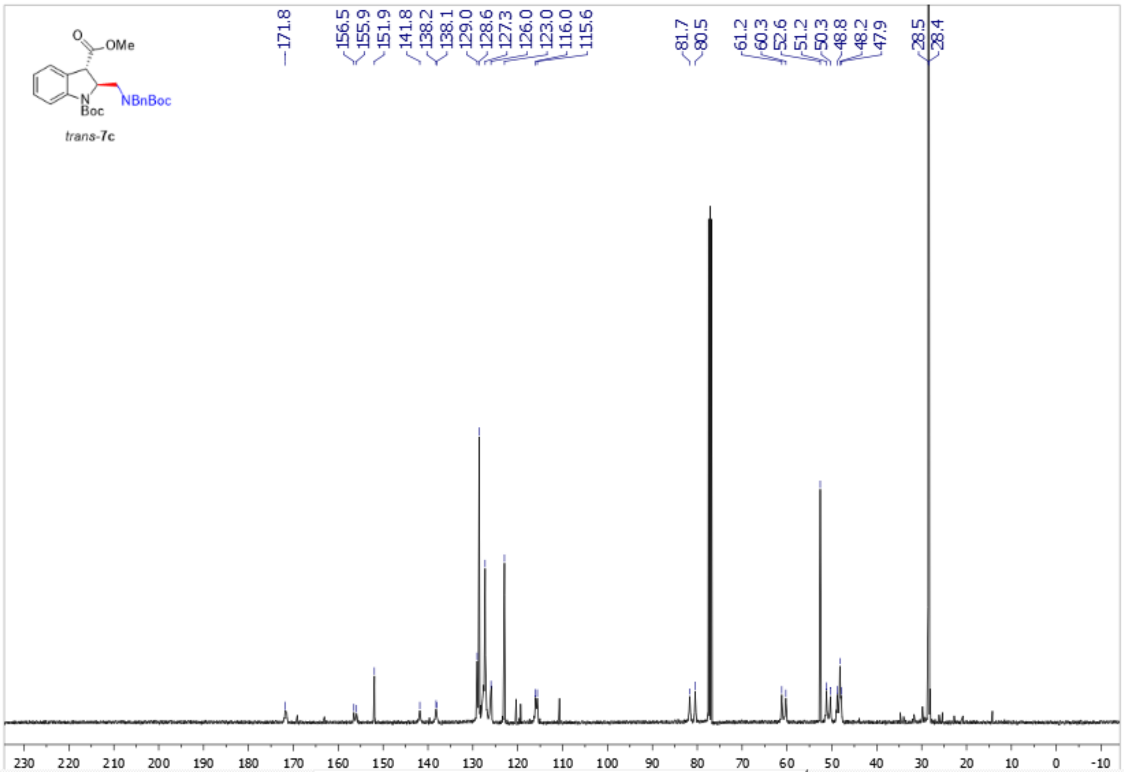

Supplementary Figure 155. ^{13}C NMR spectra of *trans*-**7c**

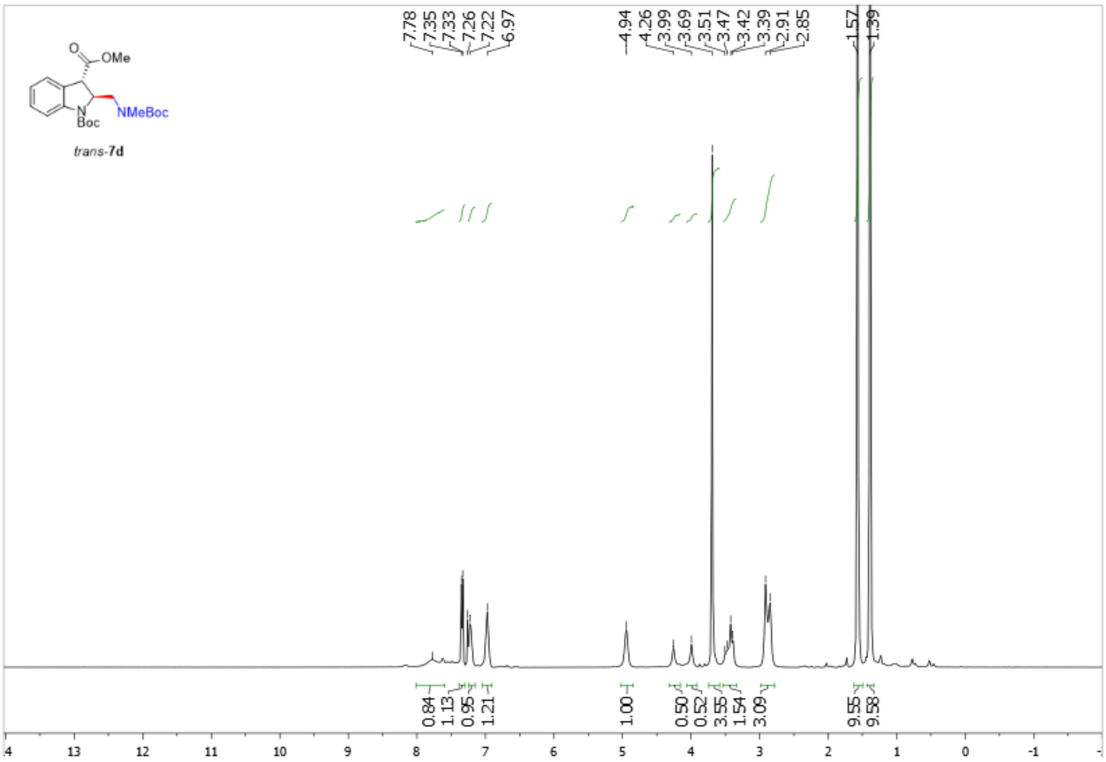

Supplementary Figure 156. ^{1}H NMR spectra of *trans*-**7d**

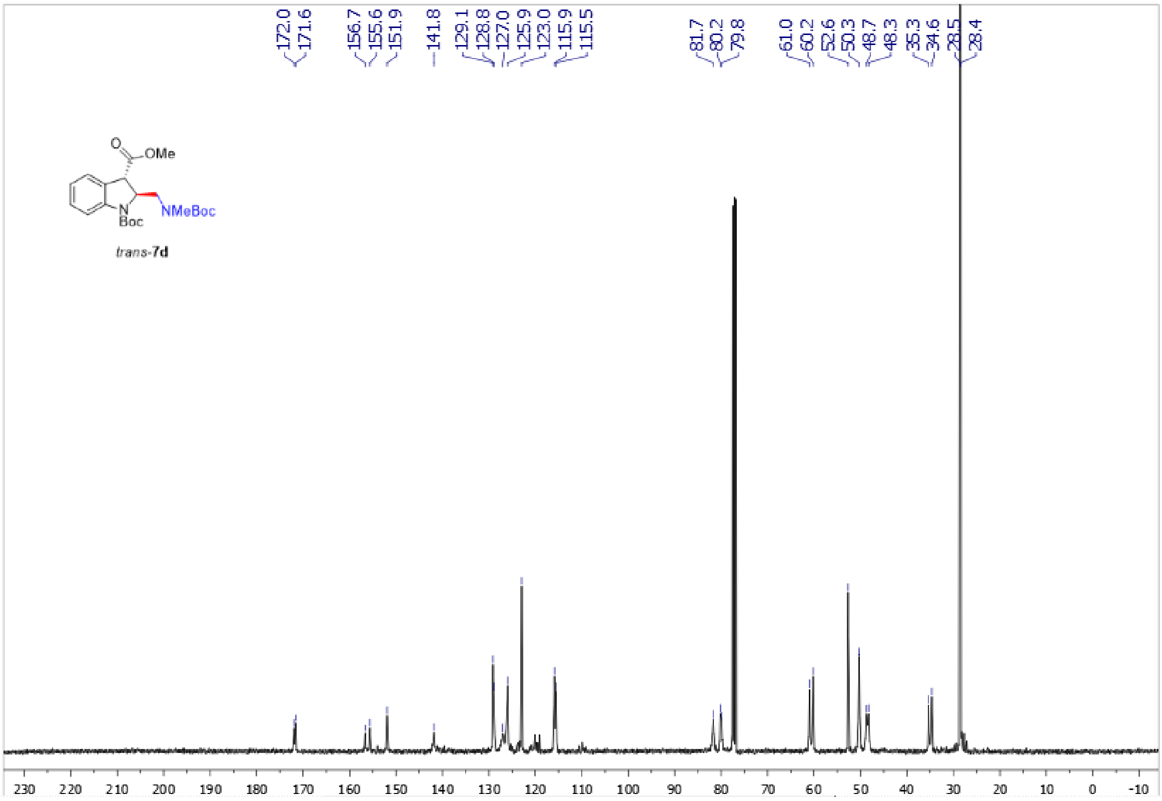

Supplementary Figure 157. ^{13}C NMR spectra of *trans*-**7d**

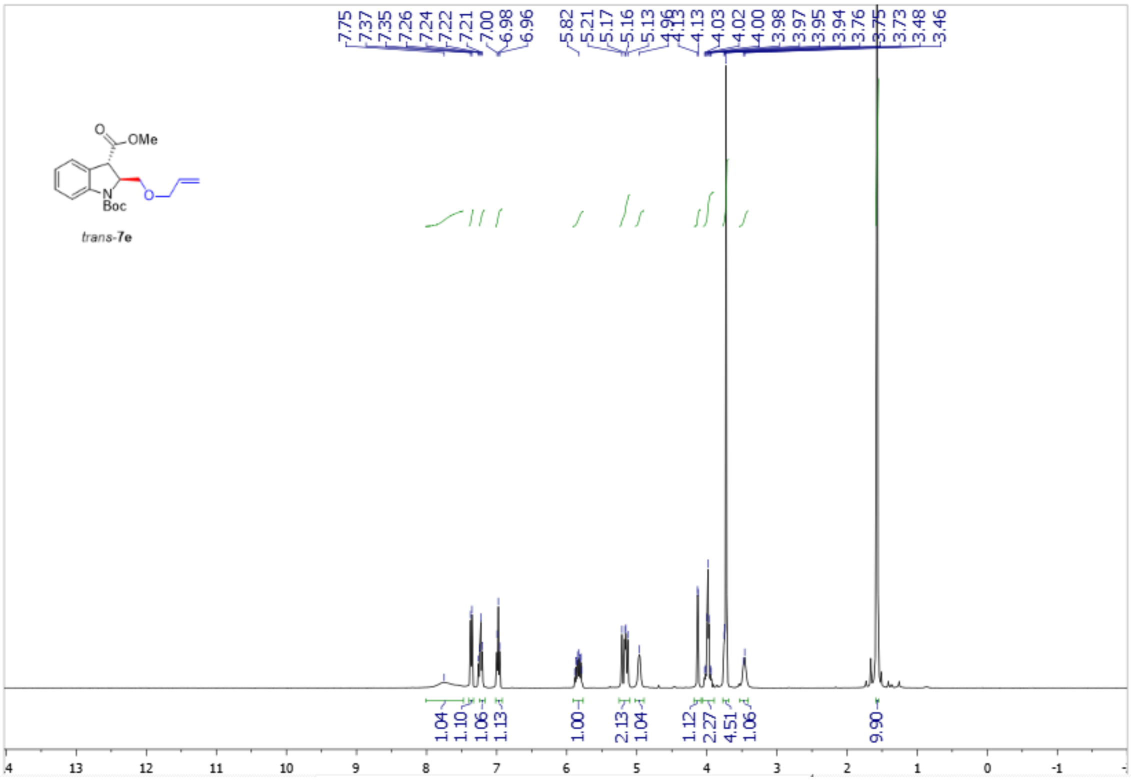

Supplementary Figure 158. ^{1}H NMR spectra of *trans*-**7e**

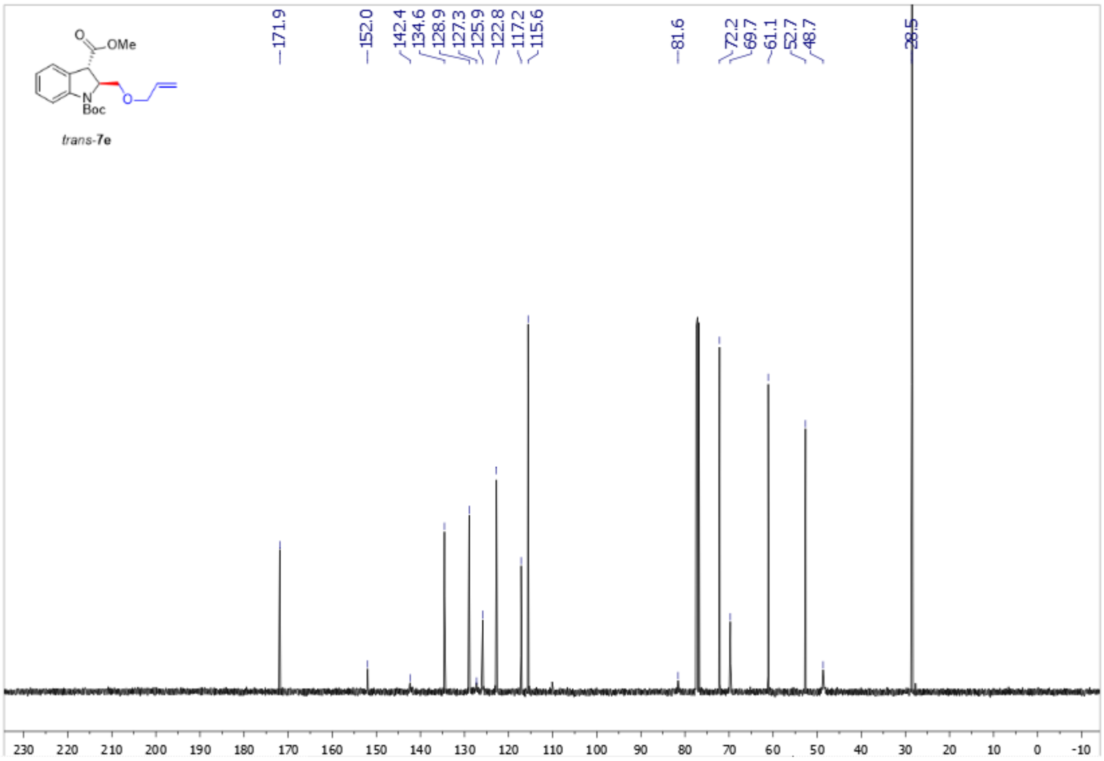

Supplementary Figure 159. ^{13}C NMR spectra of *trans*-**7e**

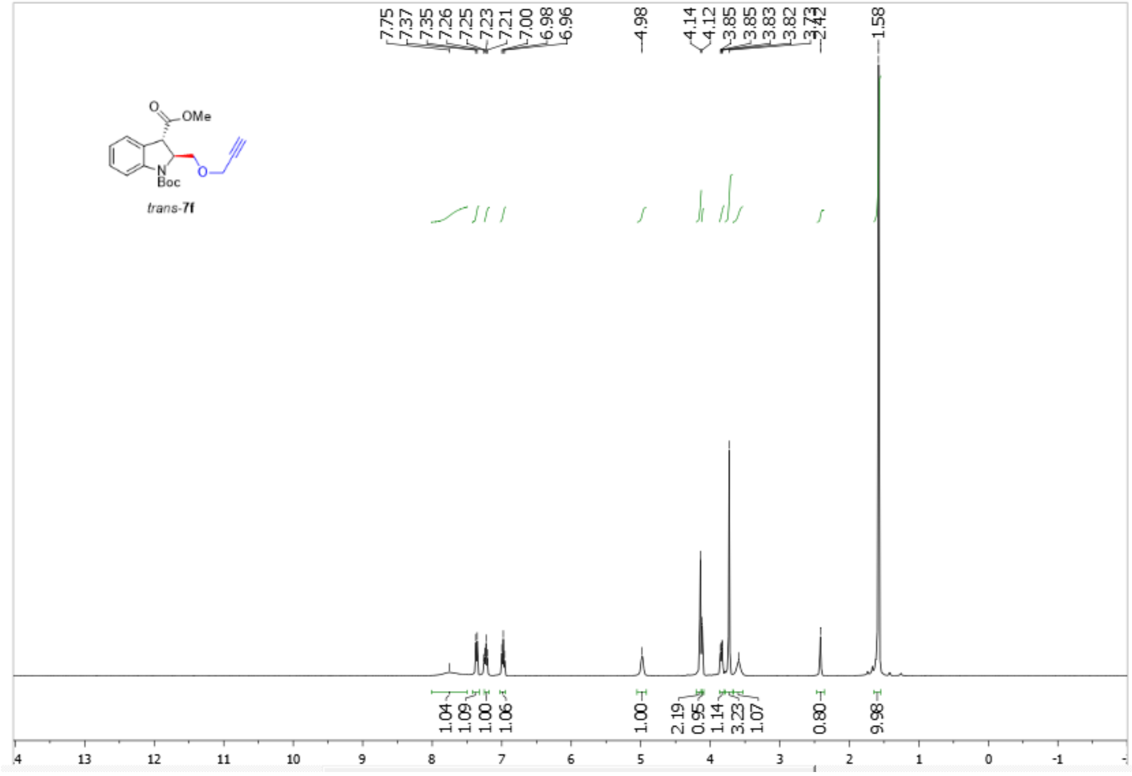

Supplementary Figure 160. ^{1}H NMR spectra of *trans*-**7f**

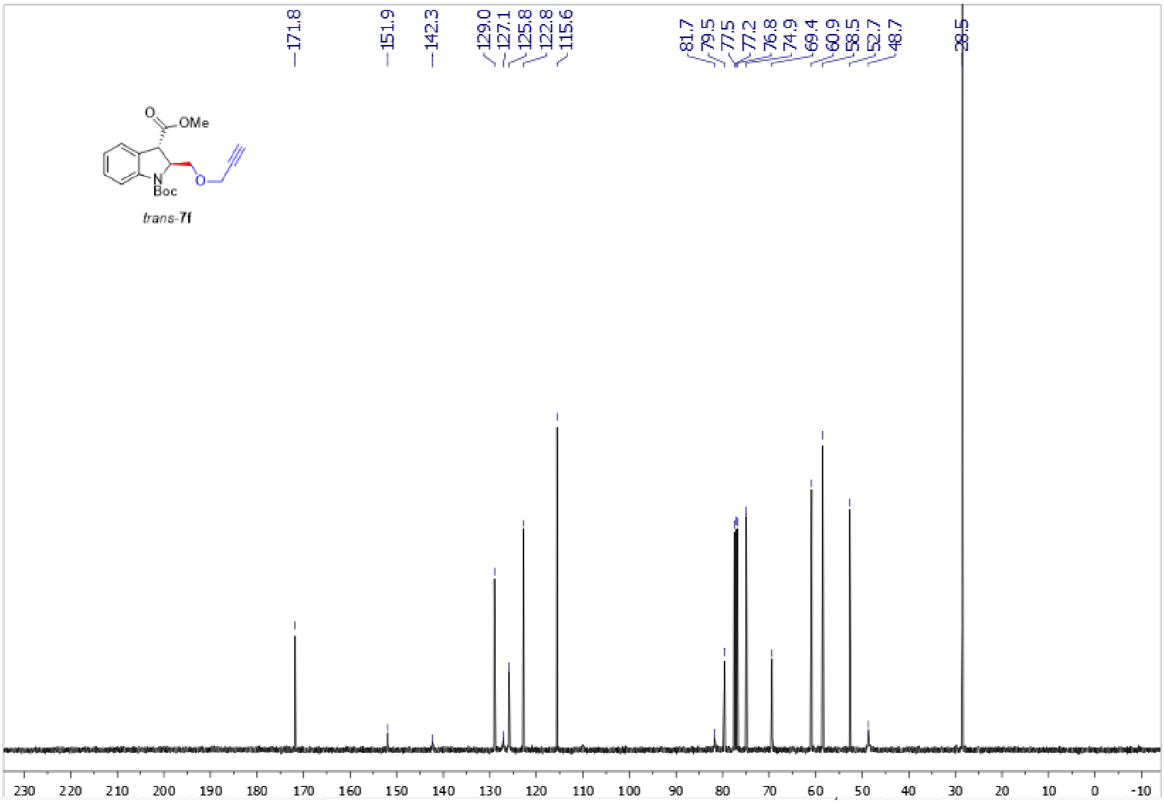

Supplementary Figure 161. ^{13}C NMR spectra of *trans*-**7f**

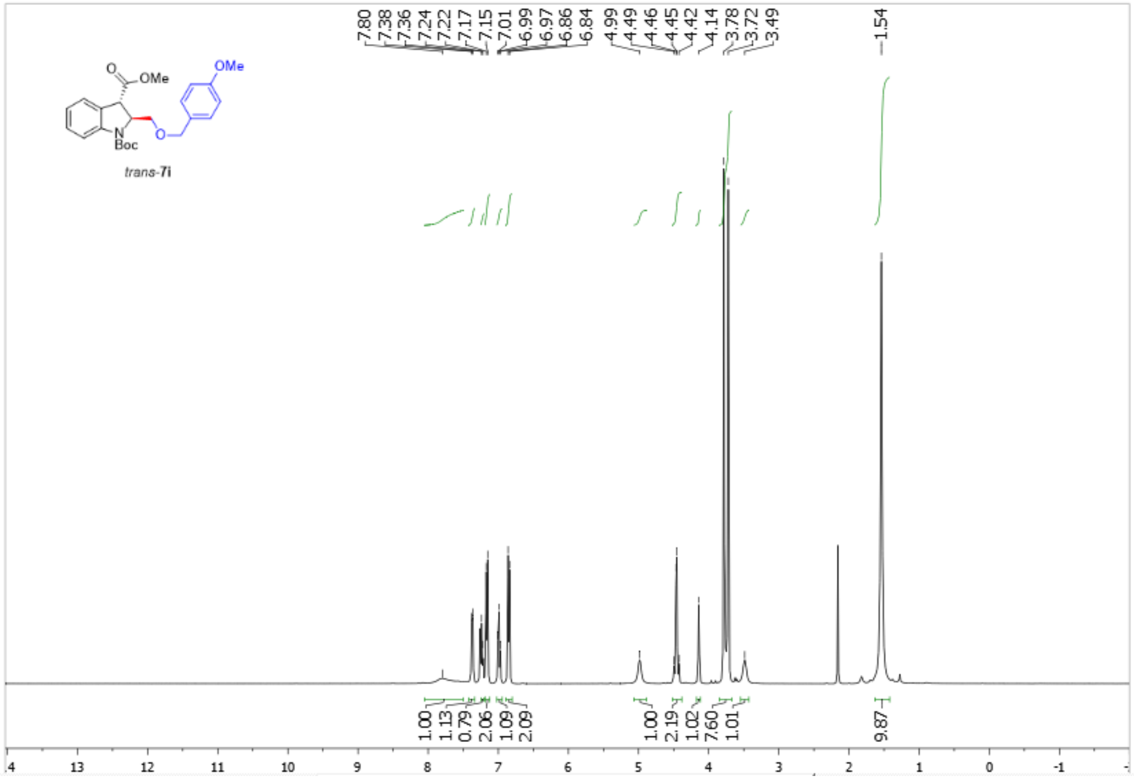

Supplementary Figure 162. ^{1}H NMR spectra of *trans*-**7i**

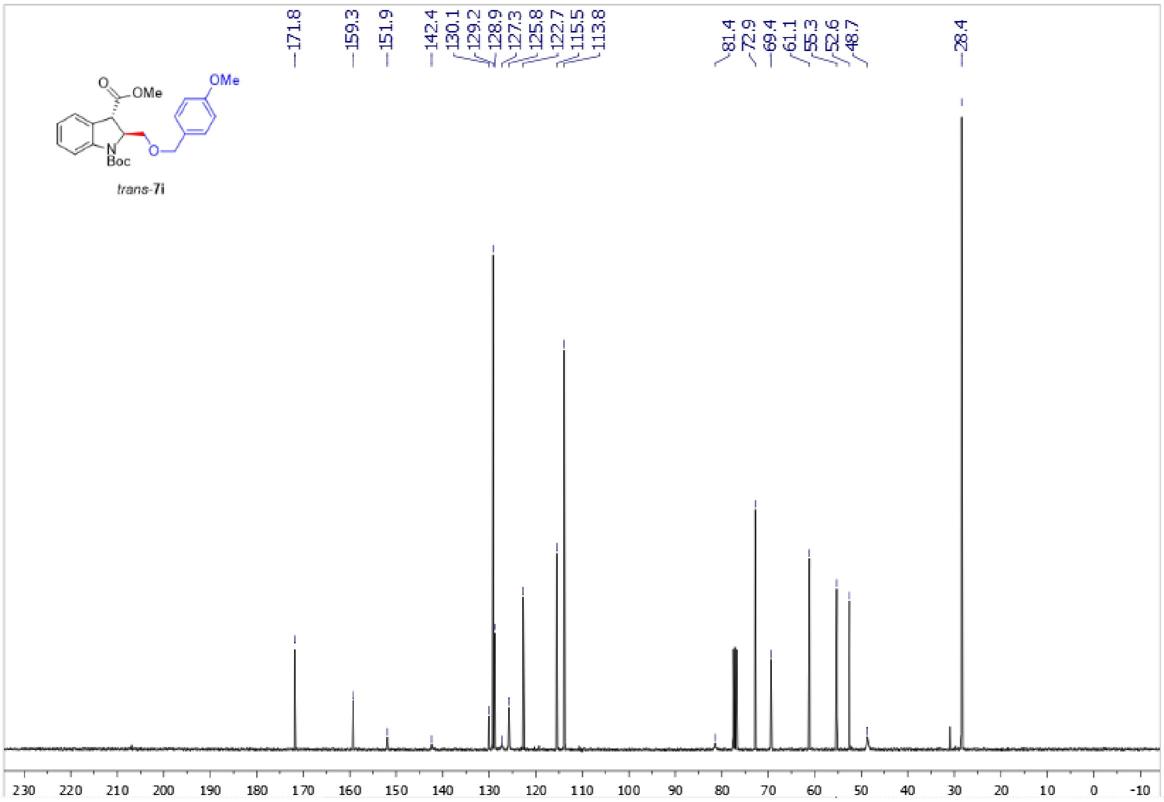

Supplementary Figure 163. ^{13}C NMR spectra of *trans*-**7i**

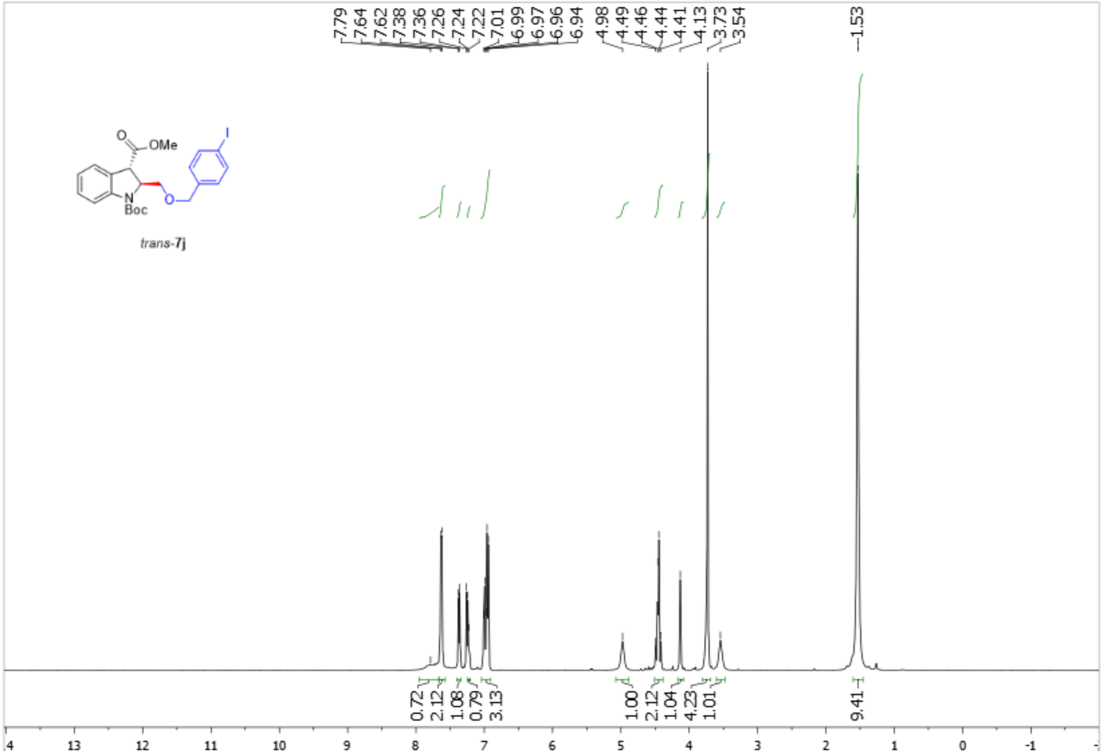

Supplementary Figure 164. ^{1}H NMR spectra of *trans*-**7j**

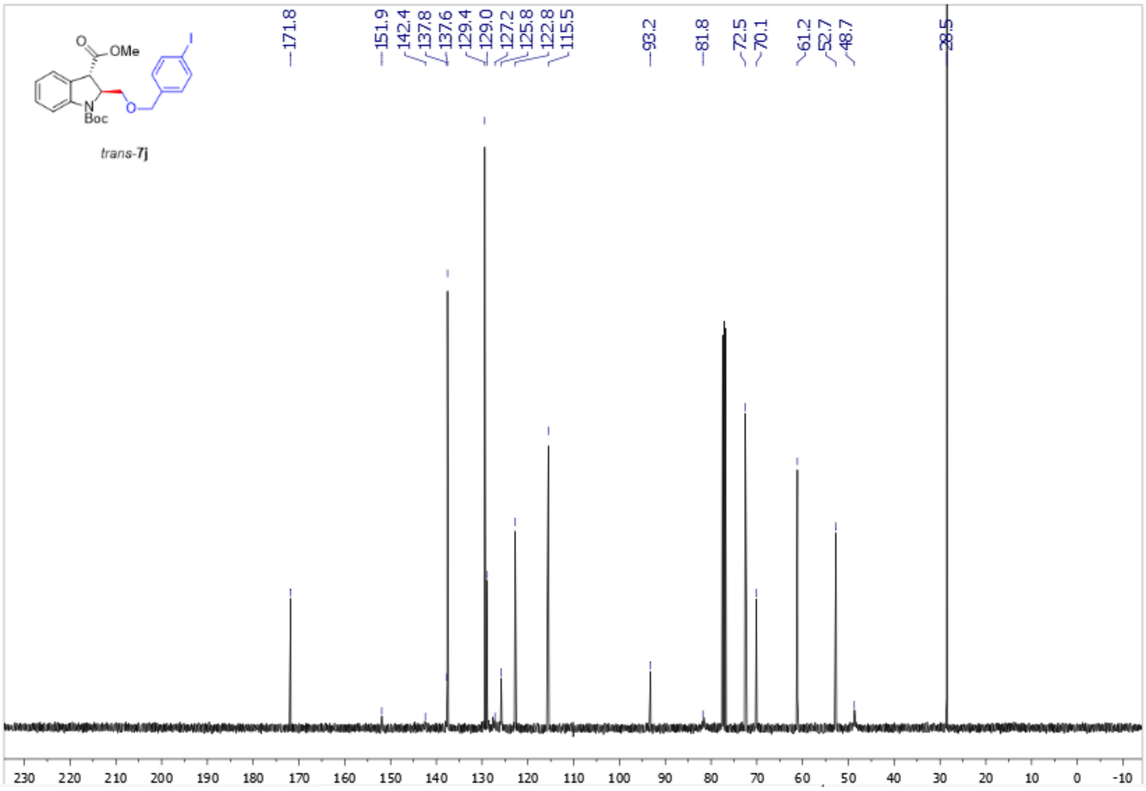

Supplementary Figure 165. ^{13}C NMR spectra of *trans*-**7j**

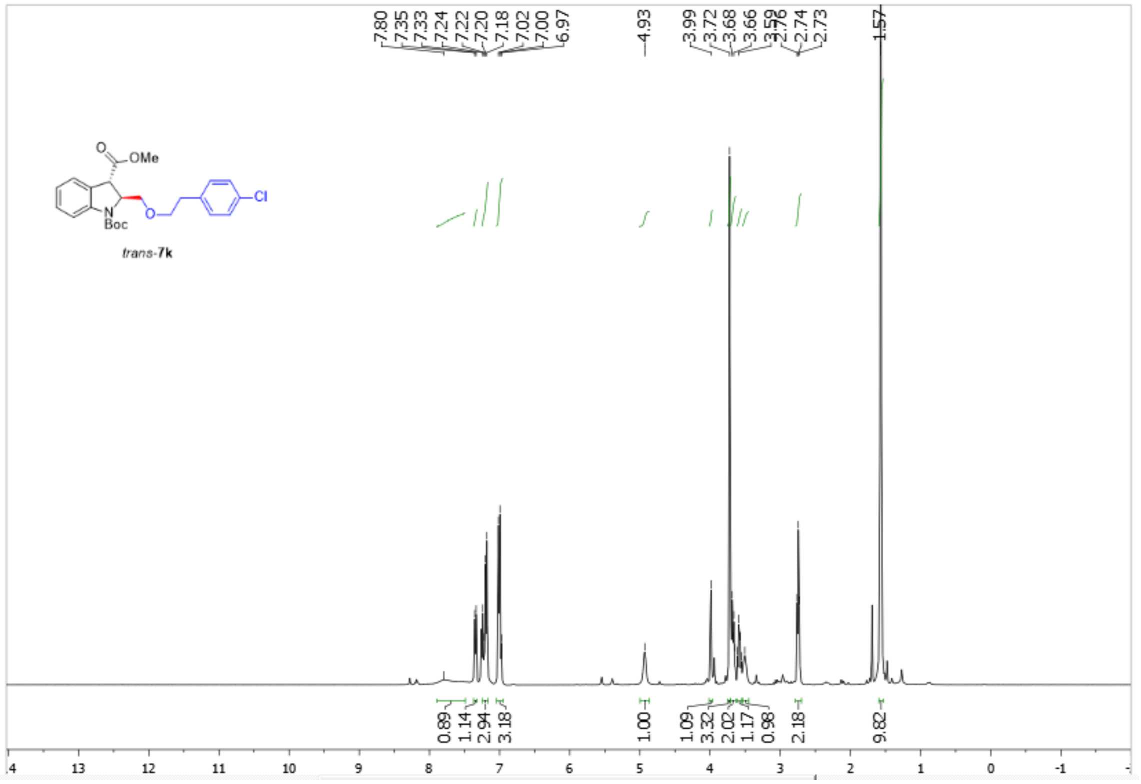

Supplementary Figure 166. ^{1}H NMR spectra of *trans*-**7k**

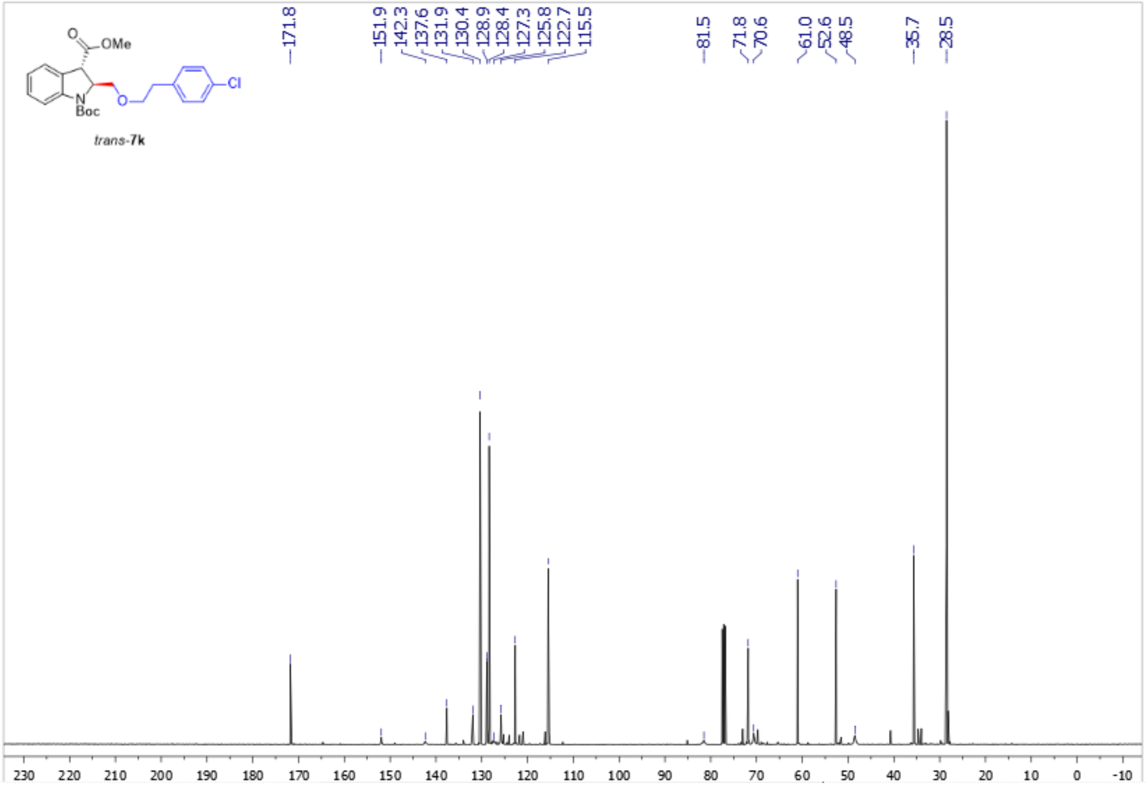

Supplementary Figure 167. ^{13}C NMR spectra of *trans*-**7k**

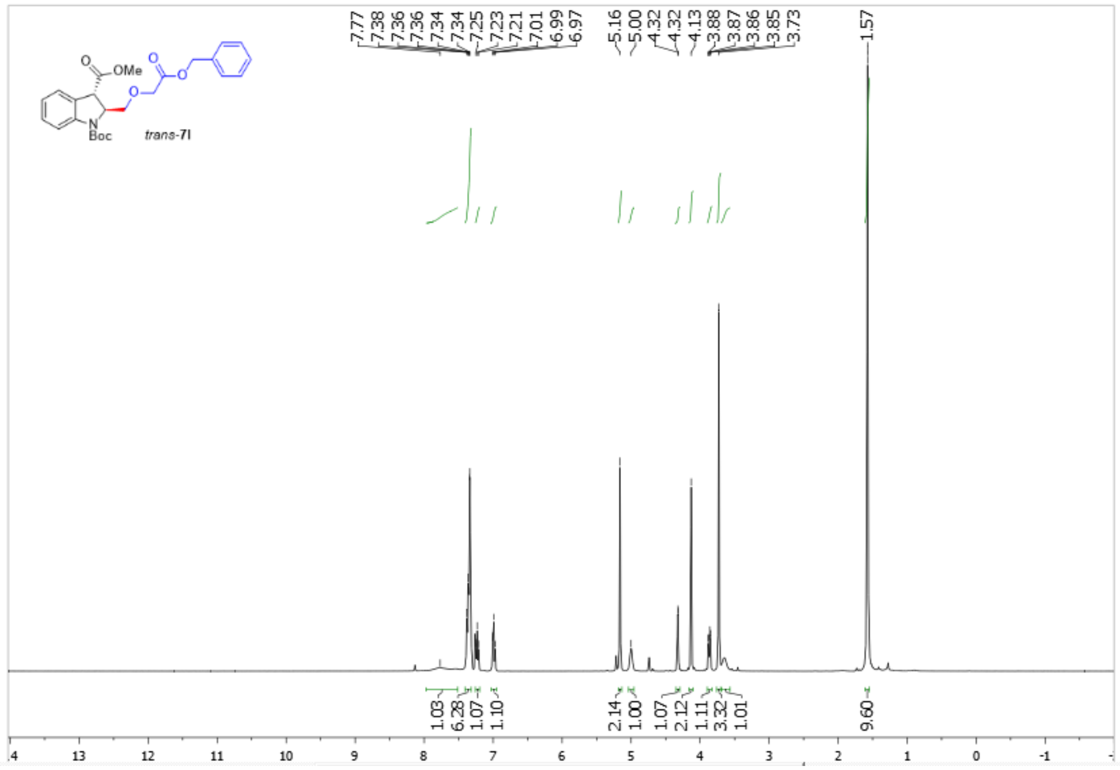

Supplementary Figure 168. ^{1}H NMR spectra of *trans*-**7l**

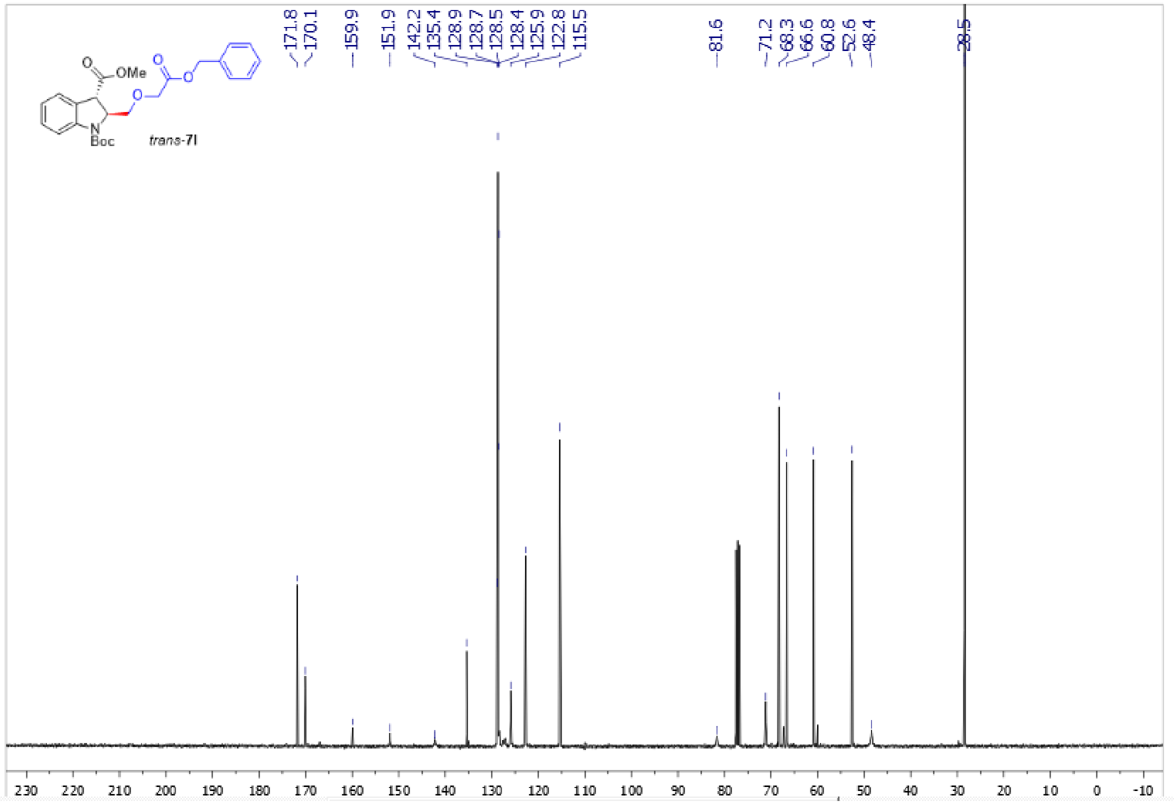

Supplementary Figure 169. ^{13}C NMR spectra of *trans*-**7l**

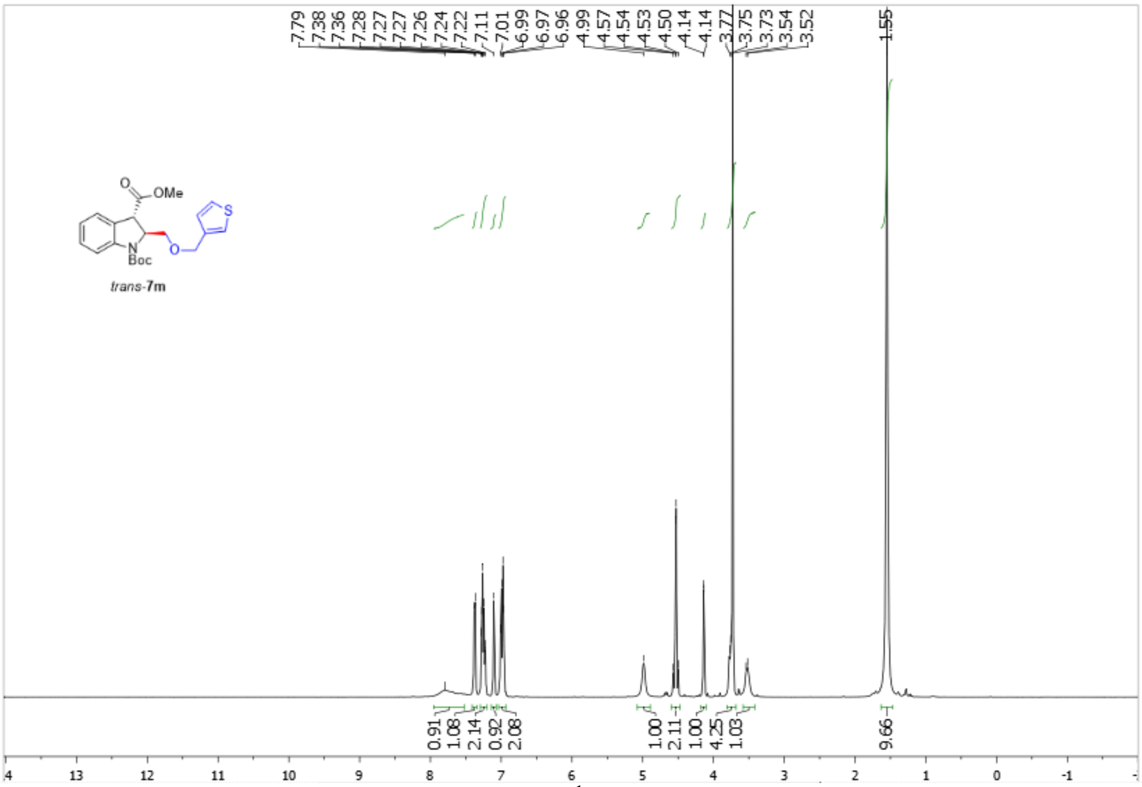

Supplementary Figure 170. ^{1}H NMR spectra of *trans*-**7m**

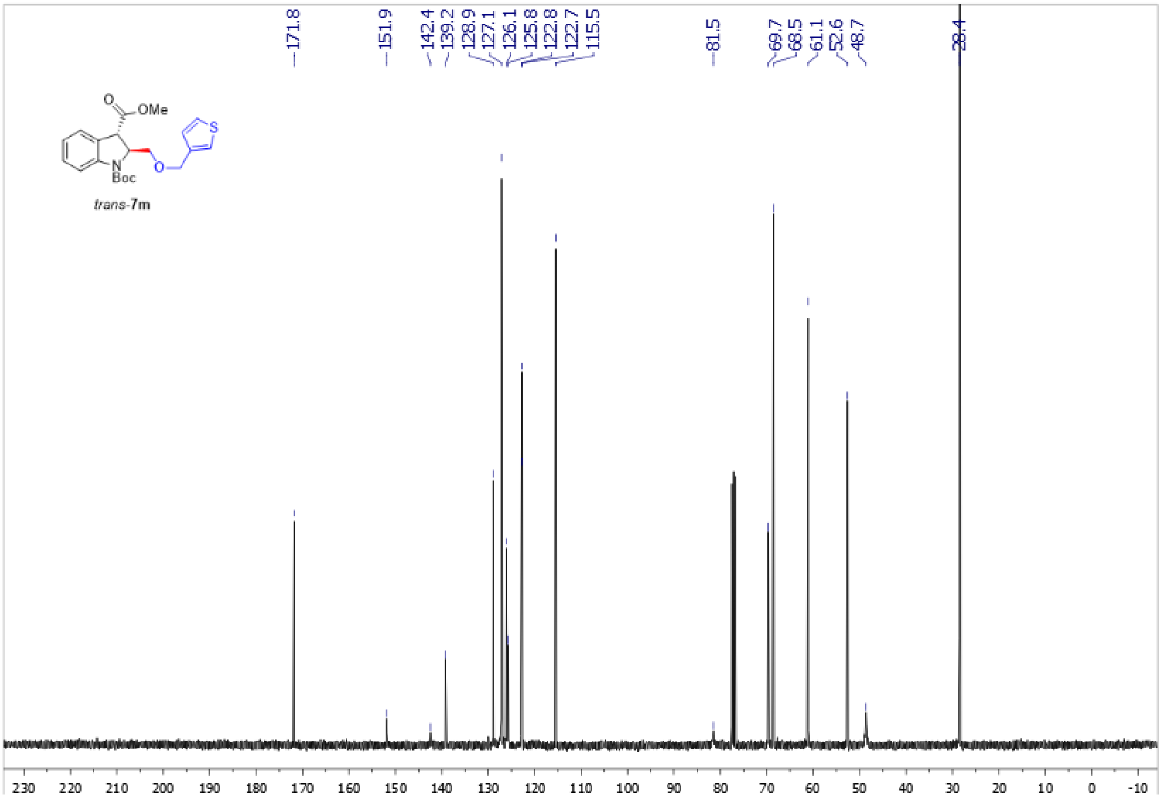

Supplementary Figure 171. ^{13}C NMR spectra of *trans*-**7m**

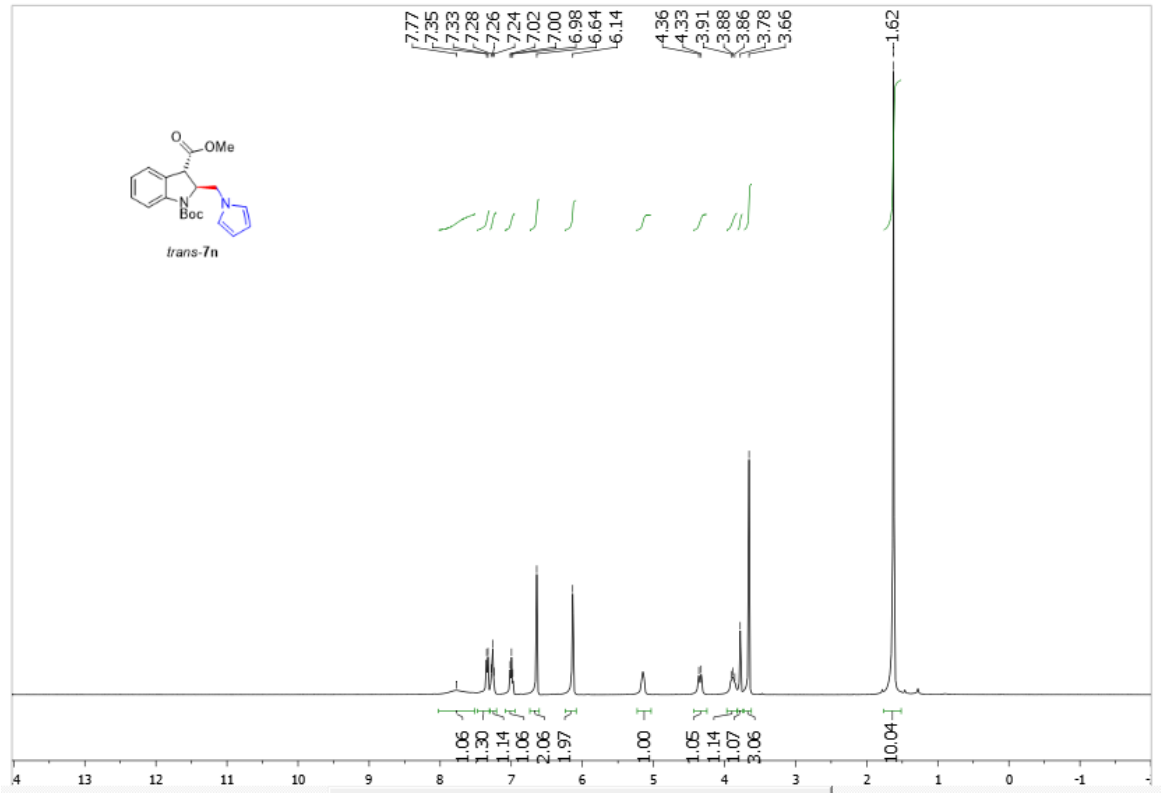

Supplementary Figure 172. ^{1}H NMR spectra of *trans*-**7n**

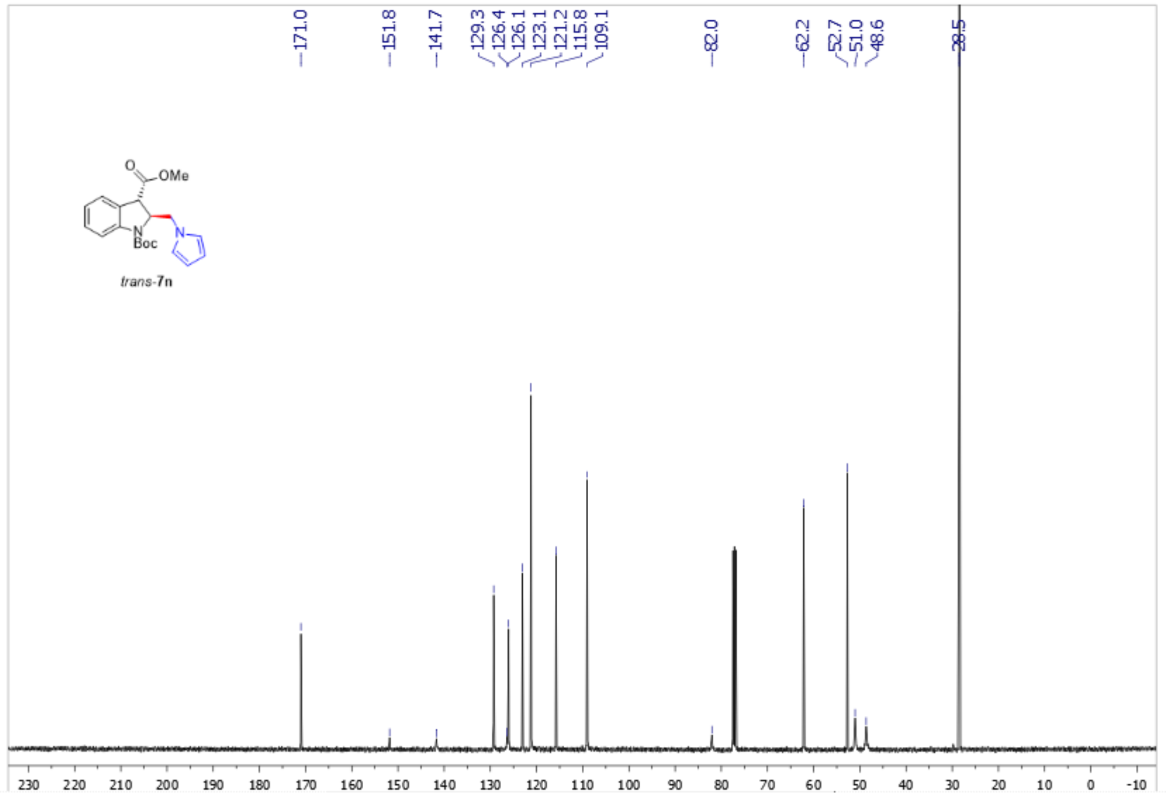

Supplementary Figure 173. ^{13}C NMR spectra of *trans*-**7n**

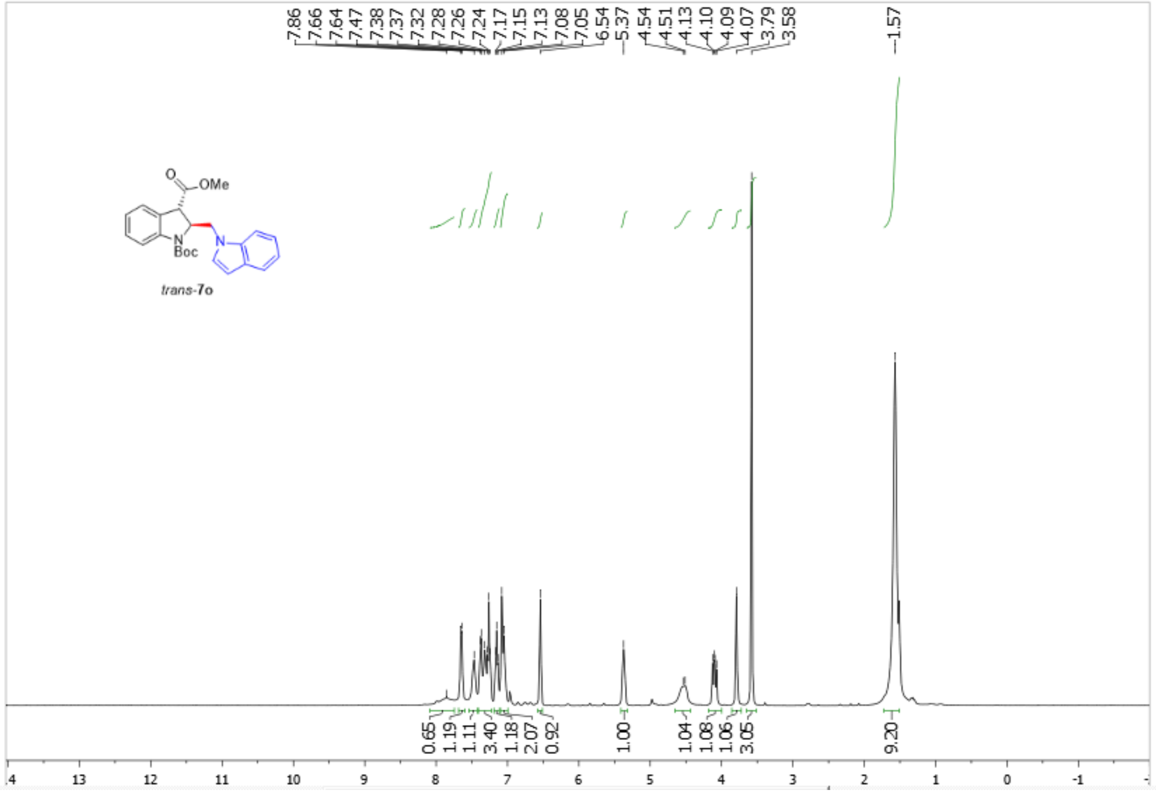

Supplementary Figure 174. ^{1}H NMR spectra of *trans*-**7o**

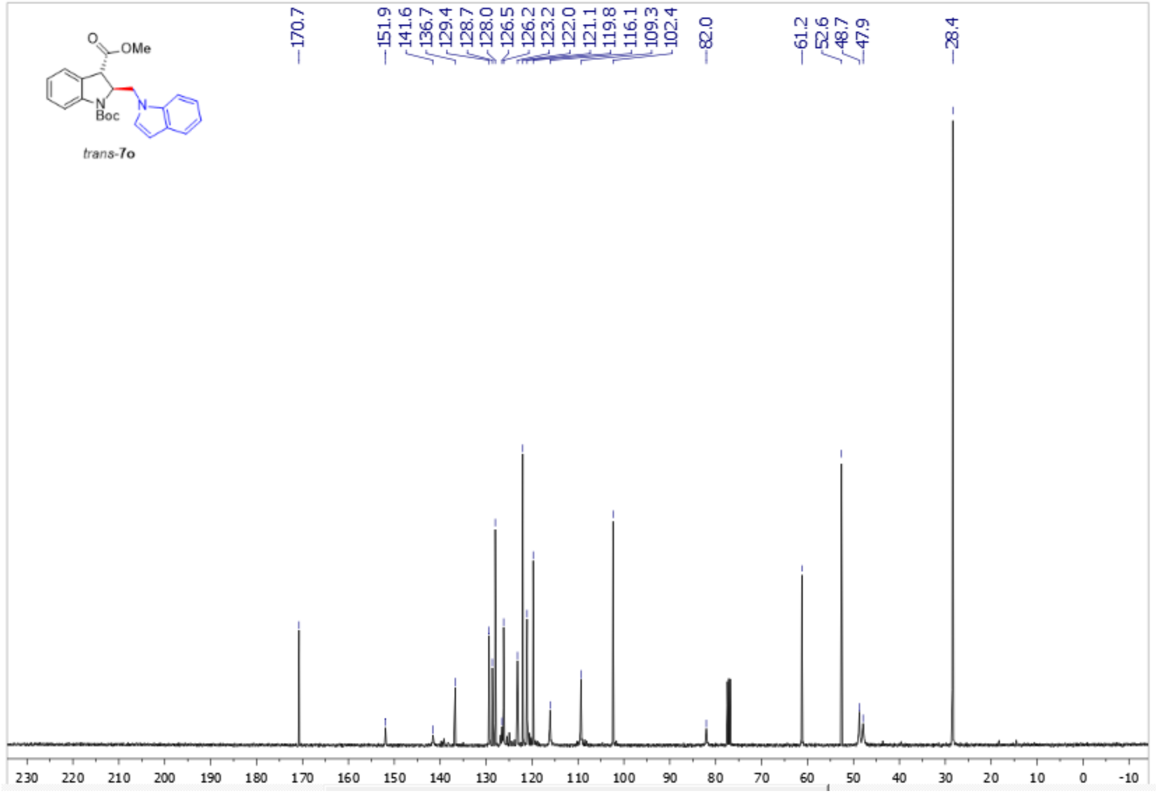

Supplementary Figure 175. ^{13}C NMR spectra of *trans*-**7o**

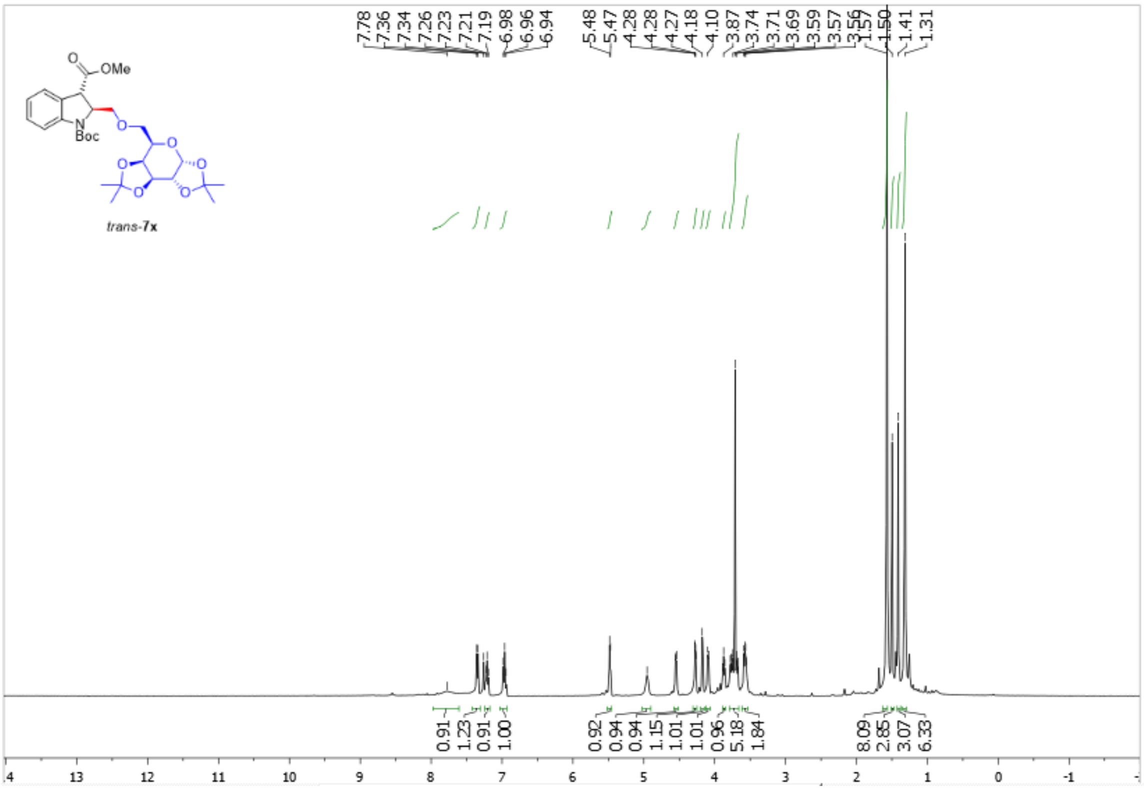

Supplementary Figure 176. ^{1}H NMR spectra of *trans*-**7x**

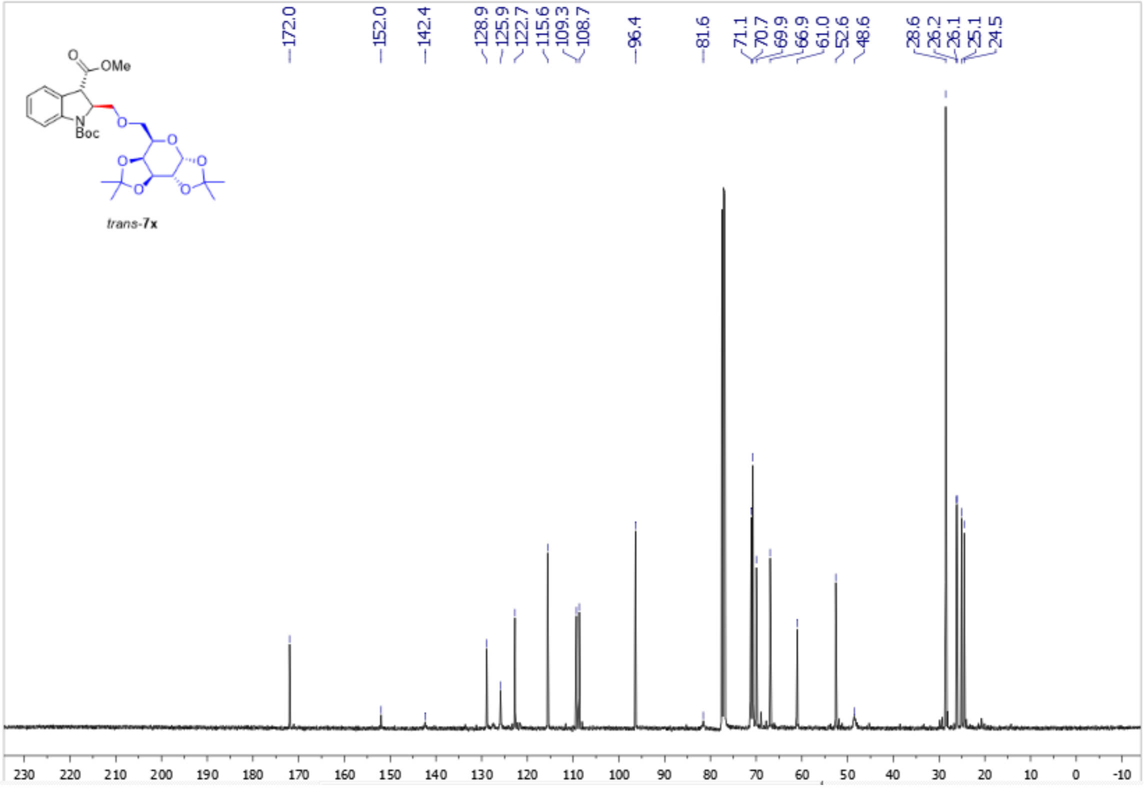

Supplementary Figure 177. ^{13}C NMR spectra of *trans*-**7x**

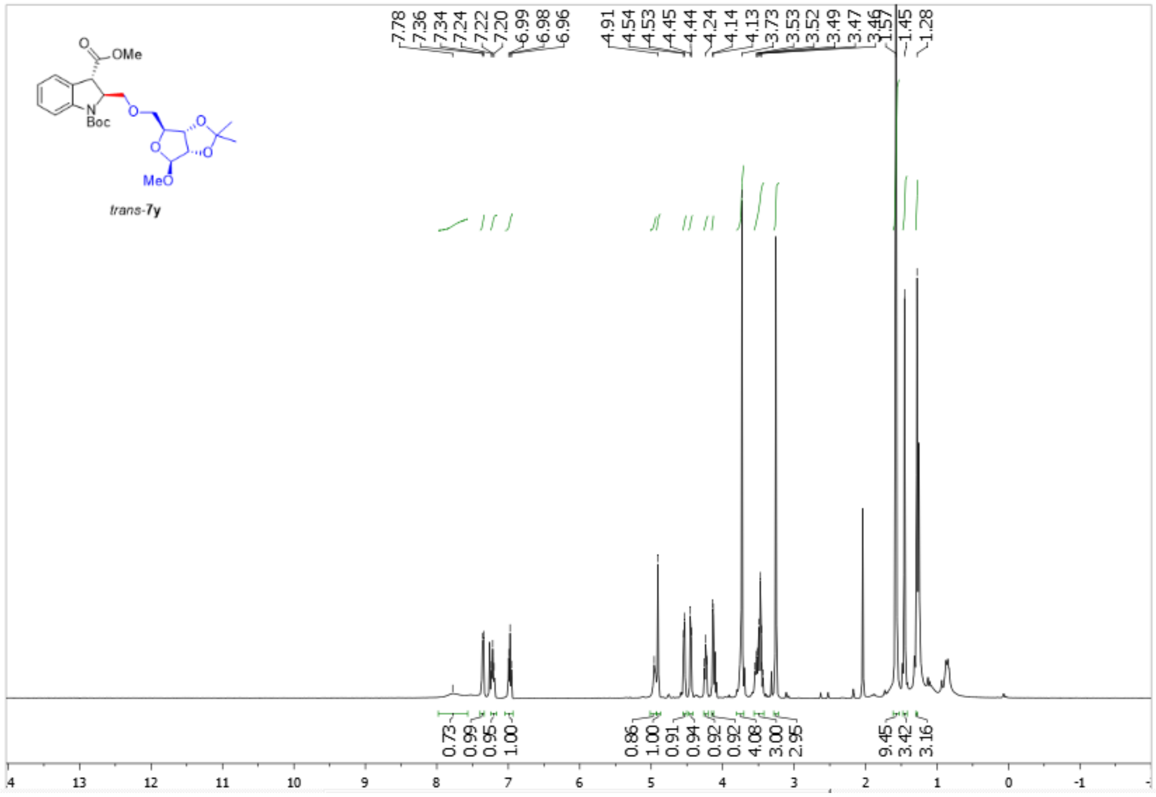

Supplementary Figure 178. ^{1}H NMR spectra of *trans*-**7y**

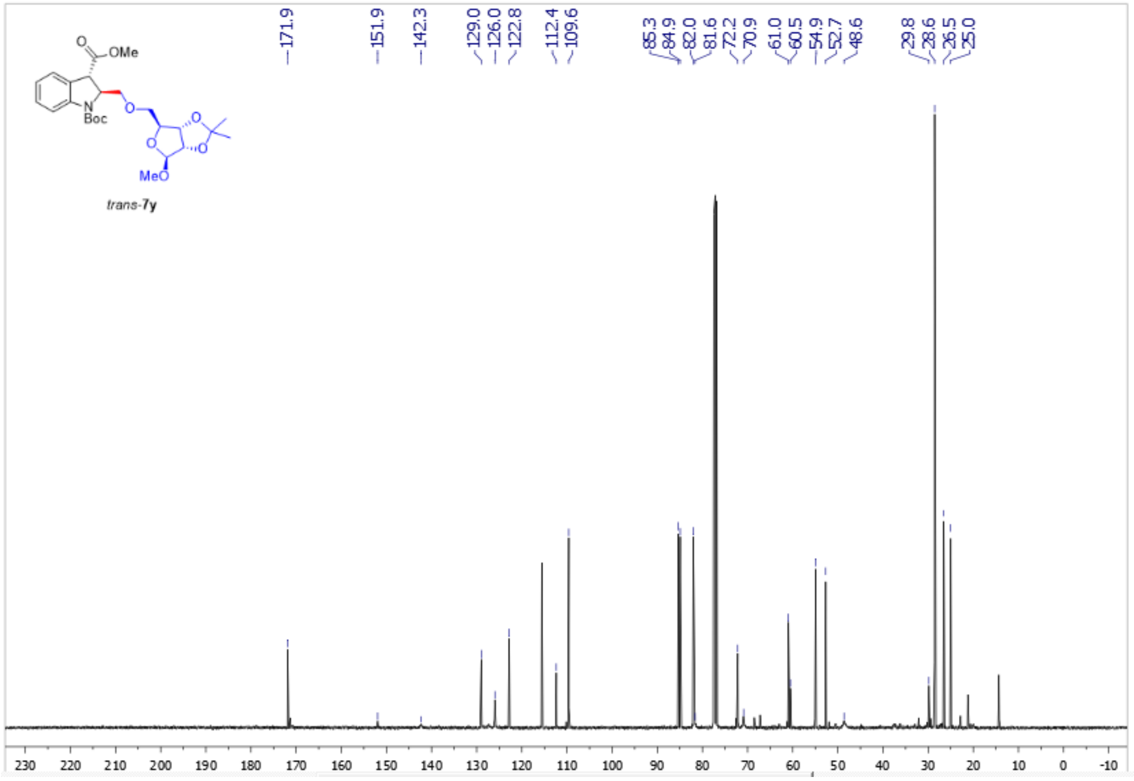

Supplementary Figure 179. ^{13}C NMR spectra of *trans*-**7y**

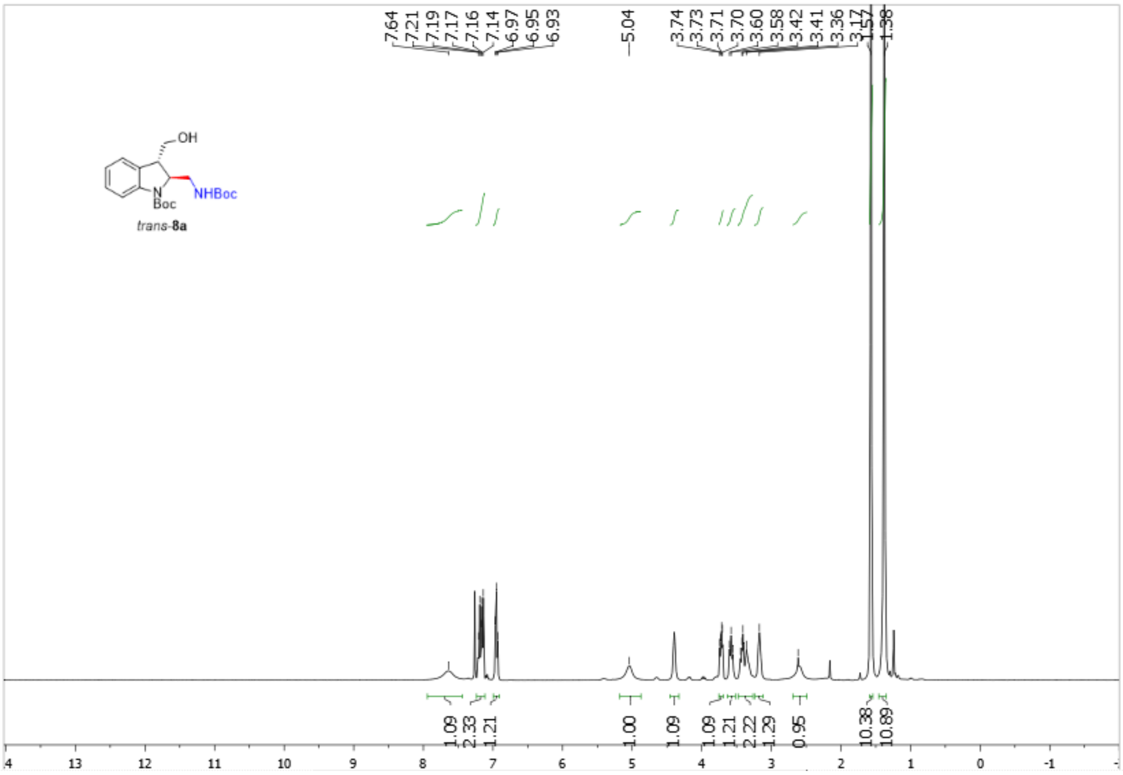

Supplementary Figure 181. ^{1}H NMR spectra of *trans*-**8a**

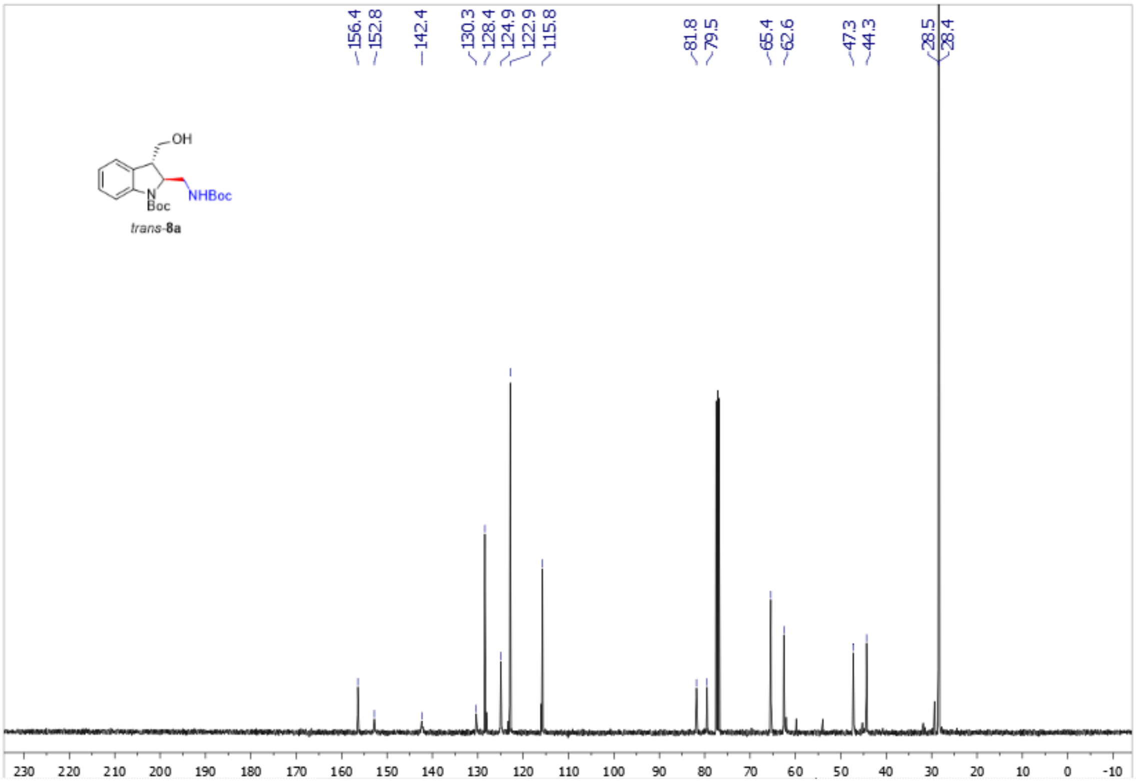

Supplementary Figure 182. ^{13}C NMR spectra of *trans*-**8a**

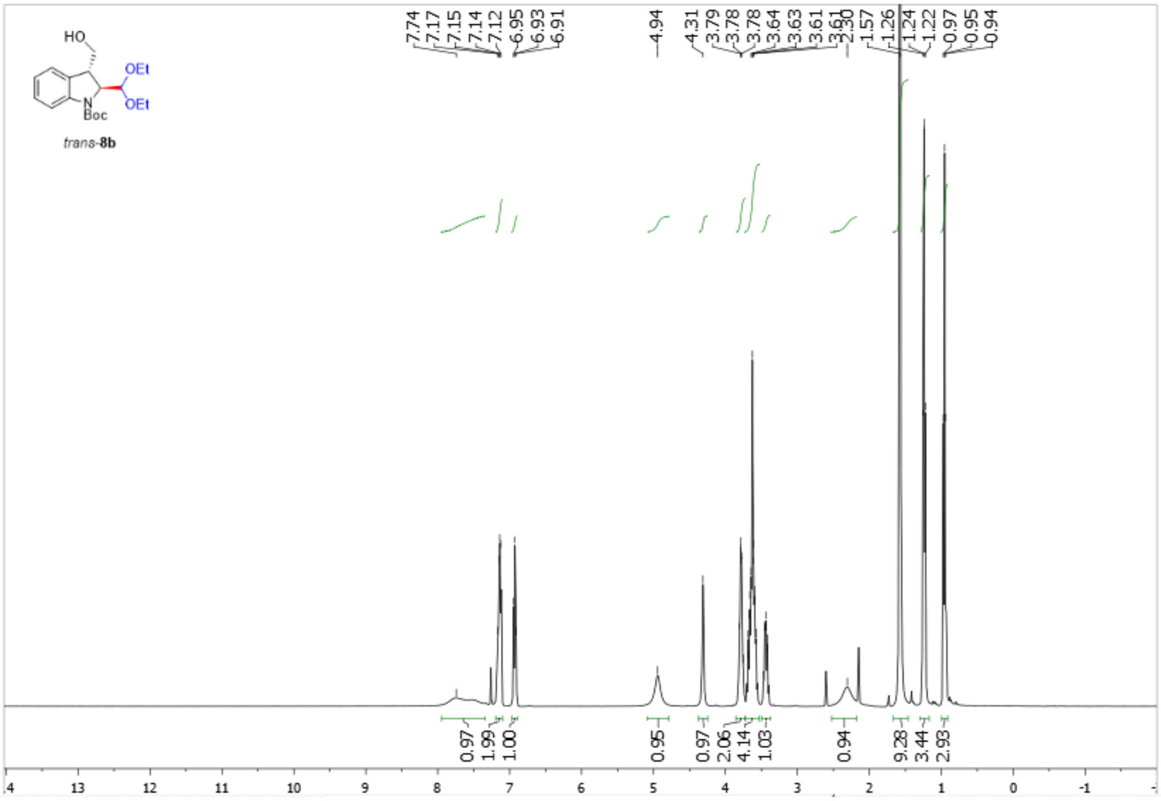

Supplementary Figure 183. ^{1}H NMR spectra of *trans*-**8b**

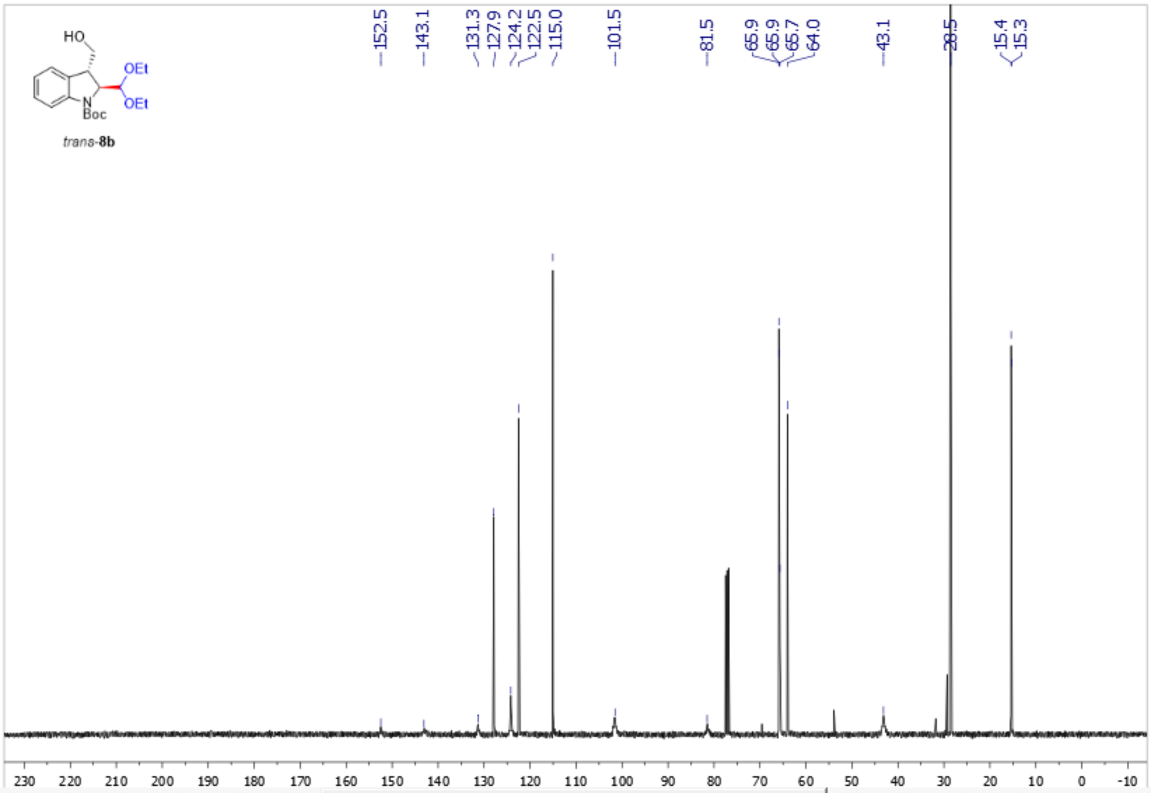

Supplementary Figure 184. ^{13}C NMR spectra of *trans*-**8b**

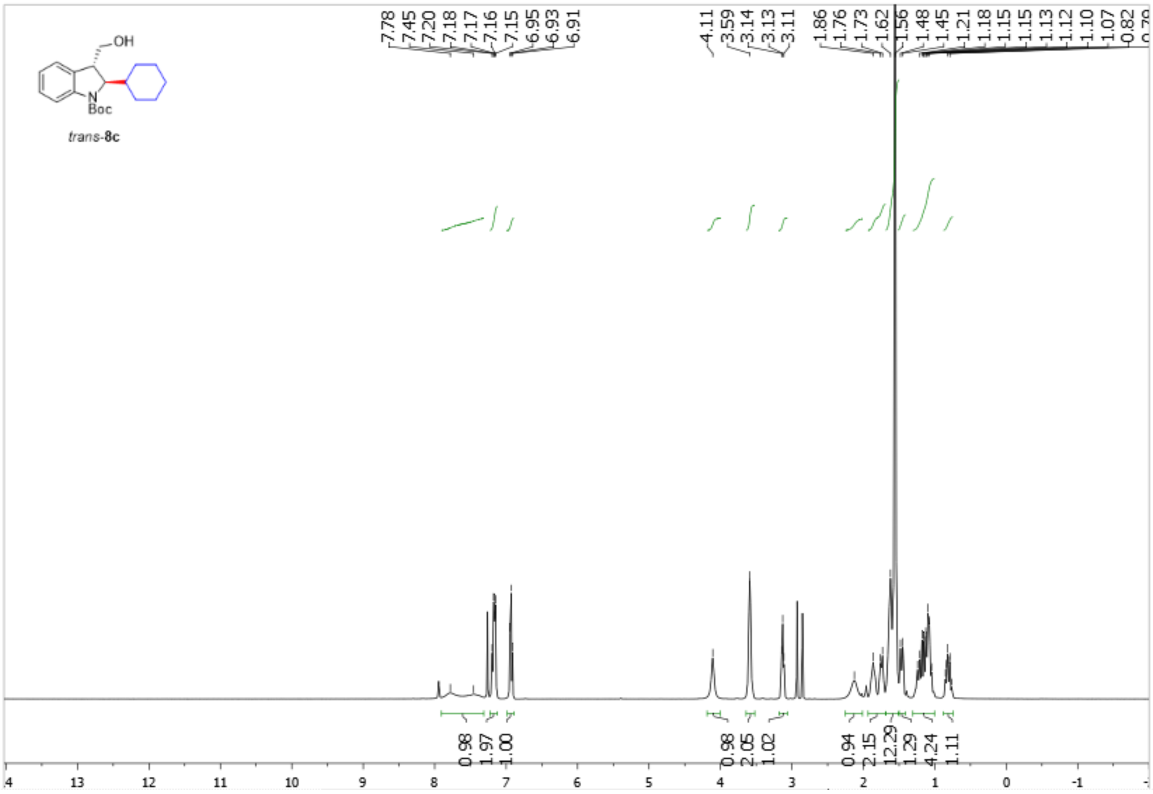

Supplementary Figure 185. ^{1}H NMR spectra of *trans*-**8c**

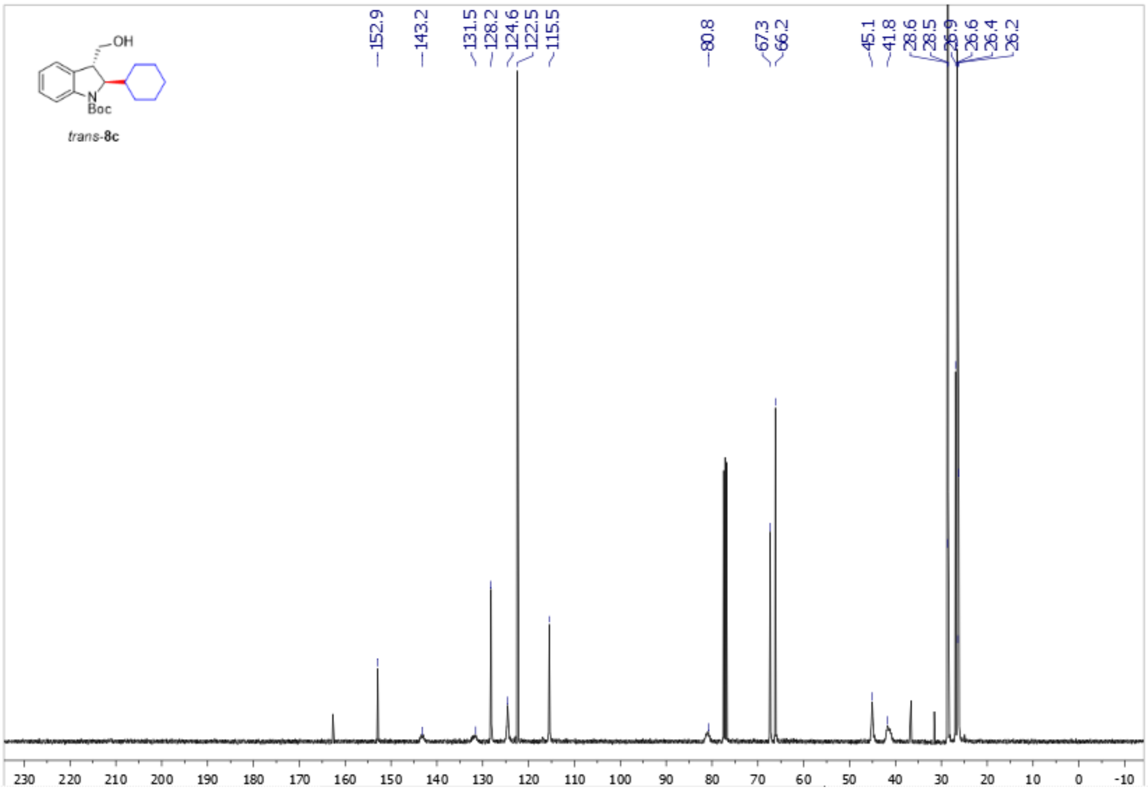

Supplementary Figure 186. ^{13}C NMR spectra of *trans*-**8c**

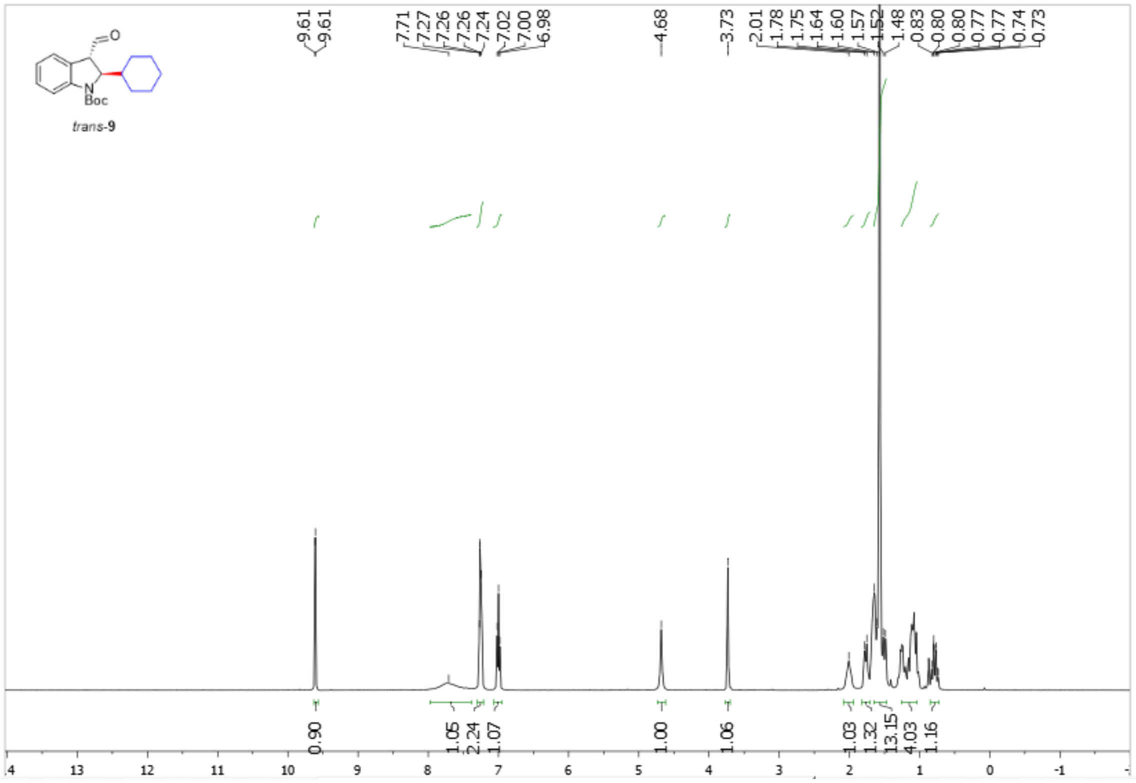

Supplementary Figure 187. ^{1}H NMR spectra of *trans*-**9**

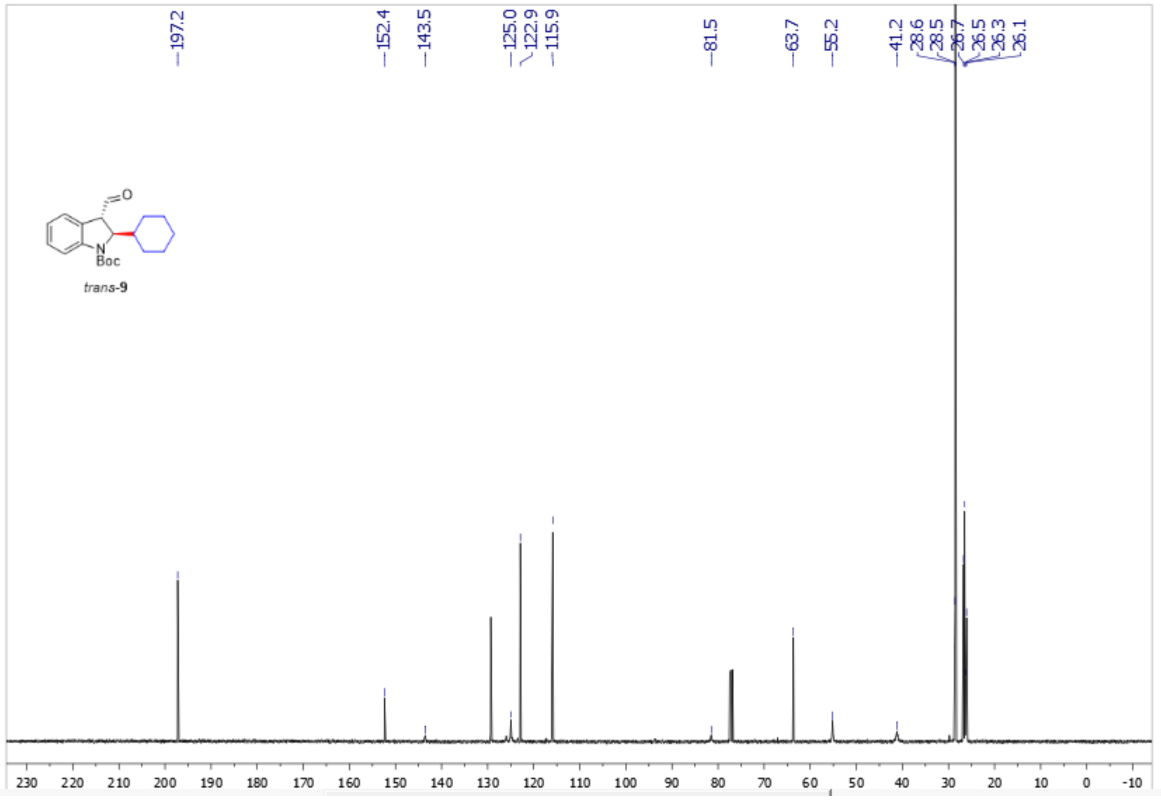

Supplementary Figure 188. ^{13}C NMR spectra of *trans*-**9**

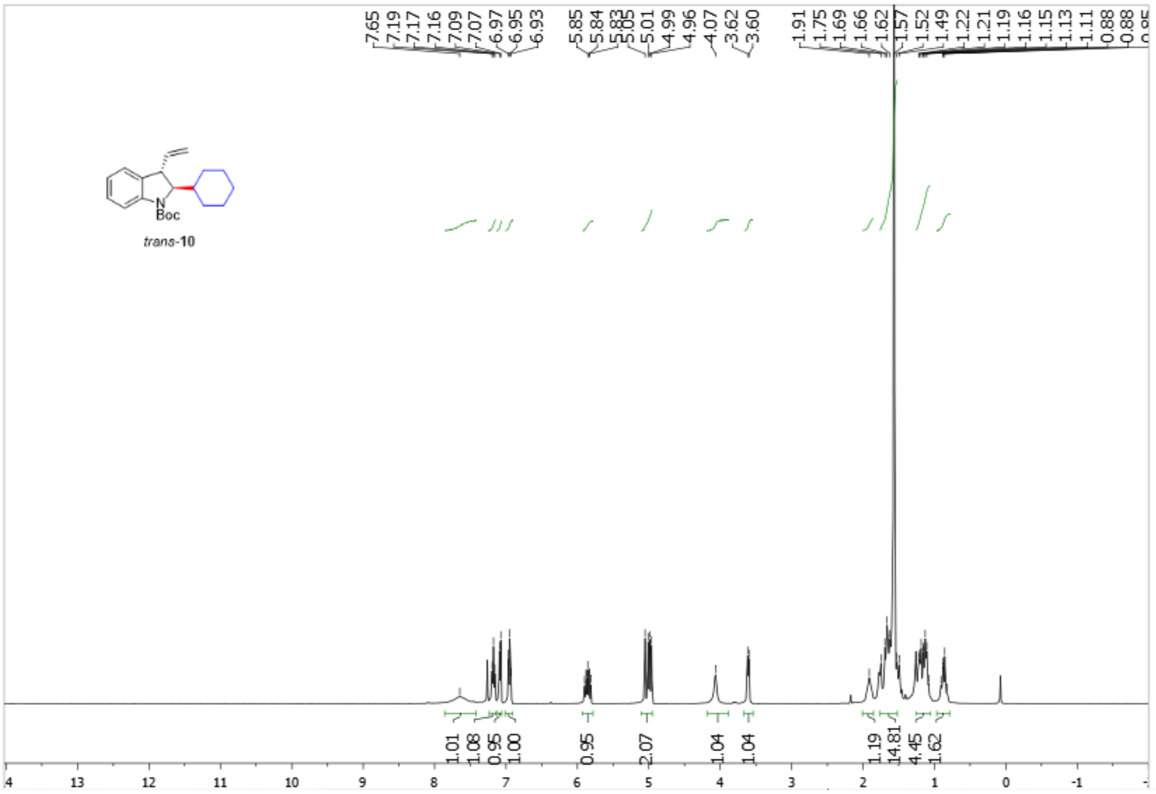

Supplementary Figure 189. ^{1}H NMR spectra of *trans*-**10**

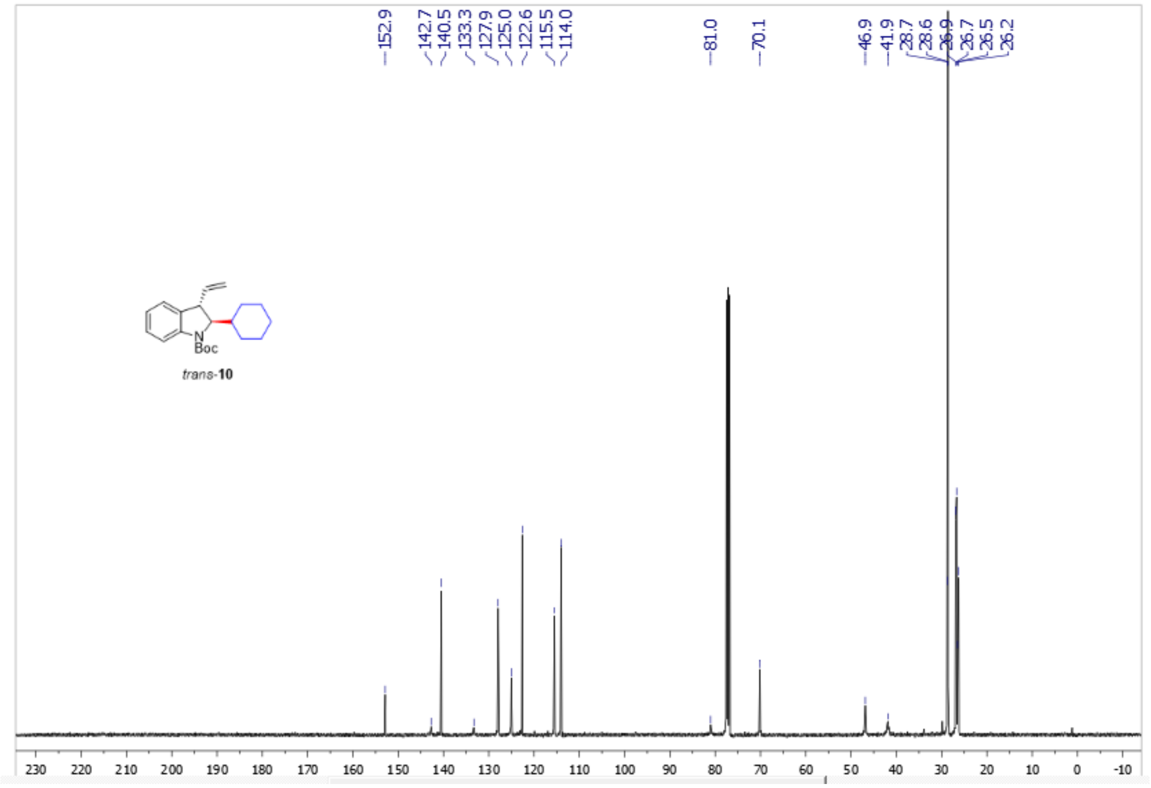

Supplementary Figure 190. ^{13}C NMR spectra of *trans*-**10**

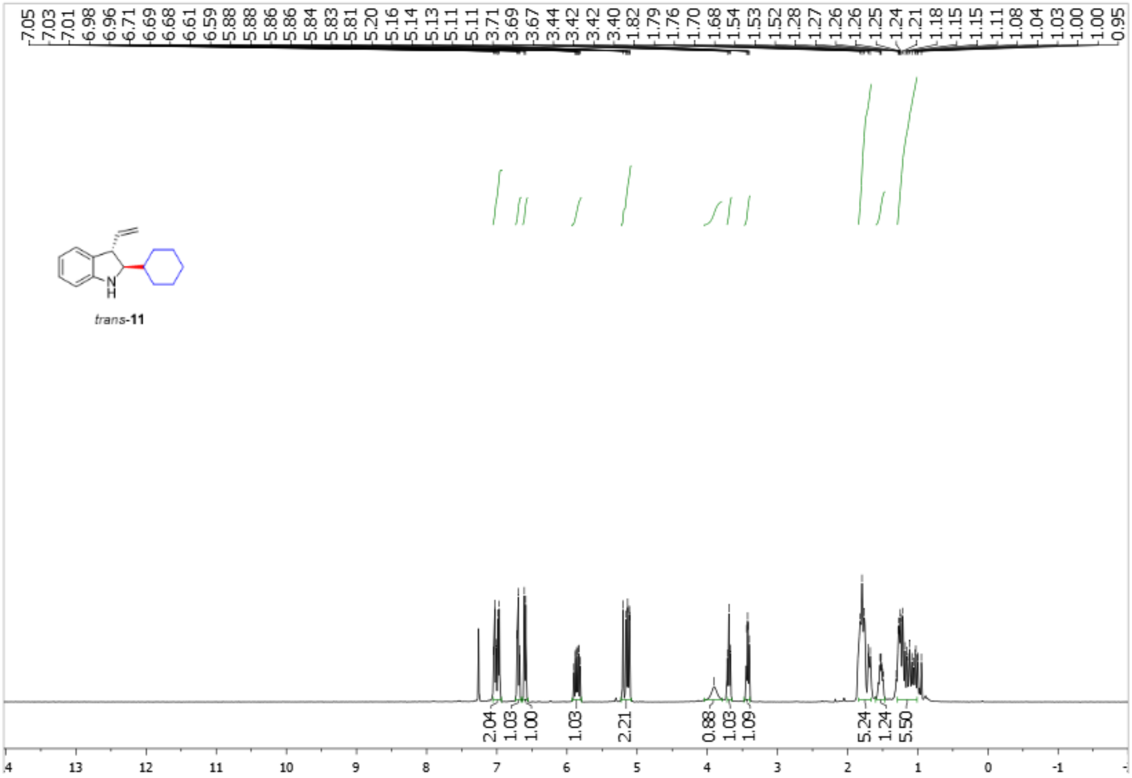

Supplementary Figure 191. ^{1}H NMR spectra of *trans*-**11**

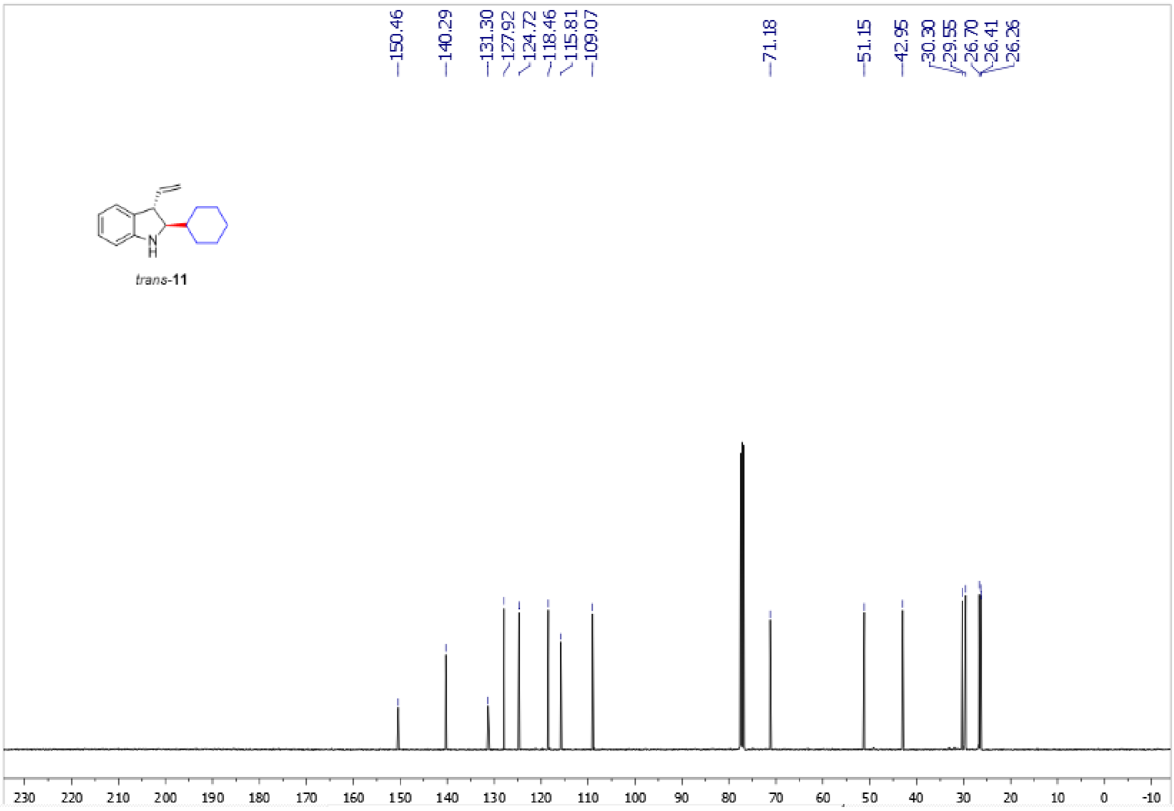

Supplementary Figure 192. ^{13}C NMR spectra of *trans*-**11**
