# Supplementary material for: Organophotocatalytic dearomatization of indoles, pyrroles and benzo(thio)furans via a Giese-type transformation
Source: Commun Chem. 2021 Feb 19;4:20. doi: 10.1038/s42004-021-00460-y (PMC9814947; doi:10.1038/s42004-021-00460-y)
Supplement: Supplementary file 5 — Supplementary Data 2 [file 42004_2021_460_MOESM5_ESM.pdf]

Supplementary Data 2: Chiral HPLC analysis spectra

<Chromatogram>

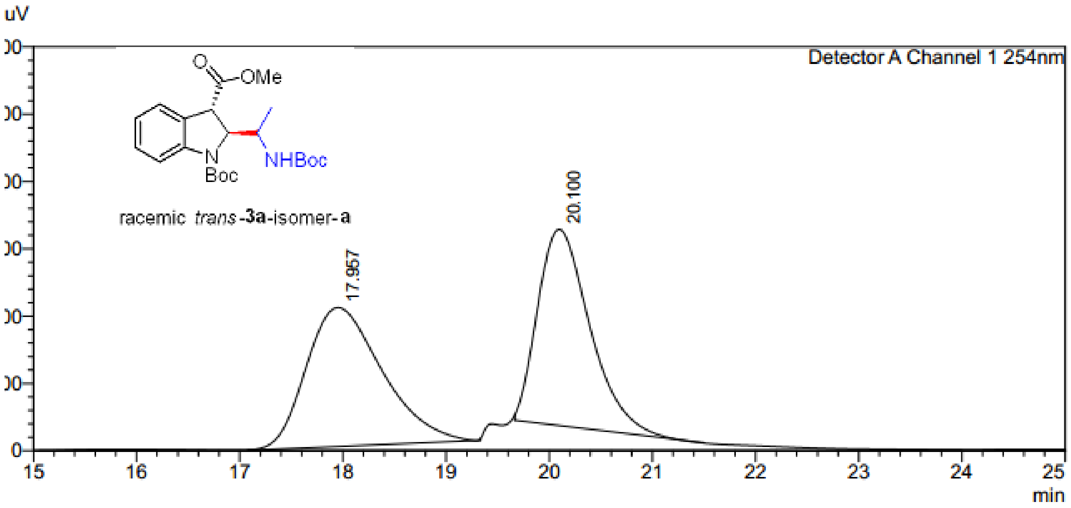

<Peak Table>

Detector A Channel 1 254nm

Peak#	Ret. Time	Area	Height	Area%
1	17.957	5287790	102789	50.767
2	20.100	5128056	143513	49.233
Total		10415846	246301	100.000

Supplementary Figure 193. HPLC spectra of racemic *trans*-**3a**-isomer-**a**

<Chromatogram>

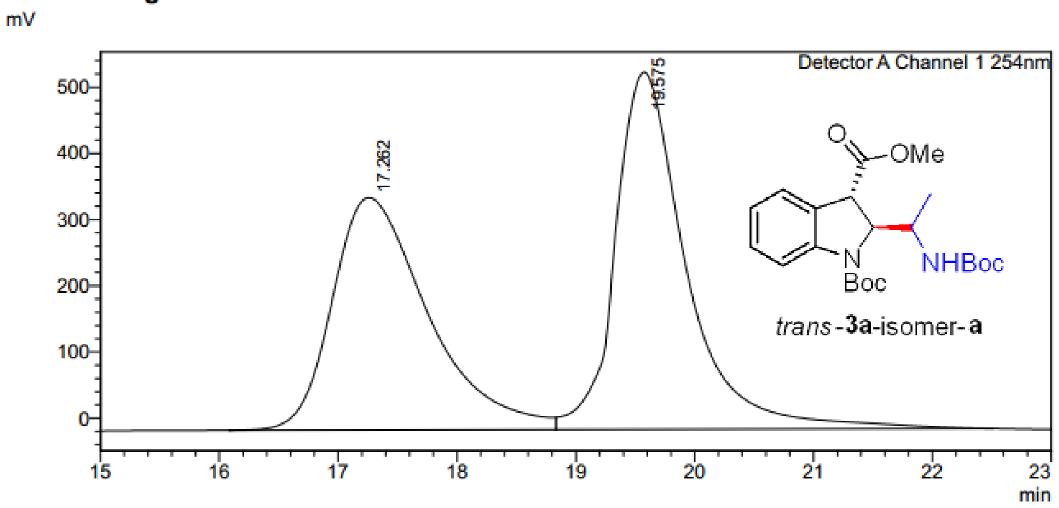

<Peak Table>

Detector A Channel 1 254nm

Peak#	Ret. Time	Area	Height	Area%
1	17.262	19695520	348440	46.834
2	19.575	22358194	532833	53.166
Total		42053713	881273	100.000

Supplementary Figure 194. HPLC spectra of *trans*-**3a**-isomer-**a**

<Chromatogram>

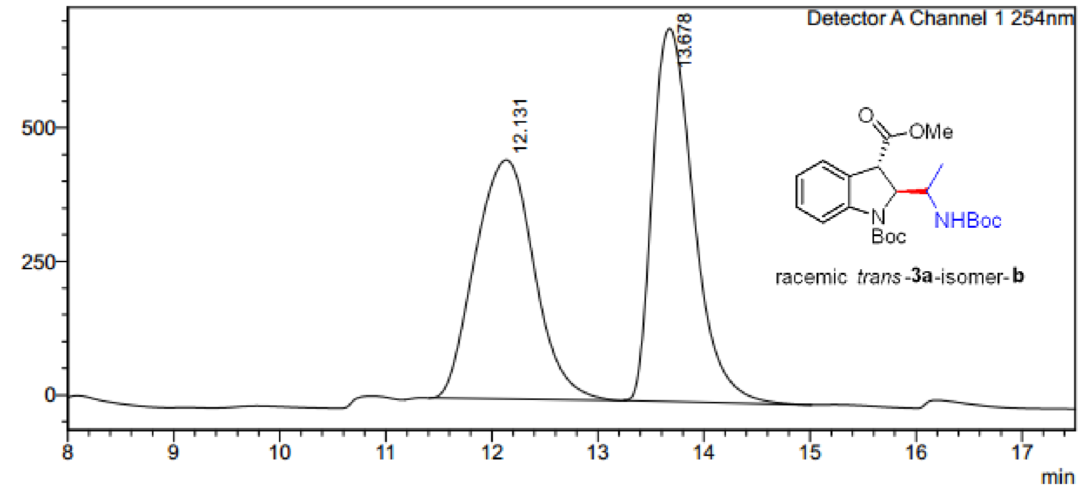

<Peak Table>

Detector A Channel 1 254nm

Peak#	Ret. Time	Area	Height	Area%
1	12.131	17406072	445944	47.959
2	13.678	18887221	681628	52.041
Total		36293293	1127571	100.000

Supplementary Figure 195. HPLC spectra of racemic *trans*-**3a**-isomer-**b**

<Chromatogram>

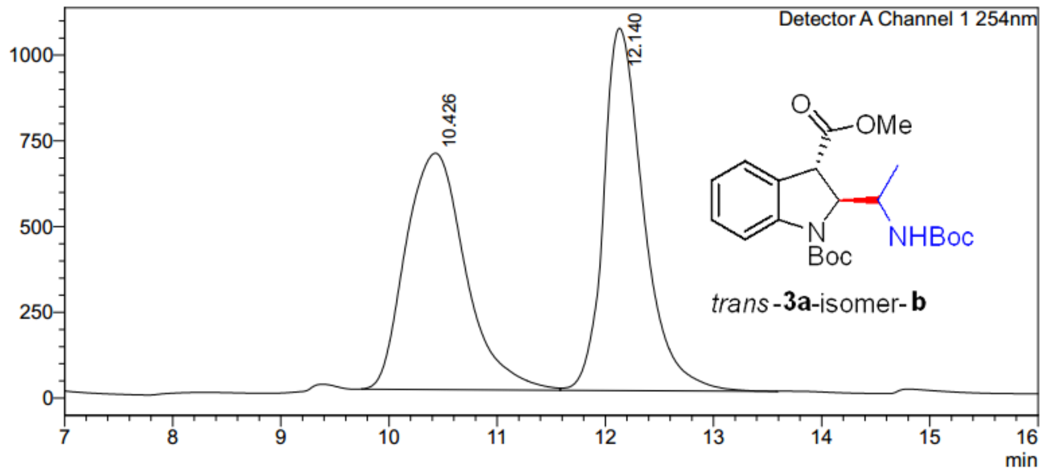

<Peak Table>

Detector A Channel 1 254nm

Peak#	Ret. Time	Area	Height	Area%
1	10.426	26548069	679539	49.320
2	12.140	27279925	1045145	50.680
Total		53827994	1724684	100.000

Supplementary Figure 196. HPLC spectra of racemic **3a**-isomer-**b**

<Chromatogram>

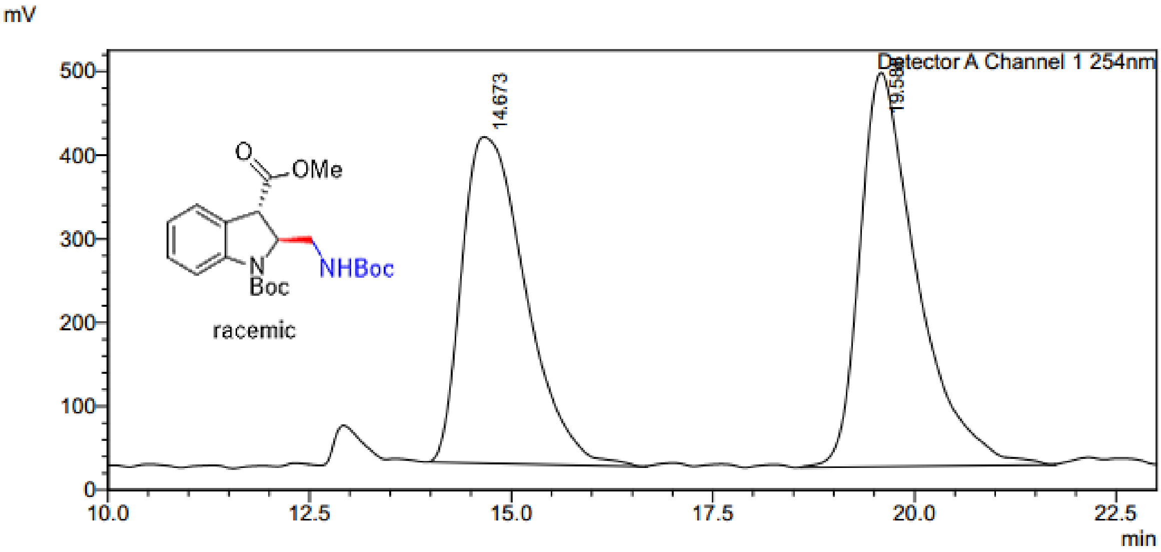

<Peak Table>

Detector A Channel 1 254nm

Peak#	Ret. Time	Area	Height	Area%
1	14.673	21488810	387521	48.055
2	19.588	23228312	463405	51.945
Total		44717122	850926	100.000

Supplementary Figure 197. HPLC spectra of racemic *trans*-**7a**

<Chromatogram>

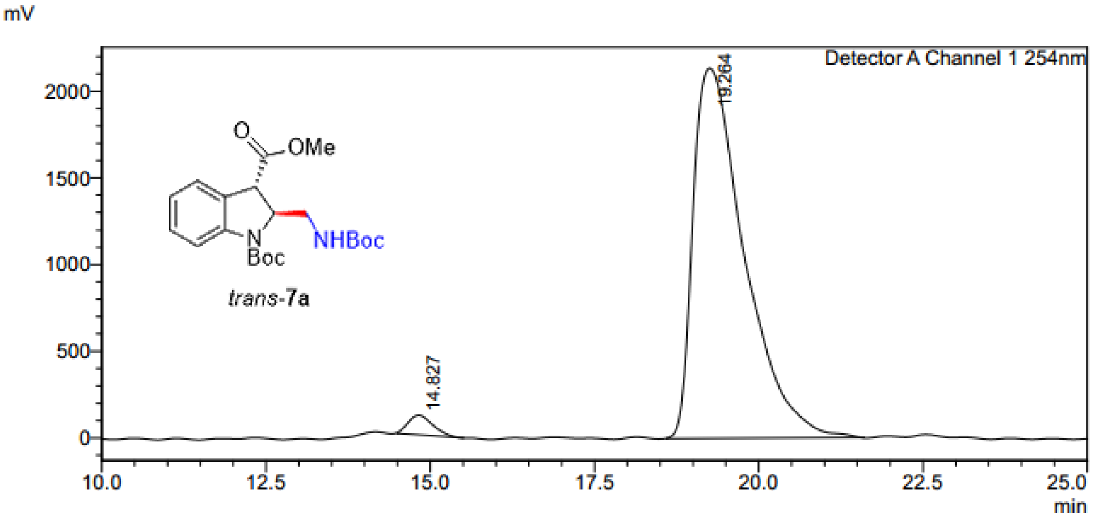

<Peak Table>

Detector A Channel 1 254nm

Peak#	Ret. Time	Area	Height	Area%
1	14.827	2928796	110173	2.463
2	19.264	116005881	2129423	97.537
Total		118934677	2239595	100.000

Supplementary Figure 198. HPLC spectra of *trans*-**7a**

<Chromatogram>

mV

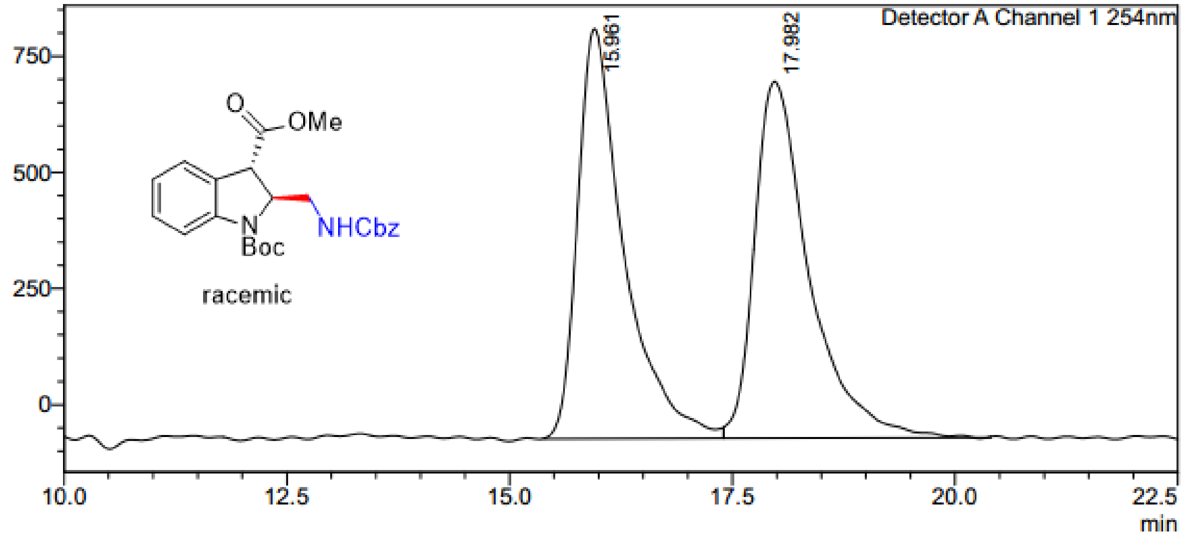

<Peak Table>

Detector A Channel 1 254nm

Peak#	Ret. Time	Area	Height	Area%
1	15.961	31946494	871941	49.303
2	17.982	32849795	759948	50.697
Total		64796290	1631889	100.000

Supplementary Figure 199. HPLC spectra of racemic *trans*-**7b**

<Chromatogram>

mV

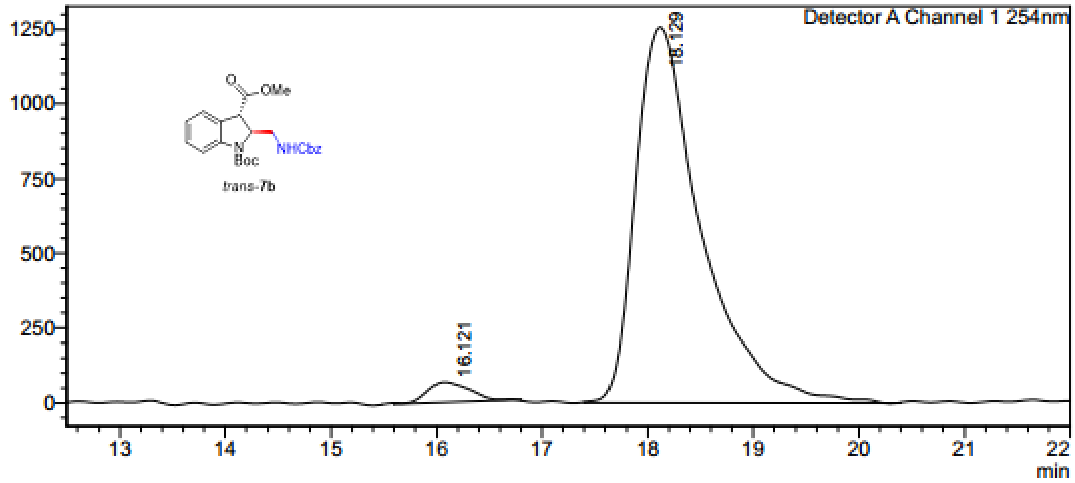

<Peak Table>

Detector A Channel 1 254nm

Peak#	Ret. Time	Area	Height	Area%
1	16.121	1865735	63533	3.304
2	18.129	54600773	1224043	96.696
Total		56466508	1287575	100.000

Supplementary Figure 200. HPLC spectra of *trans*-**7b**

<Chromatogram>

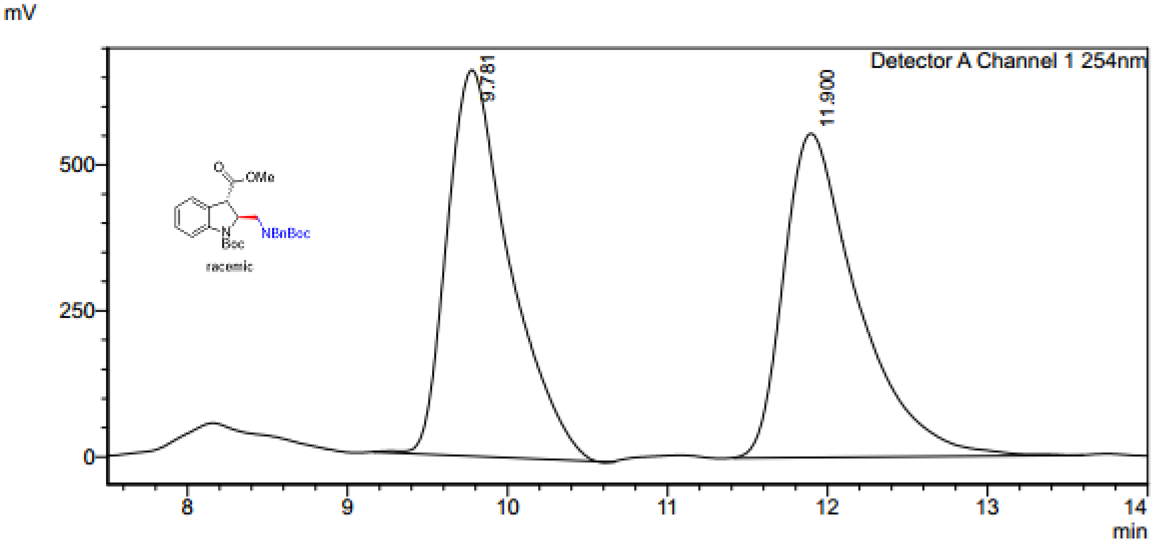

<Peak Table>

Detector A Channel 1 254nm

Peak#	Ret. Time	Area	Height	Area%
1	9.781	18038085	649522	50.166
2	11.900	17918945	547275	49.834
Total		35957030	1196797	100.000

Supplementary Figure 201. HPLC spectra of racemic *trans*-**7c**

<Chromatogram>

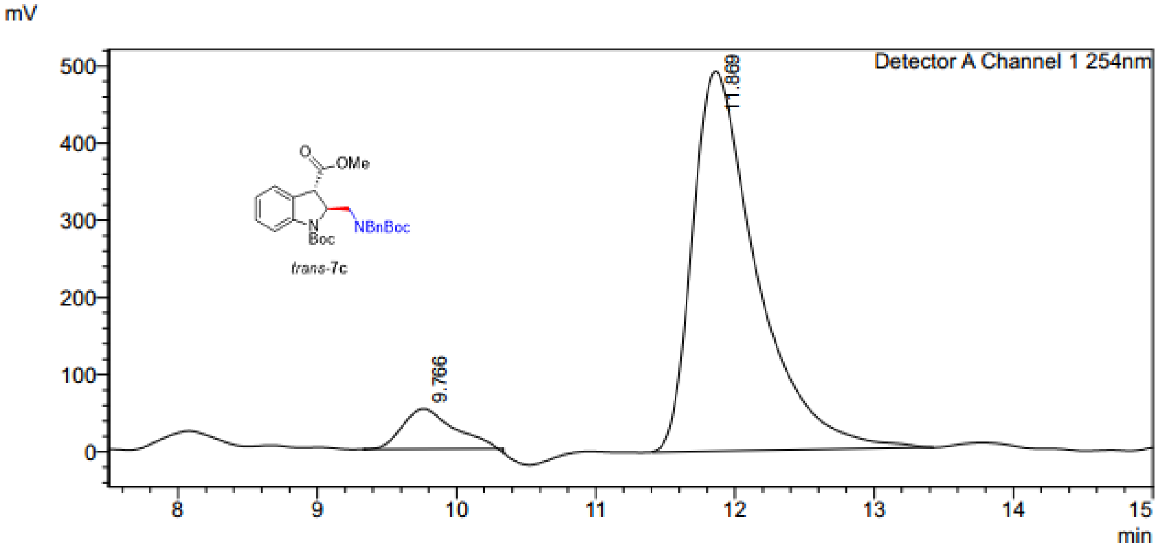

<Peak Table>

Detector A Channel 1 254nm

Peak#	Ret. Time	Area	Height	Area%
1	9.766	1348248	50283	7.857
2	11.869	15812498	486368	92.143
Total		17160746	536652	100.000

Supplementary Figure 202. HPLC spectra of *trans*-**7c**

<Chromatogram>

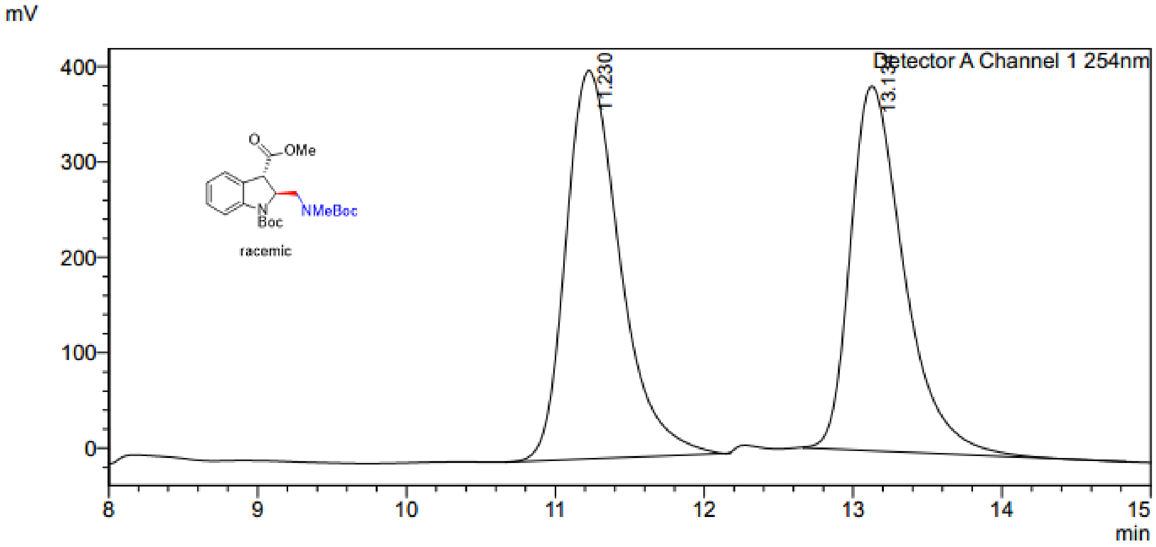

<Peak Table>

Detector A Channel 1 254nm

Peak#	Ret. Time	Area	Height	Area%
1	11.230	10315193	401110	51.952
2	13.134	9540075	377865	48.048
Total		19855268	778975	100.000

Supplementary Figure 203. HPLC spectra of racemic *trans*-**7d**

<Chromatogram>

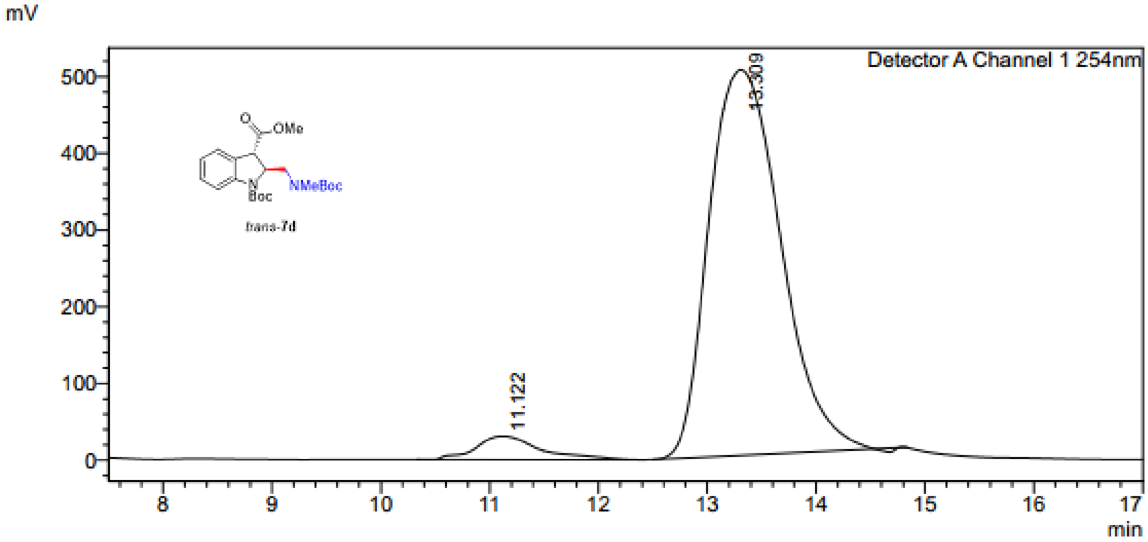

<Peak Table>

Detector A Channel 1 254nm

Peak#	Ret. Time	Area	Height	Area%
1	11.122	1275421	30357	5.229
2	13.309	23114092	501035	94.771
Total		24389513	531392	100.000

Supplementary Figure 204. HPLC spectra of *trans*-**7d**

<Chromatogram>

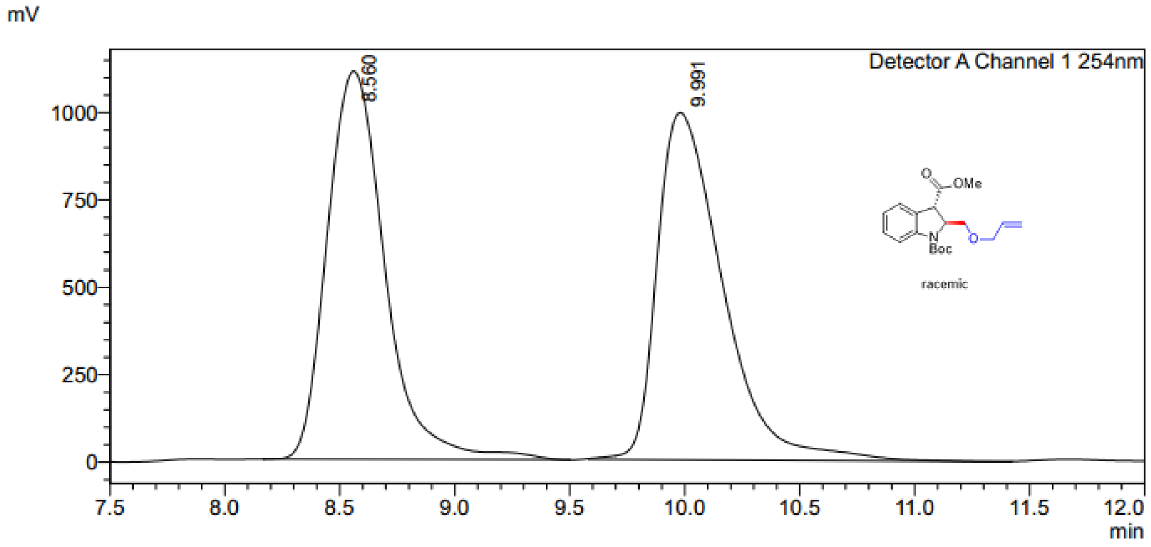

<Peak Table>

Detector A Channel 1 254nm

Peak#	Ret. Time	Area	Height	Area%
1	8.560	19407729	1077762	49.590
2	9.991	19728560	959907	50.410
Total		39136289	2037669	100.000

Supplementary Figure 205. HPLC spectra of racemic *trans*-**7e**

<Chromatogram>

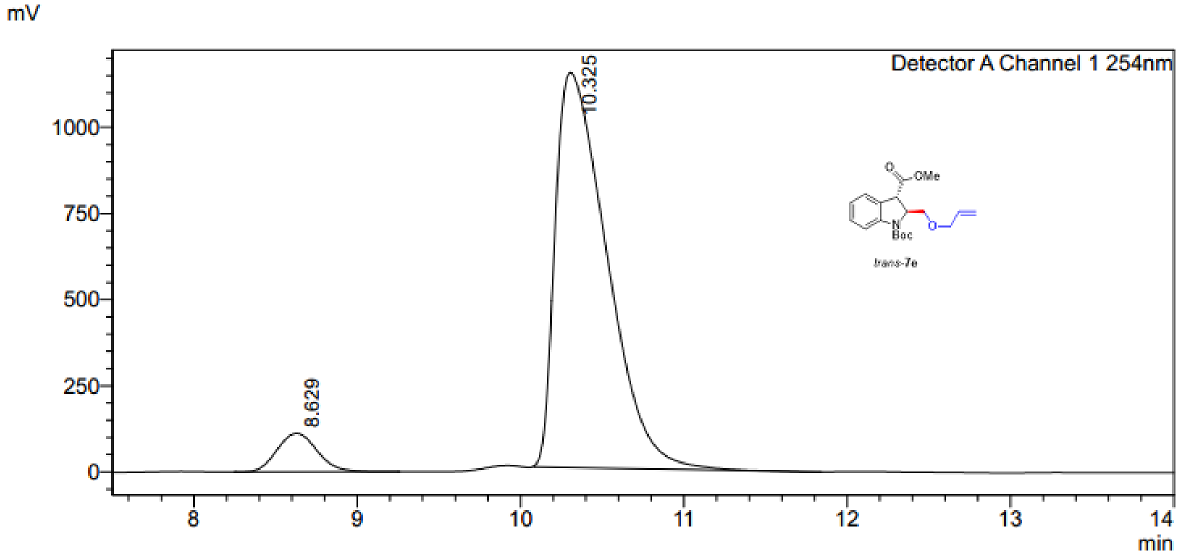

<Peak Table>

Detector A Channel 1 254nm

Peak#	Ret. Time	Area	Height	Area%
1	8.629	1905194	109075	7.027
2	10.325	25207613	1123285	92.973
Total		27112807	1232360	100.000

Supplementary Figure 206. HPLC spectra of *trans*-**7e**

<Chromatogram>

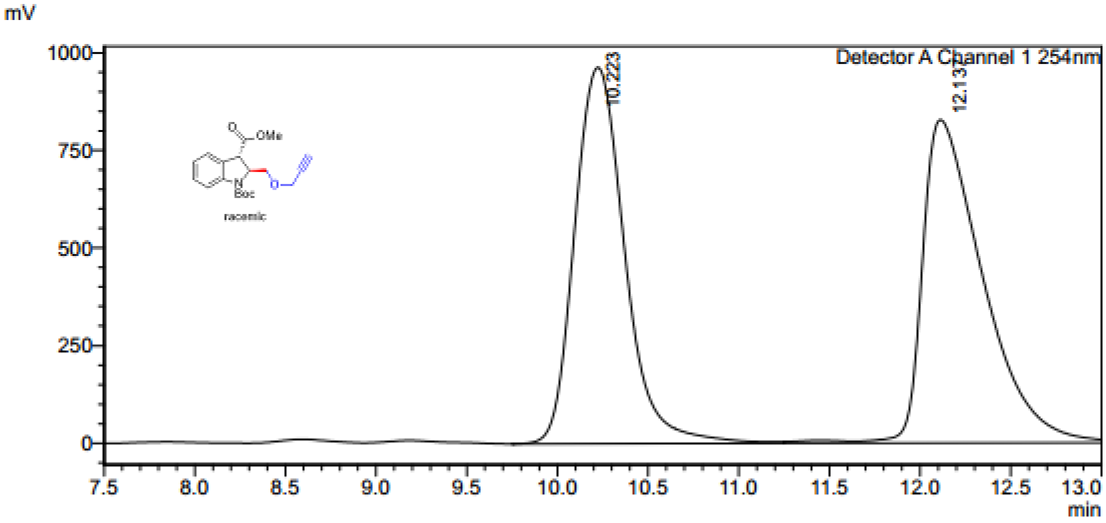

<Peak Table>

Detector A Channel 1 254nm

Peak#	Ret. Time	Area	Height	Area%
1	10.223	18571451	944779	48.982
2	12.137	19343206	812563	51.018
Total		37914657	1757342	100.000

Supplementary Figure 207. HPLC spectra of racemic *trans*-**7f**

<Chromatogram>

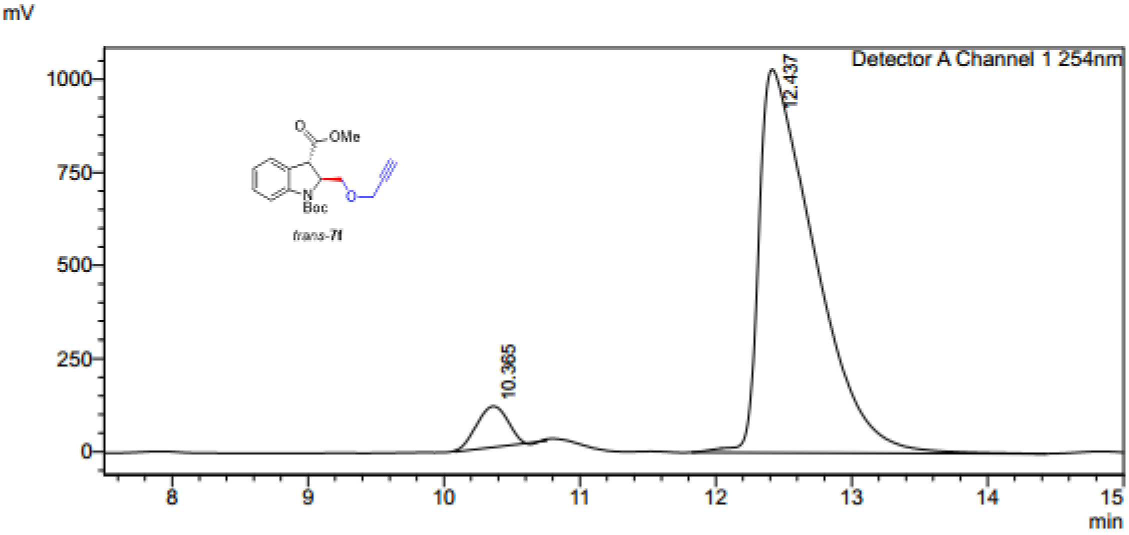

<Peak Table>

Detector A Channel 1 254nm

Peak#	Ret. Time	Area	Height	Area%
1	10.365	1772466	106857	5.707
2	12.437	29287036	988206	94.293
Total		31059503	1095064	100.000

Supplementary Figure 208. HPLC spectra of *trans*-**7f**

<Chromatogram>

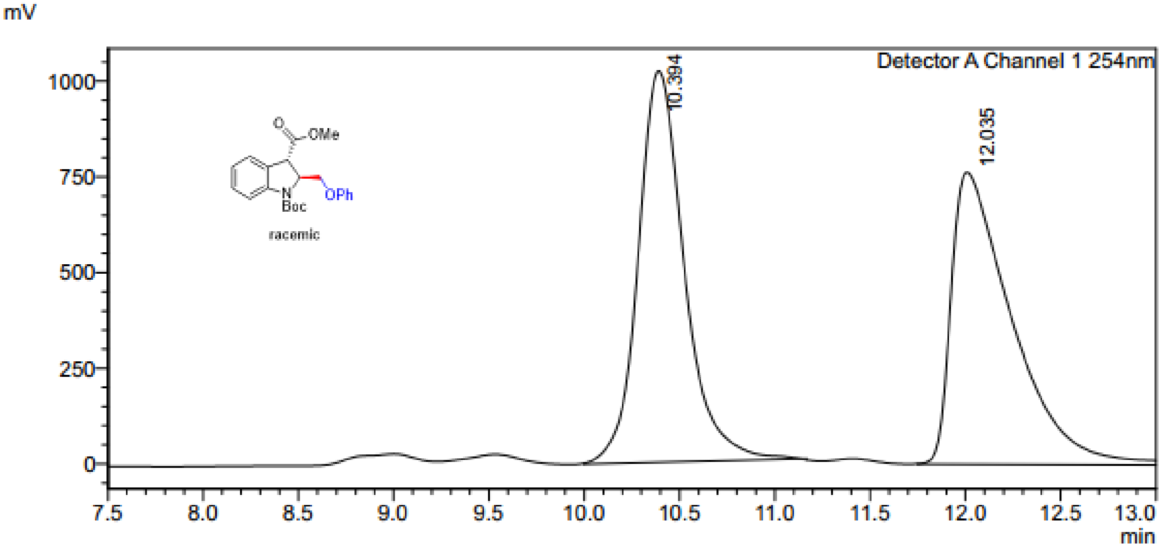

<Peak Table>

Detector A Channel 1 254nm

Peak#	Ret. Time	Area	Height	Area%
1	10.394	17197238	988269	50.225
2	12.035	17043152	729055	49.775
Total		34240390	1717324	100.000

Supplementary Figure 209. HPLC spectra of racemic *trans*-**7g**

<Chromatogram>

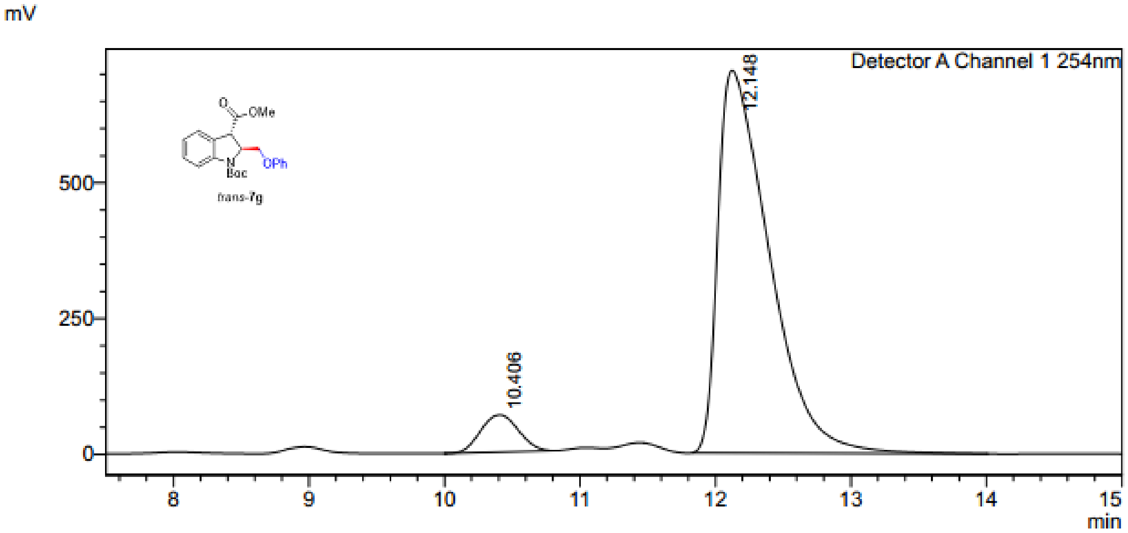

<Peak Table>

Detector A Channel 1 254nm

Peak#	Ret. Time	Area	Height	Area%
1	10.406	1328469	66161	6.666
2	12.148	18600313	696522	93.334
Total		19928782	762683	100.000

Supplementary Figure 210. HPLC spectra of *trans*-**7g**

<Chromatogram>

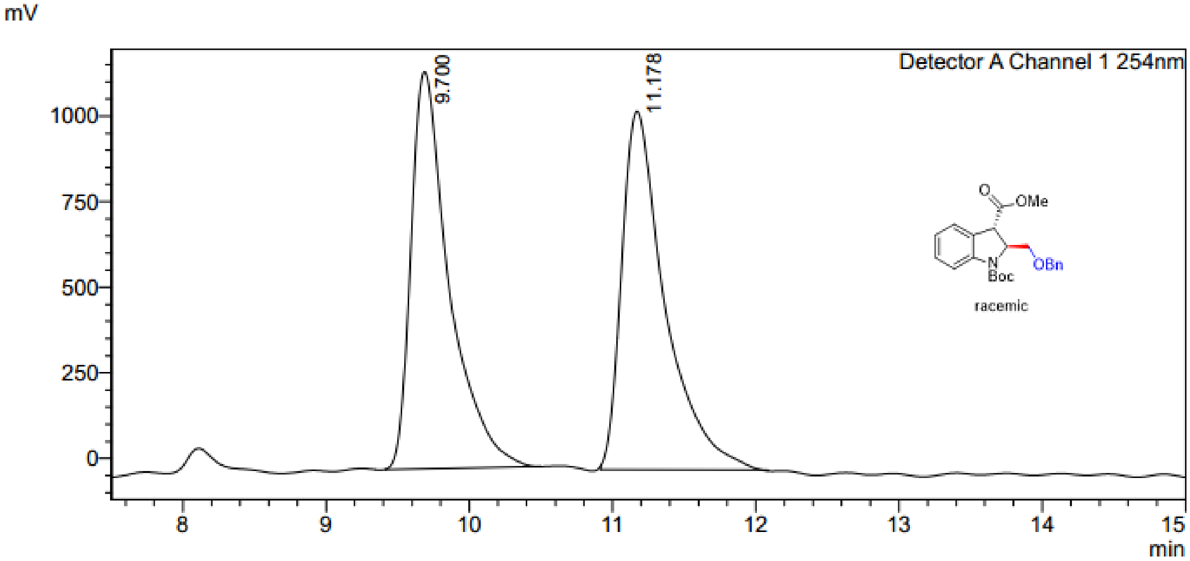

<Peak Table>

Detector A Channel 1 254nm

Peak#	Ret. Time	Area	Height	Area%
1	9.700	21565307	1107972	49.465
2	11.178	22031937	978133	50.535
Total		43597244	2086105	100.000

Supplementary Figure 211. HPLC spectra of racemic *trans*-**7h**

<Chromatogram>

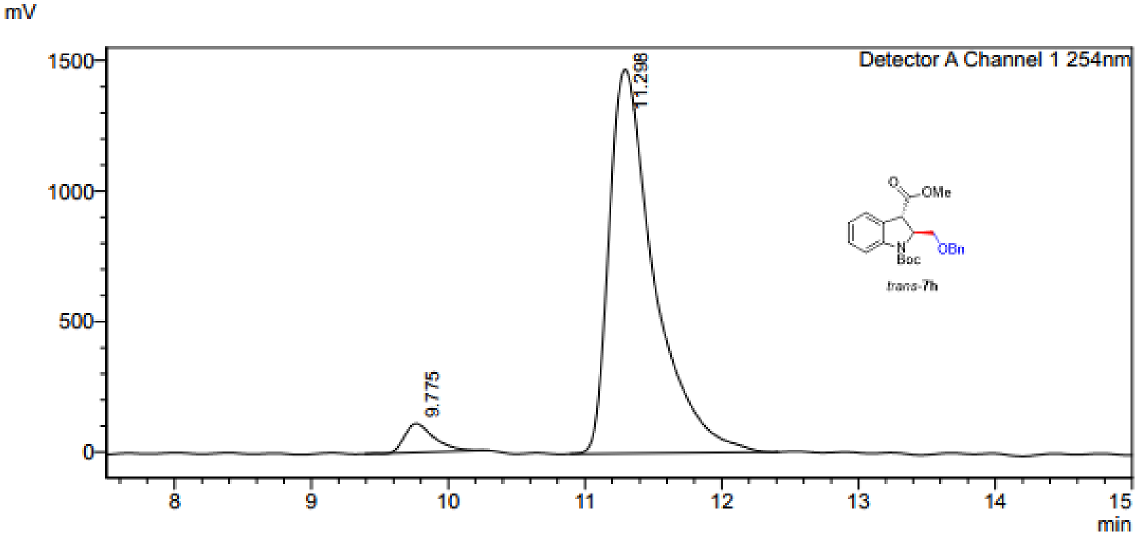

<Peak Table>

Detector A Channel 1 254nm

Peak#	Ret. Time	Area	Height	Area%
1	9.775	1686730	104177	4.764
2	11.298	33718380	1364912	95.236
Total		35405110	1469088	100.000

Supplementary Figure 212. HPLC spectra of *trans*-**7h**

<Chromatogram>

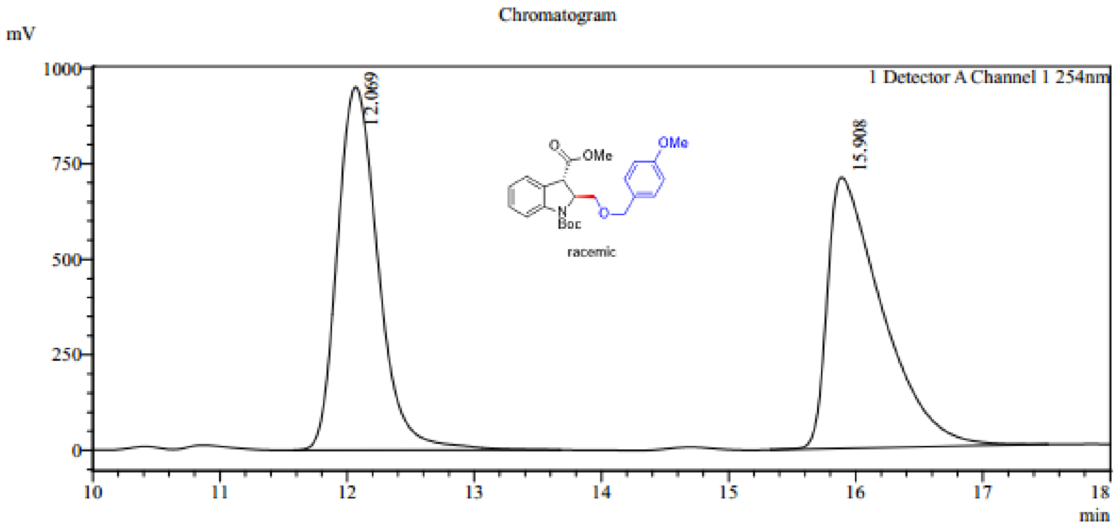

<Peak Table>

Detector A Channel 1 254nm

Peak#	Ret. Time	Area	Height	Area%
1	12.069	21255740	928156	49.906
2	15.908	21336176	697915	50.094
Total		42591916	1626070	100.000

Supplementary Figure 213. HPLC spectra of racemic *trans*-**7i**

<Chromatogram>

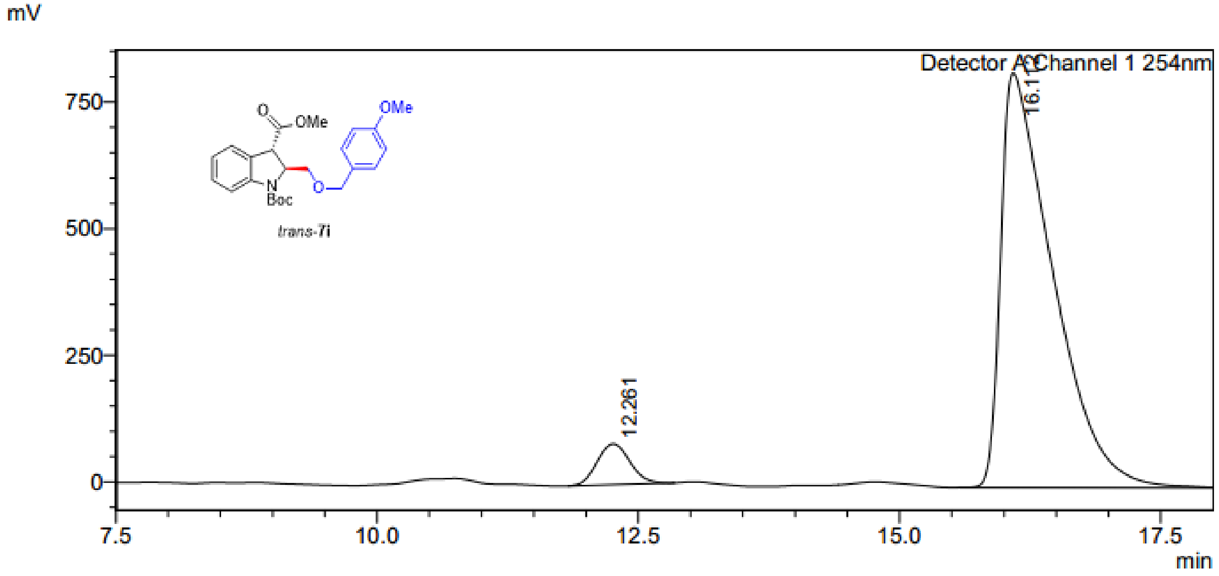

<Peak Table>

Detector A Channel 1 254nm

Peak#	Ret. Time	Area	Height	Area%
1	12.261	1767002	77324	5.859
2	16.112	28390622	798296	94.141
Total		30157623	875619	100.000

Supplementary Figure 214. HPLC spectra of *trans*-**7i**

<Chromatogram>

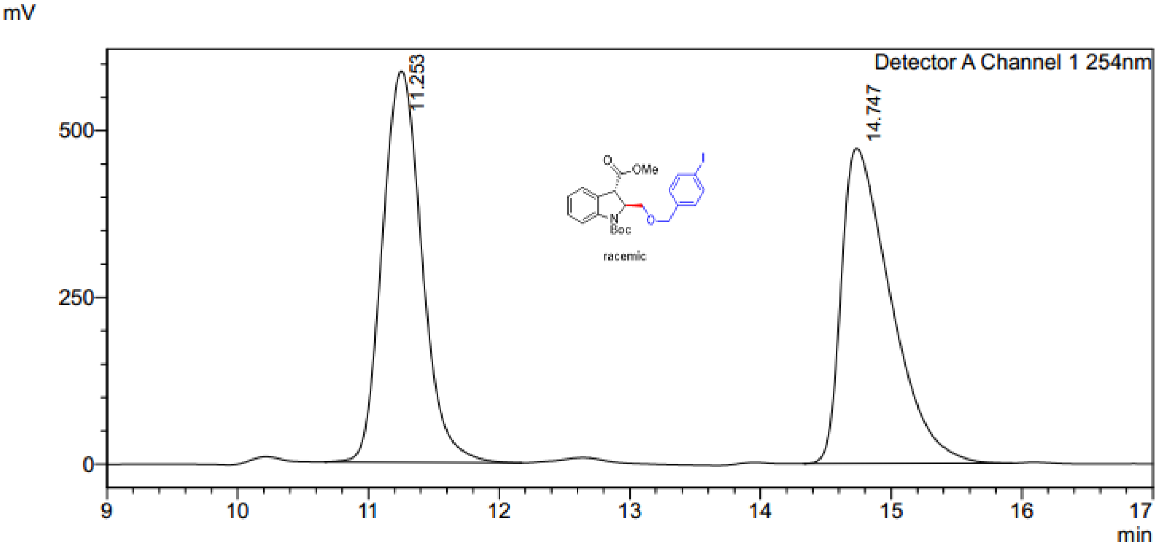

<Peak Table>

Detector A Channel 1 254nm

Peak#	Ret. Time	Area	Height	Area%
1	11.253	12346261	554622	49.876
2	14.747	12407627	458946	50.124
Total		24753888	1013568	100.000

Supplementary Figure 215. HPLC spectra of racemic *trans*-**7j**

<Chromatogram>

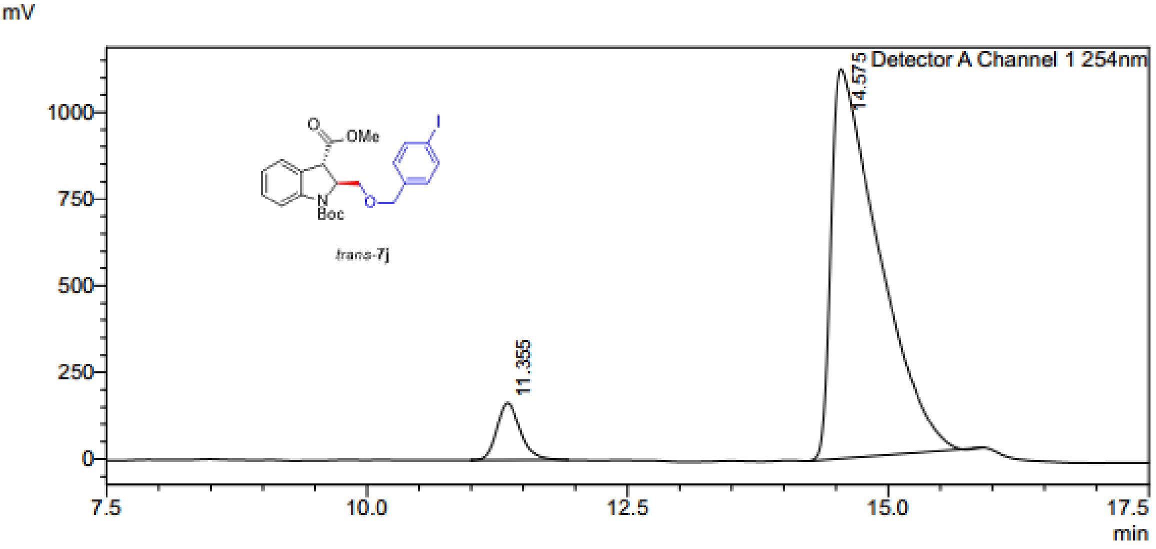

<Peak Table>

Detector A Channel 1 254nm

Peak#	Ret. Time	Area	Height	Area%
1	11.355	2572034	153345	6.689
2	14.575	35877338	1105218	93.311
Total		38449371	1258562	100.000

Supplementary Figure 216. HPLC spectra of *trans*-**7j**

<Chromatogram>

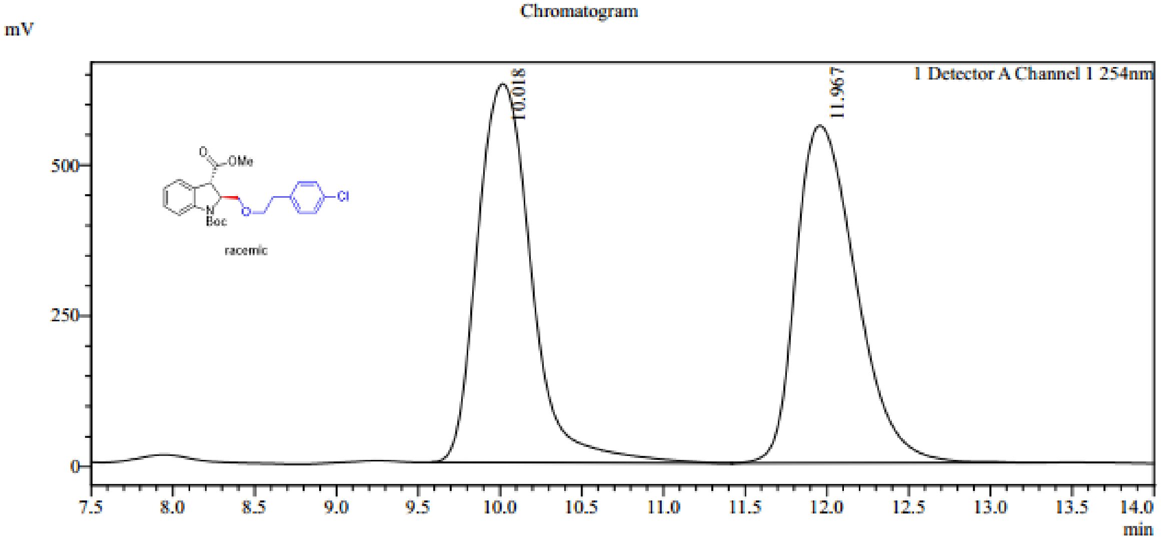

<Peak Table>

Detector A Channel 1 254nm

Peak#	Ret. Time	Area	Height	Area%
1	10.018	14472954	612732	50.234
2	11.967	14338212	554948	49.766
Total		28811166	1167680	100.000

Supplementary Figure 217. HPLC spectra of racemic *trans*-**7k**

<Chromatogram>

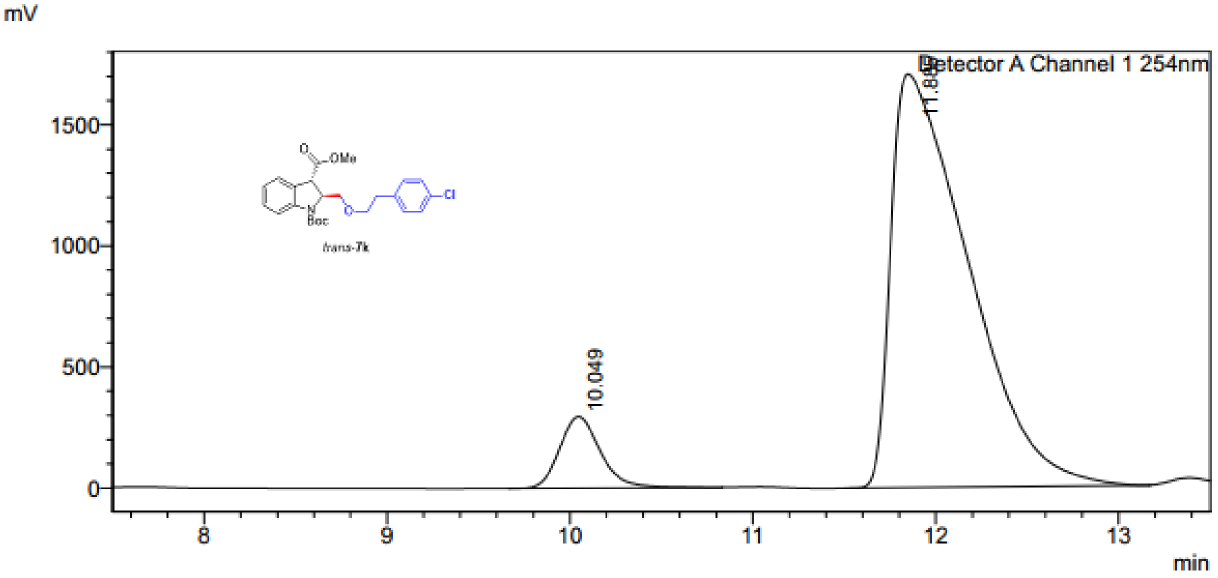

<Peak Table>

Detector A Channel 1 254nm

Peak#	Ret. Time	Area	Height	Area%
1	10.049	4594102	286314	8.386
2	11.885	50186919	1676772	91.614
Total		54781021	1963086	100.000

Supplementary Figure 218. HPLC spectra of *trans*-**7k**

<Chromatogram>

mV

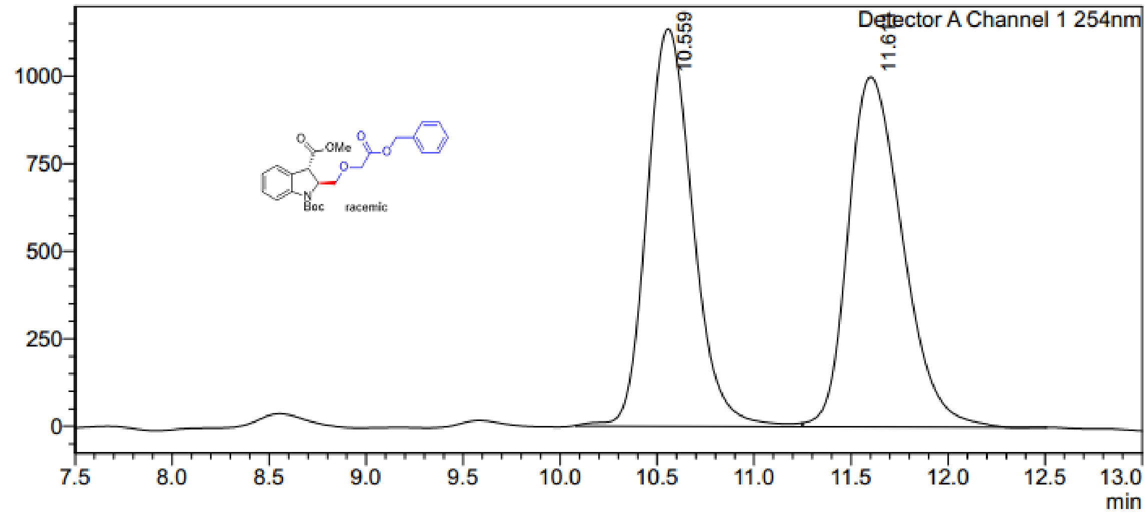

<Peak Table>

Detector A Channel 1 254nm

Peak#	Ret. Time	Area	Height	Area%
1	10.559	18811957	1103671	49.057
2	11.611	19534883	966660	50.943
Total		38346840	2070331	100.000

Supplementary Figure 219. HPLC spectra of racemic *trans*-**7l**

<Chromatogram>

mV

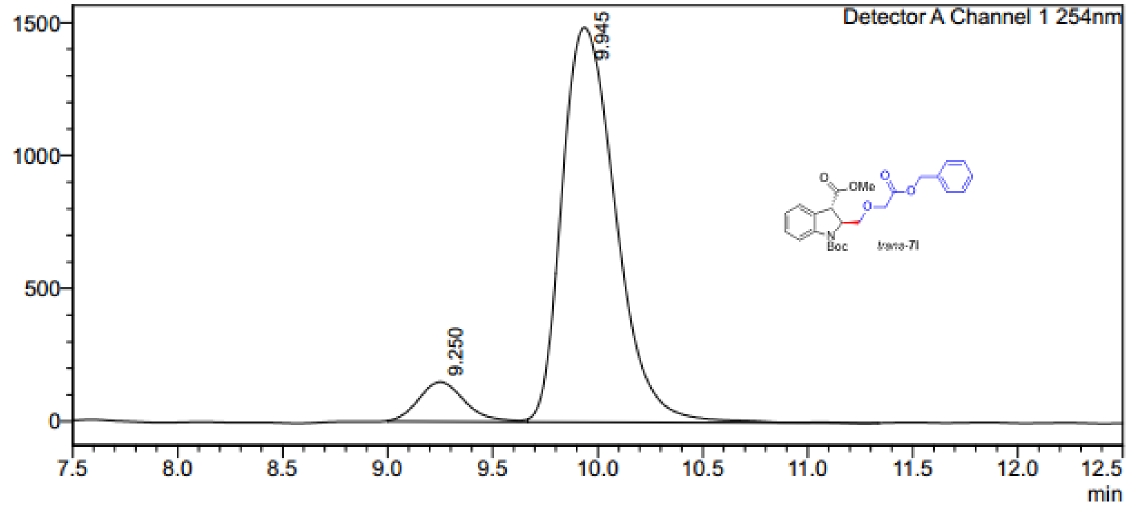

<Peak Table>

Detector A Channel 1 254nm

Peak#	Ret. Time	Area	Height	Area%
1	9.250	2292208	135139	7.959
2	9.945	26507915	1433795	92.041
Total		28800122	1568934	100.000

Supplementary Figure 220. HPLC spectra of *trans*-**7l**

<Chromatogram>

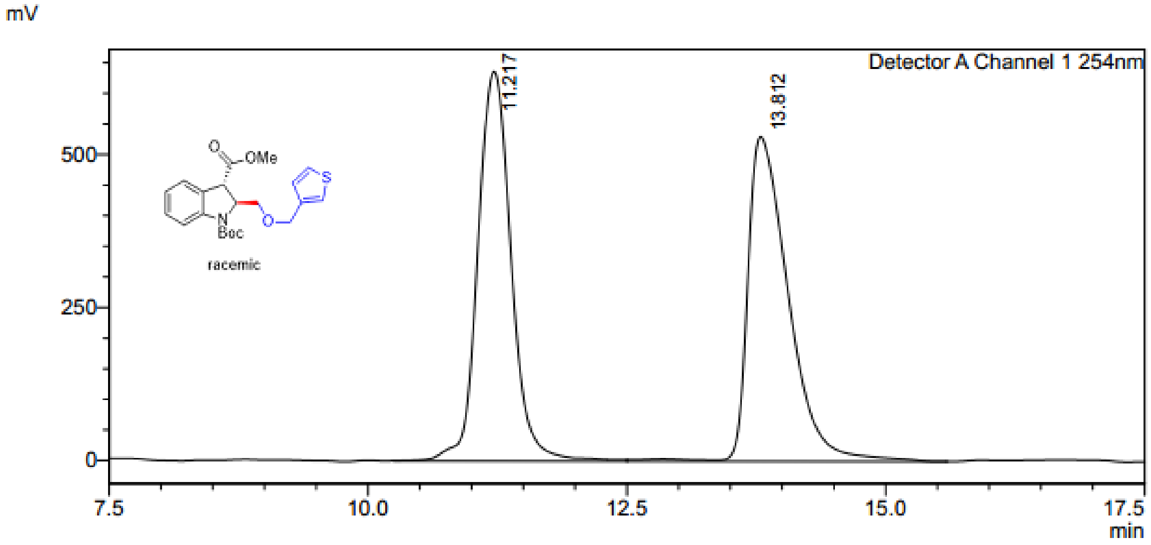

<Peak Table>

Detector A Channel 1 254nm

Peak#	Ret. Time	Area	Height	Area%
1	11.217	14272081	628588	49.699
2	13.812	14445183	525192	50.301
Total		28717264	1153781	100.000

Supplementary Figure 221. HPLC spectra of racemic *trans*-**7m**

<Chromatogram>

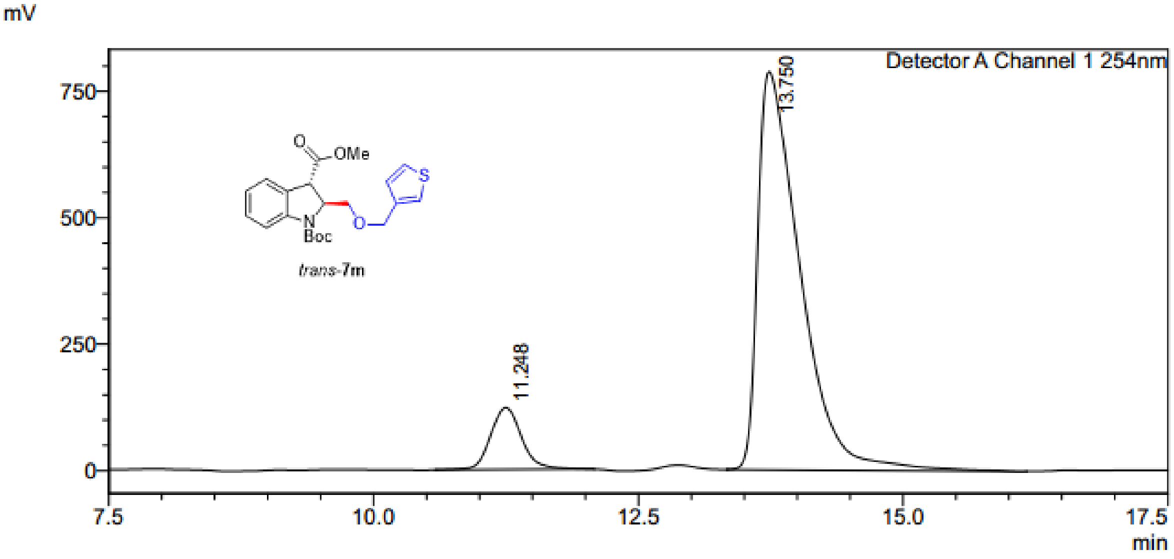

<Peak Table>

Detector A Channel 1 254nm

Peak#	Ret. Time	Area	Height	Area%
1	11.248	2501986	116110	10.275
2	13.750	21848952	760632	89.725
Total		24350937	876742	100.000

Supplementary Figure 222. HPLC spectra of *trans*-**7m**

<Chromatogram>

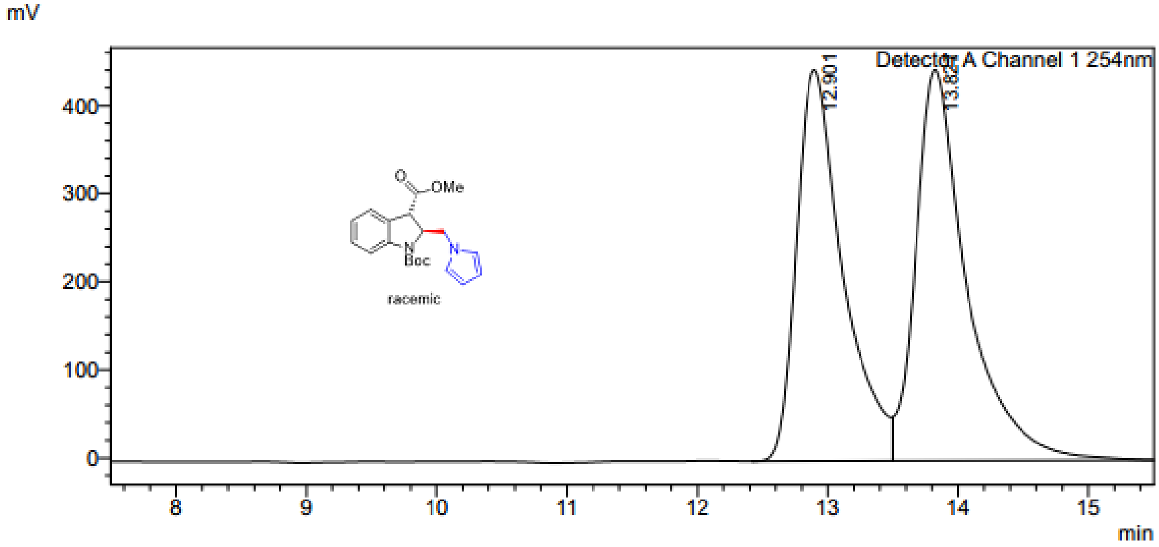

<Peak Table>

Detector A Channel 1 254nm

Peak#	Ret. Time	Area	Height	Area%
1	12.901	10991978	434231	47.644
2	13.827	12078936	429063	52.356
Total		23070914	863295	100.000

Supplementary Figure 223. HPLC spectra of racemic *trans*-**7n**

<Chromatogram>

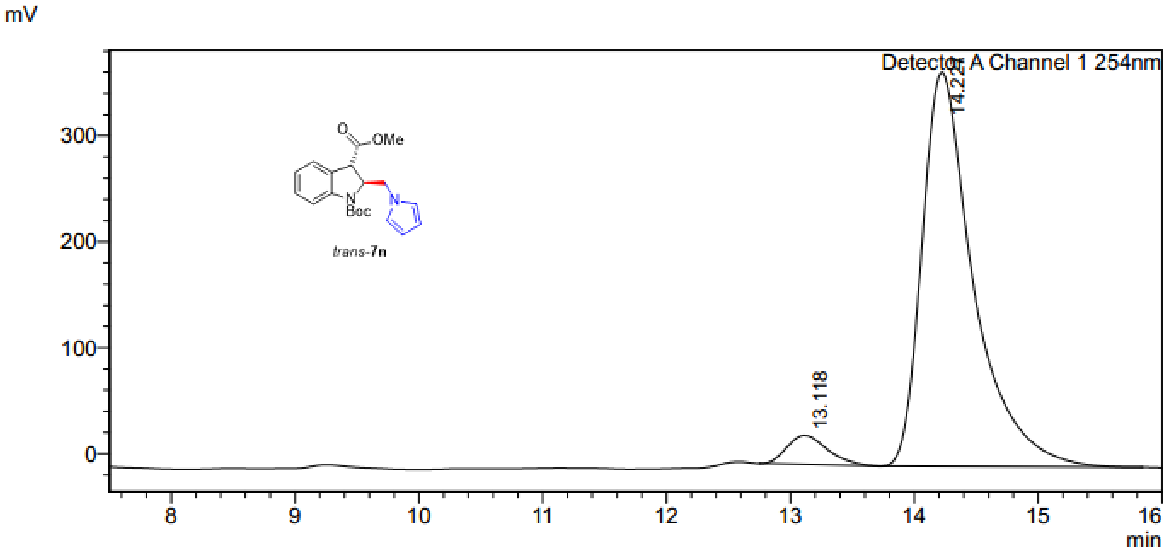

<Peak Table>

Detector A Channel 1 254nm

Peak#	Ret. Time	Area	Height	Area%
1	13.118	615432	26747	5.401
2	14.227	10778801	367070	94.599
Total		11394233	393818	100.000

Supplementary Figure 224. HPLC spectra of *trans*-**7n**

<Chromatogram>

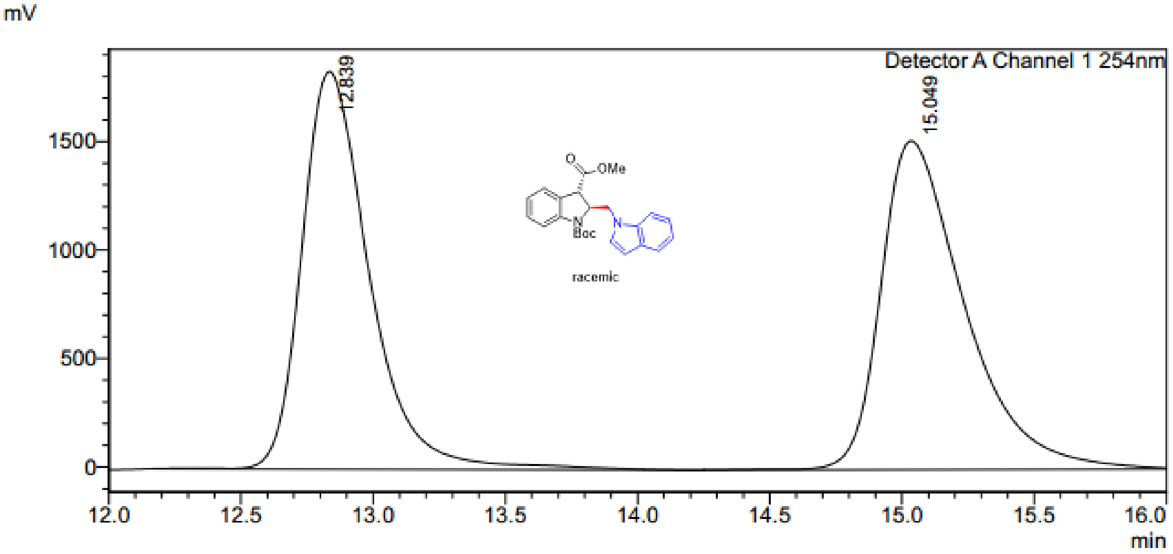

<Peak Table>

Detector A Channel 1 254nm

Peak#	Ret. Time	Area	Height	Area%
1	12.839	32669412	1676630	49.382
2	15.049	33487397	1487741	50.618
Total		66156809	3164371	100.000

Supplementary Figure 225. HPLC spectra of racemic *trans*-**7o**

<Chromatogram>

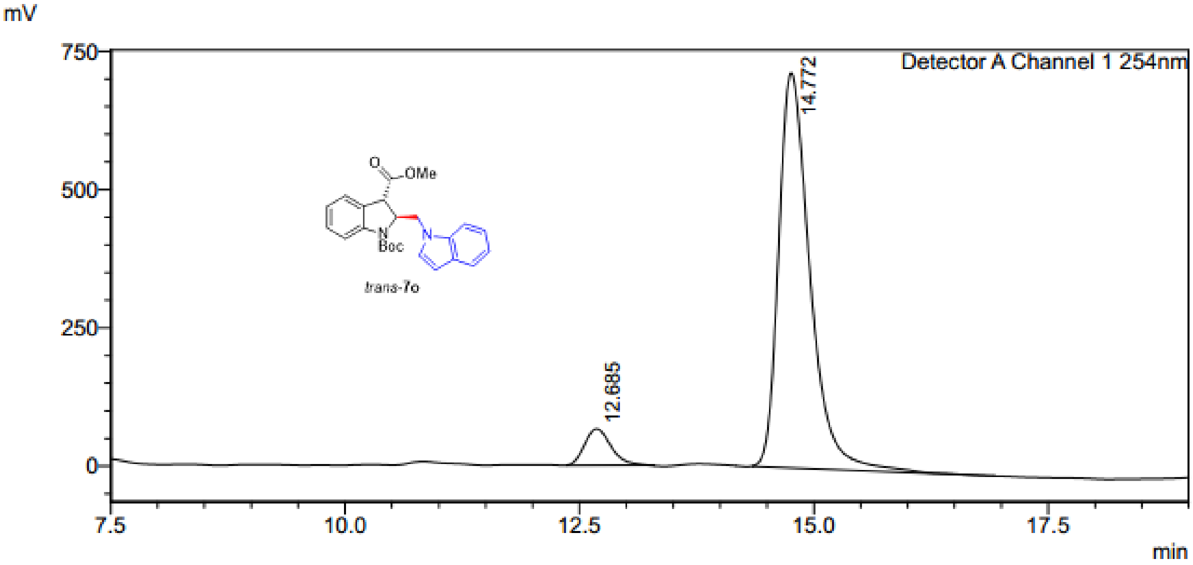

<Peak Table>

Detector A Channel 1 254nm

Peak#	Ret. Time	Area	Height	Area%
1	12.685	1293338	62798	7.215
2	14.772	16632935	694863	92.785
Total		17926273	757661	100.000

Supplementary Figure 226. HPLC spectra of *trans*-**7o**

<Chromatogram>

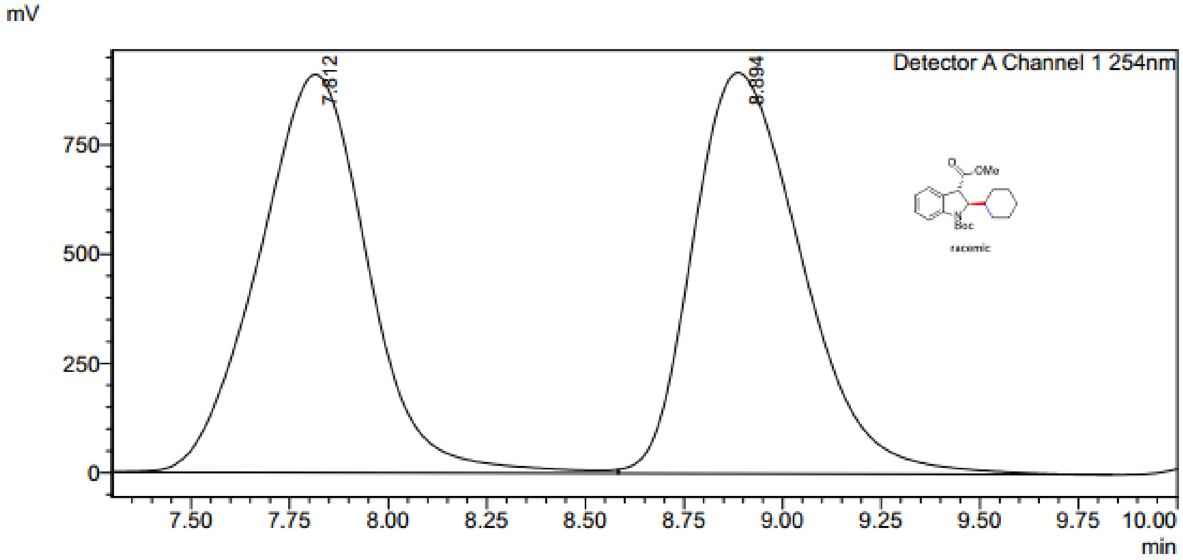

<Peak Table>

Detector A Channel 1 254nm

Peak#	Ret. Time	Area	Height	Area%
1	7.812	17561538	882282	49.968
2	8.894	17584174	898430	50.032
Total		35145712	1780712	100.000

Supplementary Figure 227. HPLC spectra of racemic *trans*-**7p**

<Chromatogram>

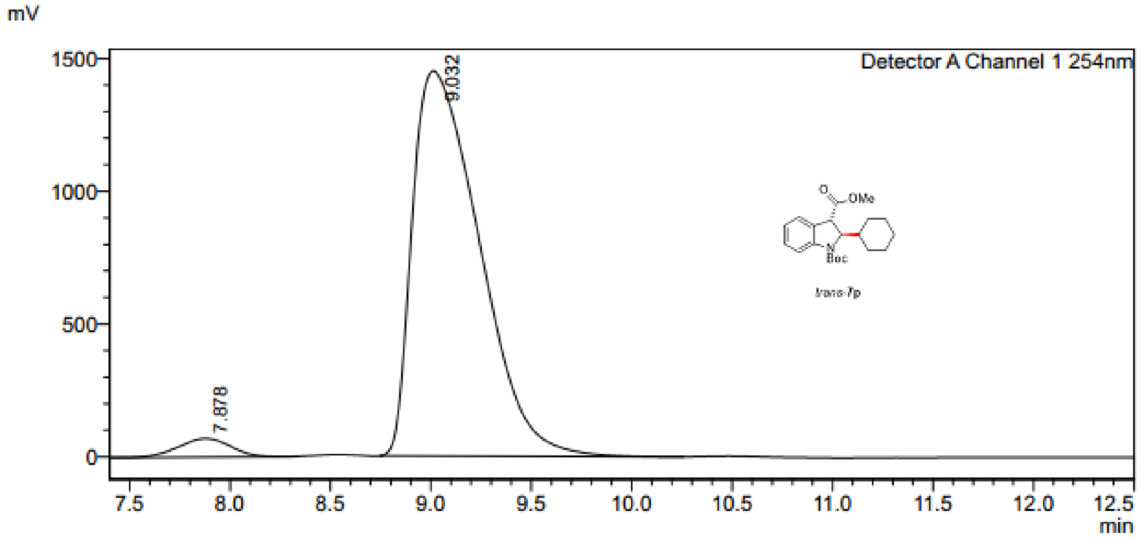

<Peak Table>

Detector A Channel 1 254nm

Peak#	Ret. Time	Area	Height	Area%
1	7.878	1246810	67703	3.575
2	9.032	33629020	1413315	96.425
Total		34875830	1481018	100.000

Supplementary Figure 228. HPLC spectra of *trans*-**7p**

<Chromatogram>

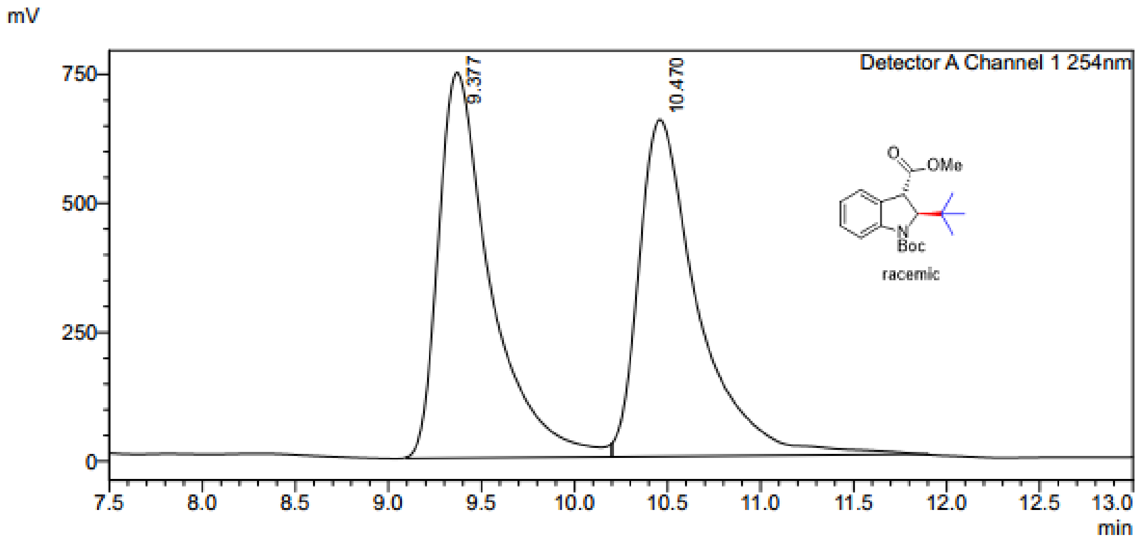

<Peak Table>

Detector A Channel 1 254nm

Peak#	Ret. Time	Area	Height	Area%
1	9.377	14571112	711673	50.322
2	10.470	14384876	632993	49.678
Total		28955988	1344666	100.000

Supplementary Figure 229. HPLC spectra of racemic *trans*-**7q**

<Chromatogram>

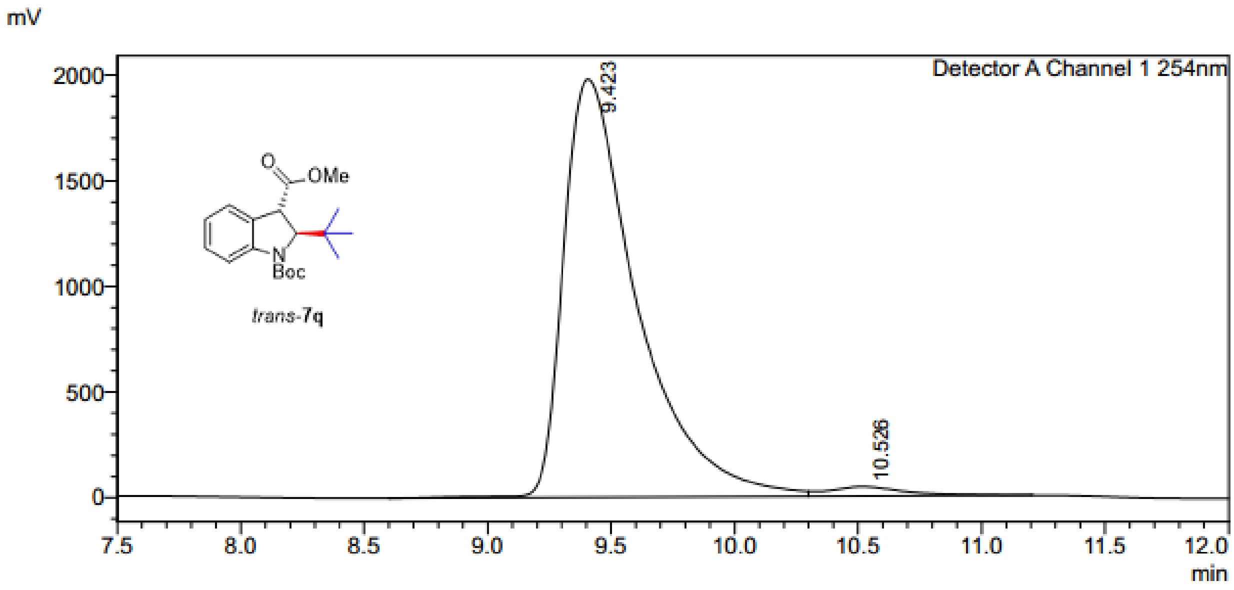

<Peak Table>

Detector A Channel 1 254nm

Peak#	Ret. Time	Area	Height	Area%
1	9.423	40918894	1825135	97.787
2	10.526	926127	40588	2.213
Total		41845021	1865722	100.000

Supplementary Figure 230. HPLC spectra of *trans*-**7q**

<Chromatogram>

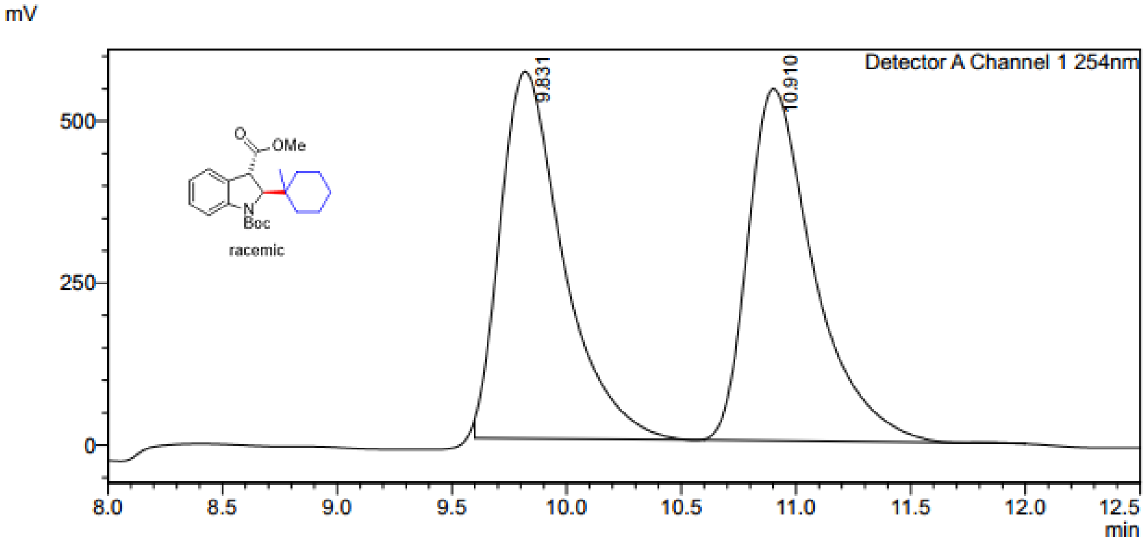

<Peak Table>

Detector A Channel 1 254nm

Peak#	Ret. Time	Area	Height	Area%
1	9.831	10804951	530567	49.804
2	10.910	10890038	490741	50.196
Total		21694989	1021308	100.000

Supplementary Figure 231. HPLC spectra of racemic *trans*-**7r**

<Chromatogram>

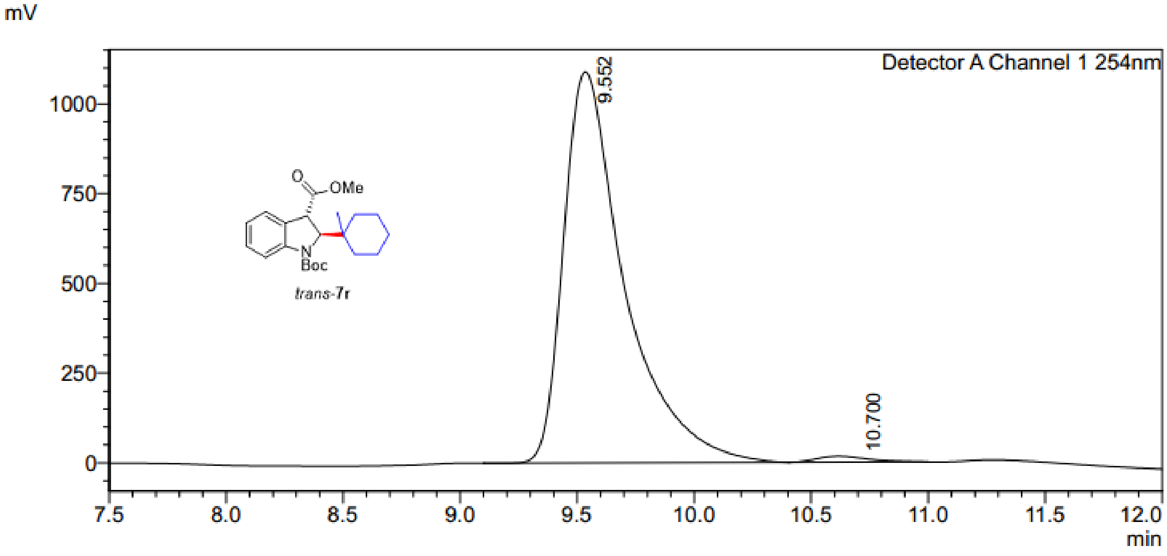

<Peak Table>

Detector A Channel 1 254nm

Peak#	Ret. Time	Area	Height	Area%
1	9.552	19683434	1036886	98.843
2	10.700	230357	14197	1.157
Total		19913790	1051083	100.000

Supplementary Figure 232. HPLC spectra of *trans*-**7r**

<Chromatogram>

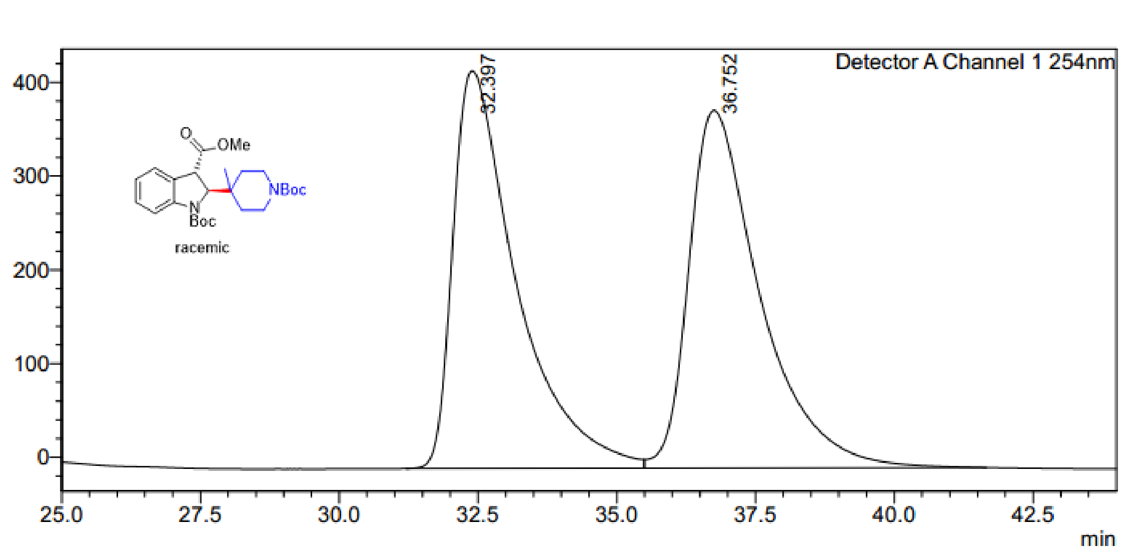

<Peak Table>

Detector A Channel 1 254nm

Peak#	Ret. Time	Area	Height	Area%
1	32.397	33652968	423955	49.628
2	36.752	34157261	381521	50.372
Total		67810229	805477	100.000

Supplementary Figure 233. HPLC spectra of racemic *trans*-**7s**

<Chromatogram>

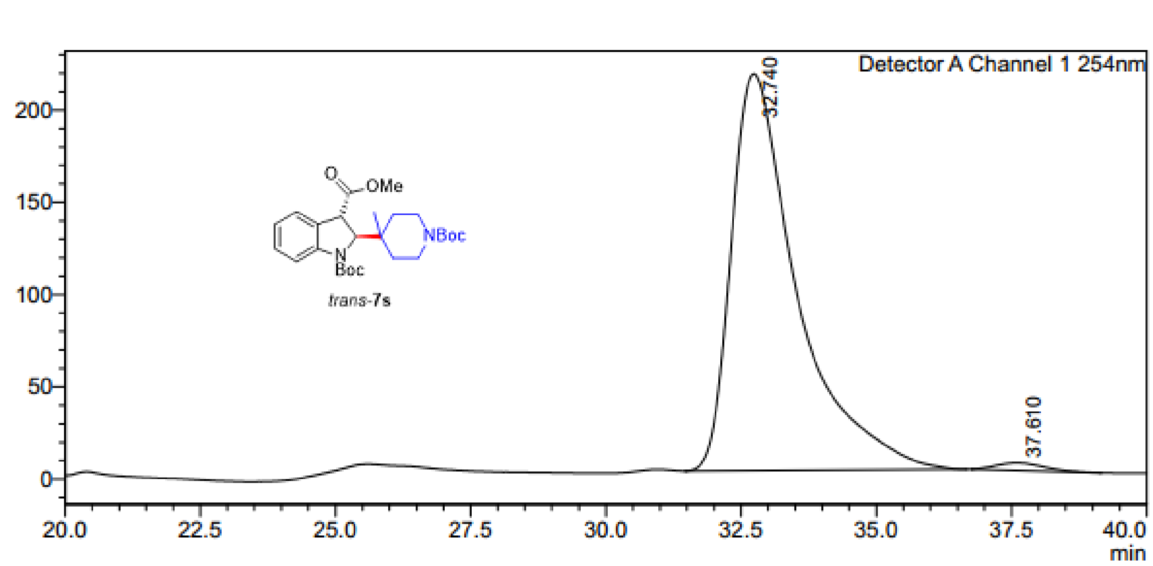

<Peak Table>

Detector A Channel 1 254nm

Peak#	Ret. Time	Area	Height	Area%
1	32.740	18150598	214909	98.661
2	37.610	246282	4044	1.339
Total		18396880	218953	100.000

Supplementary Figure 234. HPLC spectra of *trans*-**7s**

<Chromatogram>
mV

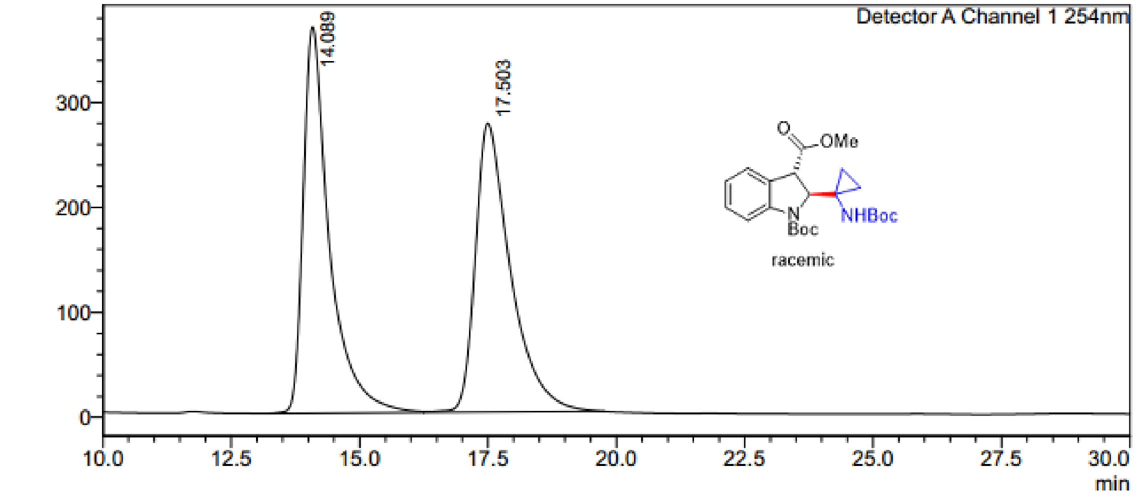

<Peak Table>

Detector A Channel 1 254nm

Peak#	Ret. Time	Area	Height	Conc.	Area%
1	14.089	13064704	356651	49.863	49.863
2	17.503	13136589	270590	50.137	50.137
Total		26201292	627241		100.000

Supplementary Figure 235. HPLC spectra of racemic *trans*-**7t**

<Chromatogram>
mV

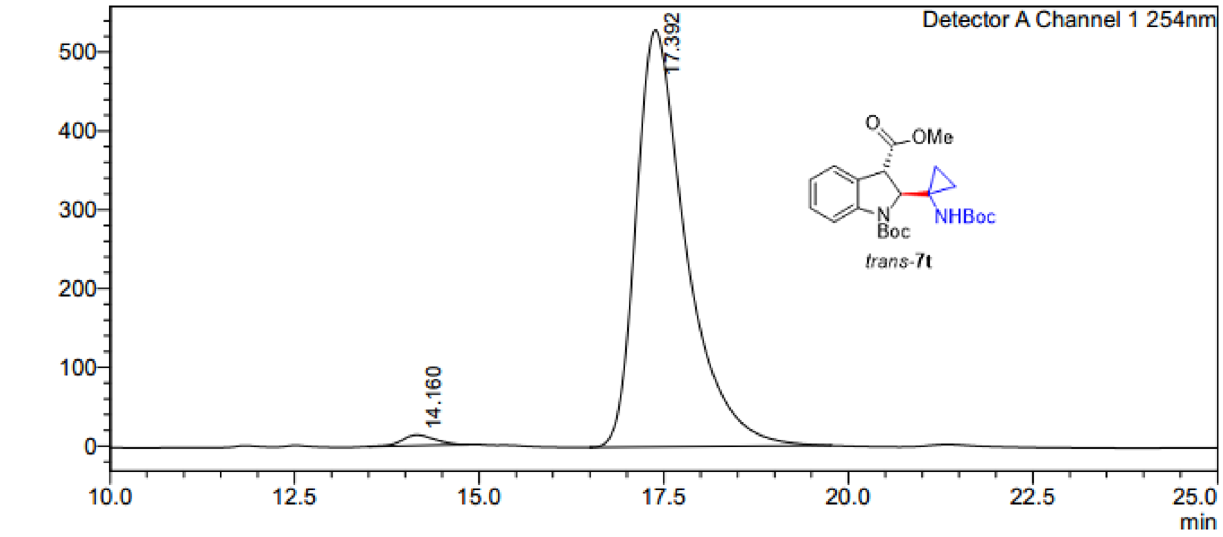

<Peak Table>

Detector A Channel 1 254nm

Peak#	Ret. Time	Area	Height	Area%
1	14.160	411352	13137	1.624
2	17.392	24921732	526448	98.376
Total		25333084	539585	100.000

Supplementary Figure 236. HPLC spectra of *trans*-**7t**

<Chromatogram>

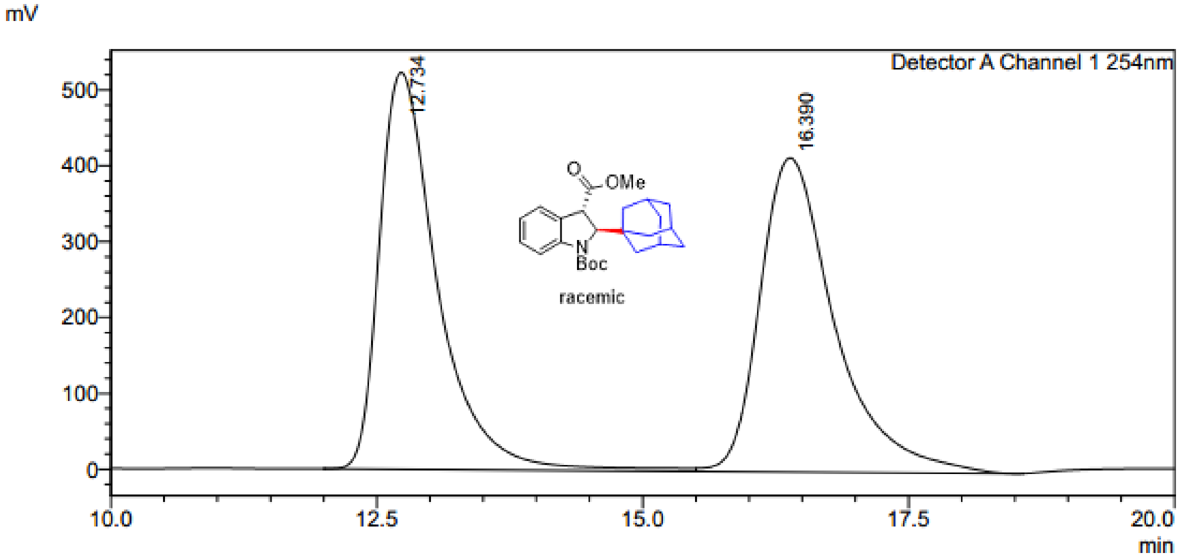

<Peak Table>

Detector A Channel 1 254nm

Peak#	Ret. Time	Area	Height	Area%
1	12.734	19933070	518623	49.589
2	16.390	20263332	412281	50.411
Total		40196403	930904	100.000

Supplementary Figure 237. HPLC spectra of racemic *trans*-**7u**

<Chromatogram>

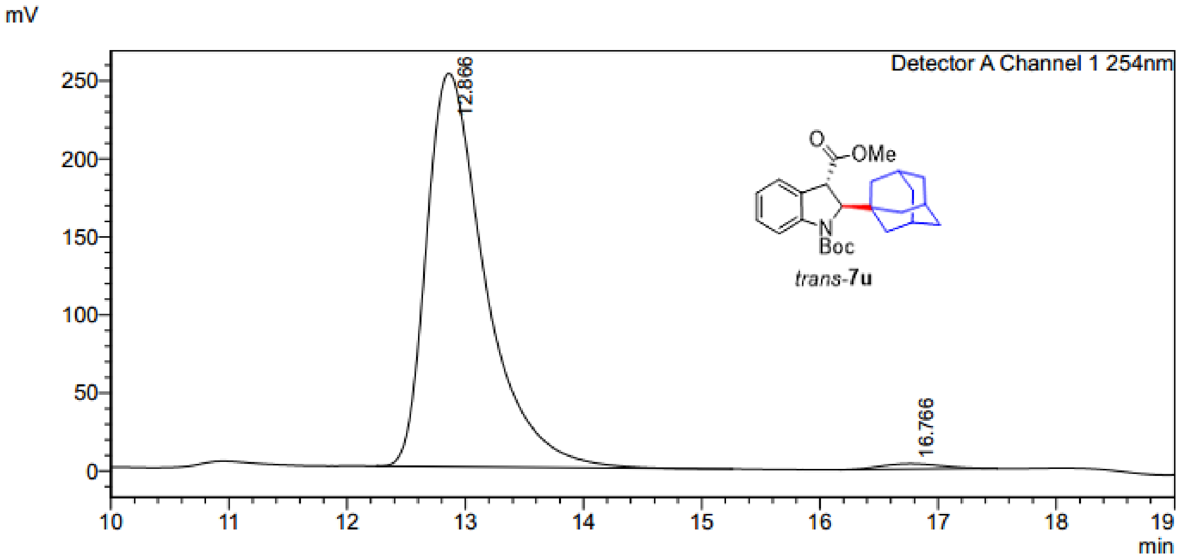

<Peak Table>

Detector A Channel 1 254nm

Peak#	Ret. Time	Area	Height	Area%
1	12.866	8679467	249463	98.544
2	16.766	128222	3445	1.456
Total		8807689	252908	100.000

Supplementary Figure 238. HPLC spectra of *trans*-**7u**

<Chromatogram>

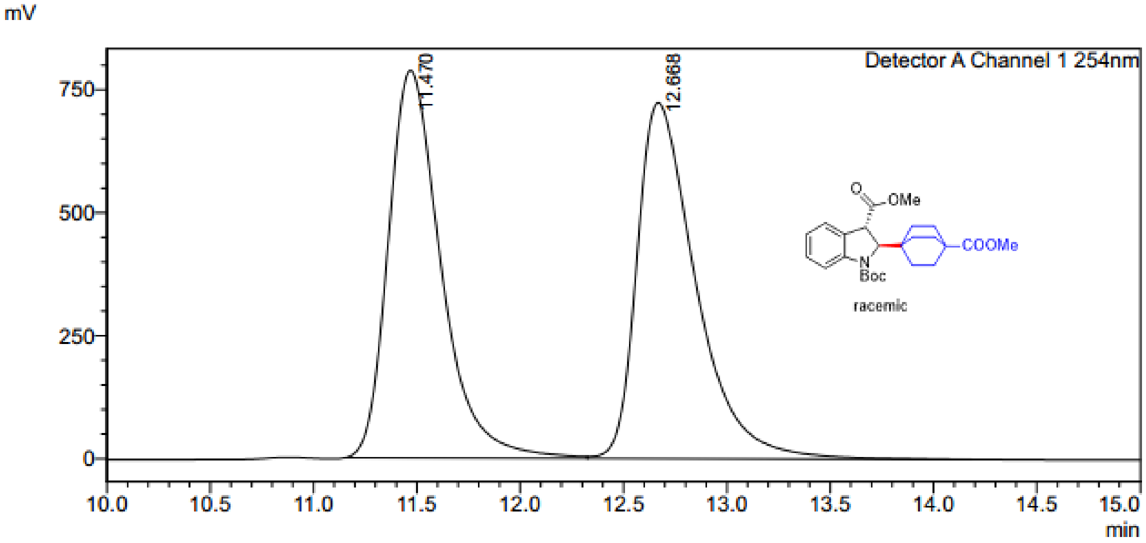

<Peak Table>

Detector A Channel 1 254nm

Peak#	Ret. Time	Area	Height	Area%
1	11.470	13981040	787983	49.348
2	12.668	14350543	723729	50.652
Total		28331583	1511711	100.000

Supplementary Figure 239. HPLC spectra of racemic *trans*-**7v**

<Chromatogram>

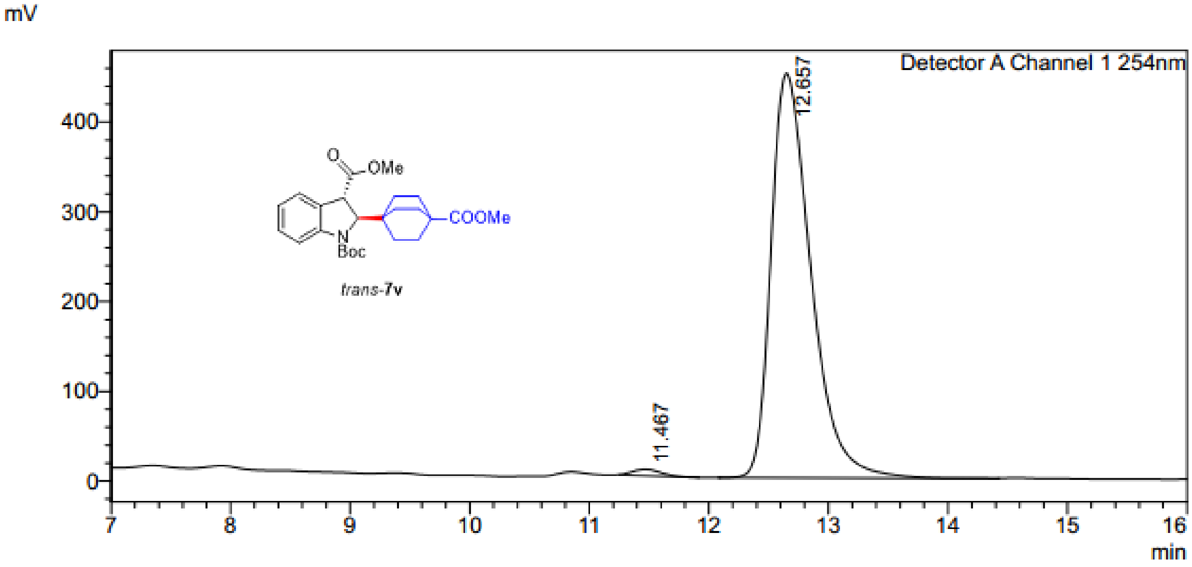

<Peak Table>

Detector A Channel 1 254nm

Peak#	Ret. Time	Area	Height	Area%
1	11.467	115934	6937	1.097
2	12.657	10457003	439212	98.903
Total		10572937	446148	100.000

Supplementary Figure 240. HPLC spectra of *trans*-**7v**

<Chromatogram>

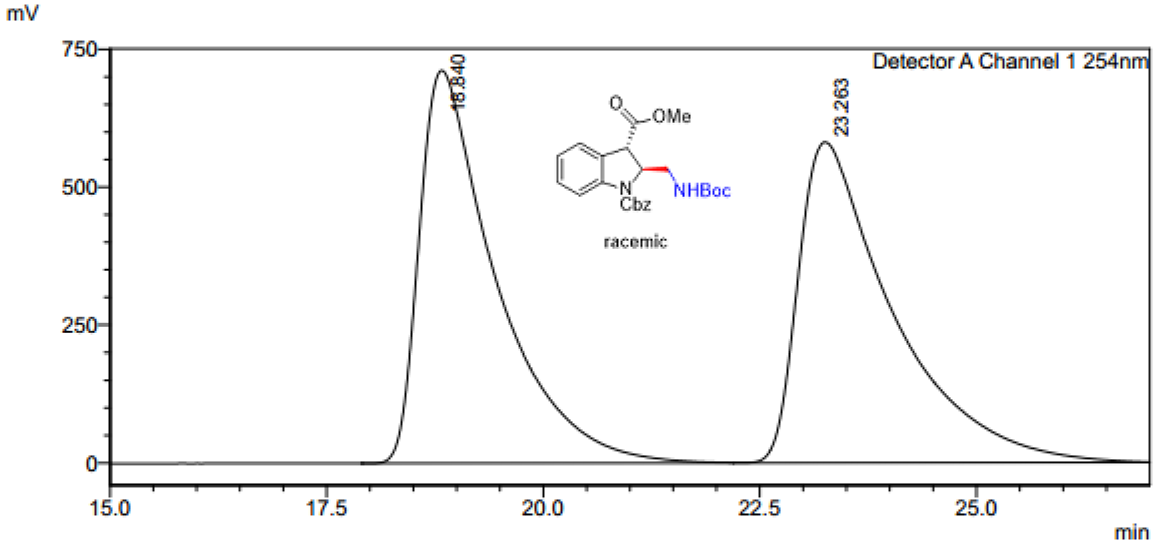

<Peak Table>

Detector A Channel 1 254nm

Peak#	Ret. Time	Area	Height	Area%
1	18.840	43703642	707298	49.808
2	23.263	44040060	579635	50.192
Total		87743701	1286933	100.000

Supplementary Figure 241. HPLC spectra of racemic *trans*-**7w**

<Chromatogram>

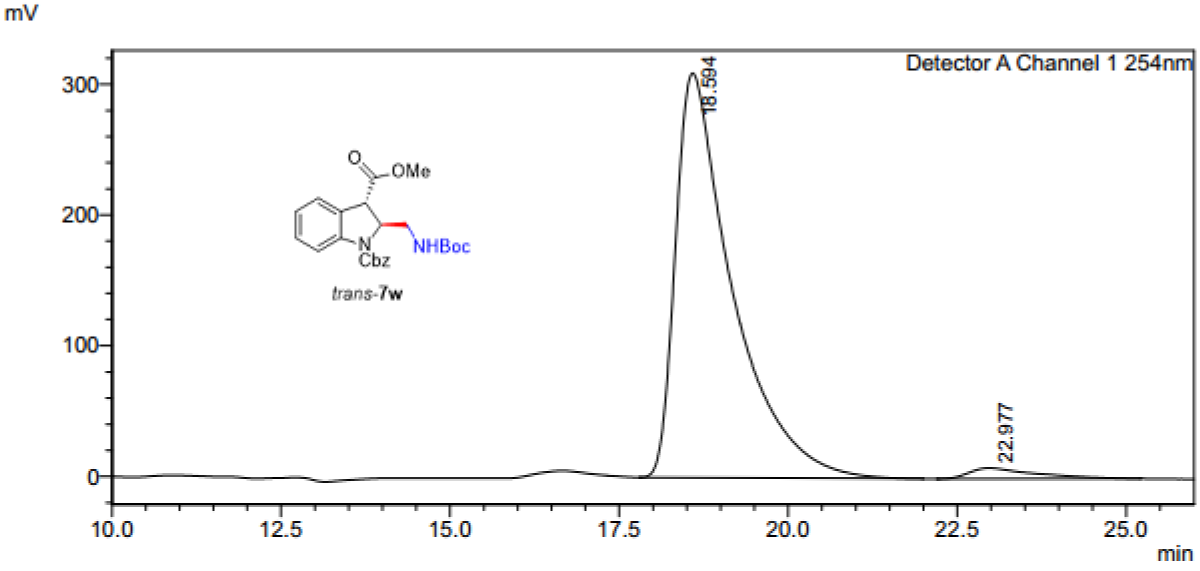

<Peak Table>

Detector A Channel 1 254nm

Peak#	Ret. Time	Area	Height	Area%
1	18.594	18060706	305361	97.047
2	22.977	549480	8128	2.953
Total		18610186	313489	100.000

Supplementary Figure 242. HPLC spectra of *trans*-**7w**

<Chromatogram>

mV

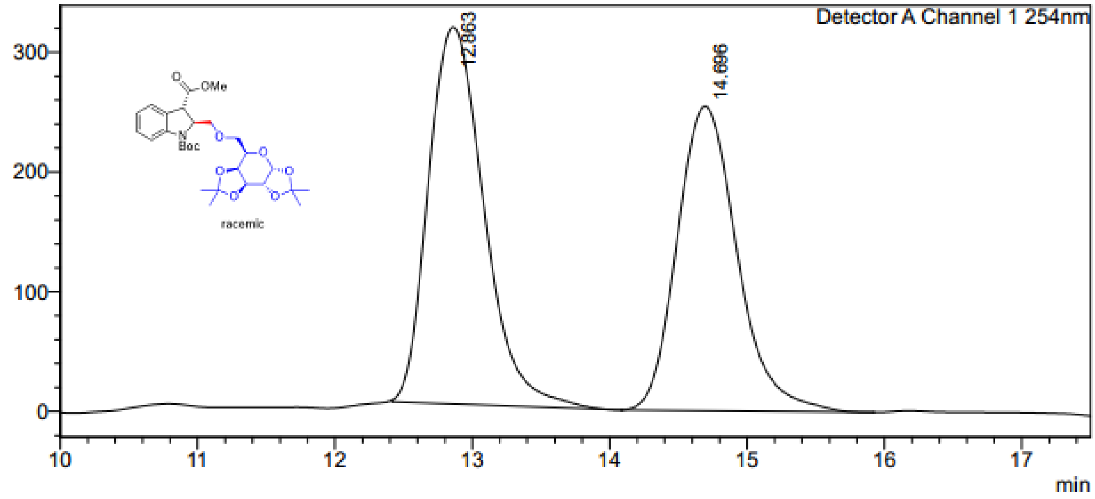

<Peak Table>

Detector A Channel 1 254nm

Peak#	Ret. Time	Area	Height	Area%
1	12.863	8852908	310617	53.403
2	14.696	7724531	251416	46.597
Total		16577439	562033	100.000

Supplementary Figure 243. HPLC spectra of racemic *trans*-**7x**

<Chromatogram>

mV

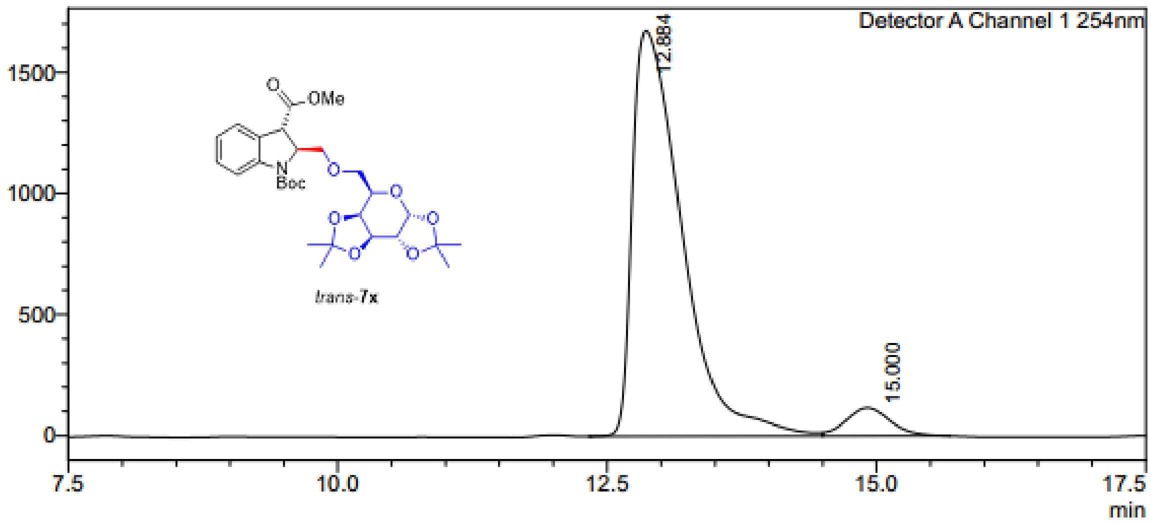

<Peak Table>

Detector A Channel 1 254nm

Peak#	Ret. Time	Area	Height	Area%
1	12.884	53511366	1659789	94.219
2	15.000	3283537	111474	5.781
Total		56794903	1771263	100.000

Supplementary Figure 244. HPLC spectra of *trans*-**7x**

<Chromatogram>

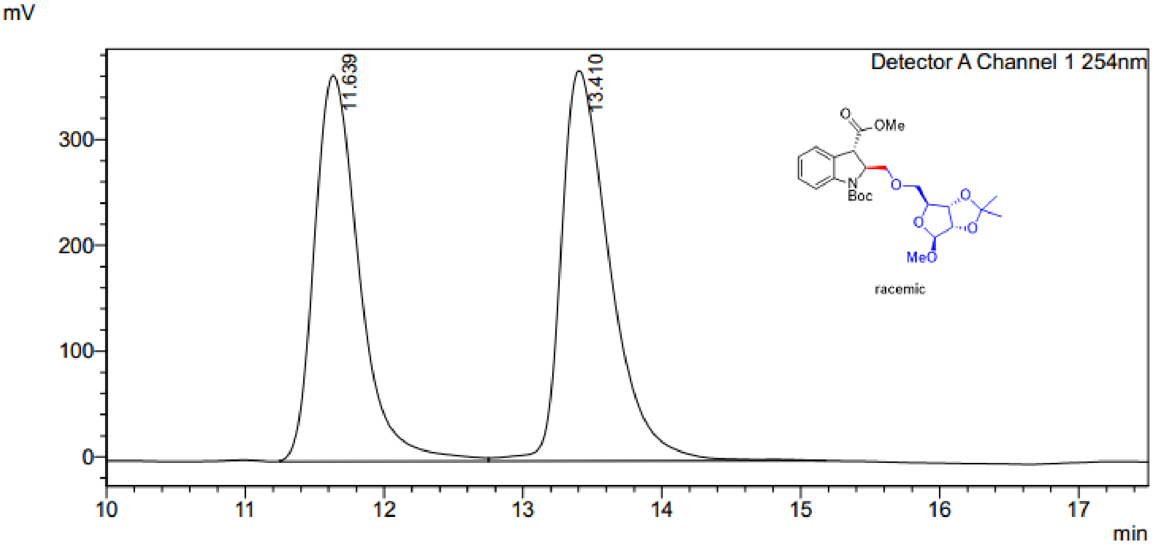

<Peak Table>

Detector A Channel 1 254nm

Peak#	Ret. Time	Area	Height	Area%
1	11.639	8201890	359643	47.663
2	13.410	9006152	357707	52.337
Total		17208043	717350	100.000

Supplementary Figure 245. HPLC spectra of racemic *trans*-**7y**

<Chromatogram>

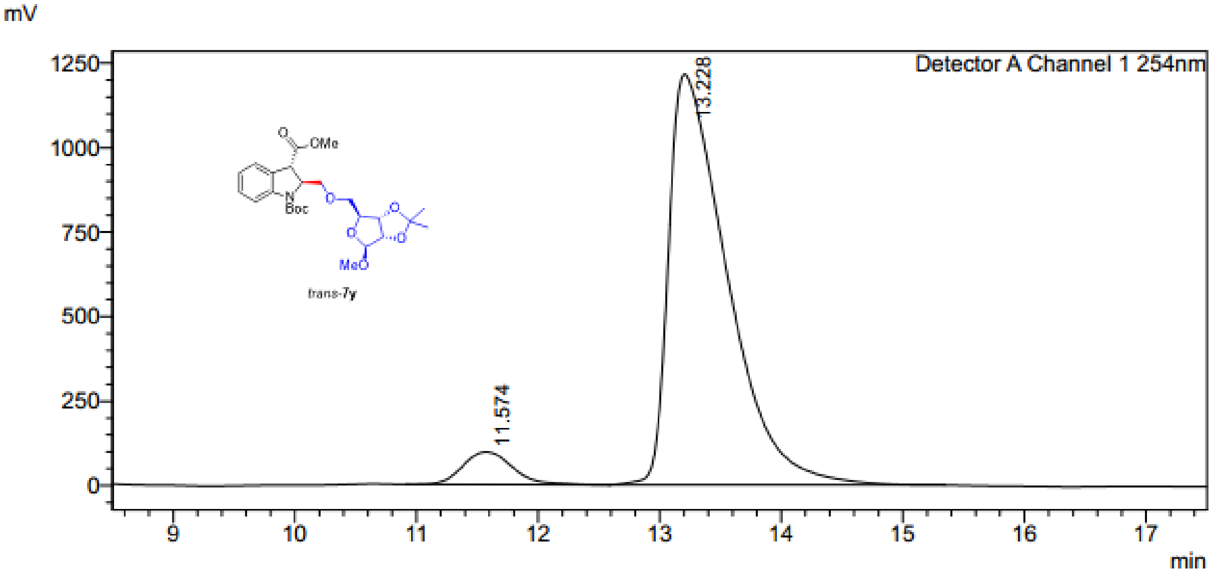

<Peak Table>

Detector A Channel 1 254nm

Peak#	Ret. Time	Area	Height	Area%
1	11.574	2774478	94933	6.381
2	13.228	40704950	1205955	93.619
Total		43479429	1300888	100.000

Supplementary Figure 246. HPLC spectra of *trans*-**7y**

<Chromatogram>

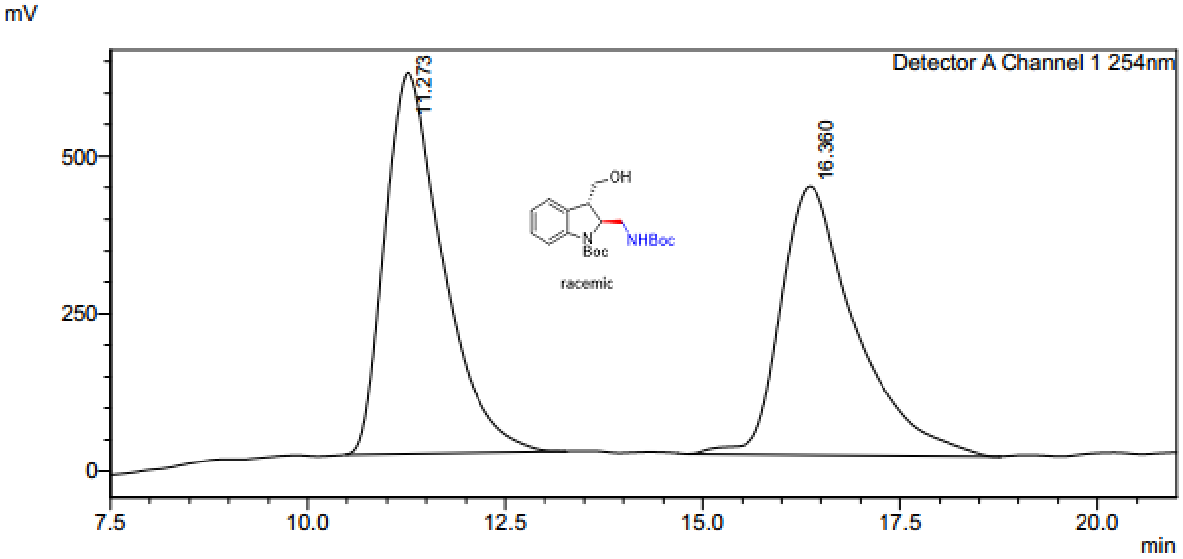

<Peak Table>

Detector A Channel 1 254nm

Peak#	Ret. Time	Area	Height	Conc.	Area%
1	11.273	30823634	599166	52.370	52.370
2	16.360	28033779	423265	47.630	47.630
Total		58857413	1022431		100.000

Supplementary Figure 247. HPLC spectra of racemic *trans*-**8a**

<Chromatogram>

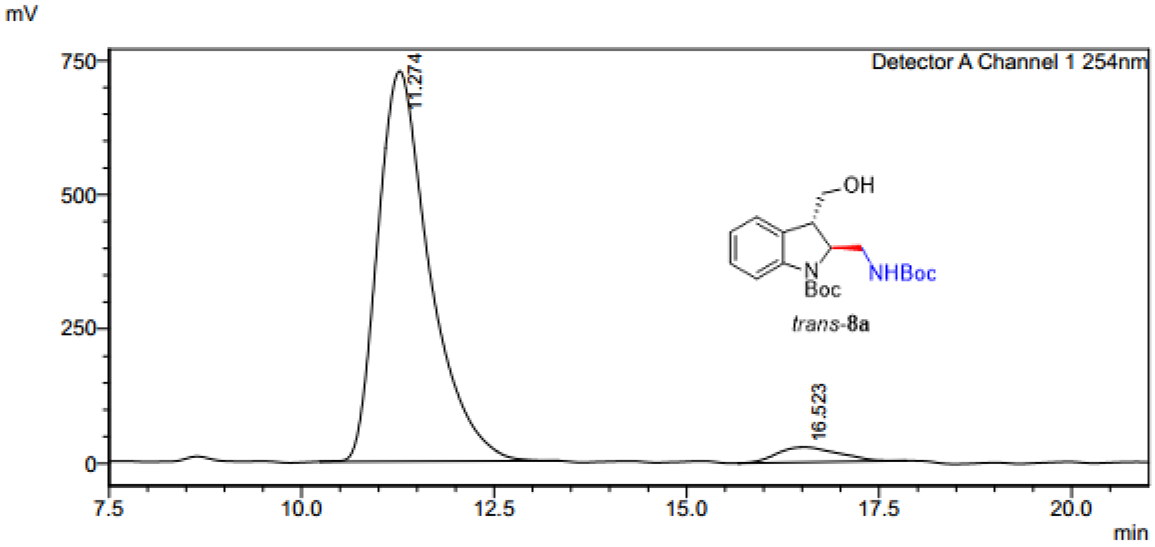

<Peak Table>

Detector A Channel 1 254nm

Peak#	Ret. Time	Area	Height	Area%
1	11.274	34088713	721356	95.631
2	16.523	1557420	28020	4.369
Total		35646133	749376	100.000

Supplementary Figure 248. HPLC spectra of *trans*-**8a**

<Chromatogram>

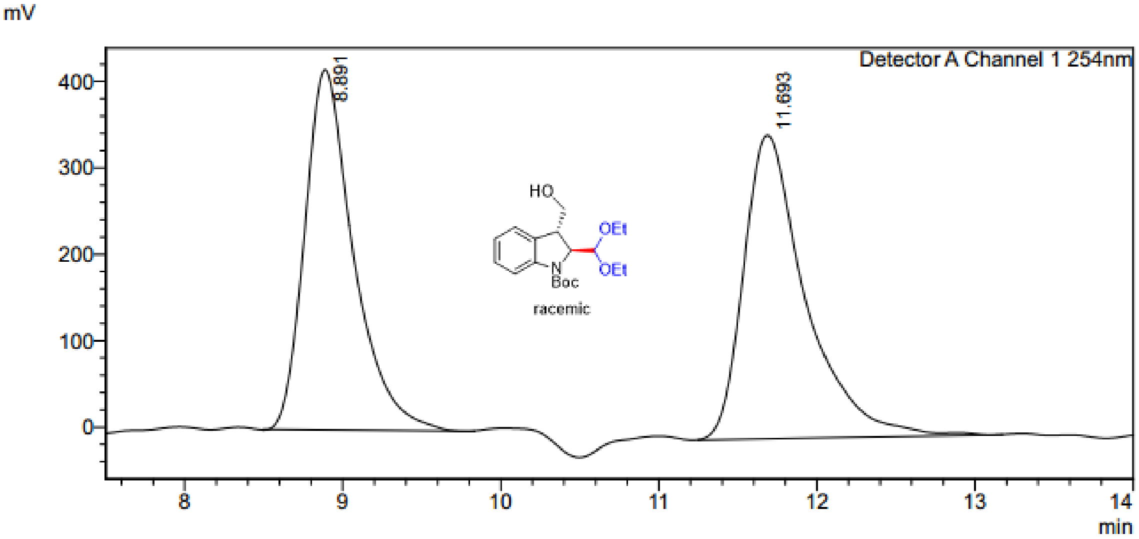

<Peak Table>

Detector A Channel 1 254nm

Peak#	Ret. Time	Area	Height	Area%
1	8.891	8939504	407953	48.230
2	11.693	9595675	343319	51.770
Total		18535179	751272	100.000

Supplementary Figure 249. HPLC spectra of racemic *trans*-**8b**

<Chromatogram>

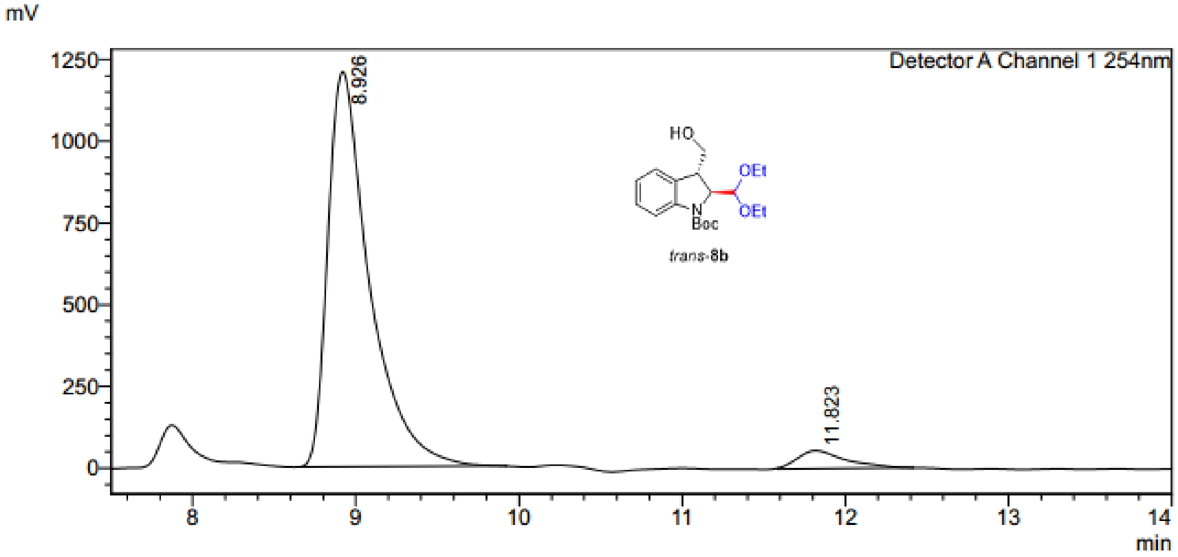

<Peak Table>

Detector A Channel 1 254nm

Peak#	Ret. Time	Area	Height	Area%
1	8.926	21523130	1097136	95.021
2	11.823	1127672	52749	4.979
Total		22650802	1149885	100.000

Supplementary Figure 250. HPLC spectra of *trans*-**8b**

<Chromatogram>

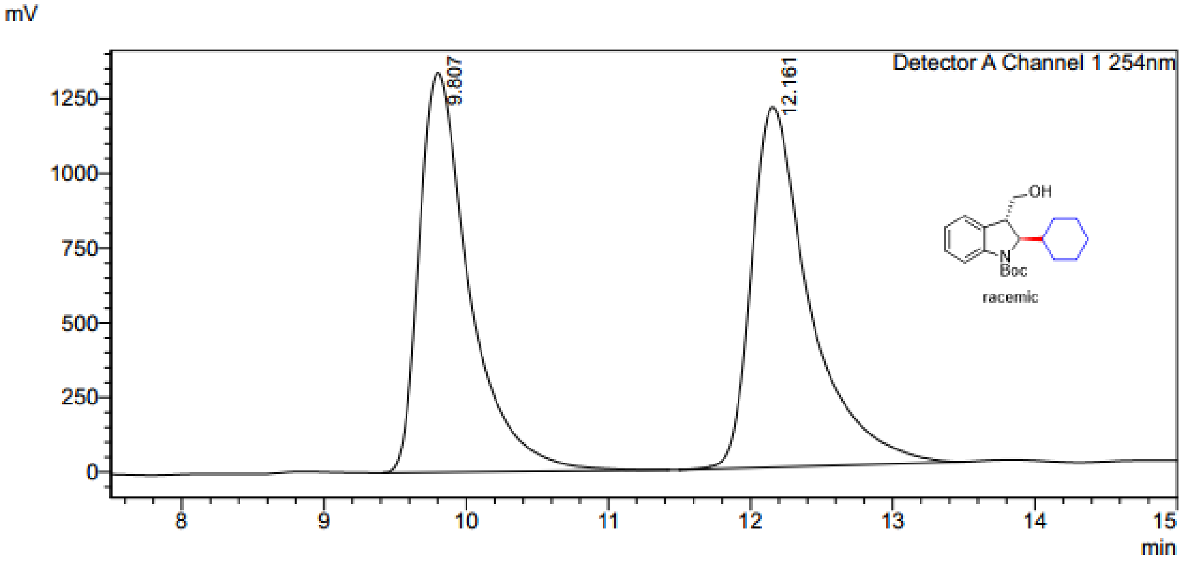

<Peak Table>

Detector A Channel 1 254nm

Peak#	Ret. Time	Area	Height	Area%
1	9.807	32860655	1317480	48.914
2	12.161	34319968	1171920	51.086
Total		67180623	2489401	100.000

Supplementary Figure 251. HPLC spectra of racemic *trans*-**8c**

<Chromatogram>

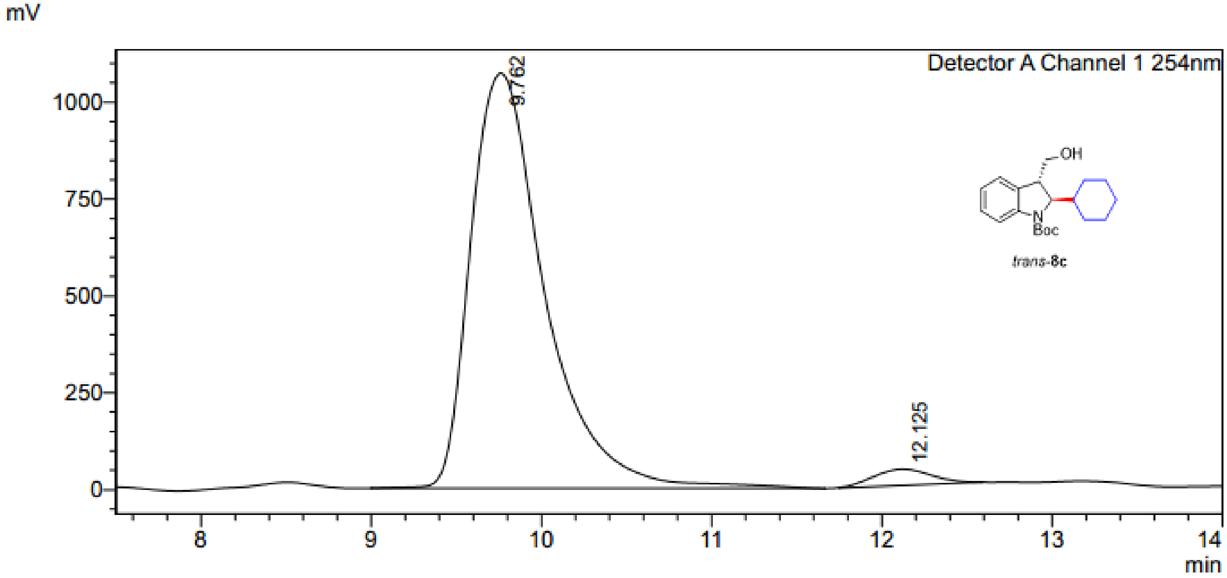

<Peak Table>

Detector A Channel 1 254nm

Peak#	Ret. Time	Area	Height	Area%
1	9.762	32504891	1048379	97.009
2	12.125	1002145	41497	2.991
Total		33507036	1089876	100.000

Supplementary Figure 252. HPLC spectra of *trans*-**8c**

<Chromatogram>

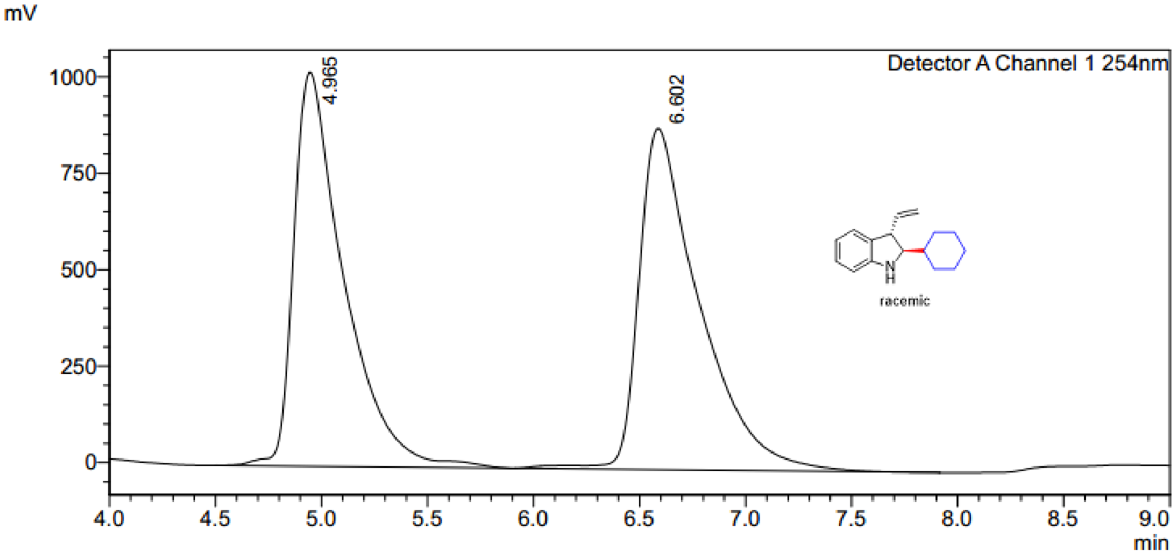

<Peak Table>

Detector A Channel 1 254nm

Peak#	Ret. Time	Area	Height	Area%
1	4.965	16776511	974366	48.750
2	6.602	17636695	815266	51.250
Total		34413205	1789631	100.000

Supplementary Figure 253. HPLC spectra of racemic *trans*-**11**

<Chromatogram>

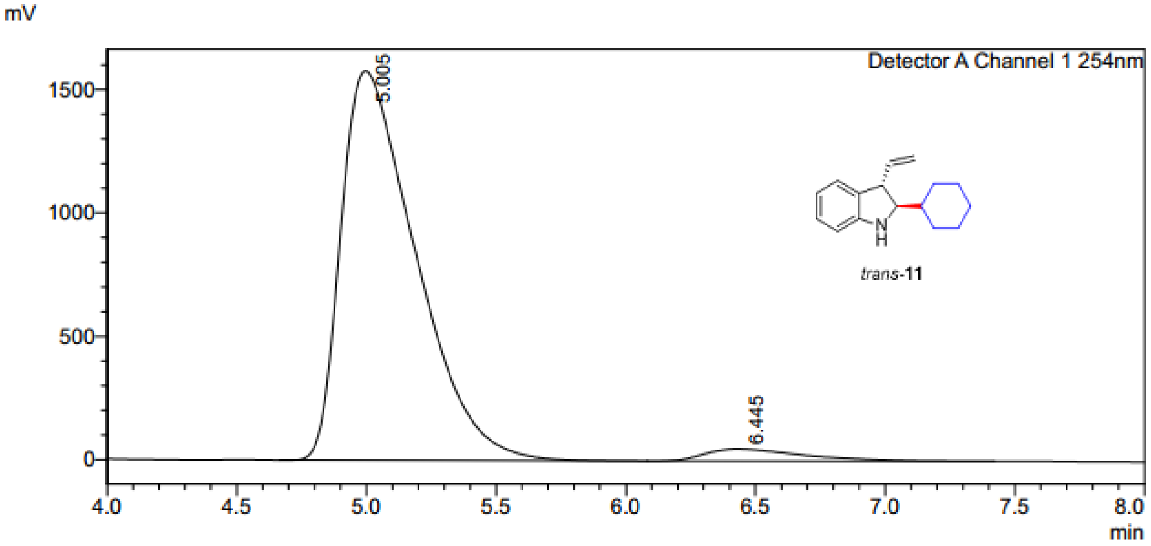

<Peak Table>

Detector A Channel 1 254nm

Peak#	Ret. Time	Area	Height	Area%
1	5.005	31728601	1465764	96.082
2	6.445	1293674	47872	3.918
Total		33022275	1513636	100.000

Supplementary Figure 254. HPLC spectra of *trans*-**11**
